# Effectiveness and safety of pharmacist prescribing: a systematic review

**DOI:** 10.1136/bmjopen-2025-112886

**Published:** 2026-06-25

**Authors:** Áine Teahan, Melissa Sharp, Ailish Farragher, Judith Strawbridge, Jean Long

**Affiliations:** 1 Evidence Centre, Health Research Board, Dublin, Ireland; 2 School of Pharmacy, Royal College of Surgeons in Ireland, Dublin, Ireland

**Keywords:** Prescriptions, Pharmacists, Systematic Review

## Abstract

**Abstract:**

**Objective:**

To examine the effectiveness and safety of pharmacist prescribing across multiple healthcare settings.

**Design:**

A systematic review of quantitative studies.

**Eligibility criteria for the selection of studies:**

Quantitative studies assessing the effectiveness and safety of pharmacist prescribing compared with non-pharmacist prescribing in any healthcare setting and for any healthcare population. A clear statement of pharmacists’ prescriptive authority was required for inclusion in this systematic review.

**Data sources:**

A systematic search was conducted using six electronic databases: Embase (Ovid), MEDLINE (EBSCO), SCiELO, Dimensions AI, Cochrane Library and Epistemonikos. Database searches were conducted from database inception to 29 January 2025. Additional grey literature searches were conducted using Google and DuckDuckGo. Both backward and forward citation chasing were conducted for all included studies.

**Data extraction and synthesis:**

Data were extracted using standardised bespoke data extraction forms. The revised Cochrane Risk of Bias 2 tool for randomised controlled trials and the Risk of Bias in Non-Randomised Studies of Interventions tool were used to assess risk of bias. A narrative synthesis approach was applied following the Synthesis Without Meta-analysis guideline. The Grading of Recommendations Assessment, Development and Evaluation approach was used to assess the level of certainty of the evidence.

**Results:**

Of the 39 included studies, 32 studies reported on effectiveness and 20 studies reported on safety across 15 health conditions. Healthcare settings included outpatient (n=14), primary care (n=10), community pharmacy (n=6), inpatient (n=5), emergency department (n=1) and long-term care (n=3). These studies were based in the USA (n=26), Canada (n=5), the UK (n=4), Australia (n=2) and Singapore (n=2). In total, there were 153 outcomes related to safety and effectiveness. For 74 outcomes, no significant difference was reported between pharmacist prescribing and non-pharmacist prescribing, while 46 outcomes were significantly improved with pharmacist prescribing. Four outcomes reported in favour of non-pharmacist prescribing. Inferential statistics were not reported for 29 outcomes, meaning we cannot comment on their statistical significance. The certainty of evidence was low or very low for all outcomes.

**Conclusions:**

The consistency of effectiveness and safety findings across studies, showing either no significant difference (indicating equivalence of care and outcomes) or significant improvement in pharmacist prescribing groups, suggests it is a potential policy option. Future research on implementation, public and patient preferences and cost-effectiveness would provide valuable insights into the potential benefits of pharmacist prescribing at a health system level.

**STRENGTHS AND LIMITATIONS OF THIS STUDY**

- The review followed best practice guidelines and included a comprehensive range of databases, healthcare populations and healthcare settings, ensuring broad coverage of available evidence.
- Only studies with explicit statements of prescriptive authority were included, ensuring clarity and consistency in what constituted pharmacist prescribing.
- Risk of bias was assessed using validated tools appropriate to study design (eg, Risk of Bias 2 (RoB 2) for parallel RCTs, RoB 2 for cluster RCTs and Risk of Bias in Non-Randomised Studies of Interventions).
- The Grading of Recommendations Assessment, Development and Evaluation assessments for outcomes across most of the studies were very low certainty.
- Several studies did not report inferential statistics, preventing full interpretation of some outcomes.

## Introduction

 A variety of health workforce strategies are implemented to improve access to and efficiency of health services globally. Internationally, there is growing demand for health services due to an ageing population and a rising burden of chronic diseases. One effective strategy to meet these increased health demands is to allow a wider range of suitably qualified healthcare professionals to prescribe medications.^1^,^5^

Prescribing by healthcare professionals, other than medical doctors and dentists, has been introduced in many jurisdictions as a strategy to address workforce pressures and improve access to medicines. Non-medical prescribers generally include pharmacists and nurse prescribers but also include other non-medical healthcare professionals who have completed approved training programmes. A Cochrane review in 2018 reported that non-medical prescribers achieve outcomes comparable to medical prescribers across various health metrics, including systolic blood pressure, glycated haemoglobin, low-density lipoprotein cholesterol levels, medication adherence, patient satisfaction and health-related quality of life.^6^ However, much of the synthesised evidence was not specific to pharmacists, and substantial heterogeneity existed across professional groups and prescribing models.

**Figure 1:**
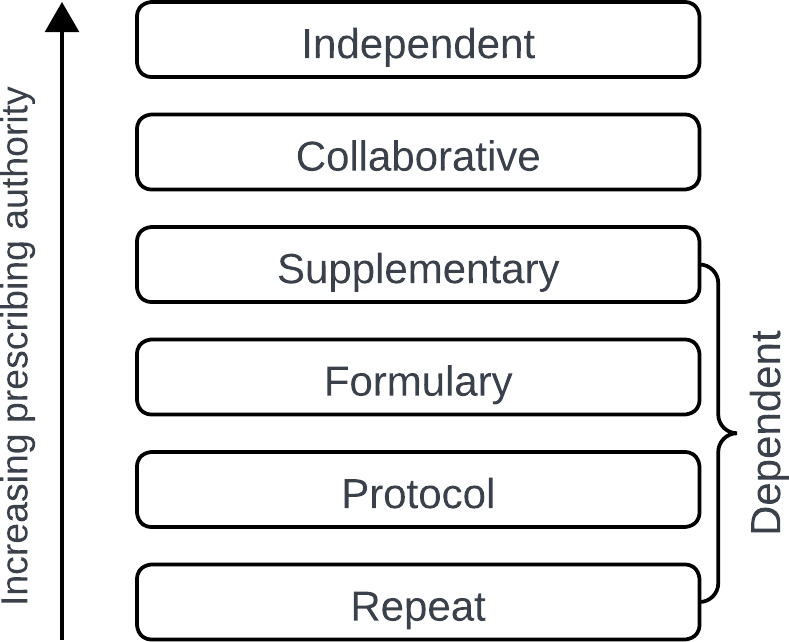
Increasing autonomy in pharmacist prescribing models.

Our systematic review specifically focuses on prescribing by pharmacists. Pharmacists’ scope of prescriptive authority varies internationally, and formal definitions vary. Some frameworks and policy documents classify pharmacist prescribing only as independent or dependent (with dependent prescribing including collaborative approaches),^7 8^ whereas others provide distinct classifications for independent, collaborative and dependent pharmacist prescribing.^9^ In this paper, we have categorised pharmacist prescribing into three models: independent prescribing, collaborative prescribing and dependent prescribing (figure 1) to better reflect variation in prescriptive authority across studies.^1^

Independent prescribing, where pharmacists can prescribe autonomously within their scope of practice, is established in the UK^10^ and parts of Canada.^11^ Collaborative prescribing, where pharmacists prescribe under a formal agreement with a doctor or healthcare team, is practised more widely, with New Zealand and parts of the USA being notable examples. Dependent prescribing, where pharmacists are limited to prescribing according to protocols or formularies, is practised in Singapore, Australia, the UK and Ireland.^12 13^ Australia is actively piloting expanded pharmacist prescribing in several states,^14^ while Ireland is considering broader reforms.^9^

From a health system perspective, introducing pharmacist prescribing may have beneficial economic implications for healthcare systems.^15 16^ Pharmacists can reduce current spending by changing treatments (such as switching from intravenous to oral therapy) and can prevent future expenses by discontinuing unnecessary or inappropriate medications, potentially avoiding adverse drug events and associated costs like general practitioner referrals or hospital admissions. Some studies have reported associations between pharmacist involvement in prescribing and reductions in medication errors and inappropriate prescribing, which may translate into downstream cost implications.^16^,^18^

We identified several systematic reviews assessing the effectiveness of non-medical prescribing aggregated across all healthcare professionals^4 6 17^ or pharmacy services more specifically.^15 16 18 19^ We identified two systematic reviews on pharmacist prescribing in hospital settings^1^ and pharmacist prescribing for minor ailments.^20^ The former reported pharmacists achieve prescribing standards comparable to physicians while reducing errors and omissions,^1^ and the latter reported significant cost savings associated with pharmacist prescribing.^20^ However, these reviews were limited to specific settings or service models and did not synthesise evidence across multiple healthcare settings.

Although the existing systematic reviews provide valuable insights, no systematic review has synthesised evidence related to the effectiveness and safety of pharmacist prescribing across multiple settings. Given international variation in prescribing models and ongoing policy reforms, a comprehensive synthesis of the evidence specific to pharmacist prescribing is warranted. This systematic review addresses two research questions: (1) Is pharmacist prescribing effective? and (2) Is pharmacist prescribing safe?

## Methods

### Protocol and registration

A protocol was registered in the International Prospective Register of Systematic Reviews (PROSPERO; CRD42024621602).^21^ This study followed the Preferred Reporting Items for Systematic Reviews and Meta-Analyses (PRISMA) 2020 reporting statement^22^ (online supplemental appendix A). A separate search was conducted on cost-effectiveness as part of this protocol; these findings are currently being prepared for publication by the authors.

### Patient and public involvement statement

Patients and members of the public were not involved in the design and conduct of this research.

### Search strategy

A systematic search was conducted using six electronic databases: Embase (Ovid), MEDLINE (EBSCO), SCiELO, Dimensions AI, Cochrane Library and Epistemonikos. Search strategies were developed by an information specialist (AF) and peer reviewed by a second information specialist following the Peer Review of Electronic Search Strategies guidelines.^23^ Detailed search strategies for each database are presented in online supplemental appendix B. The original database search was conducted between 10 and 22 July 2024. The updated database searches were conducted on 29 January 2025. No search date limit was applied to minimise the risk of missing relevant studies.

Additional grey literature searches were conducted using Google and DuckDuckGo (online supplemental appendix C). Following the Terminology, Application, and Reporting of Citation Searching statement, both backwards and forward citation chasing were conducted for all included studies.^24^ We also conducted citation searching of relevant systematic reviews in this field (online supplemental appendix C). Authors of included studies were not contacted for additional information.

### Eligibility criteria

Eligibility criteria are structured by population, intervention, comparator, outcome, setting, date and language. Both effectiveness and safety outcomes are included. Effectiveness is defined as the extent to which pharmacist prescribing produces the intended outcomes in clinical practice.^25^ ‘Safety’ is defined as the occurrence and prevention of adverse effects and the reduction of risk of unnecessary harm associated with pharmacist prescribing.^26 27^ Table 1 outlines the pre-specified eligibility criteria.

**Table 1:** Eligibility criteria

Domain	Inclusion	Exclusion
Population	Human participants receiving pharmacist prescribing/deprescribing services	Animal studies
Intervention	Prescribing/deprescribing services provided by pharmacists, including the following: Independent prescribing/deprescribing Collaborative prescribing/deprescribing Supplementary prescribing/deprescribing Formulary prescribing/deprescribing Protocol prescribing/deprescribing Any mode of delivery (in-person, online or telephone).	Prescribing/deprescribing services provided by other healthcare professionals Co-interventions delivered by non-pharmacists Pharmacist services that do not include prescribing/deprescribing services, including the following: Medicine information Compliance, adherence and/or concordance Disease screening Disease prevention Clinical intervention or identification and resolving drug-related problems Medication use reviews Disease state management Therapeutic decisions with medical practitioners.
Comparison	The following comparator groups met the inclusion criteria if provided by a non-pharmacist prescriber: Usual care No intervention Partial intervention Alternative intervention Control series.	No comparator group Pharmacist prescriber comparator group
Outcomes	Patient effectiveness outcomes, including the following: Adherence Health-related quality of life Access to care Waiting times Healthcare utilisation. Clinical outcomes, including the following: Mortality Pain Mental health Hypertension Blood sugar control Cholesterol Lung function Anticoagulation Infection Surgery. Medication-related safety outcomes, including the following: Overuse Underuse Medication appropriateness Clinically significant drug-to-drug interaction Prescribing errors Prescribing duplication Reduction in inappropriate polypharmacy. Patient adverse events, including the following: Drug-related hospital admissions Serious adverse drug reactions.	Any other outcome
Study design	Randomised controlled trials Non-randomised trials Prospective cohort studies Retrospective cohort studies in the same time period Interrupted time series studies*	Retrospective cohort studies in different time periods Cross-sectional studies Case studies Opinion pieces Qualitative studies Reviews Conference abstracts
Date	None	None

* Must include at least three observation points in the pre-intervention phase and three in the post-intervention phase.

A clear statement of pharmacists’ prescriptive authority, explicitly reporting that pharmacists prescribed, was required for study inclusion. In studies involving collaborative or dependent prescribing, a description of the role of other healthcare professionals in the prescribing process was also required.

### Study selection and data extraction

Screening was managed in EPPI-Reviewer Web software. Two reviewers (ÁT, MS) independently double-screened each title and abstract. Two reviewers independently double-screened each full text. Discrepancies were resolved through discussion with a third reviewer (AF).

### Data extraction

Data were extracted using standardised bespoke data extraction forms. Data extraction was performed by one reviewer (ÁT or MS) and checked by a second (ÁT or MS). Disagreements were resolved through discussion with a third reviewer. The data extraction forms included publication details, study details, intervention details (aim, pharmacist prescriptive authority, mode of delivery, duration and outcomes) and effect estimates (online supplemental appendix D).

### Risk of bias (RoB) assessments

The revised Cochrane Risk of Bias 2 (RoB 2) tool for RCTs^28^ and the Risk of Bias in Non-Randomised Studies of Interventions (ROBINS-I) tool^29^ were used to assess risk of bias. Studies were assessed by one author and validated by a second author (ÁT, MS). Additional quality assessment was conducted using NHLBI tools.^30^ As these findings align with the RoB 2 and ROBINS-I assessment, they are not presented in the main report but are included in online supplemental appendix E for transparency.

### Certainty of the evidence

The Grading of Recommendations Assessment, Development and Evaluation (GRADE) approach was used to assess the level of certainty of the evidence contributing to the findings on safety and effectiveness.^31^ Studies were assessed by one author and validated by a second author (ÁT, MS).

### Synthesis

#### Descriptive data

Descriptive data from the included studies were documented in the table of characteristics (online supplemental appendix F). All outcome data were extracted under two headings: effectiveness and safety. Under each heading, data were categorised by healthcare population and then by outcome.

#### Feasibility assessment for meta-analysis

For each outcome, a feasibility assessment for meta-analysis was conducted (online supplemental appendix G). We first grouped studies by healthcare population and then by outcome. Following this, for each outcome, we assessed comparability considering number of studies, study design, risk of bias, population, intervention and outcome.

#### Narrative synthesis

The feasibility assessment indicated it was not appropriate to proceed with meta-analysis. We applied a narrative approach following the Synthesis Without Meta-analysis guidelines (online supplemental appendix H).^32^ We grouped studies by relevant characteristics, including population demographics, type of intervention and comparator, outcomes measured and study design. We created detailed tables summarising the key characteristics and findings of the included studies. A p value <0.05 was considered statistically significant; this was recorded as ‘significant’ in the findings sections.

## Results

### Search results

**Figure 2:**
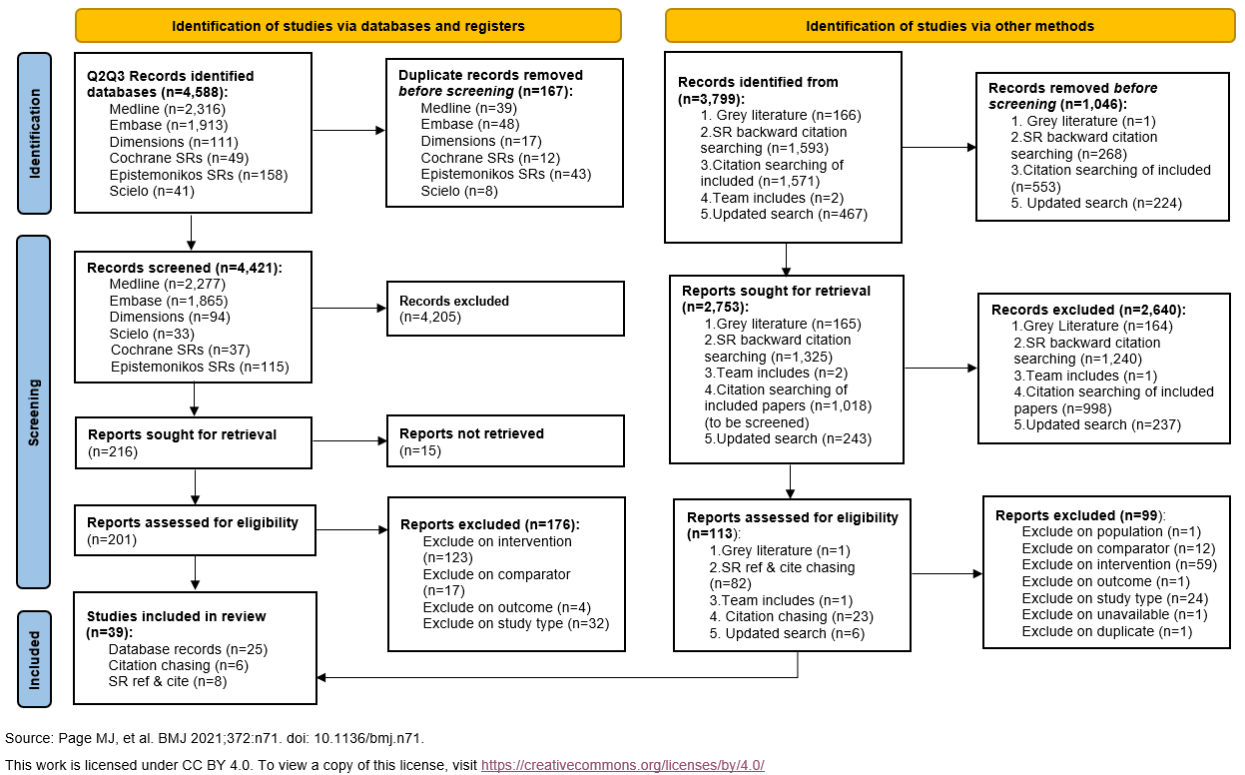
PRISMA flow diagram. PRISMA, Preferred Reporting Items for Systematic Reviews and Meta-Analyses.

Figure 2 outlines the flow of information throughout the searching and screening process.^22^ Our final search yield was 39 records. Reasons for study exclusion at the full-text screening stage are presented in online supplemental appendix I.

### Characteristics of included studies

A tabular account of the study characteristics is provided in Appendix F. Full study extraction forms are provided in Appendix D. Publication dates ranged from 1983 to 2024. All included studies comprised adult populations. Of the 39 included studies, 32 studies reported on effectiveness^33^,^64^ and 20 studies reported on safety.^4345 46 49 50 52 54^,^59 64^ These studies were based in the USA,^33^,^3537^ Canada,^45 46 56 58 66^ the UK,^51 57 63 64^ Australia^70 71^ and Singapore.^36 40^ Comparator groups included medical prescribing (n=38)^33^,^5355^ and nurse prescribing (n=1).^54^

Of the 32 studies assessing effectiveness, three were based in community pharmacies,^56 60 61^ 12 were based in outpatient clinics,^3637 39 42 43 47^,^49 52 54 55 62^ 10 in primary care,^33^,^3538 40 41 44^ three in long-term care^57^,^59^ and four in inpatient settings.^50 51 53 64^ The prescriptive authority varied: 18 studies assessed collaborative practice agreements,^33^,^4345^ seven assessed protocol prescribing,^4448^,^51 58 64^ one assessed formulary prescribing^59^ and six assessed independent prescribing.^53 56 57 60 61 63^

Of the 20 studies assessing safety, four studies were based in community pharmacies,^56 66 68 69^ seven in outpatient clinics,^43 49 52 54 55 65 67^ two in primary care,^45 46^ three in long-term care,^57^,^59^ three in inpatient settings^50 64 71^ and one in an emergency department.^70^ The prescriptive authority varied: nine studies assessed collaborative practice agreements,^43 45 50 52 54 55 65 67 70^ four assessed protocol prescribing,^49 50 58 64^ one assessed formulary prescribing,^59^ one assessed supplementary prescribing^71^ and five assessed independent prescribing.^56 57 66 68 69^

### Risk of bias assessment

**Figure 3:**
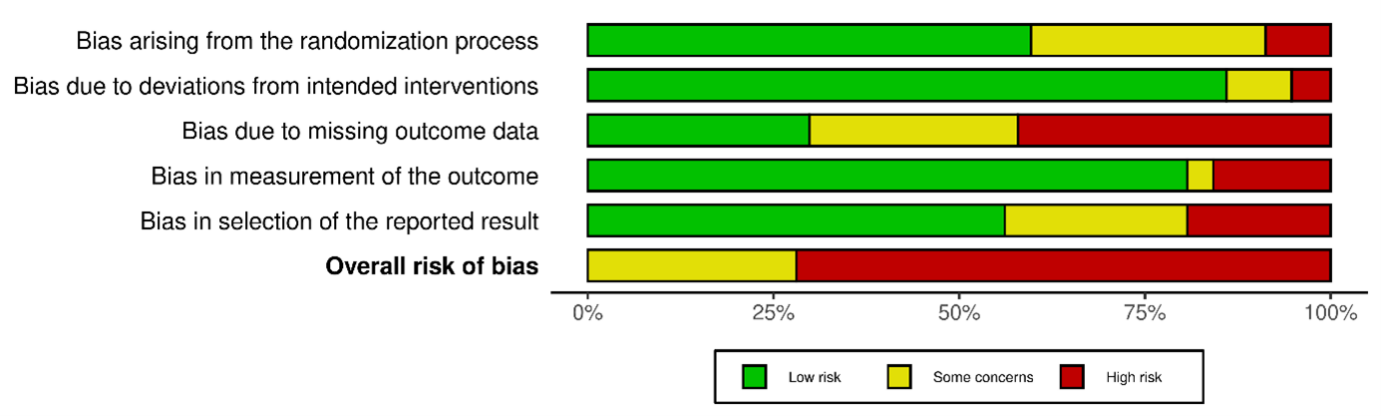
Summary of RoB 2 assessment for parallel RCTs (n=9). RCT, randomised controlled trial; RoB 2, Risk of Bias 2.

Nine parallel RCTs reported on 57 outcomes. The RoB 2 assessments were ‘high risk’ in 41 outcomes and as ‘some concerns’ in 16 outcomes (figure 3). A full account is provided in online supplemental appendix J.

**Figure 4:**
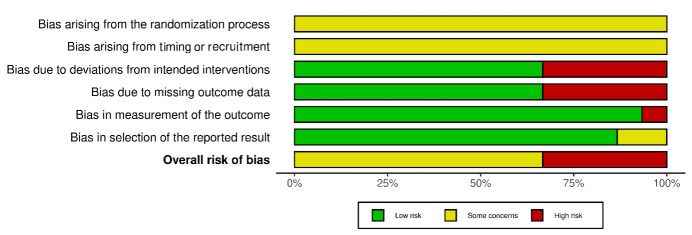
Summary of RoB 2 assessment for cluster RCTs (n=2). RCT, randomised controlled trial; RoB 2, Risk of bias 2.

Two cluster RCTs reported on 15 outcomes. The RoB 2 for cluster RCTs assessments was ‘high risk’ in five outcomes and as ‘some concerns’ in 10 outcomes (figure 4). A full account is provided in online supplemental appendix K.

**Figure 5:**
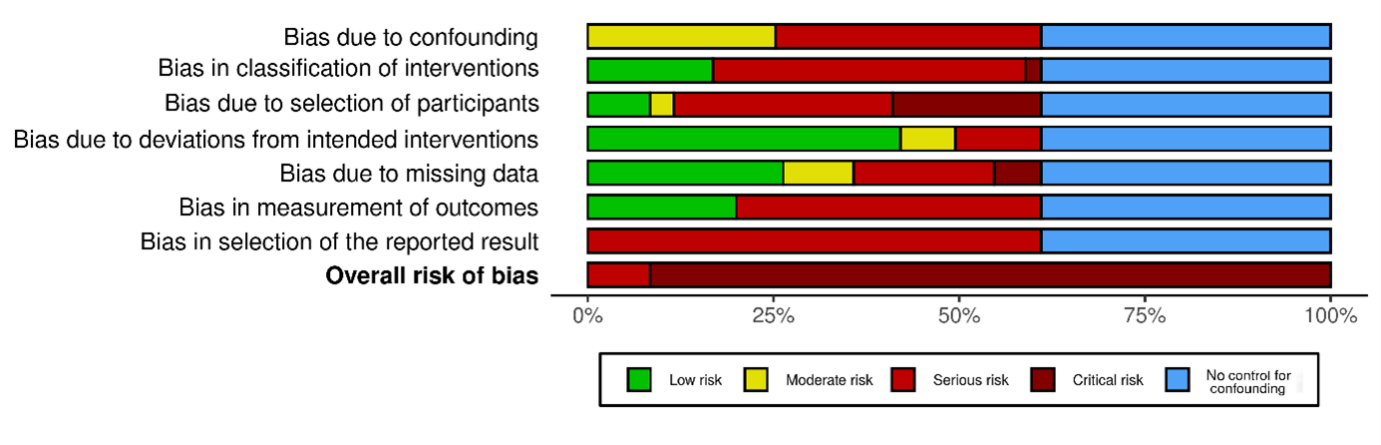
Summary of ROBINS-I assessment for non-randomised studies (n=28). ROBINS-I, Risk of Bias in Non-Randomised Studies of Interventions.

Twenty-eight non-randomised studies reported on 95 outcomes. The ROBINS-I V2 assessments were scored as ‘critical risk of bias’ in 87 outcomes and as ‘serious risk of bias’ in eight outcomes (figure 5). A full account is provided in online supplemental appendix L.

Among RCTs, the domains most frequently judged to be at some concerns or high risk of bias were randomisation procedures and missing outcome data. In non-randomised studies, not controlling for confounding variables was a common source of bias.

### Certainty of the evidence

Evidence relating to 142 outcomes was graded as very low certainty using GRADE. Evidence related to 11 outcomes was graded as low certainty. The most commonly downgraded domain was risk of bias. A full account of the GRADE assessment is provided in online supplemental appendix M.

### Findings

Studies were classified under two headings: effectiveness and safety. Under each heading, we categorised the findings first by healthcare population and then by outcome domain (online supplemental appendix N and appendix O). Overall, the majority of reported outcomes showed no significant difference or significantly improved outcomes in pharmacist prescribing compared with medical prescribing across all models of prescriptive authority (see online supplemental appendix P).

#### Effectiveness

**Figure 6:**
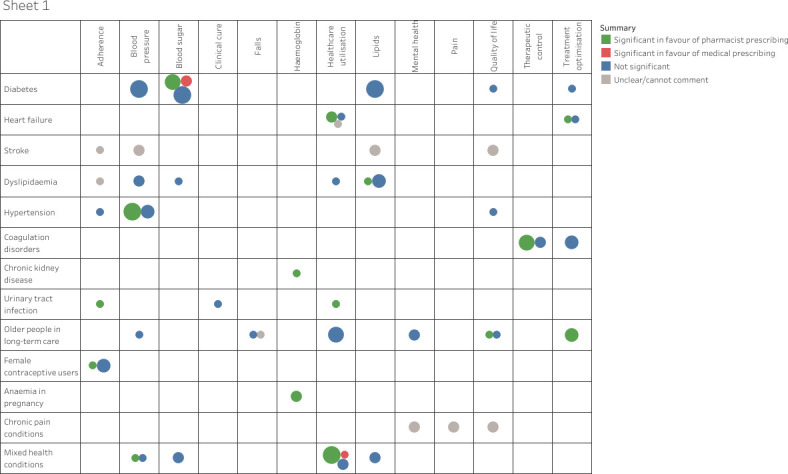
Effectiveness outcomes. *Size of circles indicates number of outcomes.

Of the 39 included studies, 32 studies reported on effectiveness.^33^,^64^ Figure 6 maps the frequency of significant outcomes in favour of pharmacist prescribing compared with medical prescribing across effectiveness outcome domains. The most frequently reported outcome domains were blood pressure, healthcare utilisation and blood sugar (online supplemental appendix Q).

#### Diabetes

Six retrospective cohort studies^34^,^39^ and two RCTs^40 41^ assessed the effectiveness of pharmacist prescribing for people with diabetes.^34^,^41^ The effectiveness outcomes assessed were blood glucose,^34^,^41^ blood pressure,^37 38^ lipids^37 38^ and health-related quality of life.^40^

For blood glucose outcomes, one study reported significantly higher mean levels and a proportion of participants achieving their glycated haemoglobin (HbA1c) goals in a medical prescribing group.^38^ No significant difference in HbA1c goal achieved,^34 35^ mean change in HbA1c^39 41^ or fasting blood glucose^37^ was reported between pharmacist prescribing and medical prescribing. Significant improvement in mean HbA1c levels was reported in pharmacist prescribing compared with medical prescribing.^36 37 40^ A significantly higher proportion of participants achieving at least a 1% decrease in HbA1c levels was reported in the pharmacist prescribing group compared with medical prescribing.^41^

No significant difference in blood pressure,^37 38^ lipids^37 38^ or health-related quality of life^40^ was reported between pharmacist prescribing and medical prescribing groups.

#### Heart failure

Two retrospective cohort studies^42 43^ and one prospective cohort study^44^ assessed the effectiveness of pharmacist prescribing for people with heart failure.^42^,^44^ The outcomes assessed related to healthcare utilisation,^42^ target angiotensin receptor-neprilysin inhibitor (ARNI) dose achieved^43^ and deprescribing.^44^

A significant improvement in 30-day all-cause readmission events but no significant difference in 30-day heart failure readmission events was reported in pharmacist prescribing compared with medical prescribing.^42^ Higher emergency department visits were reported in the pharmacist prescribing group; however, we cannot comment on the significance of this finding as no inferential statistics were reported.^42^

Significantly higher rates of achieving target ARNI doses, with significantly fewer clinic visits, were reported in pharmacist prescribing compared with medical prescribing groups.^43^ However, there was no significant difference in the number of days required to achieve target doses between groups.^43^ One study reported significantly higher rates of aspirin deprescribing in pharmacist prescribing compared with medical prescribing groups.^44^

#### Stroke

One RCT assessed the effectiveness of pharmacist prescribing for people with a recent minor ischaemic stroke or transient ischaemic attack.^45^ Higher numbers of participants were reported to meet a combined blood pressure and lipid level goal in pharmacist prescribing compared with medical prescribing.^45^ Similar findings were reported between groups for systolic blood pressure levels, low-density lipoprotein (LDL) cholesterol levels, change in high-density lipoprotein (HDL) cholesterol levels, adherence, self-rated health and health-related quality of life.^45^ Inferential statistics were not reported by the authors.

#### Dyslipidaemia

One cluster RCT assessed the effectiveness of pharmacist prescribing for people with dyslipidaemia.^46^ Participants in the pharmacist prescribing group were significantly more likely to achieve lipid targets (a dichotomous variable) but reported no significant difference between groups in continuous lipid levels.^46^ No significant difference in blood pressure or healthcare utilisation was reported between groups.^46^ More participants reported medication adherence in the pharmacist prescribing group; however, we cannot comment on the significance of this finding.^46^

#### Hypertension

One retrospective cohort study^47^ and one RCT^48^ assessed effectiveness outcomes related to hypertension. For blood pressure goal achieved, change in systolic blood pressure and change in diastolic blood pressure, the RCT reported significant improvement in pharmacist prescribing compared with medical prescribing,^48^ while the retrospective cohort study reported no significant difference between groups.^47^ Significant improvement was also reported in mean systolic blood pressure levels in pharmacist prescribing compared with medical prescribing.^48^ No significant difference was reported in mean diastolic blood pressure levels, medication adherence or health-related quality of life.^48^

#### Coagulation disorders

Three retrospective cohort studies,^49 52 54^ one non-randomised trial^51^ and one RCT^53^ assessed the effectiveness of pharmacist prescribing for people with coagulation disorders.^49^,^54^ Significant improvement was reported in the control of the International Normalised Ratio (INR) in pharmacist prescribing compared with medical prescribing.^51 52^ No significant difference in INR control achieved was reported between pharmacist prescribing and nurse prescribing.^54^

The time that INR was in a therapeutic range was higher in pharmacist prescribing compared with medical prescribing.^52^ No significant difference was reported in time to achieve therapeutic proconvertin and prothrombin levels,^53^ partial thromboplastin time^53^ or prothrombin time ratio in the therapeutic range between pharmacist prescribing and medical prescribing groups.^49^ A similar time to achieve therapeutic INR was reported in pharmacist and medical prescribing groups, but no inferential statistics were reported.^50^

#### Chronic kidney disease

One retrospective cohort study reported significant improvement in the haemoglobin goal achieved in pharmacist prescribing compared with medical prescribing for people with chronic kidney disease.^55^

#### Urinary tract infection

One non-randomised trial reported significant improvement in time to access care and medication adherence in pharmacist prescribing compared with medical prescribing for urinary tract infection in women.^56^ No significant difference in clinical cure at 2 weeks was reported between groups.

#### Older people in long-term care

Two RCTs^57 58^ and one non-randomised trial^59^ assessed the effectiveness of pharmacist prescribing for older people in long-term care. One RCT reported no significant difference in falls between pharmacist and medical prescribing.^57^ The other RCT reported similar numbers of falls between groups; no inferential statistics were reported.^58^

Significant improvement in pharmacist prescribing compared with medical prescribing was reported for drug burden.^57 59^ Two RCTs assessed health-related quality of life, reporting significant improvement in pharmacist prescribing compared with medical prescribing^57^ or no significant difference between groups.^58^ No significant difference in depression,^58^ anxiety,^58^ systolic blood pressure levels,^58^ hospitalisations^57^,^59^ and emergency department admissions^58^ was reported between pharmacist prescribing and medical prescribing groups.

#### Female contraceptive users

One prospective cohort study^60^ and one retrospective cohort study^61^ assessed the effectiveness of pharmacist prescribing of contraception for women focusing on medication adherence (no break in coverage) or medication continuation at 12 months compared with medical prescribing. In one study, where 88.9% of their sample intended to avoid pregnancy for the next 12 months, no significant difference in adherence or continuation was reported between groups.^60^ The other study reported significantly higher adherence in pharmacist prescribing compared with medical prescribing; no data on pregnancy intention were collected.^61^

#### Anaemia in pregnancy

One retrospective cohort study reported significant improvement in haemoglobin goal achieved and mean haemoglobin levels in pharmacist prescribing compared with medical prescribing.^62^

#### Chronic pain conditions

One RCT reported improvement in chronic pain intensity (p=0.02), chronic pain disability (p=0.55), health-related quality of life (p=0.04), depression (p=0.32) and anxiety (p=0.14) in a one-way analysis of variance between three trial arms (pharmacist prescribing vs pharmacist medication review vs medical prescribing).^63^ The one-way analysis of variance does not identify which specific arm comparisons account for the observed difference. Subsequently, it is not possible to comment on the significance of the effect between either pharmacist prescribing and medical prescribing or pharmacist prescribing and pharmacist medication review.

#### Mixed health conditions

One retrospective cohort study^33^ and one RCT^64^ assessed the effectiveness of pharmacist prescribing for people with mixed health conditions. Significantly more ambulatory care visits were reported in pharmacist prescribing compared with physician prescribing, but no significant difference was reported between pharmacist prescribing and primary care provider prescribing.^33^

Significant improvement in length of hospital stay and hospital readmissions was reported in pharmacist prescribing compared with medical prescribing^64^ and in pharmacist prescribing compared with physician prescribing or primary care provider prescribing.^33^

There was no significant difference in emergency department visits in pharmacist prescribing compared with physician prescribing.^33^ There were significantly fewer emergency department visits with pharmacist prescribing compared with the primary care provider prescribing.^33^

There was no significant difference in blood pressure goal achieved in the pharmacist prescribing group compared with the physician prescribing group.^33^ A significantly higher percentage of patients achieved their blood pressure goals in the pharmacist prescribing group compared with the primary care provider prescribing group.^33^ No significant difference in HbA1c goals achieved was reported between the pharmacist prescribing group compared with the physician prescribing or primary care provider prescribing groups.^33^

#### Safety

**Figure 7:**
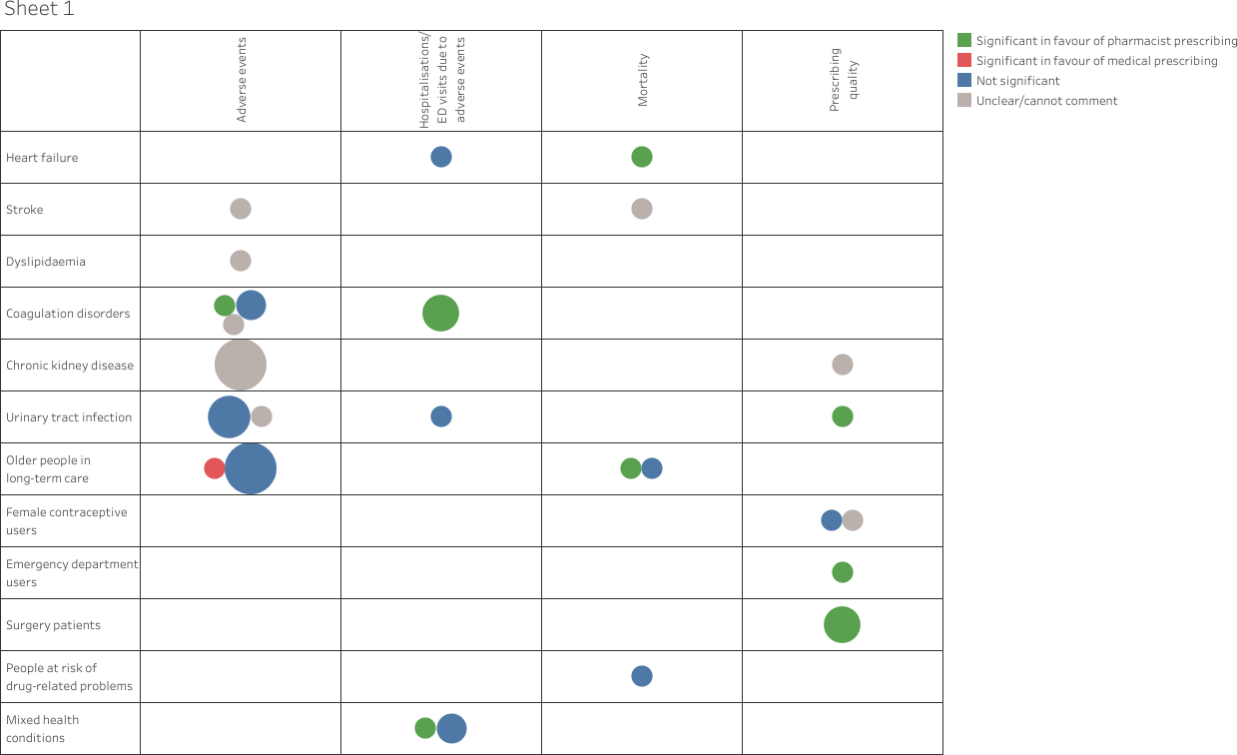
Safety outcomes. *Size of circles indicate number of outcomes.

Of the 39 included studies, 20 studies reported on safety.^4345 46 49 50 52 54^,^59 64^ Figure 7 maps the frequency of significant outcomes in favour of pharmacist prescribing compared with medical prescribing across safety outcome domains. The most frequently reported outcome domains were adverse events, prescribing quality and hospitalisations/emergency department visits due to adverse events (Appendix Q).

#### Heart failure

One retrospective cohort study reported no significant difference in heart failure hospitalisations and significantly fewer all-cause deaths in pharmacist prescribing compared with medical prescribing in heart failure patients.^43^

#### Stroke

One RCT reported vascular adverse events and mortality rates appeared similar in pharmacist prescribing compared with medical prescribing in people with a recent minor ischaemic stroke or transient ischaemic attack;^45^ however, we cannot comment on the significance of these findings.

#### Dyslipidaemia

One RCT reported similar adverse event rates in pharmacist prescribing compared with medical prescribing groups for people with dyslipidaemia; however, we cannot comment on the significance of these findings.^46^

#### Coagulation disorders

Four retrospective cohort studies assessed the safety of pharmacist prescribing for people with coagulation disorders.^49 50 52 54^

There was no significant difference in bleeding adverse events^49^ or thromboembolic adverse events^49^ in pharmacist prescribing compared with medical prescribing, but significantly lower anticoagulation-related adverse events^52^ in pharmacist prescribing compared with medical prescribing. One study reported fewer bleeding adverse events/adverse drug events in pharmacist prescribing compared with medical prescribing; however, we cannot comment on the significance of this finding.^50^

Significantly lower hospitalisations and emergency department visits were reported in pharmacist prescribing compared with medical prescribing.^52^ A significantly lower likelihood of warfarin-related hospitalisations/emergency department visits was reported in pharmacist prescribing compared with nurse prescribing groups.^54^

#### Chronic kidney disease

Two retrospective cohort studies assessed safety outcomes in people with chronic kidney disease for pharmacist prescribing compared with medical prescribing.^55 65^

A higher rate of thromboembolic events per 180 days was reported in pharmacist prescribing compared with medical prescribing,^55^ but a lower rate was reported in pharmacist prescribing compared with usual care; we cannot comment on the significance of these findings.^55^ A higher rate of heart failure adverse events and uncontrolled adverse events per 180 days was reported in pharmacist prescribing compared with both medical prescribing and usual care; we cannot comment on the significance of these findings.^55^

Although a lower number of prescribing errors in pharmacist prescribing compared with medical prescribing, we cannot comment on the significance of this finding.^65^

#### Urinary tract infection

Two non-randomised trials assessed safety outcomes of pharmacist prescribing for people with urinary tract infections.^56 66^ No significant difference in gastrointestinal adverse events, vaginal candidiasis adverse events, headache adverse events, other adverse events or physician/emergency department visits was reported between the pharmacist prescribing and medical prescribing groups.^56^ Fewer total adverse events were reported in pharmacist prescribing; however, we cannot comment on the significance of this finding.^56^ A significantly higher concordance with antimicrobial guidelines was reported in pharmacist prescribing compared with medical prescribing.^66^

#### Older people in long-term care

Two RCTs^57 58^ and one non-randomised trial^59^ assessed safety outcomes of pharmacist prescribing for older people in long-term care. There were significantly lower mortality events^59^ or no significant difference^57^ reported in pharmacist prescribing compared with medical prescribing. There were significantly higher hypotension adverse events reported in pharmacist prescribing compared with medical prescribing.^58^ No significant difference between groups was reported in syncope adverse events, hypokalaemia adverse events, hyperkalaemia adverse events, hyponatraemia adverse events, orthostatic presyncope adverse events or estimated glomerular filtration rate.^58^

#### Female contraceptive users

A prospective cohort study^68^ and a retrospective cohort study^69^ reported on medical contraindications among women prescribed contraception in pharmacist prescribing groups compared with medical prescribing groups.^68 69^ One study reported no significant difference between groups.^68^ The other study reported a lower rate of medical contraindication in pharmacist prescribing; we cannot comment on the significance of this finding.^69^

#### Emergency department patients

One RCT reported significantly fewer prescribing errors for emergency department patients in pharmacist prescribing compared with the medical prescribing group.^70^

#### Surgery patients

One RCT reported significantly lower medications charted at incorrect frequency, medications charted at incorrect dose and doses missed in pharmacist prescribing compared with medical prescribing groups.^71^

#### People at risk of drug-related problems

One RCT reported no significant difference in mortality for patients at risk of drug-related problems between the pharmacist prescribing and medical prescribing groups.^64^

#### Mixed health conditions

One retrospective cohort study for people with mixed health conditions reported no significant difference in the likelihood of hospitalisations and emergency department visits due to acute kidney events and gastrointestinal bleeding adverse events between pharmacist prescribing and medical prescribing.^67^ A significantly lower likelihood of hospitalisations and emergency department visits due to pain was reported in pharmacist prescribing.^67^

## Discussion

Internationally, this is the most comprehensive evidence review on pharmacist prescribing that has been conducted to date. This review covers a diverse range of healthcare settings, populations and prescriptive authorities. Our findings broadly align with previous systematic reviews of pharmacist prescribing in hospital settings,^1^ in minor ailment management schemes^20^ and those focused on non-medical prescribing aggregated across all healthcare professionals.^4 6 17^

Out of the 153 outcomes related to safety and effectiveness, 46 outcomes were significantly improved with pharmacist prescribing. For 74 outcomes, no significant difference was reported indicating equivalence of care and outcomes between pharmacist prescribing and other prescribing groups. Inferential statistics were not reported for 29 outcomes, meaning we cannot comment on the statistical significance. Only four outcomes reported better outcomes in medical prescribing compared with pharmacist prescribing including decreased outpatient clinic visits but more hospitalisations, improved HbA1C and significantly fewer hypotension adverse events.

Overall, most reported outcomes showed either no significant difference or improved outcomes for pharmacist prescribing compared with medical prescribing across models of prescriptive authority. However, not all models have been evaluated across all health populations or healthcare settings; for example, no studies of independent prescribing were conducted in outpatient settings, and no CPA studies were based in community settings. Subsequently, it is not possible to determine a single ‘best’ model, and different models of prescriptive authority may be more appropriate depending on the healthcare setting and population. In addition, variations in country-specific legislative frameworks, protocols and national guidelines were not examined in detail across the included studies and may influence the implementation and effectiveness of different models.

A limitation of this review is that we only included studies with a clear statement of prescriptive authority. This approach was necessary to ensure consistency in the studies selected. It is possible that studies excluded on this basis may have met our inclusion criteria in other respects and could still offer valuable insights if prescriptive authority had been explicitly stated. Additionally, in countries with established independent prescribing models (eg, Canada), large research projects have investigated enhanced models. The comparator groups of these studies included usual care pharmacist prescribers and therefore did not meet our inclusion criteria. These studies investigated how increased engagement of independent pharmacist prescribers could lower cardiovascular risk,^73^ optimise hypertension treatments,^74^ improve lipid levels^75^ and reduce the likelihood of drug overdose.^76 77^ A methodological limitation of this study was the lack of independent data extraction. Additionally, three of the included studies are dated pre-2000, limiting their relevance to modern health systems.^49 53 59^

Our included studies only investigate pharmacist prescribing in adults; no studies investigating paediatric populations met our inclusion criteria. We were unable to identify an existing systematic review on pharmacist prescribing in paediatric populations.^78^ Additionally, limited research exists on mental health, respiratory conditions and infectious diseases. Conducting primary research in these populations would contribute to a broader understanding of pharmacist prescribing for different healthcare needs.

Our evidence base was characterised by several methodological limitations, including a predominance of observational designs and high risk of bias, particularly due to confounding in non-randomised studies. Consequently, the findings demonstrate a consistent positive trend but do not provide strong causal evidence. As outlined in figures 3 and 4, and figure 5, sources of bias in included studies differed according to study design. Among RCTs, the domains most frequently judged to be at some concerns or high risk of bias were randomisation procedures and missing outcome data. In non-randomised studies, not controlling for confounding variables was a common source of bias. Future research could minimise risk of bias by addressing these methodological limitations and by providing transparent and comprehensive reporting in published articles.

Although studies generally specified the type of prescribing authority, heterogeneity in study design, clinical context, outcomes measured and implementation models limited the feasibility of stratified synthesis by prescribing type. As a result, the implications for policy and practice should be considered in light of this variability. Future research using robust comparative designs within clearly defined prescribing models would strengthen the evidence base and support more precise policy conclusions.

Although the certainty of the evidence is very low, the consistency of findings showing either no significant difference or significant improvement in pharmacist prescribing across prescriptive models suggests it is a viable policy option. This is particularly the case if pharmacist prescribing is shown to be cost-effective. A recent study supports this interpretation recommending that when policy options demonstrate potential equivalence, decision-makers should favour the less costly alternative, even when the GRADE assessment indicated very low certainty.^79^ The authors are currently conducting a separate systematic review on the cost-effectiveness of pharmacist prescribing.

To conclude, this comprehensive systematic review reported pharmacist prescribing achieved outcomes that were equivalent to, or significantly improved, compared with other prescribers across a range of healthcare populations and healthcare settings. The direction of evidence consistently supported pharmacist prescribing, with few outcomes favouring other prescribers.

## Supplementary material

10.1136/bmjopen-2025-112886 online supplemental file 1

## Data Availability

Data are available upon reasonable request.
