# Supplementary material for: Effectiveness and safety of pharmacist prescribing: a systematic review
Source: BMJ Open. 2026 Jun 25;16(6):e112886. doi: 10.1136/bmjopen-2025-112886 (PMC13311757; doi:10.1136/bmjopen-2025-112886)
Supplement: online supplemental file 1 [file bmjopen-16-6-s001.pdf]

# Table of Contents


Appendix A Preferred Reporting Items for Systematic reviews and Meta-Analyses (PRISMA) ....................2

Appendix B Literature search details ....................5

Appendix C Supplementary searches ....................13

Appendix D Data extraction form of 39 included studies ....................18

Appendix E National Heart, Lung, and Blood Institute tool quality assessments ....................463

Appendix F Table of characteristics ....................470

Appendix G Feasibility assessment for meta-analysis ....................487

Appendix H Synthesis Without Meta-analysis (SWiM) ....................503

Appendix I Excluded studies ....................504

Appendix J Cochrane Risk of Bias 2 tool (for parallel randomised controlled trials) ....................529

Appendix K Cochrane Risk of Bias 2 tool (for cluster randomised controlled trials) ....................541

Appendix L Risk Of Bias In Non-Randomised Studies – of Interventions assessment ....................544

Appendix M Grading of Recommendations Assessment, Development, and Evaluation ....................566

Appendix N Table of effectiveness findings ....................592

Appendix O Table of safety findings ....................606

Appendix P Reported outcome significance across models of prescriptive authority ....................614

Appendix Q Number of outcomes reported by outcome domain ....................615

# Appendix A Preferred Reporting Items for Systematic reviews and Meta-Analyses (PRISMA)

| Section and topic | Item # | Checklist item | Location where item is reported |
|---|---|---|---|
| **TITLE** | | | |
| Title | 1 | Identify the report as a systematic review. | p1 |
| **ABSTRACT** | | | |
| Abstract | 2 | See the PRISMA 2020 for Abstracts checklist. | p1 |
| **INTRODUCTION** | | | |
| Rationale | 3 | Describe the rationale for the review in the context of existing knowledge. | p3-4 |
| Objectives | 4 | Provide an explicit statement of the objective(s) or question(s) the review addresses. | p4 |
| **METHODS** | | | |
| Eligibility criteria | 5 | Specify the inclusion and exclusion criteria for the review and how studies were grouped for the syntheses. | Table 5 |
| Information sources | 6 | Specify all databases, registers, websites, organisations, reference lists and other sources searched or consulted to identify studies. Specify the date when each source was last searched or consulted. | Appendix B, Appendix C |
| Search strategy | 7 | Present the full search strategies for all databases, registers and websites, including any filters and limits used. | Appendix B, Appendix C |
| Selection process | 8 | Specify the methods used to decide whether a study met the inclusion criteria of the review, including how many reviewers screened each record and each report retrieved, whether they worked independently, and if applicable, details of automation tools used in the process. | p5 |
| Data collection process | 9 | Specify the methods used to collect data from reports, including how many reviewers collected data from each report, whether they worked independently, any processes for obtaining or confirming data from study investigators, and if applicable, details of automation tools used in the process. | p5-6 |
| Data items | 10a | List and define all outcomes for which data were sought. Specify whether all results that were compatible with each outcome domain in each study were sought (e.g. for all measures, time points, analyses), and if not, the methods used to decide which results to collect. | p5-6 |
| | 10b | List and define all other variables for which data were sought (e.g. participant and intervention characteristics, funding sources). Describe any assumptions made about any missing or unclear information. | p5-6 |
| Study risk of bias assessment | 11 | Specify the methods used to assess risk of bias in the included studies, including details of the tool(s) used, how many reviewers assessed each study | p6 |

| Section and topic | Item # | Checklist item | Location where item is reported |
|---|---|---|---|
| | | and whether they worked independently, and if applicable, details of automation tools used in the process. | |
| Effect measures | 12 | Specify for each outcome the effect measure(s) (e.g. risk ratio, mean difference) used in the synthesis or presentation of results. | p6 |
| Synthesis methods | 13a | Describe the processes used to decide which studies were eligible for each synthesis (e.g. tabulating the study intervention characteristics and comparing against the planned groups for each synthesis (item #5)). | p6 |
| | 13b | Describe any methods required to prepare the data for presentation or synthesis, such as handling of missing summary statistics, or data conversions. | p6 |
| | 13c | Describe any methods used to tabulate or visually display results of individual studies and syntheses. | p6 |
| | 13d | Describe any methods used to synthesize results and provide a rationale for the choice(s). If meta-analysis was performed, describe the model(s), method(s) to identify the presence and extent of statistical heterogeneity, and software package(s) used. | p6 |
| | 13e | Describe any methods used to explore possible causes of heterogeneity among study results (e.g. subgroup analysis, meta-regression). | Not applicable |
| | 13f | Describe any sensitivity analyses conducted to assess robustness of the synthesized results. | Not applicable |
| Reporting bias assessment | 14 | Describe any methods used to assess risk of bias due to missing results in a synthesis (arising from reporting biases). | p6 |
| Certainty assessment | 15 | Describe any methods used to assess certainty (or confidence) in the body of evidence for an outcome. | p5 |
| **RESULTS** | | | |
| Study selection | 16a | Describe the results of the search and selection process, from the number of records identified in the search to the number of studies included in the review, ideally using a flow diagram. | p6 |
| | 16b | Cite studies that might appear to meet the inclusion criteria, but which were excluded, and explain why they were excluded. | Appendix I |
| Study characteristics | 17 | Cite each included study and present its characteristics. | Appendix F |
| Risk of bias in studies | 18 | Present assessments of risk of bias for each included study. | Appendix J, Appendix K, Appendix L |
| Results of individual studies | 19 | For all outcomes, present, for each study: (a) summary statistics for each group (where appropriate) and (b) an effect estimate and its precision (e.g. confidence/credible interval), ideally using structured tables or plots. | Appendix F |

| Section and topic | Item # | Checklist item | Location where item is reported |
|---|---|---|---|
| Results of syntheses | 20a | For each synthesis, briefly summarise the characteristics and risk of bias among contributing studies. | p7 |
| | 20b | Present results of all statistical syntheses conducted. If meta-analysis was done, present for each the summary estimate and its precision (e.g. confidence/credible interval) and measures of statistical heterogeneity. If comparing groups, describe the direction of the effect. | Not applicable |
| | 20c | Present results of all investigations of possible causes of heterogeneity among study results. | Not applicable |
| | 20d | Present results of all sensitivity analyses conducted to assess the robustness of the synthesized results. | Not applicable |
| Reporting biases | 21 | Present assessments of risk of bias due to missing results (arising from reporting biases) for each synthesis assessed. | Appendix J, Appendix K, Appendix L |
| Certainty of evidence | 22 | Present assessments of certainty (or confidence) in the body of evidence for each outcome assessed. | Appendix M |
| **DISCUSSION** | | | |
| Discussion | 23a | Provide a general interpretation of the results in the context of other evidence. | p14-15 |
| | 23b | Discuss any limitations of the evidence included in the review. | p14-15 |
| | 23c | Discuss any limitations of the review processes used. | p14-15 |
| | 23d | Discuss implications of the results for practice, policy, and future research. | p14-15 |
| **OTHER INFORMATION** | | | |
| Registration and protocol | 24a | Provide registration information for the review, including register name and registration number, or state that the review was not registered. | p5 |
| | 24b | Indicate where the review protocol can be accessed, or state that a protocol was not prepared. | p5 |
| | 24c | Describe and explain any amendments to information provided at registration or in the protocol. | p5 |
| Support | 25 | Describe sources of financial or non-financial support for the review, and the role of the funders or sponsors in the review. | p16 |
| Competing interests | 26 | Declare any competing interests of review authors. | p16 |
| Availability of data, code and other materials | 27 | Report which of the following are publicly available and where they can be found: template data collection forms; data extracted from included studies; data used for all analyses; analytic code; any other materials used in the review. | p16 |

# Appendix B Literature search details

EBSCO MEDLINE search strategy

Research question: Q1 and Q2 (effectiveness and safety)

Search date: 11 July 2024

| # | Query | Limiters/Expanders | Last Run Via | Results |
|---|---|---|---|---|
| S34 | S16 AND S32 | Limiters - Peer Reviewed<br>Expanders - Apply equivalent subjects<br>Search modes - Proximity | Interface - EBSCOhost Research Databases<br>Search Screen - Advanced Search<br>Database - MEDLINE | 2,316 |
| S33 | S16 AND S32 | Expanders - Apply equivalent subjects<br>Search modes - Proximity | Interface - EBSCOhost Research Databases<br>Search Screen - Advanced Search<br>Database - MEDLINE | 2,336 |
| S32 | S17 OR S18 OR S19 OR S20 OR S21 OR S22 OR S23 OR S24 OR S25 OR S26 OR S27 OR S28 OR S29 OR S30 OR S31 | Expanders - Apply equivalent subjects<br>Search modes - Proximity | Interface - EBSCOhost Research Databases<br>Search Screen - Advanced Search<br>Database - MEDLINE | 2,911,761 |
| S31 | PT clinical trial | Expanders - Apply equivalent subjects<br>Search modes - Proximity | Interface - EBSCOhost Research Databases<br>Search Screen - Advanced Search<br>Database - MEDLINE | 540,156 |
| S30 | TI ("Prospective Stud*") OR AB ("Prospective Stud*") | Expanders - Apply equivalent subjects<br>Search modes - Proximity | Interface - EBSCOhost Research Databases<br>Search Screen - Advanced Search<br>Database - MEDLINE | 217,819 |
| S29 | TI ("Retrospective Stud*") OR AB ("Retrospective Stud*") | Expanders - Apply equivalent subjects<br>Search modes - Proximity | Interface - EBSCOhost Research Databases<br>Search Screen - Advanced Search<br>Database - MEDLINE | 244,874 |
| S28 | TI ( ("non-randomi#ed controlled" or "nonrandomi#ed controlled" or (nonrandom* N2 control*) ) OR AB ( ("non-randomi#ed controlled" or "nonrandomi#ed controlled" or (nonrandom* N2 control*) ) | Expanders - Apply equivalent subjects<br>Search modes - Proximity | Interface - EBSCOhost Research Databases<br>Search Screen - Advanced Search<br>Database - MEDLINE | 5,352 |
| S27 | TI ( ("randomi#ed controlled" or (random* N2 control*) ) OR AB ( ("randomi#ed controlled" or random* N2 control*) ) | Expanders - Apply equivalent subjects<br>Search modes - Proximity | Interface - EBSCOhost Research Databases<br>Search Screen - Advanced Search<br>Database - MEDLINE | 411,573 |
| S26 | (MH "Prospective Studies") | Expanders - Apply equivalent subjects<br>Search modes - Proximity | Interface - EBSCOhost Research Databases<br>Search Screen - Advanced Search<br>Database - MEDLINE | 691,899 |
| S25 | (MH "Retrospective Studies") | Expanders - Apply equivalent subjects<br>Search modes - Proximity | Interface - EBSCOhost Research Databases<br>Search Screen - | 1,215,173 |

| | | | | |
|---|---|---|---|---|
| | | | Advanced Search Database - MEDLINE | |
| S24 | (MH "Non-Randomized Controlled Trials as Topic") | Expanders - Apply equivalent subjects Search modes - Proximity | Interface - EBSCOhost Research Databases Search Screen - Advanced Search Database - MEDLINE | 1,104 |
| S23 | MH "Randomized Controlled Trials as Topic+") | Expanders - Apply equivalent subjects Search modes - Proximity | Interface - EBSCOhost Research Databases Search Screen - Advanced Search Database - MEDLINE | 175,610 |
| S22 | (MH "Controlled Clinical Trials as Topic+") | Expanders - Apply equivalent subjects Search modes - Proximity | Interface - EBSCOhost Research Databases Search Screen - Advanced Search Database - MEDLINE | 181,415 |
| S21 | TI (time* series AND (((pre OR before OR prior) N/5 (post OR after OR follow*)) OR quasi-experiment* OR quasiexperiment* OR natural experiment* OR ARIMA OR autoregress* OR auto-regress* OR segmented OR segments OR piecewise OR piece-wise OR interrupt* OR implement* OR guideline*) OR AB (time* series AND (((pre OR before OR prior) N/5 (post OR after OR follow*)) OR quasi-experiment* OR quasiexperiment* OR natural experiment* OR ARIMA OR autoregress* OR auto-regress* OR segmented OR segments OR piecewise OR piece-wise OR interrupt* OR implement* OR guideline*) | Expanders - Apply equivalent subjects Search modes - Proximity | Interface - EBSCOhost Research Databases Search Screen - Advanced Search Database - MEDLINE | 14,463 |
| S20 | TX ((piecewise OR piece-wise)) | Expanders - Apply equivalent subjects Search modes - Proximity | Interface - EBSCOhost Research Databases Search Screen - Advanced Search Database - MEDLINE | 7,259 |
| S19 | TX (integrat* moving average OR slope change) | Expanders - Apply equivalent subjects Search modes - Proximity | Interface - EBSCOhost Research Databases Search Screen - Advanced Search Database - MEDLINE | 2,733 |
| S18 | TI (segment and regression) OR AB (segment and regression) | Expanders - Apply equivalent subjects Search modes - Proximity | Interface - EBSCOhost Research Databases Search Screen - Advanced Search Database - MEDLINE | 11,737 |
| S17 | TI "(interrupt* time* series)" OR AB "(interrupt* time* series)" | Expanders - Apply equivalent subjects Search modes - Proximity | Interface - EBSCOhost Research Databases Search Screen - | 6,546 |

| | | | | |
|---|---|---|---|---|
| | | | Advanced Search<br>Database - MEDLINE | |
| S16 | S1 OR S2 OR S3 OR S4 OR S5 OR S6 OR S7 OR S8 OR S9 OR S10 OR S11 OR S12 OR S13 OR S14 OR S15 | Expanders - Apply equivalent subjects<br>Search modes - Proximity | Interface - EBSCOhost Research Databases<br>Search Screen - Advanced Search<br>Database - MEDLINE | 10,952 |
| S15 | TX (prescrib* N3 pharmacist*) | Expanders - Apply equivalent subjects<br>Search modes - Proximity | Interface - EBSCOhost Research Databases<br>Search Screen - Advanced Search<br>Database - MEDLINE | 1,960 |
| S14 | TI ( (collaborative n5 pharmacist) or (pharmacist-physician) ) OR AB ( (collaborative n5 pharmacist) or (pharmacist-physician) ) | Expanders - Apply equivalent subjects<br>Search modes - Proximity | Interface - EBSCOhost Research Databases<br>Search Screen - Advanced Search<br>Database - MEDLINE | 1,001 |
| S13 | SU "pharmacist-physician" | Expanders - Apply equivalent subjects<br>Search modes - Proximity | Interface - EBSCOhost Research Databases<br>Search Screen - Advanced Search<br>Database - MEDLINE | 30 |
| S12 | (MH “Evidence-Based Pharmacy Practice”) OR (MH “Scope of Practice”) | Expanders - Apply equivalent subjects<br>Search modes - Proximity | Interface - EBSCOhost Research Databases<br>Search Screen - Advanced Search<br>Database - MEDLINE | 265 |
| S11 | ((prescrib* N3 pharmacist*) AND ((MH “Scope of Practice”) or (MH “Evidence-Based Pharmacy Practice”)) or (SU "pharmacist-physician" or TI (collaborative n5 pharmacist) or TI (pharmacist-physician)) | Expanders - Apply equivalent subjects<br>Search modes - Proximity | Interface - EBSCOhost Research Databases<br>Search Screen - Advanced Search<br>Database - MEDLINE | 301 |
| S10 | TI ((("non-medical prescribing" or "non-medical prescribing" or prescribing or pharmacist-led) N7 pharmacist*))) OR AB ((("non-medical prescribing" or "non-medical prescribing" or prescribing or pharmacist-led) N7 pharmacist*))) | Expanders - Apply equivalent subjects<br>Search modes - Proximity | Interface - EBSCOhost Research Databases<br>Search Screen - Advanced Search<br>Database - MEDLINE | 3,334 |
| S9 | TX (pharmacist N2 prescri*) AND SU (Medication Therapy Management*) | Expanders - Apply equivalent subjects<br>Search modes - Proximity | Interface - EBSCOhost Research Databases<br>Search Screen - Advanced Search<br>Database - MEDLINE | 55 |
| S8 | TX ("pharmacist-prescriber*") OR "pharmacist prescriber*" or “independent prescrib*” or "prescribing clinical pharmacist*" or "pharmacist-led" or (prescribing N3 NMP) ) | Expanders - Apply equivalent subjects<br>Search modes - Proximity | Interface - EBSCOhost Research Databases<br>Search Screen - Advanced Search<br>Database - MEDLINE | 2,288 |
| S7 | TI (("pharmacist-prescriber*" OR "pharmacist prescriber*" or “independent prescrib*” or | Expanders - Apply equivalent subjects<br>Search modes - Proximity | Interface - EBSCOhost Research Databases<br>Search Screen - | 2,249 |

| | | | | |
|---|---|---|---|---|
| | ("prescribing clinical pharmacist*") or "pharmacist-led" or (prescribing N3 NMP) or "pharmacist-independent prescriber" or "pharmacist independent prescriber" or (pharmacist N2 PIP))) OR AB (("pharmacist-prescriber*" OR "pharmacist prescriber*" or “independent prescrib*” or ("prescribing clinical pharmacist*") or "pharmacist-led" or (prescribing N3 NMP) or "pharmacist-independent prescriber" or "pharmacist independent prescriber" or (pharmacist N2 PIP)) | | Advanced Search Database - MEDLINE | |
| S6 | TI ( (pharmacist* and prescribing) N5 (authority or "additional authori*" or “additional prescribing authori#ation” or right# or train* OR "prescribing training")) OR AB ( (pharmacist* and prescribing) N5 (authority or "additional authori*" or “additional prescribing authori#ation” or right# or train* OR "prescribing training")) | Expanders - Apply equivalent subjects Search modes - Proximity | Interface - EBSCOhost Research Databases Search Screen - Advanced Search Database - MEDLINE | 494 |
| S5 | TI (((pharmacist* and prescrib*) and (formular* or (pharmacist N2 protocol) or "supplementary prescribing" or "prescribing practice" or "non-medical prescri*" or "non medical prescri*"))) OR AB (((pharmacist* and prescrib*) and (formular* or (pharmacist N2 protocol) or "supplementary prescribing" or "prescribing practice" or "non-medical prescri*" or "non medical prescri*"))) | Expanders - Apply equivalent subjects Search modes - Proximity | Interface - EBSCOhost Research Databases Search Screen - Advanced Search Database - MEDLINE | 411 |
| S4 | TI (((pharmacist* N5 deprescrib*) OR deprescrip* )) OR AB (((pharmacist* N5 deprescrib*) OR deprescrip* )) | Expanders - Apply equivalent subjects Search modes - Proximity | Interface - EBSCOhost Research Databases Search Screen - Advanced Search Database - MEDLINE | 330 |
| S3 | (MM "Deprescriptions") | Expanders - Apply equivalent subjects Search modes - Proximity | Interface - EBSCOhost Research Databases Search Screen - Advanced Search Database - MEDLINE | 991 |
| S2 | (MH "Non-Medical Prescribing") | Expanders - Apply equivalent subjects Search modes - Proximity | Interface - EBSCOhost Research Databases Search Screen - Advanced Search Database - MEDLINE | 1 |

| | | | | |
|---|---|---|---|---|
| S1 | (MH "Pharmacists") and ((TI (prescrb*) OR AB (prescri*)) or (TX prescri*)) | Expanders - Apply equivalent subjects<br>Search modes - Proximity | Interface - EBSCOhost Research Databases<br>Search Screen - Advanced Search<br>Database - MEDLINE | 5,682 |

Embase search strategy

Research question: Q1 and Q2 (effectiveness and safety)

Search date: 18 July 2024

| Search line | Search string | Results |
|---|---|---|
| 1 | exp Pharmacist/ | 103,942 |
| 2 | exp Prescription/ | 269,654 |
| 3 | 1 and 2 | 21,695 |
| 4 | deprescribing.mp. | 2,957 |
| 5 | "non-medical prescrib*".mp. | 481 |
| 6 | (pharmacist* and (prescrib* adj3 protocol)).ti. or (pharmacist* and (prescrib* adj3 protocol)).ab. | 71 |
| 7 | (pharmacist* and prescrib* and formular*).ti. or (pharmacist* and prescrib* and formular*).ab. | 539 |
| 8 | (pharmacist* and prescrib* and ("supplementary prescribing" or "prescribing practice" or "non-medical prescri*" or "non medical prescri*")).ti. or (pharmacist* and prescrib* and ("supplementary prescribing" or "prescribing practice" or "non-medical prescri*" or "non medical prescri*")).ab. | 417 |
| 9 | (pharmacist* and prescribing and (authori* or right* or train* or "prescribing training")).ti. or (pharmacist* and prescribing and (authori* or right* or train* or "prescribing training")).ab. | 2,329 |
| 10 | pharmacist-prescriber.mp. | 119 |
| 11 | pharmacist prescriber.mp. | 119 |
| 12 | "independent prescriber*".mp. | 332 |
| 13 | "prescribing clinical pharmacist*".mp. | 6 |
| 14 | "non-medical prescriber".mp. | 61 |
| 15 | "non medical prescriber".mp. | 61 |
| 16 | "clinical pharmacist".mp. | 8,405 |
| 17 | "pharmacist-independent prescriber".mp. | 46 |
| 18 | "pharmacist independent prescriber".mp. | 46 |
| 19 | ((prescribing adj3 NMP).ti. or prescribing.mp.) adj3 NMP.ab. [mp=title, abstract, heading word, drug trade name, original title, device manufacturer, drug manufacturer, device trade name, keyword heading word, floating subheading word, candidate term word] | 69 |
| 20 | (prescribing adj3 PIP).ti. or (prescribing adj3 PIP).ab. | 285 |
| 21 | (pharmacist* adj2 prescri*).mp. and ("pharmacy (shop)"/ or hospital pharmacy/) [mp=title, abstract, heading word, drug trade name, original title, device manufacturer, drug manufacturer, device trade name, keyword heading word, floating subheading word, candidate term word] | 333 |
| 22 | ("non-medical prescribing" or "non-medical prescribing").ti. or ("non-medical prescribing" or "non-medical prescribing").ab. | 295 |
| 23 | (("pharmacist-led" or "pharmacist led") and ("non-medical prescribing" or "non-medical prescribing")).ti. or (("pharmacist-led" or "pharmacist led") and ("non-medical prescribing" or "non-medical prescribing")).ab. | 9 |

| Search line | Search string | Results |
|---|---|---|
| 24 | 3 or 5 or 6 or 7 or 8 or 9 or 10 or 11 or 12 or 13 or 14 or 15 or 19 or 21 or 22 or 23 | 23,694 |
| 25 | exp time series analysis/ | 42,052 |
| 26 | time series.mp. or time series analysis/ | 72,555 |
| 27 | exp controlled study/ or exp major clinical study/ | 13,061,958 |
| 28 | exp quasi experimental study/ or exp controlled study/ | 10,703,317 |
| 29 | quasiexperimental.mp. | 1,202 |
| 30 | ARIMA.mp. | 2,386 |
| 31 | autoregress.mp. | 2 |
| 32 | piecewise.mp. | 6,302 |
| 33 | interrupted time series.mp. | 8,209 |
| 34 | segment.mp. | 378,331 |
| 35 | (segment and regression).ti. or (segment and regression).ab. | 13,304 |
| 36 | (integrat* moving average or slope change).tw. | 2,653 |
| 37 | (piecewise or piece-wise).tw. | 6,741 |
| 38 | "controlled clinical trial (topic)"/ | 13,518 |
| 39 | exp "randomized controlled trial (topic)"/ | 277,695 |
| 40 | exp retrospective study/ | 1,649,086 |
| 41 | exp prospective study/ | 927,157 |
| 42 | pragmatic trial/ | 2,825 |
| 43 | ("randomi*ed controlled" or (random* adj2 control*)).ti. or ("randomi*ed controlled" or (random* adj2 control*)).ab. | 525,102 |
| 44 | ((time* series and (pre or before or prior)) adj5 (post or after or follow*)).ti. or ((time* series and (pre or before or prior)) adj5 (post or after or follow*)).ab. | 6,432 |
| 45 | ((time* series and (pre or before or prior)) adj5 (quasi-experiment* or quasiexperiment* or natural experiment* or ARIMA or autoregress* or auto-regress* or segmented or segments or piecewise or piece-wise or interrupt* or implement* or guideline*)).ti. or ((time* series and (pre or before or prior)) adj5 (quasi-experiment* or quasiexperiment* or natural experiment* or ARIMA or autoregress* or auto-regress* or segmented or segments or piecewise or piece-wise or interrupt* or implement* or guideline*)).ab. | 5,940 |
| 46 | 25 or 26 or 27 or 28 or 29 or 30 or 31 or 32 or 33 or 34 or 35 or 36 or 37 or 38 or 39 or 40 or 41 or 42 or 43 or 44 or 45 | 14,424,402 |
| 47 | 24 and 46 | 7,481 |
| 48 | limit 47 to (article-in-press status or embase status or in-process status) | 4,198 |
| 49 | limit 48 to "remove medline records" | 1,913 |

Dimensions.ai search strategy

Research question: Q1 and Q2 (effectiveness and safety)

Search date: 12 July 2024

| # | Query | Results |
|---|---|---|
| #1 | (((((mesh_terms:(Non-Medical Prescribing)) AND (mesh_terms:Pharmacists)) | |
| #2 | OR (mesh_terms:Deprescriptions)) AND (mesh_terms:Pharmacists)) | |
| #3 | OR (title:((pharmacist* and (supplemental or formulary or formularies or "independent prescribing" or "non-medical prescribing" or "non medical prescribing"))))) | |
| #4 | OR (abstract:((pharmacist* and (supplemental or formulary or formularies or "independent prescribing" or "non-medical prescribing" or "non medical prescribing")))) | |
| Total | | 94 |

Cochrane library search strategy

Research question: Q1 and Q2 (effectiveness and safety)

Search date: 22 July 2024

| # | Search string | Results |
| --- | --- | --- |
| #1 | MeSH descriptor: [Pharmacists] explode all trees | 1120 |
| #2 | prescribe* or prescription* or prescribed or prescriber | 48162 |
| #3 | #1 and #2 | 324 |
| #4 | MeSH descriptor: [Non-Medical Prescribing] explode all trees | 0 |
| #5 | pharmacist* N5 (deprescrib* or deprescrip*) | 1 |
| #6 | (formular* or "supplementary prescribing" or "prescribing practice" or "non-medical prescri*" or "non medical prescri*") N2 pharmacist* | 17 |
| #7 | (authority or" additional authori*" or "additional prescribing authori*ation" or right* or train* OR "prescribing training") N10 pharmacist* | 0 |
| #8 | ("pharmacist-prescriber*" OR "pharmacist prescriber*" or "independent prescrib*" or "prescribing clinical pharmacist*" or pharmacist-led or "pharmacist-independent prescriber" or "pharmacist independent prescriber") | 750 |
| #9 | "Medication Therapy Management" N2 pharmacist* | 13 |
| #10 | #3 or #4 or #5 or #6 or #7 or #8 in Cochrane Reviews, Cochrane Protocols, Trials | 991 |

Epistemonikos search strategy

Research question: Q1 and Q2 (effectiveness and safety)

Search: 22 July 2024

| | Search string |
| --- | --- |
| TITLE | ("pharmacist prescribing" OR "pharmacists prescribing" OR "Non-Medical Prescribing" OR (formulary OR formularies OR "independent prescribing") |
| | OR |
| ABSTRACT | ("pharmacist prescribing" OR "pharmacists prescribing" OR "Non-Medical Prescribing" OR (formulary OR formularies OR "independent prescribing") |
| Limit | “last 10 years” |
| RESULT | 158 |

SciELO search strategy

Research question: Q1 and Q2 (effectiveness and safety)

Search: 10 July 2024

| Search string | |
|---|---|
| (((((ti:((Pharmacist and prescribing) )) OR (ab:((Pharmacist and prescribing) ))) AND (ti:(deprescribing or deprescription)) OR (ab:(deprescribing or deprescription))) AND (ti:(pharmacist-prescriber or "pharmacist prescriber" or "independent prescriber" or "prescribing clinical pharmacist" or "clinical pharmacist" "pharmacist independent prescriber")) OR (ab:(pharmacist-prescriber or "pharmacist prescriber" or "independent prescriber" or "prescribing clinical pharmacist" or "clinical pharmacist" "pharmacist independent prescriber"))) AND (ti:(pharmacist and ("supplementary prescribing" or formulary or formularies or "prescribing protocol" or "drug protocol" or "treatment protocol" or "medical protocol" or authorization or authorisation or training or "prescribing practice"))) OR (ab:(pharmacist and ("supplementary prescribing" or formulary or formularies or "prescribing protocol" or "drug protocol" or "treatment protocol" or "medical protocol" or authorization or authorisation or training or "prescribing practice")))) AND (ti:(("non-medical prescribing" or "non medical prescribing"))) OR (ab:(("non-medical prescribing" or "non medical prescribing"))) | |
| FILTERS | No filters |
| RESULT | 41 |

# Appendix C Supplementary searches

## Search engines

| | Date | Search engine | Search strings | Results screened by IS |
|---|---|---|---|---|
| Q2 and Q3 | 09 Sept 2024 | DuckDuckGo | Deprescription or deprescribe<br>Pharmacist prescribing<br>Pharmacists' prescribing<br>Pharmacist intervention | 200 |
| | 09 Sept 2024 | Google | Deprescription or deprescribe<br>Pharmacist prescribing<br>Pharmacists' prescribing<br>Pharmacist intervention | 200 |
| Q4 | 09 Sept 2024 | DuckDuckGo | pharmacist prescribing and costs cost benefit cost analysis /<br>independent prescribing and costs cost benefit cost analysis /<br>formulary prescribing and pharmacists and costs cost benefit cost analysis /<br>deprescribing and pharmacists and costs cost benefit cost analysis / | 200 |
| | 09 Sept 2024 | Google | Economic analysis and pharmacist deprescribing<br>Economic analysis and pharmacist prescribing | 200 |

## Systematic review citation chasing: Q1, Q2, Q3 (effectiveness, safety, cost-effectiveness)

| Systematic reviews identified from Cochrane Library and Epistemonikos (n=15) |
|---|
| de Barra M, Scott CL, Scott NW, et al. Pharmacist services for non-hospitalised patients. Cochrane Database of Systematic Reviews Published Online First: 2018. doi:10.1002/14651858.CD013102 |
| de Barra M, Scott CL, Scott NW, et al. Pharmacist services for non-hospitalised patients. Cochrane Database of Systematic Reviews Published Online First: 2018. doi:10.1002/14651858.CD013102 |
| Eckhaus LM, Ti AJ, Curtis KM, et al. Patient and pharmacist perspectives on pharmacist-prescribed contraception: A systematic review. Contraception 2021;103:66–74. doi:10.1016/j.contraception.2020.10.012 |
| Eng Whui Poh, McArthur Alexa, Stephenson Matthew, et al. Effects of pharmacist prescribing on patient outcomes in the hospital setting: a systematic review. JBI Database of Systematic Reviews & Implementation Reports 2018;16:1823–73. doi:10.11124/JBISRIR-2017-003697 |
| Gillaizeau F, Chan E, Trinquart L, et al. Computerized advice on drug dosage to improve prescribing practice. Cochrane Database of Systematic Reviews Published Online First: 2013. doi:10.1002/14651858.CD002894.pub3 |
| Greer N, Bolduc J, Geurkink E, et al. Pharmacist-Led Chronic Disease Management: A Systematic Review of Effectiveness and Harms Compared to Usual Care. 2015. |
| Kamitani E, Mizuno Y, DeLuca JB, et al. Systematic review of alternative HIV pre-exposure prophylaxis (PrEP) care delivery models to improve PrEP services. AIDS (London, England) Published Online First: 2023. doi:10.1097/QAD.0000000000003601 |

| |
|---|
| Kc B, Alrasheedy AA, Leggat PA, et al. Types and outcomes of pharmacist-managed travel health services: A systematic review. Travel medicine and infectious disease 2022;51:102494. doi:10.1016/j.tmaid.2022.102494 |
| Mills T, Patel N, Ryan K. Pharmacist non-medical prescribing in primary care. A systematic review of views, opinions and attitudes. International journal of clinical practice 2020;:e13827. doi:10.1111/ijcp.13827 |
| Oñatibia-Astibia A, Malet-Larrea A, Gastelurrutia MÁ, et al. Community pharmacist interventions to improve adherence to lipid lowering medication and their influence on clinical outcomes: A systematic review and meta-analysis. Journal of evaluation in clinical practice Published Online First: 2020. doi:10.1111/jep.13451 |
| Ramos DC, Ferreira L, Santos Júnior GAD, et al. Pharmacist prescribing: a review of perceptions and attitudes of patients, pharmacists and other interested professionals. Ciencia & saude coletiva 2022;27:3531–46. doi:10.1590/1413-81232022279.19972021 |
| Ruiz-Ramos J, Hernández MH, Juanes-Borrego AM, et al. The Impact of Pharmaceutical Care in Multidisciplinary Teams on Health Outcomes: Systematic Review and Meta-Analysis. Journal of the American Medical Directors Association 2021;22:2518–26. doi:10.1016/j.jamda.2021.05.038 |
| Thakur T, Frey M, Chewning B. Pharmacist roles, training, and perceived barriers in naloxone dispensing: A systematic review. Journal of the American Pharmacists Association : JAPhA 2019;60:178–94. doi:10.1016/j.japh.2019.06.016 |
| Walpola RL, Issakhany D, Gisev N, et al. The accessibility of pharmacist prescribing and impacts on medicines access: A systematic review. Research in social & administrative pharmacy : RSAP Published Online First: 2024. doi:10.1016/j.sapharm.2024.01.006 |
| Wright DJ, Maskrey V, Blyth A, et al. Systematic review and narrative synthesis of pharmacist provided medicines optimisation services in care homes for older people to inform the development of a generic training or accreditation process. The International journal of pharmacy practice 2020;28:207–19. doi:10.1111/ijpp.12591 |
| Wu JH, Khalid F, Langford BJ, et al. Community pharmacist prescribing of antimicrobials: A systematic review from an antimicrobial stewardship perspective. Canadian pharmacists journal : CPJ = Revue des pharmaciens du Canada : RPC 2021;154:179–92. doi:10.1177/1715163521999417 |

| | |
|---|---|
| **Records identified for backward citations chasing** | 1,031 |
| **Records identified for forward citation chasing** | 434 |
| **Total** | 1,465 |

| **Systematic reviews identified through Medline, Embase, Dimensions.ai, EconLit, Econpapers database searches (n=22)** |
|---|
| Ahumada-Canale Antonio, Quirland Camila, Martinez-Mardones Francisco J, et al. Economic evaluations of pharmacist-led medication review in outpatients with hypertension, type 2 diabetes mellitus, and dyslipidaemia: a systematic review. The European journal of health economics : HEPAC : health economics in prevention and care 2019;20:1103–16. doi:10.1007/s10198-019-01080-z |
| Al Raiisi Fatma, Stewart Derek, Fernandez-Llimos Fernando, et al. Clinical pharmacy practice in the care of Chronic Kidney Disease patients: a systematic review. International journal of clinical pharmacy 2019;41:630–66. doi:10.1007/s11096-019-00816-4 |

Alabkal Rahma M, Medlinskiene Kristina, Silcock Jonathan, et al. Impact of Pharmacist-Led Interventions to Improve Clinical Outcomes for Adults With Type 2 Diabetes at Risk of Developing Cardiovascular Disease: A Systematic Review and Meta-analysis. Journal of pharmacy practice 2023;36:888–99. doi:10.1177/08971900211064459

Baumgartner Andrew D, Clark Collin M, LaValley Susan A, et al. Interventions to deprescribe potentially inappropriate medications in the elderly: Lost in translation? Journal of Clinical Pharmacy and Therapeutics 2019;45:453–61. doi:10.1111/jcpt.13103

Bužančić Iva, Kummer Ingrid, Držaić Margita, et al. Community-based pharmacists' role in deprescribing: A systematic review. British Journal of Clinical Pharmacology 2021;88:452–63. doi:10.1111/bcp.14947

Cao V F. S, Cowley E, Koshman S L, et al. Pharmacist-led optimization of heart failure medications: A systematic review. JACCP Journal of the American College of Clinical Pharmacy 2021;4:862–70. doi:10.1002/jac5.1450

Croke A, Cardwell K, Clyne B, et al. The effectiveness and cost of integrating pharmacists within general practice to optimize prescribing and health outcomes in primary care patients with polypharmacy: A systematic review. medRxiv Published Online First: 2022. doi:10.1101/2022.12.15.22283519

Croke Aisling, Cardwell Karen, Clyne Barbara, et al. The effectiveness and cost of integrating pharmacists within general practice to optimize prescribing and health outcomes in primary care patients with polypharmacy: a systematic review. BMC primary care 2023;24:41. doi:10.1186/s12875-022-01952-z

De Oliveira Gildasio S, Jr, Castro-Alves Lucas J, et al. Effectiveness of Pharmacist Intervention to Reduce Medication Errors and Health-Care Resources Utilization After Transitions of Care: A Meta-analysis of Randomized Controlled Trials. Journal of patient safety 2021;17:375–80. doi:10.1097/PTS.0000000000000283

Elnour A A, Raja N S, Abdi F, et al. Protocol for systematic review and meta-analysis of randomized controlled trials, cost-benefit analysis and interrupted time-series interventions on pharmacist's prescribing. Pharmacy Practice 2022;20:2713. doi:10.18549/PharmPract.2022.3.2713

Entezari-Maleki Taher, Dousti Samaneh, Hamishehkar Hadi, et al. A systematic review on comparing 2 common models for management of warfarin therapy; pharmacist-led service versus usual medical care. Journal of clinical pharmacology 2016;56:24–38. doi:10.1002/jcph.576

Guillaume L, Cooper R, Avery A, et al. Supplementary prescribing by community and primary care pharmacists: an analysis of PACT data, 2004-2006. Journal of clinical pharmacy and therapeutics 2008;33:11–6. doi:10.1111/j.1365-2710.2008.00869.x

Guillot J, Schott A, Roy H, et al. Evolution of pharmacy practice models in infectiology: A 30-year review. Pharmacien Hospitalier et Clinicien 2013;48:239–48. doi:10.1016/j.phclin.2013.03.003

Hou Kelu, Yang Hui, Ye Zhikang, et al. Effectiveness of Pharmacist-led Anticoagulation Management on Clinical Outcomes: A Systematic Review and Meta-Analysis. Journal of pharmacy & pharmaceutical sciences : a publication of the Canadian Society for Pharmaceutical Sciences, Societe canadienne des sciences pharmaceutiques 2017;20:378–96. doi:10.18433/J3SQ0B

Jeong Sohyun, Lee Minhee, Ji Eunhee. Effect of pharmaceutical care interventions on glycemic control in patients with diabetes: a systematic review and meta-analysis. Therapeutics and clinical risk management 2018;14:1813–29. doi:10.2147/TCRM.S169748

Nicoll Ruairidh, Robertson Lynn, Gemmell Elliot, et al. Models of care for chronic kidney disease: A systematic review. Nephrology (Carlton, Vic) 2018;23:389–96. doi:10.1111/nep.13198

Noblet Timothy, Marriott John, Graham-Clarke Emma, et al. Clinical and cost-effectiveness of non-medical prescribing: A systematic review of randomised controlled trials. PloS one 2018;13:e0193286. doi:10.1371/journal.pone.0193286

| | |
|---|---|
| Ragab M H, Al-Hindi M Y, Alrayees M M. Neonatal parenteral nutrition: Review of the pharmacist role as a prescriber. Saudi Pharmaceutical Journal 2016;24:429–40. doi:10.1016/j.jsps.2014.06.009 | |
| Stone R H, Rafie S, Ernest D, et al. Emergency contraception access and counseling in urban pharmacies: A comparison between states with and without pharmacist prescribing. Pharmacy 2020;8:1–10. doi:10.3390/pharmacy8020105 | |
| Vaismoradi Mojtaba, Jordan Sue, Logan Patricia A, et al. A Systematic Review of the Legal Considerations Surrounding Medicines Management. Medicina (Kaunas, Lithuania) 2021;57. doi:10.3390/medicina57010065 | |
| Varas-Doval R, Saéz-Benito L, Gastelurrutia M A, et al. Systematic review of pragmatic randomised control trials assessing the effectiveness of professional pharmacy services in community pharmacies. BMC health services research 2021;21:156. doi:10.1186/s12913-021-06150-8 | |
| Weeks Greg, George Johnson, Maclure Katie, et al. Non-medical prescribing versus medical prescribing for acute and chronic disease management in primary and secondary care. The Cochrane database of systematic reviews 2016;11:CD011227. doi:10.1002/14651858.CD011227.pub2 | |
| **Records identified for backward citations chasing** | 1,115 |
| **Records identified for forward citation chasing** | 478 |
| **Total identified** | 1593 |

*Table A3: Summary of systematic review citation chasing*

| Question and # of relevant systematic reviews | References retrieved | Citations retrieved | Total retrieved | Removed through deduplication | Screened on title & abstract | Included in final paper |
|---|---|---|---|---|---|---|
| Q1, Q2, Q3 systematic Reviews (n=15) from systematic review search in Cochrane Library and Epistemonikos | 1,115 | 478 | 1,593 | 1,733 | 1,325 | 8 |
| Q1, Q2, Q3 systematic Reviews (n=22) from database search | 1,031 | 434 | 1,465 | | | |
| Total | 2,319 | 1,048 | 3,367 | 1,788 | 1,570 | 8 |

## Grey literature searches

Searches completed between (August and September 2024)

| Source | Organisation | Website |
|---|---|---|
| Australia | Advanced Pharmacy Australia (formerly The Society of Hospital Pharmacists of Australia (SHPA) as of the 28 August, 2024) | https://www.adpha.au/ |
| | Australian Pharmacy Council | https://www.pharmacycouncil.org.au/ |

| Source | Organisation | Website |
|---|---|---|
| | Pharmacist Society of Australia | https://www.psa.org.au/psa-release-pharmacist-prescribing-position-statement/ |
| Canada | The Canadian Pharmacists Association (CPhA) | https://www.pharmacists.ca/ |
| | College of Pharmacists of BC | https://www.bcpharmacists.org/contact-us |
| | Deprescribing.org | https://deprescribing.org/about/ |
| | New Brunswick's Pharmacists' Association | https://nbpharma.ca/ |
| | The National Association of Pharmacy Regulatory Authorities (NAPRA) | https://www.napra.ca/resources/pharmacy-regulatory-authorities/ |
| UK | The Pharmacists' Defence Association (PDA) | https://www.the-pda.org/ |
| New Zealand | Health, Quality and Safety Commission | https://www.hqsc.govt.nz/ |
| | Pharmaceutical Society of New Zealand | https://www.psnz.org.nz/ |
| | Pharmacy Council | https://pharmacycouncil.org.nz/ |
| Databases/ International | CADTH | https://www.cadth.ca/ |
| | HIQA | https://www.hiqa.ie/ |
| | Health Evidence | https://www.healthevidence.org/ |
| | INAHTA | https://database.inahta.org/ |
| | The International Pharmaceutical Federation (FIP) | https://www.fip.org/ |
| | World Health Organization (WHO) | https://www.who.int/data/gho/gho-search |
| | London School of Economics | https://eprints.lse.ac.uk/ |
| | National Centre for Pharmacoeconomics, Ireland | https://www.ncpe.ie/about/ |
| | Econpapers | https://econpapers.repec.org/ |
| | CEA Registry | https://cear.tuftsmedicalcenter.org/ |
| | Health Evidence | https://www.healthevidence.org/ |
| | Centre for Reviews and Dissemination | https://www.crd.york.ac.uk/CRDWeb/ |

# Appendix D Data extraction form of 39 included studies

## Aspinall *et al.* 2012

### Study eligibility

| Study Characteristics | Eligibility criteria |
|---|---|
| Title | Impact of Pharmacist-Managed Erythropoiesis-Stimulating Agents Clinics for Patients With Non–Dialysis-Dependent CKD |
| Author (year) | Aspinall et al. 2012 |
| Country | Not specified, USA |
| Type of study | Retrospective cohort |
| Participants | Outpatients with non-dialysis-dependent chronic kidney disease receiving erythropoiesis-stimulating agents |
| Types of intervention | Pharmacist managed erythropoiesis-stimulating agent clinic |
| Types of comparison | 1. Physician managed erythropoiesis-stimulating agent clinic<br>2. Usual care |
| Types of outcome measures | Quality of erythropoiesis prescribing (proportion of hemoglobin values within the target range of 10-12 g/dl); average number of hemoglobin and iron tests per patient; adverse events (thromboembolica, heart failure, uncontrolled hypertension) |
| Prescriptive authority | Collaborative practice agreement |
| Include/Exclude | Include |
| Notes | None |

**DO NOT PROCEED IF STUDY EXCLUDED FROM REVIEW**

## Characteristics of included studies

### Methods

| | Descriptions as stated in report/paper | | Page number |
|---|---|---|---|
| **Aim of study** | To compare the quality of erythropoiesis-stimulating agent prescribing and monitoring for patients with non–dialysis-dependent CKD (NDD-CKD) | | 371 |
| **Design** | Retrospective cohort | | 372 |
| **Unit of allocation** *(by individuals, cluster/ groups or body parts)* | Individuals | | 372 |
| **Start-end date** | January 2009 – June 2009 | | 372 |
| **Duration of participation** *(from recruitment to last follow-up/ baseline to last follow-up- group level)* *1. Time of consent until last measurement for each individual.* *2. Baseline to final follow-up for each individual* | Six months | | 372 |
| **Study duration** *(as above with the exception of interim analyses or other circumstances* | Six months | | 372 |
| **Ethical approval needed/obtained for study** | Yes | Institutional review boards for participating sites and the VA Center for Medication Safety approved the study. | 372 |
| **Notes** | None | | |

### Participants

| | Description *Include comparative information for each intervention or comparison group if available* | Page number |
|---|---|---|

| | | | |
|---|---|---|---|
| **Population description** *(from which study participants are drawn)* | Outpatients with non-dialysis-dependent chronic kidney disease receiving erythropoiesis-stimulating agents | | 372 |
| **Setting** *(including location and social context)* | Outpatient: VA Medical Centers with either pharmacist-managed clinics or physician-based care | | 372 |
| **Inclusion/exclusion criteria** | Veterans who were "dual managed" (ie, VA and non-VA providers co-managing the ESA) at usual-care sites were included; this type of care is not permitted in pharmacist-managed ESA clinics. Because only 4%-6% of patients in each group were new to ESA therapy (ie, started ESA therapy in December 2008), our analysis focuses on patients who were receiving ESA treatment on a long-term ongoing basis. No patient was excluded based on the number of ESA doses received or hemoglobin tests ordered. | | 372 |
| **Method of recruitment of participants** | Random selection | | 372 |
| **Informed consent obtained** | Not reported | NA | NA |
| **Total no. randomised** *(or total pop. at start of study for NRCTs)* | 572 individuals | | 374 (table 1) |
| **Clusters** | NA | | NA |
| **Baseline imbalances** | Except for age, racial, and ethnicity differences between veterans managed in ESA clinics and usual care sites, baseline patient characteristics were similar in all 3 groups (pharmacist-managed ESA clinics, usual-care sites, and usual care at ESA clinics; Table 1). | | 373 |
| **Withdrawals and exclusions** | Not reported | | NA |
| **Age (years)** | Intervention: 73.9 ± 10.9<br>Comparator 1 (physician ESA clinic): 76.2 ± 12.0<br>Comparator 2 (usual care): 78.4 ± 8.8 | | 374 (table 1) |
| **Sex (female)** | Intervention: 1.9%<br>Comparator 1 (physician ESA clinic): 3.3%<br>Comparator 2 (usual care): 3.0% | | 374 (table 1) |
| **Subgroups measure** | Not reported | | NA |
| **Subgroups reported** | Not reported | | NA |
| **Notes** | None | | |

## Intervention group

| | Description as stated in report/paper | Page number |
|---|---|---|

| | | |
|---|---|---|
| **Group name** | Pharmacist managed erythropoiesis-stimulating agent clinic | 372 |
| **No. randomised/assigned to group** *(specify whether no. people or clusters)* | 314 individuals | 372 (table 1) |
| **Description** *(include sufficient detail for replication, e.g. content, dose, components)* | Pharmacists' scope of practice allowed them to dose and monitor ESA therapy. Patients at most sites were referred to the pharmacist-managed ESA clinic by a medical provider. Study clinics had been operational since at least August 1, 2008, and evidence-based protocols were in place for the management of patients receiving ESAs. | 372 |
| **Duration of treatment period** | Six months | 372 |
| **Timing** | Not reported | NA |
| **Co-interventions** | Not reported | NA |
| **Notes** | None | |

## Comparator group 1

| | Description as stated in report/paper | Page number |
|---|---|---|
| **Group name** | Physician managed erythropoiesis-stimulating agent clinic (usual care at clinic site) | 372 |
| **No. randomised/assigned to group** *(specify whether no. people or clusters)* | 91 individuals | 372 (table 1) |
| **Description** *(include sufficient detail for replication, e.g. content, dose, components)* | Erythropoiesis-stimulating agent managed by physicians in clinic | 372 |
| **Duration of treatment period** | Six months | 372 |
| **Timing** | Not reported | NA |
| **Co-interventions** | Not reported | NA |
| **Notes** | None | |

## Comparator group 2

| | Description as stated in report/paper | Page number |
|---|---|---|
| **Group name** | Usual care | 372 |

| | | |
|---|---|---|
| **No. randomised/assigned to group** *(specify whether no. people or clusters)* | 167 individuals | 372 (table 1) |
| **Description** *(include sufficient detail for replication, e.g. content, dose, components)* | Usual care (i.e. not at clinic) | 372 |
| **Duration of treatment period** | Six months | 372 |
| **Timing** | Not reported | NA |
| **Co-interventions** | Not reported | NA |
| **Notes** | None | |

## Outcomes

### Quality of erythropoiesis prescribing (proportion of hemoglobin values within the target range of 10-12 g/dl)

| | **Description as stated in report/paper** | | **Page number** |
|---|---|---|---|
| **Outcome name** | Quality of erythropoiesis prescribing (proportion of hemoglobin values within the target range of 10-12 g/dl); | | 372 |
| **Time points measured** *(specify whether from start or end of intervention)* | Six months | | 373 |
| **Time points reported** | Six months | | `373 |
| **Outcome definition** *(with diagnostic criteria if relevant)* | Proportion of hemoglobin values within the target range US Food and Drug Administration (FDA) recommended hemoglobin levels of 10-12 g/dL in patients with CKD. This guidance was consistent with the 2007 update of the NKF-KDOQI (National Kidney Foundation's Kidney Disease Outcomes Quality Initiative) guidelines, which recommend a hemoglobin target generally in the range of 11-12 g/dL | | 373, 372 |
| **Unit of measurement** *(if relevant)* | g/dL | | 373 |
| **Scales: upper and lower limits** *(indicate whether high or low score is good)* | Target range 10-12 g/dLl | | 373 |
| **Is outcome/tool validated?** | No | Electronic health record | 372 |

| | | |
|---|---|---|
| **Imputation of missing data** *(e.g. assumptions made for ITT analysis)* | Not reported | NA |
| **Assumed risk estimate** *(e.g. baseline or population risk noted in Background)* | Not reported | NA |
| **Power** *(e.g. power & sample size calculation, level of power achieved)* | Not reported | NA |
| **Notes** | None | |

## Thromboembolica (adverse event)

| | Description as stated in report/paper | | Page number |
|---|---|---|---|
| **Outcome name** | Thromboembolica (adverse event) | | 372 |
| **Time points measured** *(specify whether from start or end of intervention)* | Six months | | 373 |
| **Time points reported** | Six months | | `373 |
| **Outcome definition** *(with diagnostic criteria if relevant)* | Event resulted in emergency department visit or hospitalization. Thromboembolic events include myocardial infarction, stroke, deep vein thrombosis, and pulmonary embolism. | | 377 (table 5) |
| **Unit of measurement** *(if relevant)* | Event data | | 373 |
| **Scales: upper and lower limits** *(indicate whether high or low score is good)* | Lower score is desired | | 373 |
| **Is outcome/tool validated?** | No | Electronic health record | 372 |
| **Imputation of missing data** *(e.g. assumptions made for ITT analysis)* | Not reported | | NA |
| **Assumed risk estimate** *(e.g. baseline or population risk noted in Background)* | Not reported | | NA |
| **Power** *(e.g. power & sample size calculation, level of power achieved)* | The study was not powered to detect differences in adverse events by type of care. | | 372 |

| | |
|---|---|
| **Notes** | None |

## Heart failure (adverse event)

| | Description as stated in report/paper | | Page number |
|---|---|---|---|
| **Outcome name** | Heart failure (adverse event) | | 372 |
| **Time points measured** *(specify whether from start or end of intervention)* | Six months | | 373 |
| **Time points reported** | Six months | | `373 |
| **Outcome definition** *(with diagnostic criteria if relevant)* | Event resulted in emergency department visit or hospitalization. | | 377 (table 5) |
| **Unit of measurement** *(if relevant)* | Event data | | 373 |
| **Scales: upper and lower limits** *(indicate whether high or low score is good)* | Lower score is desired | | 373 |
| **Is outcome/tool validated?** | No | Electronic health record | 372 |
| **Imputation of missing data** *(e.g. assumptions made for ITT analysis)* | Not reported | | NA |
| **Assumed risk estimate** *(e.g. baseline or population risk noted in Background)* | Not reported | | NA |
| **Power** *(e.g. power & sample size calculation, level of power achieved)* | The study was not powered to detect differences in adverse events by type of care. | | 372 |
| **Notes** | None | | |

## Uncontrolled hypertension (adverse event)

| | Description as stated in report/paper | Page number |
|---|---|---|
| **Outcome name** | Uncontrolled hypertension (adverse event) | 372 |
| **Time points measured** *(specify whether from start or end of intervention)* | Six months | 373 |
| **Time points reported** | Six months | 373 |

| | | | |
|---|---|---|---|
| **Outcome definition** *(with diagnostic criteria if relevant)* | Systolic blood pressure >160 mm Hg or diastolic blood pressure >100 mm Hg. | | 377 (table 5) |
| **Unit of measurement** *(if relevant)* | Event data | | 373 |
| **Scales: upper and lower limits** *(indicate whether high or low score is good)* | Lower score is desired | | 373 |
| **Is outcome/tool validated?** | No | Electronic health record | 372 |
| **Imputation of missing data** *(e.g. assumptions made for ITT analysis)* | Not reported | | NA |
| **Assumed risk estimate** *(e.g. baseline or population risk noted in Background)* | Not reported | | NA |
| **Power** *(e.g. power & sample size calculation, level of power achieved)* | The study was not powered to detect differences in adverse events by type of care. | | 372 |
| **Notes** | None | | |

## Funding/conflict of interest

| | | |
|---|---|---|
| **Study funding sources** *(including role of funders)* | There was no specific funding support for the work. However, these findings are the result of work supported in kind by the VA Center for Medication Safety/VA Pharmacy Benefits Management Services, Hines, IL; VA Pittsburgh Healthcare System, Pittsburgh, PA; and the other VA medical centers that participated in the study. | 379 |
| **Possible conflicts of interest** *(for study authors)* | The authors declare that they have no relevant financial interests. | 379 |
| **Notes** | None | |

## Data and analysis

### Quality of erythropoiesis prescribing (proportion of hemoglobin values within the target range of 10-12 g/dl)

| | Description as stated in report/paper | Page number |
|---|---|---|
| **Outcome** | Quality of erythropoiesis prescribing (proportion of hemoglobin values within the target range of 10-12 g/dl) | 372 |

| | | | | | |
|---|---|---|---|---|---|
| **Time point (specify from start or end of intervention)** | Six months | | | | 372 |
| **Results** | Intervention | | Comparison 1 | | 375 (table 2) |
| | No. with event | Total doses in group | No. with event | Total doses in group | |
| | 1284 | 1807 | 179 | 346 | |
| | Intervention | | Comparison 2 | | |
| | 1284 | 1807 | 345 | 606 | |
| **Any other results reported** | p<0.001 pharmacist-managed clinics versus usual-care sites and usual care at ESA clinic sites; no differences between the latter 2 groups. | | | | 375 (table 2) |
| | Patients at usual-care sites had a significantly higher risk of hemoglobin values <10 g/dL (odds ratio [OR], 2.13; *P*< 0.009; Table 6) and >12 g/dL (OR, 3.93; *P* < 0.001);<br><br>usual-care patients at ESA clinic sites also were at higher risk of both outcomes (ORs of 2.72 for hemoglobin level less than target and 2.93 for hemoglobin level greater than target, *P* <0.001 for each). | | | | 375 |
| **No. missing participants** | 0 | | NA | | NA |
| **Reasons missing** | NA | | NA | | NA |
| **Statistical methods used and appropriateness of these** | Proportion of hemoglobin values that were within, less than, and greater than the target range of 10-12 g/dL. Random-effect multinomial models with clustering at the patient and site levels were used to compare the groups. To assess provider response to values outside the target range, we calculated and compared the average daily ESA dose during the 30 days before and after each hemoglobin measurement to ascertain whether the ESA dose was increased after a hemoglobin value less than target or held/decreased after a value greater than target. Random-effect logistic regression models with clustering at the patient and site levels were used to compare the groups. | | | | 372 |
| **Confounders** | Not reported | | | | NA |
| **Notes** | None | | | | |

## Thromboembolica (adverse event)

| | Description as stated in report/paper | Page number |
|---|---|---|
| **Outcome** | Thromboembolica (adverse event) | 372 |

| | | | | | |
|---|---|---|---|---|---|
| **Time point** *(specify from start or end of intervention)* | Six months | | | | 372 |
| **Results** | Intervention | | Comparison 1 | | 277 (table 5) |
| | No. with event | Total patient days in group | No. with event | Total patient days in group | |
| | 6 | 51077 | 3 | 13094 | |
| | Intervention | | Comparison 2 | | |
| | 6 | 51077 | 7 | 27521 | |
| **Any other results reported** | Adverse-event rates (per 180 patient-days of ESA therapy) were clinically similar across the 3 types of care. | | | | 373 |
| **No. missing participants** | 0 | | NA | | NA |
| **Reasons missing** | NA | | NA | | NA |
| **Statistical methods used and appropriateness of these** | For the clinical outcome measures of adverse events and uncontrolled hypertension, we estimated rates (per 180 person-days). The study was not powered to detect differences in adverse events by type of care. | | | | 372 |
| **Confounders** | Not reported | | | | NA |
| **Notes** | None | | | | |

## Heart failure (adverse event)

| | **Description as stated in report/paper** | | | | **Page number** |
|---|---|---|---|---|---|
| **Outcome** | Heart failure (adverse event) | | | | 372 |
| **Time point** *(specify from start or end of intervention)* | Six months | | | | 372 |
| **Results** | Intervention | | Comparison 1 | | 277 (table 5) |
| | No. with event | Total in group | No. with event | Total in group | |
| | 18 | 51077 | 7 | 13094 | |
| | Intervention | | Comparison 2 | | |
| | 18 | 51077 | 9 | 27521 | |
| **Any other results reported** | Adverse-event rates (per 180 patient-days of ESA therapy) were clinically similar across the 3 types of care. | | | | 373 |
| **No. missing participants** | NA | | NA | | NA |
| **Reasons missing** | NA | | NA | | NA |
| **Statistical methods used and appropriateness of these** | For the clinical outcome measures of adverse events and uncontrolled hypertension, we estimated rates (per 180 person-days). The study was not powered to detect differences in adverse events by type of care. | | | | 372 |
| **Confounders** | Not reported | | | | NA |
| **Notes** | None | | | | |

## Uncontrolled hypertension (adverse event)

| | Description as stated in report/paper | | | | Page number |
|---|---|---|---|---|---|
| **Outcome** | Uncontrolled hypertension (adverse event) | | | | 372 |
| **Time point** *(specify from start or end of intervention)* | Six months | | | | 372 |
| **Results** | Intervention | | Comparison 1 | | 277 (table 5) |
| | No. with event | Total in group | No. with event | Total in group | |
| | 185 | 51077 | 50 | 13094 | |
| | Intervention | | Comparison 2 | | |
| | 185 | 51077 | 73 | 27521 | |
| **Any other results reported** | Adverse-event rates (per 180 patient-days of ESA therapy) were clinically similar across the 3 types of care. | | | | 373 |
| **No. missing participants** | NA | | NA | | NA |
| **Reasons missing** | NA | | NA | | NA |
| **Statistical methods used and appropriateness of these** | For the clinical outcome measures of adverse events and uncontrolled hypertension, we estimated rates (per 180 person-days). The study was not powered to detect differences in adverse events by type of care. | | | | 372 |
| **Confounders** | Not reported | | | | NA |
| **Notes** | None | | | | |

## Conclusions

| | Description as stated in report/paper | Page number |
|---|---|---|
| **Key conclusions of study authors** | Relative to usual care, pharmacist-managed clinics provided improved quality of ESA dosing and monitoring for patients with NDD-CKD. | 371/378 |
| **Notes** | None | |

# Beahm et al. 2018

## Study eligibility

| Study Characteristics | Eligibility criteria |
|---|---|
| **Title** | Outcomes of Urinary Tract Infection Management by Pharmacists (RxOUTMAP): A study of pharmacist prescribing and care in patients with uncomplicated urinary tract infections in the community |
| **Author (year)** | Beahm et al. 2018 |
| **Country** | New Brunswick, Canada |
| **Type of study** | Prospective registry trial |
| **Participants** | Adult female patients with UTI (urinary tract infection) |
| **Types of intervention** | Pharmacist-initial arm |

| | |
|---|---|
| **Types of comparison** | Physician-initial arm |
| **Types of outcome measures** | Clinical cure at two weeks; adverse events; patient adherence; number of follow-ups, treatment failures (and reasons for); time from decision to seek care until seen by pharmacist (Pharmacist-Initial Arm) or physician (Physician-Initial Arm); patient satisfaction. |
| **Prescriptive authority** | Independent |
| **Include/Exclude** | Include |
| **Notes** | Of the enrolled patients who received a prescription from a physician first, pharmacists modified 40.4% of those initial prescriptions. |

**DO NOT PROCEED IF STUDY EXCLUDED FROM REVIEW**

## Characteristics of included studies

### Methods

| | Descriptions as stated in report/paper | | Page number |
|---|---|---|---|
| **Aim of study** | To evaluate the effectiveness, safety and patient satisfaction with pharmacist prescribing and care in patients with uncomplicated UTI. | | 305 |
| **Design** | Prospective registry trial (prospective cohort study) | | 306 |
| **Unit of allocation** *(by individuals, cluster/ groups or body parts)* | Individuals | | 307 |
| **Start-end date** | June 2017-April 2018 | | 308 |
| **Duration of participation** *(from recruitment to last follow-up)* | Two weeks | | 308 |
| **Ethical approval needed/obtained for study** | Yes | The study was approved by the Health Research Ethics Boards of the University of Alberta (Pro00072493) and the Horizon Health Network (New Brunswick) (RS 2017-2443). | 308 |
| **Notes** | None | | |

### Participants

| | **Description** *Include comparative information for each intervention or comparison group if available* | Page number |
|---|---|---|
| **Population description** *(from which study participants are drawn)* | Patients were included in the study if they were at least 19 years of age and either 1) presented to the pharmacy with symptoms suggestive of UTI without a current prescription to treat it from another health care provider (Pharmacist-Initial Arm) or 2) presented with a new prescription for the treatment of UTI from another health care provider (Physician-Initial Arm). | 307 |
| **Setting** *(including location and social context)* | 39 community pharmacies from across the province of New Brunswick. | 306/307 |

| | | | |
|---|---|---|---|
| **Inclusion/exclusion criteria** | Patients were excluded if they had signs or symptoms suggestive of pyelonephritis or systemic illness, the presence of complicating factors and if receiving an antibacterial for UTI prophylaxis. Complicating factors included male sex, pregnancy, indwelling urinary catheter, poorly controlled diabetes, chronic obstruction, nephrolithiasis, chronic renal insufficiency and immunosuppression. Patients were also excluded if it was their second or more recurrence of symptomatic UTI in the past 30 days. | | 307 |
| **Method of recruitment of participants** | Patients were included in the study if they were at least 19 years of age and presented to a community pharmacy with symptoms suggestive of a UTI or were seeking a new prescription (see population above). Pharmacists obtained consent and collected data | | 307 |
| **Informed consent obtained** | Yes | Pharmacists obtained informed consent from patients for study participation and collection of data before enrolling them in the study. | 307 |
| **Total no. randomised** *(or total pop. at start of study for NRCTs)* | N=750 | | 308 |
| **Clusters** | Not reported | | NA |
| **Baseline imbalances** | No significant baseline differences at p=0.05 were reported between arms for age, biological sex, weight, creatinine clearance, dysuria, new or increased urinary frequency, new or increased urine urgency.<br><br>Significant baseline differences at p=0.05 were reported for serum creatinine levels (higher in pharmacist-initial group), suprapubic pain (higher prevalence in physician-initial arm), and time from decision to seek care until seen (longer in physician-initial arm) | | 310 (table 1) |
| **Withdrawals and exclusions** | Loss to follow-up: n=64 | | 309 (fig 1) |
| **Age (years)** | Intervention: 40.4 ± 15.9<br>Comparator: 43.7 ± 16.1 | | 308 |
| **Sex (female)** | 100% female | | 310 (table 1) |
| **Subgroups measure** | None | | NA |
| **Subgroups reported** | None | | NA |
| **Notes** | None | | |

## Intervention group

| | Description as stated in report/paper | Page number |
|---|---|---|
| **Group name** | Pharmacist-initial | 307 |

| | | |
|---|---|---|
| **No. randomised/assigned to group** *(specify whether no. people or clusters)* | 686 individuals | 309 (fig 1) |
| **Description** *(include sufficient detail for replication, e.g. content, dose, components)* | Pharmacists performed patient assessments for symptoms of UTI and prescribed antibacterial therapy, modified antibacterial therapy, provided education only or referred to physician, as appropriate. | 307 |
| **Duration of treatment period** | Pharmacists conducted follow-ups with included patients at 2 weeks to assess for resolution of symptoms, adherence to therapy and any adverse events. | 307 |
| **Timing** | Not reported | NA |
| **Co-interventions** | Education was provided to all patients and included information on what to expect and instructions to come back if symptoms were not improving or worsening after a few days | 307 |
| **Notes** | None | |

## Comparator group

| | **Description as stated in report/paper** | **Page number** |
|---|---|---|
| **Group name** | Physician-initial | 307 |
| **No. randomised/assigned to group** *(specify whether no. people or clusters)* | 94 individuals | 309 (fig 1) |
| **Description** *(include sufficient detail for replication, e.g. content, dose, components)* | A new prescription for the treatment of UTI from another health care provider (Physician-Initial Arm). | 307 |
| **Duration of treatment period** | Pharmacists conducted follow-ups with included patients at 2 weeks to assess for resolution of symptoms, adherence to therapy and any adverse events. | 307 |
| **Timing** | Not reported | NA |
| **Co-interventions** | Not reported | NA |
| **Notes** | None | |

# Outcomes

## Clinical cure at two weeks

| | **Description as stated in report/paper** | **Page number** |
|---|---|---|
| **Outcome name** | Clinical cure at two weeks | 307 |

| | | |
|---|---|---|
| **Time points measured** *(specify whether from start or end of intervention)* | Baseline; two week follow-up | 307 |
| **Time points reported** | Two week follow-up | 307 |
| **Outcome definition** *(with diagnostic criteria if relevant)* | Sustained resolution of symptoms at two weeks | 308 |
| **Unit of measurement** *(if relevant)* | NA | NA |
| **Scales: upper and lower limits** *(indicate whether high or low score is good)* | Higher scores are desired | NA |
| **Is outcome/tool validated?** | No | NA |
| **Imputation of missing data** *(e.g. assumptions made for ITT analysis)* | Not reported | NA |
| **Assumed risk estimate** *(e.g. baseline or population risk noted in Background)* | The primary outcome for efficacy was compared to our hypothesis of 90% clinical cure | 307 |
| **Power** *(e.g. power & sample size calculation, level of power achieved)* | We estimated that with a 95% confidence level and a 5% margin of error, the sample size required would be 384 patients. This number was inflated to 500 to allow for 20% loss to follow-up and was further increased to 750 for added power in the analyses of secondary outcomes. | 307 |
| **Notes** | None | |

## Time to access care from decision to seek care

| | **Description as stated in report/paper** | **Page number** |
|---|---|---|
| **Outcome name** | Time to access care from decision to seek care | 307 |
| **Time points measured** *(specify whether from start or end of intervention)* | Baseline | 307 |
| **Time points reported** | Baseline | 307 |
| **Outcome definition** *(with diagnostic criteria if relevant)* | Time from decision to seek care | 308 |
| **Unit of measurement** *(if relevant)* | Days | NA |

| | | | |
|---|---|---|---|
| **Scales: upper and lower limits** *(indicate whether high or low score is good)* | Lower scores are desired | | NA |
| **Is outcome/tool validated?** | No | | NA |
| **Imputation of missing data** *(e.g. assumptions made for ITT analysis)* | Not reported | | NA |
| **Assumed risk estimate** *(e.g. baseline or population risk noted in Background)* | Not reported | | NA |
| **Power** *(e.g. power & sample size calculation, level of power achieved)* | We estimated that with a 95% confidence level and a 5% margin of error, the sample size required would be 384 patients. This number was inflated to 500 to allow for 20% loss to follow-up and was further increased to 750 for added power in the analyses of secondary outcomes. | | 307 |
| **Notes** | None | | |

## All adverse events

| | Description as stated in report/paper | | Page number |
|---|---|---|---|
| **Outcome name** | All adverse events | | 307 |
| **Time points measured** *(specify whether from start or end of intervention)* | Two week follow-up | | 307 |
| **Time points reported** | Two week follow-up | | |
| **Outcome definition** *(with diagnostic criteria if relevant)* | Adverse events | | 308 |
| **Unit of measurement** *(if relevant)* | NA | | NA |
| **Scales: upper and lower limits** *(indicate whether high or low score is good)* | Lower scores are desired | | NA |
| **Is outcome/tool validated?** | No | | NA |
| **Imputation of missing data** *(e.g. assumptions made for ITT analysis)* | Not reported | | NA |

| | | |
|---|---|---|
| **Assumed risk estimate** *(e.g. baseline or population risk noted in Background)* | Not reported | 307 |
| **Power** *(e.g. power & sample size calculation, level of power achieved)* | We estimated that with a 95% confidence level and a 5% margin of error, the sample size required would be 384 patients. This number was inflated to 500 to allow for 20% loss to follow-up and was further increased to 750 for added power in the analyses of secondary outcomes. | 307 |
| **Notes** | None | |

## Gastrointestinal adverse events

| | **Description as stated in report/paper** | | **Page number** |
|---|---|---|---|
| **Outcome name** | Gastrointestinal adverse events | | 307 |
| **Time points measured** *(specify whether from start or end of intervention)* | Two week follow-up | | 307 |
| **Time points reported** | Two week follow-up | | 307 |
| **Outcome definition** *(with diagnostic criteria if relevant)* | Adverse events | | 308 |
| **Unit of measurement** *(if relevant)* | NA | | NA |
| **Scales: upper and lower limits** *(indicate whether high or low score is good)* | Lower scores are desired | | NA |
| **Is outcome/tool validated?** | No | | NA |
| **Imputation of missing data** *(e.g. assumptions made for ITT analysis)* | Not reported | | NA |
| **Assumed risk estimate** *(e.g. baseline or population risk noted in Background)* | Not reported | | 307 |
| **Power** *(e.g. power & sample size calculation, level of power achieved)* | We estimated that with a 95% confidence level and a 5% margin of error, the sample size required would be 384 patients. This number was inflated to 500 to allow for 20% loss to follow-up and was further increased to 750 for added power in the analyses of secondary outcomes. | | 307 |

| | |
|---|---|
| **Notes** | None |

## Vaginal candidiasis adverse events

| | Description as stated in report/paper | | Page number |
|---|---|---|---|
| **Outcome name** | Vaginal candidiasis adverse events | | 307 |
| **Time points measured** *(specify whether from start or end of intervention)* | Two week follow-up | | 307 |
| **Time points reported** | Two week follow-up | | 307 |
| **Outcome definition** *(with diagnostic criteria if relevant)* | Secondary vaginal infection. Adverse events | | 308 |
| **Unit of measurement** *(if relevant)* | NA | | NA |
| **Scales: upper and lower limits** *(indicate whether high or low score is good)* | Lower scores are desired | | NA |
| **Is outcome/tool validated?** | No | | NA |
| **Imputation of missing data** *(e.g. assumptions made for ITT analysis)* | Not reported | | NA |
| **Assumed risk estimate** *(e.g. baseline or population risk noted in Background)* | Not reported | | 307 |
| **Power** *(e.g. power & sample size calculation, level of power achieved)* | We estimated that with a 95% confidence level and a 5% margin of error, the sample size required would be 384 patients. This number was inflated to 500 to allow for 20% loss to follow-up and was further increased to 750 for added power in the analyses of secondary outcomes. | | 307 |
| **Notes** | None | | |

## Headache adverse events

| | Description as stated in report/paper | Page number |
|---|---|---|
| **Outcome name** | Headache adverse events | 307 |
| **Time points measured** *(specify whether from start or end of intervention)* | Two week follow-up | 307 |

| | | | |
|---|---|---|---|
| **Time points reported** | Two week follow-up | | 307 |
| **Outcome definition** *(with diagnostic criteria if relevant)* | Adverse events | | 308 |
| **Unit of measurement** *(if relevant)* | NA | | NA |
| **Scales: upper and lower limits** *(indicate whether high or low score is good)* | Lower scores are desired | | NA |
| **Is outcome/tool validated?** | No | | NA |
| **Imputation of missing data** *(e.g. assumptions made for ITT analysis)* | Not reported | | NA |
| **Assumed risk estimate** *(e.g. baseline or population risk noted in Background)* | Not reported | | 307 |
| **Power** *(e.g. power & sample size calculation, level of power achieved)* | We estimated that with a 95% confidence level and a 5% margin of error, the sample size required would be 384 patients. This number was inflated to 500 to allow for 20% loss to follow-up and was further increased to 750 for added power in the analyses of secondary outcomes. | | 307 |
| **Notes** | None | | |

## Other adverse events

| | Description as stated in report/paper | Page number |
|---|---|---|
| **Outcome name** | Other adverse events | 307 |
| **Time points measured** *(specify whether from start or end of intervention)* | Two week follow-up | 307 |
| **Time points reported** | Two week follow-up | 307 |
| **Outcome definition** *(with diagnostic criteria if relevant)* | Adverse events. Included events such as insomnia or rash | 308, 311 table 3 |
| **Unit of measurement** *(if relevant)* | NA | NA |
| **Scales: upper and lower limits** *(indicate whether high or low score is good)* | Lower scores are desired | NA |

| | | | |
|---|---|---|---|
| **Is outcome/tool validated?** | No | | NA |
| **Imputation of missing data** *(e.g. assumptions made for ITT analysis)* | Not reported | | NA |
| **Assumed risk estimate** *(e.g. baseline or population risk noted in Background)* | Not reported | | 307 |
| **Power** *(e.g. power & sample size calculation, level of power achieved)* | We estimated that with a 95% confidence level and a 5% margin of error, the sample size required would be 384 patients. This number was inflated to 500 to allow for 20% loss to follow-up and was further increased to 750 for added power in the analyses of secondary outcomes. | | 307 |
| **Notes** | None | | |

## Physician or emergency department visits

| | Description as stated in report/paper | | Page number |
|---|---|---|---|
| **Outcome name** | Physician or emergency department visits | | 307 |
| **Time points measured** *(specify whether from start or end of intervention)* | Two week follow-up | | 307 |
| **Time points reported** | Two week follow-up | | 307 |
| **Outcome definition** *(with diagnostic criteria if relevant)* | Physician or emergency department visits | | 308 |
| **Unit of measurement** *(if relevant)* | NA | | NA |
| **Scales: upper and lower limits** *(indicate whether high or low score is good)* | Lower scores are desired | | NA |
| **Is outcome/tool validated?** | No | | NA |
| **Imputation of missing data** *(e.g. assumptions made for ITT analysis)* | Not reported | | NA |

| | | |
|---|---|---|
| **Assumed risk estimate** *(e.g. baseline or population risk noted in Background)* | Not reported | 307 |
| **Power** *(e.g. power & sample size calculation, level of power achieved)* | We estimated that with a 95% confidence level and a 5% margin of error, the sample size required would be 384 patients. This number was inflated to 500 to allow for 20% loss to follow-up and was further increased to 750 for added power in the analyses of secondary outcomes. | 307 |
| **Notes** | None | |

## Adherence

| | Description as stated in report/paper | | Page number |
|---|---|---|---|
| **Outcome name** | All adverse events | | 307 |
| **Time points measured** *(specify whether from start or end of intervention)* | Two week follow-up | | 307 |
| **Time points reported** | Two week follow-up | | 307 |
| **Outcome definition** *(with diagnostic criteria if relevant)* | Taken as prescribed. No doses missed. | | 308, 311 Table 4 |
| **Unit of measurement** *(if relevant)* | NA | | NA |
| **Scales: upper and lower limits** *(indicate whether high or low score is good)* | Higher scores are desired | | NA |
| **Is outcome/tool validated?** | No | | NA |
| **Imputation of missing data** *(e.g. assumptions made for ITT analysis)* | Not reported | | NA |
| **Assumed risk estimate** *(e.g. baseline or population risk noted in Background)* | Not reported | | 307 |
| **Power** *(e.g. power & sample size calculation, level of power achieved)* | We estimated that with a 95% confidence level and a 5% margin of error, the sample size required would be 384 patients. This number was inflated to 500 to allow for 20% loss to follow-up and was further increased to 750 for added power in the analyses of secondary outcomes. | | 307 |

| | |
|---|---|
| **Notes** | None |

## Funding/conflict of interest

| | | |
|---|---|---|
| **Study funding sources** *(including role of funders)* | The study was funded by investigator-initiated grants from the New Brunswick Pharmacists' Association and the Canadian Foundation for Pharmacy. The sponsors had no role in the study design, conduct, analysis/interpretation of the data or decision to publish and did not have access to the study data. | 313 |
| **Possible conflicts of interest** *(for study authors)* | The authors declared no potential conflicts of interest with respect to the research, authorship and/or publication of this article. | 314 |
| **Notes** | Pharmacist assessment fees were reimbursed to pharmacies from the study budget to allow them to waive their pharmacist assessment fees for participating patients to enhance study enrollment. | |

## Data and analysis

### Clinical cure at two weeks

| | Description as stated in report/paper | | | | Page number |
|---|---|---|---|---|---|
| **Outcome** | Clinical cure at two weeks | | | | 307 |
| **Time point** *(specify from start or end of intervention)* | Two-week follow-up | | | | 307 |
| **Results** | Intervention | | Comparison | | 310 (table 2) |
| | No. with event | Total in group | No. with event | Total in group | |
| | 528 | 596 | 82 | 90 | |
| **Any other results reported** | p>0.99 | | | | 310 (table 2) |
| **No. missing participants** | 60 | | 4 | | 309 (figure 1) |
| **Reasons missing** | Not reported | | Not reported | | NA |
| **Statistical methods used and appropriateness of these** | The primary outcome for efficacy was compared to our hypothesis of 90% clinical cure using the chi-squared test. Between-arm comparisons were conducted using the chi-squared test or Fisher's exact test for categorical variables, as appropriate, and t-test was used for continuous variables. When data deviated from normality, the Wilcoxon-Mann-Whitney test was used. | | | | 307/308 |
| **Notes** | None | | | | |

### Time to access care from decision to seek care

| | Description as stated in report/paper | Page number |
|---|---|---|

| | | | | | | | |
|---|---|---|---|---|---|---|---|
| **Outcome** | Time to access care from decision to seek care | | | | | | 308 |
| **Time point** *(specify from start or end of intervention)* | Baseline | | | | | | 308 |
| **Results** | Intervention | | | Comparison | | | 308 |
| | Mean | SD *(or other variance, specify)* | No. participants | Mean | SD *(or other variance, specify)* | No. participants | |
| | 1.7 | 2.4 | 656 | 2.8 | 3.8 | 94 | |
| **Any other results reported** *(e.g. mean difference, CI, P value)* | p= 0.0153 | | | | | | 308 |
| **No. missing participants** | 0 | | | 0 | | | |
| **Statistical methods used and appropriateness of these** *(e.g. adjustment for correlation)* | Between-arm comparisons were conducted using the chi-squared test or Fisher's exact test for categorical variables, as appropriate, and t-test was used for continuous variables. When data deviated from normality, the Wilcoxon-Mann-Whitney test was used. | | | | | | 307/308 |
| **Confounders** | None | | | | | | |
| **Notes** | None | | | | | | |

## All adverse events

| | **Description as stated in report/paper** | | | | **Page number** |
|---|---|---|---|---|---|
| **Outcome** | All adverse events | | | | 307 |
| **Time point** *(specify from start or end of intervention)* | Two-week follow-up | | | | 307 |
| **Results** | Intervention | | Comparison | | 311 (table 3) |
| | No. with event | Total in group | No. with event | Total in group | |
| | 44 | 596 | 10 | 90 | |
| **Any other results reported** | Not reported | | | | 311 (table 3) |
| **No. missing participants** | 60 | | 4 | | 309 (figure 1) |
| **Reasons missing** | Not reported | | Not reported | | NA |

| | | |
|---|---|---|
| **Statistical methods used and appropriateness of these** | The primary outcome for efficacy was compared to our hypothesis of 90% clinical cure using the chi-squared test. Between-arm comparisons were conducted using the chi-squared test or Fisher's exact test for categorical variables, as appropriate, and t-test was used for continuous variables. When data deviated from normality, the Wilcoxon-Mann-Whitney test was used. | 307/308 |
| **Notes** | None | |

## Gastrointestinal adverse events

| | **Description as stated in report/paper** | | | | **Page number** |
|---|---|---|---|---|---|
| **Outcome** | Gastrointestinal adverse events | | | | 307 |
| **Time point** *(specify from start or end of intervention)* | Two-week follow-up | | | | 307 |
| **Results** | Intervention | | Comparison | | 311 (table 3) |
| | No. with event | Total in group | No. with event | Total in group | |
| | 27 | 596 | 5 | 90 | |
| **Any other results reported** | P=0.7895 | | | | 311 (table 3) |
| **No. missing participants** | 60 | | 4 | | 309 (figure 1) |
| **Reasons missing** | Not reported | | Not reported | | NA |
| **Statistical methods used and appropriateness of these** | The primary outcome for efficacy was compared to our hypothesis of 90% clinical cure using the chi-squared test. Between-arm comparisons were conducted using the chi-squared test or Fisher's exact test for categorical variables, as appropriate, and t-test was used for continuous variables. When data deviated from normality, the Wilcoxon-Mann-Whitney test was used. | | | | 307/308 |
| **Notes** | None | | | | |

## Vaginal candidiasis adverse events

| | **Description as stated in report/paper** | | | | **Page number** |
|---|---|---|---|---|---|
| **Outcome** | Vaginal candidiasis adverse events | | | | 307 |
| **Time point** *(specify from start or end of intervention)* | Two-week follow-up | | | | 307 |
| **Results** | Intervention | | Comparison | | 311 (table 3) |
| | No. with event | Total in group | No. with event | Total in group | |
| | 5 | 596 | 3 | 90 | |
| **Any other results reported** | p=0.1079 | | | | 311 (table 3) |

| | | | |
|---|---|---|---|
| **No. missing participants** | 60 | 4 | 309 (figure 1) |
| **Reasons missing** | Not reported | Not reported | NA |
| **Statistical methods used and appropriateness of these** | | | |
| **Notes** | None | | |

## Headache adverse events

| | Description as stated in report/paper | | | | Page number |
|---|---|---|---|---|---|
| **Outcome** | Headache adverse events | | | | 307 |
| **Time point** *(specify from start or end of intervention)* | Two-week follow-up | | | | 307 |
| **Results** | Intervention | | Comparison | | 311 (table 3) |
| | No. with event | Total in group | No. with event | Total in group | |
| | 6 | 596 | 0 | 90 | |
| **Any other results reported** | p=0.7551 | | | | 311 (table 3) |
| **No. missing participants** | 60 | | 4 | | 309 (figure 1) |
| **Reasons missing** | Not reported | | Not reported | | NA |
| **Statistical methods used and appropriateness of these** | The primary outcome for efficacy was compared to our hypothesis of 90% clinical cure using the chi-squared test. Between-arm comparisons were conducted using the chi-squared test or Fisher's exact test for categorical variables, as appropriate, and t-test was used for continuous variables. When data deviated from normality, the Wilcoxon-Mann-Whitney test was used. | | | | 307/308 |
| **Notes** | None | | | | |

## Other adverse events

| | Description as stated in report/paper | | | | Page number |
|---|---|---|---|---|---|
| **Outcome** | Other adverse events | | | | 307 |
| **Time point** *(specify from start or end of intervention)* | Two-week follow-up | | | | 307 |
| **Results** | Intervention | | Comparison | | 311 (table 3) |
| | No. with event | Total in group | No. with event | Total in group | |
| | 6 | 596 | 2 | 90 | |
| **Any other results reported** | p=0.5963 | | | | 311 (table 3) |
| **No. missing participants** | 60 | | 4 | | 309 (figure 1) |
| **Reasons missing** | Not reported | | Not reported | | NA |

| | | | | | |
|---|---|---|---|---|---|
| **Statistical methods used and appropriateness of these** | The primary outcome for efficacy was compared to our hypothesis of 90% clinical cure using the chi-squared test. Between-arm comparisons were conducted using the chi-squared test or Fisher's exact test for categorical variables, as appropriate, and t-test was used for continuous variables. When data deviated from normality, the Wilcoxon-Mann-Whitney test was used. | | | | 307/308 |
| **Notes** | None | | | | |

## Physician or emergency department visits

| | Description as stated in report/paper | | | | Page number |
|---|---|---|---|---|---|
| **Outcome** | Physician or emergency department visits | | | | 307 |
| **Time point** *(specify from start or end of intervention)* | Two-week follow-up | | | | 307 |
| **Results** | Intervention | | Comparison | | 311 (table 3) |
| | No. with event | Total in group | No. with event | Total in group | |
| | 3 | 596 | 2 | 90 | |
| **Any other results reported** | p=0.2273 | | | | 311 (table 3) |
| **No. missing participants** | 60 | | 4 | | 309 (figure 1) |
| **Reasons missing** | Not reported | | Not reported | | NA |
| **Statistical methods used and appropriateness of these** | The primary outcome for efficacy was compared to our hypothesis of 90% clinical cure using the chi-squared test. Between-arm comparisons were conducted using the chi-squared test or Fisher's exact test for categorical variables, as appropriate, and t-test was used for continuous variables. When data deviated from normality, the Wilcoxon-Mann-Whitney test was used. | | | | 307/308 |
| **Notes** | None | | | | |

## Adherence

| | Description as stated in report/paper | | | | Page number |
|---|---|---|---|---|---|
| **Outcome** | Adherence | | | | |
| **Time point** *(specify from start or end of intervention)* | Two-week follow-up | | | | 307 |
| **Results** | Intervention | | Comparison | | 311 (table 4) |
| | No. with event | Total in group | No. with event | Total in group | |
| | 575 | 596 | 81 | 90 | |
| **Any other results reported** | p=0.0008 | | | | 311 (table 4) |

| | | | |
|---|---|---|---|
| **No. missing participants** | 60 | 4 | 309 (figure 1) |
| **Reasons missing** | Not reported | Not reported | NA |
| **Statistical methods used and appropriateness of these** | The primary outcome for efficacy was compared to our hypothesis of 90% clinical cure using the chi-squared test. Between-arm comparisons were conducted using the chi-squared test or Fisher's exact test for categorical variables, as appropriate, and t-test was used for continuous variables. When data deviated from normality, the Wilcoxon-Mann-Whitney test was used. | | 307/308 |
| **Notes** | None | | |

## Conclusions

| | **Description as stated in report/paper** | **Page number** |
|---|---|---|
| **Key conclusions of study authors** | We found that pharmacist management of UTI was highly efficacious and safe. Importantly, we also found that patient satisfaction with this clinical service was very high, especially in the areas of thoroughness of the assessment, accessibility and trust in the care provided by their pharmacists. | 309 |
| **Notes** | None | |

# Beahm et al. 2021

## Study eligibility

| **Study Characteristics** | **Eligibility criteria** |
|---|---|
| **Title** | Antimicrobial utilization and stewardship in patients with uncomplicated urinary tract infections managed by pharmacists in the community: A sub-study of the RxOUTMAP trial |
| **Author (year)** | Beahm et al. 2021 |
| **Country** | New Brunswick, Canada |
| **Type of study** | Prospective registry trial |
| **Participants** | Adult female patients with UTI (urinary tract infection) |
| **Types of intervention** | Pharmacist-initial arm |
| **Types of comparison** | Physician-initial arm |
| **Types of outcome measures** | Prescribing appropriateness |
| **Prescriptive authority** | Independent |

| | |
|---|---|
| **Include/Exclude** | Include |
| **Notes** | Of the enrolled patients who received a prescription from a physician first, pharmacists modified 40.4% of those initial prescriptions. |

**DO NOT PROCEED IF STUDY EXCLUDED FROM REVIEW**

## Characteristics of included studies

### Methods

| | Descriptions as stated in report/paper | | Page number |
|---|---|---|---|
| **Aim of study** | Our objective was to further evaluate the appropriateness of antibacterial prescribing in the RxOUTMAP study. | | 206 |
| **Design** | Prospective registry trial | | 206 |
| **Unit of allocation** *(by individuals, cluster/ groups or body parts)* | Individuals | | 206 |
| **Start-end date** | June 2017-April 2018 | | 207 |
| **Duration of participation** *(from recruitment to last follow-up)* | Two weeks | | 206 |
| **Ethical approval needed/obtained for study** | Yes | The study was approved by the Health Research Ethics Boards of the University of Alberta (Pro00072493) and the Horizon Health Network (New Brunswick) (RS 2017-2443). | 207 |
| **Notes** | None | | |

### Participants

| | **Description** *Include comparative information for each intervention or comparison group if available* | Page number |
|---|---|---|
| **Population description** *(from which study participants are drawn)* | Either patients presented to the pharmacy with symptoms suggestive of UTI without having just received a prescription to treat it from another health care provider (pharmacist-initial arm), or they presented with a new prescription for UTI treatment from another health care provider (physician-initial arm). | 206 |
| **Setting** *(including location and social context)* | 39 community pharmacies from across the province of New Brunswick. | 206 |
| **Inclusion/exclusion criteria** | Patients were excluded if they had complicating factors or signs or symptoms suggestive of pyelonephritis or systemic illness. | 206 |

| | | | |
|---|---|---|---|
| **Method of recruitment of participants** | Patients were included in the study if they were at least 19 years of age and either 1) presented to the pharmacy with symptoms suggestive of UTI without a current prescription to treat it from another health care provider (Pharmacist-Initial Arm) or 2) presented with a new prescription for the treatment of UTI from another health care provider (Physician-Initial Arm). | | 307 (Beahm et al. 2018) |
| **Informed consent obtained** | Yes | Patients provided informed consent to participate in the study. | 211 |
| **Total no. randomised** *(or total pop. at start of study for NRCTs)* | N=750 | | 207 |
| **Clusters** | Not applicable | | NA |
| **Baseline imbalances** | No significant baseline differences at p=0.05 were reported between arms for age, biological sex, weight, creatinine clearance, dysuria, new or increased urinary frequency, new or increased urine urgency, recent urine cultures with bacterial growth, past 3 months,<br><br>Significant baseline differences at p=0.05 were reported for serum creatinine levels (higher in pharmacist-initial group), suprapubic pain (higher prevalence in physician-initial arm), and recent antibacterial exposure (higher in physician-initial arm).<br><br>Patients in the physician-initial arm were more likely to have received any antibacterial agent (for any indication) in the 3 months preceding their presentation; however, of those who had recent exposure to an antibacterial, there was no significant difference between study arms in having recently received nitrofurantoin | | 208 (table 1), 207 |
| **Withdrawals and exclusions** | None | | 309 (fig 1, Beahm et al. 2018) |
| **Age (years)** | Intervention: 40.4 ± 15.9<br>Comparator: 43.7 ± 16.1 | | 207 |
| **Sex (female)** | Intervention: 100%<br>Comparator: 100% | | 207 |
| **Subgroups measure** | None | | NA |
| **Subgroups reported** | None | | NA |
| **Notes** | None | | |

## Intervention group

| | Description as stated in report/paper | Page number |
|---|---|---|

| | | |
|---|---|---|
| **Group name** | Pharmacist-initial | 209 (fig 1) |
| **No. randomised/assigned to group** *(specify whether no. people or clusters)* | 656 individuals | 209 (fig 1) |
| **Description** *(include sufficient detail for replication, e.g. content, dose, components)* | Pharmacists performed patient assessments for symptoms of UTI and prescribed antibacterial therapy, modified antibacterial therapy, provided education only or referred to physician, as appropriate. | 206 |
| **Duration of treatment period** | 3 to 14 days | 209 (fig 2) |
| **Timing** | Not reported | NA |
| **Co-interventions** | Education was provided to all patients and included instructions to return if symptoms did not improve or worsened after a few days. | 206 |
| **Notes** | None | |

## Comparator group

| | Description as stated in report/paper | Page number |
|---|---|---|
| **Group name** | Physician-initial | 209 (fig 1) |
| **No. randomised/assigned to group** *(specify whether no. people or clusters)* | 94 individuals | 209 (fig 1) |
| **Description** *(include sufficient detail for replication, e.g. content, dose, components)* | A new prescription for the treatment of UTI from another health care provider (Physician-Initial Arm). | 206 |
| **Duration of treatment period** | Pharmacists conducted follow-ups with included patients at 2 weeks to assess for resolution of symptoms, adherence to therapy and any adverse events. | 209 (fig 2) |
| **Timing** | Not reported | NA |
| **Co-interventions** | Not reported | NA |
| **Notes** | None | |

# Outcomes

## Antibacterial therapy guideline concordance

| | Description as stated in report/paper | Page number |
|---|---|---|
| **Outcome name** | Antibacterial therapy guideline concordance | 207 |

| | | |
|---|---|---|
| **Time points measured** *(specify whether from start or end of intervention)* | Initial antibacterials prescribed | 207 |
| **Time points reported** | Initial antibacterials prescribed | 207 |
| **Outcome definition** *(with diagnostic criteria if relevant)* | Antibacterial therapy was considered guideline concordant when appropriate daily oral dosages of nitrofurantoin (for 5 d), trimethoprim– sulfamethoxazole (TMP–SMX) (3 d), TMP (3 d), fosfomycin (1 dose), or cefuroxime axetil (7 d) were prescribed or if circumstances warranted using a regimen outside of these first-line options. These regimens are consistent with local guidelines that were made available to study pharmacists, as well as with international guidelines. Other outcomes included descriptions of which antibacterials were prescribed and for what duration, as well as prescribing to optimize guideline-discordant therapy from physicians. These outcomes were prespecified before the initiation of the study. | 207 |
| **Unit of measurement** *(if relevant)* | NA | NA |
| **Scales: upper and lower limits** *(indicate whether high or low score is good)* | Higher scores are desired | NA |
| **Is outcome/tool validated?** | No | NA |
| **Imputation of missing data** *(e.g. assumptions made for ITT analysis)* | Not reported | NA |
| **Assumed risk estimate** *(e.g. baseline or population risk noted in Background)* | Not reported | NA |
| **Power** *(e.g. power & sample size calculation, level of power achieved)* | Between-arm comparisons used $\chi^2$ or Fisher exact tests for categorical variables, as appropriate, and *t*-test was used for continuous variables. The Wilcoxon–Mann–Whitney test was used when data deviated from normality. | 207 |
| **Notes** | None | |

## Funding/conflict of interest

| | | |
|---|---|---|
| **Study funding sources** *(including role of funders)* | This study was funded by investigator-initiated grants from the New Brunswick Pharmacists' Association and the Canadian Foundation for Pharmacy. | 211 |

| | | |
|---|---|---|
| **Possible conflicts of interest** *(for study authors)* | RTT receives personal fees from Shoppers Drug Mart, personal fees from Emergent BioSystems, grants from Merck Canada, and grants from Sanofi, outside the submitted work. | 211 |
| **Notes** | None | |

## Data and analysis

### Antibacterial therapy guideline concordance

| | **Description as stated in report/paper** | | | | **Page number** |
|---|---|---|---|---|---|
| **Outcome** | Antibacterial therapy guideline concordance | | | | 207 |
| **Time point** *(specify from start or end of intervention)* | Initial antibacterials prescribed | | | | 207 |
| **Results** | Intervention | | Comparison | | 207 |
| | No. with event | Total in group | No. with event | Total in group | |
| | 624 | 656 | 33 | 94 | |
| **Any other results reported** | p<0.001 | | | | 207 |
| **No. missing participants** | 0 | | 0 | | 207 |
| **Reasons missing** | NA | | NA | | NA |
| **Statistical methods used and appropriateness of these** | Between-arm comparisons used $\chi^2$ or Fisher exact tests for categorical variables, as appropriate, and *t*-test was used for continuous variables. The Wilcoxon–Mann–Whitney test was used when data deviated from normality. | | | | 207 |
| **Notes** | None | | | | |

## Conclusions

| | **Description as stated in report/paper** | **Page number** |
|---|---|---|
| **Key conclusions of study authors** | Our study demonstrates that antimicrobial prescribing by pharmacists for uncomplicated UTI is highly guideline concordant, with physicians representing usual-care prescribing for longer treatment durations and more fluoroquinolones. This represents an important opportunity for antimicrobial stewardship interventions by pharmacists in the community. | 210 |
| **Notes** | None | |

# Boddy et al. 2001

## Study eligibility

| **Study Characteristics** | **Eligibility criteria** |
|---|---|
| **Title** | Pharmacist involvement with warfarin dosing for inpatients |
| **Author (year)** | Boddy 2001 |
| **Country** | England |
| **Type of study** | Non-randomised controlled trial |
| **Participants** | Patient being prescribed warfarin |
| **Types of intervention** | Haematology pharmacist prescribed following new guidelines |
| **Types of comparison** | Consultant physician and junior doctor prescribed following prescription |
| **Types of outcome measures** | Proportion of INRs within therapeutic range; proportion of INRs <2.0; proportion of INRs >6.0; number of days to reach INR >2.0; number of INRs requested? |
| **Prescriptive authority** | Per protocol |
| **Include/Exclude** | Include |
| **Notes** | Per protocol prescribing p.31 |

**DO NOT PROCEED IF STUDY EXCLUDED FROM REVIEW**

## Characteristics of included studies

### Methods

| | Descriptions as stated in report/paper | | Page number |
|---|---|---|---|
| **Aim of study** | To evaluate the anticoagulation control on the medical wards<br>To implement warfarin guidelines<br>To investigate the benefits of the guidelines for the doctors compared to pharmacists | | 31 |
| **Design** | Non-randomised controlled trial | | 31 |
| **Unit of allocation** *(by individuals, cluster/ groups or body parts)* | Individuals | | 31 |
| **Start-end date** | Not reported | | NA |
| **Duration of participation** *(from recruitment to last follow-up)* | Four weeks | | 31 |
| **Data collection period** | 12 weeks | | 31 |
| **Ethical approval needed/obtained for study** | Not reported | | NA |
| **Notes** | | | |

### Participants

| | **Description** *Include comparative information for each intervention or comparison group if available* | | Page number |
|---|---|---|---|
| **Population description** *(from which study participants are drawn)* | Warfarin dosing by the doctors was investigated over 4 weeks on 4 acute medical wards for all medical patients not elderly care. | | 31 |
| **Setting** *(including location and social context)* | Four acute medical wards | | 31 |
| **Method of recruitment of participants** | Not reported | | NA |
| **Informed consent obtained** | Not reported | | NA |
| **Total no. randomised** *(or total pop. at start of study for NRCTs)* | 138 individuals | | 32 (table 1) |
| **Clusters** | NA | | NA |

| | | |
|---|---|---|
| **Baseline imbalances** | The patients in each study group were well matched in terms of age, sex and indication for warfarin (p<0.01). | 32 |
| **Withdrawals and exclusions** | Not reported | NA |
| **Age (years)** | Intervention: 54 (range 22 - 74)<br>Comparator: 57 (range 23 - 74) | 32 (table 1) |
| **Sex (female)** | Intervention (mean): 51.4%<br>Comparator (mean): 46.9% | 32 (table 1) |
| **Subgroups measure** | Not reported | NA |
| **Subgroups reported** | Not reported | NA |
| **Notes** | 'The results for patients already established on warfarin were better in all cases compared to those for patients commenced on warfarin in hospital.' Does not provide baseline imbalances. | |

## Intervention group

| | Description as stated in report/paper | Page number |
|---|---|---|
| **Group name** | Pharmacist dosing | 32 (table 1) |
| **No. randomised/assigned to group** *(specify whether no. people or clusters)* | 74 | 32 (table 1) |
| **Description** *(include sufficient detail for replication, e.g. content, dose, components)* | Pharmacists dosing warfarin | 32 |
| **Duration of treatment period** | Not reported | NA |
| **Timing** | Not reported | NA |
| **Co-interventions** | NA | NA |
| **Notes** | None | |

## Comparator group

| | Description as stated in report/paper | Page number |
|---|---|---|
| **Group name** | Doctor dosing with guidelines | 32 (table 1) |
| **No. randomised/assigned to group** *(specify whether no. people or clusters)* | 64 | 32 (table 1) |
| **Description** *(include sufficient detail for replication, e.g. content, dose, components)* | Doctors dosing warfarin | 32 (table 1) |

| | | |
|---|---|---|
| **Duration of treatment period** | Not reported | NA |
| **Timing** | Not reported | NA |
| **Co-interventions** | NA | NA |
| **Notes** | None | |

## Outcomes

### INR control achieved

| | **Description as stated in report/paper** | | **Page number** |
|---|---|---|---|
| **Outcome name** | INR control achieved | | 32 |
| **Time points measured** *(specify whether from start or end of intervention)* | Not reported | | NA |
| **Time points reported** | Not reported | | NA |
| **Outcome definition** *(with diagnostic criteria if relevant)* | Proportion of INRs within the therapeutic range over the project period | | 32 |
| **Unit of measurement** *(if relevant)* | INR | | 32 |
| **Scales: upper and lower limits** *(indicate whether high or low score is good)* | Therapeutic range not specified by authors, assuming between 2.0 and 6.0 | | 32 |
| **Is outcome/tool validated?** | No | | 32 |
| **Imputation of missing data** *(e.g. assumptions made for ITT analysis)* | Not reported | | NA |
| **Assumed risk estimate** *(e.g. baseline or population risk noted in Background)* | Not reported | | NA |
| **Power** *(e.g. power & sample size calculation, level of power achieved)* | The study period for this stage was 12 weeks, as advised by the statistician to enable sufficient patient numbers to be recorded. | | 31 |
| **Notes** | None | | |

### INR <2.0

| | **Description as stated in report/paper** | **Page number** |
|---|---|---|
| **Outcome name** | INR control achieved | 32 |

| | | |
|---|---|---|
| **Time points measured** *(specify whether from start or end of intervention)* | Not reported | NA |
| **Time points reported** | Not reported | NA |
| **Outcome definition** *(with diagnostic criteria if relevant)* | Patient are at greatest risk of clotting in this situation | 32 |
| **Unit of measurement** *(if relevant)* | INR | 32 |
| **Scales: upper and lower limits** *(indicate whether high or low score is good)* | Therapeutic range not specified by authors, assuming between 2.0 and 6.0 | 32 |
| **Is outcome/tool validated?** | No | 32 |
| **Imputation of missing data** *(e.g. assumptions made for ITT analysis)* | Not reported | NA |
| **Assumed risk estimate** *(e.g. baseline or population risk noted in Background)* | Not reported | NA |
| **Power** *(e.g. power & sample size calculation, level of power achieved)* | The study period for this stage was 12 weeks, as advised by the statistician to enable sufficient patient numbers to be recorded. | 31 |
| **Notes** | None | |

## INR >6.0

| | Description as stated in report/paper | Page number |
|---|---|---|
| **Outcome name** | INR control achieved | 32 |
| **Time points measured** *(specify whether from start or end of intervention)* | Not reported | NA |
| **Time points reported** | Not reported | NA |
| **Outcome definition** *(with diagnostic criteria if relevant)* | Associated with increased risk of bleeding | 32 |

| | | | |
|---|---|---|---|
| **Unit of measurement** *(if relevant)* | INR | | 32 |
| **Scales: upper and lower limits** *(indicate whether high or low score is good)* | Therapeutic range not specified by authors, assuming between 2.0 and 6.0 | | 32 |
| **Is outcome/tool validated?** | No | | 32 |
| **Imputation of missing data** *(e.g. assumptions made for ITT analysis)* | Not reported | | NA |
| **Assumed risk estimate** *(e.g. baseline or population risk noted in Background)* | Not reported | | NA |
| **Power** *(e.g. power & sample size calculation, level of power achieved)* | The study period for this stage was 12 weeks, as advised by the statistician to enable sufficient patient numbers to be recorded. | | 31 |
| **Notes** | None | | |

## Funding/conflict of interest

| | | |
|---|---|---|
| **Study funding sources** *(including role of funders)* | Not reported | NA |
| **Possible conflicts of interest** *(for study authors)* | Not reported | NA |
| **Notes** | None | |

## Data and analysis

### INR within therapeutic range

| | **Description as stated in report/paper** | | | | **Page number** |
|---|---|---|---|---|---|
| **Outcome** | INR within therapeutic range | | | | 32 |
| **Time point** *(specify from start or end of intervention)* | Day 4 onwards | | | | 32 |
| **Results** | Intervention | | Comparison | | 32 (table 1) |
| | No. with event | Total in group | No. with event | Total in group | |
| | 43 | 74 | 12 | 64 | |
| **Any other results reported** | p=0.001 | | | | 32 |
| **No. missing participants** | Not reported | | | | NA |

| | | | | | |
|---|---|---|---|---|---|
| **Reasons missing** | NA | | | | NA |
| **Statistical methods used and appropriateness of these** | Chi squared | | | | 32 |
| **Notes** | None | | | | |

## INR <2.0

| | **Description as stated in report/paper** | | | | **Page number** |
|---|---|---|---|---|---|
| **Outcome** | INR within therapeutic range | | | | 32 |
| **Time point** *(specify from start or end of intervention)* | Day 4 onwards | | | | 32 |
| **Results** | Intervention | | Comparison | | 32 (table 1) |
| | No. with event | Total in group | No. with event | Total in group | |
| | 7 | 74 | 19 | 64 | |
| **Any other results reported** | Not significant | | | | 32 |
| **No. missing participants** | Not reported | | | | NA |
| **Reasons missing** | NA | | | | NA |
| **Statistical methods used and appropriateness of these** | Chi squared | | | | 32 |
| **Notes** | None | | | | |

## INR >6.0

| | **Description as stated in report/paper** | | | | **Page number** |
|---|---|---|---|---|---|
| **Outcome** | INR within therapeutic range | | | | 32 |
| **Time point** *(specify from start or end of intervention)* | Day 4 onwards | | | | 32 |
| **Results** | Intervention | | Comparison | | 32 (table 1) |
| | No. with event | Total in group | No. with event | Total in group | |
| | 1 | 74 | 3 | 64 | |
| **Any other results reported** | Not significant | | | | 32 |
| **No. missing participants** | Not reported | | | | NA |
| **Reasons missing** | NA | | | | NA |
| **Statistical methods used and appropriateness of these** | Chi squared | | | | 32 |

| | |
|---|---|
| **Notes** | None |

## Conclusions

| | Description as stated in report/paper | Page number |
|---|---|---|
| **Key conclusions of study authors** | The study demonstrated a significant improvement in INR control with the pharmacist dosing when compared to the results achieved by the doctors. | 34 |
| **Notes** | None | |

# Bruhn et al. 2013

## Study eligibility

| Study Characteristics | Eligibility criteria |
|---|---|
| **Title** | Pharmacist-led management of chronic pain in primary care: results from a randomised controlled exploratory trial |
| **Author (year)** | Bruhn et al. 2013 |
| **Country** | Scotland and England |
| **Type of study** | Randomised controlled exploratory trial |
| **Participants** | Patients on repeat prescribed pain medication |
| **Types of intervention** | Pharmacist medication review with face-to-face pharmacist prescribing |
| **Types of comparison** | 1. Pharmacist medication review with feedback to GP and no planned patient contact<br>2. Treatment as usual (TAU) |
| **Types of outcome measures** | SF-12v2, the Chronic Pain Grade (CPG), the Health Utilities Index, and the Hospital Anxiety and Depression Scale |
| **Prescriptive authority** | Independent |
| **Include/Exclude** | Include |
| **Notes** | |

**DO NOT PROCEED IF STUDY EXCLUDED FROM REVIEW**

## Characteristics of included studies

### Methods

| | Descriptions as stated in report/paper | | Page number |
|---|---|---|---|
| **Aim of study** | To compare the effectiveness of pharmacist medication review, with or without pharmacist prescribing, with standard care, for patients with chronic pain. | | 1 |
| **Design** | An exploratory randomised controlled trial | | 1 |
| **Unit of allocation** *(by individuals, cluster/ groups or body parts)* | Individuals | | 1 |
| **Start-end date** | March 2010-January 2011 | | 2 |
| **Duration of participation** *(from recruitment to last follow-up)* | Six months | | 2 |
| **Study duration** | Six months | | 2 |
| **Ethical approval needed/obtained for study** | Yes | Ethical approval was granted by the National Research Ethics Service Committee—North of Scotland (reference number 09/S0801/107). HS Research and Development approval was granted by NHS Grampian and East Norfolk & Waveney Research Governance Committees. | 2 |
| **Notes** | None | | |

### Participants

| | **Description** *Include comparative information for each intervention or comparison group if available* | Page number |
|---|---|---|
| **Population description** *(from which study participants are drawn)* | Patients registered with the recruited practices were eligible for inclusion if they were over 18 years of age, living in their own houses and receiving regular prescribed medication for pain. | 2 |
| **Setting** *(including location and social context)* | General practices with prescribing pharmacists | 1 |

| | | | |
|---|---|---|---|
| **Inclusion/exclusion criteria** | Patients registered with the recruited practices were eligible for inclusion if they were over 18 years of age, living in their own houses and receiving regular prescribed medication for pain.<br><br>Patients were identified by a computerised search of the drug records of all individuals registered with the practice, to identify those who had received either two or more acute prescriptions, and/or one repeat prescription within the last 120 days, for an analgesic (British National Formulary (BNF section 4.7) and/or NSAID (BNF section 10.1.1).<br><br>Medications that can be used for analgesia but whose primary indication is not chronic pain (eg triptans, antiepileptics or antidepressants) were excluded as these drugs identify few additional eligible patients.<br><br>In accordance with trial criteria, GPs excluded and recorded reasons for patients who had: a concomitant severe mental health problem or terminal illness; had suffered recent bereavement; had a known alcohol or drug addiction; suffered pain caused by cancer or other malignancy; were unable to give informed consent; other (unspecified) reasons. | | 2 |
| **Method of recruitment of participants** | Practices in the Grampian Health Board area, Scotland (n=18) and East Anglia region of England (n=4) known to have an attached Royal Pharmaceutical Society of Great Britain registered independent pharmacist prescriber, were eligible to take part. From those indicating a willingness to participate, convenience sampling was used to identify six general practices: three in Grampian and three in East Anglia.<br>Eligible patients were sent an invitation pack (letter, information sheet, consent form) by practice staff between March and June 2010. Consent forms were returned directly to the researchers, who sent out a baseline questionnaire. | | 2 |
| **Informed consent obtained** | Yes | Patients gave informed consent before taking part. | 2 |
| **Total no. randomised** *(or total pop. at start of study for NRCTs)* | 196 | | 4 (figure 1) |
| **Clusters** | NA | | NA |

| | | |
|---|---|---|
| **Baseline imbalances** | There was no statistically significant difference between participants and non-participants in terms of age, gender and index of multiple deprivation. | 6 |
| **Withdrawals and exclusions** | 23% drop-out rate across three arms | 4 (figure 1) |
| **Age (years)** | Intervention (pharmacist medication review and prescribing): 66.1 ± 12.1<br>Comparator 1 (pharmacist medication review): 65.7 ± 14.2<br>Comparator 2 (treatment as usual): 64.9 ± 11.6 | 4 (figure 1) |
| **Sex (female)** | Pharmacist medication review and prescribing: n=37 (54.4%)<br>Pharmacist medication review: n=46 (74.2%)<br>Treatment as usual: n=37 (58.7%) | 4 (figure 1) |
| **Subgroups measure** | NA | NA |
| **Subgroups reported** | NA | NA |
| **Notes** | None | |

## Intervention group

| | **Description as stated in report/paper** | **Page number** |
|---|---|---|
| **Group name** | Pharmacist medication review and prescribing | 3 |
| **No. randomised/assigned to group** *(specify whether no. people or clusters)* | 70 individuals | 4 (figure 1) |
| **Description** *(include sufficient detail for replication, e.g. content, dose, components)* | Pharmacists conducted a paper-based medication review of each patient's medical record and patients were asked to complete a pain diary to inform the consultation. A pharmaceutical care plan was agreed between the pharmacist and the patient. At the end of the consultation any required prescriptions for medicines were issued by the pharmacist.<br><br>Owing to Controlled Drug (CD) regulations in place at the time, prescribing for CDs was performed using a supplementary prescribing CMP,17 rather than independent prescribing. | 3 |
| **Duration of treatment period** | Six months | 3 |
| **Timing** | Not reported | 3 |
| **Co-interventions** | Medication review | 3 |
| **Notes** | None | |

## Comparator group 1

| | Description as stated in report/paper | Page number |
|---|---|---|
| **Group name** | Pharmacist medication review | 3 |
| **No. randomised/assigned to group** *(specify whether no. people or clusters)* | 63 | 4 (figure 1) |
| **Description** *(include sufficient detail for replication, e.g. content, dose, components)* | The pharmacists conducted a paper-based medication review focussed on pain-related prescription medications, before creating a pharmaceutical care plan which detailed any recommendations for medication changes. The plan was passed to the patient's GP for implementation. The GPs were asked subsequently about actions taken as a result of the recommendations. | 3 |
| **Duration of treatment period** | Six months | 3 |
| **Timing** | Not reported | 3 |
| **Co-interventions** | None | 3 |
| **Notes** | None | |

### Comparator group 2

| | Description as stated in report/paper | Page number |
|---|---|---|
| **Group name** | Treatment as usual | 3 |
| **No. randomised/assigned to group** *(specify whether no. people or clusters)* | 63 individuals | 4 (figure 1) |
| **Description** *(include sufficient detail for replication, e.g. content, dose, components)* | Patients received standard general practice care. | 3 |
| **Duration of treatment period** | Six months | 3 |
| **Timing** | Not reported | 3 |
| **Co-interventions** | None | 3 |
| **Notes** | None | |

## Outcomes

### Chronic pain intensity and disability

| | Description as stated in report/paper | Page number |
|---|---|---|
| **Outcome name** | Chronic pain intensity | 3 |

| | | | |
|---|---|---|---|
| **Time points measured** *(specify whether from start or end of intervention)* | Baseline, six month follow-up | | 3 |
| **Time points reported** | Questionnaires were posted to participants at baseline (pre-randomisation) and 3 and 6 months post-randomisation (follow-up was conducted between July 2010 and January 2011). | | 3 |
| **Outcome definition** *(with diagnostic criteria if relevant)* | Chronic pain intensity and disability<br><br>The CPG is a seven-item scale which assesses pain severity on two dimensions: disability and intensity. The scale classifies pain according to the level of intensity and disability (I (low disability–low intensity) to IV (high disability–severely limiting)). | | 3 |
| **Unit of measurement** *(if relevant)* | NA | | NA |
| **Scales: upper and lower limits** *(indicate whether high or low score is good)* | Lower scores are desired | | NA |
| **Is outcome/tool validated?** | Yes | Chronic Pain Grade (CPG) | NA |
| **Imputation of missing data** *(e.g. assumptions made for ITT analysis)* | Not reported | | NA |
| **Assumed risk estimate** *(e.g. baseline or population risk noted in Background)* | Not reported | | NA |
| **Power** *(e.g. power & sample size calculation, level of power achieved)* | As this was an exploratory trial to estimate the effect size for a larger trial, no formal sample size calculation was possible. We aimed to recruit 30 participants per practice (n=180; with an additional six per practice for training purposes, that is, 216 in total). This was deemed sufficient to give reliable effect size estimates for the outcome measures of health status or CPG. | | 3 |
| **Notes** | None | | |

## Health-related quality of life

| | Description as stated in report/paper | Page number |
|---|---|---|
| **Outcome name** | Health related quality of life | 3 |

| | | | |
|---|---|---|---|
| **Time points measured** *(specify whether from start or end of intervention)* | Baseline, six month follow-up | | 3 |
| **Time points reported** | Questionnaires were posted to participants at baseline (pre-randomisation) and 3 and 6 months post-randomisation (follow-up was conducted between July 2010 and January 2011). | | 3 |
| **Outcome definition** *(with diagnostic criteria if relevant)* | Health-related quality of life | | 3 |
| **Unit of measurement** *(if relevant)* | NA | | NA |
| **Scales: upper and lower limits** *(indicate whether high or low score is good)* | Higher scores are desired | | NA |
| **Is outcome/tool validated?** | Yes | Health Utilities Index | 3 |
| **Imputation of missing data** *(e.g. assumptions made for ITT analysis)* | Not reported | | NA |
| **Assumed risk estimate** *(e.g. baseline or population risk noted in Background)* | Not reported | | NA |
| **Power** *(e.g. power & sample size calculation, level of power achieved)* | As this was an exploratory trial to estimate the effect size for a larger trial, no formal sample size calculation was possible. We aimed to recruit 30 participants per practice (n=180; with an additional six per practice for training purposes, that is, 216 in total). This was deemed sufficient to give reliable effect size estimates for the outcome measures of health status or CPG. | | 3 |
| **Notes** | None | | |

## Quality of life (physical)

| | Description as stated in report/paper | Page number |
|---|---|---|
| **Outcome name** | Quality of life physical | 3 |
| **Time points measured** *(specify whether from start or end of intervention)* | Baseline, six month follow-up | 3 |

| | | | |
|---|---|---|---|
| **Time points reported** | Questionnaires were posted to participants at baseline (pre-randomisation) and 3 and 6 months post-randomisation (follow-up was conducted between July 2010 and January 2011). | | 3 |
| **Outcome definition** *(with diagnostic criteria if relevant)* | Quality of life physical component | | 3 |
| **Unit of measurement** *(if relevant)* | NA | | NA |
| **Scales: upper and lower limits** *(indicate whether high or low score is good)* | Higher scores are desired | | NA |
| **Is outcome/tool validated?** | Yes | SF-12 | 3 |
| **Imputation of missing data** *(e.g. assumptions made for ITT analysis)* | Not reported | | NA |
| **Assumed risk estimate** *(e.g. baseline or population risk noted in Background)* | Not reported | | NA |
| **Power** *(e.g. power & sample size calculation, level of power achieved)* | As this was an exploratory trial to estimate the effect size for a larger trial, no formal sample size calculation was possible. We aimed to recruit 30 participants per practice (n=180; with an additional six per practice for training purposes, that is, 216 in total). This was deemed sufficient to give reliable effect size estimates for the outcome measures of health status or CPG. | | 3 |
| **Notes** | None | | |

## Quality of life (mental health)

| | Description as stated in report/paper | Page number |
|---|---|---|
| **Outcome name** | Health related quality of life | 3 |
| **Time points measured** *(specify whether from start or end of intervention)* | Baseline, six month follow-up | 3 |
| **Time points reported** | Questionnaires were posted to participants at baseline (pre-randomisation) and 3 and 6 months post-randomisation (follow-up was conducted between July 2010 and January 2011). | 3 |

| | | | |
|---|---|---|---|
| **Outcome definition** *(with diagnostic criteria if relevant)* | Quality of life mental health component | | 3 |
| **Unit of measurement** *(if relevant)* | NA | | NA |
| **Scales: upper and lower limits** *(indicate whether high or low score is good)* | Higher scores are desired | | NA |
| **Is outcome/tool validated?** | Yes | SF-12 | 3 |
| **Imputation of missing data** *(e.g. assumptions made for ITT analysis)* | Not reported | | NA |
| **Assumed risk estimate** *(e.g. baseline or population risk noted in Background)* | Not reported | | NA |
| **Power** *(e.g. power & sample size calculation, level of power achieved)* | As this was an exploratory trial to estimate the effect size for a larger trial, no formal sample size calculation was possible. We aimed to recruit 30 participants per practice (n=180; with an additional six per practice for training purposes, that is, 216 in total). This was deemed sufficient to give reliable effect size estimates for the outcome measures of health status or CPG. | | 3 |
| **Notes** | None | | |

## Depression

| | Description as stated in report/paper | Page number |
|---|---|---|
| **Outcome name** | Depression | 3 |
| **Time points measured** *(specify whether from start or end of intervention)* | Baseline, six month follow-up | 3 |
| **Time points reported** | Questionnaires were posted to participants at baseline (pre-randomisation) and 3 and 6 months post-randomisation (follow-up was conducted between July 2010 and January 2011). | 3 |
| **Outcome definition** *(with diagnostic criteria if relevant)* | Depression | 3 |
| **Unit of measurement** *(if relevant)* | NA | NA |

| | | | |
|---|---|---|---|
| **Scales: upper and lower limits** *(indicate whether high or low score is good)* | Lower scores are desired.<br>7 items; each item scored from 0 (not present) to 3 (highly present). Standard thresholds and previously used labels were applied: no depression/anxiety (0-7), mild (8-10), moderate (11-15) or severe (>15). | | 3 |
| **Is outcome/tool validated?** | Yes | Hospital Anxiety and Depression Scale (HAD-S) | NA |
| **Imputation of missing data** *(e.g. assumptions made for ITT analysis)* | Not reported | | NA |
| **Assumed risk estimate** *(e.g. baseline or population risk noted in Background)* | Not reported | | NA |
| **Power** *(e.g. power & sample size calculation, level of power achieved)* | As this was an exploratory trial to estimate the effect size for a larger trial, no formal sample size calculation was possible. We aimed to recruit 30 participants per practice (n=180; with an additional six per practice for training purposes, that is, 216 in total). This was deemed sufficient to give reliable effect size estimates for the outcome measures of health status or CPG. | | 3 |
| **Notes** | None | | |

## Anxiety

| | Description as stated in report/paper | Page number |
|---|---|---|
| **Outcome name** | Anxiety | 3 |
| **Time points measured** *(specify whether from start or end of intervention)* | Baseline, six month follow-up | 3 |
| **Time points reported** | Questionnaires were posted to participants at baseline (pre-randomisation) and 3 and 6 months post-randomisation (follow-up was conducted between July<br>2010 and January 2011). | 3 |
| **Outcome definition** *(with diagnostic criteria if relevant)* | Anxiety | 3 |
| **Unit of measurement** *(if relevant)* | NA | NA |

| | | | |
|---|---|---|---|
| **Scales: upper and lower limits** *(indicate whether high or low score is good)* | Lower scores are desired.<br>7 items; each item scored from 0 (not present) to 3 (highly present). Standard thresholds and previously used labels were applied: no depression/anxiety (0-7), mild (8-10), moderate (11-15) or severe (>15). | | NA |
| **Is outcome/tool validated?** | Yes | Hospital Anxiety and Depression Scale (HAD-S) | NA |
| **Imputation of missing data** *(e.g. assumptions made for ITT analysis)* | Not reported | | NA |
| **Assumed risk estimate** *(e.g. baseline or population risk noted in Background)* | Not reported | | NA |
| **Power** *(e.g. power & sample size calculation, level of power achieved)* | As this was an exploratory trial to estimate the effect size for a larger trial, no formal sample size calculation was possible. We aimed to recruit 30 participants per practice (n=180; with an additional six per practice for training purposes, that is, 216 in total). This was deemed sufficient to give reliable effect size estimates for the outcome measures of health status or CPG. | | 3 |
| **Notes** | None | | |

## Funding/conflict of interest

| | | |
|---|---|---|
| **Study funding sources** *(including role of funders)* | The project was funded by the Medical Research Council (grant ID: 85356). They had no further involvement in any aspect of study conduct; all researchers were independent of the funding body. | 11 |
| **Possible conflicts of interest** *(for study authors)* | Authors stated no conflicts of interest. | 11 |
| **Notes** | None | |

## Data and analysis

### Chronic pain intensity

| | **Description as stated in report/paper** | **Page number** |
|---|---|---|
| **Outcome** | Chronic pain: Intensity | 7 (table 2) |
| **Time point** *(specify from start or end of intervention)* | Six month follow-up | 7 (table 2) |

| | | | | | | | | | | |
|---|---|---|---|---|---|---|---|---|---|---|
| **Results (baseline intensity)** | Intervention | | | Comparison | | | Comparison 2 (TAU) | | | 7 (table 2) |
| | Mean | SD | No. pp | Mean | SD | No. pp | Mean | SD | No. pp | |
| | 66.1 | 16.0 | 68 | 68.4 | 17.6 | 63 | 65.4 | 18.0 | 63 | |
| **Results (Six month follow-up intensity)** | Intervention | | | Comparison | | | Comparison 2 (TAU) | | | 7 (table 2) |
| | Mean | SD | No. pp | Mean | SD | No. pp | Mean | SD | No. pp | |
| | 58.1 | 19.5 | 44 | 67.4 | 21.7 | 45 | 65.6 | 19.6 | 53 | |
| **Results (baseline disability)** | Intervention | | | Comparison | | | Comparison 2 (TAU) | | | 7 (table 2) |
| | Median | IQR | No. pp | Median | IQR | No. pp | Median | IQR | No. pp | |
| | 60.0 | 30.0; 75.8 | 68 | 66.7 | 45.0; 80.0 | 63 | 56.7 | 36.7; 80.0 | 63 | |
| **Results (Six month follow-up disability)** | Intervention | | | Comparison | | | Comparison 2 (TAU) | | | 7 (table 2) |
| | Median | IQR | No. pp | Median | IQR | No. pp | Median | IQR | No. pp | |
| | 40.0 | 20.0;60.0 | 48 | 53.3 | 29.2; 73.3 | 46 | 50.0 | 25.0; 80.0 | 53 | |
| **Any other results reported** *(e.g. mean difference, CI, P value)* | Intensity between groups p=0.02<br>Disability between groups: p=0.55 | | | | | | | | | 7 (table 2) |
| **No. missing participants** | N=54 missing/withdrawals at 3 and 6 month follow-up<br>70 allocated to intervention -> 44 returned 6 month questionnaire<br>63 allocated to comparison 1 -> 45 returned 6 month questionnaire<br>63 allocated to comparison 2 -> 53 returned 6 month questionnaire | | | | | | | | | 4 (table 1) |
| **Statistical methods used and appropriateness of these** *(e.g. adjustment for correlation)* | Exploratory analyses for parametric data included the paired t-test for within-arm comparisons of mean difference between baseline and 6 months and one-way analysis of variance for between arm comparisons of mean difference. For non-parametric data it included the Wilcoxon signed rank test for within-arm comparisons of median difference and the Kruskal-Wallis test for between arm comparisons of median difference. Categorical data were analysed using the marginal homogeneity test for within-arm comparisons (with null hypothesis that the distribution of CPG grade or HADS group does not change between baseline and 6 month follow-up) and the $\chi^2$ test for between-arm comparisons; analyses reported here are based on 6 month follow-up data (other than for participant experiences). Within arm effect sizes, expressed in terms of a Pearson correlation coefficient (r) have been calculated using the formulas from Rosenthal. Effect sizes can be directly compared using Cohen's29 criteria of r=0.1 (small effect); r=0.3 (medium effect) and r=0.5 (large effect). | | | | | | | | | 5/6 |

| | |
|---|---|
| **Notes** | None |

## Health-related quality of life

| | **Description as stated in report/paper** | | | | | | | | | **Page number** |
|---|---|---|---|---|---|---|---|---|---|---|
| **Outcome** | Health-related quality of life | | | | | | | | | Not reported |
| **Time point** *(specify from start or end of intervention)* | Not reported | | | | | | | | | Not reported |
| **Results (baseline)** | Intervention | | | Comparison | | | Comparison 2 (TAU) | | | |
| | Mean | SD | No. pp | Mean | SD | No. pp | Mean | SD | No. pp | |
| | | | | | | | | | | |
| **Results (Six month follow-up)** | Intervention | | | Comparison | | | Comparison 2 (TAU) | | | |
| | Mean | SD | No. pp | Mean | SD | No. pp | Mean | SD | No. pp | |
| | | | | | | | | | | |
| **Any other results reported** *(e.g. mean difference, CI, P value)* | Not reported | | | | | | | | | Not reported |
| **No. missing participants** | Not reported | | | | | | | | | Not reported |
| **Statistical methods used and appropriateness of these** *(e.g. adjustment for correlation)* | Not reported | | | | | | | | | Not reported |
| **Notes** | No information given in paper | | | | | | | | | |

## Quality of life (physical)

| | **Description as stated in report/paper** | | | | | | | | | **Page number** |
|---|---|---|---|---|---|---|---|---|---|---|
| **Outcome** | Quality of life (physical) | | | | | | | | | 8 (table 3) |
| **Time point** *(specify from start or end of intervention)* | Six month follow-up | | | | | | | | | 8 (table 3) |
| **Results (Six month follow-up)** | Intervention | | | Comparison | | | Comparison 2 (TAU) | | | 8 (table 3) |
| | Mean | SD | No. pp | Mean | SD | No. pp | Mean | SD | No. pp | |
| | 35.3 | 10.8 | 41 | 34.62 | 11.26 | 43 | 32.59 | 9.14 | 45 | |

| | | |
|---|---|---|
| **Any other results reported** *(e.g. mean difference, CI, P value)* | In contrast, the SF-12, a measure of general health and functionality showed no significant difference between intervention arms, reflecting either no effect or lack of power to detect an effect. | 10 |
| **No. missing participants** | N=57 missing/withdrawals at 3 and 6 month follow-up | 8 (table 3) |
| **Statistical methods used and appropriateness of these** *(e.g. adjustment for correlation)* | Exploratory analyses for parametric data included the paired t-test for within-arm comparisons of mean difference between baseline and 6 months and one-way analysis of variance for between arm comparisons of mean difference. For non-parametric data it included the Wilcoxon signed rank test for within-arm comparisons of median difference and the Kruskal-Wallis test for between arm comparisons of median difference. Categorical data were analysed using the marginal homogeneity test for within-arm comparisons (with null hypothesis that the distribution of CPG grade or HADS group does not change between baseline and 6 month follow-up) and the $\chi^2$ test for between-arm comparisons; analyses reported here are based on 6 month follow-up data (other than for participant experiences). Within arm effect sizes, expressed in terms of a Pearson correlation coefficient (r) have been calculated using the formulas from Rosenthal. Effect sizes can be directly compared using Cohen's29 criteria of r=0.1 (small effect); r=0.3 (medium effect) and r=0.5 (large effect). | 5/6 |
| **Notes** | None | |

## Quality of life (mental health)

| | Description as stated in report/paper | | | | | | | | | Page number |
|---|---|---|---|---|---|---|---|---|---|---|
| **Outcome** | Quality of life (mental health) | | | | | | | | | 8 (table 3) |
| **Time point** *(specify from start or end of intervention)* | Six month follow-up | | | | | | | | | 8 (table 3) |
| **Results (Six month follow-up)** | Intervention | | | Comparison | | | Comparison 2 (TAU) | | | 8 (table 3) |
| | Median | IQR | No. pp | Median | IQR | No. pp | Median | IQR | No. pp | |
| | 49.6 | 42.8, 58.1 | 42 | 47.9 | 38.9, 56.2 | 43 | 51.5 | 41.3, 60.7 | 45 | |
| **Any other results reported** *(e.g. mean difference, CI, P value)* | In contrast, the SF-12, a measure of general health and functionality showed no significant difference between intervention arms, reflecting either no effect or lack of power to detect an effect. | | | | | | | | | 10 |
| **No. missing participants** | N=56 missing/withdrawals at 3 and 6 month follow-up | | | | | | | | | 8 (table 3) |

| | | |
|---|---|---|
| **Statistical methods used and appropriateness of these** *(e.g. adjustment for correlation)* | Exploratory analyses for parametric data included the paired t-test for within-arm comparisons of mean difference between baseline and 6 months and one-way analysis of variance for between arm comparisons of mean difference. For non-parametric data it included the Wilcoxon signed rank test for within-arm comparisons of median difference and the Kruskal-Wallis test for between arm comparisons of median difference. Categorical data were analysed using the marginal homogeneity test for within-arm comparisons (with null hypothesis that the distribution of CPG grade or HADS group does not change between baseline and 6 month follow-up) and the $\chi^2$ test for between-arm comparisons; analyses reported here are based on 6 month follow-up data (other than for participant experiences). Within arm effect sizes, expressed in terms of a Pearson correlation coefficient (r) have been calculated using the formulas from Rosenthal. Effect sizes can be directly compared using Cohen's29 criteria of r=0.1 (small effect); r=0.3 (medium effect) and r=0.5 (large effect). | 5/6 |
| **Notes** | None | |

## Depression

<table>
<tr><th></th><th colspan="9">Description as stated in report/paper</th><th>Page number</th></tr>
<tr><td>Outcome</td><td colspan="9">Depression</td><td>9 (table 5)</td></tr>
<tr><td>Time point (specify from start or end of intervention)</td><td colspan="9">Six month follow-up</td><td>9 (table 5)</td></tr>
<tr><td rowspan="3">Results (baseline)</td><td colspan="3">Intervention</td><td colspan="3">Comparison</td><td colspan="3">Comparison 2 (TAU)</td><td rowspan="3">9 (table 5)</td></tr>
<tr><td>Median</td><td>IQR</td><td>No. pp</td><td>Median</td><td>IQR</td><td>No. pp</td><td>Median</td><td>IQR</td><td>No. pp</td></tr>
<tr><td>5.0</td><td>3.0, 8.0</td><td>68</td><td>4.5</td><td>2.3, 8.0</td><td>63</td><td>5.0</td><td>3.0, 8.0</td><td>63</td></tr>
<tr><td rowspan="3">Results (Six month follow-up)</td><td colspan="3">Intervention</td><td colspan="3">Comparison</td><td colspan="3">Comparison 2 (TAU)</td><td rowspan="3">9 (table 5)</td></tr>
<tr><td>Median</td><td>IQR</td><td>No. pp</td><td>Median</td><td>IQR</td><td>No. pp</td><td>Median</td><td>IQR</td><td>No. pp</td></tr>
<tr><td>4.0</td><td>2.0, 8.0</td><td>42</td><td>5.0</td><td>2.0, 8.8</td><td>44</td><td>5.0</td><td>2.0, 10.0</td><td>51</td></tr>
<tr><td>Any other results reported (e.g. mean difference, CI, P value)</td><td colspan="9">Between groups p=0.02</td><td>9 (table 5)</td></tr>
<tr><td>No. missing participants</td><td colspan="9">N=54 missing/withdrawals at 3 and 6 month follow-up</td><td>4 (table 1)</td></tr>
</table>

| | | |
|---|---|---|
| **Statistical methods used and appropriateness of these** *(e.g. adjustment for correlation)* | Exploratory analyses for parametric data included the paired t-test for within-arm comparisons of mean difference between baseline and 6 months and one-way analysis of variance for between arm comparisons of mean difference. For non-parametric data it included the Wilcoxon signed rank test for within-arm comparisons of median difference and the Kruskal-Wallis test for between arm comparisons of median difference. Categorical data were analysed using the marginal homogeneity test for within-arm comparisons (with null hypothesis that the distribution of CPG grade or HADS group does not change between baseline and 6 month follow-up) and the $\chi^2$ test for between-arm comparisons; analyses reported here are based on 6 month follow-up data (other than for participant experiences). Within arm effect sizes, expressed in terms of a Pearson correlation coefficient (r) have been calculated using the formulas from Rosenthal. Effect sizes can be directly compared using Cohen's29 criteria of r=0.1 (small effect); r=0.3 (medium effect) and r=0.5 (large effect). | 5/6 |
| **Notes** | None | |

## Anxiety

| | **Description as stated in report/paper** | | | | | | | | | **Page number** |
|---|---|---|---|---|---|---|---|---|---|---|
| **Outcome** | Anxiety | | | | | | | | | 9 (table 5) |
| **Time point** *(specify from start or end of intervention)* | Six month follow-up | | | | | | | | | 9 (table 5) |
| **Results (baseline)** | Intervention | | | Comparison | | | Comparison 2 (TAU) | | | 9 (table 5) |
| | Median | IQR | No. pp | Median | IQR | No. pp | Median | IQR | No. pp | |
| | 7.0 | 3.3, 10.8 | 68 | 5.0 | 3.0, 10.0 | 63 | 6.0 | 4.0, 10.0 | 63 | |
| **Results (Six month follow-up)** | Intervention | | | Comparison | | | Comparison 2 (TAU) | | | 9 (table 5) |
| | Median | IQR | No. pp | Median | IQR | No. pp | Median | IQR | No. pp | |
| | 5.0 | 2.3, 9.8 | 44 | 5.0 | 3.0, 10.0 | 43 | 7.0 | 4.0, 10.0 | 48 | |
| **Any other results reported** *(e.g. mean difference, CI, P value)* | Between groups p=0.05 | | | | | | | | | 9 (table 5) |
| **No. missing participants** | N=54 missing/withdrawals at 3 and 6 month follow-up | | | | | | | | | 4 (table 1) |

| | | |
|---|---|---|
| **Statistical methods used and appropriateness of these** *(e.g. adjustment for correlation)* | Exploratory analyses for parametric data included the paired t-test for within-arm comparisons of mean difference between baseline and 6 months and one-way analysis of variance for between arm comparisons of mean difference. For non-parametric data it included the Wilcoxon signed rank test for within-arm comparisons of median difference and the Kruskal-Wallis test for between arm comparisons of median difference. Categorical data were analysed using the marginal homogeneity test for within-arm comparisons (with null hypothesis that the distribution of CPG grade or HADS group does not change between baseline and 6 month follow-up) and the $\chi^2$ test for between-arm comparisons; analyses reported here are based on 6 month follow-up data (other than for participant experiences). Within arm effect sizes, expressed in terms of a Pearson correlation coefficient (r) have been calculated using the formulas from Rosenthal. Effect sizes can be directly compared using Cohen's29 criteria of r=0.1 (small<br>effect); r=0.3 (medium effect) and r=0.5 (large effect). | 5/6 |
| **Notes** | None | |
| **Notes** | None | |

## Conclusions

| | Description as stated in report/paper | Page number |
|---|---|---|
| **Key conclusions of study authors** | Our results suggest that pharmacist prescribing (and possibly pharmacist review alone) for patients with chronic pain is feasible, acceptable and may lead to improvements in pain and other measures. | 10 |
| **Notes** | None | |

# Chenella et al. 1983

## Study eligibility

| Study Characteristics | Eligibility criteria |
|---|---|
| **Title** | Comparison of Physician and Pharmacist Management of Anticoaguiant Therapy of Inpatients |
| **Author (year)** | Chenella et al. 1983 |
| **Country** | California, US |
| **Type of study** | Trial |
| **Participants** | Adult inpatients requiring anticoagulant services |
| **Types of intervention** | Pharmacist prescriber |
| **Types of comparison** | Physician prescriber |

| | |
|---|---|
| **Types of outcome measures** | Prescribing patterns; anticoagulant doses; clinical responses (partial thromboplastin time, days to therapeutic doe);adverse events (major bleeding, minor bleeding, and all) |
| **Prescriptive Authority** | Per protocol |
| **Include/Exclude** | Include |
| **Notes** | |

**DO NOT PROCEED IF STUDY EXCLUDED FROM REVIEW**

## Characteristics of included studies

### Methods

| | Descriptions as stated in report/paper | | Page number |
|---|---|---|---|
| **Aim of study** | The purpose of the study was to determine the ability of the pharmacist to independently adjust heparin and warfarin dosages. In this paper, the results of that study are reported. | | 1642 |
| **Design** | Randomised control trial | | 1643 |
| **Unit of allocation** *(by individuals, cluster/ groups or body parts)* | Individuals | | 1643 |
| **Start-end date** | May-October 1980 | | 1643 |
| **Duration of participation** *(from recruitment to last follow-up)* | Not reported | | NA |
| **Study duration** | Until therapeutic range met.<br>The heparin dose was adjusted daily… Twenty-four hours after this degree of warfarin effect was attained, the heparin infusion was discontinued and daily adjustments were then made by the referring physician | | 1643 |
| **Ethical approval needed/obtained for study** | Yes | The institutional research committee approved the study. | 1644 |
| **Notes** | None | | |

### Participants

| | **Description**<br>*Include comparative information for each intervention or comparison group if available* | Page number |
|---|---|---|
| **Population description** *(from which study participants are drawn)* | Eighty-one consecutive hospitalized patients who were referred to the anticoagulant service by their primary physicians | 1643 |
| **Setting** *(including location and social context)* | Hospital | 1643 |
| **Inclusion/exclusion criteria** | Not reported | NA |
| **Method of recruitment of participants** | Eighty-one consecutive hospitalized patients who were referred to the anticoagulant service by their primary physicians | 1643 |

| | | | |
|---|---|---|---|
| **Informed consent obtained** | Not reported | | NA |
| **Total no. randomised** *(or total pop. at start of study for NRCTs)* | 81 individuals | | 1643 |
| **Clusters** | NA | | |
| **Baseline imbalances** | Demographic and baseline laboratory data for the two groups are listed in Table 2 [Age, No. men, Height, Weight, Baseline creatinine, Baseline PCV(%), Baseline P&P, Platelets on smear]. There were no significant differences observed for any of these factors. Table 3 lists the initial diagnoses for all patients [Pulmonary embolism, Venous thrombosis, Cardiac prophylaxis, Arterial surgery, Cerebrovascular thrombosis]. There were no significant differences between the two groups with respect to the reason for anticoagulant therapy. | | 1644 |
| **Withdrawals and exclusions** | One patient in the physician-prescriber group died while in the study. (on third day of treatment).<br>Unclear if her data was treated as normal. | | 1645 |
| **Age (years)** | Intervention: 46.0 ± 16.0<br>Comparator: 52 ± 16.0 | | 1644 (table 2) |
| **Sex (female)** | Intervention (mean): 54.8%<br>Comparator mean): 59.0% | | 1644 (table 2) |
| **Subgroups measure** | Prescribing patterns | | 1644 (table 5) |
| **Subgroups reported** | First seven patients and last seven patients in each arm. | | 1644 (table 5) |
| **Notes** | The mean values for heparin dose and PTT during the first 24 hours of treatment were not significantly different between study groups (Table 5). There were no significant differences in mean heparin dose and PTT between the first and last seven patients assigned to the pharmacist-prescriber group. Likewise, there were no significant differences in these variables between the first and last seven patients in the physician-prescriber group. 1644 | | |

## Intervention group

| | Description as stated in report/paper | Page number |
|---|---|---|
| **Group name** | Pharmacist prescriber | 1643 |
| **No. randomised/assigned to group** *(specify whether no. people or clusters)* | 42 | 1644 (table 2) |
| **Description** *(include sufficient detail for replication, e.g. content, dose, components)* | Patients in the pharmacist-prescriber group had a pharmacist write daily heparin and warfarin dosage adjustments. | 1643 |

| | | |
|---|---|---|
| **Duration of treatment period** | Not reported | NA |
| **Timing** | Not reported | NA |
| **Co-interventions** | Not reported | NA |
| **Notes** | None | |

### Comparator group

| | Description as stated in report/paper | Page number |
|---|---|---|
| **Group name** | Pharmacist prescriber | 1643 |
| **No. randomised/assigned to group** *(specify whether no. people or clusters)* | 42 | 1644 (table 2) |
| **Description** *(include sufficient detail for replication, e.g. content, dose, components)* | The dosage determined by the prescribing physician was given to patients in the physician-prescriber group, and dosages simulated by a pharmacist were not given. | 1643 |
| **Duration of treatment period** | Not reported | NA |
| **Timing** | Not reported | NA |
| **Co-interventions** | Not reported | NA |
| **Notes** | None | |

### Outcomes

#### Partial thromboplastin time (PTT)

| | Description as stated in report/paper | Page number |
|---|---|---|
| **Outcome name** | Partial thromboplastin time | 1643 |
| **Time points measured** *(specify whether from start or end of intervention)* | Initial blood sample obtained 3-24 hours after the infusion began and at daily intervals thereafter (until therapeutic range was met and they were discharged back to the referring physician) | 1643 |
| **Time points reported** | Not reported | NA |
| **Outcome definition** *(with diagnostic criteria if relevant)* | Clotting time. The clotting time of normal pool plasma at this hospital was 40 seconds. | 1643 |
| **Unit of measurement** *(if relevant)* | Seconds | |
| **Scales: upper and lower limits** *(indicate whether high or low score is good)* | Target range between 60 and 90 seconds | 1643 |

| | | | |
|---|---|---|---|
| **Is outcome/tool validated?** | No | | NA |
| **Imputation of missing data** *(e.g. assumptions made for ITT analysis)* | No missing data | | NA |
| **Assumed risk estimate** *(e.g. baseline or population risk noted in Background)* | Not reported | | NA |
| **Power** *(e.g. power & sample size calculation, level of power achieved)* | To detect a difference of 3000 units/24 hours in the mean heparin dose at $p < 0.05$, each of the two groups had to have at least 32 patients. This difference was the minimal incremental change ordered for any patient. | | 4, Footnote of text from 2 |
| **Notes** | None | | |

### Number of days to achieve therapeutic proconvertin and prothrombin

| | Description as stated in report/paper | | Page number |
|---|---|---|---|
| **Outcome name** | Prothrombin time by the proconvertin and prothrombin | | 1643 |
| **Time points measured** *(specify whether from start or end of intervention)* | Initial blood sample obtained 3-24 hours after the infusion began and at daily intervals thereafter (until therapeutic range was met and they were discharged back to the referring physician) | | 1643 |
| **Time points reported** | Not reported | | NA |
| **Outcome definition** *(with diagnostic criteria if relevant)* | *Proconvertin and prothrombin are proteins involved in anticoagulation.* | | NA |
| **Unit of measurement** *(if relevant)* | Days | | |
| **Scales: upper and lower limits** *(indicate whether high or low score is good)* | 10-20% activity (normal range was 80-130% activity). | | 1643 |
| **Is outcome/tool validated?** | No | | No |
| **Imputation of missing data** *(e.g. assumptions made for ITT analysis)* | No missing data | | NA |
| **Assumed risk estimate** *(e.g. baseline or population risk noted in Background)* | Not reported | | NA |

| | | |
|---|---|---|
| **Power** *(e.g. power & sample size calculation, level of power achieved)* | Not reported | NA |
| **Notes** | None | |

## Funding/conflict of interest

| | | |
|---|---|---|
| **Study funding sources** *(including role of funders)* | Not reported | NA |
| **Possible conflicts of interest** *(for study authors)* | Not reported | NA |
| **Notes** | None | |

# Data and analysis

## Partial thromboplastin time (PTT)

| | **Description as stated in report/paper** | | | | | | **Page number** |
|---|---|---|---|---|---|---|---|
| **Outcome** | Partial thromboplastin time (PTT) | | | | | | 1643 |
| **Time point** *(specify from start or end of intervention)* | Baseline; daily until therapeutic range met | | | | | | 1643 |
| **Results** | Intervention | | | Comparison | | | 1644 (table 4) |
| | Mean | SD *(or other variance, specify)* | No. participants | Mean | SD *(or other variance, specify)* | No. participants | |
| | 82 | 14 | 42 | 84 | 17 | 39 | |
| **Any other results reported** *(e.g. mean difference, CI, P value)* | No significant difference | | | | | | 1644 |
| **No. missing participants** | NA | | | | | | NA |
| **Statistical methods used and appropriateness of these** *(e.g. adjustment for correlation)* | Mean values for the two groups were compared using the two-tailed Student's t test for impaired data. Chi square analysis with Yate's correction was used to analyze associations between discrete variables. Actual and simulated doses of the anticoagulants were compared using linear regression analysis. Calculations were made using the Statistical Analysis System.'' Results were deemed significant at values of $p < 0.05$. | | | | | | 1644 |

| | |
|---|---|
| **Notes** | **None** |

## No. days to achieve therapeutic proconvertin and prothrombin

| | Description as stated in report/paper | | | | | | Page number |
|---|---|---|---|---|---|---|---|
| **Outcome** | No. days to achieve therapeutic proconvertin and prothrombin | | | | | | 1643 |
| **Time point** *(specify from start or end of intervention)* | Baseline; daily until therapeutic range met | | | | | | 1643 |
| **Results** | Intervention | | | Comparison | | | 1644 (table 4) |
| | Mean | SD *(or other variance, specify)* | No. participants | Mean | SD *(or other variance, specify)* | No. participants | |
| | 5.7 | 1.4 | 42 | 5.8 | 2.1 | 39 | |
| **Any other results reported** *(e.g. mean difference, CI, P value)* | No significant difference | | | | | | 1644 |
| **No. missing participants** | NA | | | | | | NA |
| **Statistical methods used and appropriateness of these** *(e.g. adjustment for correlation)* | Mean values for the two groups were compared using the two-tailed Student's t test for impaired data. Chi square analysis with Yate's correction was used to analyze associations between discrete variables. Actual and simulated doses of the anticoagulants were compared using linear regression analysis. Calculations were made using the Statistical Analysis System.'' Results were deemed significant at values of p < 0.05. | | | | | | 1644 |
| **Notes** | | | | | | | |

## Conclusions

| | Description as stated in report/paper | Page number |
|---|---|---|
| **Key conclusions of study authors** | We have shown that certified pharmacist prescribers can adjust doses of anticoagulants for inpatients according to a protocol as safely as an experienced physician. These results provide support for other pharmacists seeking to expand their clinical practice or initiate legislation supporting current practice situations. | 1645 |
| **Notes** | None | |

## Cohen et al. 1985

## Study eligibility

| Study Characteristics | Eligibility criteria |
|---|---|
| Title | Evaluation of a pharmacist-managed anticoagulation clinic |
| Author (year) | Cohen et al. 1985 |
| Country | Michigan, USA |
| Type of study | Retrospective cohort |
| Participants | Male outpatients who, over a 2.5-year period, had been monitored for warfarin therapy for a minimum of 3 months |
| Types of intervention | Pharmacist-managed anticoagulation clinic |
| Types of comparison | Physician-managed anticoagulation clinic |
| Types of outcome measures | Prothrombin time; warfarin-related complications (bleeding and thromboembolic events) |
| Prescriptive authority | Protocol |
| Include/Exclude | Include |
| Notes | None |

**DO NOT PROCEED IF STUDY EXCLUDED FROM REVIEW**

## Characteristics of included studies

### Methods

| | Descriptions as stated in report/paper | | Page number |
|---|---|---|---|
| **Aim of study** | The objective of this study was to compare the management of patients on warfarin therapy by the ASC and by other VAMC clinics at the same institution. Specifically, the two groups were compared primarily on the basis of PT data and also on the incidence of warfarin-related complications | | 168 |
| **Design** | Retrospective cohort | | 169 |
| **Unit of allocation** *(by individuals, cluster/ groups or body parts)* | Individuals | | 169 |
| **Start-end date** | Not reported | | NA |
| **Duration of participation** *(from recruitment to last follow-up/ baseline to last follow-up- group level) 1. Time of consent until last measurement for each individual. 2. Baseline to final follow-up for each individual* | Over a 2.5-year period, had been monitored for warfarin therapy for a minimum of 3 months | | 168 |
| **Study duration** *(as above with the exception of interim analyses or other circumstances* | Information was recorded for the most recent 2-year period or for the entire course of therapy, whichever was less. | | 169 |
| **Ethical approval needed/obtained for study** | Not reported | Not reported | NA |
| **Notes** | None | | |

### Participants

| | **Description** *Include comparative information for each intervention or comparison group if available* | **Page number** |
|---|---|---|

| | | | |
|---|---|---|---|
| **Population description** *(from which study participants are drawn)* | Male outpatients who, over a 2.5-year period, had been monitored for warfarin therapy for a minimum of 3 months | | 168 |
| **Setting** *(including location and social context)* | VA outpatient medical centre | | 169 |
| **Inclusion/exclusion criteria** | Patients identified by a complete review of pharmacy prescription records were separated into two groups based upon where the patient was monitored for warfarin therapy. The 78 patients in Group 1 were followed by the ASC. The 17 patients in Group 2 were followed by other VAMC clinics. Patients who received warfarin from the VAMC pharmacy but who were monitored by their local physicians and patients transferred to other VA hospitals were not included in the study. | | 169 |
| **Method of recruitment of participants** | Pharmacy prescription record review | | 169 |
| **Informed consent obtained** | Not reported | NA | NA |
| **Total no. randomised** *(or total pop. at start of study for NRCTs)* | 95 individuals | | 169 |
| **Clusters** | NA | | NA |
| **Baseline imbalances** | The two groups were not significantly different regarding demographic characteristics (Table 1). The per cent of patients with pre-existing conditions that could complicate anticoagulant control were also found not to differ significantly between the groups (Table 2). Although not statistically significant, the mean diastolic blood pressures measured at the beginning of the evaluation period in those patients diagnosed as hypertensive was higher in the ASC patients (Group I: 93.9 _+ 9.4 mmHg, in 26 patients; Group 11: 87.7 7.8 mmHg in six patients, P=O.14). (All values are means+ SD).<br><br>No significant difference related to indications<br>for warfarin therapy existed between the two groups | | 169 |
| **Withdrawals and exclusions** | Not reported | | NA |
| **Age (years)** | Intervention: 55.8 ± 10.5<br>Comparator: 57.8 ± 9.4 | | 170 (table 1) |
| **Sex (female)** | Intervention: 0%<br>Comparator: 0% | | 170 (table 1) |
| **Subgroups measure** | Not reported | | NA |

| | | |
|---|---|---|
| **Subgroups reported** | Not reported | NA |
| **Notes** | None | |

## Intervention group

| | **Description as stated in report/paper** | **Page number** |
|---|---|---|
| **Group name** | Pharmacist-managed anticoagulation clinic | 168 |
| **No. randomised/assigned to group** *(specify whether no. people or clusters)* | 78 | 170 (table 1) |
| **Description** *(include sufficient detail for replication, e.g. content, dose, components)* | Patients, once referred to the ASC by VAMC physicians, are managed by clinic pharmacists according to a VAMC-approved protocol. In accordance with the protocol, ASC patients: are thoroughly educated by the clinic pharmacists about the safe and appropriate use of anticoagulants; are scheduled for periodic return visits to the ASC for assessment of their therapeutic status; have their progress documented in the charts and the clinic profiles; are screened by the pharmacists for drug-drug, drugfood, drug-disease interactions and patient compliance problems at each visit to the clinic; and have warfarin dosage adjustments performed by the clinic pharmacists as necessary to maintain a safe and effective level of anticoagulation | 168 |
| **Duration of treatment period** | Over a 2.5-year period, had been monitored for warfarin therapy for a minimum of 3 months | 168 |
| **Timing** | Not reported | NA |
| **Co-interventions** | Education, chart review, general counselling (for adherence, diet, and lifestyle problems) | NA |
| **Notes** | None | |

## Comparator group

| | **Description as stated in report/paper** | **Page number** |
|---|---|---|
| **Group name** | Physician-managed anticoagulation clinic | 168 |
| **No. randomised/assigned to group** *(specify whether no. people or clusters)* | 17 | 170 (table 1) |

| | | |
|---|---|---|
| **Description** *(include sufficient detail for replication, e.g. content, dose, components)* | Some VAMC physicians have chosen to manage personally the anticoagulant therapy of their warfarin-treated patients in other VA clinics. These patients are monitored, according to the preference of the individual's physicians and, unlike the ASC, no uniform protocol is followed | 168 |
| **Duration of treatment period** | Over a 2.5-year period, had been monitored for warfarin therapy for a minimum of 3 months | 168 |
| **Timing** | Not reported | NA |
| **Co-interventions** | Not reported | NA |
| **Notes** | None | |

## Outcomes

### Prothrombin time ratio

| | Description as stated in report/paper | | Page number |
|---|---|---|---|
| **Outcome name** | Prothrombin time ratio | | 169 |
| **Time points measured** *(specify whether from start or end of intervention)* | Information was recorded for the most recent 2-year period or for the entire course of therapy, whichever was less. | | 169 |
| **Time points reported** | Information was recorded for the most recent 2-year period or for the entire course of therapy, whichever was less. | | 169 |
| **Outcome definition** *(with diagnostic criteria if relevant)* | Prothrombin time control. The PTs for all patients were determined in the same VAMC laboratory using a photo-optical sensor method with fibrin clot formation as the endpoint. | | 169 |
| **Unit of measurement** *(if relevant)* | Event data | | 172 |
| **Scales: upper and lower limits** *(indicate whether high or low score is good)* | Therapeutic mean PT ratio: 1.5-2.5<br>Low mean PT radio: < 1.5<br>High mean PT ratio . 2.5 | | 172 |
| **Is outcome/tool validated?** | No | Bloodwork | NA |
| **Imputation of missing data** *(e.g. assumptions made for ITT analysis)* | Not reported | | NA |
| **Assumed risk estimate** *(e.g. baseline or population risk noted in Background)* | Not reported | | NA |

| | | |
|---|---|---|
| **Power** *(e.g. power & sample size calculation, level of power achieved)* | Not reported | NA |
| **Notes** | None | |

## Warfarin-related complications (bleeding)

| | **Description as stated in report/paper** | | **Page number** |
|---|---|---|---|
| **Outcome name** | Bleeding | | 169 |
| **Time points measured** *(specify whether from start or end of intervention)* | Information was recorded for the most recent 2-year period or for the entire course of therapy, whichever was less. | | 169 |
| **Time points reported** | Information was recorded for the most recent 2-year period or for the entire course of therapy, whichever was less. | | 169 |
| **Outcome definition** *(with diagnostic criteria if relevant)* | Warfarin-related complications including bleeding | | 169 |
| **Unit of measurement** *(if relevant)* | Event data | | 172 |
| **Scales: upper and lower limits** *(indicate whether high or low score is good)* | Lower scores are desired | | NA |
| **Is outcome/tool validated?** | No | Bloodwork | NA |
| **Imputation of missing data** *(e.g. assumptions made for ITT analysis)* | Not reported | | NA |
| **Assumed risk estimate** *(e.g. baseline or population risk noted in Background)* | Not reported | | NA |
| **Power** *(e.g. power & sample size calculation, level of power achieved)* | Not reported | | NA |
| **Notes** | None | | |

## Warfarin-related complications (thromboembolic events)

| | **Description as stated in report/paper** | **Page number** |
|---|---|---|
| **Outcome name** | Thromboembolic event | 169 |
| **Time points measured** *(specify whether from start or end of intervention)* | Information was recorded for the most recent 2-year period or for the entire course of therapy, whichever was less. | 169 |

| | | | |
|---|---|---|---|
| **Time points reported** | Information was recorded for the most recent 2-year period or for the entire course of therapy, whichever was less. | | 169 |
| **Outcome definition** *(with diagnostic criteria if relevant)* | Warfarin-related complications including thromboembolic events | | 169 |
| **Unit of measurement** *(if relevant)* | Event data | | 172 |
| **Scales: upper and lower limits** *(indicate whether high or low score is good)* | Lower scores are desired | | NA |
| **Is outcome/tool validated?** | No | Bloodwork | NA |
| **Imputation of missing data** *(e.g. assumptions made for ITT analysis)* | Not reported | | NA |
| **Assumed risk estimate** *(e.g. baseline or population risk noted in Background)* | Not reported | | NA |
| **Power** *(e.g. power & sample size calculation, level of power achieved)* | Not reported | | NA |
| **Notes** | None | | |

## Funding/conflict of interest

| | | |
|---|---|---|
| **Study funding sources** *(including role of funders)* | Not reported | NA |
| **Possible conflicts of interest** *(for study authors)* | Not reported | NA |
| **Notes** | None | |

## Data and analysis

### Prothrombin time ratio

| | **Description as stated in report/paper** | | | | **Page number** |
|---|---|---|---|---|---|
| **Outcome** | Prothrombin time ratio | | | | 169 |
| **Time point** *(specify from start or end of intervention)* | Not reported | | | | NA |
| **Results** | Intervention | | Comparison | | 172 (table 4) |
| | No. with event | Total in group | No. with event | Total in group | |

| | | | | | |
|---|---|---|---|---|---|
| | 68 | 78 | 14 | 17 | |
| **Any other results reported** | Differences were not significant. | | | | 172 (table 4) |
| | Distribution of all PT ratios by category of PT control<br><br>737 events in intervention (n=78); 78 events in control (n=17); Differences were not significant. | | | | 172 (table 5) |
| **No. missing participants** | 0 | | NA | | NA |
| **Reasons missing** | NA | | NA | | NA |
| **Statistical methods used and appropriateness of these** | Data were analysed by two-tailed t-tests and Fisher's exact tests, as appropriate, with p<0.05 required for statistical significance. The exact test was used instead of %-square analysis to avoid the problems created by small expected frequencies in the contingency tables. | | | | 169 |
| **Confounders** | Not reported | | | | NA |
| **Notes** | None | | | | |

## Warfarin-related complications (bleeding)

| | **Description as stated in report/paper** | | | | **Page number** |
|---|---|---|---|---|---|
| **Outcome** | Warfarin-related complications (bleeding) | | | | 169 |
| **Time point** *(specify from start or end of intervention)* | Not reported | | | | NA |
| **Results** | Intervention | | Comparison | | 172 (table 4) |
| | No. with event | Total in group | No. with event | Total in group | |
| | 4 | 78 | 1 | 17 | |
| **Any other results reported** | Differences were not significant. | | | | 173 (table 6) |
| **No. missing participants** | 0 | | NA | | NA |
| **Reasons missing** | NA | | NA | | NA |
| **Statistical methods used and appropriateness of these** | Comparison by Fisher's exact test, differences were not significant. | | | | 173 (table 6) |
| **Confounders** | Not reported | | | | NA |
| **Notes** | None | | | | |

## Warfarin-related complications (thromboembolic events)

| | **Description as stated in report/paper** | **Page number** |
|---|---|---|
| **Outcome** | Warfarin-related complications (thromboembolic events) | 169 |
| **Time point** *(specify from start or end of intervention)* | Not reported | NA |

| Results | Intervention | | Comparison | | |
|---|---|---|---|---|---|
| | No. with event | Total in group | No. with event | Total in group | 172 (table 4) |
| | 2 | 78 | 0 | 17 | |
| Any other results reported | Differences were not significant. | | | | 173 (table 6) |
| No. missing participants | 0 | | NA | | NA |
| Reasons missing | NA | | NA | | NA |
| Statistical methods used and appropriateness of these | Comparison by Fisher's exact test, differences were not significant. | | | | 173 (table 6) |
| Confounders | Not reported | | | | NA |
| Notes | None | | | | |

## Conclusions

| | Description as stated in report/paper | Page number |
|---|---|---|
| Key conclusions of study authors | These results of PT and complication rates therefore strongly suggest that the ASC was at least as successful as the other VAMC clinics in monitoring warfarin therapy. | 173/174 |
| Notes | None | |

# Cowart et al. 2020

## Study eligibility

| Study Characteristics | Eligibility criteria |
|---|---|
| Title | Using an advanced practice pharmacist in a team-based care model to decrease time to hemoglobin A1C goal among patients with type 2 diabetes, florida, 2017–2019 |
| Author (year) | Cowart et al. 2020 |
| Country | Florida, USA |
| Type of study | Retrospective cohort |
| Participants | Patients with Type 2 diabetes |
| Types of intervention | Pharmacist-physician management |
| Types of comparison | Usual care |

| | |
|---|---|
| **Types of outcome measures** | Time to achieve an HbA1c of less than 7%; goal achieved |
| **Prescriptive authority** | Collaborative practice agreement |
| **Include/Exclude** | Include |
| **Notes** | None |

**DO NOT PROCEED IF STUDY EXCLUDED FROM REVIEW**

## Characteristics of included studies

### Methods

| | Descriptions as stated in report/paper | | Page number |
|---|---|---|---|
| **Aim of study** | The aim of our study was to analyze the time to achieve an HbA1c of less than 7% for a pharmacist–physician managed (PPM) cohort, as compared with a usual medical care (UMC) cohort of patients with type 2 diabetes. | | 1 |
| **Design** | Retrospective cohort | | 1 |
| **Unit of allocation** *(by individuals, cluster/ groups or body parts)* | Individuals | | 2 |
| **Start-end date** | January 2017 – July 2019 | | 1 |
| **Duration of participation** *(from recruitment to last follow-up/ baseline to last follow-up- group level)* *1. Time of consent until last measurement for each individual.* *2. Baseline to final follow-up for each individual* | No set treatment period. Treatment continued until therapeutic range was reached for each individual. Median time to achieve an HbA1c of less than 7% in the PPM cohort was 470 days, as compared with 569 days in the UMC cohort (median difference of 99 days, P = .60) | | 2 |
| **Study duration** *(as above with the exception of interim analyses or other circumstances* | As above | | NA |
| **Ethical approval needed/obtained for study** | Yes | Our study was certified exempt by the USF Institutional Review Board. | 2 |
| **Notes** | None | | |

### Participants

| | **Description** *Include comparative information for each intervention or comparison group if available* | **Page number** |
|---|---|---|

| | | | |
|---|---|---|---|
| **Population description** *(from which study participants are drawn)* | Patients with type 2 diabetes | | 1 |
| **Setting** *(including location and social context)* | Family medicine clinic | | 1 |
| **Inclusion/exclusion criteria** | Inclusion criteria were adults, aged 18 to 80 years, having type 2 diabetes for least 12 months, and an HbA1c at 7% or higher at the index visit (the first visit during the study) | | 1 |
| **Method of recruitment of participants** | Electronic health records | | 2 |
| **Informed consent obtained** | Not applicable (exempt) | NA | NA |
| **Total no. randomised** *(or total pop. at start of study for NRCTs)* | 257 individuals | | 2 |
| **Clusters** | NA | | NA |
| **Baseline imbalances** | Groups did not differ substantially at baseline, except for HbA1c, which was significantly higher in the PPM cohort as compared with the UMC cohort (P < 0.001). | | 2 |
| **Withdrawals and exclusions** | None reported | | NA |
| **Age (SD)** | Intervention: 59.75 years (11.56)<br>Comparator: 57.87 years (12.57) | | 5 (table 1) |
| **Sex (female)** | Not reported | | NA |
| **Subgroups measure** | Adherence, baseline HBA1C | | 6 (table 2) |
| **Subgroups reported** | Adherence, baseline HBA1C | | 6 (table 2) |
| **Notes** | None | | |

## Intervention group

| | Description as stated in report/paper | Page number |
|---|---|---|
| **Group name** | Physician-pharmacist management | 1 |
| **No. randomised/assigned to group** *(specify whether no. people or clusters)* | 76 individuals | 2 |

| | | |
|---|---|---|
| **Description** *(include sufficient detail for replication, e.g. content, dose, components)* | A collaborative drug therapy management agreement gave APPs the authority to initiate, titrate, or discontinue antidiabetic medications; order drug therapy–related laboratory tests; and provide diabetes self-management education. APPs practiced in the same clinic as the PCP and independently saw patients face-to-face following referrals from the PCP. Visits with the APP were scheduled for 30 to 60 minutes, whereas visits with patients in the UMC cohort were 20 to 40 minutes. Visits in the UMC cohort were either routine follow-up visits or sick visits. | 2 |
| **Duration of treatment period** | No set treatment period. Treatment continued until therapeutic range was reached for each individual. Median time to achieve an HbA1c of less than 7% in the PPM cohort was 470 days, as compared with 569 days in the UMC cohort (median difference of 99 days, P =0.60) | 2 |
| **Timing** | As above | NA |
| **Co-interventions** | Not reported | NA |
| **Notes** | None | |

## Comparator group

| | Description as stated in report/paper | Page number |
|---|---|---|
| **Group name** | Usual care (primary care provider) | 2 |
| **No. randomised/assigned to group** *(specify whether no. people or clusters)* | 181 individuals | 2 |
| **Description** *(include sufficient detail for replication, e.g. content, dose, components)* | Patients were assigned to the UMC cohort if they were managed solely by their PCPs and did not have a clinic visit with an APP during the study. | 2 |
| **Duration of treatment period** | No set treatment period. Treatment continued until therapeutic range was reached for each individual. Median time to achieve an HbA1c of less than 7% in the PPM cohort was 470 days, as compared with 569 days in the UMC cohort (median difference of 99 days, P =0.60) | 2 |
| **Timing** | As above | NA |
| **Co-interventions** | Not reported | NA |
| **Notes** | None | |

# Outcomes

## HbA1c goal achieved

| | Description as stated in report/paper | | Page number |
|---|---|---|---|
| **Outcome name** | HbA1c goal achieved | | 2 |
| **Time points measured** *(specify whether from start or end of intervention)* | Baseline; follow-up time was calculated as time in days from first visit to achieving the HbA1c goal or last clinic visit | | 2 |
| **Time points reported** | Baseline; follow-up time was calculated as time in days from first visit to achieving the HbA1c goal or last clinic visit | | 2 |
| **Outcome definition** *(with diagnostic criteria if relevant)* | HbA1C goals achieved | | 2 |
| **Unit of measurement** *(if relevant)* | Event data | | 5 (table 1) |
| **Scales: upper and lower limits** *(indicate whether high or low score is good)* | Higher scores are desired | | NA |
| **Is outcome/tool validated?** | Yes | Bloodwork | NA |
| **Imputation of missing data** *(e.g. assumptions made for ITT analysis)* | NA | | NA |
| **Assumed risk estimate** *(e.g. baseline or population risk noted in Background)* | Not reported | | NA |
| **Power** *(e.g. power & sample size calculation, level of power achieved)* | Not reported | | NA |
| **Notes** | None | | |

## Time to achieve HbA1c goal

| | Description as stated in report/paper | Page number |
|---|---|---|
| **Outcome name** | Time to achieve HbA1c goal | 2 |
| **Time points measured** *(specify whether from start or end of intervention)* | Baseline; follow-up time was calculated as time in days from first visit to achieving the HbA1c goal or last clinic visit | 2 |
| **Time points reported** | Baseline; follow-up time was calculated as time in days from first visit to achieving the HbA1c goal or last clinic visit | 2 |
| **Outcome definition** *(with diagnostic criteria if relevant)* | Time to achieve HbA1c goal | 2 |

| | | | |
|---|---|---|---|
| **Unit of measurement** *(if relevant)* | Continuous (days) | | 5 (table 1) |
| **Scales: upper and lower limits** *(indicate whether high or low score is good)* | Lower scores are desired | | NA |
| **Is outcome/tool validated?** | Yes | Bloodwork | NA |
| **Imputation of missing data** *(e.g. assumptions made for ITT analysis)* | NA | | NA |
| **Assumed risk estimate** *(e.g. baseline or population risk noted in Background)* | Not reported | | NA |
| **Power** *(e.g. power & sample size calculation, level of power achieved)* | Not reported | | NA |
| **Notes** | None | | |

## Funding/conflict of interest

| | | |
|---|---|---|
| **Study funding sources** *(including role of funders)* | This study was funded by an internal seed grant from the University of South Florida, Taneja College of Pharmacy, Department of Pharmacotherapeutics and Clinical Research | 3 |
| **Possible conflicts of interest** *(for study authors)* | No further disclosures made | NA |
| **Notes** | None | |

## Data and analysis

### HbA1C goal achieved

| | **Description as stated in report/paper** | | | | **Page number** |
|---|---|---|---|---|---|
| **Outcome** | HbA1c goal achieved | | | | 2 |
| **Time point** *(specify from start or end of intervention)* | Follow-up time was calculated as time in days from first visit to achieving the HbA1c goal or last clinic visit | | | | 2 |
| **Results** | Intervention | | Comparison | | 5 (table 2) |
| | No. with event | Total in group | No. with event | Total in group | |
| | 38 | 76 | 78 | 181 | |

| | | | |
|---|---|---|---|
| **Any other results reported** | In the PPM cohort, 50% of patients met an HbA1c goal of less than 7%, as compared with 43.1% in the UMC cohort (P = .31). When stratified by adherence to clinic visits, 33.3% of patients in the PPM cohort with low adherence to clinic visits met an HbA1c goal of less than 7%, as compared with 38.7% of patients with low adherence to clinic visits in the UMC cohort. A higher percentage of patients in the PPM cohort with moderate adherence (63.3%) and high adherence (50.0%) to clinic visits met an HbA1c goal of less than 7% compared with patients in the UMC cohort with moderate and high adherence to clinic visits (46.6% and 44.6%, respectively). | | 2 |
| **No. missing participants** | NA | NA | NA |
| **Reasons missing** | NA | NA | NA |
| **Statistical methods used and appropriateness of these** | Chi-square tests and 2 independent sample t tests were used to determine whether a statistically significant difference existed between groups for categorical and continuous variables, respectively, at baseline. Follow-up time was calculated as time in days from first visit to achieving the HbA1c goal or last clinic visit. Statistical significance was defined as P < .05. The analysis was conducted using SAS version 9.4 (SAS Institute, Inc). | | 2 |
| **Confounders** | Not reported | | NA |
| **Notes** | None | | |

## Time to achieve HbA1C goal

| | Description as stated in report/paper | | | | | | Page number |
|---|---|---|---|---|---|---|---|
| **Outcome** | Time to achieve HbA1c goal | | | | | | 2 |
| **Time point** *(specify from start or end of intervention)* | Follow-up time was calculated as time in days from first visit to achieving the HbA1c goal or last clinic visit | | | | | | 2 |
| **Results** | Intervention | | | Comparison | | | 5 (table 2) |
| | Median | SD *(or other variance, specify)* | No. participants | Median | SD *(or other variance, specify)* | No. participants | |
| | 470 | NR | 76 | 569 | NR | 181 | |

| | | | |
|---|---|---|---|
| **Any other results reported** *(e.g. mean difference, CI, P value)* | Median time to achieve an HbA1c of less than 7% in the PPM cohort was 470 days, as compared with 569 days in the UMC cohort (median difference of 99 days, P = .60) (Table 2). However, when results were stratified by baseline HbA1c, the median time to achieve an HbA1c of less than 7% was 512 and 668 days for the PPM and UMC cohorts, respectively (P = .11). Similarly, when results were stratified by adherence to clinic visits, the median time to achieve an HbA1c of less than 7% in the PPM cohort was 441 days based on moderate adherence to clinic visits, and 381 days based on high adherence to clinic visits (Table 2). Among those included in the PPM cohort with low adherence to clinic visits, time to achieve an HbA1c less than 7% was not estimable. That is, 50% of patients in the PPM cohort with low adherence to clinic visits did not achieve an HbA1c less than 7%, based on the time specified in this analysis. Among patients in the UMC cohort, time to achieve an HbA1c less than 7% was 612 days for those with low adherence, 457 days for moderate adherence, and 569 days for high adherence. However, these differences were not statistically significant (P = .80). | | 2 |
| **No. missing participants** | NA | NA | NA |
| **Statistical methods used and appropriateness of these** *(e.g. adjustment for correlation)* | Chi-square tests and 2 independent sample t tests were used to determine whether a statistically significant difference existed between groups for categorical and continuous variables, respectively, at baseline. Follow-up time was calculated as time in days from first visit to achieving the HbA1c goal or last clinic visit. Statistical significance was defined as P < .05. The analysis was conducted using SAS version 9.4 (SAS Institute, Inc).NA | | 2 |
| **Confounders** | NA | | NA |
| **Notes** | None | | |

## Conclusions

| | Description as stated in report/paper | Page number |
|---|---|---|
| **Key conclusions of study authors** | Patients exposed to an APP in our study (PPM) experienced a shorter median time to achieve an HbA1c of less than 7% than did those receiving usual care (UMC), although results were not statistically significant. | 3-4 |
| **Notes** | None | |

# Cowart et al. 2022

## Study eligibility

| Study Characteristics | Eligibility criteria |
|---|---|

| | |
|---|---|
| **Title** | Measurement of Pharmacist-Physician Collaborative Care on Therapeutic Inertia in Patients With Type 2 Diabetes |
| **Author (year)** | Cowart et al. 2022 |
| **Country** | Florida, US |
| **Type of study** | Retrospective cohort study |
| **Participants** | Patient with type 2 diabetes |
| **Types of intervention** | Pharmacist-physician practice models |
| **Types of comparison** | Usual medical care |
| **Types of outcome measures** | Time to treatment intensification, HgA1C goal achieved |
| **Prescriptive authority** | Collaborative practice agreement |
| **Include/Exclude** | Include |
| **Notes** | Collaborative drug therapy management agreement allowed APPs to initiate, titrate, or discontinue antidiabetic medications, order antidiabetic therapy–related laboratory tests, and provide diabetes-related counselling. |

**DO NOT PROCEED IF STUDY EXCLUDED FROM REVIEW**

## Characteristics of included studies

### Methods

| | Descriptions as stated in report/paper | | Page number |
|---|---|---|---|
| **Aim of study** | to (1) evaluate time to TI in a PPM as compared with UMC and (2) to explore characteristics (method and type) of antidiabetic TI in the PPM and UMC cohorts. | | 156 |
| **Design** | Retrospective cohort study | | 156 |
| **Unit of allocation** *(by individuals, cluster/ groups or body parts)* | Individuals | | 156 |
| **Start-end date** | January 2017-December 2018 | | 156 |
| **Duration of participation** *(from recruitment to last follow-up)* | At least six months follow-up data available<br><br>Minimum requirements for intervention group: One visit with primary care provider, one visit with advanced pharmacist practitioner<br><br>Minimum requirements for control group: One visit with primary care provider | | 156 |
| **Ethical approval needed/obtained for study** | Yes | This study was certified exempt by the USF Institutional Review Board. | 156 |
| **Notes** | None | | |

### Participants

| | | |
|---|---|---|
| | **Description**<br>*Include comparative information for each intervention or comparison group if available* | **Page number** |
| **Population description**<br>*(from which study participants are drawn)* | Patients were included if they were adults (≥18 years of age), had T2D for at least 12 months, and had an A1C >7% between January 1, 2017, and June 30, 2018 | 156 |
| **Setting**<br>*(including location and social context)* | Primary care | 156 |

| | | |
|---|---|---|
| **Inclusion/exclusion criteria** | Patients were included if they were adults (≥18 years of age), had T2D for at least 12 months, and had an A1C >7% between January 1, 2017, and June 30, 2018, which we termed the *identification period*. We defined the index date as the first visit with a recorded A1C >7% during the identification period. Patients were excluded if they had <6 months of follow-up data available, had their antidiabetic treatment intensified on the index date (to ensure that the cohort did not experience the outcome at baseline), or had missing data on baseline antidiabetic medication. | 156 |
| **Method of recruitment of participants** | Patients were assigned to the PPM cohort if they had at least 1 visit with their primary care physician (PCP) and at least 1 visit with an advanced practice pharmacist (APP). Those assigned to the UMC cohort were managed solely by their PCP. | 156 |
| **Informed consent obtained** | Not applicable (exempted status) | NA |
| **Total no. randomised** *(or total pop. at start of study for NRCTs)* | 56 individuals | 157 (table 1) |
| **Clusters** | NA | NA |
| **Baseline imbalances** | Patients in both cohorts were matched (1:1) on the logit of the propensity scores using a caliper width of 0.94 SD of the propensity score | 156 |
| **Withdrawals and exclusions** | NA | NA |
| **Age (years)** | Intervention: 55.8 ± 10.5<br>Comparator: 57.8 ± 9.4 | 157 (table 1) |
| **Sex (female)** | Not reported | 157 (table 1) |
| **Subgroups measure** | As a secondary objective, we compared time to A1C goal between study groups in all patients and within the subgroup that experienced TI, using the Kaplan-Meier method. | 156 |
| **Subgroups reported** | Patients who received TI | 159 |
| **Notes** | None | |

## Intervention group

| | Description as stated in report/paper | Page number |
|---|---|---|
| **Group name** | Pharmacist and physician prescribing | 156 |
| **No. randomised/assigned to group** *(specify whether no. people or clusters)* | N=28 | 157 (table 1) |

| | | |
|---|---|---|
| **Description** *(include sufficient detail for replication, e.g. content, dose, components)* | Patients were assigned to the PPM cohort if they had at least 1 visit with their primary care physician (PCP) and at least 1 visit with an advanced practice pharmacist (APP). Those assigned to the UMC cohort were managed solely by their PCP. A collaborative drug therapy management agreement allowed APPs to initiate, titrate, or discontinue antidiabetic medications, order antidiabetic therapy–related laboratory tests, and provide diabetes-related counselling. | 156 |
| **Duration of treatment period** | A minimum of one visit with the primary care provider and one visit with the advanced practice pharmacist. Visits were scheduled for 30 to 60 minutes. 8 visits (and 12 with PCP), with no missed appointments, was 100% adherent. | 156 |
| **Timing** | Not reported | NA |
| **Co-interventions** | 12 visits with primary care provider (usual medical care) | 156 |
| **Notes** | None | |

## Comparator group

| | Description as stated in report/paper | Page number |
|---|---|---|
| **Group name** | Physician prescribing | 156 |
| **No. randomised/assigned to group** *(specify whether no. people or clusters)* | N=28 | 157 (table 1) |
| **Description** *(include sufficient detail for replication, e.g. content, dose, components)* | Visits in the UMC cohort were routine follow-up visits or sick visits. | 156 |
| **Duration of treatment period** | 12 visits with primary care provider. Visits were 20 to 40 minutes. | 156 |
| **Timing** | Not reported | NA |
| **Co-interventions** | None | 156 |
| **Notes** | None | |

## Outcomes

### Time to treatment intensification

| | Description as stated in report/paper | Page number |
|---|---|---|
| **Outcome name** | Time to treatment intensification | 158 (table 2) |

| | | | |
|---|---|---|---|
| **Time points measured** *(specify whether from start or end of intervention)* | To calculate the time to antidiabetic TI, the index date was subtracted from the first date of antidiabetic TI occurring in the medical record. If treatment was not intensified, then the date of the last clinic visit during the study time period was used. | | 156 |
| **Time points reported** | As above, varied but minimum 6 months of follow-up data available. | | 156 |
| **Outcome definition** *(with diagnostic criteria if relevant)* | As above | | 156 |
| **Unit of measurement** *(if relevant)* | Days | | 158 (table 2) |
| **Scales: upper and lower limits** *(indicate whether high or low score is good)* | Fewer days is desirable | | 158 (table 2) |
| **Is outcome/tool validated?** | No | | NA |
| **Imputation of missing data** *(e.g. assumptions made for ITT analysis)* | NA | | NA |
| **Assumed risk estimate** *(e.g. baseline or population risk noted in Background)* | Not reported | | NA |
| **Power** *(e.g. power & sample size calculation, level of power achieved)* | Not reported | | NA |
| **Notes** | None | | |

## HgA1C level goal achieved

| | Description as stated in report/paper | Page number |
|---|---|---|
| **Outcome name** | HgA1C level goal achieved | 159 |
| **Time points measured** *(specify whether from start or end of intervention)* | Baseline, 1 year post index | 159 |
| **Time points reported** | As above | 159 |
| **Outcome definition** *(with diagnostic criteria if relevant)* | As above | 159 |
| **Unit of measurement** *(if relevant)* | % | 159 |

| | | | |
|---|---|---|---|
| **Scales: upper and lower limits** *(indicate whether high or low score is good)* | Lower than 7% is desirable | | 159 |
| **Is outcome/tool validated?** | No | | NA |
| **Imputation of missing data** *(e.g. assumptions made for ITT analysis)* | NA | | NA |
| **Assumed risk estimate** *(e.g. baseline or population risk noted in Background)* | Not reported | | NA |
| **Power** *(e.g. power & sample size calculation, level of power achieved)* | Not reported | | NA |
| **Notes** | None | | |

## Funding/conflict of interest

| | | |
|---|---|---|
| **Study funding sources** *(including role of funders)* | The authors disclosed receipt of the following financial support for the research, authorship, and/or publication of this article: This work was supported by a grant from the USF Health Taneja College of Pharmacy, Department of Pharmacotherapeutics and Clinical Research. The funding source had no role in the preparation or writing of this article. | 160 |
| **Possible conflicts of interest** *(for study authors)* | The authors declared no potential conflicts of interest with respect to the research, authorship, and/or publication of this article. | 160 |
| **Notes** | None | |

## Data and analysis

### Time to treatment intensification

| | **Description as stated in report/paper** | | **Page number** |
|---|---|---|---|
| **Outcome** | Time to treatment intensification | | 158 (table 2) |
| **Time point** *(specify from start or end of intervention)* | To calculate the time to antidiabetic TI, the index date was subtracted from the first date of antidiabetic TI occurring in the medical record. If treatment was not intensified, then the date of the last clinic visit during the study time period was used. | | 156 |
| **Results** | Intervention | Comparison | 158 (table 2) |

| | | | | | | | |
|---|---|---|---|---|---|---|---|
| | Median | IQR *(or other variance, specify)* | No. participants | Median | IQR *(or other variance, specify)* | No. participants | |
| | 37.5 | 8, 216.5 | 28 | 142 | 16, 465 | 28 | |
| **Any other results reported** *(e.g. mean difference, CI, P value)* | p=0.19 | | | | | | 159 |
| **No. missing participants** | None | | | | | | NA |
| **Statistical methods used and appropriateness of these** *(e.g. adjustment for correlation)* | We assessed covariate balance between study groups using a standardized mean difference threshold of 0.25. The distribution of baseline characteristics in the matched cohort was summarized using counts and percentages for categorical variables, whereas means and SDs or medians and interquartile ranges were used for continuous variables. Pearson χ2 tests and paired *t*-tests were used to determine whether a statistically significant difference existed between the matched cohorts for categorical and continuous variables, respectively, at baseline. Statistical significance was defined as a *P* <0.05.<br><br>To calculate the time to antidiabetic TI, the index date was subtracted from the first date of antidiabetic TI occurring in the medical record. If treatment was not intensified, then the date of the last clinic visit during the study time period was used. Median time (in days) to TI was calculated by using the Kaplan-Meier estimate among each cohort and compared using a log rank test. As a secondary objective, we compared time to A1C goal between study groups in all patients and within the subgroup that experienced TI, using the Kaplan-Meier method. | | | | | | 156 |
| **Notes** | **None** | | | | | | |

## HgA1C level goal achieved

| | Description as stated in report/paper | | | | Page number |
|---|---|---|---|---|---|
| **Outcome** | HgA1C level goal achieved | | | | 159 |
| **Time point** *(specify from start or end of intervention)* | 1 year post index | | | | 159 |
| **Results** | Intervention | | Comparison | | 158 |
| | No. with event | Total in group | No. with event | Total in group | |
| | 7 | 28 | 5 | 28 | |

| | | | |
|---|---|---|---|
| **Any other results reported** | p=0.66 | | 158 |
| **No. missing participants** | None | | NA |
| **Reasons missing** | NA | | NA |
| **Statistical methods used and appropriateness of these** | We assessed covariate balance between study groups using a standardized mean difference threshold of 0.25. The distribution of baseline characteristics in the matched cohort was summarized using counts and percentages for categorical variables, whereas means and SDs or medians and interquartile ranges were used for continuous variables. Pearson χ2 tests and paired *t*-tests were used to determine whether a statistically significant difference existed between the matched cohorts for categorical and continuous variables, respectively, at baseline. Statistical significance was defined as a *P* <0.05. | | 156 |
| **Notes** | Subgroup analysis: This effect was maintained in the subgroup (n = 49) of patients who received TI (PPM, 23.1%, vs UMC, 17.8%). 159 | | |

## Conclusions

| | **Description as stated in report/paper** | **Page number** |
|---|---|---|
| **Key conclusions of study authors** | In conclusion, a shorter time to TI and improvement in A1C goal achievement was observed with pharmacist-physician collaborative care as compared with UM | 160 |
| **Notes** | None | |

# Damaske et al. 2005

## Study eligibility

| **Study Characteristics** | **Eligibility criteria** |
|---|---|
| **Title** | Development and Implementation of a Pharmacist-Managed Inpatient Warfarin Protocol |
| **Author (year)** | Damaske et al. 2005 |
| **Country** | Texas, USA |
| **Type of study** | Controlled trial |
| **Participants** | Inpatients prescribed warfarin (indication included atrial fibrillation, cerebrovascular accident, pulmonary embolism, deep vein thrombosis/ venous thromboembolism) |
| **Types of intervention** | Pharmacist managed warfarin protocol |
| **Types of comparison** | Usual care (physician-directed warfarin) |

| | |
|---|---|
| **Types of outcome measures** | Average time to therapeutic INR; patients who had supratherapeutic INR; patients experiencing bleeds/adverse drug events; patients who received the correct first dose of 5 mg |
| **Prescriptive authority** | Protocol |
| **Include/Exclude** | Include |
| **Notes** | |

**DO NOT PROCEED IF STUDY EXCLUDED FROM REVIEW**

# Characteristics of included studies

## Methods

| | Descriptions as stated in report/paper | | Page number |
|---|---|---|---|
| **Aim of study** | A pilot study was conducted comparing results from the protocol with results from usual, physician-directed warfarin therapy. | | 397 |
| **Design** | Pilot control trial | | HRB assigned |
| **Unit of allocation** *(by individuals, cluster/ groups or body parts)* | Individual | | 398 |
| **Start-end date** | August 2004- November 2004 | | 398 |
| **Duration of participation** *(from recruitment to last follow-up)* | INR results on day 2 of therapy and every day until a therapeutic value of 2 to 3 was present for 2 consecutive days or until the patient was discharged. | | 398 |
| **Study duration** | INR results on day 2 of therapy and every day until a therapeutic value of 2 to 3 was present for 2 consecutive days or until the patient was discharged. | | 398 |
| **Ethical approval needed/obtained for study** | Yes | Institutional review board approval for the pilot study was obtained. | 397 |
| **Notes** | None | | |

## Participants

| | **Description** *Include comparative information for each intervention or comparison group if available* | Page number |
|---|---|---|
| **Population description** *(from which study participants are drawn)* | Inpatients prescribed warfarin (indication included atrial fibrillation, cerebrovascular accident, pulmonary embolism, deep vein thrombosis/venous thromboembolism) | 398 (figure 2) |
| **Setting** *(including location and social context)* | Hospital | 397 |

| | | |
|---|---|---|
| **Inclusion/exclusion criteria** | All patients in the identified service lines with an indication for warfarin were eligible to participate in the study unless one of the following exclusion criteria was met: a prosthetic heart valve; target INR >3.0; active bleeding; hematocrit <25%; elevated baseline INR (>1.3) without being on warfarin therapy prior to admission; epidural catheter; ventriculostomy; or lumbar puncture within 24 hours. Both patients newly started on warfarin and patients restarted on the drug after it had been withdrawn for procedures or other medical reasons were eligible for the study. | 398 |
| **Method of recruitment of participants** | All patients in the identified service lines with an indication for warfarin were eligible to participate [consecutive] | 398 |
| **Informed consent obtained** | Not reported | NA |
| **Total no. randomised** *(or total pop. at start of study for NRCTs)* | 51 | 398 |
| **Clusters** | NA | NA |
| **Baseline imbalances** | Not reported | NA |
| **Withdrawals and exclusions** | Not reported | NA |
| **Age** | Not reported | NA |
| **Sex (female)** | Not reported | NA |
| **Subgroups measure** | Not reported | NA |
| **Subgroups reported** | Not reported | NA |
| **Notes** | None | |

## Intervention group

| | Description as stated in report/paper | Page number |
|---|---|---|
| **Group name** | Pharmacist managed protocol group | 398 |
| **No. randomised/assigned to group** *(specify whether no. people or clusters)* | 22 | 398 |
| **Description** *(include sufficient detail for replication, e.g. content, dose, components)* | Pharmacists followed a warfarin dosing protocol for 6 days. Dosage changes continued on day 7 of warfarin therapy until discharge by adjusting the dose 10% to 20% if the patient was still not within the target therapeutic range. | 398 |
| **Duration of treatment period** | 6 days or until therapeutic range was reached | 398 |
| **Timing** | An initial warfarin dose of 5 mg was then given at 6:00 PM on day 1 of the protocol. Results were then obtained daily. | 398 |

| | | |
|---|---|---|
| **Co-interventions** | Ordering lab tests, provided education to all patients before discharge | 398, 399 |

| | | | |
|---|---|---|---|
| **Notes** | Dosage adjustments vary per protocol day and INR. If a medication is initiated that is known to interact with warfarin, the pharmacist will reduce doses or increase doses of warfarin according to clinical judgment. | | |
| | Day of warfarin therapy | INR | Dose (mg) |
| | 2 | <1.5 | <1.5 |
| | | 1.5–1.9 | 1.5–1.9 |
| | | 2.0–2.5 | 2.0–2.5 |
| | | >2.5 | >2.5 |
| | 3 | <1.5 | 5–10 |
| | | 1.5–1.9 | 2.5–5 |
| | | 2.0–2.5 | 0–2.5 |
| | | 2.6–3.0 | 0–2 |
| | | >3.0 | No dose |
| | 4 | <1.5 | 10 |
| | | 1.5–1.9 | 5–7.5 |
| | | 2.0–3.0 | 0–5 |
| | | >3.0 | No dose |
| | 5 | <1.5 | 10 |
| | | 1.5–1.9 | 7.5–10 |
| | | 2.0–3.0 | 0–5 |
| | | >3.0 | No dose |
| | 6 | <1.5 | 7.5–12.5 |
| | | 1.5–1.9 | 5–10 |
| | | 2.0–3.0 | 0–5 |
| | | >3.0 | No dose |

## Comparator group

| | **Description as stated in report/paper** | **Page number** |
|---|---|---|
| **Group name** | Physician managed | 398 |
| **No. randomised/assigned to group** *(specify whether no. people or clusters)* | 29 | 398 |
| **Description** *(include sufficient detail for replication, e.g. content, dose, components)* | Usual care-no further description given | 399 |
| **Duration of treatment period** | Not specified | NA |
| **Timing** | Not reported | NA |

| | | |
|---|---|---|
| **Co-interventions** | Not reported | NA |
| **Notes** | None | |

## Outcomes

### Average time to therapeutic INR

| | **Description as stated in report/paper** | | **Page number** |
|---|---|---|---|
| **Outcome name** | Average time to therapeutic INR | | 399 (table 2) |
| **Time points measured** *(specify whether from start or end of intervention)* | Baseline; when therapeutic range was reached | | 399 (table 2) |
| **Time points reported** | Baseline; when therapeutic range was reached | | 399 (table 2) |
| **Outcome definition** *(with diagnostic criteria if relevant)* | Target range of 2.0 to 3.0 | | 399 |
| **Unit of measurement** *(if relevant)* | Days | | 398 (table 1) |
| **Scales: upper and lower limits** *(indicate whether high or low score is good)* | Within range | | 398 (table 1) |
| **Is outcome/tool validated?** | No | Bloodwork | 398 |
| **Imputation of missing data** *(e.g. assumptions made for ITT analysis)* | Not reported | | NA |
| **Assumed risk estimate** *(e.g. baseline or population risk noted in Background)* | Not reported | | NA |
| **Power** *(e.g. power & sample size calculation, level of power achieved)* | Not reported | | NA |
| **Notes** | None | | |

### Patients experiencing bleeds/adverse drug events (%)

| | **Description as stated in report/paper** | **Page number** |
|---|---|---|
| **Outcome name** | Adverse events | 399 (table 2) |

| | | | |
|---|---|---|---|
| **Time points measured** *(specify whether from start or end of intervention)* | Baseline; when therapeutic range was reached | | 399 (table 2) |
| **Time points reported** | Baseline; when therapeutic range was reached | | 399 (table 2) |
| **Outcome definition** *(with diagnostic criteria if relevant)* | Bleeding or other adverse events | | 398 (table 1) |
| **Unit of measurement** *(if relevant)* | Event data | | 399 (table 2) |
| **Scales: upper and lower limits** *(indicate whether high or low score is good)* | Within range | | 399 (table 2) |
| **Is outcome/tool validated?** | No | Reported on data collection sheet | 399 (table 2) |
| **Imputation of missing data** *(e.g. assumptions made for ITT analysis)* | Not reported | | NA |
| **Assumed risk estimate** *(e.g. baseline or population risk noted in Background)* | Not reported | | NA |
| **Power** *(e.g. power & sample size calculation, level of power achieved)* | Not reported | | NA |
| **Notes** | None | | |

## Funding/conflict of interest

| | | |
|---|---|---|
| **Study funding sources** *(including role of funders)* | Not reported | NA |
| **Possible conflicts of interest** *(for study authors)* | Not reported | NA |
| **Notes** | None | |

## Data and analysis

### Average time to therapeutic INR

| | Description as stated in report/paper | Page number |
|---|---|---|
| **Outcome** | Average time to therapeutic INR | 399 (table 2) |

| | | | | | | | |
|---|---|---|---|---|---|---|---|
| **Time point** *(specify from start or end of intervention)* | Baseline; when therapeutic range was reached | | | | | | 399 (table 2) |
| **Results** | Intervention | | | Comparison | | | 399 (table 2) |
| | Mean | Range | No. participants | Mean | Range | No. participants | |
| | 5.6 | 4-11 | 22 | 6.0 | 4-11 | 29 | |
| **Any other results reported** *(e.g. mean difference, CI, P value)* | Not reported | | | | | | NA |
| **No. missing participants** | Not reported | | | | | | NA |
| **Statistical methods used and appropriateness of these** *(e.g. adjustment for correlation)* | Not reported | | | | | | NA |
| **Notes** | One patient was removed from the protocol and managed based on the pharmacist's and physician's clinical judgment after her INR reached 7.4. She was started on the protocol upon admission, and her INR was maintained between 2 and 3 for several days. She required a transfer to the intensive care unit, where she received fluconazole. Within 2 days, her INR was 7.4 and the physician ordered 10 mg of vitamin K, which she received. Her INR was later stabilized and she had no further warfarin issues for the remainder of her hospitalization. 399 | | | | | | |

## Patients experiencing bleeds/adverse drug events

| | **Description as stated in report/paper** | | | | **Page number** |
|---|---|---|---|---|---|
| **Outcome** | Patients experiencing bleeds/adverse drug events | | | | 399 (table 2) |
| **Time point** *(specify from start or end of intervention)* | Baseline; when therapeutic range was reached | | | | 399 (table 2) |
| **Results** | Intervention | | Comparison | | 399 (table 2) |
| | No. with event | Total in group | No. with event | Total in group | |
| | 2 | 22 | 3 | 29 | |
| **Any other results reported** | Not reported | | | | NA |
| **No. missing participants** | Not reported | | | | NA |

| | | | |
|---|---|---|---|
| **Reasons missing** | Not reported | | NA |
| **Statistical methods used and appropriateness of these** | Not reported | | NA |
| **Notes** | One patient was removed from the protocol and managed based on the pharmacist's and physician's clinical judgment after her INR reached 7.4. She was started on the protocol upon admission, and her INR was maintained between 2 and 3 for several days. She required a transfer to the intensive care unit, where she received fluconazole. Within 2 days, her INR was 7.4 and the physician ordered 10 mg of vitamin K, which she received. Her INR was later stabilized and she had no further warfarin issues for the remainder of her hospitalization. 399 | | |

## Conclusions

| | Description as stated in report/paper | Page number |
|---|---|---|
| **Key conclusions of study authors** | Results showed that outcomes in the protocol group were at least as good as those in the physician-managed group *(Table 2).* | 399 |
| **Notes** | None | |

# Hahn et al. 2019

## Study eligibility

| Study Characteristics | Eligibility criteria |
|---|---|
| **Title** | Effect of Pharmacist Clinic Visits on 30-Day Heart Failure Readmission Rates at a County Hospital |
| **Author (year)** | Hahn et al. 2019 |
| **Country** | Texas, USA |
| **Type of study** | Retrospective cohort study |
| **Participants** | Patients were 18 years or older with an index heart failure (HF) exacerbation admission. |
| **Types of intervention** | Clinical pharmacy specialist (CPS) with collaborative practice agreement (CPA) (High Intensity Bundle) |
| **Types of comparison** | • Medication therapy management (MTM) pharmacist without CPA (Low Intensity Bundle)<br>• No pharmacist (Standard of Care [SOC]). |
| **Types of outcome measures** | 30-day all-cause readmission rate; 30-day HF readmissions; average number of days until readmission in those who were readmitted; adherence to follow-up pharmacist appointments; number of days from discharge to the first pharmacist follow-up visit; and average number of days from discharge to the first physician follow-up visit. |
| **Prescriptive authority** | Collaborative practice agreement |

| | |
|---|---|
| **Include/Exclude** | Include |
| **Notes** | None |

**DO NOT PROCEED IF STUDY EXCLUDED FROM REVIEW**

## Characteristics of included studies

### Methods

| | Descriptions as stated in report/paper | | Page number |
|---|---|---|---|
| **Aim of study** | To evaluate post-discharge care provided by either a clinical pharmacy specialist (CPS) with CPA, MTM pharmacist without CPA, or no pharmacist and their impact on all-cause and HF readmission rates. | | 359 |
| **Design** | Retrospective cohort study | | 359 |
| **Unit of allocation** *(by individuals, cluster/ groups or body parts)* | Individuals | | 359 |
| **Start-end date** | January 2015-July 2017 | | 359 |
| **Duration of participation** *(from recruitment to last follow-up)* | January 2015-July 2017 | | 359 |
| **Study duration** | 30 day follow-up | | |
| **Ethical approval needed/obtained for study** | Yes | This study was approved by University of Texas at Southwestern Institutional Review Board. | 359 |
| **Notes** | Patients were randomly assigned to each bundle. 359 | | |

### Participants

| | **Description** *Include comparative information for each intervention or comparison group if available* | Page number |
|---|---|---|
| **Population description** *(from which study participants are drawn)* | Patients were 18 years or older with an index heart failure (HF) exacerbation admission. | 359 |
| **Setting** *(including location and social context)* | Identified inpatient, seen post discharge outpatient | 359 |

| | | |
|---|---|---|
| **Inclusion/exclusion criteria** | Criteria for inclusion were patients with a HF exacerbation index admission and were at least 18 years old. Criteria for exclusion were patients who were identified by the PIECES e-Model as being at high risk for readmission who received an intensive bundle of coordinated multidisciplinary services, had a diagnosis of end-stage renal disease (ESRD), were actively incarcerated, had admission to the inpatient psychiatric facility, were actively pregnant, were enrolled in hospice care, had completed appointments with both a CPS with CPA and MTM pharmacist without CPA post-discharge, or were seen in congestive heart failure (CHF) clinic. | 359 |
| **Method of recruitment of participants** | Electronic medical record | 359 |
| **Informed consent obtained** | Not reported | NA |
| **Total no. randomised** *(or total pop. at start of study for NRCTs)* | 98 | 360 (figure 1) |
| **Clusters** | NA | NA |
| **Baseline imbalances** | Baseline characteristics were similar except for gender (Table 1). Patients in the control arm had more unfunded patients compared to those in the CPS with CPA or MTM without CPA arms (Table 1). | 361 (table 1) |
| **Withdrawals and exclusions** | A total of 683 patients were assessed for eligibility with 98 patients meeting study inclusion criteria (Figure 1). Among the excluded patients, the primary reasons for exclusion were due to enrollment through the PIECES e-Model (n = 264) and scheduled appointment in CHF clinic (n = 127). | 360 |
| **Age (years)** | Intervention: Mean 60.0 ± SD 13.0<br>Comparator 1 (MTM with no CPA): Mean 63.0 ± SD 14.0<br>Comparator 2 (mean): Mean 59.0 ± SD 14.0 | 361 (table 1) |
| **Sex (female)** | Intervention: 62.9%<br>Comparator 1: 46.4%<br>Comparator 2: 34.3% | 361 (table 1) |
| **Subgroups measure** | Not reported | NA |
| **Subgroups reported** | Not reported | NA |
| **Notes** | None | |

## Intervention group

| | **Description as stated in report/paper** | **Page number** |
|---|---|---|
| **Group name** | Clinical pharmacy specialist with collaborative practice agreement | 359 |

| | | |
|---|---|---|
| **No. randomised/assigned to group** *(specify whether no. people or clusters)* | 35 | 359 |
| **Description** *(include sufficient detail for replication, e.g. content, dose, components)* | Patients seen by a CPS were managed under a CPA with cardiology physician supervision recognized by the Texas State Board of Pharmacy and were scheduled with the goal to be seen within 10 days after discharge.<br><br>Interventions provided by the CPS with CPA included similar interventions to MTM pharmacists. However, interventions also included ordering referrals (primary care physician [PCP], nutrition, smoking cessation, medication access specialist, and anticoagulation management), making therapeutic medication changes including medication discontinuations and initiations, ordering medication refills and labs, and triaging acute issues by admitting patients directly to the Emergency Department (ED).<br><br>The CPA also allowed the pharmacist to provide follow-up care during the 21-days post-hospital discharge with a second visit within 7 to 21 days of the initial visit, depending on the acuity of symptoms and patients' understanding of diet, fluid intake, and medication adherence.<br><br>Patients were discharged from the CPS clinic if clinically stable with appropriate follow-up. | 359 |
| **Duration of treatment period** | Discharge, within 10 days after discharge, follow-up care during 21 days post-discharge | 359 |
| **Timing** | At discharge | 359 |
| **Co-interventions** | Medication therapy management | 359 |
| **Notes** | None | |

## Comparator group 1

| | Description as stated in report/paper | Page number |
|---|---|---|
| **Group name** | Medication therapy management (MTM) pharmacist without CPA | 359 |
| **No. randomised/assigned to group** *(specify whether no. people or clusters)* | 28 | 359 |

| | | |
|---|---|---|
| **Description** *(include sufficient detail for replication, e.g. content, dose, components)* | such as providing adherence tools and educating on therapeutic lifestyle changes, medication adherence, and disease states. | |
| **Duration of treatment period** | Patients were identified during hospital admission when a consult was ordered for pharmacist medication discharge counseling. Patients were then scheduled a post-hospital discharge appointment with the MTM pharmacist with the goal to be seen within 10 days<br>after discharge.<br>Patients were provided information describing MTM services in the hospital and were scheduled to have a telephone or face-to-face visit after the initial hospital encounter.<br>Interventions provided by the MTM pharmacist without CPA included reconciling medications, providing adherence tools, educating on therapeutic lifestyle changes, medication adherence, and disease states, providing information on when to seek ED care, coordinating care, and providing recommendations to physicians to optimize therapy | 359 |
| **Timing** | At discharge, within 10 days after discharge | 359 |
| **Co-interventions** | Not reported | NA |
| **Notes** | None | |

## Comparator group 2

| | **Description as stated in report/paper** | **Page number** |
|---|---|---|
| **Group name** | Standard care | 359 |
| **No. randomised/assigned to group** *(specify whether no. people or clusters)* | 35 | 359 |
| **Description** *(include sufficient detail for replication, e.g. content, dose, components)* | Patients in the control arm had a pharmacist consult for discharge counseling ordered during the index admission. However, they did not receive a follow-up appointment with either a MTM pharmacist or CPS after discharge<br>due to limited resources. | 359 |
| **Duration of treatment period** | One session at discharge | 359 |
| **Timing** | At discharge | 359 |
| **Co-interventions** | Not reported | 359 |
| **Notes** | None | |

## Outcomes

### 30-day all cause readmissions of HF patients

| | Description as stated in report/paper | | Page number |
|---|---|---|---|
| **Outcome name** | 30-day all cause readmissions of HF patients | | 359 |
| **Time points measured** *(specify whether from start or end of intervention)* | 30 days post-discharge | | 359 |
| **Time points reported** | 30 days post-discharge | | 359 |
| **Outcome definition** *(with diagnostic criteria if relevant)* | 30-day all cause readmissions of heart failure patients | | 359 |
| **Unit of measurement** *(if relevant)* | Event data | | NA |
| **Scales: upper and lower limits** *(indicate whether high or low score is good)* | Lower scores are desired | | NA |
| **Is outcome/tool validated?** | No | | NA |
| **Imputation of missing data** *(e.g. assumptions made for ITT analysis)* | NA | | NA |
| **Assumed risk estimate** *(e.g. baseline or population risk noted in Background)* | Not reported | | NA |
| **Power** *(e.g. power & sample size calculation, level of power achieved)* | Not reported | | NA |
| **Notes** | None | | |

### Rate of 30-day HF readmissions

| | Description as stated in report/paper | Page number |
|---|---|---|
| **Outcome name** | Rate of 30-day HF readmissions | 359 |
| **Time points measured** *(specify whether from start or end of intervention)* | Rate of 30-day HF readmissions | 359 |
| **Time points reported** | 30 days post-discharge | 359 |

| | | | |
|---|---|---|---|
| **Outcome definition** *(with diagnostic criteria if relevant)* | 30-day readmissions due to heart failure | | 359 |
| **Unit of measurement** *(if relevant)* | Event data | | NA |
| **Scales: upper and lower limits** *(indicate whether high or low score is good)* | Lower scores are desired | | NA |
| **Is outcome/tool validated?** | No | | NA |
| **Imputation of missing data** *(e.g. assumptions made for ITT analysis)* | NA | | NA |
| **Assumed risk estimate** *(e.g. baseline or population risk noted in Background)* | Not reported | | NA |
| **Power** *(e.g. power & sample size calculation, level of power achieved)* | Not reported | | NA |
| **Notes** | None | | |

### Number of ED visits

| | **Description as stated in report/paper** | | **Page number** |
|---|---|---|---|
| **Outcome name** | Number of ED visits | | 359 |
| **Time points measured** *(specify whether from start or end of intervention)* | 30 days post-discharge | | 359 |
| **Time points reported** | 30 days post-discharge | | 359 |
| **Outcome definition** *(with diagnostic criteria if relevant)* | Number of emergency department visits in 30 days post discharge | | 359 |
| **Unit of measurement** *(if relevant)* | Event data | | NA |
| **Scales: upper and lower limits** *(indicate whether high or low score is good)* | Lower scores are desired | | NA |
| **Is outcome/tool validated?** | No | | NA |

| | | |
|---|---|---|
| **Imputation of missing data** *(e.g. assumptions made for ITT analysis)* | NA | NA |
| **Assumed risk estimate** *(e.g. baseline or population risk noted in Background)* | Not reported | NA |
| **Power** *(e.g. power & sample size calculation, level of power achieved)* | Not reported | NA |
| **Notes** | None | |

## Funding/conflict of interest

| | | |
|---|---|---|
| **Study funding sources** *(including role of funders)* | The author(s) received no financial support for the research, authorship, and/or publication of this article. | 364 |
| **Possible conflicts of interest** *(for study authors)* | The author(s) declared no potential conflicts of interest with respect to the research, authorship, and/or publication of this article. | 364 |
| **Notes** | None | |

## Data and analysis

### 30-day all cause readmissions of HF patients

| | Description as stated in report/paper | | | | Page number |
|---|---|---|---|---|---|
| **Outcome** | 30-day all cause readmissions of HF patients | | | | 362 (table 2) |
| **Time point** *(specify from start or end of intervention)* | 30 days post discharge | | | | 362 (table 2) |
| **Results** | Intervention | | Comparison 1 | | 362 (table 2) |
| | No. with event | Total in group | No. with event | Total in group | |
| | 3 | 35 | 2 | 28 | |
| | Intervention | | Comparison 2 | | 362 (table 2) |
| | No. with event | Total in group | No. with event | Total in group | |
| | 3 | 35 | 9 | 35 | |
| **Any other results reported** | Intervention vs comparison 1: p=0.057<br>Intervention vs. comparison 2: p=0.046 | | | | 362 (table 2) |
| **No. missing participants** | 0 | | | | NA |
| **Reasons missing** | NA | | | | NA |

| | | |
|---|---|---|
| **Statistical methods used and appropriateness of these** | Descriptive statistics were used to assess the baseline characteristics. Nominal variables of baseline characteristics and readmission rates were evaluated using a chi-square test and/or Fisher exact test when appropriate. Continuous variables of baseline characteristics were evaluated using a Mann– Whitney test except for age in which a *t* test was used. A Log Rank (Mantel–Cox) analysis was used to assess the primary outcome. For the secondary outcome of HF readmissions, a trended analysis was used to identify if incremental differences existed between groups. An a priori significance level of 0.05 was used. SPSS Version 19 was utilized to assess nominal and continuous variables when applicable. | 360 |
| **Notes** | None | |

## Rate of 30-day HF readmissions

| | Description as stated in report/paper | | | | Page number |
|---|---|---|---|---|---|
| **Outcome** | Rate of 30-day HF readmissions | | | | 362 (table 2) |
| **Time point** *(specify from start or end of intervention)* | 30 days post-discharge | | | | 362 (table 2) |
| **Results** | Intervention | | Comparison 1 | | 362 (table 2) |
| | No. with event | Total in group | No. with event | Total in group | |
| | 1 | 35 | 2 | 28 | |
| | Intervention | | Comparison 2 | | 362 (table 2) |
| | No. with event | Total in group | No. with event | Total in group | |
| | 1 | 35 | 6 | 35 | |
| **Any other results reported** | Intervention vs comparison 1: p=0.28<br>Intervention vs. comparison 2: p=0.11 | | | | 362 (table 2) |
| **No. missing participants** | 0 | | | | NA |
| **Reasons missing** | NA | | | | NA |

| | | |
|---|---|---|
| **Statistical methods used and appropriateness of these** | Descriptive statistics were used to assess the baseline characteristics. Nominal variables of baseline characteristics and readmission rates were evaluated using a chi-square test and/or Fisher exact test when appropriate. Continuous variables of baseline characteristics were evaluated using a Mann– Whitney test except for age in which a *t* test was used. A Log Rank (Mantel–Cox) analysis was used to assess the primary outcome. For the secondary outcome of HF readmissions, a trended analysis was used to identify if incremental differences existed between groups. An a priori significance level of 0.05 was used. SPSS Version 19 was utilized to assess nominal and continuous variables when applicable. | 360 |
| **Notes** | None | |

## Number of ED visits

| | Description as stated in report/paper | | | | Page number |
|---|---|---|---|---|---|
| **Outcome** | Number of ED visits | | | | 362 (table 2) |
| **Time point** *(specify from start or end of intervention)* | 30 days post-discharge | | | | 362 (table 2) |
| **Results** | Intervention | | Comparison 1 | | 362 (table 2) |
| | No. with event | Total in group | No. with event | Total in group | |
| | 5 | 35 | 6 | 28 | |
| | Intervention | | Comparison 2 | | 362 (table 2) |
| | No. with event | Total in group | No. with event | Total in group | |
| | 5 | 35 | 0 | 35 | |
| **Any other results reported** | Not enough information for comparison | | | | 362 (table 2) |
| **No. missing participants** | 0 | | | | NA |
| **Reasons missing** | NA | | | | NA |

| | | |
|---|---|---|
| **Statistical methods used and appropriateness of these** | Descriptive statistics were used to assess the baseline characteristics. Nominal variables of baseline characteristics and readmission rates were evaluated using a chi-square test and/or Fisher exact test when appropriate. Continuous variables of baseline characteristics were evaluated using a Mann– Whitney test except for age in which a *t* test was used. A Log Rank (Mantel–Cox) analysis was used to assess the primary outcome. For the secondary outcome of HF readmissions, a trended analysis was used to identify if incremental differences existed between groups. An a priori significance level of 0.05 was used. SPSS Version 19 was utilized to assess nominal and continuous variables when applicable. | 360 |
| **Notes** | None | |

## Conclusions

| | **Description as stated in report/paper** | **Page number** |
|---|---|---|
| **Key conclusions of study authors** | This retrospective cohort study demonstrated that follow-up appointments with a CPS with CPA or MTM pharmacist without CPA after hospital discharge were associated with lower 30-day all-cause readmission rates. post-discharge follow-up visits with a CPS with CPA may be more effective in reducing HF readmissions compared to visits performed by a MTM pharmacist without CPA. | 364 |
| **Notes** | None | |

# Hall et al. 2011

## Study eligibility

| **Study Characteristics** | **Eligibility criteria** |
|---|---|
| **Title** | Health Care Expenditures and Therapeutic Outcomes of a Pharmacist-Managed Anticoagulation Service versus Usual Medical Care |
| **Author (year)** | Hall et al. (2011) |
| **Country** | Pennsylvania, USA |
| **Type of study** | Retrospective cohort |
| **Participants** | Adults who received warfarin therapy |
| **Types of intervention** | Pharmacist-managed anticoagulation service |

| | |
|---|---|
| **Types of comparison** | Usual care |
| **Types of outcome measures** | Direct anticoagulation service cost and overall medical care costs; anticoagulation-related adverse events; hospitalizations and emergency department visits; frequency of international normalized ratio (INR) testing; quantity of warfarin refills |
| **Prescriptive authority** | Collaborative practice agreement |
| **Include/Exclude** | Include |
| **Notes** | None |

**DO NOT PROCEED IF STUDY EXCLUDED FROM REVIEW**

## Characteristics of included studies

### Methods

| | Descriptions as stated in report/paper | | Page number |
|---|---|---|---|
| **Aim of study** | To evaluate the differences in health care expenditures while accounting for operational costs, therapeutic outcomes, and patient compliance with laboratory tests and warfarin refills in patients receiving warfarin therapy management by a pharmacist managed anticoagulation service compared with those receiving usual medical care. | | 687 |
| **Design** | Retrospective cohort | | 688 |
| **Unit of allocation** *(by individuals, cluster/ groups or body parts)* | Individuals | | 688 |
| **Start-end date** | October 2007 – September 2008 | | 688 |
| **Duration of participation** *(from recruitment to last follow-up/ baseline to last follow-up- group level)* *1. Time of consent until last measurement for each individual.* *2. Baseline to final follow-up for each individual* | Patients had at least two INR measurements taken within any 60-day period throughout the study period were eligible for participation in the study. Patients in the anticoagulation service group must have received care through the anticoagulation service for at least 2 months during the study period.<br><br>Mean follow-up in intervention was 99.3 days. Mean follow-up in usual care was 103.8 days. | | 688<br><br>690 (table 2) |
| **Study duration** *(as above with the exception of interim analyses or other circumstances* | Patients had at least two INR measurements taken within any 60-day period throughout the study period were eligible for participation in the study. Patients in the anticoagulation service group must have received care through the anticoagulation service for at least 2 months during the study period. | | 688 |
| **Ethical approval needed/obtained for study** | Yes | The study was reviewed and approved by the University of Pittsburgh Institutional Review Board. | 688 |
| **Notes** | None | | |

### Participants

| | Description<br>*Include comparative information for each intervention or comparison group if available* | | Page number |
|---|---|---|---|
| **Population description**<br>*(from which study participants are drawn)* | Patients who had at least two INR measurements taken within any 60-day period throughout the study period were eligible for participation in the study. Patients in the anticoagulation service group must have received care through the anticoagulation service for at least 2 months during the study period. | | 688 |
| **Setting**<br>*(including location and social context)* | Outpatient clinic | | 688 |
| **Inclusion/exclusion criteria** | Patients who had UPMC Health Plan medical insurance between October 1, 2007, and September 30, 2008 (study period) and had at least two INR measurements taken within any 60-day period throughout the study period were eligible for participation in the study. Patients in the anticoagulation service group must have received care through the anticoagulation service for at least 2 months during the study period. | | 688 |
| **Method of recruitment of participants** | Electronic health records | | 688 |
| **Informed consent obtained** | Not reported | NA | NA |
| **Total no. randomised**<br>*(or total pop. at start of study for NRCTs)* | 350 individuals | | 688 |
| **Clusters** | NA | | NA |
| **Baseline imbalances** | No significant difference between following variables:<br>• Age (yrs)<br>• Follow-up period (days)<br>• No. (%) of Patients Female<br>• Anticoagulation indication<br>• Arial fibrillation<br>• Heart valve replacement<br>• Coronary artery disease<br>• Cerebral vascular disease<br>• Hypercoagulable state | | 690 (table 2) |
| **Withdrawals and exclusions** | None | | NA |
| **Age** | Intervention: 63.7 years<br>Comparator: 65.1 years | | 690 (table 2) |
| **Sex (female)** | Intervention: 77%<br>Comparator: 77% | | 690 (table 2) |

| | | |
|---|---|---|
| **Subgroups measure** | Not reported | NA |
| **Subgroups reported** | Not reported | NA |
| **Notes** | None | |

## Intervention group

| | Description as stated in report/paper | Page number |
|---|---|---|
| **Group name** | Pharmacist-managed anticoagulation service | 688 |
| **No. randomised/assigned to group** *(specify whether no. people or clusters)* | 175 | 690 (table 2) |
| **Description** *(include sufficient detail for replication, e.g. content, dose, components)* | The pharmacists manage patients' anticoagulation through a collaborative care agreement and protocol under the referring physician's authority. Patients are scheduled to have blood tests performed on specific dates and may go to the laboratory of their choice. The INR results are faxed to the anticoagulation service or retrieved through interface with the electronic medical record system (EPIC; Verona, WI). All contact with the patients occurs by telephone and is documented in the outpatient electronic medical record system. The anticoagulation service manages about 140 INR results/day, including those from patients starting therapy and from patients receiving low-molecular weight heparin as bridge to warfarin therapy. The primary function of the pharmacy technician is retrieving INR results, rescheduling missed appointments, and triaging incoming telephone calls. The pharmacist focuses on providing direct patient care by contacting patients with INR results, handling new referrals, and managing issues with chronic anticoagulation, such as interruptions in therapy, bridging with low-molecular-weight heparin, and changes in drug therapy and diet. The UPMC anticoagulation service also serves as a learning site for pharmacy students and residents. | 688 |
| **Duration of treatment period** | Mean follow-up was 99.3 days | 690 (table 2) |
| **Timing** | Not reported | NA |
| **Co-interventions** | Not reported | NA |
| **Notes** | None | |

## Comparator group

| | Description as stated in report/paper | Page number |
|---|---|---|

| | | |
|---|---|---|
| **Group name** | Usual care | 688 |
| **No. randomised/assigned to group** *(specify whether no. people or clusters)* | 175 | 690 (table 2) |
| **Description** *(include sufficient detail for replication, e.g. content, dose, components)* | Patients in the usual care group were not followed by the anticoagulation service at any time during the study period. The usual care (comparison) group was created by matching on the inclusion criteria and age within 10 years, sex, length of follow-up period in the program, and therapeutic indication based on *International Classification of Diseases, Ninth Revision* (ICD-9) codes. | 688 |
| **Duration of treatment period** | Mean follow-up was 103.8 days. | 690 |
| **Timing** | Not reported | NA |
| **Co-interventions** | Not reported | NA |
| **Notes** | None | |

## Outcomes

### INR within therapeutic range

| | **Description as stated in report/paper** | | **Page number** |
|---|---|---|---|
| **Outcome name** | INR within therapeutic range | | 691 |
| **Time points measured** *(specify whether from start or end of intervention)* | Not reported | | NA |
| **Time points reported** | Not reported | | NA |
| **Outcome definition** *(with diagnostic criteria if relevant)* | INR within therapeutic range. The INR goal range for each therapeutic indication was in accordance with American College of Chest Physicians' clinical practice guidelines (Table 1)<br>For this analysis, an INR result was considered within goal range if it was within ± 0.2 of the upper and lower goal range limit | | 689 table 1, 690 |
| **Unit of measurement** *(if relevant)* | % | | 691 |
| **Scales: upper and lower limits** *(indicate whether high or low score is good)* | Within range of 2.0-3.5 | | 689 table 1 |
| **Is outcome/tool validated?** | No | NA | NA |

| | | |
|---|---|---|
| **Imputation of missing data** *(e.g. assumptions made for ITT analysis)* | NA | NA |
| **Assumed risk estimate** *(e.g. baseline or population risk noted in Background)* | Not reported | NA |
| **Power** *(e.g. power & sample size calculation, level of power achieved)* | Sample size was determined by a power curve analysis that indicated that 100 matched pairs would be needed to detect a significant difference (P ≤ 0.05) in time in INR range of at least 0.1 with 0.8 power. | 689 |
| **Notes** | None | |

## Time that INR was within therapeutic range

| | Description as stated in report/paper | | Page number |
|---|---|---|---|
| **Outcome name** | Time that INR was within therapeutic range | | 691/692 |
| **Time points measured** *(specify whether from start or end of intervention)* | Not reported | | NA |
| **Time points reported** | Not reported | | NA |
| **Outcome definition** *(with diagnostic criteria if relevant)* | Percentage of time that INR within therapeutic range. | | 690 |
| **Unit of measurement** *(if relevant)* | % | | 691 |
| **Scales: upper and lower limits** *(indicate whether high or low score is good)* | Higher scores are desired | | NA |
| **Is outcome/tool validated?** | No | NA | NA |
| **Imputation of missing data** *(e.g. assumptions made for ITT analysis)* | NA | | NA |
| **Assumed risk estimate** *(e.g. baseline or population risk noted in Background)* | Not reported | | NA |

| | | |
|---|---|---|
| **Power** *(e.g. power & sample size calculation, level of power achieved)* | Sample size was determined by a power curve analysis that indicated that 100 matched pairs would be needed to detect a significant difference (P ≤ 0.05) in time in INR range of at least 0.1 with 0.8 power. | 689 |
| **Notes** | None | |

## Anticoagulation-related adverse events

| | Description as stated in report/paper | | Page number |
|---|---|---|---|
| **Outcome name** | Anticoagulation-related adverse events | | 692 |
| **Time points measured** *(specify whether from start or end of intervention)* | Not reported | | NA |
| **Time points reported** | Not reported | | NA |
| **Outcome definition** *(with diagnostic criteria if relevant)* | Anticoagulation-related adverse events | | 692 |
| **Unit of measurement** *(if relevant)* | Event data | | 692 |
| **Scales: upper and lower limits** *(indicate whether high or low score is good)* | Lower scores are desired | | NA |
| **Is outcome/tool validated?** | No | NA | NA |
| **Imputation of missing data** *(e.g. assumptions made for ITT analysis)* | NA | | NA |
| **Assumed risk estimate** *(e.g. baseline or population risk noted in Background)* | Not reported | | NA |
| **Power** *(e.g. power & sample size calculation, level of power achieved)* | Sample size was determined by a power curve analysis that indicated that 100 matched pairs would be needed to detect a significant difference (P ≤ 0.05) in time in INR range of at least 0.1 with 0.8 power. | | 689 |
| **Notes** | None | | |

## Anticoagulation-related hospital admissions

| | Description as stated in report/paper | Page number |
|---|---|---|
| **Outcome name** | Anticoagulation-related hospital admissions | 692 |

| | | | |
|---|---|---|---|
| **Time points measured** *(specify whether from start or end of intervention)* | Not reported | | NA |
| **Time points reported** | Not reported | | NA |
| **Outcome definition** *(with diagnostic criteria if relevant)* | Anticoagulation-related hospital admissions | | 692 |
| **Unit of measurement** *(if relevant)* | Event data | | 692 |
| **Scales: upper and lower limits** *(indicate whether high or low score is good)* | Lower scores are desired | | NA |
| **Is outcome/tool validated?** | No | NA | NA |
| **Imputation of missing data** *(e.g. assumptions made for ITT analysis)* | NA | | NA |
| **Assumed risk estimate** *(e.g. baseline or population risk noted in Background)* | Not reported | | NA |
| **Power** *(e.g. power & sample size calculation, level of power achieved)* | Sample size was determined by a power curve analysis that indicated that 100 matched pairs would be needed to detect a significant difference ($P \leq 0.05$) in time in INR range of at least 0.1 with 0.8 power. | | 689 |
| **Notes** | None | | |

## Anticoagulation-related emergency department visits

| | **Description as stated in report/paper** | **Page number** |
|---|---|---|
| **Outcome name** | Anticoagulation-related emergency department visits | 692 |
| **Time points measured** *(specify whether from start or end of intervention)* | Not reported | NA |
| **Time points reported** | Not reported | NA |
| **Outcome definition** *(with diagnostic criteria if relevant)* | Anticoagulation-related emergency department visits | 692 |
| **Unit of measurement** *(if relevant)* | Event data | 692 |

| | | | |
|---|---|---|---|
| **Scales: upper and lower limits** *(indicate whether high or low score is good)* | Lower scores are desired | | NA |
| **Is outcome/tool validated?** | No | NA | NA |
| **Imputation of missing data** *(e.g. assumptions made for ITT analysis)* | NA | | NA |
| **Assumed risk estimate** *(e.g. baseline or population risk noted in Background)* | Not reported | | NA |
| **Power** *(e.g. power & sample size calculation, level of power achieved)* | Sample size was determined by a power curve analysis that indicated that 100 matched pairs would be needed to detect a significant difference ($P \leq 0.05$) in time in INR range of at least 0.1 with 0.8 power. | | 689 |
| **Notes** | None | | |

## Funding/conflict of interest

| | | |
|---|---|---|
| **Study funding sources** *(including role of funders)* | Not reported | NA |
| **Possible conflicts of interest** *(for study authors)* | Not reported | NA |
| **Notes** | None | |

## Data and analysis

### INR within therapeutic range

| | **Description as stated in report/paper** | | | | **Page number** |
|---|---|---|---|---|---|
| **Outcome** | INR within therapeutic range | | | | 692 (table 5) |
| **Time point** *(specify from start or end of intervention)* | Not reported | | | | NA |
| **Results** | Intervention | | Comparison | | 692 (table 5) |
| | No. with event | Total in group | No. with event | Total in group | |
| | 118 (67.2%) | 175 | 96 (54.6%) | 175 | |
| **Any other results reported** | p<0.0001 | | | | |
| **No. missing participants** | 0 | | NA | | NA |
| **Reasons missing** | NA | | NA | | NA |

| | | |
|---|---|---|
| **Statistical methods used and appropriateness of these** | For this analysis, an INR result was considered within goal range if it was within 0.2 of the upper and lower goal range limits. The time spent in INR range was determined by using linear interpolation methodology to assign an INR value to each day throughout the assessment period for each patient. The interpolated values were used to calculate patient time in target range, and differences between groups were examined by using a standard *t* test. | 690 |
| **Confounders** | Not reported | NA |
| **Notes** | None | |

## Time that INR was within therapeutic range

| | Description as stated in report/paper | | | | Page number |
|---|---|---|---|---|---|
| **Outcome** | Time that INR was within therapeutic range | | | | 692 (table 5) |
| **Time point** *(specify from start or end of intervention)* | Not reported | | | | NA |
| **Results** | Intervention | | Comparison | | 692 (table 5) |
| | No. with event | Total in group | No. with event | Total in group | |
| | 129 (73.3%) | 175 | 107 (61.3%) | 175 | |
| **Any other results reported** | p<0.0001 | | | | 692 (table 5) |
| **No. missing participants** | 0 | | NA | | NA |
| **Reasons missing** | NA | | NA | | NA |
| **Statistical methods used and appropriateness of these** | For this analysis, an INR result was considered within goal range if it was within 0.2 of the upper and lower goal range limits. The time spent in INR range was determined by using linear interpolation methodology to assign an INR value to each day throughout the assessment period for each patient. The interpolated values were used to calculate patient time in target range, and differences between groups were examined by using a standard *t* test. | | | | 690 |
| **Confounders** | Not reported | | | | NA |
| **Notes** | None | | | | |

## Anticoagulation-related adverse events

| | Description as stated in report/paper | | | | Page number |
|---|---|---|---|---|---|
| **Outcome** | Anticoagulation-related adverse events | | | | 691 |
| **Time point** *(specify from start or end of intervention)* | Not reported | | | | NA |
| **Results** | Intervention | | Comparison | | 691 |
| | No. of events | Total in group | No. of events | Total in group | |

| | | | | | |
|---|---|---|---|---|---|
| | 14 | 175 | 41 | 175 | |
| **Any other results reported** | p<0.0001<br>In the anticoagulation service group, nine patients experienced 14 anticoagulation-related adverse events, for an overall event rate of 5.1%, whereas in the usual care group, 27 patients experienced 41 anticoagulation-related adverse events, for an event rate of 15.4% (p<0.0001) | | | | 691 |
| **No. missing participants** | 0 | | NA | | NA |
| **Reasons missing** | NA | | NA | | NA |
| **Statistical methods used and appropriateness of these** | For this analysis, an INR result was considered within goal range if it was within 0.2 of the upper and lower goal range limits. The time spent in INR range was determined by using linear interpolation methodology to assign an INR value to each day throughout the assessment period for each patient. The interpolated values were used to calculate patient time in target range, and differences between groups were examined by using a standard *t* test. | | | | 690 |
| **Confounders** | Not reported | | | | NA |
| **Notes** | None | | | | |

## Anticoagulation-related hospital admissions

| | **Description as stated in report/paper** | | | | **Page number** |
|---|---|---|---|---|---|
| **Outcome** | Anticoagulation-related hospital admissions | | | | 691 |
| **Time point** *(specify from start or end of intervention)* | Not reported | | | | NA |
| **Results** | Intervention | | Comparison | | 691 |
| | No. with event | Total in group | No. with event | Total in group | |
| | 3 | 175 | 14 | 175 | |
| **Any other results reported** | p<0.00001 | | | | |
| **No. missing participants** | 0 | | NA | | NA |
| **Reasons missing** | NA | | NA | | NA |
| **Statistical methods used and appropriateness of these** | For this analysis, an INR result was considered within goal range if it was within 0.2 of the upper and lower goal range limits. The time spent in INR range was determined by using linear interpolation methodology to assign an INR value to each day throughout the assessment period for each patient. The interpolated values were used to calculate patient time in target range, and differences between groups were examined by using a standard *t* test. | | | | 690 |
| **Confounders** | Not reported | | | | NA |
| **Notes** | None | | | | |

### Anticoagulation-related emergency department visits

| | Description as stated in report/paper | | | | Page number |
|---|---|---|---|---|---|
| **Outcome** | Anticoagulation-related emergency department visits | | | | 691 |
| **Time point** *(specify from start or end of intervention)* | Not reported | | | | NA |
| **Results** | Intervention | | Comparison | | 691 |
| | No. with event | Total in group | No. with event | Total in group | |
| | 58 | 175 | 134 | 175 | |
| **Any other results reported** | p<0.00001 | | | | 691 |
| **No. missing participants** | 0 | | NA | | NA |
| **Reasons missing** | NA | | NA | | NA |
| **Statistical methods used and appropriateness of these** | For this analysis, an INR result was considered within goal range if it was within 0.2 of the upper and lower goal range limits. The time spent in INR range was determined by using linear interpolation methodology to assign an INR value to each day throughout the assessment period for each patient. The interpolated values were used to calculate patient time in target range, and differences between groups were examined by using a standard *t* test. | | | | 690 |
| **Confounders** | Not reported | | | | NA |
| **Notes** | None | | | | |

### Conclusions

| | Description as stated in report/paper | Page number |
|---|---|---|
| **Key conclusions of study authors** | The anticoagulation service group also demonstrated improved anticoagulation control and fewer adverse events compared with the matched comparison group. | 691 |
| **Notes** | None | |

## Hernández-Muñoz et al. 2021

### Study eligibility

| **Study Characteristics** | **Eligibility criteria** |
|---|---|
| **Title** | Impact of Pharmacist-Led Drug Therapy Management Services on HbA1c Values in a Predominantly Hispanic Population Visiting an Outpatient Endocrinology Clinic |

| | |
|---|---|
| **Author (year)** | Hernández-Muñoz et al. 2021 |
| **Country** | Texas, USA |
| **Type of study** | Retrospective cohort |
| **Participants** | Patients 18 years old and a diagnosis of type 2 diabetes mellitus at the index date were included. |
| **Types of intervention** | Diabetic care collaborative management (two pharmacists working with six endocrinologists) |
| **Types of comparison** | Standard care |
| **Types of outcome measures** | HbA1C levels |
| **Prescriptive authority** | Collaborative practice agreement |
| **Include/Exclude** | Include |
| **Notes** | Only subgroup analysis excluding registered dietitian and licensed professional counsellor reported on. |

**DO NOT PROCEED IF STUDY EXCLUDED FROM REVIEW**

## Characteristics of included studies

### Methods

| | Descriptions as stated in report/paper | | Page number |
|---|---|---|---|
| **Aim of study** | This study aims to analyze a year's worth of endocrinologists' referral data to describe the impact of the pharmacist-led DCCM program on the absolute change in HbA1c from baseline as compared to the group of diabetic patients not referred to the pharmacists by the endocrinologists. | | 858 |
| **Design** | Retrospective cohort | | 858 |
| **Unit of allocation** *(by individuals, cluster/ groups or body parts)* | Individuals | | 858 |
| **Start-end date** | September 2017 - October 2018 | | 858 |
| **Duration of participation** *(from recruitment to last follow-up)* | The mean+SD number of days between the pre- and post-HbA1c was 108 + 34 days. | | 862 |
| **Study duration** | Not reported | | NA |
| **Ethical approval needed/obtained for study** | Yes | The research proposal was reviewed and approved by the institutional review board. | 858 |
| **Notes** | None | | |

### Participants

| | **Description**<br>*Include comparative information for each intervention or comparison group if available* | **Page number** |
|---|---|---|
| **Population description**<br>*(from which study participants are drawn)* | Patients 18 years old and a diagnosis of type 2 diabetes mellitus at the index date were included. | 858 |

| | | | |
|---|---|---|---|
| **Setting** *(including location and social context)* | Hospital outpatient clinic | | 858 |
| **Inclusion/exclusion criteria** | Patients >18 years old and a diagnosis of type 2 diabetes mellitus at the index date were included. Patients without pre and postindex date HbA1c information, with a preindex date HbA1c <7% or with <28 days between the index date and postintervention HbA1c test were excluded. | | 858 |
| **Method of recruitment of participants** | Electronic health records | | 858 |
| **Informed consent obtained** | No | | NA |
| **Total no. randomised** *(or total pop. at start of study for NRCTs)* | Intervention subgroup: 35<br>Standard care: 86 | | 861 (figure 1) |
| **Clusters** | NA | | NA |
| **Baseline imbalances** | Not reported for subgroup populations | | NA |
| **Withdrawals and exclusions** | 0 | | NA |
| **Age** | Not reported for subgroup populations | | NA |
| **Sex (female)** | Not reported for subgroup populations | | NA |
| **Subgroups measure** | HbA1C | | 859 |
| **Subgroups reported** | Pharmacist-led diabetes management subgroup with no co-interventions | | 859 |
| **Notes** | | | |

## Intervention group

| | Description as stated in report/paper | Page number |
|---|---|---|
| **Group name** | Pharmacist-led diabetes management subgroup | |
| **No. randomised/assigned to group** *(specify whether no. people or clusters)* | 35 | 861 (figure 1) |
| **Description** *(include sufficient detail for replication, e.g. content, dose, components)* | Patients referred to the pharmacist were scheduled for an approximate 30-minute appointment (the PDTM intervention) on a separate day from the patient's visit with the endocrinologist. The endocrinologist determined the number of days between the initial endocrinologist visit and the first scheduled pharmacist visit at the time of referral based on each patient's unique factors. Subsequent follow-up PDTM appointments were determined by the pharmacist based on the patient's progress toward meeting his or her glycemic goals and the number of days until the patient's next visit with the endocrinologist. During the PDTM visit, the pharmacist, via written protocol with the endocrinologists of the clinic, could independently adjust (increase/decrease), substitute, or discontinue the patient's diabetic pharmacotherapy regimen as needed. As per the conditions of the collaborative practice agreement, the pharmacist could initiate a new diabetic agent after verbal consent from the endocrinologist. | 858 |
| **Duration of treatment period** | The mean+SD number of days between the pre- and post-HbA1c was 108 + 34 days. | 862 |
| **Timing** | The mean+SD number of days between the pre- and post-HbA1c was 108 + 34 days. | 862 |
| **Co-interventions** | Compliance, acquisition, dietary, and blood test result assessment | 858 |
| **Notes** | None | |

## Comparator group

| | Description as stated in report/paper | Page number |
|---|---|---|
| **Group name** | Standard care | 858 |
| **No. randomised/assigned to group** *(specify whether no. people or clusters)* | 86 individuals | 861 (figure 1) |

| | | |
|---|---|---|
| **Description** *(include sufficient detail for replication, e.g. content, dose, components)* | Standard care led by endocrinologist | 858 |
| **Duration of treatment period** | The mean+SD number of days between the pre- and post-HbA1c was 108 + 34 days. | 862 |
| **Timing** | The mean+SD number of days between the pre- and post-HbA1c was 108 + 34 days. | 862 |
| **Co-interventions** | NA | NA |
| **Notes** | None | |

## Outcomes

### Change in HbA1c pre- and postindex date

| | **Description as stated in report/paper** | | **Page number** |
|---|---|---|---|
| **Outcome name** | Change in HbA1c pre- and postindex date | | 858 |
| **Time points measured** *(specify whether from start or end of intervention)* | Change in HbA1c pre- and postindex date | | 858 |
| **Time points reported** | The mean+SD number of days between the pre- and post-HbA1c was 108 + 34 days. | | 862 |
| **Outcome definition** *(with diagnostic criteria if relevant)* | absolute change in HbA1c pre- and postindex date (HbA1c post _ HbA1c pre) was calculated for each patient. | | 858 |
| **Unit of measurement** *(if relevant)* | HgA1c levels (%) | | 858 |
| **Scales: upper and lower limits** *(indicate whether high or low score is good)* | Lower is desired | | NA |
| **Is outcome/tool validated?** | No | Bloodwork | NA |
| **Imputation of missing data** *(e.g. assumptions made for ITT analysis)* | NA | | NA |
| **Assumed risk estimate** *(e.g. baseline or population risk noted in Background)* | Not reported | | NA |
| **Power** *(e.g. power & sample size calculation, level of power achieved)* | Not calculated for subgroup analysis | | NA |

| | | |
|---|---|---|
| **Confounders** | Controlling for age, gender, ethnicity, years with diabetes, BMI, number of comorbidities, number of medications for diabetes treatment, baseline HbA1c values, and PDTM, RD, or LPC interventions during the study period. | 859 |
| **Notes** | None | |

## Funding/conflict of interest

| | | |
|---|---|---|
| **Study funding sources** *(including role of funders)* | The author(s) received no financial support for the research, authorship, and/or publication of this article. | 862 |
| **Possible conflicts of interest** *(for study authors)* | The author(s) declared no potential conflicts of interest with respect to the research, authorship, and/or publication of this article. | 862 |
| **Notes** | None | |

## Data and analysis

### Change in HbA1c pre- and postindex date

| | **Description as stated in report/paper** | | | | | | **Page number** |
|---|---|---|---|---|---|---|---|
| **Outcome** | Change in HbA1c pre- and postindex date | | | | | | 858 |
| **Time point** *(specify from start or end of intervention)* | Index (baseline); post-index ( mean+SD number of days between the pre- and post-HbA1c was 108 + 34 days) | | | | | | 862 |
| **Results** | Intervention | | | Comparison | | | 860 (table 4) |
| | Mean | SE *(or other variance, specify)* | No. participants | Mean | SE *(or other variance, specify)* | No. participants | |
| | -0.2 | 0.3 | 35 | REF | REF | 86 | |
| **Any other results reported** *(e.g. mean difference, CI, P value)* | p=0.39 | | | | | | 860 (table 4) |
| **No. missing participants** | 0 | | | | | | NA |

| | | |
|---|---|---|
| **Statistical methods used and appropriateness of these** *(e.g. adjustment for correlation)* | Patients included in the PDTM group were regrouped into four mutually exclusive groups: (1) patients who received PDTM interventions only; (2) patients who received PDTM and RD interventions only; (3) patients who received PDTM and LPC interventions only; and (4) patients who received PDTM, RD, and LPC interventions. The same regrouping was conducted for the SOC group: (1) patients with an endocrinologist encounter only (ie, no intervention); (2) patients with an endocrinologist encounter and an RD intervention only; (3) patients with an endocrinologist encounter and an LPC intervention only; and (4) patients with an endocrinologist encounter and an RD and LPC interventions. A GLM was used to compare the mean absolute change in HbA1c for each of the<br>seven groups with _1 intervention from a pharmacist, RD, or LPC to the group who received care from the endocrinologist only, while adjusting for age, gender, ethnicity, years with diabetes, BMI, number of comorbidities, number of medications for diabetes treatment, and baseline HbA1c values. SAS 9.4 was used to verify the distribution of residuals and to execute the GLM models for the primary and secondary an | 859 |
| **Notes** | None | |

## Conclusions

| | **Description as stated in report/paper** | **Page number** |
|---|---|---|
| **Key conclusions of study authors** | No significant difference between HgA1C mean change in PDTM and standard care groups. | 860 (table 4) |
| **Notes** | None | |

# Jameson et al. 2010

## Study eligibility

| **Study Characteristics** | **Eligibility criteria** |
|---|---|
| **Title** | Pharmacist Collaborative Management of Poorly Controlled Diabetes Mellitus: A Randomized Controlled Trial |
| **Author (year)** | Jameson et al. (2010) |
| **Country** | Michigan, USA |
| **Type of study** | RCT |
| **Participants** | Patients with diabetes |

| | |
|---|---|
| **Types of intervention** | Pharmacist management of diabetes |
| **Types of comparison** | Usual care |
| **Types of outcome measures** | HbA1C (reduction and patients who achieved at least a 1.0% decrease) |
| **Prescriptive authority** | Collaborative practice agreement |
| **Include/Exclude** | Include |
| **Notes** | None |

**DO NOT PROCEED IF STUDY EXCLUDED FROM REVIEW**

## Characteristics of included studies

### Methods

| | Descriptions as stated in report/paper | | Page number |
|---|---|---|---|
| **Aim of study** | To investigate the effect of pharmacist management of poorly controlled diabetes mellitus in a community-based primary care group. | | 250 |
| **Design** | Prospective randomised controlled trial | | 250 |
| **Unit of allocation** *(by individuals, cluster/ groups or body parts)* | Individuals | | 251 |
| **Start-end date** | May 2006-December 2007 | | 251 |
| **Duration of participation** *(from recruitment to last follow-up/ baseline to last follow-up- group level)* *1. Time of consent until last measurement for each individual.* *2. Baseline to final follow-up for each individual* | 12 months | | 251 |
| **Study duration** *(as above with the exception of interim analyses or other circumstances* | 12 months | | 251 |
| **Ethical approval needed/obtained for study** | Yes | The research committee and the institutional review board of Saint Mary's Health Care, Grand Rapids, Michigan, approved this study. | 251 |
| **Notes** | None | | |

### Participants

| | **Description**<br>*Include comparative information for each intervention or comparison group if available* | **Page number** |
|---|---|---|
| **Population description**<br>*(from which study participants are drawn)* | Patients with diabetes 18 years or older having A1C levels of 9.0% or higher or no office visits within 12 months were contacted by a study nurse. | 251 |
| **Setting**<br>*(including location and social context)* | The study site was the Advantage Health Physician Network (AHPN), which uses an electronic registry that identifies all adults with diabetes mellitus. Thirteen AHPN offices participated, including 3 urban, 9 suburban, and 1 rural site. Clinical. [primary care] | 251 |
| **Inclusion/exclusion criteria** | The nurse saw the patient at his or her home and determined study eligibility. Patients were excluded from the study if an endocrinologist was managing their diabetes or if they were not expected to live for the duration of the study. | 251 |
| **Method of recruitment of participants** | The study site was the Advantage Health Physician Network (AHPN), which uses an electronic registry that identifies all adults with diabetes mellitus. Thirteen AHPN offices participated, including 3 urban, 9 suburban, and 1 rural site. Clinical | 251 |
| **Informed consent obtained** | Yes<br>The study nurse obtained institutional review board–approved informed consent at this point from patients who agreed to participate and to attend all study visits. | 251 |
| **Total no. randomised**<br>*(or total pop. at start of study for NRCTs)* | 104 individuals | 252 |
| **Clusters** | NA | NA |
| **Baseline imbalances** | The only significant difference between study groups at baseline was lower A1C level in the intervention group. | 252 |
| **Withdrawals and exclusions** | Of these, 491 patients were successfully contacted and were invited to participate in the study. Two hundred thirty-five patients were excluded; 191(39.0%) declined participation, and 44 (9.0%) were managed by a specialist. An additional 152 (31.0%) had an A1C level of less than 9.0% at the time they saw the study nurse. The remaining 104 patients were randomized, 52 to the control group and 52 to the intervention group. | 251/252 |
| **Age (years)** | Intervention: 49.3 ± 10.8<br>Comparator: 49.7 ± 10.9 | 252 (table 1) |

| | | |
|---|---|---|
| **Sex (female)** | Intervention: 51.1%<br>Comparator: 51.0% | 252 (table 1) |
| **Subgroups measure** | Patients of white race/ethnicity; patients of non-white race/ethnicity; male patients | 253 (table 2) |
| **Subgroups reported** | Post hoc subgroup analysis showed that male patients in the intervention group achieved a statistically significant improvement in their A1C level (median, −1.90%; interquartile range, −0.05% to −2.95%) versus the control group (median, −0.15%; interquartile range, 0.98% to −1.38%]). | 252 |
| **Notes** | None | |

## Intervention group

| | Description as stated in report/paper | Page number |
|---|---|---|
| **Group name** | Pharmacist management of diabetes | 252 (table 1) |
| **No. randomised/assigned to group** *(specify whether no. people or clusters)* | 52 individuals | 252 (table 1) |
| **Description** *(include sufficient detail for replication, e.g. content, dose, components)* | Patients in the intervention group also met with the pharmacist at their respective primary care site for an assessment of adherence, barriers to optimizing blood glucose levels, and current medication regimen. All intervention patients received individualized education regarding diabetes self-management, including diet, exercise, blood glucose level testing, medications, and insulin. The pharmacist followed guidelines of the Management of Hyperglycemia in Type 2 Diabetes. This included early switching to insulin therapy after failure of 2 oral medications. The patient's primary care physician approved any changes in medication or therapy, although the pharmacist was given autonomy to adjust insulin doses as needed. The number of subsequent visits with the pharmacist was based on the need to further educate the patient about diabetes control or to monitor therapeutic changes. Follow-up visits were supplemented with telephone calls as needed for medication management. Patients were followed up for 12 months after study enrollment. | 251 |
| **Duration of treatment period** | 12 months | 251 |

| | | |
|---|---|---|
| **Timing** | The pharmacist averaged 6 office visits and 3 telephone calls per patient over the course of a year. Office visits lasted between 30 and 60 minutes. Telephone calls were 10 to 20 minutes in length | 252 |
| **Co-interventions** | Individualized education regarding diabetes self-management, including diet, exercise, blood glucose level testing, medications, and insulin. | 251 |
| **Notes** | None | |

## Comparator group

| | Description as stated in report/paper | Page number |
|---|---|---|
| **Group name** | Usual care | 252 (table 1) |
| **No. randomised/assigned to group** *(specify whether no. people or clusters)* | 52 individuals | 252 (table 1) |
| **Description** *(include sufficient detail for replication, e.g. content, dose, components)* | Usual care | 252 |
| **Duration of treatment period** | 12 months | 251 |
| **Timing** | NA | NA |
| **Co-interventions** | NA | NA |
| **Notes** | None | |

## Outcomes

### Reduction in HbA1C

| | Description as stated in report/paper | Page number |
|---|---|---|
| **Outcome name** | Reduction in HbA1C | 252 |
| **Time points measured** *(specify whether from start or end of intervention)* | Baseline; 12 months | 252 |
| **Time points reported** | 12 months | 252 |
| **Outcome definition** *(with diagnostic criteria if relevant)* | Reduction in HbA1C | 252 |
| **Unit of measurement** *(if relevant)* | % | 252 |

| | | | |
|---|---|---|---|
| **Scales: upper and lower limits** *(indicate whether high or low score is good)* | Lower is desired | | NA |
| **Is outcome/tool validated?** | No | Bloodwork | NA |
| **Imputation of missing data** *(e.g. assumptions made for ITT analysis)* | NA | | NA |
| **Assumed risk estimate** *(e.g. baseline or population risk noted in Background)* | Not reported | | NA |
| **Power** *(e.g. power & sample size calculation, level of power achieved)* | A power analysis indicated that at least 39 patients were needed in each study group to show a clinically significant decrease in A1C level of at least 1.0% ($\beta = 0.20$ and $\alpha = 0.05$). $\chi^2$ Test and Fisher exact test were used where appropriate for nominal data. Mann-Whitney test was used for nonparametric quantitative data and unpaired *t* test for parametric quantitative data. Significance was set at $P < .05$. The statistical software used was NCSS 2004 (Kaysville, UT). | | 251 |
| **Notes** | None | | |

## Patients who achieved at least a 1.0% decrease in HbA1C

| | **Description as stated in report/paper** | | **Page number** |
|---|---|---|---|
| **Outcome name** | Patients who achieved at least a 1.0% decrease in HbA1C | | 252 |
| **Time points measured** *(specify whether from start or end of intervention)* | Baseline; 12 months | | 252 |
| **Time points reported** | 12 months | | 252 |
| **Outcome definition** *(with diagnostic criteria if relevant)* | Patients who achieved at least a 1.0% decrease in HbA1C | | 252 |
| **Unit of measurement** *(if relevant)* | % | | 252 |
| **Scales: upper and lower limits** *(indicate whether high or low score is good)* | Higher is desired (more patients achieving a decrease) | | NA |
| **Is outcome/tool validated?** | No | Bloodwork | NA |

| | | |
|---|---|---|
| **Imputation of missing data** *(e.g. assumptions made for ITT analysis)* | NA | NA |
| **Assumed risk estimate** *(e.g. baseline or population risk noted in Background)* | Not reported | NA |
| **Power** *(e.g. power & sample size calculation, level of power achieved)* | A power analysis indicated that at least 39 patients were needed in each study group to show a clinically significant decrease in A1C level of at least 1.0% ($\beta = 0.20$ and $\alpha = 0.05$). $\chi 2$ Test and Fisher exact test were used where appropriate for nominal data. Mann-Whitney test was used for nonparametric quantitative data and unpaired *t* test for parametric quantitative data. Significance was set at $P < .05$. The statistical software used was NCSS 2004 (Kaysville, UT). | 251 |
| **Notes** | None | |

## Funding/conflict of interest

| | | |
|---|---|---|
| **Study funding sources** *(including role of funders)* | Financial support for this study was provided by Advantage Health Physician Network, Doran Foundation, Michigan Pharmacist Foundation, Priority Health, and Western Michigan Society of Health System Pharmacists. | 255 |
| **Possible conflicts of interest** *(for study authors)* | Dr Jameson reports no relationship or financial interest with any entity that would pose a conflict of interest with the subject matter of this article. Dr Baty is a practicing clinician in the Advantage Health Physician Network, the site where the study was conducted | 255 |
| **Notes** | None | |

## Data and analysis

### Reduction in HbA1C

| | **Description as stated in report/paper** | | | | | | **Page number** |
|---|---|---|---|---|---|---|---|
| **Outcome** | Reduction in HbA1C | | | | | | 253 |
| **Time point** *(specify from start or end of intervention)* | 12 months | | | | | | 253 |
| **Results** | Intervention | | | Comparison | | | 253 (table 2) |
| | Median reduction | IQR | No. participants | Median reduction | IQR | No. participants | |

| | | | | | | | |
|---|---|---|---|---|---|---|---|
| | −1.50 | −0.03, −2.68 | 52 | −0.40 | 0.50, −2.10 | 51 | |
| **Any other results reported** *(e.g. mean difference, CI, P value)* | p=0.06<br><br>The overall median A1C reduction in the intervention group was 1.1% greater than that of the control group. This difference approached but did not achieve statistical significance. | | | | | | 252 |
| **No. missing participants** | 1 individual | | | One patient in the control group was excluded as an outlier based on A1C level exceeding 3 SDs from the mean A1C change. | | | 252 |
| **Statistical methods used and appropriateness of these** *(e.g. adjustment for correlation)* | A power analysis indicated that at least 39 patients were needed in each study group to show a clinically significant decrease in A1C level of at least 1.0% ($\beta = 0.20$ and $\alpha = 0.05$). $\chi 2$ Test and Fisher exact test were used where appropriate for nominal data. Mann-Whitney test was used for nonparametric quantitative data and unpaired *t* test for parametric quantitative data. Significance was set at $P < .05$. The statistical software used was NCSS 2004 (Kaysville, UT). | | | | | | 251 |
| **Confounders** | Not controlled for | | | | | | 251 |
| **Notes** | Post hoc subgroup analysis showed that male patients in the intervention group achieved a statistically significant improvement in their A1C level (median, −1.90%; interquartile range, −0.05% to −2.95%) versus the control group (median, −0.15%; interquartile range, 0.98% to −1.38%]). Table 2 gives details of these results. | | | | | | |

## Patients who achieved at least a 1.0% decrease in HbA1C

| | **Description as stated in report/paper** | | | | **Page number** |
|---|---|---|---|---|---|
| **Outcome** | Patients who achieved at least a 1.0% decrease in HbA1C | | | | 253 |
| **Time point** *(specify from start or end of intervention)* | 12 months | | | | 253 |
| **Results** | Intervention | | Comparison | | 254 (table 3) |
| | No. with event | Total in group | No. with event | Total in group | |
| | 35 | 52 | 21 | 51 | |
| **Any other results reported** | p=0.02 | | | | |
| **No. missing participants** | 1 individual | One patient in the control group was excluded as an outlier based on A1C level exceeding 3 SDs from the mean A1C change. | | | 252 |

| | | |
|---|---|---|
| **Reasons missing** | A power analysis indicated that at least 39 patients were needed in each study group to show a clinically significant decrease in A1C level of at least 1.0% (β = 0.20 and α = 0.05). χ2 Test and Fisher exact test were used where appropriate for nominal data. Mann-Whitney test was used for nonparametric quantitative data and unpaired *t* test for parametric quantitative data. Significance was set at *P* <.05. The statistical software used was NCSS 2004 (Kaysville, UT). | 251 |
| **Statistical methods used and appropriateness of these** | Post hoc subgroup analysis showed that male patients in the intervention group achieved a statistically significant improvement in their A1C level (median, −1.90%; interquartile range, −0.05% to −2.95%) versus the control group (median, −0.15%; interquartile range, 0.98% to −1.38%]). Table 2 gives details of these results. | 251 |
| **Confounders** | Not controlled for | 251 |
| **Notes** | Many patients of nonwhite race/ethnicity and male patients (post hoc) in the intervention group exceeded the 1.0% improvement mark. No treatment effects using this measure were seen for patients of white race/ethnicity or for female patients 252 | |

## Conclusions

| | Description as stated in report/paper | Page number |
|---|---|---|
| **Key conclusions of study authors** | Patients with poorly controlled diabetes improved A1C levels significantly when pharmacist management was added to an aggressive organizational diabetes management program. Our results suggest that clinically trained pharmacists can help primary care providers improve diabetes management, especially among male patients and among patients of nonwhite race/ethnicity. | 250 |
| **Notes** | None | |

# Holland et al. 2023

## Study eligibility

| Study Characteristics | Eligibility criteria |
|---|---|
| **Title** | Evaluation of effectiveness and safety of pharmacist independent prescribers in care homes: cluster randomised controlled trial |
| **Author (year)** | Holland et al. 2023 |
| **Country** | East of England, Grampian (Scotland), Northern England, and Northern Ireland |

| | |
|---|---|
| **Type of study** | Cluster randomised control trial |
| **Participants** | People living in care homes |
| **Types of intervention** | Pharmacist prescribing |
| **Types of comparison** | Usual care (general practitioner prescribing) |
| **Types of outcome measures** | Fall rate/person at six months; quality of life (EQ-5D by proxy); Drug Burden Index; hospital admissions; and mortality. |
| **Prescriptive authority** | Independent |
| **Include/Exclude** | Include |
| **Notes** | None |

**DO NOT PROCEED IF STUDY EXCLUDED FROM REVIEW**

## Characteristics of included studies

### Methods

| | Descriptions as stated in report/paper | | Page number |
|---|---|---|---|
| **Aim of study** | To estimate the effectiveness, cost effectiveness (to be reported elsewhere), and safety of pharmacy independent prescribers in care homes. | | 1 |
| **Design** | Cluster randomised control trial | | 2 |
| **Unit of allocation** *(by individuals, cluster/ groups or body parts)* | Cluster | | 2 |
| **Start-end date** | March 2018-March 2020 | | 2 |
| **Duration of participation** *1. Time of consent until last measurement for each individual. 2. Baseline to final follow-up for each individual* | Baseline; six months | | 3 (figure 1) |
| **Study duration** *(as above with the exception of interim analyses or other circumstances* | Baseline; six months | | 3 (figure 1) |
| **Ethical approval needed/obtained for study** | Yes | Ethics approval was granted by the NHS East of England Central Cambridge Research Ethics Committee (for England and Northern Ireland) (reference 17/EE/0360) and by Scotland A REC (reference 17/SS/0118). | 8 |
| **Notes** | None | | |

### Participants

| | **Description**<br>*Include comparative information for each intervention or comparison group if available* | **Page number** |
|---|---|---|
| **Population description**<br>*(from which study participants are drawn)* | People who were under the care of a participating general practice, aged over 65 years, permanently resident in a participating care home | 2 |
| **Setting**<br>*(including location and social context)* | Care homes | 2 |
| **Inclusion/exclusion criteria** | We recruited triads (clusters) of a general practice, a pharmacist independent prescriber (PIP), and care home(s) providing approximately 20 residents each. All the PIPs needed to be UK accredited prescribers and were excluded if they already provided a similar service to the recruited care home or had a conflict of interest through employment with the supplying community pharmacy. We included general practices if they managed sufficient care home residents to support recruitment of 20 eligible participants. We included care homes if they provided care primarily to adults aged over 65 years and were associated with a participating general practice. We excluded them if their residents already received regular, drug focused review services (defined as monthly or more frequently) or if they were under formal investigation by a regulator. We included residents who were under the care of a participating general practice, aged over 65 years, permanently resident in a participating care home, taking at least one regular medicine, and able to provide (directly or via an appropriate representative) informed consent/assent. We excluded residents if they were receiving end-of-life care or participating in another study. | 2 |

| | | | |
|---|---|---|---|
| **Method of recruitment of participants** | We used invitation packs, containing invitation letters, information sheets, and consent forms, to recruit PIPs and general practices, identified using local networks. Consenting general practitioners then approached up to three care homes to enable recruitment of approximately 20 residents. Care home managers distributed invitation packs, signed by the general practitioner, to potential residents or appropriate third parties (for example, next of kin) for those residents lacking capacity to consent. | | 2 |
| **Informed consent obtained** | Yes | We used invitation packs, containing invitation letters, information sheets, and consent forms, | 20 |
| **Total no. randomised** *(or total pop. at start of study for NRCTs)* | 882 individuals | | 3 (figure 1) |
| **Clusters** | 49 clusters (triads of GP, PIP, and care homes) | | 2 |
| **Baseline imbalances** | Most variables were similar between groups, but the control group had rather more male residents (33% *v* 28%) and a greater proportion in nursing home care (59% *v* 42%). The intervention group had higher Barthel scores (8.34 *v* 7.07; ie, greater independence) and a greater rate of falls, with mean falls in the previous 90 days of 0.78 compared with 0.57 for controls. | | 6 |
| **Withdrawals and exclusions** | Almost all losses to follow-up at six months (137/168; 82%) were due to deaths of residents. Excluding those, primary outcome data were available for 96% of participants. | | 6 |
| **Age (years)** | Intervention: Mean 85.1 ± SD 7.7<br>Comparator: Mean 85.4 ± SD 7.6 | | 4 (table 1) |
| **Sex (female)** | Intervention: 72%<br>Comparator: 67% | | 4 (table 1) |
| **Subgroups measure** | Not reported | | NA |
| **Subgroups reported** | We used an analogous generalised estimating equation model for secondary and sensitivity analyses, with an appropriate change to the link and error term, depending on the nature of the outcome of interest. | | 5 |
| **Notes** | None | | |

## Intervention group

| | **Description as stated in report/paper** | **Page number** |
|---|---|---|
| **Group name** | Pharmacist prescribing | 3 (figure 1) |
| **No. randomised/assigned to group** *(specify whether no. people or clusters)* | 25 clusters; 454 individuals | 3 (figure 1) |

| | | |
|---|---|---|
| **Description** *(include sufficient detail for replication, e.g. content, dose, components)* | The PIPs visited the care homes to do medication reviews and optimise therapy for all participating residents, and they created pharmaceutical care plans to record their activity and provide a plan for future activity. Pharmaceutical care plans also allowed the PIP's actions/plans to be recorded for the care home and the resident's general practitioner. Additionally, the PIPs provided general support for improving care home processes for ordering medicines (to minimise opportunity for missed doses), administration of medicines (to reduce administration errors), medicines reconciliation when residents transferred between settings (to minimise opportunity for transcription errors), and staff training (to optimise requests for new medicines such as antipsychotics, laxatives, and analgesics). The nature and extent of delivery of each element of the intervention was individualised for the care home by the PIPs, each of whom was allocated four hours a week to manage an average of 20 residents over six months. | 3 |
| **Duration of treatment period** | Six months | NA |
| **Timing** | Not reported | NA |
| **Co-interventions** | Medication review, education | 3, 6 |
| **Notes** | None | |

## Comparator group

| | **Description as stated in report/paper** | **Page number** |
|---|---|---|
| **Group name** | Usual care | 3 (figure 1) |
| **No. randomised/assigned to group** *(specify whether no. people or clusters)* | 24 clusters; 428 individuals | 3 (figure 1) |

| | | |
|---|---|---|
| **Description** *(include sufficient detail for replication, e.g. content, dose, components)* | Participants in the control group received usual general practitioner led care, which could range from visits purely in response to individual requests to regular weekly sessions to provide more proactive care. Pharmacist provision could range from provision of medicines only (by a community pharmacist) to three, six, or 12 monthly visits by primary care based pharmacists doing medication reviews. Few, if any, of these reviews would have involved pharmacists actively prescribing, as opposed simply to providing advice to the general practitioner. PIPs recruited and trained within the trial had no interaction with control homes. | 3 |
| **Duration of treatment period** | Six months | |
| **Timing** | Not reported | NA |
| **Co-interventions** | Not reported | |
| **Notes** | None | |

## Outcomes

### Fall rate/person

| | Description as stated in report/paper | | Page number |
|---|---|---|---|
| **Outcome name** | Fall rate/person | | 4 (table 2) |
| **Time points measured** *(specify whether from start or end of intervention)* | Six months | | 4 (table 2) |
| **Time points reported** | Six months | | 4 (table 2) |
| **Outcome definition** *(with diagnostic criteria if relevant)* | Falls per person in six months | | NA |
| **Unit of measurement** *(if relevant)* | Event data | | 4 (table 2) |
| **Scales: upper and lower limits** *(indicate whether high or low score is good)* | Lower score desired | | 4 (table 2) |
| **Is outcome/tool validated?** | No | Care home falls record | 4 |

| | | |
|---|---|---|
| **Imputation of missing data** *(e.g. assumptions made for ITT analysis)* | • Analysed in their originally randomised groups<br>• Missing data addressed by including follow-up time as an offset<br>• Primary outcome model adjusted for baseline fall rate, drug burden index, Barthel Score, Charlson co-morbidity index and care home status<br>• GEE to account for clustered trial design<br>• Sample size calculations accounted for 20% attrition | 5 |
| **Assumed risk estimate** *(e.g. baseline or population risk noted in Background)* | Not reported | NA |
| **Power** *(e.g. power & sample size calculation, level of power achieved)* | We aimed for 880 participants (440/arm). This number was sufficient to provide 80% statistical power to detect a 21% difference in fall rate from 1.50 to 1.18 per resident over six months, using a two sided 5% significance level, and included an assumed attrition of 20%. | 5 |
| **Notes** | None | |

## Quality of life

| | **Description as stated in report/paper** | | **Page number** |
|---|---|---|---|
| **Outcome name** | Quality of life | | 4 |
| **Time points measured** *(specify whether from start or end of intervention)* | Six months | | 4 |
| **Time points reported** | Six months | | 4 |
| **Outcome definition** *(with diagnostic criteria if relevant)* | Quality of life | | NA |
| **Unit of measurement** *(if relevant)* | Utility score, with 0 indicating death and 1 full health | | 4 |
| **Scales: upper and lower limits** *(indicate whether high or low score is good)* | Higher score desired | | 4 |
| **Is outcome/tool validated?** | Yes | EQ-5D | 4 |
| **Imputation of missing data** *(e.g. assumptions made for ITT analysis)* | Intention to treat | | 5 |

| | | |
|---|---|---|
| **Assumed risk estimate** *(e.g. baseline or population risk noted in Background)* | Not reported | NA |
| **Power** *(e.g. power & sample size calculation, level of power achieved)* | We aimed for 880 participants (440/arm). This number was sufficient to provide 80% statistical power to detect a 21% difference in fall rate from 1.50 to 1.18 per resident over six months, using a two sided 5% significance level, and included an assumed attrition of 20%. | 5 |
| **Notes** | None | |

## Drug Burden Index

| | Description as stated in report/paper | | Page number |
|---|---|---|---|
| **Outcome name** | Drug burden index | | 4 |
| **Time points measured** *(specify whether from start or end of intervention)* | Six months | | 4 |
| **Time points reported** | Baseline; six months | | 4 |
| **Outcome definition** *(with diagnostic criteria if relevant)* | A measure of anticholinergic and sedative drug exposure, collected via medication data recorded by the general practitioner, | | 4 |
| **Unit of measurement** *(if relevant)* | Continuous | | NA |
| **Scales: upper and lower limits** *(indicate whether high or low score is good)* | Lower scores are desired | | NA |
| **Is outcome/tool validated?** | Yes | Drug Burden Index | NA |
| **Imputation of missing data** *(e.g. assumptions made for ITT analysis)* | Intention to treat | | 5 |
| **Assumed risk estimate** *(e.g. baseline or population risk noted in Background)* | Not reported | | NA |
| **Power** *(e.g. power & sample size calculation, level of power achieved)* | We aimed for 880 participants (440/arm). This number was sufficient to provide 80% statistical power to detect a 21% difference in fall rate from 1.50 to 1.18 per resident over six months, using a two sided 5% significance level, and included an assumed attrition of 20%. | | 5 |
| **Notes** | None | | |

## Hospital admissions

| | Description as stated in report/paper | | Page number |
|---|---|---|---|
| **Outcome name** | Hospital admissions over six months | | 4 |
| **Time points measured** *(specify whether from start or end of intervention)* | Six months | | 4 |
| **Time points reported** | Six months | | 4 |
| **Outcome definition** *(with diagnostic criteria if relevant)* | Unexpected hospital admissions over six months | | 4 |
| **Unit of measurement** *(if relevant)* | Event data | | NA |
| **Scales: upper and lower limits** *(indicate whether high or low score is good)* | Lower scores are desired | | NA |
| **Is outcome/tool validated?** | No | General practice records supplemented by care home records | 4 |
| **Imputation of missing data** *(e.g. assumptions made for ITT analysis)* | Intention to treat | | 5 |
| **Assumed risk estimate** *(e.g. baseline or population risk noted in Background)* | Not reported | | NA |
| **Power** *(e.g. power & sample size calculation, level of power achieved)* | We aimed for 880 participants (440/arm). This number was sufficient to provide 80% statistical power to detect a 21% difference in fall rate from 1.50 to 1.18 per resident over six months, using a two sided 5% significance level, and included an assumed attrition of 20%. | | 5 |
| **Notes** | None | | |

## Mortality

| | Description as stated in report/paper | Page number |
|---|---|---|
| **Outcome name** | Mortality | 4 |
| **Time points measured** *(specify whether from start or end of intervention)* | Six months | 4 |
| **Time points reported** | Six months | 4 |

| | | | |
|---|---|---|---|
| **Outcome definition** *(with diagnostic criteria if relevant)* | Time from consent to death | | 5 |
| **Unit of measurement** *(if relevant)* | Event data | | NA |
| **Scales: upper and lower limits** *(indicate whether high or low score is good)* | Lower scores are desired | | NA |
| **Is outcome/tool validated?** | No | GP updates | NA |
| **Imputation of missing data** *(e.g. assumptions made for ITT analysis)* | Intention to treat | | 5 |
| **Assumed risk estimate** *(e.g. baseline or population risk noted in Background)* | Not reported | | NA |
| **Power** *(e.g. power & sample size calculation, level of power achieved)* | We aimed for 880 participants (440/arm). This number was sufficient to provide 80% statistical power to detect a 21% difference in fall rate from 1.50 to 1.18 per resident over six months, using a two sided 5% significance level, and included an assumed attrition of 20%. | | 5 |
| **Notes** | None | | |

## Funding/conflict of interest

| | | |
|---|---|---|
| **Study funding sources** *(including role of funders)* | This work was funded by National Institutes of Health Research (NIHR) through their Programme Grant for Applied Research (PGfAR) stream (RP-PG-0613-20007). The funder had no role in design, data collection, data analysis, data interpretation, or writing of this paper. | 8 |
| **Possible conflicts of interest** *(for study authors)* | DW has received speaker fees from Desitin Pharma and speaker fees and unrestricted education grants from Rosemont Pharmaceuticals; CB has received personal fees as editor in chief of the International Journal of Pharmacy Practice, during the conduct of the study; CH was a commissioned member of the NIHR HS&DR Panel (2015-19); GB was a member of a CTU funded by NIHR (to 2021); LS was a member of the NIHR EME Funding Committee (2010-14); AA was a commissioned member of the NIHR HS&DR Board (2014-16); AZ was a member of the NIHR HTA MPOH Pharmaceuticals Panel (2011- 18); no other relationships or activities that could appear to have influenced the submitted work. | 8 |

| | |
|---|---|
| **Notes** | None |

## Data and analysis

### Fall rate/person

| | Description as stated in report/paper | | | | Page number |
|---|---|---|---|---|---|
| **Outcome** | Fall rate/person | | | | 4 (table 2) |
| **Time point** *(specify from start or end of intervention)* | Six months | | | | 4 (table 2) |
| **Results** | Intervention | | Comparison | | 4 (table 2) |
| | No. of events | Total in group | No. of events | Total in group | |
| | 697 | 449 | 538 | 427 | |
| **Any other results reported** | Rate ratio (95%CI); p-value: 0.91 (0.66 to 1.26); 0.58<br>1.55 per resident in the intervention group.<br>1.26 per resident in the control group | | | | 4 (table 2), 6 |
| **No. missing participants** | Not reported per arm | | Not reported per arm | | NA |
| **Reasons missing** | Adjusted model 1 included 844 participants | | Full adjusted model (2) included 812 participants | | 4 (table 2) |
| **Statistical methods used and appropriateness of these** | The primary analysis was done on an intention-to-treat basis (that is, participants were analysed within their allocated group, rather than by actual treatment received), with a per protocol analysis also completed for participants deemed to have received the PIP intervention as intended. We anticipated that the primary outcome would follow a Poisson distribution, but the data proved to best fit a negative binomial model, which we used instead. We estimated parameters by using a generalised estimating equation approach, to account for the clustered design, with an offset included for length of follow-up. Length of follow-up varied from participant to participant owing to death or dropout. The final primary outcome model included baseline fall rate, key prognostic variables (defined as baseline values of Drug Burden Index,28 Barthel score,27 Charlson Comorbidity Index,23 and home status (nursing/residential)), with group as a fixed factor. We also included an offset of logarithm of follow-up time to allow inclusion of information from participants lost to follow-up before six months. We used an analogous generalised estimating equation model for secondary and sensitivity analyses, with an appropriate change to the link and error term, depending on the nature of the outcome of interest. | | | | 5 |
| **Notes** | None | | | | |

## Quality of life

| | Description as stated in report/paper | | | | | | Page number |
|---|---|---|---|---|---|---|---|
| **Outcome** | Quality of life (EQ-5D) | | | | | | 5 (table 4) |
| **Time point** *(specify from start or end of intervention)* | Six months | | | | | | 5 (table 4) |
| **Results** | Intervention | | | Comparison | | | 5 (table 4) |
| | Mean | SD *(or other variance, specify)* | No. participants | Mean | SD *(or other variance, specify)* | No. participants | |
| | 0.26 | 0.35 | 449 | 0.21 | 0.33 | 427 | |
| **Any other results reported** *(e.g. mean difference, CI, P value)* | Absolute difference (95% CI ); p-value: 0.042 (−0.043 to 0.052); 0.86 | | | | | | 5 (table 4) |
| **No. missing participants** | 53 | | | 47 | | | 5 (table 4) |
| **Statistical methods used and appropriateness of these** *(e.g. adjustment for correlation)* | We used an analogous generalised estimating equation model for secondary and sensitivity analyses, with an appropriate change to the link and error term, depending on the nature of the outcome of interest. | | | | | | 5 |
| **Notes** | None | | | | | | |

## Drug burden index

| | Description as stated in report/paper | | | | | | Page number |
|---|---|---|---|---|---|---|---|
| **Outcome** | Drug Burden Index | | | | | | 5 (table 3) |
| **Time point** *(specify from start or end of intervention)* | Six months | | | | | | 5 (table 3) |
| **Results** | Intervention | | | Comparison | | | 5 (table 3) |
| | Mean | SD *(or other variance, specify)* | No. participants | Mean | SD *(or other variance, specify)* | No. participants | |
| | 0.66 | 0.74 | 449 | 0.73 | 0.69 | 427 | |

| | | | |
|---|---|---|---|
| **Any other results reported** *(e.g. mean difference, CI, P value)* | Rate ratio (95%CI); p-value: 0.83 (0.74 to 0.92); <0.001 | | 5 (table 3) |
| **No. missing participants** | 10 | 9 | NA |
| **Statistical methods used and appropriateness of these** *(e.g. adjustment for correlation)* | We used an analogous generalised estimating equation model for secondary and sensitivity analyses, with an appropriate change to the link and error term, depending on the nature of the outcome of interest. | | 5 |
| **Notes** | None | | |

## Hospital admissions

| | **Description as stated in report/paper** | | | | | | **Page number** |
|---|---|---|---|---|---|---|---|
| **Outcome** | Hospital admissions | | | | | | 5 (table 3) |
| **Time point** *(specify from start or end of intervention)* | Six months | | | | | | 5 (table 3) |
| **Results** | Intervention | | | Comparison | | | 5 (table 3) |
| | Mean | SD *(or other variance, specify)* | No. participants | Mean | SD *(or other variance, specify)* | No. participants | |
| | 0.19 | 0.5 | 449 | 0.18 | 0.47 | 427 | |
| **Any other results reported** *(e.g. mean difference, CI, P value)* | Rate ratio (95%CI); p-value: 0.90 (0.61 to 1.32); 0.57 | | | | | | 5 (table 3) |
| **No. missing participants** | 10 | | | 9 | | | NA |
| **Statistical methods used and appropriateness of these** *(e.g. adjustment for correlation)* | We used an analogous generalised estimating equation model for secondary and sensitivity analyses, with an appropriate change to the link and error term, depending on the nature of the outcome of interest. | | | | | | 5 |

| | |
|---|---|
| **Notes** | None |

## Mortality

| | Description as stated in report/paper | | | | Page number |
|---|---|---|---|---|---|
| **Outcome** | Mortality | | | | 6 |
| **Time point** *(specify from start or end of intervention)* | Six months | | | | 6 |
| **Results** | Intervention | | Comparison | | 6 |
| | No. with event | Total in group | No. with event | Total in group | |
| | 66 | 449 | 71 | 427 | |
| **Any other results reported** | Adjusted hazard ratio (95%CI); p-value: 0.93 (0.64 to 1.35); 0.68<br>Mean time to death of 109 and 103 days respectively (int v. control) | | | | 6 |
| **No. missing participants** | NA | | NA | | NA |
| **Reasons missing** | NA | | NA | | NA |
| **Statistical methods used and appropriateness of these** | We used a Cox proportional hazards regression model (time from consent to death or otherwise censored) for mortality analyses, in which we used. robust sandwich estimates of standard errors to adjust for clustering within care homes. We used SAS v.9.4 for analyses. | | | | 5/6 |
| **Notes** | None | | | | |

## Conclusions

| | Description as stated in report/paper | Page number |
|---|---|---|
| **Key conclusions of study authors** | This large, rigorously conducted, cluster randomised controlled trial, testing a pharmacist independent prescriber regularly visiting care homes to manage residents' pharmaceutical care, showed that this was a safe, well received intervention,31 which decreased anticholinergic/sedative prescribing. Although this would be expected to realise future clinical benefits, the intervention showed no improvement in our primary outcome of falls. | 8 |
| **Notes** | None | |

# Lau et al. 2020

## Study eligibility

| Study Characteristics | Eligibility criteria |
|---|---|

| | |
|---|---|
| **Title** | Telemonitoring and protocolized case management for hypertensive community dwelling older adults (TECHNOMED): a randomized controlled trial |
| **Author (year)** | Lau et al. 2022 |
| **Country** | Alberta, Canada |
| **Type of study** | Open label RCT |
| **Participants** | Community dwelling older people |
| **Types of intervention** | Home BP telemonitoring (HBPM) and pharmacist-led case management |
| **Types of comparison** | Enhanced usual care with HBPM alone |
| **Types of outcome measures** | Proportion achieving systolic BP targets on 24-h ambulatory BP monitoring; health-related quality of life; depression; generalised anxiety; hypotension (SBP < 110 mmHg), non-mechanical falls; syncope; orthostatic BP changes (defined as >20mmHg fall in SBP, >10mmHg fall in DBP, or >30 BPM increase in heart rate from supine to sitting/standing AOBP measurement); orthostatic presyncope; electrolyte disturbances (serum potassium < 3.3 mmol/l, potassium > 5.0 mmol/l, and sodium < 130 mmol/l) |
| **Prescriptive authority** | Protocol |
| **Include/Exclude** | Include |
| **Notes** | None |

**DO NOT PROCEED IF STUDY EXCLUDED FROM REVIEW**

## Characteristics of included studies

### Methods

| | Descriptions as stated in report/paper | | Page number |
|---|---|---|---|
| **Aim of study** | To compare the efficacy and safety of combining HBPM telemonitoring and protocolized case management, vs. Enhanced usual care with HBPM only, in older, community-dwelling adults. | | 1703 |
| **Design** | Open label RCT | | 1703 |
| **Unit of allocation** *(by individuals, cluster/ groups or body parts)* | Individuals | | 1703 |
| **Start-end date** | September 2016-April 2019 | | 1703 |
| **Duration of participation** *(from recruitment to last follow-up/ baseline to last follow-up- group level)* *1. Time of consent until last measurement for each individual.* *2. Baseline to final follow-up for each individual* | 12 months | | 1703 |
| **Study duration** *(as above with the exception of interim analyses or other circumstances* | 12 | | 1703 |
| **Ethical approval needed/obtained for study** | Yes | University of Alberta Health Research Ethics Board approval was obtained prior to study initiation and all patients provided written informed consent. | 1703 |
| **Notes** | None | | |

### Participants

| | Description *Include comparative information for each intervention or comparison group if available* | Page number |
|---|---|---|

| | | | |
|---|---|---|---|
| **Population description** *(from which study participants are drawn)* | Older adults living in supportive living residences. These participants were independent in basic activities of daily living and lived in separate apartments, though some received help with instrumental activities of daily living such as meal preparation and housecleaning. | | 1703 |
| **Setting** *(including location and social context)* | Supportive living residences | | 1703 |
| **Inclusion/exclusion criteria** | We included older adults (age > 65 years) with a diagnosis of hypertension, determined by self-report and documented prescription of an anti-hypertensive medication. Exclusion criteria were extreme hypertension (SBP > 220mmHg or diastolic BP [DBP] > 110mmHg), inability to communicate in English, heart failure with reduced ejection fraction (because BP targets may be less important than achieving guideline-recommended dosing in these patients), limited life expectancy (<12 months), cognitive impairment (Short Portable Mental Status Questionnaire score > 5), inability to perform HBPM, severe depression (Patient Health Questionnaire-8 [PHQ-8] score > 15), concurrent receipt of pharmacy case management for cardiovascular risk factor control, or concurrent participation in a cardiovascular trial. | | 1703 |
| **Method of recruitment of participants** | Participants were recruited from 29 supportive living residences, with five residences contributing 75% of individuals. | | 1705 |
| **Informed consent obtained** | Yes | University of Alberta Health Research Ethics Board approval was obtained prior to study initiation and all patients provided written informed consent. | 1703 |
| **Total no. randomised** *(or total pop. at start of study for NRCTs)* | 120 | | 1705 (figure 1) |
| **Clusters** | NA | | NA |
| **Baseline imbalances** | There were fewer females randomized to the intervention (69%) than to the control (85%) arms. | | 1705 |
| **Withdrawals and exclusions** | The remaining 120 participants were randomized, 61 to the intervention, and 59 to enhanced usual care. During the study, 14 participants from each group withdrew early (23% overall). Reasons for withdrawal were similar between groups. Follow-up was curtailed by the COVID-19 pandemic in five participants. At the end of the study, 92 participants remained – 47 in the intervention arm, 45 in the control arm. Seven of these participants (three in the intervention arm, four in the control arm) declined final 24-h ABPM measurements, despite completing other outcome measures. | | 1704 |

| | | |
|---|---|---|
| **Age (years)** | Intervention: 79.8 ± 7.7<br>Comparator: 79.2 ± 7.4 | 1706 (table 1) |
| **Sex (female)** | Intervention: 69%<br>Comparator: 85% | 1706 (table 1) |
| **Subgroups measure** | Uncontrolled BP; aged >80 years | 1707 |
| **Subgroups reported** | At baseline, 56 participants (48%) had uncontrolled BP by ABPM. In this subgroup, intervention participants (n=30) appeared more likely than control participants (n=26) to meet BP targets at 12 months on ABPM (RD +34% [+5% to +64%], P=0.034, favoring the intervention; unadjusted OR 6.00 [0.70–51.10], P=0.101) as well as on other BP modalities (HBPM +29%, _1% to +58%]; AOBP +15%, [-14% to +45%]).<br><br>Sixty-four participants were age >80. In this subgroup, substantial mean BP changes were observed (e.g., mean HBPM difference _11.0/_4.3mmHg, P¼0.003 [SBP] and 0.015 [DBP], unadjusted) in directions similar to those of the total study sample. | 1707 |
| **Notes** | None | |

## Intervention group

| | Description as stated in report/paper | Page number |
|---|---|---|
| **Group name** | Pharmacist-led case management | 1703 |
| **No. randomised/assigned to group** *(specify whether no. people or clusters)* | 61 individuals | 1705 (figure 1) |

| | | |
|---|---|---|
| **Description** *(include sufficient detail for replication, e.g. content, dose, components)* | The intervention included home BP telemonitoring and protocolized, pharmacist-led case management. For HBPM, all participants received a validated electronic upper arm oscillometric BP monitor with wireless transfer capabilities.<br><br>Participants were instructed to perform home BP series consisting of duplicate measurements in the morning and evening for 7 consecutive days. For each 7-day HBPM series, the first day's measurements were discarded, and the mean of subsequent measurements calculated and used to guide medication titration. At minimum, all participants were instructed to perform 7-day series every 3 months.<br><br>Pharmacist CMs employed by a third party were trained by physicians from the University of Alberta Hypertension Clinic to administer behavioural counselling, teach BP self-monitoring, and encourage medication adherence; and to review telemonitored BP summaries, remind participants to complete HBPM series, and adjust medications according to protocol | 1703 |
| **Duration of treatment period** | 12 months | 1703 |
| **Timing** | Participants were instructed to perform home BP series consisting of duplicate measurements in the morning and evening for 7 consecutive days. For each 7-day HBPM series, the first day's measurements were discarded, and the mean of subsequent measurements calculated and used to guide medication titration. At minimum, all participants were instructed to perform 7-day series every 3 months | 1703 |
| **Co-interventions** | Behavioural counselling, teach BP self-monitoring, and encourage medication adherence; and to review telemonitored BP summaries, remind participants to complete HBPM series, | 1703 |
| **Notes** | None | |

## Comparator group

| | Description as stated in report/paper | Page number |
|---|---|---|
| **Group name** | Enhanced usual care | 1703 |

| | | |
|---|---|---|
| **No. randomised/assigned to group** *(specify whether no. people or clusters)* | 59 individuals | 1705 (figure 1) |
| **Description** *(include sufficient detail for replication, e.g. content, dose, components)* | Participants in the enhanced usual care arm received HBPM equipment and training only. HBPM readings were tele-transmitted for study purposes but were not made available to patients or case managers, except if necessary for safety reasons, at the discretion of the data safety monitoring physician (J.R.). Enhanced usual care participants were encouraged to perform 7-day HBPM series to review at visits with their existing provider every 3 months. | 1703/1704 |
| **Duration of treatment period** | 12 months | 1703 |
| **Timing** | Enhanced usual care participants were encouraged to perform 7-day HBPM series to review at visits with their existing provider every 3 months. | 1704 |
| **Co-interventions** | Not reported | NA |
| **Notes** | None | |

## Outcomes

### Proportion achieving systolic BP targets on 24-h ambulatory BP monitoring

| | **Description as stated in report/paper** | **Page number** |
|---|---|---|
| **Outcome name** | Proportion achieving systolic BP targets on 24-h ambulatory BP monitoring | 1704 |
| **Time points measured** *(specify whether from start or end of intervention)* | Baseline; 12 months | 1704 |
| **Time points reported** | 12 months | 1704 |

| | | | |
|---|---|---|---|
| **Outcome definition** *(with diagnostic criteria if relevant)* | Proportion achieving systolic BP targets on 24-h ambulatory BP monitoring (ABPM)<br><br>age-specific BP targets, on ABPM, at 12 months: SBP 110 and <130mmHg (age 65–79 years), and SBP 110 and <140mmHg (age 80 years).<br><br>An ABPM average SBP <130mmHg is approximately equivalent to the office BP of <140mmHg recommended in contemporaneous and current Hypertension Canada guidelines [10].<br><br>The target of <140mmHg in those of age >=80 years was considered equivalent to the office BP threshold of <150mmHg for older, frailer adults recommended by European Society of Hypertension Clinical Practice Guidelines and Canadian Hypertension Education Program guidelines at the time of study initiation [28,29]. | | 1704 |
| **Unit of measurement** *(if relevant)* | % | | 1704 |
| **Scales: upper and lower limits** *(indicate whether high or low score is good)* | Higher proportion is desired | | NA |
| **Is outcome/tool validated?** | No | BP monitor | 1703 |
| **Imputation of missing data** *(e.g. assumptions made for ITT analysis)* | Not reported | | NA |
| **Assumed risk estimate** *(e.g. baseline or population risk noted in Background)* | Not reported | | NA |
| **Power** *(e.g. power & sample size calculation, level of power achieved)* | A priori, recruitment of 100 and retention of 80 participants per arm was planned to detect a relative 40% increase in the proportion of participants in target BP range with 80% power. | | 1704 |
| **Notes** | None | | |

## Health related quality of life

| | Description as stated in report/paper | Page number |
|---|---|---|
| **Outcome name** | Health related quality of life | 1704 |

| | | | |
|---|---|---|---|
| **Time points measured** *(specify whether from start or end of intervention)* | Baseline; 12 months | | 1704 |
| **Time points reported** | 12 months | | 1704 |
| **Outcome definition** *(with diagnostic criteria if relevant)* | Health related quality of life | | 1709 |
| **Unit of measurement** *(if relevant)* | Mean change | | 1709 |
| **Scales: upper and lower limits** *(indicate whether high or low score is good)* | Higher score is desired | | NA |
| **Is outcome/tool validated?** | Yes | EQ-5D | 1709 |
| **Imputation of missing data** *(e.g. assumptions made for ITT analysis)* | Not reported | | NA |
| **Assumed risk estimate** *(e.g. baseline or population risk noted in Background)* | Not reported | | NA |
| **Power** *(e.g. power & sample size calculation, level of power achieved)* | A priori, recruitment of 100 and retention of 80 participants per arm was planned to detect a relative 40% increase in the proportion of participants in target BP range with 80% power. | | 1704 |
| **Notes** | None | | |

## Depression

| | Description as stated in report/paper | Page number |
|---|---|---|
| **Outcome name** | Depression | 1704 |
| **Time points measured** *(specify whether from start or end of intervention)* | Baseline; 12 months | 1704 |
| **Time points reported** | 12 months | 1704 |
| **Outcome definition** *(with diagnostic criteria if relevant)* | Depression | 1704 |
| **Unit of measurement** *(if relevant)* | Mean change | 1704 |

| | | | |
|---|---|---|---|
| **Scales: upper and lower limits** *(indicate whether high or low score is good)* | Lower score but higher positive mean change desired | | NA |
| **Is outcome/tool validated?** | Yes | PHQ-8 | 1709 |
| **Imputation of missing data** *(e.g. assumptions made for ITT analysis)* | Not reported | | NA |
| **Assumed risk estimate** *(e.g. baseline or population risk noted in Background)* | Not reported | | NA |
| **Power** *(e.g. power & sample size calculation, level of power achieved)* | A priori, recruitment of 100 and retention of 80 participants per arm was planned to detect a relative 40% increase in the proportion of participants in target BP range with 80% power. | | 1704 |
| **Notes** | None | | |

## Anxiety

| | Description as stated in report/paper | | Page number |
|---|---|---|---|
| **Outcome name** | Anxiety | | 1704 |
| **Time points measured** *(specify whether from start or end of intervention)* | Baseline; 12 months | | 1704 |
| **Time points reported** | 12 months | | 1704 |
| **Outcome definition** *(with diagnostic criteria if relevant)* | Anxiety | | 1704 |
| **Unit of measurement** *(if relevant)* | Mean change | | 1704 |
| **Scales: upper and lower limits** *(indicate whether high or low score is good)* | Lower score but higher positive mean change desired | | NA |
| **Is outcome/tool validated?** | Yes | GAD | 1703 |
| **Imputation of missing data** *(e.g. assumptions made for ITT analysis)* | Not reported | | NA |

| | | |
|---|---|---|
| **Assumed risk estimate** *(e.g. baseline or population risk noted in Background)* | Not reported | NA |
| **Power** *(e.g. power & sample size calculation, level of power achieved)* | A priori, recruitment of 100 and retention of 80 participants per arm was planned to detect a relative 40% increase in the proportion of participants in target BP range with 80% power. | 1704 |
| **Notes** | None | |

### Fall requiring medication attention

| | Description as stated in report/paper | | Page number |
|---|---|---|---|
| **Outcome name** | Fall requiring medical attention | | 1704 |
| **Time points measured** *(specify whether from start or end of intervention)* | Baseline; 12 months | | 1704 |
| **Time points reported** | 12 months | | 1704 |
| **Outcome definition** *(with diagnostic criteria if relevant)* | Syncope | | 1710 |
| **Unit of measurement** *(if relevant)* | Events | | 1710 |
| **Scales: upper and lower limits** *(indicate whether high or low score is good)* | Lower score is desired | | NA |
| **Is outcome/tool validated?** | No | Self-report, review of provincial electronic health care records | 1704 |
| **Imputation of missing data** *(e.g. assumptions made for ITT analysis)* | Not reported | | NA |
| **Assumed risk estimate** *(e.g. baseline or population risk noted in Background)* | Not reported | | NA |
| **Power** *(e.g. power & sample size calculation, level of power achieved)* | A priori, recruitment of 100 and retention of 80 participants per arm was planned to detect a relative 40% increase in the proportion of participants in target BP range with 80% power. | | 1704 |
| **Notes** | None | | |

### Syncope

| | Description as stated in report/paper | | Page number |
|---|---|---|---|
| **Outcome name** | Syncope | | 1704 |
| **Time points measured** *(specify whether from start or end of intervention)* | Baseline; 12 months | | 1704 |
| **Time points reported** | 12 months | | 1704 |
| **Outcome definition** *(with diagnostic criteria if relevant)* | Syncope | | 1710 |
| **Unit of measurement** *(if relevant)* | Events | | 1710 |
| **Scales: upper and lower limits** *(indicate whether high or low score is good)* | Lower score is desired | | NA |
| **Is outcome/tool validated?** | No | Self-report, review of provincial electronic health care records | 1704 |
| **Imputation of missing data** *(e.g. assumptions made for ITT analysis)* | Not reported | | NA |
| **Assumed risk estimate** *(e.g. baseline or population risk noted in Background)* | Not reported | | NA |
| **Power** *(e.g. power & sample size calculation, level of power achieved)* | A priori, recruitment of 100 and retention of 80 participants per arm was planned to detect a relative 40% increase in the proportion of participants in target BP range with 80% power. | | 1704 |
| **Notes** | None | | |

## Hypotension

| | Description as stated in report/paper | Page number |
|---|---|---|
| **Outcome name** | Hypotension | 1704 |
| **Time points measured** *(specify whether from start or end of intervention)* | Baseline; 12 months | 1704 |
| **Time points reported** | 12 months | 1704 |
| **Outcome definition** *(with diagnostic criteria if relevant)* | Hypotension | 1710 |

| | | | |
|---|---|---|---|
| **Unit of measurement** *(if relevant)* | Events | | 1710 |
| **Scales: upper and lower limits** *(indicate whether high or low score is good)* | Lower score is desired | | NA |
| **Is outcome/tool validated?** | No | Self-report, review of provincial electronic health care records | 1704 |
| **Imputation of missing data** *(e.g. assumptions made for ITT analysis)* | Not reported | | NA |
| **Assumed risk estimate** *(e.g. baseline or population risk noted in Background)* | Not reported | | NA |
| **Power** *(e.g. power & sample size calculation, level of power achieved)* | A priori, recruitment of 100 and retention of 80 participants per arm was planned to detect a relative 40% increase in the proportion of participants in target BP range with 80% power. | | 1704 |
| **Notes** | None | | |

## Hypokalemia

| | **Description as stated in report/paper** | | **Page number** |
|---|---|---|---|
| **Outcome name** | Hypokalemia | | 1704 |
| **Time points measured** *(specify whether from start or end of intervention)* | Baseline; 12 months | | 1704 |
| **Time points reported** | 12 months | | 1704 |
| **Outcome definition** *(with diagnostic criteria if relevant)* | Hypokalemia | | 1710 |
| **Unit of measurement** *(if relevant)* | Events | | 1710 |
| **Scales: upper and lower limits** *(indicate whether high or low score is good)* | Lower score is desired | | NA |
| **Is outcome/tool validated?** | No | Self-report, review of provincial electronic health care records | 1704 |

| | | |
|---|---|---|
| **Imputation of missing data** *(e.g. assumptions made for ITT analysis)* | Not reported | NA |
| **Assumed risk estimate** *(e.g. baseline or population risk noted in Background)* | Not reported | NA |
| **Power** *(e.g. power & sample size calculation, level of power achieved)* | A priori, recruitment of 100 and retention of 80 participants per arm was planned to detect a relative 40% increase in the proportion of participants in target BP range with 80% power. | 1704 |
| **Notes** | None | |

## Hyperkalemia

| | Description as stated in report/paper | | Page number |
|---|---|---|---|
| **Outcome name** | Hyperkalemia | | 1704 |
| **Time points measured** *(specify whether from start or end of intervention)* | Baseline; 12 months | | 1704 |
| **Time points reported** | 12 months | | 1704 |
| **Outcome definition** *(with diagnostic criteria if relevant)* | Hyperkalemia | | 1710 |
| **Unit of measurement** *(if relevant)* | Events | | 1710 |
| **Scales: upper and lower limits** *(indicate whether high or low score is good)* | Lower score is desired | | NA |
| **Is outcome/tool validated?** | No | Self-report, review of provincial electronic health care records | 1704 |
| **Imputation of missing data** *(e.g. assumptions made for ITT analysis)* | Not reported | | NA |
| **Assumed risk estimate** *(e.g. baseline or population risk noted in Background)* | Not reported | | NA |

| | | |
|---|---|---|
| **Power** *(e.g. power & sample size calculation, level of power achieved)* | A priori, recruitment of 100 and retention of 80 participants per arm was planned to detect a relative 40% increase in the proportion of participants in target BP range with 80% power. | 1704 |
| **Notes** | None | |

## Hyponatremia

| | Description as stated in report/paper | | Page number |
|---|---|---|---|
| **Outcome name** | Hyponatremia | | 1704 |
| **Time points measured** *(specify whether from start or end of intervention)* | Baseline; 12 months | | 1704 |
| **Time points reported** | 12 months | | 1704 |
| **Outcome definition** *(with diagnostic criteria if relevant)* | Hyponatremia | | 1710 |
| **Unit of measurement** *(if relevant)* | Events | | 1710 |
| **Scales: upper and lower limits** *(indicate whether high or low score is good)* | Lower score is desired | | NA |
| **Is outcome/tool validated?** | No | Self-report, review of provincial electronic health care records | 1704 |
| **Imputation of missing data** *(e.g. assumptions made for ITT analysis)* | Not reported | | NA |
| **Assumed risk estimate** *(e.g. baseline or population risk noted in Background)* | Not reported | | NA |
| **Power** *(e.g. power & sample size calculation, level of power achieved)* | A priori, recruitment of 100 and retention of 80 participants per arm was planned to detect a relative 40% increase in the proportion of participants in target BP range with 80% power. | | 1704 |
| **Notes** | None | | |

## Orthostatic presyncope

| | Description as stated in report/paper | Page number |
|---|---|---|
| **Outcome name** | Orthostatic presyncope | 1704 |

| | | | |
|---|---|---|---|
| **Time points measured** *(specify whether from start or end of intervention)* | Baseline; 12 months | | 1704 |
| **Time points reported** | 12 months | | 1704 |
| **Outcome definition** *(with diagnostic criteria if relevant)* | Orthostatic presyncope | | 1710 |
| **Unit of measurement** *(if relevant)* | Events | | 1710 |
| **Scales: upper and lower limits** *(indicate whether high or low score is good)* | Lower score is desired | | NA |
| **Is outcome/tool validated?** | No | Self-report, review of provincial electronic health care records | 1704 |
| **Imputation of missing data** *(e.g. assumptions made for ITT analysis)* | Not reported | | NA |
| **Assumed risk estimate** *(e.g. baseline or population risk noted in Background)* | Not reported | | NA |
| **Power** *(e.g. power & sample size calculation, level of power achieved)* | A priori, recruitment of 100 and retention of 80 participants per arm was planned to detect a relative 40% increase in the proportion of participants in target BP range with 80% power. | | 1704 |
| **Notes** | None | | |

## Change in eGFR

| | Description as stated in report/paper | Page number |
|---|---|---|
| **Outcome name** | Change in eGFR | 1704 |
| **Time points measured** *(specify whether from start or end of intervention)* | Baseline; 12 months | 1704 |
| **Time points reported** | 12 months | 1704 |
| **Outcome definition** *(with diagnostic criteria if relevant)* | Estimated glomerular filtration rate | 1710 |
| **Unit of measurement** *(if relevant)* | ml/min/1.73 $m^2$ | 1710 |

| | | | |
|---|---|---|---|
| **Scales: upper and lower limits** *(indicate whether high or low score is good)* | Range not specified | | NA |
| **Is outcome/tool validated?** | No | Self-report, review of provincial electronic health care records | 1704 |
| **Imputation of missing data** *(e.g. assumptions made for ITT analysis)* | Not reported | | NA |
| **Assumed risk estimate** *(e.g. baseline or population risk noted in Background)* | Not reported | | NA |
| **Power** *(e.g. power & sample size calculation, level of power achieved)* | A priori, recruitment of 100 and retention of 80 participants per arm was planned to detect a relative 40% increase in the proportion of participants in target BP range with 80% power. | | 1704 |
| **Notes** | None | | |

## ED admissions

| | Description as stated in report/paper | | Page number |
|---|---|---|---|
| **Outcome name** | ED admissions | | 1704 |
| **Time points measured** *(specify whether from start or end of intervention)* | Baseline; 12 months | | 1704 |
| **Time points reported** | 12 months | | 1704 |
| **Outcome definition** *(with diagnostic criteria if relevant)* | Admissions to emergency department | | 1710 |
| **Unit of measurement** *(if relevant)* | Mean (SD) | | 1710 |
| **Scales: upper and lower limits** *(indicate whether high or low score is good)* | Lower score is desired | | NA |
| **Is outcome/tool validated?** | No | Self-report, review of provincial electronic health care records | 1704 |
| **Imputation of missing data** *(e.g. assumptions made for ITT analysis)* | Not reported | | NA |

| | | |
|---|---|---|
| **Assumed risk estimate** *(e.g. baseline or population risk noted in Background)* | Not reported | NA |
| **Power** *(e.g. power & sample size calculation, level of power achieved)* | A priori, recruitment of 100 and retention of 80 participants per arm was planned to detect a relative 40% increase in the proportion of participants in target BP range with 80% power. | 1704 |
| **Notes** | None | |

## Hospitalisations

| | Description as stated in report/paper | | Page number |
|---|---|---|---|
| **Outcome name** | Hospitalisations | | 1704 |
| **Time points measured** *(specify whether from start or end of intervention)* | Baseline; 12 months | | 1704 |
| **Time points reported** | 12 months | | 1704 |
| **Outcome definition** *(with diagnostic criteria if relevant)* | Hospitalisations | | 1710 |
| **Unit of measurement** *(if relevant)* | Mean (SD) | | 1710 |
| **Scales: upper and lower limits** *(indicate whether high or low score is good)* | Lower score is desired | | NA |
| **Is outcome/tool validated?** | No | Self-report, review of provincial electronic health care records | 1704 |
| **Imputation of missing data** *(e.g. assumptions made for ITT analysis)* | Not reported | | NA |
| **Assumed risk estimate** *(e.g. baseline or population risk noted in Background)* | Not reported | | NA |
| **Power** *(e.g. power & sample size calculation, level of power achieved)* | A priori, recruitment of 100 and retention of 80 participants per arm was planned to detect a relative 40% increase in the proportion of participants in target BP range with 80% power. | | 1704 |
| **Notes** | None | | |

## Funding/conflict of interest

| | | |
|---|---|---|
| **Study funding sources** *(including role of funders)* | TECHNOMED is funded by Canadian Institute for Health Research (CIHR) grant number EH2-143571 and Alberta Innovates Health Solutions grant number 201900506. | 1711 |
| **Possible conflicts of interest** *(for study authors)* | Disclosures: R.P. is the Chief Executive Officer, and J.R. is the ChiefMedical Officer of mmHg Inc., a provider of digital health, software solutions, including remote patient monitoring. S.K. is the Director of the Real World Evidence Consortium at the University of Alberta that conducts investigator initiated industry sponsored research. | 1711 |
| **Notes** | None | |

## Data and analysis

### Proportion achieving systolic BP targets on 24-h ambulatory BP monitoring

| | **Description as stated in report/paper** | | | | **Page number** |
|---|---|---|---|---|---|
| **Outcome** | Proportion achieving systolic BP targets on 24-h ambulatory BP monitoring | | | | 1704 |
| **Time point** *(specify from start or end of intervention)* | Baseline; 12 months | | | | 1704 |
| **Results** | Intervention | | Comparison | | 1707 (table 2) |
| | No. with event | Total in group | No. with event | Total in group | |
| | 31 | 47 | 25 | 45 | |
| **Any other results reported** | OR (95% CI): 1.48 (0.87, 2.52) p=0.153 | | | | 1707 (table 2) |
| **No. missing participants** | 14 | | 14 | | 1705 (table 1) |
| **Reasons missing** | Declined to continue (n=8); deceased (n=1); lost to follow-up (n=1); institutionalised (n=0); COVID-related (n=4) | | Declined to continue (n=7); deceased (n=2); lost to follow-up (n=2); institutionalised (n=2); COVID-related (n=1) | | 1705 (table 1) |

| | | |
|---|---|---|
| **Statistical methods used and appropriateness of these** | with adjustment for age, sex, and baseline achievement of SBP targets using logistic regression. Mean changes in BP and additional outcomes were compared using similarly parameterized logistic and linear regression models. Clustering within residences was accounted for in the 24-h ABPM outcomes using generalized estimating equations specifying exchangeable within-group correlations and robust variance estimators. Post-hoc analyses were performed in subgroups of only participants with uncontrolled BP at baseline, and in participants aged >80 years. Statistical adjustment by regression was not possible in subgroup analyses due to low numbers. Safety endpoints were compared between randomization arms using chi-squared tests. All estimates were considered statistically significant at P<0.05. No attempt was made to adjust for multiple comparisons. | 1704 |
| **Notes** | None | |

## Health related quality of life

| | Description as stated in report/paper | | | | | | Page number |
|---|---|---|---|---|---|---|---|
| **Outcome** | Health related quality of life | | | | | | 1704 |
| **Time point** *(specify from start or end of intervention)* | Baseline; 12 months | | | | | | 1704 |
| **Results** | Intervention | | | Comparison | | | 1709 (table 3) |
| | Mean change | SD *(or other variance, specify)* | No. participants | Mean change | SD *(or other variance, specify)* | No. participants | |
| | -0.0 | 0.2 | 47 | -0.0 | 0.1 | 45 | |
| **Any other results reported** *(e.g. mean difference, CI, P value)* | Intervention vs. control difference in changes over time (95% CI): 0.0 (0.0, 0.1)<br>p=0.547 | | | | | | 1709 (table 3) |
| **No. missing participants** | 14 | | | 14 | | | 1705 (table 1) |
| **Reasons missing** | Declined to continue (n=8); deceased (n=1); lost to follow-up (n=1); institutionalised (n=0); COVID-related (n=4) | | | Declined to continue (n=7); deceased (n=2); lost to follow-up (n=2); institutionalised (n=2); COVID-related (n=1) | | | 1705 (table 1) |

| | | |
|---|---|---|
| **Statistical methods used and appropriateness of these** *(e.g. adjustment for correlation)* | with adjustment for age, sex, and baseline achievement of SBP targets using logistic regression. Mean changes in BP and additional outcomes were compared using similarly parameterized logistic and linear regression models. Clustering within residences was accounted for in the 24-h ABPM outcomes using generalized estimating equations specifying exchangeable within-group correlations and robust variance estimators. Post-hoc analyses were performed in subgroups of only participants with uncontrolled BP at baseline, and in participants aged >=80 years. Statistical adjustment by regression was not possible in subgroup analyses due to low numbers. Safety endpoints were compared between randomization arms using chi-squared tests. All estimates were considered statistically significant at P<0.05. No attempt was made to adjust for multiple comparisons. | 1704 |
| **Notes** | None | |

## Depression

| | Description as stated in report/paper | | | | | | Page number |
|---|---|---|---|---|---|---|---|
| **Outcome** | Depression | | | | | | 1704 |
| **Time point** *(specify from start or end of intervention)* | Baseline; 12 months | | | | | | 1704 |
| **Results** | Intervention | | | Comparison | | | 1709 (table 3) |
| | Mean change | SD *(or other variance, specify)* | No. participants | Mean change | SD *(or other variance, specify)* | No. participants | |
| | 1.0 | 3.8 | 47 | 1.2 | 3.1 | 45 | |
| **Any other results reported** *(e.g. mean difference, CI, P value)* | Intervention vs. control difference in changes over time (95% CI): 0.2 (-1.7, 1.2)<br>p=0.924 | | | | | | 1709 (table 3) |
| **No. missing participants** | 14 | | | 14 | | | 1705 (table 1) |
| **Reasons missing** | Declined to continue (n=8); deceased (n=1); lost to follow-up (n=1); institutionalised (n=0); COVID-related (n=4) | | | Declined to continue (n=7); deceased (n=2); lost to follow-up (n=2); institutionalised (n=2); COVID-related (n=1) | | | 1705 (table 1) |

| | | |
|---|---|---|
| **Statistical methods used and appropriateness of these** *(e.g. adjustment for correlation)* | with adjustment for age, sex, and baseline achievement of SBP targets using logistic regression. Mean changes in BP and additional outcomes were compared using similarly parameterized logistic and linear regression models. Clustering within residences was accounted for in the 24-h ABPM outcomes using generalized estimating equations specifying exchangeable within-group correlations and robust variance estimators. Post-hoc analyses were performed in subgroups of only participants with uncontrolled BP at baseline, and in participants aged >=80 years. Statistical adjustment by regression was not possible in subgroup analyses due to low numbers. Safety endpoints were compared between randomization arms using chi-squared tests. All estimates were considered statistically significant at P<0.05. No attempt was made to adjust for multiple comparisons. | 1704 |
| **Notes** | None | |

## Anxiety

| | Description as stated in report/paper | | | | | | Page number |
|---|---|---|---|---|---|---|---|
| **Outcome** | Anxiety | | | | | | 1704 |
| **Time point** *(specify from start or end of intervention)* | Baseline; 12 months | | | | | | 1704 |
| **Results** | Intervention | | | Comparison | | | 1709 (table 3) |
| | Mean change | SD *(or other variance, specify)* | No. participants | Mean change | SD *(or other variance, specify)* | No. participants | |
| | -0.4 | 1.8 | 47 | 0.1 | 1.4 | 45 | |
| **Any other results reported** *(e.g. mean difference, CI, P value)* | Intervention vs. control difference in changes over time (95% CI): -0.4 (-1.1, 0.2)<br>p=0.430 | | | | | | 1709 (table 3) |
| **No. missing participants** | 14 | | | 14 | | | 1705 (table 1) |
| **Reasons missing** | Declined to continue (n=8); deceased (n=1); lost to follow-up (n=1); institutionalised (n=0); COVID-related (n=4) | | | Declined to continue (n=7); deceased (n=2); lost to follow-up (n=2); institutionalised (n=2); COVID-related (n=1) | | | 1705 (table 1) |

| | | |
|---|---|---|
| **Statistical methods used and appropriateness of these** *(e.g. adjustment for correlation)* | with adjustment for age, sex, and baseline achievement of SBP targets using logistic regression. Mean changes in BP and additional outcomes were compared using similarly parameterized logistic and linear regression models. Clustering within residences was accounted for in the 24-h ABPM outcomes using generalized estimating equations specifying exchangeable within-group correlations and robust variance estimators. Post-hoc analyses were performed in subgroups of only participants with uncontrolled BP at baseline, and in participants aged >=80 years. Statistical adjustment by regression was not possible in subgroup analyses due to low numbers. Safety endpoints were compared between randomization arms using chi-squared tests. All estimates were considered statistically significant at P<0.05. No attempt was made to adjust for multiple comparisons. | 1704 |
| **Notes** | None | |

## Fall requiring medical attention

| | **Description as stated in report/paper** | | | | **Page number** |
|---|---|---|---|---|---|
| **Outcome** | Fall requiring medical attention | | | | 1704 |
| **Time point** *(specify from start or end of intervention)* | Baseline; 12 months | | | | 1704 |
| **Results** | Intervention | | Comparison | | 1710 (table 6) |
| | No. with event | Total in group | No. with event | Total in group | |
| | 9 | 47 | 9 | 45 | |
| **Any other results reported** | p=0.939 | | | | 1710 (table 6) |
| **No. missing participants** | 14 | | 14 | | 1705 (table 1) |
| **Reasons missing** | Declined to continue (n=8); deceased (n=1); lost to follow-up (n=1); institutionalised (n=0); COVID-related (n=4) | | Declined to continue (n=7); deceased (n=2); lost to follow-up (n=2); institutionalised (n=2); COVID-related (n=1) | | 1705 (table 1) |

| | | |
|---|---|---|
| **Statistical methods used and appropriateness of these** | with adjustment for age, sex, and baseline achievement of SBP targets using logistic regression. Mean changes in BP and additional outcomes were compared using similarly parameterized logistic and linear regression models. Clustering within residences was accounted for in the 24-h ABPM outcomes using generalized estimating equations specifying exchangeable within-group correlations and robust variance estimators. Post-hoc analyses were performed in subgroups of only participants with uncontrolled BP at baseline, and in participants aged >=80 years. Statistical adjustment by regression was not possible in subgroup analyses due to low numbers. Safety endpoints were compared between randomization arms using chi-squared tests. All estimates were considered statistically significant at P<0.05. No attempt was made to adjust for multiple comparisons. | 1704 |
| **Notes** | None | |

## Syncope

| | Description as stated in report/paper | | | | Page number |
|---|---|---|---|---|---|
| **Outcome** | Syncope | | | | 1704 |
| **Time point** *(specify from start or end of intervention)* | Baseline; 12 months | | | | 1704 |
| **Results** | Intervention | | Comparison | | 1710 (table 6) |
| | No. with event | Total in group | No. with event | Total in group | |
| | 11 | 47 | 9 | 45 | |
| **Any other results reported** | p=0.683 | | | | 1710 (table 6) |
| **No. missing participants** | 14 | | 14 | | 1705 (table 1) |
| **Reasons missing** | Declined to continue (n=8); deceased (n=1); lost to follow-up (n=1); institutionalised (n=0); COVID-related (n=4) | | Declined to continue (n=7); deceased (n=2); lost to follow-up (n=2); institutionalised (n=2); COVID-related (n=1) | | 1705 (table 1) |

| | | |
|---|---|---|
| **Statistical methods used and appropriateness of these** | with adjustment for age, sex, and baseline achievement of SBP targets using logistic regression. Mean changes in BP and additional outcomes were compared using similarly parameterized logistic and linear regression models. Clustering within residences was accounted for in the 24-h ABPM outcomes using generalized estimating equations specifying exchangeable within-group correlations and robust variance estimators. Post-hoc analyses were performed in subgroups of only participants with uncontrolled BP at baseline, and in participants aged >=80 years. Statistical adjustment by regression was not possible in subgroup analyses due to low numbers. Safety endpoints were compared between randomization arms using chi-squared tests. All estimates were considered statistically significant at P<0.05. No attempt was made to adjust for multiple comparisons. | 1704 |
| **Notes** | None | |

## Hypotension

| | **Description as stated in report/paper** | | | | **Page number** |
|---|---|---|---|---|---|
| **Outcome** | Hypotension | | | | 1704 |
| **Time point** *(specify from start or end of intervention)* | Baseline; 12 months | | | | 1704 |
| **Results** | Intervention | | Comparison | | 1710 (table 6) |
| | No. with event | Total in group | No. with event | Total in group | |
| | 13 | 47 | 3 | 45 | |
| **Any other results reported** | p=0.009 | | | | 1710 (table 6) |
| **No. missing participants** | 14 | | 14 | | 1705 (table 1) |
| **Reasons missing** | Declined to continue (n=8); deceased (n=1); lost to follow-up (n=1); institutionalised (n=0); COVID-related (n=4) | | Declined to continue (n=7); deceased (n=2); lost to follow-up (n=2); institutionalised (n=2); COVID-related (n=1) | | 1705 (table 1) |

| | | |
|---|---|---|
| **Statistical methods used and appropriateness of these** | with adjustment for age, sex, and baseline achievement of SBP targets using logistic regression. Mean changes in BP and additional outcomes were compared using similarly parameterized logistic and linear regression models. Clustering within residences was accounted for in the 24-h ABPM outcomes using generalized estimating equations specifying exchangeable within-group correlations and robust variance estimators. Post-hoc analyses were performed in subgroups of only participants with uncontrolled BP at baseline, and in participants aged >=80 years. Statistical adjustment by regression was not possible in subgroup analyses due to low numbers. Safety endpoints were compared between randomization arms using chi-squared tests. All estimates were considered statistically significant at P<0.05. No attempt was made to adjust for multiple comparisons. | 1704 |
| **Notes** | None | |

## Hypokalemia

| | **Description as stated in report/paper** | | | | **Page number** |
|---|---|---|---|---|---|
| **Outcome** | Hypokalemia | | | | 1704 |
| **Time point** *(specify from start or end of intervention)* | Baseline; 12 months | | | | 1704 |
| **Results** | Intervention | | Comparison | | 1710 (table 6) |
| | No. with event | Total in group | No. with event | Total in group | |
| | 3 | 47 | 5 | 45 | |
| **Any other results reported** | p=0.435 | | | | 1710 (table 6) |
| **No. missing participants** | 14 | | 14 | | 1705 (table 1) |
| **Reasons missing** | Declined to continue (n=8); deceased (n=1); lost to follow-up (n=1); institutionalised (n=0); COVID-related (n=4) | | Declined to continue (n=7); deceased (n=2); lost to follow-up (n=2); institutionalised (n=2); COVID-related (n=1) | | 1705 (table 1) |

| | | |
|---|---|---|
| **Statistical methods used and appropriateness of these** | with adjustment for age, sex, and baseline achievement of SBP targets using logistic regression. Mean changes in BP and additional outcomes were compared using similarly parameterized logistic and linear regression models. Clustering within residences was accounted for in the 24-h ABPM outcomes using generalized estimating equations specifying exchangeable within-group correlations and robust variance estimators. Post-hoc analyses were performed in subgroups of only participants with uncontrolled BP at baseline, and in participants aged >=80 years. Statistical adjustment by regression was not possible in subgroup analyses due to low numbers. Safety endpoints were compared between randomization arms using chi-squared tests. All estimates were considered statistically significant at P<0.05. No attempt was made to adjust for multiple comparisons. | 1704 |
| **Notes** | None | |

## Hyperkalemia

| | **Description as stated in report/paper** | | | | **Page number** |
|---|---|---|---|---|---|
| **Outcome** | Hyperkalemia | | | | 1704 |
| **Time point** *(specify from start or end of intervention)* | Baseline; 12 months | | | | 1704 |
| **Results** | Intervention | | Comparison | | 1710 (table 6) |
| | No. with event | Total in group | No. with event | Total in group | |
| | 6 | 47 | 4 | 45 | |
| **Any other results reported** | p=0.545 | | | | 1710 (table 6) |
| **No. missing participants** | 14 | | 14 | | 1705 (table 1) |
| **Reasons missing** | Declined to continue (n=8); deceased (n=1); lost to follow-up (n=1); institutionalised (n=0); COVID-related (n=4) | | Declined to continue (n=7); deceased (n=2); lost to follow-up (n=2); institutionalised (n=2); COVID-related (n=1) | | 1705 (table 1) |

| | | |
|---|---|---|
| **Statistical methods used and appropriateness of these** | with adjustment for age, sex, and baseline achievement of SBP targets using logistic regression. Mean changes in BP and additional outcomes were compared using similarly parameterized logistic and linear regression models. Clustering within residences was accounted for in the 24-h ABPM outcomes using generalized estimating equations specifying exchangeable within-group correlations and robust variance estimators. Post-hoc analyses were performed in subgroups of only participants with uncontrolled BP at baseline, and in participants aged >=80 years. Statistical adjustment by regression was not possible in subgroup analyses due to low numbers. Safety endpoints were compared between randomization arms using chi-squared tests. All estimates were considered statistically significant at P<0.05. No attempt was made to adjust for multiple comparisons. | 1704 |
| **Notes** | None | |

## Hyponatremia

| | Description as stated in report/paper | | | | Page number |
|---|---|---|---|---|---|
| **Outcome** | Hyponatremia | | | | 1704 |
| **Time point** *(specify from start or end of intervention)* | Baseline; 12 months | | | | 1704 |
| **Results** | Intervention | | Comparison | | 1710 (table 6) |
| | No. with event | Total in group | No. with event | Total in group | |
| | 1 | 47 | 2 | 45 | |
| **Any other results reported** | p=0.539 | | | | 1710 (table 6) |
| **No. missing participants** | 14 | | 14 | | 1705 (table 1) |
| **Reasons missing** | Declined to continue (n=8); deceased (n=1); lost to follow-up (n=1); institutionalised (n=0); COVID-related (n=4) | | Declined to continue (n=7); deceased (n=2); lost to follow-up (n=2); institutionalised (n=2); COVID-related (n=1) | | 1705 (table 1) |

| | | |
|---|---|---|
| **Statistical methods used and appropriateness of these** | with adjustment for age, sex, and baseline achievement of SBP targets using logistic regression. Mean changes in BP and additional outcomes were compared using similarly parameterized logistic and linear regression models. Clustering within residences was accounted for in the 24-h ABPM outcomes using generalized estimating equations specifying exchangeable within-group correlations and robust variance estimators. Post-hoc analyses were performed in subgroups of only participants with uncontrolled BP at baseline, and in participants aged >=80 years. Statistical adjustment by regression was not possible in subgroup analyses due to low numbers. Safety endpoints were compared between randomization arms using chi-squared tests. All estimates were considered statistically significant at P<0.05. No attempt was made to adjust for multiple comparisons. | 1704 |
| **Notes** | None | |

## Orthostatic presyncope

| | Description as stated in report/paper | | | | Page number |
|---|---|---|---|---|---|
| **Outcome** | Orthostatic presyncope | | | | 1704 |
| **Time point** *(specify from start or end of intervention)* | Baseline; 12 months | | | | 1704 |
| **Results** | Intervention | | Comparison | | 1710 (table 6) |
| | No. with event | Total in group | No. with event | Total in group | |
| | 7 | 47 | 5 | 45 | |
| **Any other results reported** | p=0.590 | | | | 1710 (table 6) |
| **No. missing participants** | 14 | | 14 | | 1705 (table 1) |
| **Reasons missing** | Declined to continue (n=8); deceased (n=1); lost to follow-up (n=1); institutionalised (n=0); COVID-related (n=4) | | Declined to continue (n=7); deceased (n=2); lost to follow-up (n=2); institutionalised (n=2); COVID-related (n=1) | | 1705 (table 1) |

| | | |
|---|---|---|
| **Statistical methods used and appropriateness of these** | with adjustment for age, sex, and baseline achievement of SBP targets using logistic regression. Mean changes in BP and additional outcomes were compared using similarly parameterized logistic and linear regression models. Clustering within residences was accounted for in the 24-h ABPM outcomes using generalized estimating equations specifying exchangeable within-group correlations and robust variance estimators. Post-hoc analyses were performed in subgroups of only participants with uncontrolled BP at baseline, and in participants aged _80 years. Statistical adjustment by regression was not possible in subgroup analyses due to low numbers. Safety endpoints were compared between randomization arms using chi-squared tests. All estimates were considered statistically significant at P<0.05. No attempt was made to adjust for multiple comparisons. | 1704 |
| **Notes** | None | |

## Change in eGFR

| | **Description as stated in report/paper** | | | | | | **Page number** |
|---|---|---|---|---|---|---|---|
| **Outcome** | Change in eGFR | | | | | | 1704 |
| **Time point** *(specify from start or end of intervention)* | Baseline; 12 months | | | | | | 1704 |
| **Results** | Intervention | | | Comparison | | | 1710 (table 6) |
| | Mean change | SD *(or other variance, specify)* | No. participants | Mean change | SD *(or other variance, specify)* | No. participants | |
| | -2.68 | 8.75 | 47 | -1.05 | 9.14 | 45 | |
| **Any other results reported** *(e.g. mean difference, CI, P value)* | p=0.381 | | | | | | 1710 (table 6) |
| **No. missing participants** | 14 | | | 14 | | | 1705 (table 1) |
| **Reasons missing** | Declined to continue (n=8); deceased (n=1); lost to follow-up (n=1); institutionalised (n=0); COVID-related (n=4) | | | Declined to continue (n=7); deceased (n=2); lost to follow-up (n=2); institutionalised (n=2); COVID-related (n=1) | | | 1705 (table 1) |

| | | |
|---|---|---|
| **Statistical methods used and appropriateness of these** *(e.g. adjustment for correlation)* | with adjustment for age, sex, and baseline achievement of SBP targets using logistic regression. Mean changes in BP and additional outcomes were compared using similarly parameterized logistic and linear regression models. Clustering within residences was accounted for in the 24-h ABPM outcomes using generalized estimating equations specifying exchangeable within-group correlations and robust variance estimators. Post-hoc analyses were performed in subgroups of only participants with uncontrolled BP at baseline, and in participants aged _80 years. Statistical adjustment by regression was not possible in subgroup analyses due to low numbers. Safety endpoints were compared between randomization arms using chi-squared tests. All estimates were considered statistically significant at P<0.05. No attempt was made to adjust for multiple comparisons. | 1704 |
| **Notes** | None | |

## ED admissions

| | **Description as stated in report/paper** | | | | | | **Page number** |
|---|---|---|---|---|---|---|---|
| **Outcome** | ED admissions | | | | | | 1704 |
| **Time point** *(specify from start or end of intervention)* | Baseline; 12 months | | | | | | 1704 |
| **Results** | Intervention | | | Comparison | | | 1710 (table 6) |
| | Mean | SD *(or other variance, specify)* | No. participants | Mean | SD *(or other variance, specify)* | No. participants | |
| | 0.59 | 1.19 | 47 | 0.39 | 0.77 | 45 | |
| **Any other results reported** *(e.g. mean difference, CI, P value)* | p=0.276 | | | | | | 1710 (table 6) |
| **No. missing participants** | 14 | | | 14 | | | 1705 (table 1) |
| **Reasons missing** | Declined to continue (n=8); deceased (n=1); lost to follow-up (n=1); institutionalised (n=0); COVID-related (n=4) | | | Declined to continue (n=7); deceased (n=2); lost to follow-up (n=2); institutionalised (n=2); COVID-related (n=1) | | | 1705 (table 1) |

| | | |
|---|---|---|
| **Statistical methods used and appropriateness of these** *(e.g. adjustment for correlation)* | with adjustment for age, sex, and baseline achievement of SBP targets using logistic regression. Mean changes in BP and additional outcomes were compared using similarly parameterized logistic and linear regression models. Clustering within residences was accounted for in the 24-h ABPM outcomes using generalized estimating equations specifying exchangeable within-group correlations and robust variance estimators. Post-hoc analyses were performed in subgroups of only participants with uncontrolled BP at baseline, and in participants aged _80 years. Statistical adjustment by regression was not possible in subgroup analyses due to low numbers. Safety endpoints were compared between randomization arms using chi-squared tests. All estimates were considered statistically significant at P<0.05. No attempt was made to adjust for multiple comparisons. | 1704 |
| **Notes** | None | |

## Hospitalisations

| | Description as stated in report/paper | | | | | | Page number |
|---|---|---|---|---|---|---|---|
| **Outcome** | Hospitalisations | | | | | | 1704 |
| **Time point** *(specify from start or end of intervention)* | Baseline; 12 months | | | | | | 1704 |
| **Results** | Intervention | | | Comparison | | | 1710 (table 6) |
| | Mean change | SD *(or other variance, specify)* | No. participants | Mean change | SD *(or other variance, specify)* | No. participants | |
| | 0.31 | 0.70 | 47 | 0.24 | 0.73 | 45 | |
| **Any other results reported** *(e.g. mean difference, CI, P value)* | p=0.569 | | | | | | 1710 (table 6) |
| **No. missing participants** | 14 | | | 14 | | | 1705 (table 1) |
| **Reasons missing** | Declined to continue (n=8); deceased (n=1); lost to follow-up (n=1); institutionalised (n=0); COVID-related (n=4) | | | Declined to continue (n=7); deceased (n=2); lost to follow-up (n=2); institutionalised (n=2); COVID-related (n=1) | | | 1705 (table 1) |

| | | |
|---|---|---|
| **Statistical methods used and appropriateness of these** *(e.g. adjustment for correlation)* | with adjustment for age, sex, and baseline achievement of SBP targets using logistic regression. Mean changes in BP and additional outcomes were compared using similarly parameterized logistic and linear regression models. Clustering within residences was accounted for in the 24-h ABPM outcomes using generalized estimating equations specifying exchangeable within-group correlations and robust variance estimators. Post-hoc analyses were performed in subgroups of only participants with uncontrolled BP at baseline, and in participants aged _80 years. Statistical adjustment by regression was not possible in subgroup analyses due to low numbers. Safety endpoints were compared between randomization arms using chi-squared tests. All estimates were considered statistically significant at P<0.05. No attempt was made to adjust for multiple comparisons. | 1704 |
| **Notes** | None | |

## Conclusions

| | **Description as stated in report/paper** | **Page number** |
|---|---|---|
| **Key conclusions of study authors** | We did not detect a difference in the proportion of participants with at-target BPs and this null result may have been influenced by small numbers of participants, high degree of baseline BP control, and non-adherence to HBPM. While there was no difference in mean ABPM between groups, the mean change in HBPM was improved in intervention participants to a statistically and clinically significant degree. Though intervention participants had a higher rate of SBP <110 mmHg, a very conservative definition of hypotension, there was no increase in non-mechanical falls, orthostatic symptoms, or acute care utilization. | 1707 |
| **Notes** | None | |

# Lum et al. 2023

## Study eligibility

| **Study Characteristics** | **Eligibility criteria** |
|---|---|
| **Title** | Clinical activities that contributed to the effectiveness of a cardiologist–pharmacist collaborative care model in managing diabetes |
| **Author (year)** | Lum et al. 2023 |
| **Country** | Singapore |
| **Type of study** | Retrospective cohort study |
| **Participants** | Individuals aged 21 years and above, diagnosed with Type 2 diabetes were included in this study |
| **Types of intervention** | Pharmacist managed |

| | |
|---|---|
| **Types of comparison** | Cardiologist managed |
| **Types of outcome measures** | Changes in mean HbA1c over 12 months |
| **Prescriptive authority** | Collaborative practice agreement |
| **Include/Exclude** | Include |
| **Notes** | None |

**DO NOT PROCEED IF STUDY EXCLUDED FROM REVIEW**

## Characteristics of included studies

### Methods

| | Descriptions as stated in report/paper | | Page number |
|---|---|---|---|
| **Aim of study** | To evaluate the changes in mean HbA1c level over 12 months and to identify care activities that were associated with this change. | | 541 |
| **Design** | Retrospective cohort study | | 541 |
| **Unit of allocation** *(by individuals, cluster/ groups or body parts)* | Individual | | 541 |
| **Start-end date** | January 2014-September 2019 | | 541 |
| **Duration of participation** *(from recruitment to last follow-up/ baseline to last follow-up- group level)* *1. Time of consent until last measurement for each individual.* *2. Baseline to final follow-up for each individual* | 3 to 12 months | | 541 |
| **Study duration** *(as above with the exception of interim analyses or other circumstances* | 3 to 12 months | | 541 |
| **Ethical approval needed/obtained for study** | Yes | This study was approved by the National Healthcare Group Domain Specific Review Board with waiver of informed consent (reference number: 2019/00033). | 546 |
| **Notes** | None | | |

### Participants

| | **Description** *Include comparative information for each intervention or comparison group if available* | **Page number** |
|---|---|---|

| | | | |
|---|---|---|---|
| **Population description** *(from which study participants are drawn)* | Individuals aged 21 years and above, diagnosed with Type 2 diabetes were included in this study. Individuals without HbA1c readings, or who visited the RFMP Clinic or cardiologist clinic for <3 months over the 12-month period were excluded from this study. | | 541 |
| **Setting** *(including location and social context)* | Hospital- outpatient | | 541 |
| **Inclusion/exclusion criteria** | Individuals aged 21 years and above, diagnosed with Type 2 diabetes were included in this study. Individuals without HbA1c readings, or who visited the RFMP Clinic or cardiologist clinic for <3 months over the 12-month period were excluded from this study. | | 541 |
| **Method of recruitment of participants** | Each eligible person who visited the RFMP clinic or received care from a cardiologist in the tertiary hospital between 1 January 2014 and 30 September 2019 were screened for eligibility. Each eligible person who visited the RFMP clinic was assigned to the intervention (INT) group and matched 1:1 based on baseline HbA1c value (±0.2%) with an eligible person who received the usual care from a cardiologist, designated as cardiologist care (CC) group. | | 541 |
| **Informed consent obtained** | Waived | Written informed consent to participate and publish was waived by the National Healthcare Group Domain Specific Review Board. | 546 |
| **Total no. randomised** *(or total pop. at start of study for NRCTs)* | 420 individuals | | 543 (table 1) |
| **Clusters** | NA | | NA |
| **Baseline imbalances** | Baseline sociodemographic and clinical characteristics of participants between the INT and CC were similar except for SBP ($P$ < 0.001), TG level ($P$ = 0.025) and proportions of participants with ischaemic heart disease ($P$ = 0.006) and chronic kidney disease ($P$ = 0.003). | | 542 |

| | | |
|---|---|---|
| **Withdrawals and exclusions** | In the INT group, 116 out of 643 people were excluded due to missing baseline HbA1c value (*n* = 30, 4.7%) and <3 months of attendance at the RFMP clinic (*n* = 86, 13.4%); another 317 people without Type 2 diabetes were also excluded, leaving 210 (32.7%) people for analysis.[9] For the CC group, 1372 out of 2930 people without Type 2 diabetes were excluded. In addition, 1126 (38.4%) people with <3 months of attendance at the cardiologist clinic and another 109 (3.7%) people with missing baseline HbA1c value were also excluded, leaving 320 (10.9%) patients pair matched to the 210 INT group based on baseline HbA1c value (±0.2%). | 542 |
| **Age (years)** | Intervention: 65.8 ± 11.2<br>Comparator: 65.4 ± 11.1 | 543 (table 1) |
| **Sex (female)** | Intervention: 30.5%<br>Comparator: 35.7% | 543 (table 1) |
| **Subgroups measure** | Not reported | NA |
| **Subgroups reported** | Not reported | NA |
| **Notes** | None | |

## Intervention group

| | **Description as stated in report/paper** | **Page number** |
|---|---|---|
| **Group name** | Pharmacist managed clinic | 541 |
| **No. randomised/assigned to group** *(specify whether no. people or clusters)* | 210 | 543 (table 1) |
| **Description** *(include sufficient detail for replication, e.g. content, dose, components)* | The PCC is a referral-based comprehensive medication management service where pharmacists worked collaboratively with cardiologists in co-managing high-risk patients with uncontrolled Type 2 diabetes.[10] Patients are referred to pharmacists by the cardiologists, and pharmacists will provide medication review, identify drug–drug or drug–disease-related problems if any, furnish prescriptions on behalf of the cardiologists with appropriate dose adjustments, empower patients through evidence-based goal settings and establishment of tailored therapy under collaborative practice agreement. Referred patients are followed up by the clinical pharmacists every 2–8 weeks depending on the level of control and discharged from the pharmacist service upon attainment of clinical targets | 541 |
| **Duration of treatment period** | 3-12 months | 541 |

| | | |
|---|---|---|
| **Timing** | Not reported | NA |
| **Co-interventions** | medication review, identify drug–drug or drug–disease-related problems, empower patients through evidence-based goal settings | 541 |
| **Notes** | None | |

## Comparator group

| | **Description as stated in report/paper** | **Page number** |
|---|---|---|
| **Group name** | Cardiologist managed clinic | 541 |
| **No. randomised/assigned to group** *(specify whether no. people or clusters)* | 210 | 543 (table 1) |
| **Description** *(include sufficient detail for replication, e.g. content, dose, components)* | Usual care from a cardiologist | 541 |
| **Duration of treatment period** | 3-12 months | 541 |
| **Timing** | Not reported | NA |
| **Co-interventions** | Not reported | NA |
| **Notes** | None | |

## Outcomes

### Changes in mean HbA1c over 12 months

| | **Description as stated in report/paper** | **Page number** |
|---|---|---|
| **Outcome name** | Changes in mean HbA1c over 12 months | 542 |
| **Time points measured** *(specify whether from start or end of intervention)* | Baseline; 3 month; 6 month; 9 month; 12 months | 544 (figure 1) |
| **Time points reported** | 12 months | 541 |
| **Outcome definition** *(with diagnostic criteria if relevant)* | Changes in mean HbA1c over 12 months | 541 |
| **Unit of measurement** *(if relevant)* | % | 544 (figure 1) |
| **Scales: upper and lower limits** *(indicate whether high or low score is good)* | Lower scores are desired | NA |

| | | | |
|---|---|---|---|
| **Is outcome/tool validated?** | No | Bloodwork | NA |
| **Imputation of missing data** *(e.g. assumptions made for ITT analysis)* | Not reported | | NA |
| **Assumed risk estimate** *(e.g. baseline or population risk noted in Background)* | Not reported | | NA |
| **Power** *(e.g. power & sample size calculation, level of power achieved)* | The sample size was calculated based on an effect size of 0.28, and accounting for Type I error of 5%, a total sample size of 404 was required to achieve the desired power of 80%. | | 541 |
| **Notes** | None | | |

## Funding/conflict of interest

| | | |
|---|---|---|
| **Study funding sources** *(including role of funders)* | This research received no specific grant from any funding agency in the public, commercial or not-for-profit sectors. | 546 |
| **Possible conflicts of interest** *(for study authors)* | Authors declared no conflict of interest. | 546 |
| **Notes** | None | |

## Data and analysis

### Changes in mean HbA1c over 12 months

| | **Description as stated in report/paper** | | | | | | **Page number** |
|---|---|---|---|---|---|---|---|
| **Outcome** | Changes in mean HbA1c over 12 months | | | | | | 542 |
| **Time point** *(specify from start or end of intervention)* | 12 months | | | | | | 542 |
| **Results** | Intervention | | | Comparison | | | 542 |
| | Mean change | SD *(or other variance, specify)* | No. participants | Mean change | SD *(or other variance, specify)* | No. participants | |
| | -0.4% | NR | 210 | -0.2% | NR | 210 | |

| | | | |
|---|---|---|---|
| **Any other results reported** *(e.g. mean difference, CI, P value)* | p<0.001<br><br>The mean HbA1c among INT participants decreased significantly after 6 months (INT: -0.4% versus CC: -0.1%, P = 0.016), with maintenance of improvement at 12 months (INT: -0.4% versus CC: -0.2%, P < 0.001) (Figure 1). | | 542 |
| **No. missing participants** | NA | NA | NA |
| **Statistical methods used and appropriateness of these** *(e.g. adjustment for correlation)* | The sample size was calculated based on an effect size of 0.28, and accounting for Type I error of 5%, a total sample size of 404 was required to achieve the desired power of 80%.[9] Data analysis was performed using the SPSS statistical software (version 27.0; SPSS Inc, Chicago, IL). All statistical tests were two tailed with a significance level (α) of 0.05. Descriptive analyses examining the difference between the two groups were conducted using Student's *t*-test or Mann–Whitney *U*-test for continuous variables and chi-square test for categorical variables as appropriate. Linear mixed model with group (i.e. INT and CC groups) and time (i.e. baseline, 3-, 6-, 9- and 12-month) as fixed effects and HbA1c as outcome was constructed with adjustment for covariates such as blood pressure, lipids and number of visits to healthcare providers. Intercepts, accounting for variability in the participants' HbA1c, were included as random effect. Frequency of each clinical activity was analysed descriptively and included as predictors in a linear regression model with change in HbA1c over 12 months as the outcome. Multiple imputation was used to impute missing data. | | 541 |
| **Notes** | BMI was a moderator of the relationship between participation in PCC model and decrease in HbA1c. Specifically, our findings suggested that a BMI of <24.1 kg/m2 interacted with the interventional group and collectively impacted glycaemic<br>control. | | |

## Conclusions

| | Description as stated in report/paper | Page number |
|---|---|---|
| Key conclusions of study authors | Collaborations between cardiologist and pharmacist have resulted in significant positive clinical outcomes. Furthermore, this study has identified the clinical activities performed by pharmacists that could have resulted in positive outcomes, such as medication management, address of drug-related problems and lifestyle counselling. This study highlighted the important role a PCC model in a specialty setting can play in providing lifestyle counselling and appropriate medication management to improve glycaemic control for people with cardiovascular diseases. Integrating pharmacists into a collaborative care team can also further alleviate system barriers and challenges faced by many healthcare systems. | 546 |
| Notes | None | |

## Maeng et al. 2018

### Study eligibility

| Study Characteristics | Eligibility criteria |
|---|---|
| Title | Impact of a pharmacist-led diabetes management on outcomes, utilization, and cost |
| Author (year) | Maeng et al. 2018 |
| Country | Pennsylvania and New Jersey, USA |
| Type of study | Retrospective cohort study |
| Participants | Patients with diabetes |
| Types of intervention | Pharmacist managed |
| Types of comparison | PCP managed |

| | |
|---|---|
| **Types of outcome measures** | the percent of patients meeting three predefined targets concurrently and percent of patients meeting each individual target at 12 months following the initial MTDM visit with a pharmacist. Those three targets were: glycemic control of HbA1c <8; blood pressure of systolic pressure <130 mmHg and diastolic pressure <80 mmHg; and low-density lipoprotein cholesterol (LDL-C) levels of <100 or <70 mg/dL for patients with coronary heart disease or chronic kidney disease. |
| **Prescriptive authority** | Collaborative practice agreement |
| **Include/Exclude** | Include |
| **Notes** | None |

**DO NOT PROCEED IF STUDY EXCLUDED FROM REVIEW**

## Characteristics of included studies

### Methods

| | Descriptions as stated in report/paper | | Page number |
|---|---|---|---|
| **Aim of study** | To assess the impact of an MTDM program on the achievement of guideline-based disease targets, health care utilization, and cost. | | 552 |
| **Design** | Retrospective cohort study | | 552 |
| **Unit of allocation** *(by individuals, cluster/ groups or body parts)* | Individuals | | 552 |
| **Start-end date** | February 2011 to December 2014 | | 552 |
| **Duration of participation** *(from recruitment to last follow-up/ baseline to last follow-up- group level) 1. Time of consent until last measurement for each individual. 2. Baseline to final follow-up for each individual* | Minimum 12 months | | 552 |
| **Study duration** *(as above with the exception of interim analyses or other circumstances* | Minimum 12 months | | 552 |
| **Ethical approval needed/obtained for study** | Yes | This study was funded by GlaxoSmithKline and approved by Geisinger's Institutional Review Board. | 552 |
| **Notes** | None | | |

### Participants

| | **Description** *Include comparative information for each intervention or comparison group if available* | Page number |
|---|---|---|

| | | | |
|---|---|---|---|
| **Population description** *(from which study participants are drawn)* | The study population was defined as patients who were aged ≥18 years during the study period, who had a primary or secondary diagnosis for DM defined as an ICD, ninth Revision, Clinical Modification (ICD-9-CM) diagnosis code of 250 | | 552/553 |
| **Setting** *(including location and social context)* | Primary care clinic | | 552 |
| **Inclusion/exclusion criteria** | The study population was defined as patients who were aged ≥18 years during the study period, who had a primary or secondary diagnosis for DM defined as an ICD, ninth Revision, Clinical Modification (ICD-9-CM) diagnosis code of 250 received health care services from a Geisinger primary care clinic; and had ≥1 month of both medical and prescription drug coverage through GHP. Patients were excluded if they were pregnant (ICD-9-CM 650.xx) or had gestational diabetes (ICD-9-CM 648.8); if they had any MTDM encounter prior to February 2011 (ie, exposed to MTDM during the pilot phase); or if they had previously requested not to be included in any research studies. Patients with <6 months of EHR data prior to their index date (defined as the date of first visit with a MTDM pharmacist) or <12 months of EHR data after index date were excluded to ensure adequate baseline and follow-up observation. Patients with only one MTDM encounter were also excluded, as a single encounter was attributable to a lack of engagement within the MTDM. | | 552/553 |
| **Method of recruitment of participants** | Geisinger's EHR and GHP claims databases were queried to obtain the retrospective data covering a 6-year period from January 1, 2009, to December 31, 2014. | | 552 |
| **Informed consent obtained** | Not reported | NA | NA |
| **Total no. randomised** *(or total pop. at start of study for NRCTs)* | 5500 individuals | | 554 |
| **Clusters** | NA | | NA |
| **Baseline imbalances** | After the propensity score match, patient characteristics were well balanced (Table 1). Furthermore, prevalence of comorbid conditions was also similar after the matching (shown in Table S1). | | 554 |
| **Withdrawals and exclusions** | NA | | NA |
| **Age** | Intervention: 59 ± 13<br>Comparator: 59 ± 13 | | 556 (table 1) |

| | | |
|---|---|---|
| **Sex (female)** | Intervention: 50.0%<br>Comparator: 48.0% | 556 (table 1) |
| **Subgroups measure** | Not reported | NA |
| **Subgroups reported** | Not reported | NA |
| **Notes** | None | |

## Intervention group

| | Description as stated in report/paper | Page number |
|---|---|---|
| **Group name** | Pharmacist managed MTDM | 552 |
| **No. randomised/assigned to group** *(specify whether no. people or clusters)* | 2750 | 556 (table 1) |
| **Description** *(include sufficient detail for replication, e.g. content, dose, components)* | At the initial visit, the pharmacist extensively interviews and educates the patient and verifies information with the EHR. The pharmacist is authorized to manage prescriptions for all the DM-related conditions. If a new prescription is needed, the MTDM pharmacist ensures that a written, e-prescribed, or telephoned prescription is generated by following collaborative practice guidelines. The pharmacist schedules subsequent MTDM appointments or laboratory testing as needed, independent of the referring physician. | 552 |
| **Duration of treatment period** | Patient follow-up ranged from 365 to 1679 days, with a median of 935 days. As noted above, not all patients had scheduled follow-up visits exactly at or near 365 days. Therefore, a sensitivity analysis was conducted in which patients whose outcome values were carried forward >6 months were excluded from the sample (Table 2). | 555 |
| **Timing** | Approximately 22% (615/2,750) of MTDM cases had between two and five MTDM visits, and over half (1378/2750) had ≥15 MTDM visits, up to a maximum of 146 MTM visits. | 554 |
| **Co-interventions** | patient education, chart review, ordering labs | 552 |
| **Notes** | None | |

## Comparator group

| | Description as stated in report/paper | Page number |
|---|---|---|
| **Group name** | Non-MTDM PCP managed | 552 |
| **No. randomised/assigned to group** *(specify whether no. people or clusters)* | 2750 | 556 (table 1) |

| | | |
|---|---|---|
| **Description** *(include sufficient detail for replication, e.g. content, dose, components)* | Usual care (PCP clinic) | 552 |
| **Duration of treatment period** | Not reported | NA |
| **Timing** | Not reported | NA |
| **Co-interventions** | Not reported | NA |
| **Notes** | None | |

## Outcomes

### HbA1c goal reached

| | **Description as stated in report/paper** | | **Page number** |
|---|---|---|---|
| **Outcome name** | HbA1c goal reached | | 557 (table 2) |
| **Time points measured** *(specify whether from start or end of intervention)* | 12 months (Because this was an observational study, some patients did not have visits scheduled at exactly 12 months of follow-up; in these cases, the most recent measures prior to 12 months were carried forward). | | 553 |
| **Time points reported** | As above | | NA |
| **Outcome definition** *(with diagnostic criteria if relevant)* | Proportion patients reaching HbA1c < 8% | | 553 |
| **Unit of measurement** *(if relevant)* | Percentage | | 557 (table 2) |
| **Scales: upper and lower limits** *(indicate whether high or low score is good)* | Higher proportion is desired | | NA |
| **Is outcome/tool validated?** | No | Bloodwork | NA |
| **Imputation of missing data** *(e.g. assumptions made for ITT analysis)* | Because this was an observational study, some patients did not have visits scheduled at exactly 12 months of follow-up; in these cases, the most recent measures prior to 12 months were carried forward | | 553 |
| **Assumed risk estimate** *(e.g. baseline or population risk noted in Background)* | Not reported | | NA |
| **Power** *(e.g. power & sample size calculation, level of power achieved)* | Not reported | | NA |
| **Notes** | None | | |

## HbA1c levels

| | Description as stated in report/paper | | Page number |
|---|---|---|---|
| **Outcome name** | HbA1c level | | 557 (table 2) |
| **Time points measured** *(specify whether from start or end of intervention)* | 12 months (Because this was an observational study, some patients did not have visits scheduled at exactly 12 months of follow-up; in these cases, the most recent measures prior to 12 months were carried forward). | | 553 |
| **Time points reported** | As above | | NA |
| **Outcome definition** *(with diagnostic criteria if relevant)* | HbA1C level (mean) | | 557 (table 2) |
| **Unit of measurement** *(if relevant)* | Percentage | | 557 (table 2) |
| **Scales: upper and lower limits** *(indicate whether high or low score is good)* | Lower percentage is desired | | NA |
| **Is outcome/tool validated?** | No | Bloodwork | NA |
| **Imputation of missing data** *(e.g. assumptions made for ITT analysis)* | Because this was an observational study, some patients did not have visits scheduled at exactly 12 months of follow-up; in these cases, the most recent measures prior to 12 months were carried forward | | 553 |
| **Assumed risk estimate** *(e.g. baseline or population risk noted in Background)* | Not reported | | NA |
| **Power** *(e.g. power & sample size calculation, level of power achieved)* | Not reported | | NA |
| **Notes** | None | | |

## Blood pressure goal reached

| | Description as stated in report/paper | Page number |
|---|---|---|
| **Outcome name** | Blood pressure goal reached | 557 (table 2) |
| **Time points measured** *(specify whether from start or end of intervention)* | 12 months (Because this was an observational study, some patients did not have visits scheduled at exactly 12 months of follow-up; in these cases, the most recent measures prior to 12 months were carried forward). | 553 |
| **Time points reported** | As above | NA |

| | | | |
|---|---|---|---|
| **Outcome definition** *(with diagnostic criteria if relevant)* | Proportion patients achieving blood pressure of systolic pressure <130 mmHg and diastolic pressure <80 mmHg | | 553 |
| **Unit of measurement** *(if relevant)* | Percentage | | 557 (table 2) |
| **Scales: upper and lower limits** *(indicate whether high or low score is good)* | Higher proportion is desired | | NA |
| **Is outcome/tool validated?** | No | Blood pressure measurement | NA |
| **Imputation of missing data** *(e.g. assumptions made for ITT analysis)* | Because this was an observational study, some patients did not have visits scheduled at exactly 12 months of follow-up; in these cases, the most recent measures prior to 12 months were carried forward | | 553 |
| **Assumed risk estimate** *(e.g. baseline or population risk noted in Background)* | Not reported | | NA |
| **Power** *(e.g. power & sample size calculation, level of power achieved)* | Not reported | | NA |
| **Notes** | None | | |

## Systolic blood pressure levels

| | **Description as stated in report/paper** | | **Page number** |
|---|---|---|---|
| **Outcome name** | Systolic blood pressure | | 557 (table 2) |
| **Time points measured** *(specify whether from start or end of intervention)* | 12 months (Because this was an observational study, some patients did not have visits scheduled at exactly 12 months of follow-up; in these cases, the most recent measures prior to 12 months were carried forward). | | 553 |
| **Time points reported** | As above | | NA |
| **Outcome definition** *(with diagnostic criteria if relevant)* | Systolic blood pressure | | 553 |
| **Unit of measurement** *(if relevant)* | Mean mmHg | | 557 (table 2) |
| **Scales: upper and lower limits** *(indicate whether high or low score is good)* | Lower percentage is desired | | NA |
| **Is outcome/tool validated?** | No | Blood pressure measurement | NA |

| | | |
|---|---|---|
| **Imputation of missing data** *(e.g. assumptions made for ITT analysis)* | Because this was an observational study, some patients did not have visits scheduled at exactly 12 months of follow-up; in these cases, the most recent measures prior to 12 months were carried forward | 553 |
| **Assumed risk estimate** *(e.g. baseline or population risk noted in Background)* | Not reported | NA |
| **Power** *(e.g. power & sample size calculation, level of power achieved)* | Not reported | NA |
| **Notes** | None | |

## Diastolic blood pressure levels

| | Description as stated in report/paper | | Page number |
|---|---|---|---|
| **Outcome name** | Diastolic blood pressure | | 557 (table 2) |
| **Time points measured** *(specify whether from start or end of intervention)* | 12 months (Because this was an observational study, some patients did not have visits scheduled at exactly 12 months of follow-up; in these cases, the most recent measures prior to 12 months were carried forward). | | 553 |
| **Time points reported** | As above | | NA |
| **Outcome definition** *(with diagnostic criteria if relevant)* | Diastolic blood pressure | | 553 |
| **Unit of measurement** *(if relevant)* | Mean mmHg | | 557 (table 2) |
| **Scales: upper and lower limits** *(indicate whether high or low score is good)* | Lower percentage is desired | | NA |
| **Is outcome/tool validated?** | No | Blood pressure measurement | NA |
| **Imputation of missing data** *(e.g. assumptions made for ITT analysis)* | Because this was an observational study, some patients did not have visits scheduled at exactly 12 months of follow-up; in these cases, the most recent measures prior to 12 months were carried forward | | 553 |
| **Assumed risk estimate** *(e.g. baseline or population risk noted in Background)* | Not reported | | NA |
| **Power** *(e.g. power & sample size calculation, level of power achieved)* | Not reported | | NA |
| **Notes** | None | | |

## LDL cholesterol goal reached

| | Description as stated in report/paper | | Page number |
|---|---|---|---|
| **Outcome name** | LDL cholesterol goal reached | | 557 (table 2) |
| **Time points measured** *(specify whether from start or end of intervention)* | 12 months (Because this was an observational study, some patients did not have visits scheduled at exactly 12 months of follow-up; in these cases, the most recent measures prior to 12 months were carried forward). | | 553 |
| **Time points reported** | As above | | NA |
| **Outcome definition** *(with diagnostic criteria if relevant)* | Proportion patients reaching LDL-C levels of <100 or <70 mg/dL | | 553 |
| **Unit of measurement** *(if relevant)* | Percentage | | 557 (table 2) |
| **Scales: upper and lower limits** *(indicate whether high or low score is good)* | Higher proportion is desired | | NA |
| **Is outcome/tool validated?** | No | Bloodwork | NA |
| **Imputation of missing data** *(e.g. assumptions made for ITT analysis)* | Because this was an observational study, some patients did not have visits scheduled at exactly 12 months of follow-up; in these cases, the most recent measures prior to 12 months were carried forward | | 553 |
| **Assumed risk estimate** *(e.g. baseline or population risk noted in Background)* | Not reported | | NA |
| **Power** *(e.g. power & sample size calculation, level of power achieved)* | Not reported | | NA |
| **Notes** | None | | |

## LDL cholesterol levels

| | Description as stated in report/paper | Page number |
|---|---|---|
| **Outcome name** | LDL cholesterol levels | 557 (table 2) |
| **Time points measured** *(specify whether from start or end of intervention)* | 12 months (Because this was an observational study, some patients did not have visits scheduled at exactly 12 months of follow-up; in these cases, the most recent measures prior to 12 months were carried forward). | 553 |
| **Time points reported** | As above | NA |

| | | | |
|---|---|---|---|
| **Outcome definition** *(with diagnostic criteria if relevant)* | LDL-C levels (mean mg/dL) | | 553 |
| **Unit of measurement** *(if relevant)* | Mean mg/dL | | 557 (table 2) |
| **Scales: upper and lower limits** *(indicate whether high or low score is good)* | Lower scores are desired | | NA |
| **Is outcome/tool validated?** | No | Bloodwork | NA |
| **Imputation of missing data** *(e.g. assumptions made for ITT analysis)* | Because this was an observational study, some patients did not have visits scheduled at exactly 12 months of follow-up; in these cases, the most recent measures prior to 12 months were carried forward | | 553 |
| **Assumed risk estimate** *(e.g. baseline or population risk noted in Background)* | Not reported | | NA |
| **Power** *(e.g. power & sample size calculation, level of power achieved)* | Not reported | | NA |
| **Notes** | None | | |

## Funding/conflict of interest

| | | |
|---|---|---|
| **Study funding sources** *(including role of funders)* | Funding for this study has been provided by GlaxoSmithKline. | 559 |
| **Possible conflicts of interest** *(for study authors)* | The authors report no conflicts of interest in this work. | 559 |
| **Notes** | None | |

## Data and analysis

### HbA1c goal reached

| | **Description as stated in report/paper** | | | | **Page number** |
|---|---|---|---|---|---|
| **Outcome** | HbA1c goal reached | | | | 557 (table 2) |
| **Time point** *(specify from start or end of intervention)* | 12 months (Because this was an observational study, some patients did not have visits scheduled at exactly 12 months of follow-up; in these cases, the most recent measures prior to 12 months were carried forward). | | | | 553 |
| **Results** | Intervention | | Comparison | | 557 (table 2) |
| | No. with event | Total in group | No. with event | Total in group | |

| | | | | | |
|---|---|---|---|---|---|
| | 1396 | 2750 | 1564 | 2750 | |
| **Any other results reported** | Both groups had reductions noted in HbA1c from baseline, but the change in HbA1c was more modest in the MTDM group than the comparison group (–0.5% vs –0.7%, *P*<0.0001), resulting in 51% of MTDM patients and 57% of comparison patients at goal (*P*<0.0001). | | | | 555 |
| **No. missing participants** | NA | | NA | | NA |
| **Reasons missing** | NA | | NA | | NA |
| **Statistical methods used and appropriateness of these** | To test for differences in the proportions of patients in the two cohorts who had achieved target levels for HbA1c, LDL-C, and BP at 12 months following the index date, a series of logistic regression models were used with the cohort (MTDM or control) as the explanatory variable. Differences in the proportions of patients who had achieved each target individually and all three targets as a composite outcome were compared, with differences of *P*<0.05 considered statistically significant. Linear regression models were also used to test for differences between cohorts in mean and difference in mean change from baseline, for all three outcome measures at last follow-up. | | | | 553 |
| **Confounders** | Matched cohort<br><br>Race, whether index visit was a primary care visit; age; most recent HbA1c, LDL-C, systolic and diastolic BP, and BMI prior to index; comorbidities; history of coronary artery bypass grafting or coronary revascularization procedures; whether the patient was taking antihypertensive, antihyperlipidemic, or statin medication prior to index; number of ED visits in the prior year; and number of inpatient admissions in the previous year. | | | | 553 |
| **Notes** | None | | | | |

## HbA1c levels

| | **Description as stated in report/paper** | | **Page number** |
|---|---|---|---|
| **Outcome** | HbA1c levels | | 557 (table 2) |
| **Time point** *(specify from start or end of intervention)* | 12 months (Because this was an observational study, some patients did not have visits scheduled at exactly 12 months of follow-up; in these cases, the most recent measures prior to 12 months were carried forward). | | 553 |
| **Results** | Intervention | Comparison | 557 (table 2) |

| | Mean | SD *(or other variance, specify)* | No. participants | Mean | SD *(or other variance, specify)* | No. participants | |
|---|---|---|---|---|---|---|---|
| | 8.3 | 1.8 | 2750 | 8.0 | 1.7 | 2750 | |
| **Any other results reported** *(e.g. mean difference, CI, P value)* | p<0.0001 | | | | | | 557 (table 2) |
| **No. missing participants** | NA | | | NA | | | NA |
| **Statistical methods used and appropriateness of these** *(e.g. adjustment for correlation)* | To test for differences in the proportions of patients in the two cohorts who had achieved target levels for HbA1c, LDL-C, and BP at 12 months following the index date, a series of logistic regression models were used with the cohort (MTDM or control) as the explanatory variable. Differences in the proportions of patients who had achieved each target individually and all three targets as a composite outcome were compared, with differences of *P*<0.05 considered statistically significant. Linear regression models were also used to test for differences between cohorts in mean and difference in mean change from baseline, for all three outcome measures at last follow-up. | | | | | | 553 |
| **Confounders** | Matched cohort<br><br>Race, whether index visit was a primary care visit; age;<br>most recent HbA1c, LDL-C, systolic and diastolic BP, and<br>BMI prior to index; comorbidities; history of coronary artery bypass grafting or coronary revascularization procedures; whether the patient was taking antihypertensive, antihyperlipidemic, or statin medication prior to index; number of ED visits in the prior year; and number of inpatient admissions in the previous year. | | | | | | 553 |
| **Notes** | None | | | | | | |

## Blood pressure goal reached

| | **Description as stated in report/paper** | | | | **Page number** |
|---|---|---|---|---|---|
| **Outcome** | Blood pressure goal reached | | | | 557 (table 2) |
| **Time point** *(specify from start or end of intervention)* | 12 months (Because this was an observational study, some patients did not have visits scheduled at exactly 12 months of follow-up; in these cases, the most recent measures prior to 12 months were carried forward). | | | | 553 |
| **Results** | Intervention | | Comparison | | 557 (table 2) |
| | No. with event | Total in group | No. with event | Total in group | |

| | | | | | |
|---|---|---|---|---|---|
| | 1287 | 2750 | 1241 | 2750 | |
| **Any other results reported** | p=0.21 | | | | 557 (table 2) |
| **No. missing participants** | NA | | NA | | NA |
| **Reasons missing** | NA | | NA | | NA |
| **Statistical methods used and appropriateness of these** | To test for differences in the proportions of patients in the two cohorts who had achieved target levels for HbA1c, LDL-C, and BP at 12 months following the index date, a series of logistic regression models were used with the cohort (MTDM or control) as the explanatory variable. Differences in the proportions of patients who had achieved each target individually and all three targets as a composite outcome were compared, with differences of *P*<0.05 considered statistically significant. Linear regression models were also used to test for differences between cohorts in mean and difference in mean change from baseline, for all three outcome measures at last follow-up. | | | | 553 |
| **Confounders** | Matched cohort<br><br>Race, whether index visit was a primary care visit; age; most recent HbA1c, LDL-C, systolic and diastolic BP, and BMI prior to index; comorbidities; history of coronary artery bypass grafting or coronary revascularization procedures; whether the patient was taking antihypertensive, antihyperlipidemic, or statin medication prior to index; number of ED visits in the prior year; and number of inpatient admissions in the previous year. | | | | 553 |
| **Notes** | None | | | | |

## Systolic blood pressure levels

| | **Description as stated in report/paper** | | | | | | **Page number** |
|---|---|---|---|---|---|---|---|
| **Outcome** | Systolic blood pressure levels | | | | | | 557 (table 2) |
| **Time point** *(specify from start or end of intervention)* | 12 months (Because this was an observational study, some patients did not have visits scheduled at exactly 12 months of follow-up; in these cases, the most recent measures prior to 12 months were carried forward). | | | | | | 553 |
| **Results** | Intervention | | | Comparison | | | 557 (table 2) |
| | Mean | SD *(or other variance, specify)* | No. participants | Mean | SD *(or other variance, specify)* | No. participants | |
| | 129 | 16 | 2750 | 129 | 17 | 2750 | |

| | | | |
|---|---|---|---|
| **Any other results reported** *(e.g. mean difference, CI, P value)* | p=0.57 | | 557 (table 2) |
| **No. missing participants** | NA | NA | NA |
| **Statistical methods used and appropriateness of these** *(e.g. adjustment for correlation)* | To test for differences in the proportions of patients in the two cohorts who had achieved target levels for HbA1c, LDL-C, and BP at 12 months following the index date, a series of logistic regression models were used with the cohort (MTDM or control) as the explanatory variable. Differences in the proportions of patients who had achieved each target individually and all three targets as a composite outcome were compared, with differences of *P*<0.05 considered statistically significant. Linear regression models were also used to test for differences between cohorts in mean and difference in mean change from baseline, for all three outcome measures at last follow-up. | | 553 |
| **Confounders** | Matched cohort<br><br>Race, whether index visit was a primary care visit; age; most recent HbA1c, LDL-C, systolic and diastolic BP, and BMI prior to index; comorbidities; history of coronary artery bypass grafting or coronary revascularization procedures; whether the patient was taking antihypertensive, antihyperlipidemic, or statin medication prior to index; number of ED visits in the prior year; and number of inpatient admissions in the previous year. | | 553 |
| **Notes** | None | | |

## Diastolic blood pressure levels

| | Description as stated in report/paper | | | | | | Page number |
|---|---|---|---|---|---|---|---|
| **Outcome** | Diastolic blood pressure levels | | | | | | 557 (table 2) |
| **Time point** *(specify from start or end of intervention)* | 12 months (Because this was an observational study, some patients did not have visits scheduled at exactly 12 months of follow-up; in these cases, the most recent measures prior to 12 months were carried forward). | | | | | | 553 |
| **Results** | Intervention | | | Comparison | | | 557 (table 2) |
| | Mean | SD *(or other variance, specify)* | No. participants | Mean | SD *(or other variance, specify)* | No. participants | |
| | 72 | 10 | 2750 | 73 | 10 | 2750 | |

| | | | |
|---|---|---|---|
| **Any other results reported** *(e.g. mean difference, CI, P value)* | p=0.70<br><br>The MTDM cohort also showed a smaller decline in DBP from baseline than the non-MTDM comparison cohort (–0.9 to –1.8 mmHg, *P*=0.003); however, DBP goal attainment remained similar between groups. No additional differences between the cohorts were noted. | | 557 (table 2)<br><br>555/556 |
| **No. missing participants** | NA | NA | NA |
| **Statistical methods used and appropriateness of these** *(e.g. adjustment for correlation)* | To test for differences in the proportions of patients in the two cohorts who had achieved target levels for HbA1c, LDL-C, and BP at 12 months following the index date, a series of logistic regression models were used with the cohort (MTDM or control) as the explanatory variable. Differences in the proportions of patients who had achieved each target individually and all three targets as a composite outcome were compared, with differences of *P*<0.05 considered statistically significant. Linear regression models were also used to test for differences between cohorts in mean and difference in mean change from baseline, for all three outcome measures at last follow-up. | | 553 |
| **Confounders** | Matched cohort<br><br>Race, whether index visit was a primary care visit; age; most recent HbA1c, LDL-C, systolic and diastolic BP, and BMI prior to index; comorbidities; history of coronary artery bypass grafting or coronary revascularization procedures; whether the patient was taking antihypertensive, antihyperlipidemic, or statin medication prior to index; number of ED visits in the prior year; and number of inpatient admissions in the previous year. | | 553 |
| **Notes** | None | | |

## LDL cholesterol goal reached

| | Description as stated in report/paper | | | | Page number |
|---|---|---|---|---|---|
| **Outcome** | LDL cholesterol goal reached | | | | 557 (table 2) |
| **Time point** *(specify from start or end of intervention)* | 12 months (Because this was an observational study, some patients did not have visits scheduled at exactly 12 months of follow-up; in these cases, the most recent measures prior to 12 months were carried forward). | | | | 553 |
| **Results** | Intervention | | Comparison | | 557 (table 2) |
| | No. with event | Total in group | No. with event | Total in group | |
| | 1138 | 2750 | 1078 | 2750 | |
| **Any other results reported** | p=0.08 | | | | 557 (table 2) |

| | | | |
|---|---|---|---|
| **No. missing participants** | NA | NA | NA |
| **Reasons missing** | NA | NA | NA |
| **Statistical methods used and appropriateness of these** | To test for differences in the proportions of patients in the two cohorts who had achieved target levels for HbA1c, LDL-C, and BP at 12 months following the index date, a series of logistic regression models were used with the cohort (MTDM or control) as the explanatory variable. Differences in the proportions of patients who had achieved each target individually and all three targets as a composite outcome were compared, with differences of *P*<0.05 considered statistically significant. Linear regression models were also used to test for differences between cohorts in mean and difference in mean change from baseline, for all three outcome measures at last follow-up. | | 553 |
| **Confounders** | Matched cohort<br><br>Race, whether index visit was a primary care visit; age; most recent HbA1c, LDL-C, systolic and diastolic BP, and BMI prior to index; comorbidities; history of coronary artery bypass grafting or coronary revascularization procedures; whether the patient was taking antihypertensive, antihyperlipidemic, or statin medication prior to index; number of ED visits in the prior year; and number of inpatient admissions in the previous year. | | 553 |
| **Notes** | None | | |

## LDL cholesterol levels

| | Description as stated in report/paper | | | | | | Page number |
|---|---|---|---|---|---|---|---|
| **Outcome** | LDL cholesterol levels | | | | | | 557 (table 2) |
| **Time point** *(specify from start or end of intervention)* | 12 months (Because this was an observational study, some patients did not have visits scheduled at exactly 12 months of follow-up; in these cases, the most recent measures prior to 12 months were carried forward). | | | | | | 553 |
| **Results** | Intervention | | | Comparison | | | 557 (table 2) |
| | Mean | SD *(or other variance, specify)* | No. participants | Mean | SD *(or other variance, specify)* | No. participants | |
| | 91 | 37 | 2750 | 92 | 36 | 2750 | |

| | | | |
|---|---|---|---|
| **Any other results reported** *(e.g. mean difference, CI, P value)* | p=0.47 | | 557 (table 2) |
| **No. missing participants** | NA | NA | NA |
| **Statistical methods used and appropriateness of these** *(e.g. adjustment for correlation)* | To test for differences in the proportions of patients in the two cohorts who had achieved target levels for HbA1c, LDL-C, and BP at 12 months following the index date, a series of logistic regression models were used with the cohort (MTDM or control) as the explanatory variable. Differences in the proportions of patients who had achieved each target individually and all three targets as a composite outcome were compared, with differences of *P*<0.05 considered statistically significant. Linear regression models were also used to test for differences between cohorts in mean and difference in mean change from baseline, for all three outcome measures at last follow-up. | | 553 |
| **Confounders** | Matched cohort<br><br>Race, whether index visit was a primary care visit; age; most recent HbA1c, LDL-C, systolic and diastolic BP, and BMI prior to index; comorbidities; history of coronary artery bypass grafting or coronary revascularization procedures; whether the patient was taking antihypertensive, antihyperlipidemic, or statin medication prior to index; number of ED visits in the prior year; and number of inpatient admissions in the previous year. | | 553 |
| **Notes** | None | | |

## Conclusions

| | Description as stated in report/paper | Page number |
|---|---|---|
| **Key conclusions of study authors** | observations of no significant differences in clinical outcomes but a significantly higher estimated rate of ambulatory care visits and lower rate of hospitalizations. | 558 |
| **Notes** | None | |

# Manzoor et al. 2018

## Study eligibility

| Study Characteristics | Eligibility criteria |
|---|---|
| **Title** | Outcomes of systematic anticoagulation management in pharmacist and nurse specialized clinics |
| **Author (year)** | Manzoor et al. (2018) |
| **Country** | Illinois, USA |
| **Type of study** | Retrospective cohort |
| **Participants** | Patients being treated with warfarin |
| **Types of intervention** | Pharmacist-managed anticoagulation clinic |
| **Types of comparison** | Nurse-managed anticoagulation clinic |
| **Types of outcome measures** | PIR (INR values within therapeutic range divided by total INR values); warfarin-related hospitalisations |
| **Prescriptive authority** | Collaborative practice agreement |
| **Include/Exclude** | Include |
| **Notes** | None |

**DO NOT PROCEED IF STUDY EXCLUDED FROM REVIEW**

## Characteristics of included studies

### Methods

| | Descriptions as stated in report/paper | | Page number |
|---|---|---|---|
| **Aim of study** | To compare the quality of anticoagulation-related outcomes via two models of care, PMAC and NMAC. The primary outcome was the quality of anticoagulation management as measured by the proportion of INR levels in therapeutic range (PIR). The secondary outcome was warfarin-related hospitalizations and/or emergency department (ED) visits. | | 69 |
| **Design** | Retrospective cohort | | 69 |
| **Unit of allocation** *(by individuals, cluster/ groups or body parts)* | Individuals | | 69 |
| **Start-end date** | Not reported (consecutive 15 month period) | | 69 |
| **Duration of participation** *(from recruitment to last follow-up/ baseline to last follow-up- group level)* *1. Time of consent until last measurement for each individual.* *2. Baseline to final follow-up for each individual* | A minimum of 1 month | | 69 |
| **Study duration** *(as above with the exception of interim analyses or other circumstances* | As above | | NA |
| **Ethical approval needed/obtained for study** | Not reported | NA | NA |
| **Notes** | None | | |

### Participants

| | Description *Include comparative information for each intervention or comparison group if available* | | Page number |
|---|---|---|---|
| **Population description** *(from which study participants are drawn)* | Patients receiving warfarin therapy | | 69 |
| **Setting** *(including location and social context)* | Outpatient clinic | | 69 |
| **Inclusion/exclusion criteria** | In order to meet study inclusion criteria, patients were greater than 18 years of age and being treated with warfarin at UI Health for a minimum of 1 month | | 69 |
| **Method of recruitment of participants** | Electronic health record | | 69 |
| **Informed consent obtained** | Not reported | NA | NA |
| **Total no. randomised** *(or total pop. at start of study for NRCTs)* | 200 individuals | | 70 |
| **Clusters** | NA | | NA |
| **Baseline imbalances** | The average age was 58.7 +- 15.5 years and 64.2 +- 13.2 years (P = 0.007) in the PMAC and NMAC groups, respectively (Table 1). Males represented 35% and 48% (P = 0.06) of the patient sample in the PMAC and NMAC groups, respectively. African Americans represented the largest ethnicity in the study (58% and 41%, P = 0.07, PMAC and NMAC, respectively). With respect to indications for warfarin therapy, the majority of patients in the PMAC group were being treated for venous thromboembolism (66%), and patients in the NMAC group had atrial fibrillation (53%) and mechanical heart valve replacement (39%) (P < 0.0001). The average PIR was 51.8% _+-15.9% and 56.2% +- 13.9% (P = 0.04) in the PMAC and NMAC groups, respectively. Fifteen percent and 25% (P = 0.08) of patients experienced adverse events resulting in warfarin-related hospitalizations and/or ED visits in the PMAC and NMAC groups, respectively. | | 70 |
| **Withdrawals and exclusions** | None | | NA |
| **Age** | Intervention: 58.7 ± 15.5<br>Comparator: 64.2 ± 13.2 | | 70 (table 1) |
| **Sex (female)** | Intervention: 65%<br>Comparator: 52% | | 70 (table 1) |
| **Subgroups measure** | NA | | NA |
| **Subgroups reported** | NA | | NA |

| | |
|---|---|
| **Notes** | None |

## Intervention group

| | Description as stated in report/paper | Page number |
|---|---|---|
| **Group name** | Pharmacist-managed anticoagulation clinic | 70 (table 1) |
| **No. randomised/assigned to group** *(specify whether no. people or clusters)* | 100 individuals | 70 (table 1) |
| **Description** *(include sufficient detail for replication, e.g. content, dose, components)* | The PMAC is a pharmacist-run, outpatient specialized service providing care to patients referred from primary care providers and specialists from UI Health. The PMAC is recognized as a Center of Excellence by the Anticoagulation Forum. Board-certified clinical pharmacists assist with monitoring and managing UI Health patients receiving warfarin therapy. Similarly, the NMAC assists in managing anticoagulation patients at UI Health Heart Center referred by UI Health cardiologists. The nurses involved in the NMAC have undergone training and competency assessment in anticoagulation management from the clinical pharmacists. In both clinics, pharmacists and nurses independently dose and manage warfarin under institutional collaborative practice agreements | 69 |
| **Duration of treatment period** | No set treatment period. Treatment continued until therapeutic range was reached for each individual or study end. | NA |
| **Timing** | Not reported | NA |
| **Co-interventions** | Not reported | NA |
| **Notes** | None | |

## Comparator group

| | Description as stated in report/paper | Page number |
|---|---|---|
| **Group name** | Nurse-managed anticoagulation clinic | 70 (table 1) |
| **No. randomised/assigned to group** *(specify whether no. people or clusters)* | 100 individuals | 70 (table 1) |

| | | |
|---|---|---|
| **Description** *(include sufficient detail for replication, e.g. content, dose, components)* | The PMAC is a pharmacist-run, outpatient specialized service providing care to patients referred from primary care providers and specialists from UI Health. The PMAC is recognized as a Center of Excellence by the Anticoagulation Forum. Board-certified clinical pharmacists assist with monitoring and managing UI Health patients receiving warfarin therapy. Similarly, the NMAC assists in managing anticoagulation patients at UI Health Heart Center referred by UI Health cardiologists. The nurses involved in the NMAC have undergone training and competency assessment in anticoagulation management from the clinical pharmacists. In both clinics, pharmacists and nurses independently dose and manage warfarin under institutional collaborative practice agreements | 69 |
| **Duration of treatment period** | No set treatment period. Treatment continued until therapeutic range was reached for each individual or study end. | NA |
| **Timing** | Not reported | NA |
| **Co-interventions** | Not reported | NA |
| **Notes** | None | |

## Outcomes

### Proportion of INR levels in therapeutic range

| | **Description as stated in report/paper** | | **Page number** |
|---|---|---|---|
| **Outcome name** | Proportion of INR levels in therapeutic range | | 69 |
| **Time points measured** *(specify whether from start or end of intervention)* | Baseline; 15 months | | 71 |
| **Time points reported** | Baseline; 15 months | | 71 |
| **Outcome definition** *(with diagnostic criteria if relevant)* | INR values within therapeutic range divided by total INR values | | 69 |
| **Unit of measurement** *(if relevant)* | Percentage | | 71 |
| **Scales: upper and lower limits** *(indicate whether high or low score is good)* | Higher proportion desired | | 71 |
| **Is outcome/tool validated?** | No | Bloodwork | NA |

| | | | |
|---|---|---|---|
| **Imputation of missing data** *(e.g. assumptions made for ITT analysis)* | Not reported | | NA |
| **Assumed risk estimate** *(e.g. baseline or population risk noted in Background)* | Not reported | | NA |
| **Power** *(e.g. power & sample size calculation, level of power achieved)* | Power analysis determined that a minimum of 34 patients were needed in each group in order to detect a significant difference (P $\leq$ 0.05) in PIR of 0.1 with 0.8 power. | | 69 |
| **Notes** | None | | |

## Warfarin-related hospitalizations and/or emergency department visits

| | **Description as stated in report/paper** | | **Page number** |
|---|---|---|---|
| **Outcome name** | Warfarin-related hospitalizations and/or emergency department visits | | 69 |
| **Time points measured** *(specify whether from start or end of intervention)* | Baseline; 15 months | | 71 |
| **Time points reported** | Baseline; 15 months | | 71 |
| **Outcome definition** *(with diagnostic criteria if relevant)* | Hospitalisations or ED visits that occurred due to major bleeding, thromboembolic event, or nontherapeutic INRs | | 69 |
| **Unit of measurement** *(if relevant)* | Mean | | 71 |
| **Scales: upper and lower limits** *(indicate whether high or low score is good)* | Lower is desired | | 71 |
| **Is outcome/tool validated?** | No | Bloodwork | No |
| **Imputation of missing data** *(e.g. assumptions made for ITT analysis)* | Not reported | | NA |
| **Assumed risk estimate** *(e.g. baseline or population risk noted in Background)* | Not reported | | NA |

| | | |
|---|---|---|
| **Power** *(e.g. power & sample size calculation, level of power achieved)* | Power analysis determined that a minimum of 34 patients were needed in each group in order to detect a significant difference ($P \leq 0.05$) in PIR of 0.1 with 0.8 power. | 69 |
| **Notes** | None | |

## Funding/conflict of interest

| | | |
|---|---|---|
| **Study funding sources** *(including role of funders)* | Dr. E.A.N. is supported by the National Institute on Minority Health and Health Disparities under Award Number U54MD010723 and the National Heart Lung and Blood Institute under Award Number R21HL140531. | 73 |
| **Possible conflicts of interest** *(for study authors)* | The authors listed above certify that they have no affiliations with or involvement in any organization or entity with any financial or nonfinancial interest in the subject matter or materials discussed in this manuscript. | 73 |
| **Notes** | None | |

## Data and analysis

### Proportion of INR levels in therapeutic range

| | **Description as stated in report/paper** | | | | | | **Page number** |
|---|---|---|---|---|---|---|---|
| **Outcome** | Proportion of INR levels in therapeutic range | | | | | | 71 |
| **Time point** *(specify from start or end of intervention)* | 15 months | | | | | | 69 |
| **Results** | Intervention | | | Comparison | | | 71 |
| | Mean | SD *(or other variance, specify)* | No. participants | Mean | SD *(or other variance, specify)* | No. participants | |
| | NR | NR | 100 | NR | NR | 100 | |
| **Any other results reported** *(e.g. mean difference, CI, P value)* | Compared with the PMAC group, the unadjusted estimate shows that patients receiving care in the NMAC had a higher PIR ($\beta = 4.40$, $P = 0.04$). However, after adjusting for confounders, there was no statistically significant difference in PIR between the NMAC group compared with the PMAC group ($\beta$ -8.41; $p=0.07$). | | | | | | 71 |
| **No. missing participants** | 0 | | | 0 | | | NA |

| | | |
|---|---|---|
| **Statistical methods used and appropriateness of these** *(e.g. adjustment for correlation)* | Demographic and clinical characteristics were compared between the PMAC and NMAC groups using means and SD (or medians and interquartile range [IQR]) for continuous variables and proportions for categorical variables. Continuous variables were compared by Student t-tests or Wilcoxon rank sum test, as appropriate. Categorical variables were compared using chi-square test. Potential confounders were identified by determining whether they altered the crude estimates of the independent-dependent variable relationship by more than 10%. The association between model of care and each respective outcome was determined using a forward-selection multivariate model while adjusting for potential confounders. Linear and logistic multivariate modelling was undertaken for the primary and secondary outcome, respectively. All analysis was conducted using SAS (Cary, NC). | 69/70 |
| **Confounders** | Not clearly specified | NA |
| **Notes** | None | |

## Warfarin-related hospitalizations and/or emergency department visits

| | Description as stated in report/paper | | | | | | Page number |
|---|---|---|---|---|---|---|---|
| **Outcome** | Warfarin-related hospitalizations and/or emergency department visits | | | | | | 71 |
| **Time point** *(specify from start or end of intervention)* | 15 months | | | | | | 69 |
| **Results** | Intervention | | | Comparison | | | 71 |
| | Mean | SD *(or other variance, specify)* | No. participants | Mean | SD *(or other variance, specify)* | No. participants | |
| | NR | NR | 100 | NR | NR | 100 | |
| **Any other results reported** *(e.g. mean difference, CI, P value)* | After adjusting for confounders, the odds of a warfarin-related hospitalization and/or ED visit for patients in the NMAC group compared with the PMAC group is over seven times higher (odds ratio [OR] = 7.68, confidence interval 1.06, 55.94). | | | | | | 71 |
| **No. missing participants** | 0 | | | 0 | | | NA |

| | | |
|---|---|---|
| **Statistical methods used and appropriateness of these** *(e.g. adjustment for correlation)* | Demographic and clinical characteristics were compared between the PMAC and NMAC groups using means and SD (or medians and interquartile range [IQR]) for continuous variables and proportions for categorical variables. Continuous variables were compared by Student t-tests or Wilcoxon rank sum test, as appropriate. Categorical variables were compared using chi-square test. Potential confounders were identified by determining whether they altered the crude estimates of the independent-dependent variable relationship by more than 10%. The association between model of care and each respective outcome was determined using a forward-selection multivariate model while adjusting for potential confounders. Linear and logistic multivariate modelling was undertaken for the primary and secondary outcome, respectively. All analysis was conducted using SAS (Cary, NC). | 69/70 |
| **Confounders** | Not clearly specified | NA |
| **Notes** | None | |

## Conclusions

| | Description as stated in report/paper | Page number |
|---|---|---|
| **Key conclusions of study authors** | Our results indicate that while there is no statistically significant difference in PIR between PMACs and NMACs (P = 0.07), the clinical characteristics of patients managed in the PMAC were more complex and required more personalized and targeted management… With respect to the secondary outcome, our results demonstrate that NMAC patients have greater than seven times the odds of hospitalization or ED visits compared with PMAC patients. These admissions were mainly driven by nontherapeutic INRs and the perceived need for heparinization, especially given the higher proportion of patients with a mechanical valve replacement indication in this group. | 72 |
| **Notes** | None | |

# Marotti et al. 2011

## Study eligibility

| Study Characteristics | Eligibility criteria |
|---|---|
| **Title** | A randomised controlled trial of pharmacist medication histories and supplementary prescribing on medication errors in postoperative medications |
| **Author (year)** | Marotti et al. 2011 |
| **Country** | Australia |
| **Type of study** | RCT |
| **Participants** | Elective surgical patients taking regular medications with a postoperative hospital stay of one night or more |

| | |
|---|---|
| **Types of intervention** | Pharmacist prescribing |
| **Types of comparison** | Pharmacist medication review<br>Usual care |
| **Types of outcome measures** | Doses missed during inpatient stay (adherence); medication charted at incorrect dose (medication errors); medications charted at incorrect frequency (medication errors) |
| **Prescriptive authority** | Supplementary |
| **Include/Exclude** | Include |
| **Notes** | None |

**DO NOT PROCEED IF STUDY EXCLUDED FROM REVIEW**

## Characteristics of included studies

### Methods

| | Descriptions as stated in report/paper | | Page number |
|---|---|---|---|
| **Aim of study** | To measure the effect of pharmacist involvement in medication history taking and supplementary prescribing in the perioperative setting. | | 1064 |
| **Design** | A randomised, three-arm, prospective, parallel group trial | | 1065 |
| **Unit of allocation** *(by individuals, cluster/ groups or body parts)* | Individuals | | 1065 |
| **Start-end date** | November 2008 - March 2009 | | 1066 |
| **Duration of participation** *(from recruitment to last follow-up/ baseline to last follow-up- group level) 1. Time of consent until last measurement for each individual. 2. Baseline to final follow-up for each individual* | Duration of inpatient stay (not specified) | | 1065 |
| **Study duration** *(as above with the exception of interim analyses or other circumstances* | Duration of inpatient stay (not specified) | | 1065 |
| **Ethical approval needed/obtained for study** | Yes | The study protocol was approved by the Hunter New England Human Research Ethics Committee | 1065 |
| **Notes** | None | | |

### Participants

| | **Description** *Include comparative information for each intervention or comparison group if available* | Page number |
|---|---|---|

| | | | |
|---|---|---|---|
| **Population description** *(from which study participants are drawn)* | All adult elective surgery patients admitted to the John Hunter Hospital on the day of surgery were candidates for inclusion in the study. Surgery types included general, cardiothoracic, gynaecology, vascular, urology, ear nose and throat, facio-maxillary and transplant surgery. | | 1065 |
| **Setting** *(including location and social context)* | Hospital | | 1065 |
| **Inclusion/exclusion criteria** | Orthopaedic surgery patients were excluded due to local process differences. Patients were excluded from the trial if they took no regular medications, were unable to provide consent, had medications charted during a preoperative clinic visit or were admitted as a day-only patient. | | 1065 |
| **Method of recruitment of participants** | Consecutive patient | | 1065 |
| **Informed consent obtained** | Yes | After identifying patients suitable for inclusion in the trial, the trial pharmacist obtained written informed consent prior to randomisation. | 1066 |
| **Total no. randomised** *(or total pop. at start of study for NRCTs)* | 355 individuals | | 1066 (table 1) |
| **Clusters** | NA | | NA |
| **Baseline imbalances** | Baseline characteristics were well balanced between the groups (Table 1) and were not adjusted for or used as a covariate for any of the analyses. | | 1066 |
| **Withdrawals and exclusions** | Three patients were excluded at randomisation as they had previously been enrolled in the trial and were to be analysed in their original randomisation group. Two (0.5%) patients were lost to follow-up, as the notes could not be obtained. | | 1066 |
| **Age (years)** | Mean: Not reported<br>Intervention: 64 median; IQR 47-75<br>Comparator 1 (history taking): 62 median; IQR 52-71<br>Comparator 2 (usual care): 65 median; IQR 54-75 | | 1066 (table 1) |
| **Sex (female)** | Intervention: 49%<br>Comparator 1: 55%<br>Comparator 2: 51% | | 1066 (table 1) |
| **Subgroups measure** | Subgroup analysis was performed for several medication groups (beta blockers, 3-hydroxy-3-methyl-glutaryl-CoA reductase inhibitors, anti-platelets and anticoagulants) due to the possible clinical implications that missed doses of these medications may have | | 1065 |

| | | |
|---|---|---|
| **Subgroups reported** | Beta blockers: p=0.013<br>HMG-CoA reductase inhibitors: p<0.001<br>Antiplatelets: p=0.280<br>Anticoagulants: p=0.149 | 1067 (table 2) |
| **Notes** | None | |

## Intervention group

| | **Description as stated in report/paper** | **Page number** |
|---|---|---|
| **Group name** | Pharmacist medication review and prescribing | 1065 |
| **No. randomised/assigned to group** *(specify whether no. people or clusters)* | 118 individuals | 1066 (table 1) |
| **Description** *(include sufficient detail for replication, e.g. content, dose, components)* | The pharmacist interviewed patients at the time of admission on the day of surgery and documented a regular medication list. For patients randomised to supplementary pharmacist prescribing, the pharmacist also prescribed their regular medicines on the medication chart. Pharmacist prescribing was guided by protocols advising which medications should be withheld and for how long, for each type of surgery. These were developed before the study in consultation with surgeons and anaesthetists and approved by the hospital's drug and therapeutics committee. Where patients did not fit the protocol, prescribing was guided by discussion with the patient's medical team. | 1065 |
| **Duration of treatment period** | During hospital stay | 1065 |
| **Timing** | During hospital stay | 1065 |
| **Co-interventions** | Not reported | NA |
| **Notes** | None | |

## Comparator group 1

| | **Description as stated in report/paper** | **Page number** |
|---|---|---|
| **Group name** | Medication review | 1065 |
| **No. randomised/assigned to group** *(specify whether no. people or clusters)* | 119 individuals | 1066 (table 1) |

| | | |
|---|---|---|
| **Description** *(include sufficient detail for replication, e.g. content, dose, components)* | The pharmacist interviewed patients at the time of admission on the day of surgery and documented a regular medication list. | 1065 |
| **Duration of treatment period** | During hospital stay | 1065 |
| **Timing** | During hospital stay | 1065 |
| **Co-interventions** | Not reported | NA |
| **Notes** | None | |

## Comparator group 2

| | **Description as stated in report/paper** | **Page number** |
|---|---|---|
| **Group name** | Usual care | 1065 |
| **No. randomised/assigned to group** *(specify whether no. people or clusters)* | 118 individuals | 1066 (table 1) |
| **Description** *(include sufficient detail for replication, e.g. content, dose, components)* | Usual care involved no clinical pharmacist consultation prior to surgery. These patients had their medications charted immediately prior to surgery or postoperatively by the medical officer in the normal time frame. | 1065 |
| **Duration of treatment period** | During hospital stay | 1065 |
| **Timing** | During hospital stay | 1065 |
| **Co-interventions** | Not reported | NA |
| **Notes** | None | |

# Outcomes

## Doses missed during inpatient stay

| | **Description as stated in report/paper** | **Page number** |
|---|---|---|
| **Outcome name** | Doses missed during inpatient stay | 1067 (table 2) |
| **Time points measured** *(specify whether from start or end of intervention)* | Outcome measures were collected after discharge by an independent technician through retrospective chart review and patient administration system records. | 1066 |
| **Time points reported** | After discharge | 1066 |
| **Outcome definition** *(with diagnostic criteria if relevant)* | Doses missed during inpatient stay | 1067 (table 2) |
| **Unit of measurement** *(if relevant)* | Mean number | 1067 (table 2) |

| | | | |
|---|---|---|---|
| **Scales: upper and lower limits** *(indicate whether high or low score is good)* | Lower scores are desired | | NA |
| **Is outcome/tool validated?** | No | Electronic records | 1066 |
| **Imputation of missing data** *(e.g. assumptions made for ITT analysis)* | All data were analysed using intention-to-treat. The pharmacist was unable to provide the intervention for one patient in group three. Patients who had their surgery cancelled were included in the analysis to determine proportions of cancelled operations but were excluded from the remainder of analysis as these patients had no postoperative data (Figure 1). | | 1067 |
| **Assumed risk estimate** *(e.g. baseline or population risk noted in Background)* | Not reported | | NA |
| **Power** *(e.g. power & sample size calculation, level of power achieved)* | Limited data exist on the incidence of missed medication doses in this patient population, making a formal power calculation based on our primary outcome measure unfeasible. A sample size of 360 was chosen; however it was recognised that this sample size would be unlikely to detect a statistical or clinical difference in any of the secondary outcome measures. | | 1065 |
| **Notes** | None | | |

## Medications charted at incorrect dose

| | **Description as stated in report/paper** | | **Page number** |
|---|---|---|---|
| **Outcome name** | Medications charted at incorrect dose | | 1067 (table 2) |
| **Time points measured** *(specify whether from start or end of intervention)* | Outcome measures were collected after discharge by an independent technician through retrospective chart review and patient administration system records. | | 1066 |
| **Time points reported** | After discharge | | 1066 |
| **Outcome definition** *(with diagnostic criteria if relevant)* | Medications charted at incorrect dose | | 1067 (table 2) |
| **Unit of measurement** *(if relevant)* | Mean number | | 1067 (table 2) |
| **Scales: upper and lower limits** *(indicate whether high or low score is good)* | Lower scores are desired | | NA |
| **Is outcome/tool validated?** | No | Electronic records | 1066 |

| | | |
|---|---|---|
| **Imputation of missing data** *(e.g. assumptions made for ITT analysis)* | All data were analysed using intention-to-treat. The pharmacist was unable to provide the intervention for one patient in group three. Patients who had their surgery cancelled were included in the analysis to determine proportions of cancelled operations but were excluded from the remainder of analysis as these patients had no postoperative data (Figure 1). | 1067 |
| **Assumed risk estimate** *(e.g. baseline or population risk noted in Background)* | Not reported | NA |
| **Power** *(e.g. power & sample size calculation, level of power achieved)* | Limited data exist on the incidence of missed medication doses in this patient population, making a formal power calculation based on our primary outcome measure unfeasible. A sample size of 360 was chosen; however it was recognised that this sample size would be unlikely to detect a statistical or clinical difference in any of the secondary outcome measures. | 1065 |
| **Notes** | None | |

## Medications charted at incorrect frequency

| | Description as stated in report/paper | | Page number |
|---|---|---|---|
| **Outcome name** | Medications charted at incorrect frequency | | 1067 (table 2) |
| **Time points measured** *(specify whether from start or end of intervention)* | Outcome measures were collected after discharge by an independent technician through retrospective chart review and patient administration system records. | | 1066 |
| **Time points reported** | After discharge | | 1066 |
| **Outcome definition** *(with diagnostic criteria if relevant)* | Medications charted at incorrect frequency | | 1067 (table 2) |
| **Unit of measurement** *(if relevant)* | Mean number | | 1067 (table 2) |
| **Scales: upper and lower limits** *(indicate whether high or low score is good)* | Lower scores are desired | | NA |
| **Is outcome/tool validated?** | No | Electronic records | 1066 |

| | | |
|---|---|---|
| **Imputation of missing data** *(e.g. assumptions made for ITT analysis)* | All data were analysed using intention-to-treat. The pharmacist was unable to provide the intervention for one patient in group three. Patients who had their surgery cancelled were included in the analysis to determine proportions of cancelled operations but were excluded from the remainder of analysis as these patients had no postoperative data (Figure 1). | 1067 |
| **Assumed risk estimate** *(e.g. baseline or population risk noted in Background)* | Not reported | NA |
| **Power** *(e.g. power & sample size calculation, level of power achieved)* | Limited data exist on the incidence of missed medication doses in this patient population, making a formal power calculation based on our primary outcome measure unfeasible. A sample size of 360 was chosen; however it was recognised that this sample size would be unlikely to detect a statistical or clinical difference in any of the secondary outcome measures. | 1065 |
| **Notes** | None | |

## Funding/conflict of interest

| | | |
|---|---|---|
| **Study funding sources** *(including role of funders)* | Not reported | NA |
| **Possible conflicts of interest** *(for study authors)* | Not reported | NA |
| **Notes** | None | |

## Data and analysis

### Doses missed during inpatient stay

| | **Description as stated in report/paper** | | | | | | **Page number** |
|---|---|---|---|---|---|---|---|
| **Outcome** | Doses missed during inpatient stay | | | | | | 1067 (Table 2) |
| **Time point** *(specify from start or end of intervention)* | End of inpatient stay | | | | | | 1067 (Table 2) |
| **Results** | Intervention | | | Comparison 1 | | | 1067 (Table 2) |
| | Mean | CI 95% | No. participants | Mean | CI 95% | No. participants | |
| | 1.07 | 0.9-1.25 | 112 | 3.30 | 2.98-3.63 | 109 | |
| | Intervention | | | Comparison 2 | | | |

| | | | | | | | |
|---|---|---|---|---|---|---|---|
| | Mean | CI 95% | No. participants | Mean | CI 95% | No. participants | |
| | 1.07 | 0.9-1.25 | 112 | 3.21 | 2.89-3.52 | 109 | |
| **Any other results reported** *(e.g. mean difference, CI, P value)* | p<0.001 | | | | | | 1067 (Table 2) |
| **No. missing participants** | n=2 | | | Comparison 1: n=0<br>Comparison 2: n=0 | | | 1067 (Table 2) |
| **Statistical methods used and appropriateness of these** *(e.g. adjustment for correlation)* | Generalised linear models were used to assess if there was an effect due to group, with post hoc comparisons of estimated marginal means used to determine where differences between groups were occurring. For the total number of missed doses per patient, Poisson log-linear methods were used with maximum possible number of doses as a covariate. Poisson log-linear methods were also used for number of medications charted at a different dose or different frequency with total number of regular medications as a covariate. Planned sub-group analyses for missed doses of beta blockers, 3-hydroxy-3-methyl-glutaryl-CoA reductase inhibitors, anti-platelets and anticoagulants were analysed using Poisson log-linear methods, with length of hospital stay as a covariate. For all other count data, Poisson log-linear models (with no covariates) were used and for dichotomous outcomes, a binary logit model was used. Results are reported as estimated marginal means with 95% confidence intervals (CI) calculated from the generalised linear models. Results were considered significant at α=0.05. | | | | | | 1066 |
| **Notes** | Subgroup analysis<br><br>Beta blockers: p=0.013<br>HMG-CoA reductase inhibitors: p<0.001<br>Antiplatelets: p=0.280<br>Anticoagulants: p=0.149 | | | | | | |

## Medications charted at incorrect dose

| | Description as stated in report/paper | | Page number |
|---|---|---|---|
| **Outcome** | Medications charted at incorrect dose | | |
| **Time point** *(specify from start or end of intervention)* | End of inpatient stay | | 1067 (Table 2) |
| **Results** | Intervention | Comparison 1 | 1067 (Table 2) |

| | | | | | | | |
|---|---|---|---|---|---|---|---|
| | Mean | CI 95% | No. participants | Mean | CI 95% | No. participants | |
| | 0.02 | 0.00-0.04 | 112 | 0.12 | 0.05-0.18 | 109 | |
| | Intervention | | | Comparison 2 | | | |
| | Mean | CI 95% | No. participants | Mean | CI 95% | No. participants | |
| | 0.02 | 0.00-0.04 | 112 | 0.48 | 0.35-0.18 | 109 | |
| **Any other results reported** *(e.g. mean difference, CI, P value)* | p<0.001 | | | | | | |
| **No. missing participants** | n=2 | | | Comparison 1: n=0<br>Comparison 2: n=0 | | | 1067 (Table 2) |
| **Statistical methods used and appropriateness of these** *(e.g. adjustment for correlation)* | Generalised linear models were used to assess if there was an effect due to group, with post hoc comparisons of estimated marginal means used to determine where differences between groups were occurring. For the total number of missed doses per patient, Poisson log-linear methods were used with maximum possible number of doses as a covariate. Poisson log-linear methods were also used for number of medications charted at a different dose or different frequency with total number of regular medications as a covariate. Planned sub-group analyses for missed doses of beta blockers, 3-hydroxy-3-methyl-glutaryl-CoA reductase inhibitors, anti-platelets and anticoagulants were analysed using Poisson log-linear methods, with length of hospital stay as a covariate. For all other count data, Poisson log-linear models (with no covariates) were used and for dichotomous outcomes, a binary logit model was used. Results are reported as estimated marginal means with 95% confidence intervals (CI) calculated from the generalised linear models. Results were considered significant at α=0.05. | | | | | | 1066 |
| **Notes** | None | | | | | | |

## Medications charted at incorrect frequency

| | **Description as stated in report/paper** | | **Page number** |
|---|---|---|---|
| **Outcome** | | | |
| **Time point** *(specify from start or end of intervention)* | | | |
| **Results** | Intervention | Comparison 1 | 1067 (Table 2) |

| | | | | | | | |
|---|---|---|---|---|---|---|---|
| | Mean | CI 95% | No. participants | Mean | CI 95% | No. participants | |
| | 0.015 | 0.00-0.06 | 112 | 0.07 | 0.02-0.12 | 109 | |
| | Intervention | | | Comparison 2 | | | |
| | Mean | CI 95% | No. participants | Mean | CI 95% | No. participants | |
| | 0.015 | 0.00-0.06 | 112 | 0.29 | 0.19-0.39 | 109 | |
| **Any other results reported** *(e.g. mean difference, CI, P value)* | p<0.001 | | | | | | 1067 (Table 2) |
| **No. missing participants** | n=2 | | | Comparison 1: n=0<br>Comparison 2: n=0 | | | 1067 (Table 2) |
| **Statistical methods used and appropriateness of these** *(e.g. adjustment for correlation)* | Generalised linear models were used to assess if there was an effect due to group, with post hoc comparisons of estimated marginal means used to determine where differences between groups were occurring. For the total number of missed doses per patient, Poisson log-linear methods were used with maximum possible number of doses as a covariate. Poisson log-linear methods were also used for number of medications charted at a different dose or different frequency with total number of regular medications as a covariate. Planned sub-group analyses for missed doses of beta blockers, 3-hydroxy-3-methyl-glutaryl-CoA reductase inhibitors, anti-platelets and anticoagulants were analysed using Poisson log-linear methods, with length of hospital stay as a covariate. For all other count data, Poisson log-linear models (with no covariates) were used and for dichotomous outcomes, a binary logit model was used. Results are reported as estimated marginal means with 95% confidence intervals (CI) calculated from the generalised linear models. Results were considered significant at α=0.05. | | | | | | 1066 |
| **Notes** | None | | | | | | |

## Conclusions

| | **Description as stated in report/paper** | **Page number** |
|---|---|---|

| | | |
|---|---|---|
| **Key conclusions of study authors** | In conclusion, this trial confirms that in this hospital, errors in regular medication management are common for patients admitted for elective surgery. This is similar to findings in other hospitals. Improving the accuracy of documentation of patient medication by pharmacist history taking alone results in a reduction of errors. A significantly greater reduction in medication errors is achieved by changing the perioperative process, with incorporation of pharmacist supplementary prescribing into routine patient care. | 1069 |
| **Notes** | None | |

# McAlister et al. 2014

## Study eligibility

| **Study Characteristics** | **Eligibility criteria** |
|---|---|
| **Title** | Case management for blood pressure and lipid level control after minor stroke: PREVENTION randomized controlled trial |
| **Author (year)** | McAlister et al. 2014 |
| **Country** | Alberta, Canada |
| **Type of study** | RCT |
| **Participants** | Adults with recent minor ischemic stroke or transient ischemic attack whose systolic blood pressure or lipid levels were above guideline targets |
| **Types of intervention** | Pharmacist prescribing |
| **Types of comparison** | Nurse-led case management with physician prescribing |
| **Types of outcome measures** | Systolic blood pressure; fasting LDL; cholesterol; mortality; self-reported adherence |
| **Prescriptive authority** | Collaborative practice agreement |
| **Include/Exclude** | Include |
| **Notes** | None |

**DO NOT PROCEED IF STUDY EXCLUDED FROM REVIEW**

## Characteristics of included studies

### Methods

| | Descriptions as stated in report/paper | | Page number |
|---|---|---|---|
| **Aim of study** | A controlled comparison of 2 modes of case management: active prescribing (pharmacist-led case management) versus screening and delegating to primary care physicians (nurse-led case management). | | 577 |
| **Design** | Prospective, randomized controlled open-label trial | | 577 |
| **Unit of allocation** *(by individuals, cluster/ groups or body parts)* | Individuals | | 577 |
| **Start-end date** | 2009-2012 | | 577 |
| **Duration of participation** *(from recruitment to last follow-up/ baseline to last follow-up- group level)* *1. Time of consent until last measurement for each individual.* *2. Baseline to final follow-up for each individual* | 6 months | | 577 |
| **Study duration** *(as above with the exception of interim analyses or other circumstances* | 6 months | | 577 |
| **Ethical approval needed/obtained for study** | Yes | All participants provided written informed consent, and the study protocol was approved by the Health Research Ethics Board at the University of Alberta. | 578 |
| **Notes** | None | | |

### Participants

| | **Description** *Include comparative information for each intervention or comparison group if available* | **Page number** |
|---|---|---|

| | | |
|---|---|---|
| **Population description** *(from which study participants are drawn)* | We included patients older than 18 years who had an ischemic stroke or transient ischemic attack confirmed by a stroke specialist at 1 of the 3 stroke prevention clinics in Edmonton, Alberta. | 578 |
| **Setting** *(including location and social context)* | Primary care | 578 |
| **Inclusion/exclusion criteria** | Patients were eligible if they had systolic blood pressure or low-density lipoprotein (LDL) cholesterol levels above guideline-recommended targets (average systolic blood pressure over 2 visits > 140 mm Hg, fasting LDL cholesterol > 2.0 mmol/L, or total:high-density lipoprotein (HDL) cholesterol ratio > 4.0). Patients were excluded if they had impaired cognition, severe disability, were institutionalized, had a condition that would preclude<br>follow-up, had hypertensive urgency (systolic blood pressure ≥ 200 mm Hg) or had treatment refractory hypertension or dyslipidemia (i.e., already taking 3 medications and above target levels), or if they were participating in another trial. | 578 |
| **Method of recruitment of participants** | Recruited through stroke specialist | 578 |
| **Informed consent obtained** | Yes — All participants provided written informed consent, and the study protocol was approved by the Health Research Ethics Board at the University of Alberta. | 578 |
| **Total no. randomised** *(or total pop. at start of study for NRCTs)* | 279 randomised | 579 (figure 1) |
| **Clusters** | NA | NA |
| **Baseline imbalances** | The characteristics of both study groups were similar at baseline (Table 1). | 580 |
| **Withdrawals and exclusions** | Nurse-led excluded: *n* = 9<br>• Declined further participation before 6 month visit *n* = 7<br>• Moved out of region *n* = 1<br>• Died *n* = 1<br><br>Pharmacist led excluded: *n* = 31<br>• Did not receive allocated intervention (alternate non-stroke cause for symptoms detected) *n* = 13<br>• Early withdrawal *n* = 18<br>• (declined further participation before 6-month visit *n* = 16; moved out of region *n* = 1; enrolled in another trial *n* = 1) | 579 (figure 1) |
| **Age (years)** | Intervention: 66.8 ± 11.1<br>Comparator: 66.3 ± 11.3 | 581 (table 1) |

| | | |
|---|---|---|
| **Sex (female)** | Intervention: 39.2%<br>Comparator: 44.8% | 581 (table 1) |
| **Subgroups measure** | Intention to treat<br>Per protocol | 580 |
| **Subgroups reported** | The results of subgroup analyses were consistent with the main results. For example, among patients who had experienced a transient ischemic attack and who had moderate or high ABCD scores at baseline, control rates after 6 months were 48.7% in the pharmacist-led group compared with 26.3% in the nurse-led group (*p* = 0.04). | 580 |
| **Notes** | | |

## Intervention group

| | Description as stated in report/paper | Page number |
|---|---|---|
| **Group name** | Pharmacist management | 577 |
| **No. randomised/assigned to group** *(specify whether no. people or clusters)* | 143 individuals | 579 (figure 1) |
| **Description** *(include sufficient detail for replication, e.g. content, dose, components)* | Our intervention involved active case management by 4 pharmacists who saw study participants monthly in a clinic setting for 6 months. Pharmacists performed the same tasks as the nurses in the control arm, as well as initiated or titrated antihypertensive and/or lipid-lowering therapy as appropriate (using treatment algorithms and targets consistent with current Canadian guidelines). The pharmacists did not undergo standardized training; however, they were all at similar stages of their career and were provided with the same patient educational materials and treatment algorithms. | 578 |
| **Duration of treatment period** | Six months | 577 |
| **Timing** | Not reported | NA |
| **Co-interventions** | Medication review (delivered by nurses in control group) | 578 |
| **Notes** | None | |

## Comparator group

| | Description as stated in report/paper | Page number |
|---|---|---|
| **Group name** | Nurse-led case management with physician prescribing | 578 |

| | | |
|---|---|---|
| **No. randomised/assigned to group** *(specify whether no. people or clusters)* | 143 individuals | 579 (figure 1) |
| **Description** *(include sufficient detail for replication, e.g. content, dose, components)* | All 3 stroke prevention clinics in Edmonton enrolled patients, and the attending neurologists provided written suggestions to primary care physicians with respect to treatment targets for vascular risk factors. As per local practice, the neurologists delegated ongoing management to the patient's primary care physician and rarely saw patients after the initial visit. All patients in this group also had monthly clinic visits for 6 months with a study nurse who provided lifestyle advice (exercise, low-salt diet, smoking cessation, medication adherence), checked the patient's blood pressure and LDL level, and faxed blood pressure measurements and a list of current medications to the patient's primary care physician after each visit. | 578 |
| **Duration of treatment period** | Six months | 577 |
| **Timing** | Not reported | NA |
| **Co-interventions** | Medication review (delivered by nurses in control group) | 578 |
| **Notes** | None | |

## Outcomes

### Attained optimal systolic blood pressure and lipid level by 6 months

| | **Description as stated in report/paper** | **Page number** |
|---|---|---|
| **Outcome name** | Attained optimal systolic blood pressure and lipid level by 6 months | 578 |
| **Time points measured** *(specify whether from start or end of intervention)* | Baseline; 6 months | 582 (table 2) |
| **Time points reported** | 6 months | 582 (table 2) |
| **Outcome definition** *(with diagnostic criteria if relevant)* | Attained optimal systolic blood pressure and lipid level by 6 months defined as systolic blood pressure <=140 mm Hg and fasting LDL cholesterol 2.0 mmol/L, based on Canadian clinical practice guidelines | 582 (table 2), 578 |
| **Unit of measurement** *(if relevant)* | Systolic BP and lipid levels: Event data | NA |
| **Scales: upper and lower limits** *(indicate whether high or low score is good)* | Within range is desired | NA |

| | | | |
|---|---|---|---|
| **Is outcome/tool validated?** | No | BP measurement | NA |
| **Imputation of missing data** *(e.g. assumptions made for ITT analysis)* | Missing data at the 6 month follow-up assessment were imputed with a last-observation carried forward strategy; | | 579 |
| **Assumed risk estimate** *(e.g. baseline or population risk noted in Background)* | Not reported | | NA |
| **Power** *(e.g. power & sample size calculation, level of power achieved)* | Based on a survey of members of the divisions of neurology and general internal medicine at the University of Alberta, we determined that the minimal clinically important difference for the pharmacist case manager intervention to be considered useful was a 10% absolute improvement over and above any improvements in the active control group for our primary outcome. This required a sample size of 280. | | 579 |
| **Notes** | None | | |

## Systolic BP

| | Description as stated in report/paper | | Page number |
|---|---|---|---|
| **Outcome name** | Systolic BP | | 582 (table 2) |
| **Time points measured** *(specify whether from start or end of intervention)* | Baseline; 6 months | | 582 (table 2) |
| **Time points reported** | 6 months | | 582 (table 2) |
| **Outcome definition** *(with diagnostic criteria if relevant)* | Systolic BP | | 582 (table 2) |
| **Unit of measurement** *(if relevant)* | mm Hg, continuous variable | | NA |
| **Scales: upper and lower limits** *(indicate whether high or low score is good)* | Lower score is desired (guideline-recommended = <140 mm Hg) | | , 578 |
| **Is outcome/tool validated?** | No | BP measurement | NA |
| **Imputation of missing data** *(e.g. assumptions made for ITT analysis)* | Missing data at the 6 month follow-up assessment were imputed with a last-observation carried forward strategy; | | 579 |

| | | |
|---|---|---|
| **Assumed risk estimate** *(e.g. baseline or population risk noted in Background)* | Not reported | NA |
| **Power** *(e.g. power & sample size calculation, level of power achieved)* | Based on a survey of members of the divisions of neurology and general internal medicine at the University of Alberta, we determined that the minimal clinically important difference for the pharmacist case manager intervention to be considered useful was a 10% absolute improvement over and above any improvements in the active control group for our primary outcome. This required a sample size of 280. | 579 |
| **Notes** | None | |

## LDL cholesterol

| | Description as stated in report/paper | | Page number |
|---|---|---|---|
| **Outcome name** | LDL cholesterol | | 582 (table 2) |
| **Time points measured** *(specify whether from start or end of intervention)* | Baseline; 6 months | | 582 (table 2) |
| **Time points reported** | 6 months | | 582 (table 2) |
| **Outcome definition** *(with diagnostic criteria if relevant)* | LDL cholesterol | | 582 (table 2) |
| **Unit of measurement** *(if relevant)* | mmol/L, continuous variable | | NA |
| **Scales: upper and lower limits** *(indicate whether high or low score is good)* | Lower score is desired (guideline-recommended <=2.0 mmol/L) | | 578 |
| **Is outcome/tool validated?** | No | Bloodwork | NA |
| **Imputation of missing data** *(e.g. assumptions made for ITT analysis)* | Missing data at the 6 month follow-up assessment were imputed with a last-observation carried forward strategy; | | 579 |
| **Assumed risk estimate** *(e.g. baseline or population risk noted in Background)* | Not reported | | NA |

| | | |
|---|---|---|
| **Power** *(e.g. power & sample size calculation, level of power achieved)* | Based on a survey of members of the divisions of neurology and general internal medicine at the University of Alberta, we determined that the minimal clinically important difference for the pharmacist case manager intervention to be considered useful was a 10% absolute improvement over and above any improvements in the active control group for our primary outcome. This required a sample size of 280. | 579 |
| **Notes** | None | |

## Mortality

| | Description as stated in report/paper | | Page number |
|---|---|---|---|
| **Outcome name** | Mortality | | 582 (table 2) |
| **Time points measured** *(specify whether from start or end of intervention)* | Baseline; 6 months | | 582 (table 2) |
| **Time points reported** | 6 months | | 582 (table 2) |
| **Outcome definition** *(with diagnostic criteria if relevant)* | Mortality | | 582 (table 2) |
| **Unit of measurement** *(if relevant)* | Event data | | NA |
| **Scales: upper and lower limits** *(indicate whether high or low score is good)* | Lower score is desired | | NA |
| **Is outcome/tool validated?** | No | Not reported | NA |
| **Imputation of missing data** *(e.g. assumptions made for ITT analysis)* | Not reported | | NA |
| **Assumed risk estimate** *(e.g. baseline or population risk noted in Background)* | Not reported | | NA |
| **Power** *(e.g. power & sample size calculation, level of power achieved)* | Based on a survey of members of the divisions of neurology and general internal medicine at the University of Alberta, we determined that the minimal clinically important difference for the pharmacist case manager intervention to be considered useful was a 10% absolute improvement over and above any improvements in the active control group for our primary outcome. This required a sample size of 280. | | 579 |
| **Notes** | None | | |

## Vascular event

| | Description as stated in report/paper | | Page number |
|---|---|---|---|
| **Outcome name** | Vascular event | | 582 (table 2) |
| **Time points measured** *(specify whether from start or end of intervention)* | Baseline; 6 months | | 582 (table 2) |
| **Time points reported** | 6 months | | 582 (table 2) |
| **Outcome definition** *(with diagnostic criteria if relevant)* | Vascular events (Myocardial infarction, stroke or transient ischemic attack, coronary or carotid revascularization) | | 582 (table 2) |
| **Unit of measurement** *(if relevant)* | Event data | | NA |
| **Scales: upper and lower limits** *(indicate whether high or low score is good)* | Lowers score is desired | | NA |
| **Is outcome/tool validated?** | No | Not reported | NA |
| **Imputation of missing data** *(e.g. assumptions made for ITT analysis)* | Not reported | | NA |
| **Assumed risk estimate** *(e.g. baseline or population risk noted in Background)* | Not reported | | NA |
| **Power** *(e.g. power & sample size calculation, level of power achieved)* | Based on a survey of members of the divisions of neurology and general internal medicine at the University of Alberta, we determined that the minimal clinically important difference for the pharmacist case manager intervention to be considered useful was a 10% absolute improvement over and above any improvements in the active control group for our primary outcome. This required a sample size of 280. | | 579 |
| **Notes** | None | | |

## Change in HDL cholesterol

| | Description as stated in report/paper | Page number |
|---|---|---|
| **Outcome name** | Change in HDL cholesterol | 582 (table 2) |

| | | | |
|---|---|---|---|
| **Time points measured** *(specify whether from start or end of intervention)* | Baseline; 6 months | | 582 (table 2) |
| **Time points reported** | 6 months | | 582 (table 2) |
| **Outcome definition** *(with diagnostic criteria if relevant)* | Change in HDL cholesterol | | 582 (table 2) |
| **Unit of measurement** *(if relevant)* | mmol/L | | NA |
| **Scales: upper and lower limits** *(indicate whether high or low score is good)* | Lowers score is desired | | NA |
| **Is outcome/tool validated?** | No | Not reported | NA |
| **Imputation of missing data** *(e.g. assumptions made for ITT analysis)* | Not reported | | NA |
| **Assumed risk estimate** *(e.g. baseline or population risk noted in Background)* | Not reported | | NA |
| **Power** *(e.g. power & sample size calculation, level of power achieved)* | Based on a survey of members of the divisions of neurology and general internal medicine at the University of Alberta, we determined that the minimal clinically important difference for the pharmacist case manager intervention to be considered useful was a 10% absolute improvement over and above any improvements in the active control group for our primary outcome. This required a sample size of 280. | | 579 |
| **Notes** | None | | |

## Self-reported adherence of 75% or higher for blood pressure or lipid-lowering medications

| | **Description as stated in report/paper** | **Page number** |
|---|---|---|
| **Outcome name** | Self-reported adherence of 75% or higher for blood pressure or lipid-lowering medications | 582 (table 2) |
| **Time points measured** *(specify whether from start or end of intervention)* | Baseline; 6 months | 582 (table 2) |
| **Time points reported** | 6 months | 582 (table 2) |

| | | | |
|---|---|---|---|
| **Outcome definition** *(with diagnostic criteria if relevant)* | Self-reported adherence of 75% or higher for blood pressure or lipid-lowering medications | | 582 (table 2) |
| **Unit of measurement** *(if relevant)* | Self-reported | | NA |
| **Scales: upper and lower limits** *(indicate whether high or low score is good)* | Event data | | NA |
| **Is outcome/tool validated?** | No | Self-report | NA |
| **Imputation of missing data** *(e.g. assumptions made for ITT analysis)* | Not reported | | NA |
| **Assumed risk estimate** *(e.g. baseline or population risk noted in Background)* | Not reported | | NA |
| **Power** *(e.g. power & sample size calculation, level of power achieved)* | Based on a survey of members of the divisions of neurology and general internal medicine at the University of Alberta, we determined that the minimal clinically important difference for the pharmacist case manager intervention to be considered useful was a 10% absolute improvement over and above any improvements in the active control group for our primary outcome. This required a sample size of 280. | | 579 |
| **Notes** | None | | |

## Overall self-rated health

| | Description as stated in report/paper | Page number |
|---|---|---|
| **Outcome name** | Self-rated health | 582 (table 2) |
| **Time points measured** *(specify whether from start or end of intervention)* | Baseline; 6 months | 582 (table 2) |
| **Time points reported** | 6 months | 582 (table 2) |
| **Outcome definition** *(with diagnostic criteria if relevant)* | Self-rated heath scored on a 5-point Likert scale with 5 being excellent and 1 being poor. | 582 (table 2) |
| **Unit of measurement** *(if relevant)* | Continuous | NA |

| | | | |
|---|---|---|---|
| **Scales: upper and lower limits** *(indicate whether high or low score is good)* | Higher score is desired | | NA |
| **Is outcome/tool validated?** | No | Likert scale | NA |
| **Imputation of missing data** *(e.g. assumptions made for ITT analysis)* | Not reported | | NA |
| **Assumed risk estimate** *(e.g. baseline or population risk noted in Background)* | Not reported | | NA |
| **Power** *(e.g. power & sample size calculation, level of power achieved)* | Based on a survey of members of the divisions of neurology and general internal medicine at the University of Alberta, we determined that the minimal clinically important difference for the pharmacist case manager intervention to be considered useful was a 10% absolute improvement over and above any improvements in the active control group for our primary outcome. This required a sample size of 280. | | 579 |
| **Notes** | None | | |

## EQ-5D index score

| | Description as stated in report/paper | | Page number |
|---|---|---|---|
| **Outcome name** | EQ-5D | | 582 (table 2) |
| **Time points measured** *(specify whether from start or end of intervention)* | Baseline; 6 months | | 582 (table 2) |
| **Time points reported** | 6 months | | 582 (table 2) |
| **Outcome definition** *(with diagnostic criteria if relevant)* | Quality of life | | 582 (table 2) |
| **Unit of measurement** *(if relevant)* | Continuous | | NA |
| **Scales: upper and lower limits** *(indicate whether high or low score is good)* | Higher score is desired | | NA |
| **Is outcome/tool validated?** | Yes | EQ-5D | NA |

| | | |
|---|---|---|
| **Imputation of missing data** *(e.g. assumptions made for ITT analysis)* | Not reported | NA |
| **Assumed risk estimate** *(e.g. baseline or population risk noted in Background)* | Not reported | NA |
| **Power** *(e.g. power & sample size calculation, level of power achieved)* | Based on a survey of members of the divisions of neurology and general internal medicine at the University of Alberta, we determined that the minimal clinically important difference for the pharmacist case manager intervention to be considered useful was a 10% absolute improvement over and above any improvements in the active control group for our primary outcome. This required a sample size of 280. | 579 |
| **Notes** | None | |

## Funding/conflict of interest

| | | |
|---|---|---|
| **Study funding sources** *(including role of funders)* | Project-specific funding for this trial was provided by the Heart and Stroke Foundation of Alberta, the Alberta Heritage Foundation for Medical Research, and Knowledge Translation Canada. | 584 |
| **Possible conflicts of interest** *(for study authors)* | Raj Padwal is a site investigator for clinical trials for NovoNordisk and CVRx, and has received grant funding for a blood pressure cuff study. He has received personal fees for hypertension talks from Merck, Abbott and Servier and for advisory board service from Medtronic and Forest. Ashfaq Shuaib has received speaker bureau fees from CoAxia, Pfizer, BI, Sanofi, Bayer, AstraZeneca, Lundbeck, D-Pharm, BrainsGate, Tribute and Bristol Myers Squibb. He has received grant funding from Lundbeck, D-Pharm, GlaxoSmithKline, Asubio, PhotoThera, BrainsGate, WL Gore and Aga Medical. Ross Tsuyuki has received consultancy fees from Bristol Myers Squibb, AstraZeneca, PharmaSmart International, Merck and Abbott. He has received grant funding from Sanofi and AstraZeneca and serves on the data monitoring board for Boehringer Ingelheim. No other competing interests declared. | 5774 |
| **Notes** | None | |

## Data and analysis

## Attained optimal systolic blood pressure and lipid level by 6 months

| | Description as stated in report/paper | | | | Page number |
|---|---|---|---|---|---|
| **Outcome** | Attained optimal systolic blood pressure and lipid level by 6 months | | | | 582 (table 2) |
| **Time point** *(specify from start or end of intervention)* | 6 months | | | | 582 (table 2) |
| **Results** | Intervention | | Comparison | | 582 (table 2) |
| | No. with event | Total in group | No. with event | Total in group | |
| | 62 | 143 | 42 | 136 | |
| **Any other results reported** | Difference (intervention – control), SD* (95% CI): 12.5% (0.4 to 24.0) | | | | 582 (table 2) |
| **No. missing participants** | 33 | | 26 | | 579 (figure 1) |
| **Reasons missing** | • Declined further participation before 6 month visit: n=7<br>• Moved out of region: n=1<br>• Died: n=1<br>• Reason not specified: n=24 | | • Did not receive allocated intervention: n=13<br>• Early withdrawal: n=18<br>• Reason not specified: n=2 | | 579 (figure 1) |
| **Statistical methods used and appropriateness of these** | We used χ2 tests to compare the proportion of patients who attained optimal blood pressure and lipid control at 6 months (because the proportion at baseline was zero by design); we used χ2 testing for other binary secondary outcomes. To compare changes in systolic blood pressure, LDL cholesterol, and total:HDL cholesterol ratios between the intervention and control groups, we used 2-sample independent *t* tests. We conducted multiple logistic regression for our primary outcome to adjust for study site and any clinically important (> 10% imbalance between arms) or statistically significant (*p* < 0.1 between arms) baseline differences. Our primary analysis was intention-to-treat. Missing data at the 6 month follow-up assessment were imputed with a last-observation carried forward strategy; this approach conservatively assumes that all participants lost to follow-up had no change in their blood pressure or lipid levels after their last recorded value. | | | | 579 |
| **Notes** | None | | | | |

## Systolic blood pressure

| | Description as stated in report/paper | Page number |
|---|---|---|
| **Outcome** | Systolic blood pressure | 582 (table 2) |

| | | | | | | | |
|---|---|---|---|---|---|---|---|
| **Time point** *(specify from start or end of intervention)* | 6 months | | | | | | 582 (table 2) |
| **Results** | Intervention | | | Comparison | | | 582 (table 2) |
| | Mean | SD *(or other variance, specify)* | No. participants | Mean | SD *(or other variance, specify)* | No. participants | |
| | 126.5 | 17.9 | 143 | 122.2 | 13.0 | 136 | |
| **Any other results reported** *(e.g. mean difference, CI, P value)* | Difference (intervention – control), SD* (95% CI): 4.3 (0.6 to 8.0) | | | | | | 582 (table 2) |
| **No. missing participants** | 33 | | | 26 | | | 579 (figure 1) |
| **Reasons missing** | • Declined further participation before 6 month visit: n=7<br>• Moved out of region: n=1<br>• Died: n=1<br>• Reason not specified: n=24 | | | • Did not receive allocated intervention: n=13<br>• Early withdrawal: n=18<br>• Reason not specified: n=2 | | | 579 (figure 1) |
| **Statistical methods used and appropriateness of these** *(e.g. adjustment for correlation)* | We used χ2 tests to compare the proportion of patients who attained optimal blood pressure and lipid control at 6 months (because the proportion at baseline was zero by design); we used χ2 testing for other binary secondary outcomes. To compare changes in systolic blood pressure, LDL cholesterol, and total:HDL cholesterol ratios between the intervention and control groups, we used 2-sample independent *t* tests. We conducted multiple logistic regression for our primary outcome to adjust for study site and any clinically important (> 10% imbalance between arms) or statistically significant ($p < 0.1$ between arms) baseline differences. Our primary analysis was intention-to-treat. Missing data at the 6 month follow-up assessment were imputed with a last-observation carried forward strategy; this approach conservatively assumes that all participants lost to follow-up had no change in their blood pressure or lipid levels after their last recorded value. | | | | | | 579 |
| **Notes** | None | | | | | | |

## LDL cholesterol

| | Description as stated in report/paper | Page number |
|---|---|---|
| **Outcome** | LDL cholesterol | 582 (table 2) |

| | | | | | | | |
|---|---|---|---|---|---|---|---|
| **Time point** *(specify from start or end of intervention)* | 6 months | | | | | | 582 (table 2) |
| **Results** | Intervention | | | Comparison | | | 582 (table 2) |
| | Mean | SD *(or other variance, specify)* | No. participants | Mean | SD *(or other variance, specify)* | No. participants | |
| | 2.21 | 0.73 | 143 | 2.35 | 0.81 | 136 | |
| **Any other results reported** *(e.g. mean difference, CI, P value)* | Difference (intervention – control), SD* (95% CI): –0.14 (–0.32 to 0.04) | | | | | | 582 (table 2) |
| **No. missing participants** | 33 | | | 26 | | | 579 (figure 1) |
| **Reasons missing** | • Declined further participation before 6 month visit: n=7<br>• Moved out of region: n=1<br>• Died: n=1<br>• Reason not specified: n=24 | | | • Did not receive allocated intervention: n=13<br>• Early withdrawal: n=18<br>• Reason not specified: n=2 | | | 579 (figure 1) |
| **Statistical methods used and appropriateness of these** *(e.g. adjustment for correlation)* | We used χ2 tests to compare the proportion of patients who attained optimal blood pressure and lipid control at 6 months (because the proportion at baseline was zero by design); we used χ2 testing for other binary secondary outcomes. To compare changes in systolic blood pressure, LDL cholesterol, and total:HDL cholesterol ratios between the intervention and control groups, we used 2-sample independent *t* tests. We conducted multiple logistic regression for our primary outcome to adjust for study site and any clinically important (> 10% imbalance between arms) or statistically significant ($p < 0.1$ between arms) baseline differences. Our primary analysis was intention-to-treat. Missing data at the 6 month follow-up assessment were imputed with a last-observation carried forward strategy; this approach conservatively assumes that all participants lost to follow-up had no change in their blood pressure or lipid levels after their last recorded value. | | | | | | 579 |
| **Notes** | None | | | | | | |

## Mortality

| | Description as stated in report/paper | Page number |
|---|---|---|
| **Outcome** | Mortality | 582 (table 2) |

| | | | | | |
|---|---|---|---|---|---|
| **Time point** *(specify from start or end of intervention)* | 6 months | | | | 582 (table 2) |
| **Results** | Intervention | | Comparison | | 582 (table 2) |
| | No. with event | Total in group | No. with event | Total in group | |
| | 0 | 143 | 1 | 136 | |
| **Any other results reported** | Difference (intervention – control), SD* (95% CI): –0.7% (–0.7 to 0.6) | | | | 582 (table 2) |
| **No. missing participants** | 32 | | 26 | | 579 (figure 1) |
| **Reasons missing** | • Declined further participation before 6 month visit: n=7<br>• Moved out of region: n=1<br>• Reason not specified: n=24 | | • Did not receive allocated intervention: n=13<br>• Early withdrawal: n=18<br>• Reason not specified: n=2 | | 579 (figure 1) |
| **Statistical methods used and appropriateness of these** | We used χ2 tests to compare the proportion of patients who attained optimal blood pressure and lipid control at 6 months (because the proportion at baseline was zero by design); we used χ2 testing for other binary secondary outcomes. To compare changes in systolic blood pressure, LDL cholesterol, and total:HDL cholesterol ratios between the intervention and control groups, we used 2-sample independent *t* tests. We conducted multiple logistic regression for our primary outcome to adjust for study site and any clinically important (> 10% imbalance between arms) or statistically significant (*p* < 0.1 between arms) baseline differences. Our primary analysis was intention-to-treat. Missing data at the 6 month follow-up assessment were imputed with a last-observation carried forward strategy; this approach conservatively assumes that all participants lost to follow-up had no change in their blood pressure or lipid levels after their last recorded value. | | | | 579 |
| **Notes** | None | | | | |

## Vascular event

| | Description as stated in report/paper | | Page number |
|---|---|---|---|
| **Outcome** | Vascular event | | 582 (table 2) |
| **Time point** *(specify from start or end of intervention)* | 6 months | | 582 (table 2) |
| **Results** | Intervention | Comparison | 582 (table 2) |

| | | | | | |
|---|---|---|---|---|---|
| | No. with event | Total in group | No. with event | Total in group | |
| | 9 | 143 | 8 | 136 | |
| **Any other results reported** | Difference (intervention – control), SD* (95% CI): 0.4% (–5.4 to 6.0) | | | | 582 (table 2) |
| **No. missing participants** | 32 | | 26 | | 579 (figure 1) |
| **Reasons missing** | • Declined further participation before 6 month visit: n=7<br>• Moved out of region: n=1<br>• Reason not specified: n=24 | | • Did not receive allocated intervention: n=13<br>• Early withdrawal: n=18<br>• Reason not specified: n=2 | | 579 (figure 1) |
| **Statistical methods used and appropriateness of these** | We used χ2 tests to compare the proportion of patients who attained optimal blood pressure and lipid control at 6 months (because the proportion at baseline was zero by design); we used χ2 testing for other binary secondary outcomes. To compare changes in systolic blood pressure, LDL cholesterol, and total:HDL cholesterol ratios between the intervention and control groups, we used 2-sample independent *t* tests. We conducted multiple logistic regression for our primary outcome to adjust for study site and any clinically important (> 10% imbalance between arms) or statistically significant (*p* < 0.1 between arms) baseline differences. Our primary analysis was intention-to-treat. Missing data at the 6 month follow-up assessment were imputed with a last-observation carried forward strategy; this approach conservatively assumes that all participants lost to follow-up had no change in their blood pressure or lipid levels after their last recorded value. | | | | 579 |
| **Notes** | None | | | | |

## Change in HDL cholesterol

| | Description as stated in report/paper | | | | | | Page number |
|---|---|---|---|---|---|---|---|
| **Outcome** | Change in HDL cholesterol | | | | | | 582 (table 2) |
| **Time point** *(specify from start or end of intervention)* | 6 months | | | | | | 582 (table 2) |
| **Results** | Intervention | | | Comparison | | | 582 (table 2) |
| | Mean change | SD *(or other variance, specify)* | No. participants | Mean change | SD *(or other variance, specify)* | No. participants | |

| | | | | | | | |
|---|---|---|---|---|---|---|---|
| | –0.01 | 0.23 | 143 | –0.04 | 0.19 | 136 | |
| **Any other results reported** *(e.g. mean difference, CI, P value)* | Difference (intervention – control), SD* (95% CI): 0.03 (–0.02 to 0.08) | | | | | | 582 (table 2) |
| **No. missing participants** | 33 | | | 26 | | | 579 (figure 1) |
| **Reasons missing** | • Declined further participation before 6 month visit: n=7<br>• Moved out of region: n=1<br>• Died: n=1<br>• Reason not specified: n=24 | | | • Did not receive allocated intervention: n=13<br>• Early withdrawal: n=18<br>• Reason not specified: n=2 | | | 579 (figure 1) |
| **Statistical methods used and appropriateness of these** *(e.g. adjustment for correlation)* | We used χ2 tests to compare the proportion of patients who attained optimal blood pressure and lipid control at 6 months (because the proportion at baseline was zero by design); we used χ2 testing for other binary secondary outcomes. To compare changes in systolic blood pressure, LDL cholesterol, and total:HDL cholesterol ratios between the intervention and control groups, we used 2-sample independent *t* tests. We conducted multiple logistic regression for our primary outcome to adjust for study site and any clinically important (> 10% imbalance between arms) or statistically significant (*p* < 0.1 between arms) baseline differences. Our primary analysis was intention-to-treat. Missing data at the 6 month follow-up assessment were imputed with a last-observation carried forward strategy; this approach conservatively assumes that all participants lost to follow-up had no change in their blood pressure or lipid levels after their last recorded value. | | | | | | 579 |
| **Notes** | None | | | | | | |

## Self-reported adherence of 75% or higher for blood pressure or lipid-lowering medications

| | Description as stated in report/paper | | | | Page number |
|---|---|---|---|---|---|
| **Outcome** | Medication adherence | | | | 582 (table 2) |
| **Time point** *(specify from start or end of intervention)* | 6 months | | | | 582 (table 2) |
| **Results** | Intervention | | Comparison | | 582 (table 2) |
| | No. with event | Total in group | No. with event | Total in group | |
| | 138 | 143 | 132 | 136 | |
| **Any other results reported** | Difference (intervention – control), SD* (95% CI): –0.6% (–4.3 to 3.7) | | | | 582 (table 2) |

| | | | |
|---|---|---|---|
| **No. missing participants** | 32 | 26 | 579 (figure 1) |
| **Reasons missing** | • Declined further participation before 6 month visit: n=7<br>• Moved out of region: n=1<br>• Reason not specified: n=24 | • Did not receive allocated intervention: n=13<br>• Early withdrawal: n=18<br>• Reason not specified: n=2 | 579 (figure 1) |
| **Statistical methods used and appropriateness of these** | We used χ2 tests to compare the proportion of patients who attained optimal blood pressure and lipid control at 6 months (because the proportion at baseline was zero by design); we used χ2 testing for other binary secondary outcomes. To compare changes in systolic blood pressure, LDL cholesterol, and total:HDL cholesterol ratios between the intervention and control groups, we used 2-sample independent *t* tests. We conducted multiple logistic regression for our primary outcome to adjust for study site and any clinically important (> 10% imbalance between arms) or statistically significant ($p < 0.1$ between arms) baseline differences. Our primary analysis was intention-to-treat. Missing data at the 6 month follow-up assessment were imputed with a last-observation carried forward strategy; this approach conservatively assumes that all participants lost to follow-up had no change in their blood pressure or lipid levels after their last recorded value. | | 579 |
| **Notes** | None | | |

## Overall self-rated health

| | Description as stated in report/paper | | | | | | Page number |
|---|---|---|---|---|---|---|---|
| **Outcome** | Self-rated health | | | | | | 582 (table 2) |
| **Time point** *(specify from start or end of intervention)* | 6 months | | | | | | 582 (table 2) |
| **Results** | Intervention | | | Comparison | | | 582 (table 2) |
| | Mean | SD *(or other variance, specify)* | No. participants | Mean | SD *(or other variance, specify)* | No. participants | |
| | 3.5 | 0.9 | 143 | 3.4 | 0.8 | 136 | |

| | | | |
|---|---|---|---|
| **Any other results reported** *(e.g. mean difference, CI, P value)* | Difference (intervention – control), SD* (95% CI):<br>0.1 (–0.1 to 0.3) | | 582 (table 2) |
| **No. missing participants** | 33 | 26 | 579 (figure 1) |
| **Reasons missing** | • Declined further participation before 6 month visit: n=7<br>• Moved out of region: n=1<br>• Died: n=1<br>• Reason not specified: n=24 | • Did not receive allocated intervention: n=13<br>• Early withdrawal: n=18<br>• Reason not specified: n=2 | 579 (figure 1) |
| **Statistical methods used and appropriateness of these** *(e.g. adjustment for correlation)* | We used χ2 tests to compare the proportion of patients who attained optimal blood pressure and lipid control at 6 months (because the proportion at baseline was zero by design); we used χ2 testing for other binary secondary outcomes. To compare changes in systolic blood pressure, LDL cholesterol, and total:HDL cholesterol ratios between the intervention and control groups, we used 2-sample independent *t* tests. We conducted multiple logistic regression for our primary outcome to adjust for study site and any clinically important (> 10% imbalance between arms) or statistically significant ($p < 0.1$ between arms) baseline differences. Our primary analysis was intention-to-treat. Missing data at the 6 month follow-up assessment were imputed with a last-observation carried forward strategy; this approach conservatively assumes that all participants lost to follow-up had no change in their blood pressure or lipid levels after their last recorded value. | | 579 |
| **Notes** | None | | |

## EQ-5D index score

| | Description as stated in report/paper | | | | | | Page number |
|---|---|---|---|---|---|---|---|
| **Outcome** | EQ-5D quality of life | | | | | | 582 (table 2) |
| **Time point** *(specify from start or end of intervention)* | 6 months | | | | | | 582 (table 2) |
| **Results** | Intervention | | | Comparison | | | 582 (table 2) |
| | Mean | SD *(or other variance, specify)* | No. participants | Mean | SD *(or other variance, specify)* | No. participants | |
| | 0.84 | 0.15 | 143 | 0.86 | 0.17 | 136 | |

| | | | |
|---|---|---|---|
| **Any other results reported** *(e.g. mean difference, CI, P value)* | Difference (intervention – control), SD* (95% CI): –0.02 (–0.06 to 0.02) | | 582 (table 2) |
| **No. missing participants** | 33 | 26 | 579 (figure 1) |
| **Reasons missing** | • Declined further participation before 6 month visit: n=7<br>• Moved out of region: n=1<br>• Died: n=1<br>• Reason not specified: n=24 | • Did not receive allocated intervention: n=13<br>• Early withdrawal: n=18<br>• Reason not specified: n=2 | 579 (figure 1) |
| **Statistical methods used and appropriateness of these** *(e.g. adjustment for correlation)* | We used χ2 tests to compare the proportion of patients who attained optimal blood pressure and lipid control at 6 months (because the proportion at baseline was zero by design); we used χ2 testing for other binary secondary outcomes. To compare changes in systolic blood pressure, LDL cholesterol, and total:HDL cholesterol ratios between the intervention and control groups, we used 2-sample independent *t* tests. We conducted multiple logistic regression for our primary outcome to adjust for study site and any clinically important (> 10% imbalance between arms) or statistically significant ($p < 0.1$ between arms) baseline differences. Our primary analysis was intention-to-treat. Missing data at the 6 month follow-up assessment were imputed with a last-observation carried forward strategy; this approach conservatively assumes that all participants lost to follow-up had no change in their blood pressure or lipid levels after their last recorded value. | | 579 |
| **Notes** | None | | |

## Conclusions

| | Description as stated in report/paper | Page number |
|---|---|---|
| **Key conclusions of study authors** | We found that a nurse-led case management program based on monthly evaluation of risk factors, patient counselling and feedback to primary care physicians improved control of key risk factors for stroke (hypertension and dyslipidemia) by 6 months. However, even greater improvements were seen among patients whose care was managed by a pharmacist case manager who was empowered to initiate and titrate medications to attain guideline-recommended targets. We believe that both approaches hold great promise, not only for patients with stroke or transient ischemic attack but also for all patients with, or at high risk of, vascular disease, and our study provides much-needed information on their comparative effectiveness | 583 |
| **Notes** | None | |

## McFarland et al. 2009

### Study eligibility

| Study Characteristics | Eligibility criteria |
|---|---|
| Title | Drug Use Evaluation of Sitagliptin Dosing by Pharmacist Versus Non-pharmacist Clinicians in an Internal Medicine Department of a Private Physician-Owned Multispecialty Clinic |
| Author (year) | McFarland et al. 2009 |
| Country | Tennessee, USA |
| Type of study | Retrospective cohort study |
| Participants | Patients with diabetes prescribed sitagliptin for renal function |
| Types of intervention | Pharmacist-directed diabetes disease management program |
| Types of comparison | Usual care |
| Types of outcome measures | Rates of inappropriate initial dosing |
| Prescriptive authority | Collaborative practice agreement |
| Include/Exclude | Include |
| Notes | None |

**DO NOT PROCEED IF STUDY EXCLUDED FROM REVIEW**

## Characteristics of included studies

### Methods

| | Descriptions as stated in report/paper | | Page number |
|---|---|---|---|
| **Aim of study** | To evaluate the prevalence of potentially inappropriate dosing in patients for whom sitagliptin was initiated by a pharmacist versus patients in whom sitagliptin was initiated by a non-pharmacist prescriber. | | 564 |
| **Design** | Retrospect cohort study | | 563 |
| **Unit of allocation** *(by individuals, cluster/ groups or body parts)* | Individuals | | 564 |
| **Start-end date** | October 2006 - June 2008 | | 564 |
| **Duration of participation** *(from recruitment to last follow-up/ baseline to last follow-up- group level)* *1. Time of consent until last measurement for each individual.* *2. Baseline to final follow-up for each individual* | Initial sitagliptin dosing | | 564 |
| **Study duration** *(as above with the exception of interim analyses or other circumstances* | Initial sitagliptin dosing | | 564 |
| **Ethical approval needed/obtained for study** | No | This study received exempt status from the institutional review board (IRB) of the University of Tennessee Health Science Center. | 564 |
| **Notes** | None | | |

### Participants

| | **Description** *Include comparative information for each intervention or comparison group if available* | **Page number** |
|---|---|---|

| | | | |
|---|---|---|---|
| **Population description** *(from which study participants are drawn)* | Patients with diabetes prescribed sitagliptin for renal function | | 564 |
| **Setting** *(including location and social context)* | Internal medicine department of a private physician-owned multispecialty clinic | | 563 |
| **Inclusion/exclusion criteria** | Only patients for whom sitagliptin was initiated by a provider in the department of internal medicine were included in this study. | | 564 |
| **Method of recruitment of participants** | Electronic health records | | |
| **Informed consent obtained** | Not applicable | This study received exempt status from the institutional review board (IRB) of the University of Tennessee Health Science Center. | 564 |
| **Total no. randomised** *(or total pop. at start of study for NRCTs)* | 290 | | 564 |
| **Clusters** | Na | | |
| **Baseline imbalances** | Not reported | | NA |
| **Withdrawals and exclusions** | None | | NA |
| **Age** | Not reported | | NA |
| **Sex (female)** | Not reported | | NA |
| **Subgroups measure** | Not reported | | NA |
| **Subgroups reported** | Not reported | | NA |
| **Notes** | None | | |

## Intervention group

| | Description as stated in report/paper | Page number |
|---|---|---|
| **Group name** | Pharmacist-directed diabetes disease management program | 564 |
| **No. randomised/assigned to group** *(specify whether no. people or clusters)* | 158 | 564 |
| **Description** *(include sufficient detail for replication, e.g. content, dose, components)* | The pharmacist, who worked under a collaborative practice agreement, was responsible for evaluation and treatment of diabetes and conditions associated with diabetes. The pharmacist had the authority to order laboratory tests, initiate medications, make referrals, and schedule follow-up when deemed necessary. | 564 |
| **Duration of treatment period** | Not reported | NA |

| | | |
|---|---|---|
| **Timing** | Not reported | NA |
| **Co-interventions** | Not reported | NA |
| **Notes** | None | |

## Comparator group

| | Description as stated in report/paper | Page number |
|---|---|---|
| **Group name** | Usual care | 564 |
| **No. randomised/assigned to group** *(specify whether no. people or clusters)* | 132 | 564 |
| **Description** *(include sufficient detail for replication, e.g. content, dose, components)* | Patients not seen in the diabetes disease management program received usual medical care for their type 2 diabetes from their non-pharmacist provider | 564 |
| **Duration of treatment period** | Not reported | NA |
| **Timing** | Not reported | NA |
| **Co-interventions** | Not reported | NA |
| **Notes** | None | |

## Outcomes

### Rates of inappropriate initial dosing

| | Description as stated in report/paper | | Page number |
|---|---|---|---|
| **Outcome name** | Rates of inappropriate initial dosing | | 565 (table 2) |
| **Time points measured** *(specify whether from start or end of intervention)* | Initial dosing point | | 564 |
| **Time points reported** | Initial dosing point | | 564 |
| **Outcome definition** *(with diagnostic criteria if relevant)* | Initial dosing of sitagliptin was defined as the first time an order for sitagliptin was entered in the electronic medical record. | | 564 |
| **Unit of measurement** *(if relevant)* | Event | | 564 |
| **Scales: upper and lower limits** *(indicate whether high or low score is good)* | Lower score is desired | | NA |
| **Is outcome/tool validated?** | No | NA | NA |

| | | |
|---|---|---|
| **Imputation of missing data** *(e.g. assumptions made for ITT analysis)* | Not reported | NA |
| **Assumed risk estimate** *(e.g. baseline or population risk noted in Background)* | Not reported | NA |
| **Power** *(e.g. power & sample size calculation, level of power achieved)* | Not reported | NA |
| **Notes** | None | |

## Funding/conflict of interest

| | | |
|---|---|---|
| **Study funding sources** *(including role of funders)* | There was no external funding for this research. | 566 |
| **Possible conflicts of interest** *(for study authors)* | This research was presented in part as a poster at the American College of Clinical Pharmacy meeting on April 24, 2009, in Orlando, Florida. | 566 |
| **Notes** | None | |

## Data and analysis

### Rates of inappropriate initial dosing

| | **Description as stated in report/paper** | | | | **Page number** |
|---|---|---|---|---|---|
| **Outcome** | Rates of inappropriate initial dosing | | | | 565 (table 2) |
| **Time point** *(specify from start or end of intervention)* | Initial dosing point | | | | 564 |
| **Results** | Intervention | | Comparison | | |
| | No. with event | Total in group | No. with event | Total in group | |
| | 1 | 158 | 34 | 132 | |
| **Any other results reported** | No | | | | NA |
| **No. missing participants** | 0 | | 0 | | 565 (table 2) |
| **Reasons missing** | NA | | NA | | NA |
| **Statistical methods used and appropriateness of these** | Data were extracted from the electronic medical records database. | | | | 564 |
| **Confounders** | Not reported | | | | NA |
| **Notes** | None | | | | |

## Conclusions

| | Description as stated in report/paper | Page number |
|---|---|---|
| **Key conclusions of study authors** | Based on assessment of renal function, pharmacist prescribing of the initial dose of sitagliptin was associated with 1 case (0.6%) of potentially inappropriate prescribing (under-dose) compared with 34 cases of potentially inappropriate prescribing (21 overdose and 13 under-dose) by nonpharmacists. The magnitude of the threat to patient safety or other clinical outcomes from this potentially inappropriate initial prescribing of sitagliptin is unknown. | 563 |
| **Notes** | None | |

# Morello et al. 2016

## Study eligibility

| **Study Characteristics** | **Eligibility criteria** |
|---|---|
| **Title** | Clinical outcomes associated with a collaborative pharmacist-endocrinologist diabetes intense medical management “Tune Up” clinic in complex patients |
| **Author (year)** | Morello et al. (2016) |
| **Country** | California, USA |
| **Type of study** | Retrospective cohort study |
| **Participants** | Study participants >18 years old, diagnosed with Type 2 diabetes with an A1C ≥8% |
| **Types of intervention** | Pharmacist-endocrinologist managed |
| **Types of comparison** | Usual care |
| **Types of outcome measures** | Mean change in A1C at 6 months, fasting blood glucose; LDL, HDL, triglycerides, (systolic and diastolic) blood pressure |
| **Prescriptive authority** | Collaborative practice agreement |
| **Include/Exclude** | Include |
| **Notes** | None |

**DO NOT PROCEED IF STUDY EXCLUDED FROM REVIEW**

## Characteristics of included studies

### Methods

| | Descriptions as stated in report/paper | | Page number |
|---|---|---|---|
| **Aim of study** | The primary study objective was to assess mean change in A1C at 6 months after the baseline visit in the DIMM clinic and compare this group with a similar comparator group of T2DM patients who were not referred to the DIMM clinic. The secondary objective was to compare other metabolic parameters (fasting blood glucose [FBG], weight, body mass index [BMI], LDL, high density lipoprotein [HDL], triglycerides [TG], and BP) between groups. | | 9 |
| **Design** | Retrospective cohort study | | 9 |
| **Unit of allocation** *(by individuals, cluster/ groups or body parts)* | Individuals | | 9 |
| **Start-end date** | April 2009 - November 2013. | | 9 |
| **Duration of participation** *(from recruitment to last follow-up/ baseline to last follow-up- group level)* *1. Time of consent until last measurement for each individual.* *2. Baseline to final follow-up for each individual* | Patients were treated for a limited time (target 6 months), then discharged back to their PCP when metabolic goals were achieved; otherwise, patients remained in the DIMM clinic until their goals were met. | | 9 |
| **Study duration** *(as above with the exception of interim analyses or other circumstances* | Six months | | 9 |
| **Ethical approval needed/obtained for study** | Yes | This study was approved by the VASDHS Institutional Review Board for the study of human subjects. | 10 |
| **Notes** | None | | |

### Participants

| | Description<br>*Include comparative information for each intervention or comparison group if available* | | Page number |
|---|---|---|---|
| **Population description**<br>*(from which study participants are drawn)* | Study participants >18 years old, diagnosed with Type 2 diabetes with an A1C ≥8% | | 9 |
| **Setting**<br>*(including location and social context)* | VA diabetes intensive medical management (DIMMS) clinc | | 9 |
| **Inclusion/exclusion criteria** | Study participants in both groups met the inclusion criteria of >18 years old, diagnosed with T2DM, A1C ≥8% at initial (or index) visit, and follow-up visit within 6 months. Patients excluded from the study were those with type 1 diabetes mellitus, a baseline A1C <8%, and/or no follow-up visit data within 6 months. In addition, patients in the comparator group were required to have a visit (index visit) to their PCP within the first 6 months of the DIMM clinic operation (April through October 2009). This index visit was used to determine the beginning of a 6-month treatment period. | | 9 |
| **Method of recruitment of participants** | Electronic medical record | | 9 |
| **Informed consent obtained** | Not reported | NA | NA |
| **Total no. randomised**<br>*(or total pop. at start of study for NRCTs)* | 155 | | 11 (table 1) |
| **Clusters** | NA | | NA |
| **Baseline imbalances** | There were no significant differences in the demographics of each group; the majority was male, non-Hispanic, and white, with a mean age of 62 years (Table 1). Clinical parameters were similar between groups, except that the DIMM group had higher baseline mean A1C (%) than the comparator group (10.5 [SD = 1.6] vs 9.7 [SD = 1.6] *P* = 0.002). The DIMM group had approximately 1 more comorbid condition than the PCP group (8.4 [SD = 2.4] vs 7.5 [SD = 2.0]; *P* = 0.02), although CCI scores (a method of predicting mortality risk based on presence of comorbidities) did not differ significantly between groups (Table 2). The proportion of patients with comorbidities, the mean number of prescription medications, and level of medication regimen complexity were similar between groups (Table 2). | | 11 |
| **Withdrawals and exclusions** | None | | NA |

| | | |
|---|---|---|
| **Age (years)** | Intervention: 62.2 ± 8.1<br>Comparator: 62.4 ± 10.0 | 13 (table 3) |
| **Sex (female)** | Intervention: 2.0%<br>Comparator: 3.6% | 13 (table 3) |
| **Subgroups measure** | Not reported | NA |
| **Subgroups reported** | Not reported | NA |
| **Notes** | None | |

## Intervention group

| | **Description as stated in report/paper** | **Page number** |
|---|---|---|
| **Group name** | Pharmacist-endocrinologist managed | 9 |
| **No. randomised/assigned to group** *(specify whether no. people or clusters)* | 99 individuals | 11 (table 1) |
| **Description** *(include sufficient detail for replication, e.g. content, dose, components)* | The pharmacist had full laboratory ordering and prescribing authority to initiate, adjust, monitor, or discontinue medication therapy for diabetes and all related conditions (eg, hypertension, lipids, hypothyroidism, and diabetic neuropathy) to create an individualized care plan for each patient. The collaborative approach and scope of practice required that all care plans created for new patients were presented to the endocrinologist prior to implementing. This requirement was not deemed necessary for follow-up patient visits. Located in the same clinic area, the endocrinologist was available should the pharmacist identify patients who needed acute symptom evaluation or a new diagnosis (eg, hypothyroidism, cellulitis). | 10 |
| **Duration of treatment period** | Patients were treated for a limited time (target 6 months), then discharged back to their PCP when metabolic goals were achieved; otherwise, patients remained in the DIMM clinic until their goals were met. | 9 |
| **Timing** | About every 2 to 3 months, 60-minute visits were scheduled… If needed, patient-initiated 10- to 15- minute follow-up phone calls were scheduled<br><br>During the 6-month period, the number of DIMM clinic visits ranged from 2 to 4, with a mean (SD) of 3.1 (0.5) for the DIMM group. | 10, 11 |

| | | |
|---|---|---|
| **Co-interventions** | The comprehensive educational component of the one-on-one visit was individualized. Patients received relevant education on diabetes (ie, disease state, target goals, complications, and the potential to reverse and prevent them with diabetes control, hypoglycemia prevention, and treatment). All other education was very patient specific; components could include pattern management, foot care–specific nutrition tips and food exchanges that promote a healthier diet, tips on dietary fiber and timing with meals, food choices for eating out in restaurants, fun and achievable activities such as biking, dancing, swimming, and easy weight lifting strategies. | 10 |
| **Notes** | None | |

## Comparator group

| | **Description as stated in report/paper** | **Page number** |
|---|---|---|
| **Group name** | Usual care (primary care provider) | 11 (table 1) |
| **No. randomised/assigned to group** *(specify whether no. people or clusters)* | 56 individuals | 11 |
| **Description** *(include sufficient detail for replication, e.g. content, dose, components)* | Usual care by primary care providers | 9 |
| **Duration of treatment period** | Six months | 9 |
| **Timing** | Each patient had at least 2 visits during the 6 month period<br><br>It was not possible to determine the exact number of provider visits for diabetes care in the PCP group or any additional (non-DIMM) provider visits for diabetes care in the DIMM group because complex patients such as those in this study often have their diabetes care evaluated within specialty clinics such as renal and cardiovascular clinics. | 11 |
| **Co-interventions** | None | NA |
| **Notes** | None | |

## Outcomes

## Mean change in A1C at 6 months

| | **Description as stated in report/paper** | **Page number** |
|---|---|---|

| | | | |
|---|---|---|---|
| **Outcome name** | Mean change in A1C at 6 months | | 9 |
| **Time points measured** *(specify whether from start or end of intervention)* | Baseline; three months; six months | | 13 (table 3) |
| **Time points reported** | Six months | | 13 (table 3) |
| **Outcome definition** *(with diagnostic criteria if relevant)* | Blood glucose levels | | 9 |
| **Unit of measurement** *(if relevant)* | % | | 13 (table 3) |
| **Scales: upper and lower limits** *(indicate whether high or low score is good)* | Lower percentage is desired | | NA |
| **Is outcome/tool validated?** | No | Bloodwork | NA |
| **Imputation of missing data** *(e.g. assumptions made for ITT analysis)* | The last observation carried forward approach was used to impute missing values at 3 and 6 months. | | 10 |
| **Assumed risk estimate** *(e.g. baseline or population risk noted in Background)* | assuming DIMM mean (SD) change = −2.4(1.5) and comparator group mean (SD) = −1.5 (1.0), a 2-sided test of significance, and α = 0.05. | | 10 |
| **Power** *(e.g. power & sample size calculation, level of power achieved)* | A target sample size of 43 patients in each group was estimated as sufficient to detect a difference between the mean change (baseline vs 6 months) in A1C (%) between groups with 90% power, assuming DIMM mean (SD) change = −2.4(1.5) and comparator group mean (SD) = −1.5 (1.0), a 2-sided test of significance, and α = 0.05. STATA | | 10 |
| **Notes** | None | | |

## Fasting blood glucose

| | **Description as stated in report/paper** | **Page number** |
|---|---|---|
| **Outcome name** | Fasting blood glucose | 9 |
| **Time points measured** *(specify whether from start or end of intervention)* | Baseline; three months; six months | 13 (table 3) |
| **Time points reported** | Six months | 13 (table 3) |
| **Outcome definition** *(with diagnostic criteria if relevant)* | Blood glucose levels after fasting | 9 |

| | | | |
|---|---|---|---|
| **Unit of measurement** *(if relevant)* | % | | 13 (table 3) |
| **Scales: upper and lower limits** *(indicate whether high or low score is good)* | Lower percentage is desired | | NA |
| **Is outcome/tool validated?** | No | Bloodwork | NA |
| **Imputation of missing data** *(e.g. assumptions made for ITT analysis)* | The last observation carried forward approach was used to impute missing values at 3 and 6 months. | | 10 |
| **Assumed risk estimate** *(e.g. baseline or population risk noted in Background)* | Not reported | | NA |
| **Power** *(e.g. power & sample size calculation, level of power achieved)* | A target sample size of 43 patients in each group was estimated as sufficient to detect a difference between the mean change (baseline vs 6 months) in A1C (%) between groups with 90% power, assuming DIMM mean (SD) change = −2.4(1.5) and comparator group mean (SD) = −1.5 (1.0), a 2-sided test of significance, and α = 0.05. STATA | | 10 |
| **Notes** | None | | |

## LDL cholesterol

| | **Description as stated in report/paper** | | **Page number** |
|---|---|---|---|
| **Outcome name** | LDL cholesterol | | 9 |
| **Time points measured** *(specify whether from start or end of intervention)* | Baseline; three months; six months | | 13 (table 3) |
| **Time points reported** | Six months | | 13 (table 3) |
| **Outcome definition** *(with diagnostic criteria if relevant)* | Low density lipoprotein | | 9 |
| **Unit of measurement** *(if relevant)* | mg/DL | | 13 (table 3) |
| **Scales: upper and lower limits** *(indicate whether high or low score is good)* | Lower percentage is desired | | NA |
| **Is outcome/tool validated?** | No | Bloodwork | NA |

| | | |
|---|---|---|
| **Imputation of missing data** *(e.g. assumptions made for ITT analysis)* | The last observation carried forward approach was used to impute missing values at 3 and 6 months. | 10 |
| **Assumed risk estimate** *(e.g. baseline or population risk noted in Background)* | Not reported | NA |
| **Power** *(e.g. power & sample size calculation, level of power achieved)* | A target sample size of 43 patients in each group was estimated as sufficient to detect a difference between the mean change (baseline vs 6 months) in A1C (%) between groups with 90% power, assuming DIMM mean (SD) change = −2.4(1.5) and comparator group mean (SD) = −1.5 (1.0), a 2-sided test of significance, and α = 0.05. STATA | 10 |
| **Notes** | None | |

## HDL cholesterol

| | Description as stated in report/paper | | Page number |
|---|---|---|---|
| **Outcome name** | HDL cholesterol | | 9 |
| **Time points measured** *(specify whether from start or end of intervention)* | Baseline; three months; six months | | 13 (table 3) |
| **Time points reported** | Six months | | 13 (table 3) |
| **Outcome definition** *(with diagnostic criteria if relevant)* | High density lipoprotein | | 9 |
| **Unit of measurement** *(if relevant)* | mg/DL | | 13 (table 3) |
| **Scales: upper and lower limits** *(indicate whether high or low score is good)* | Lower percentage is desired | | NA |
| **Is outcome/tool validated?** | No | Bloodwork | NA |
| **Imputation of missing data** *(e.g. assumptions made for ITT analysis)* | The last observation carried forward approach was used to impute missing values at 3 and 6 months. | | 10 |
| **Assumed risk estimate** *(e.g. baseline or population risk noted in Background)* | Not reported | | NA |

| | | | |
|---|---|---|---|
| **Power** *(e.g. power & sample size calculation, level of power achieved)* | A target sample size of 43 patients in each group was estimated as sufficient to detect a difference between the mean change (baseline vs 6 months) in A1C (%) between groups with 90% power, assuming DIMM mean (SD) change = −2.4(1.5) and comparator group mean (SD) = −1.5 (1.0), a 2-sided test of significance, and α = 0.05. STATA | | 10 |
| **Notes** | None | | |

## Triglycerides

| | **Description as stated in report/paper** | | **Page number** |
|---|---|---|---|
| **Outcome name** | Triglycerides | | 9 |
| **Time points measured** *(specify whether from start or end of intervention)* | Baseline; three months; six months | | 13 (table 3) |
| **Time points reported** | Six months | | 13 (table 3) |
| **Outcome definition** *(with diagnostic criteria if relevant)* | Triglycerides | | 9 |
| **Unit of measurement** *(if relevant)* | mg/DL | | 13 (table 3) |
| **Scales: upper and lower limits** *(indicate whether high or low score is good)* | Lower percentage is desired | | NA |
| **Is outcome/tool validated?** | No | Bloodwork | NA |
| **Imputation of missing data** *(e.g. assumptions made for ITT analysis)* | The last observation carried forward approach was used to impute missing values at 3 and 6 months. | | 10 |
| **Assumed risk estimate** *(e.g. baseline or population risk noted in Background)* | Not reported | | NA |
| **Power** *(e.g. power & sample size calculation, level of power achieved)* | A target sample size of 43 patients in each group was estimated as sufficient to detect a difference between the mean change (baseline vs 6 months) in A1C (%) between groups with 90% power, assuming DIMM mean (SD) change = −2.4(1.5) and comparator group mean (SD) = −1.5 (1.0), a 2-sided test of significance, and α = 0.05. STATA | | 10 |
| **Notes** | None | | |

## Systolic blood pressure

| | **Description as stated in report/paper** | **Page number** |
|---|---|---|

| | | | |
|---|---|---|---|
| **Outcome name** | Systolic blood pressure | | 9 |
| **Time points measured** *(specify whether from start or end of intervention)* | Baseline; three months; six months | | 13 (table 3) |
| **Time points reported** | Six months | | 13 (table 3) |
| **Outcome definition** *(with diagnostic criteria if relevant)* | Systolic blood pressure | | 9 |
| **Unit of measurement** *(if relevant)* | mm hg | | 13 (table 3) |
| **Scales: upper and lower limits** *(indicate whether high or low score is good)* | Lower value is desired | | NA |
| **Is outcome/tool validated?** | No | Bloodwork | NA |
| **Imputation of missing data** *(e.g. assumptions made for ITT analysis)* | The last observation carried forward approach was used to impute missing values at 3 and 6 months. | | 10 |
| **Assumed risk estimate** *(e.g. baseline or population risk noted in Background)* | Not reported | | NA |
| **Power** *(e.g. power & sample size calculation, level of power achieved)* | A target sample size of 43 patients in each group was estimated as sufficient to detect a difference between the mean change (baseline vs 6 months) in A1C (%) between groups with 90% power, assuming DIMM mean (SD) change = −2.4(1.5) and comparator group mean (SD) = −1.5 (1.0), a 2-sided test of significance, and α = 0.05. STATA | | 10 |
| **Notes** | None | | |

## Diastolic blood pressure

| | Description as stated in report/paper | Page number |
|---|---|---|
| **Outcome name** | Diastolic blood pressure | 9 |
| **Time points measured** *(specify whether from start or end of intervention)* | Baseline; three months; six months | 13 (table 3) |
| **Time points reported** | Six months | 13 (table 3) |
| **Outcome definition** *(with diagnostic criteria if relevant)* | Diastolic blood pressure | 9 |

| | | | |
|---|---|---|---|
| **Unit of measurement** *(if relevant)* | mm hg | | 13 (table 3) |
| **Scales: upper and lower limits** *(indicate whether high or low score is good)* | Lower value is desired | | NA |
| **Is outcome/tool validated?** | No | Bloodwork | NA |
| **Imputation of missing data** *(e.g. assumptions made for ITT analysis)* | The last observation carried forward approach was used to impute missing values at 3 and 6 months. | | 10 |
| **Assumed risk estimate** *(e.g. baseline or population risk noted in Background)* | Not reported | | NA |
| **Power** *(e.g. power & sample size calculation, level of power achieved)* | A target sample size of 43 patients in each group was estimated as sufficient to detect a difference between the mean change (baseline vs 6 months) in A1C (%) between groups with 90% power, assuming DIMM mean (SD) change = −2.4(1.5) and comparator group mean (SD) = −1.5 (1.0), a 2-sided test of significance, and α = 0.05. STATA | | 10 |
| **Notes** | None | | |

## Funding/conflict of interest

| | | |
|---|---|---|
| **Study funding sources** *(including role of funders)* | The authors received no financial support for the research, authorship, and/or publication of this article. | 15 |
| **Possible conflicts of interest** *(for study authors)* | The authors declared no potential conflicts of interest with respect to the research, authorship, and/or publication of this article. | 15 |
| **Notes** | None | |

## Data and analysis

### Mean change in A1C at 6 months

| | **Description as stated in report/paper** | | **Page number** |
|---|---|---|---|
| **Outcome** | Mean change in A1C at 6 months | | 9 |
| **Time point** *(specify from start or end of intervention)* | Six months | | 13 (table 3) |
| **Results** | Intervention | Comparison | 13 (table 3) |

| | | | | | | | |
|---|---|---|---|---|---|---|---|
| | Mean | SD *(or other variance, specify)* | No. participants | Mean | SD *(or other variance, specify)* | No. participants | |
| | 8.2 | 1.9 | 99 | 9.0 | 1.5 | 56 | |
| **Any other results reported** *(e.g. mean difference, CI, P value)* | The DIMM and PCP groups achieved significant improvements in A1C (%) over the baseline and 3- and 6-month periods (Table 3): DIMM: 10.5 (SD = 1.6), 8.9 (SD = 1.9), 8.2 (SD = 1.8); PCP: 9.7 (SD = 1.6), 9.0 (SD = 1.2), 9.0 (SD = 1.5), *P* < 0.001.<br><br>After controlling for baseline A1C, lipid profile, weight, BMI, and age, patients in the DIMM group were associated with an additional 0.8 percentage point improvement in A1C ($R^2$ = 0.43) | | | | | | 11 |
| **No. missing participants** | 0 | | | 0 | | | NA |
| **Statistical methods used and appropriateness of these** *(e.g. adjustment for correlation)* | The last observation carried forward approach was used to impute missing values at 3 and 6 months. Mean values at baseline and 3 and 6 months were compared using repeated-measures ANOVA within groups. Difference in mean change from baseline between DIMM and PCP groups was evaluated using *t* tests. Linear regression analysis was used to investigate the sensitivity of results to baseline differences between groups. | | | | | | 10 |
| **Confounders** | Baseline A1C, lipid profile, weight, BMI, and age | | | | | | |
| **Notes** | None | | | | | | |

## Fasting blood glucose

| | **Description as stated in report/paper** | | | | | | **Page number** |
|---|---|---|---|---|---|---|---|
| **Outcome** | Fasting blood glucose | | | | | | 9 |
| **Time point** *(specify from start or end of intervention)* | Six months | | | | | | 13 (table 3) |
| **Results** | Intervention | | | Comparison | | | 13 (table 3) |
| | Mean | SD *(or other variance, specify)* | No. participants | Mean | SD *(or other variance, specify)* | No. participants | |
| | 159.0 | 83.2 | 99 | 194.3 | 112.0 | 55 | |
| **Any other results reported** *(e.g. mean difference, CI, P value)* | Mean change (SD): Intervention -63.8 (10.29) vs. Control -28.1 (137.5); p=0.08 | | | | | | 13 (table 3) |

| | | | |
|---|---|---|---|
| **No. missing participants** | 0 | 1 | 13 (table 3) |
| **Statistical methods used and appropriateness of these** *(e.g. adjustment for correlation)* | The last observation carried forward approach was used to impute missing values at 3 and 6 months. Mean values at baseline and 3 and 6 months were compared using repeated-measures ANOVA within groups. Difference in mean change from baseline between DIMM and PCP groups was evaluated using *t* tests. Linear regression analysis was used to investigate the sensitivity of results to baseline differences between groups. | | 10 |
| **Confounders** | Baseline A1C, lipid profile, weight, BMI, and age | | 10 |
| **Notes** | None | | |

## LDL cholesterol

| | **Description as stated in report/paper** | | | | | | **Page number** |
|---|---|---|---|---|---|---|---|
| **Outcome** | LDL cholesterol | | | | | | 9 |
| **Time point** *(specify from start or end of intervention)* | Six months | | | | | | 13 (table 3) |
| **Results** | Intervention | | | Comparison | | | 13 (table 3) |
| | Mean | SD *(or other variance, specify)* | No. participants | Mean | SD *(or other variance, specify)* | No. participants | |
| | 84.0 | 28.6 | 99 | 82.8 | 32.0 | 51 | |
| **Any other results reported** *(e.g. mean difference, CI, P value)* | Mean change (SD): Intervention -6.5 (37.3) vs. Control -10.5 (38.3); p=0.58 | | | | | | 13 (table 3) |
| **No. missing participants** | 0 | | | 5 | | | 13 (table 3) |
| **Statistical methods used and appropriateness of these** *(e.g. adjustment for correlation)* | The last observation carried forward approach was used to impute missing values at 3 and 6 months. Mean values at baseline and 3 and 6 months were compared using repeated-measures ANOVA within groups. Difference in mean change from baseline between DIMM and PCP groups was evaluated using *t* tests. Linear regression analysis was used to investigate the sensitivity of results to baseline differences between groups. | | | | | | 10 |
| **Confounders** | Baseline A1C, lipid profile, weight, BMI, and age | | | | | | 10 |
| **Notes** | None | | | | | | |

## HDL cholesterol

| | **Description as stated in report/paper** | **Page number** |
|---|---|---|

| | | | | | | | |
|---|---|---|---|---|---|---|---|
| **Outcome** | HDL cholesterol | | | | | | 9 |
| **Time point** *(specify from start or end of intervention)* | Six months | | | | | | 13 (table 3) |
| **Results** | Intervention | | | Comparison | | | 13 (table 3) |
| | Mean | SD *(or other variance, specify)* | No. participants | Mean | SD *(or other variance, specify)* | No. participants | |
| | 40.7 | 11.8 | 99 | 42.9 | 12.8 | 51 | |
| **Any other results reported** *(e.g. mean difference, CI, P value)* | Mean change (SD): Intervention -0.8 (8.0) vs. Control -0.1 (7.3); p=0.57 | | | | | | 13 (table 3) |
| **No. missing participants** | 0 | | | 5 | | | 13 (table 3) |
| **Statistical methods used and appropriateness of these** *(e.g. adjustment for correlation)* | The last observation carried forward approach was used to impute missing values at 3 and 6 months. Mean values at baseline and 3 and 6 months were compared using repeated-measures ANOVA within groups. Difference in mean change from baseline between DIMM and PCP groups was evaluated using *t* tests. Linear regression analysis was used to investigate the sensitivity of results to baseline differences between groups. | | | | | | 10 |
| **Confounders** | Baseline A1C, lipid profile, weight, BMI, and age | | | | | | 10 |
| **Notes** | None | | | | | | |

## Triglycerides

| | **Description as stated in report/paper** | | | | | | **Page number** |
|---|---|---|---|---|---|---|---|
| **Outcome** | Triglycerides | | | | | | 9 |
| **Time point** *(specify from start or end of intervention)* | Six months | | | | | | 13 (table 3) |
| **Results** | Intervention | | | Comparison | | | 13 (table 3) |
| | Mean | SD *(or other variance, specify)* | No. participants | Mean | SD *(or other variance, specify)* | No. participants | |
| | 185.9 | 125.2 | 99 | 189.2 | 164.5 | 43 | |

| | | | |
|---|---|---|---|
| **Any other results reported** *(e.g. mean difference, CI, P value)* | Mean change (SD): Intervention -80.4 (218.5) vs. Control -41.4 (167.2); p=0.33 | | 13 (table 3) |
| **No. missing participants** | 0 | 12 | 13 (table 3) |
| **Statistical methods used and appropriateness of these** *(e.g. adjustment for correlation)* | The last observation carried forward approach was used to impute missing values at 3 and 6 months. Mean values at baseline and 3 and 6 months were compared using repeated-measures ANOVA within groups. Difference in mean change from baseline between DIMM and PCP groups was evaluated using *t* tests. Linear regression analysis was used to investigate the sensitivity of results to baseline differences between groups. | | 10 |
| **Confounders** | Baseline A1C, lipid profile, weight, BMI, and age | | 10 |
| **Notes** | None | | |

## Systolic blood pressure

| | **Description as stated in report/paper** | | | | | | **Page number** |
|---|---|---|---|---|---|---|---|
| **Outcome** | Systolic blood pressure | | | | | | 9 |
| **Time point** *(specify from start or end of intervention)* | Six months | | | | | | 13 (table 3) |
| **Results** | Intervention | | | Comparison | | | 13 (table 3) |
| | Mean | SD *(or other variance, specify)* | No. participants | Mean | SD *(or other variance, specify)* | No. participants | |
| | 127.0 | 14.4 | 99 | 136.7 | 20.0 | 56 | |
| **Any other results reported** *(e.g. mean difference, CI, P value)* | Mean change (SD): Intervention -3.5 (17.2) vs. Control 1.7 (21.8); p=0.11 | | | | | | 13 (table 3) |
| **No. missing participants** | 0 | | | 0 | | | 13 (table 3) |

| | | |
|---|---|---|
| **Statistical methods used and appropriateness of these** *(e.g. adjustment for correlation)* | The last observation carried forward approach was used to impute missing values at 3 and 6 months. Mean values at baseline and 3 and 6 months were compared using repeated-measures ANOVA within groups. Difference in mean change from baseline between DIMM and PCP groups was evaluated using *t* tests. Linear regression analysis was used to investigate the sensitivity of results to baseline differences between groups. | 10 |
| **Confounders** | Baseline A1C, lipid profile, weight, BMI, and age | 10 |
| **Notes** | None | |

## Diastolic blood pressure

| | Description as stated in report/paper | | | | | | Page number |
|---|---|---|---|---|---|---|---|
| **Outcome** | Diastolic blood pressure | | | | | | 9 |
| **Time point** *(specify from start or end of intervention)* | Six months | | | | | | 13 (table 3) |
| **Results** | Intervention | | | Comparison | | | 13 (table 3) |
| | Mean | SD *(or other variance, specify)* | No. participants | Mean | SD *(or other variance, specify)* | No. participants | |
| | 71.8 | 13.1 | 99 | 74.5 | 14.9 | 56 | |
| **Any other results reported** *(e.g. mean difference, CI, P value)* | Mean change (SD): Intervention -3.6 (11.4) vs. Control -2.5 (15.1); p=0.59 | | | | | | 13 (table 3) |
| **No. missing participants** | 0 | | | 0 | | | 13 (table 3) |
| **Statistical methods used and appropriateness of these** *(e.g. adjustment for correlation)* | The last observation carried forward approach was used to impute missing values at 3 and 6 months. Mean values at baseline and 3 and 6 months were compared using repeated-measures ANOVA within groups. Difference in mean change from baseline between DIMM and PCP groups was evaluated using *t* tests. Linear regression analysis was used to investigate the sensitivity of results to baseline differences between groups. | | | | | | 10 |
| **Confounders** | Baseline A1C, lipid profile, weight, BMI, and age | | | | | | 10 |
| **Notes** | None | | | | | | |

## Conclusions

| | Description as stated in report/paper | Page number |
|---|---|---|

| | | |
|---|---|---|
| **Key conclusions of study authors** | The PharmD-Endocrinologist collaborative DIMM "tune up" clinic approach is a successful model of approximately three60-minute pharmacist visits combining patient-centered clinical care with real-time diabetes education at the same clinic visit. In 6 months, complex T2DM patients achieved a mean reduction in A1C of 2.4%, without weight gain and with low incidence of hypoglycemia. Approximately three-quarters met the VA performance measure goal of A1C of <9%. FBG and TG improvements were also significant. The DIMM clinic represents an advanced, pharmacy practice model, with proven clinical and quality improvement outcomes versus a comparator group, in patients with T2DM and high medication and comorbid complexity. | 15 |
| **Notes** | None | |

## Ogilve et al. 2022

### Study eligibility

| **Study Characteristics** | **Eligibility criteria** |
|---|---|
| **Title** | An evaluation of a collaborative pharmacist prescribing model compared to the usual medical prescribing model in the emergency department |
| **Author (year)** | Ogilve et al. (2022) |
| **Country** | Queensland, Australia |
| **Type of study** | RCT |
| **Participants** | All adult patients referred for medical admission from emergency into the hospital |
| **Types of intervention** | Pharmacist collaborative prescribing |
| **Types of comparison** | Medical practitioner prescribing |
| **Types of outcome measures** | Prescribing errors, adverse drug reaction documentation, slow release medication documentation errors, medication chart reconciliation, chart completeness |
| **Prescriptive authority** | Collaborative practice agreement |
| **Include/Exclude** | Include |
| **Notes** | None |

**DO NOT PROCEED IF STUDY EXCLUDED FROM REVIEW**

## Characteristics of included studies

### Methods

| | Descriptions as stated in report/paper | | Page number |
|---|---|---|---|
| **Aim of study** | An evaluation of a collaborative pharmacist prescribing model compared to the usual medical prescribing model in the emergency department. | | 3745 |
| **Design** | RCT | | 3745 |
| **Unit of allocation** *(by individuals, cluster/ groups or body parts)* | Individuals | | 3745 |
| **Start-end date** | October 2016 - September 2017 | | 3745 |
| **Duration of participation** *(from recruitment to last follow-up/ baseline to last follow-up- group level) 1. Time of consent until last measurement for each individual. 2. Baseline to final follow-up for each individual* | The retrospective chart audits and comparison of the two groups were conducted as a snapshot, at the point of admission in ED, between the control and intervention groups. Even though the NIMC was continued for the rest of the inpatient stay, the audit was not continued beyond the admission period. | | 3746 |
| **Study duration** *(as above with the exception of interim analyses or other circumstances* | The retrospective chart audits and comparison of the two groups were conducted as a snapshot, at the point of admission in ED, between the control and intervention groups. Even though the NIMC was continued for the rest of the inpatient stay, the audit was not continued beyond the admission period. | | 3746 |
| **Ethical approval needed/obtained for study** | Yes | Ethical approval for this project was granted on October 15, 2015 by the Metro South Human Research Ethics Committee (HREC/15/QPAH/ 381). | 3747 |
| **Notes** | None | | |

### Participants

| | **Description** *Include comparative information for each intervention or comparison group if available* | Page number |
|---|---|---|

| | | | |
|---|---|---|---|
| **Population description** *(from which study participants are drawn)* | All adult patients referred for medical admission from emergency into the hospital were considered for participation in the study. The Emergency Department Information System (EDIS) was used to identify any patients waiting for admission. | | 3745 |
| **Setting** *(including location and social context)* | In an emergency department of a medium size (170 bed) metropolitan hospital in Australia. | | 3745 |
| **Inclusion/exclusion criteria** | Adult patients were excluded if they were unwilling or unable (did not have capacity) to give informed consent; not proficient in English and would require an interpreter; being transferred from ED to other hospitals; were critically ill (clinically unstable, intubated and on assisted ventilation, intensive monitoring, reduced consciousness, severe anaphylaxis etc.); specialised patient groups (mental health/psychiatric, paediatric, obstetric, oncology, surgical, cognitively impaired); or brought in by police who may be involved in illegal activity, aggressive or under the influence of recreational drugs and/or alcohol; and patients who already had all their medications charted by an ED medical practitioner by the time a referral was made for admission. | | 3745 |
| **Method of recruitment of participants** | The referral window for medical admission for the study was scheduled Monday to Friday between 7.30am and 2pm. This time restriction was due to the researcher's availability, considering normal working hours. Normal working hours were from 7.30am to 5pm but recruitment of patients ended at 2pm to allow enough time for the necessary tasks like consent, randomisation, collaboration and prescribing to occur. The trial commenced with the start of a new intake of admitting medical practitioners, in October 2016, and continued through with the intake of a second cohort of admitting medical practitioners in January 2017. The researcher was responsible for recruiting participants into the trial throughout this time, in addition to performing regular clinical pharmacist duties. The researcher did not perform prescribing and regular clinical pharmacist duties on the same patient. The end of recruitment aligned with the end of the medical practitioner rotations in September 2017. | | 3745 |
| **Informed consent obtained** | Yes | Adult patients were excluded if they were unwilling or unable (did not have capacity) to give informed consent | 3745 |
| **Total no. randomised** *(or total pop. at start of study for NRCTs)* | 94 individuals | | 3747 |
| **Clusters** | NA | | NA |

| | | |
|---|---|---|
| **Baseline imbalances** | Not reported | NA |
| **Withdrawals and exclusions** | 21 individuals<br>*Reasons for exclusion after recruitment include medications inadvertently prescribed by medical practitioner for an intervention patient, patients discharged from ED by medical practitioner, patients transferred to Hospital in the Home (HITH) or patients transferred to another hospital directly from ED. | 3748 (figure 2) |
| **Age (years)** | Intervention: 69.8 (range 44-89)<br>Comparator: 70.9 (range 40-89) | 3748 (table 1) |
| **Sex (female)** | Intervention: 47.4%<br>Comparator: 40.0% | 3748 (table 1) |
| **Subgroups measure** | Not reported | NA |
| **Subgroups reported** | Not reported | NA |
| **Notes** | None | |

## Intervention group

| | **Description as stated in report/paper** | **Page number** |
|---|---|---|
| **Group name** | Pharmacist collaborative prescribing | 3745 |
| **No. randomised/assigned to group** *(specify whether no. people or clusters)* | 46 individuals | 3748 (figure 2) |
| **Description** *(include sufficient detail for replication, e.g. content, dose, components)* | Participants assigned to the intervention group were seen by a pharmacist in ED, (not always the prescribing pharmacist). After seeing the patient, the admitting medical practitioner developed a collaborative plan for optimising the medications together with the prescribing pharmacist. This collaborative plan was documented in the progress notes by the pharmacist prescriber, who then prescribed the medications on the NIMC, including VTE prophylaxis assessment, whilst the patient was still in ED. The scope of prescribing involved withholding or continuation of regular medications, and prescribing any new therapy based on the agreed pharmaceutical plan between admitting medical practitioner and pharmacist prescriber. The NIMC was started in ED and then continued throughout the patient stay in hospital. | 3745/3746 |

| | | |
|---|---|---|
| **Duration of treatment period** | The retrospective chart audits and comparison of the two groups were conducted as a snapshot, at the point of admission in ED, between the control and intervention groups. Even though the NIMC was continued for the rest of the inpatient stay, the audit was not continued beyond the admission period. | 3746 |
| **Timing** | The retrospective chart audits and comparison of the two groups were conducted as a snapshot, at the point of admission in ED, between the control and intervention groups. Even though the NIMC was continued for the rest of the inpatient stay, the audit was not continued beyond the admission period. | 3746 |
| **Co-interventions** | Not reported | NA |
| **Notes** | None | |

## Comparator group

| | **Description as stated in report/paper** | **Page number** |
|---|---|---|
| **Group name** | Medical practitioner prescribing | |
| **No. randomised/assigned to group** *(specify whether no. people or clusters)* | 48 individuals | 3748 (figure 2) |
| **Description** *(include sufficient detail for replication, e.g. content, dose, components)* | Participants in the control group received usual pharmacist and medical practitioner care. Control participants may (or may not) have been seen by a pharmacist in ED, to take a BPMH. This is usual care as some patients may only be seen by a pharmacist once patient was on the inpatient ward. The prescribing of the medications on the National Inpatient Medication Chart (NIMC) was the responsibility of the admitting medical practitioner whilst the patient was still in ED. The admitting medical practitioner was also responsible for assessing and prescribing VTE prophylaxis. | 3745 |
| **Duration of treatment period** | The retrospective chart audits and comparison of the two groups were conducted as a snapshot, at the point of admission in ED, between the control and intervention groups. Even though the NIMC was continued for the rest of the inpatient stay, the audit was not continued beyond the admission period. | 3746 |

| | | |
|---|---|---|
| **Timing** | The retrospective chart audits and comparison of the two groups were conducted as a snapshot, at the point of admission in ED, between the control and intervention groups. Even though the NIMC was continued for the rest of the inpatient stay, the audit was not continued beyond the admission period. | 3746 |
| **Co-interventions** | BPMH (Best Possible Medication History) | 3745 |
| **Notes** | Not reported | |

## Outcomes

### Prescribing errors

| | Description as stated in report/paper | | Page number |
|---|---|---|---|
| **Outcome name** | Prescribing errors | | 3747 |
| **Time points measured** *(specify whether from start or end of intervention)* | At time of admission | | 3746 |
| **Time points reported** | At time of admission | | 3746 |
| **Outcome definition** *(with diagnostic criteria if relevant)* | Any anomalies with regards to medication order legibility, incorrect documentation of route, dose and frequency, were counted as prescribing errors. | | 3747 |
| **Unit of measurement** *(if relevant)* | Event data | | 3747 |
| **Scales: upper and lower limits** *(indicate whether high or low score is good)* | Lower score is desired | | NA |
| **Is outcome/tool validated?** | No | NA | NA |
| **Imputation of missing data** *(e.g. assumptions made for ITT analysis)* | Not reported | | NA |
| **Assumed risk estimate** *(e.g. baseline or population risk noted in Background)* | The hypothesis was that the quality of the pharmacist collaborative prescribing model was the same as usual care (medical prescribing model). It was hypothesised that a 10% improvement in the quality of medication prescribing orders could be achieved in the intervention group. | | 3746 |
| **Power** *(e.g. power & sample size calculation, level of power achieved)* | The sample size required in each arm for a power of 80% and an alpha of 0.05 was calculated to be 250 medication orders. With a design effect of 1.4 the true required sample size was calculated to be 350 medication orders in each arm. | | 3746 |
| **Notes** | None | | |

## Funding/conflict of interest

| | | |
|---|---|---|
| **Study funding sources** *(including role of funders)* | This research study received no specific grant from any funding agency. | 3750 |
| **Possible conflicts of interest** *(for study authors)* | Not reported | NA |
| **Notes** | None | |

## Data and analysis

### Prescribing errors

| | Description as stated in report/paper | | | | Page number |
|---|---|---|---|---|---|
| **Outcome** | Prescribing errors | | | | 3747 |
| **Time point** *(specify from start or end of intervention)* | At time of admission | | | | 3746 |
| **Results** | Intervention | | Comparison | | 3748 |
| | No. event | Total in group | No. with event | Total in group | |
| | 68 | 38 | 279 | 35 | |
| **Any other results reported** | $p<0.0001$<br><br>Overall, there were 279/357 prescribing errors in the control group (78% error rate) with all but one patient (37/38) having a prescribing error. In the intervention group 68/412 (16% error rate) prescribing errors were recorded across 25/35 patients ($p < 0.001$, Fisher's exact). | | | | 3749, 3747 |
| **No. missing participants** | 11 | | 10 | | |
| **Reasons missing** | *Reasons for exclusion after recruitment include medications inadvertently prescribed by medical practitioner for an intervention patient, patients discharged from ED by medical practitioner, patients transferred to Hospital in the Home (HITH) or patients transferred to another hospital directly from ED. | | | | 3748 (figure 2) |
| **Statistical methods used and appropriateness of these** | As the numbers in this trial were anticipated to be relatively small, the Fisher's exact test, utilising the GraphPad Prism™ software, was used to analyse the data.19 All reported p values were two-sided with an alpha (significance value) of less than 0.05. | | | | 3747 |
| **Confounders** | Not reported | | | | NA |
| **Notes** | None | | | | |

## Conclusions

| | Description as stated in report/paper | Page number |
|---|---|---|
| **Key conclusions of study authors** | Safety and accuracy were the primary focus of this study. We hypothesised that a 10% improvement in prescribing errors could be achieved, and this was exceeded with an error rate in the pharmacist prescribing group being almost one-fifth of the medical practitioner prescribing group, a significant improvement. | 3749 |
| **Notes** | None | |

# O'Neill et al. 2014

## Study eligibility

| Study Characteristics | Eligibility criteria |
|---|---|
| **Title** | Collaborative Hypertension Case Management by Registered Nurses and Clinical Pharmacy Specialists within the Patient Aligned Care Teams (PACT) Model |
| **Author (year)** | O'Neill et al. 2014 |
| **Country** | Michigan, USA |
| **Type of study** | Retrospective cohort |
| **Participants** | All veteran adults with uncontrolled hypertension |
| **Types of intervention** | Pharmacist prescribing with nurse case management |
| **Types of comparison** | Physician prescribing with nurse case management |
| **Types of outcome measures** | Blood pressure goal attained (<140/90), systolic BP change, diastolic BP change, |
| **Prescriptive authority** | Collaborative practice agreement |
| **Include/Exclude** | Include |
| **Notes** | |

**DO NOT PROCEED IF STUDY EXCLUDED FROM REVIEW**

## Characteristics of included studies

### Methods

| | Descriptions as stated in report/paper | | Page number |
|---|---|---|---|
| **Aim of study** | The primary objective was to compare blood pressure between CPS-directed and physician-directed RNCM | | S676 |
| **Design** | Retrospective cohort. 'Non-randomized, retrospective comparison of a natural experiment' | | S676 |
| **Unit of allocation** *(by individuals, cluster/ groups or body parts)* | Individual | | S676 |
| **Start-end date** | 20 September 2011 – 1 November 2011 | | S677 |
| **Duration of participation** *(from recruitment to last follow-up/ baseline to last follow-up- group level)* *1. Time of consent until last measurement for each individual.* *2. Baseline to final follow-up for each individual* | Unclear. There is a list of patients from 1 May to 1 November 2011. Appointments were between 20 September and 31 October per the abstract.<br><br>Data was gathered for a maximum of 6 months (retrieved baseline data) | | S677, S675 |
| **Study duration** *(as above with the exception of interim analyses or other circumstances* | Up to 6 months | | S677 |
| **Ethical approval needed/obtained for study** | Yes | Institutional Review Board approval was achieved | S677 |
| **Notes** | | | |

### Participants

| | **Description** *Include comparative information for each intervention or comparison group if available* | **Page number** |
|---|---|---|

| | | | |
|---|---|---|---|
| **Population description** *(from which study participants are drawn)* | Veterans (with uncontrolled hypertension) | | S676 |
| **Setting** *(including location and social context)* | Patient Aligned Care Teams (PACT) within a Veterans Affairs (VA) clinic in the Midwest | | S675 |
| **Inclusion/exclusion criteria** | Inclusion: Veterans with uncontrolled hypertension<br>Exclusion: If both pharmacist and physician were involved in the index visit, enrolled in Home Based Primary Care, if they displayed non-adherence to the plan | | S675 |
| **Method of recruitment of participants** | The primary care provider refers patients to nurse case management for poorly controlled hypertension. The nurse follows-up with the patient in person or by telephone. If blood pressure is poorly controlled or a change of care plan is needed, the nurse will presents the case to either a physician or a pharmacist.<br>Data was retrospectively obtained from the VHA Information Systems and Technology Architecture report | | S676, Figure 1, S677 |
| **Informed consent obtained** | No | Institutional Review Board approval was achieved. No active consent noted | S677 |
| **Total no. randomised** *(or total pop. at start of study for NRCTs)* | 184 patients were evaluated.<br>126: 63 in each group were included<br>170 met inclusion criteria<br>14 excluded due to both pharmacist and physician involvement.<br>21 were excluded due to non-adherence to either laboratory monitoring orders or follow-up visits<br>23 were excluded due to non-adherence to recommended change in therapy. | | S678 |
| **Clusters** | None reported | | NA |
| **Baseline imbalances** | Demographic characteristics were similar between groups except race (p=0.01; Table 1). A greater proportion of patients in the physician-directed group had a race identified as “white”.<br><br>There was a significantly higher index systolic blood pressure in intervention compared with comparator groups. | | S678 (table 1) |
| **Withdrawals and exclusions** | 21 were excluded due to non-adherence to either laboratory monitoring orders or follow-up visits<br>23 were excluded due to non-adherence to recommended change in therapy. | | S678 |
| **Age** | Intervention: 63.4 ± 9.8<br>Comparator: 63.8 ± 10.0 | | S678 |
| **Sex (female)** | Intervention: n = 2 (3.2%)<br>Comparator: n = 1 (1.6%) | | S678 |

| | | |
|---|---|---|
| **Subgroups measure** | Not reported | NA |
| **Subgroups reported** | Not reported | NA |
| **Notes** | | |

## Intervention group

| | Description as stated in report/paper | Page number |
|---|---|---|
| **Group name** | Pharmacist prescribing with nurse case management | S677, S678 |
| **No. randomised/assigned to group** *(specify whether no. people or clusters)* | 63 | S677, S678 |
| **Description** *(include sufficient detail for replication, e.g. content, dose, components)* | Nurse assesses each patient. If hypertension remains poorly controlled or changes are indicated, the RN presents the case either to a physician or a CPS to collaboratively design a plan of care, which may include patient education on the self-management of their disease, changing medication therapy, consulting referral services, laboratory test ordering and/or arranging follow-up. The RN may chose to discuss the case with either a CPS (designated as "CPS-directed RNCM") or a physician (designated as "physician-directed RNCM"), based either on availability of the provider or complexity of the patient. The RN then returns to the patient and communicates any changes in the plan to the patient. | S677 |
| **Duration of treatment period** | No set treatment period. | S678 Table 1 |
| **Timing** | Unclear, 2 visits on average 3.4-3.9 weeks (+/- 1.9) | S678 Table 1 |
| **Co-interventions** | None reported | NA |
| **Notes** | | |

## Comparator group

| | Description as stated in report/paper | Page number |
|---|---|---|
| **Group name** | Physician prescribing with nurse case management | S677, S678 |
| **No. randomised/assigned to group** *(specify whether no. people or clusters)* | 63 | S677, S678 |

| | | |
|---|---|---|
| **Description** *(include sufficient detail for replication, e.g. content, dose, components)* | Nurse assesses each patient. If hypertension remains poorly controlled or changes are indicated, the RN presents the case either to a physician or a CPS to collaboratively design a plan of care, which may include patient education on the self-management of their disease, changing medication therapy, consulting referral services, laboratory test ordering and/or arranging follow-up. The RN may chose to discuss the case with either a CPS (designated as "CPS-directed RNCM") or a physician (designated as "physician-directed RNCM"), based either on availability of the provider or complexity of the patient. The RN then returns to the patient and communicates any changes in the plan to the patient. | S677 |
| **Duration of treatment period** | No set treatment period. Treatment continued until therapeutic range was reached for each individual. | S678 Table 1 |
| **Timing** | Unclear, 2 visits on average 3.4 weeks (+/- 1.6) | S678 Table 1 |
| **Co-interventions** | None reported | NA |
| **Notes** | | |

## Outcomes

### Blood pressure goal attained (<140/90)

| | Description as stated in report/paper | Page number |
|---|---|---|
| **Outcome name** | Blood pressure goal attained (<140/90) | S677 |
| **Time points measured** *(specify whether from start or end of intervention)* | To assess the main measure, blood pressure measurements at index and the next consecutive RNCM visit were collected for each patient. In the case of telephone encounters for patients who monitored their home blood pressure, the average blood pressure of multiple documented readings was used for the analysis. For face-to-face clinic appointments, home blood pressure cuff readings were included if it was documented that these were used instead of clinic blood pressure readings to determine progress to blood pressure goal, such as in the case of patients with "whitecoat hypertension." | S677 |
| **Time points reported** | Index, follow-up | S677, S678 |
| **Outcome definition** *(with diagnostic criteria if relevant)* | Not reported | NA |
| **Unit of measurement** *(if relevant)* | mmHg | S677 |
| **Scales: upper and lower limits** *(indicate whether high or low score is good)* | <140/90 is good<br>Above is not | S677 |

| | | | |
|---|---|---|---|
| **Is outcome/tool validated?** | No | | NA |
| **Imputation of missing data** *(e.g. assumptions made for ITT analysis)* | Not reported | | NA |
| **Assumed risk estimate** *(e.g. baseline or population risk noted in Background)* | Hypertension, a major cardiovascular disease risk factor, affected 30.4 % of the adult population of the United States between 2003 and 2010.1 | | S675 |
| **Power** *(e.g. power & sample size calculation, level of power achieved)* | Not reported | | NA |
| **Notes** | | | |

## Systolic blood pressure change

| | Description as stated in report/paper | | Page number |
|---|---|---|---|
| **Outcome name** | Systolic blood pressure change | | S677 |
| **Time points measured** *(specify whether from start or end of intervention)* | To assess the main measure, blood pressure measurements at index and the next consecutive RNCM visit were collected for each patient. In the case of telephone encounters for patients who monitored their home blood pressure, the average blood pressure of multiple documented readings was used for the analysis. For face-to-face clinic appointments, home blood pressure cuff readings were included if it was documented that these were used instead of clinic blood pressure readings to determine progress to blood pressure goal, such as in the case of patients with "whitecoat hypertension." | | S677 |
| **Time points reported** | Index, follow-up | | S677, S678 |
| **Outcome definition** *(with diagnostic criteria if relevant)* | Not reported | | NA |
| **Unit of measurement** *(if relevant)* | mmHg | | S677 |
| **Scales: upper and lower limits** *(indicate whether high or low score is good)* | <140/90 is good<br>Above is not | | S677 |
| **Is outcome/tool validated?** | No | | NA |

| | | |
|---|---|---|
| **Imputation of missing data** *(e.g. assumptions made for ITT analysis)* | Not reported | NA |
| **Assumed risk estimate** *(e.g. baseline or population risk noted in Background)* | Not reported | NA |
| **Power** *(e.g. power & sample size calculation, level of power achieved)* | Using a minimum average blood pressure change of 4 mmHg (SD=8), alpha level of 0.05, and 80 % power, the required sample size was calculated to be 63 patients per group. | S677 |
| **Notes** | | |

## Diastolic blood pressure change

| | Description as stated in report/paper | | Page number |
|---|---|---|---|
| **Outcome name** | Diastolic blood pressure change | | S677 |
| **Time points measured** *(specify whether from start or end of intervention)* | To assess the main measure, blood pressure measurements at index and the next consecutive RNCM visit were collected for each patient. In the case of telephone encounters for patients who monitored their home blood pressure, the average blood pressure of multiple documented readings was used for the analysis. For face-to-face clinic appointments, home blood pressure cuff readings were included if it was documented that these were used instead of clinic blood pressure readings to determine progress to blood pressure goal, such as in the case of patients with "whitecoat hypertension." | | S677 |
| **Time points reported** | Index, follow-up | | S677, S678 |
| **Outcome definition** *(with diagnostic criteria if relevant)* | Not reported | | NA |
| **Unit of measurement** *(if relevant)* | mmHg | | S677 |
| **Scales: upper and lower limits** *(indicate whether high or low score is good)* | <140/90 is good<br>Above is not | | S677 |
| **Is outcome/tool validated?** | No | | NA |
| **Imputation of missing data** *(e.g. assumptions made for ITT analysis)* | Not reported | | NA |

| | | |
|---|---|---|
| **Assumed risk estimate** *(e.g. baseline or population risk noted in Background)* | Not reported | NA |
| **Power** *(e.g. power & sample size calculation, level of power achieved)* | Not reported | NA |
| **Notes** | | |

## Funding/conflict of interest

| | | |
|---|---|---|
| **Study funding sources** *(including role of funders)* | 'None' financial disclosures reported | S680 |
| **Possible conflicts of interest** *(for study authors)* | The authors declare that they do not have a conflict of interest. | S680 |
| **Notes** | | |

## Data and analysis

### Blood pressure goal attained (<140/90)

| | Description as stated in report/paper | | | | Page number |
|---|---|---|---|---|---|
| **Outcome** | Blood pressure goal attained (<140/90) | | | | S678 |
| **Time point** *(specify from start or end of intervention)* | At end of intervention | | | | S678 |
| **Results** | Intervention | | Comparison | | S678 |
| | No. with event | Total in group | No. with event | Total in group | |
| | 41 | 63 | 44 | 63 | |

| | | | |
|---|---|---|---|
| **Any other results reported** | At follow-up, the numbers of patients with blood pressure <140/90 mmHg were similar between the two groups (n=41, 65 % in the CPS group and n=44, 70 % in the physician group, p=0.57).<br><br>OR (95% CI): 0.95 (0.40, 2.26) p=0.24 (adjusted for provider type, follow-up time, index visit blood pressure, and prior stroke)<br><br>Once provider type, follow-up time, index visit blood pressure, and prior stroke were controlled for, no significant effect for provider type on attaining blood pressure <140/90 mmHg (p=0.91)<br><br>Higher baseline systolic (p=.001) and diastolic (p=0.1) blood pressure was also associated with lower odds of attaining blood pressure 140<90 mmHg. In addition, patients who had a prior stroke had greater reductions in both systolic (p=.007) and diastolic blood pressure (p=.04), as well as greater odds of attaining blood pressure <140/90 mmHg (p=.05)<br><br>The final model for attainment of blood pressure <140/90 mmHg accounted for 18.8% of the variance (pseudo R squared = .188). | | S678, S679 Table 3, S679 |
| **No. missing participants** | Not reported | Not reported | NA |
| **Reasons missing** | Unknown. 21 missing due to nonadherence to labs/follow-up visits and 24 missing due to non-adherence to recommended change in therapy. | Unknown. 21 missing due to nonadherence to labs/follow-up visits and 24 missing due to non-adherence to recommended change in therapy. | S678 |

| | | |
|---|---|---|
| **Statistical methods used and appropriateness of these** | An independent samples t-test was also conducted to compare… attainment of blood pressure less than 140/90mmHg, for patients using CPS-directed RNCM versus physician-directed RNCM.<br><br>Separate preliminary regression models were conducted to examine the relationships between each of the patient measures with the outcomes (systolic blood pressure change, diastolic blood pressure change, attainment of blood pressure less than 140/90 mmHg), adjusting for baseline blood pressure measures. Measures that were either significant at p<0.20 or important for the current study (provider type, follow-up time, baseline blood pressure) were retained for inclusion in the multivariate model. All retained measures were simultaneously assessed using multiple regression models and were included in the final model if p<0.05…. Provider type was the independent variable of interest, and therefore, was included in the final model regardless of significance level. | S677, S678 |
| **Confounders** | Separate regression model → controlled for baseline value of blood pressure, age, number of blood pressure agents, diabetes and stroke. Only stroke was significant and remained in the final multivariable model. Final model included provider type, follow-up time, index visit blood pressure, and prior stroke. | S678 |
| **Notes** | None | |

## Systolic BP change

| | **Description as stated in report/paper** | | | | | | **Page number** |
|---|---|---|---|---|---|---|---|
| **Outcome** | Systolic BP change | | | | | | S678 |
| **Time point** *(specify from start or end of intervention)* | At end of intervention | | | | | | S678 |
| **Results** | Intervention | | | Comparison | | | S678 Table 2 |
| | Mean difference | SD | No. participants | Mean difference | SD | No. participants | |
| | 14 | 13 | 63 | 10 | 11 | 63 | |

| | | | |
|---|---|---|---|
| **Any other results reported** *(e.g. mean difference, CI, P value)* | Patients in the CPS-directed group had greater average decreases in systolic blood pressure compared to those in the physician-directed group (p=0.04)<br><br>After controlling for provider type, follow-up time, index visit blood pressure, and prior stroke, there was no significant effect for provider type on systolic blood pressure change (p=.42).<br><br>Patients who had a prior stroke had greater reductions in systolic blood pressure (p=.007)<br><br>The final model for blood pressure accounted for 14.6% of the total variance ins systolic blood pressure (adjusted r squared = .146) | | S678, S679 |
| **No. missing participants** | Unknown. 21 missing due to nonadherence to labs/follow-up visits and 24 missing due to non-adherence to recommended change in therapy. | Unknown. 21 missing due to nonadherence to labs/follow-up visits and 24 missing due to non-adherence to recommended change in therapy. | S678 |
| **Statistical methods used and appropriateness of these** *(e.g. adjustment for correlation)* | To assess the main measure, an independent samples t-test was conducted to compare difference in systolic blood pressure change between groups<br><br>Separate preliminary regression models were conducted to examine the relationships between each of the patient measures with the outcomes (systolic blood pressure change, diastolic blood pressure change, attainment of blood pressure less than 140/90 mmHg), adjusting for baseline blood pressure measures. Measures that were either significant at p<0.20 or important for the current study (provider type, follow-up time, baseline blood pressure) were retained for inclusion in the multivariate model. All retained measures were simultaneously assessed using multiple regression models and were included in the final model if p<0.05…. Provider type was the independent variable of interest, and therefore, was included in the final model regardless of significance level. | | S677, S678 |
| **Confounders** | Separate regression model → controlled for baseline value of blood pressure, age, number of blood pressure agents, diabetes and stroke. Only stroke was significant and remained in the final multivariable model. Final model included provider type, follow-up time, index visit blood pressure, and prior stroke. | | S678 |
| **Notes** | | | |

## Diastolic BP change

| | Description as stated in report/paper | Page number |
|---|---|---|

| | | | | | | | |
|---|---|---|---|---|---|---|---|
| **Outcome** | Diastolic BP change | | | | | | S678 |
| **Time point** *(specify from start or end of intervention)* | At end of intervention | | | | | | S678 |
| **Results** | Intervention | | | Comparison | | | S678 Table 2 |
| | Mean difference | SD | No. participants | Mean difference | SD | No. participants | |
| | 6 | 10 | 63 | 6 | 7 | 63 | |
| **Any other results reported** *(e.g. mean difference, CI, P value)* | The groups did not differ on measures of diastolic blood pressure changes (p=0.90).<br><br>After controlling for provider type, follow-up time, index visit blood pressure, and prior stroke, there was no significant change for diastolic blood pressure change (p=.93)<br><br>Patients who had a prior stroke had greater reductions in diastolic blood pressure (p=.04)<br><br>The final model accounted for 18.9% of the variance in diastolic blood pressure (adjusted r squared = .189) | | | | | | S678, S679 Table 2 |
| **No. missing participants** | Unknown. 21 missing due to nonadherence to labs/follow-up visits and 24 missing due to non-adherence to recommended change in therapy. | | | Unknown. 21 missing due to nonadherence to labs/follow-up visits and 24 missing due to non-adherence to recommended change in therapy. | | | S678 |
| **Statistical methods used and appropriateness of these** *(e.g. adjustment for correlation)* | An independent samples t-test was also conducted to compare diastolic blood pressure…<br><br>Separate preliminary regression models were conducted to examine the relationships between each of the patient measures with the outcomes (systolic blood pressure change, diastolic blood pressure change, attainment of blood pressure less than 140/90 mmHg), adjusting for baseline blood pressure measures. Measures that were either significant at p<0.20 or important for the current study (provider type, follow-up time, baseline blood pressure) were retained for inclusion in the multivariate model. All retained measures were simultaneously assessed using multiple regression models and were included in the final model if p<0.05…. Provider type was the independent variable of interest, and therefore, was included in the final model regardless of significance level. | | | | | | S677, S678 |

| | | |
|---|---|---|
| **Confounders** | Separate regression model → controlled for baseline value of blood pressure, age, number of blood pressure agents, diabetes and stroke. Only stroke was significant and remained in the final multivariable model. Final model included provider type, follow-up time, index visit blood pressure, and prior stroke. | S678 |
| **Notes** | | |

## Conclusions

| | **Description as stated in report/paper** | **Page number** |
|---|---|---|
| **Key conclusions of study authors** | Similar and clinically significant blood pressure reductions occurred in both the CPS-directed and physician-directed RNCM groups. Our results suggest that CPS-directed RNCM achieves blood pressure reduction comparable to physician-directed RNCM. | S680 |
| **Notes** | | |

# Rana et al. 2022

## Study eligibility

| **Study Characteristics** | **Eligibility criteria** |
|---|---|
| **Title** | A Retrospective Cohort Evaluation of a Pharmacist-Led Approach for Transitioning Patients to an Angiotensin Receptor—Neprilysin Inhibitor |
| **Author (year)** | Rana et al. 2022 |
| **Country** | USA |
| **Type of study** | Retrospective cohort |
| **Participants** | Adults with active prescriptions for an angiotensin receptor neprilysin inhibitor (ARNI) under cardiac care |
| **Types of intervention** | Pharmacist |
| **Types of comparison** | Usual care (physician) |
| **Types of outcome measures** | Percentage of patients achieving target angiotensin receptor neprilysin inhibitor (ARNI) dose; number of visits/days required to achieve target dose; heart-failure hospitalizations; all-cause death |
| **Prescriptive authority** | Collaborative practice agreement |
| **Include/Exclude** | Include |
| **Notes** | |

**DO NOT PROCEED IF STUDY EXCLUDED FROM REVIEW**

## Characteristics of included studies

### Methods

| | Descriptions as stated in report/paper | | Page number |
|---|---|---|---|
| **Aim of study** | To determine if a pharmacist-led outpatient ARNI replacement and titration program led to more patients achieving target doses of sacubitril–valsartan compared to usual care. | | 1062 |
| **Design** | Retrospective cohort | | 1062 |
| **Unit of allocation** *(by individuals, cluster/ groups or body parts)* | Individual | | 1062 |
| **Start-end date** | January 2015 – September 2018 | | 1062 |
| **Duration of participation** *(from recruitment to last follow-up/ baseline to last follow-up- group level)* *1. Time of consent until last measurement for each individual.* *2. Baseline to final follow-up for each individual* | Unclear. Until target dose achieved. | | NA |
| **Study duration** *(as above with the exception of interim analyses or other circumstances* | Unclear. Until target dose achieved. | | NA |
| **Ethical approval needed/obtained for study** | Yes | This study was approved by the Institutional Review Board. | 1062 |
| **Notes** | | | |

### Participants

| | **Description** *Include comparative information for each intervention or comparison group if available* | Page number |
|---|---|---|
| **Population description** *(from which study participants are drawn)* | Adults with active prescriptions for an angiotensin receptor neprilysin inhibitor (ARNI) under cardiac care | 1062 |

| | | |
|---|---|---|
| **Setting**<br>*(including location and social context)* | A large single health system in the United States; cardiology clinic | 1062 |
| **Inclusion/exclusion criteria** | Inclusion: at least 18 years old; diagnosis of heart failure with reduced ejection fraction (HFrEF); active sacubitril-valsartan prescription initiated between January 2015 and September 2018; at least 2 cardiology visits during the study period (Note: combination sacubitril/valsartan = ANRI)<br><br>Exclusion: no sacubitril-valsartan initiated during the study period; follow-up criteria not met; current receiving comfort care or enrolled in hospice; on dialysis; had an adverse drug event (ADE) requiring ARNI discontinuation | 1062 |
| **Method of recruitment of participants** | EHR identification for the study. Cardiac clinic providers referred patients by contacting staff to schedule a templated clinic visit with the pharmacist .<br><br>Patients were divided into 2 groups based on whether ARNI therapy was initiated by a pharmacist or by another provider across the health system, which was termed “usual care.” | 1062 |
| **Informed consent obtained** | No — This study was approved by the Institutional Review Board. EHR records. No consent. | NA |
| **Total no. randomised**<br>*(or total pop. at start of study for NRCTs)* | 791 individuals | 1063 |
| **Clusters** | None | NA |
| **Baseline imbalances** | Patients initiated on ARNI by a pharmacist were significantly younger, had higher K [potassium] levels, and lower pro-BNPs at baseline. In addition, the pharmacist group had more patients in NYHA Class II and on MRA at baseline, but a smaller percentage of patients with atrial fibrillation<br>or atrial flutter (Table 1). Median pre-ARNI enalapril equivalence was significantly higher in the usual care group (10 mg (IQR = 3.75) vs 8.96 mg (IQR = 12.4), P = .0085). However, BP, HR, SCr, and BMI at baseline were similar in both groups. | 1063 - 1064 |
| **Withdrawals and exclusions** | 100 patients were referred to the pharmacist, 96 of which were eligible, 83 of which were initiated on treatment. 17 discontinued and a further 2 did not have cardiology visits. Leaving 64 ‘enrolled and analyzed.’ | 1063, Figure 2 |
| **Age** | Intervention: 57.05 ± 12.62<br>Comparator: 64.52 ± 12.63 | 1065 |
| **Sex (female)** | Intervention: 21 (32.8%)<br>Comparator: 249 (34.4%) | 1065 |

| | | |
|---|---|---|
| **Subgroups measure** | Unknown New York Heart Association (NYHA) Class II–III symptoms | NA |
| **Subgroups reported** | Target ARNI dose: Main result persisted when excluding patient with unknown NYHA class | NA |
| **Notes** | | |

## Intervention group

| | Description as stated in report/paper | Page number |
|---|---|---|
| **Group name** | Pharmacist managed | 1062 |
| **No. randomised/assigned to group** *(specify whether no. people or clusters)* | 64 enrolled and analyzed.<br>100 patients were referred to the pharmacist, 96 of which were eligible, 83 of which were initiated on treatment. 17 discontinued and a further 2 did not have cardiology visits. | 1063, Figure 2 |
| **Description** *(include sufficient detail for replication, e.g. content, dose, components)* | The pharmacists are credentialed and privileged as advanced practice providers (APPs) and see patients individually or in multidisciplinary visits under physician supervision via a collaborative practice agreement for a variety of programs in the cardiology clinic.<br><br>Providers within the local cardiology clinic referred patients specifically for ARNI initiation by contacting staff to schedule a templated clinic visit with the pharmacist. Blood pressure (BP), potassium (K), and serum creatinine (SCr) were measured at baseline and 2 to 4 weeks after initiation or dose increases. Uptitration by telephone was not captured in the data. The pharmacist made independent decisions to decrease dose or discontinue drugs for abnormal findings with cardiology consultation as needed. The full scope of medication changes as well as labs and tests ordered were outlined in the scope of practice and collaborative practice agreement.<br><br>In the pharmacist group clinic model, the clinical pharmacist plays a role in choosing GDMT; completing Insurance prior authorization/appeals; assisting with medication financial assistance and access; and ultimately, independently initiating, titrating, and monitoring medications. These tasks involve extensive clinical review, patient counselling, and frequent follow-ups via telephone, electronic medical record, and face to-face encounters. | 1062, 1064 |

| | | |
|---|---|---|
| **Duration of treatment period** | No set treatment period. Treatment continued until therapeutic range was reached for each individual. | 1062 |
| **Timing** | Not reported | NA |
| **Co-interventions** | Not reported | NA |
| **Notes** | | |

## Comparator group

| | Description as stated in report/paper | Page number |
|---|---|---|
| **Group name** | Physician managed | 1062 |
| **No. randomised/assigned to group** *(specify whether no. people or clusters)* | 727 | 1063 Figure 1<br>1065 Table 5 |
| **Description** *(include sufficient detail for replication, e.g. content, dose, components)* | Patients were divided into 2 groups based on whether ARNI therapy was initiated by a pharmacist or by another provider across the health system, which was termed "usual care." | 1062 |
| **Duration of treatment period** | No set treatment period. Treatment continued until therapeutic range was reached for each individual. | 1062 |
| **Timing** | Not reported | NA |
| **Co-interventions** | Not reported | NA |
| **Notes** | | |

## Outcomes

### Percentage of patients who achieved a target ARNI dose

| | Description as stated in report/paper | Page number |
|---|---|---|
| **Outcome name** | Percentage of patients who achieved a target ARNI dose | 1062 |
| **Time points measured** *(specify whether from start or end of intervention)* | At end of intervention | NA |
| **Time points reported** | At end of intervention | NA |

| | | | |
|---|---|---|---|
| **Outcome definition** *(with diagnostic criteria if relevant)* | In order to estimate each patient's total daily dose of ARNI, daily doses of sacubitril and valsartan were added together to obtain a single numeric value. The total daily dose of each strength of sacubitril–valsartan combination tablets were calculated as follows: sacubitril–valsartan 24/26-mg tablet twice daily = 100 mg daily, sacubitril–valsartan 49/51-mg tablet twice daily = 200 mg daily, and sacubitril–valsartan 97/103-mg tablet twice daily = 400 mg daily. The median dose of ACEI and ARB in each group was converted to an enalapril equivalent | | 1064, 1062 |
| **Unit of measurement** *(if relevant)* | 97/103 mg twice daily | | 1062 |
| **Scales: upper and lower limits** *(indicate whether high or low score is good)* | The total daily dose of each strength of sacubitril–valsartan combination tablets were calculated as follows: sacubitril–valsartan 24/26-mg tablet twice daily = 100 mg daily, sacubitril–valsartan 49/51-mg tablet twice daily = 200 mg daily, and sacubitril–valsartan 97/103-mg tablet twice daily = 400 mg daily. | | 1062 |
| **Is outcome/tool validated?** | No | | NA |
| **Imputation of missing data** *(e.g. assumptions made for ITT analysis)* | Not reported | | NA |
| **Assumed risk estimate** *(e.g. baseline or population risk noted in Background)* | Not reported | | NA |
| **Power** *(e.g. power & sample size calculation, level of power achieved)* | Not reported | | NA |
| **Notes** | | | |

## Number of visits required to achieve target dose

| | **Description as stated in report/paper** | **Page number** |
|---|---|---|
| **Outcome name** | Number of visits required to reach target dose | 1062 |
| **Time points measured** *(specify whether from start or end of intervention)* | At end of intervention | NA |
| **Time points reported** | At end of intervention | NA |

| | | |
|---|---|---|
| **Outcome definition** *(with diagnostic criteria if relevant)* | Number of visits required to reach target dose (Target dose information listed above) | 1062, 1064 |
| **Unit of measurement** *(if relevant)* | Number of visits | 1064 |
| **Scales: upper and lower limits** *(indicate whether high or low score is good)* | 2 upwards | 1064 |
| **Is outcome/tool validated?** | No | NA |
| **Imputation of missing data** *(e.g. assumptions made for ITT analysis)* | Not reported | NA |
| **Assumed risk estimate** *(e.g. baseline or population risk noted in Background)* | Not reported | NA |
| **Power** *(e.g. power & sample size calculation, level of power achieved)* | Not reported | NA |
| **Notes** | | |

### Number of days required to achieve target/maximally tolerated dose

| | **Description as stated in report/paper** | **Page number** |
|---|---|---|
| **Outcome name** | Number of days required to reach target/maximally tolerated dose | 1062 |
| **Time points measured** *(specify whether from start or end of intervention)* | At end of intervention | NA |
| **Time points reported** | At end of intervention | NA |
| **Outcome definition** *(with diagnostic criteria if relevant)* | Number of days required to reach target/maximally tolerated dose | 1062, 1064 |
| **Unit of measurement** *(if relevant)* | Number of visits | 1064 |
| **Scales: upper and lower limits** *(indicate whether high or low score is good)* | Lower scores are desired | NA |
| **Is outcome/tool validated?** | No | NA |

| | | |
|---|---|---|
| **Imputation of missing data** *(e.g. assumptions made for ITT analysis)* | Not reported | NA |
| **Assumed risk estimate** *(e.g. baseline or population risk noted in Background)* | Not reported | NA |
| **Power** *(e.g. power & sample size calculation, level of power achieved)* | Not reported | NA |
| **Notes** | | |

## Heart failure hospitalizations

| | Description as stated in report/paper | | Page number |
|---|---|---|---|
| **Outcome name** | Heart failure hospitalizations | | 1062 |
| **Time points measured** *(specify whether from start or end of intervention)* | Within the study period | | 1062 |
| **Time points reported** | Within the study period | | 1062 |
| **Outcome definition** *(with diagnostic criteria if relevant)* | HF hospitalizations within the study period | | 1062 |
| **Unit of measurement** *(if relevant)* | Continuous count | | 1064 |
| **Scales: upper and lower limits** *(indicate whether high or low score is good)* | 0 upwards | | 1064 |
| **Is outcome/tool validated?** | No | | NA |
| **Imputation of missing data** *(e.g. assumptions made for ITT analysis)* | Not reported | | NA |
| **Assumed risk estimate** *(e.g. baseline or population risk noted in Background)* | Not reported | | NA |
| **Power** *(e.g. power & sample size calculation, level of power achieved)* | Not reported | | NA |

| | |
|---|---|
| **Notes** | |

## All-cause death

| | Description as stated in report/paper | | Page number |
|---|---|---|---|
| **Outcome name** | All-cause death | | 1062 |
| **Time points measured** *(specify whether from start or end of intervention)* | Unclear, within the study period | | 1064 |
| **Time points reported** | Unclear, within the study period | | 1064 |
| **Outcome definition** *(with diagnostic criteria if relevant)* | Death | | NA |
| **Unit of measurement** *(if relevant)* | Binary, Dead/alive | | 1062, 1064 |
| **Scales: upper and lower limits** *(indicate whether high or low score is good)* | Alive good. Dead bad. | | 1062, 1064 |
| **Is outcome/tool validated?** | No | | NA |
| **Imputation of missing data** *(e.g. assumptions made for ITT analysis)* | Not reported | | NA |
| **Assumed risk estimate** *(e.g. baseline or population risk noted in Background)* | Not reported | | NA |
| **Power** *(e.g. power & sample size calculation, level of power achieved)* | Not reported | | NA |
| **Notes** | | | |

## Funding/conflict of interest

| | | |
|---|---|---|
| **Study funding sources** *(including role of funders)* | The author(s) received no financial support for the research, authorship, and/or publication of this article. | 1067 |
| **Possible conflicts of interest** *(for study authors)* | The author(s) declared no potential conflicts of interest with respect to the research, authorship, and/or publication of this article. | 1067 |
| **Notes** | | |

## Data and analysis

### Percentage of patients who achieved a target ARNI dose

| | Description as stated in report/paper | | | | Page number |
|---|---|---|---|---|---|
| **Outcome** | Percentage of patients who achieved a target ARNI dose | | | | 1062 |
| **Time point** *(specify from start or end of intervention)* | At end of intervention | | | | 1062, 1064 |
| **Results** | Intervention | | Comparison | | 1064, 1066 Table 2 |
| | No. with event | Total in group | No. with event | Total in group | |
| | 39 | 64 | 131 | 727 | |
| **Any other results reported** | More patients in the pharmacist group were successfully titrated to target ARNI dose of 97/103 mg twice daily (60.9% (39/64) vs 18.0% (131/727); OR, 2.38; 95% CI for group difference 0.31 to 0.55; P <0 .0001).<br><br>This result persisted when excluding patients with unknown NYHA Class (60.9% (39/64) vs 14.1% (98/694); OR, 3.32; 95% CI for group difference .34 to .59; P < 0.0001). | | | | 1064 |
| **No. missing participants** | Not reported | | Not reported | | NA |
| **Reasons missing** | Not reported | | Not reported | | NA |
| **Statistical methods used and appropriateness of these** | A chi-square test was used to compare percentages of patients at target dose and difference in percentages of patients who achieved each total daily ARNI dose, viewed as categorical variables, between the 2 groups. A 95% confidence interval (CI) and a two-tailed P-value < 0.05 were considered statistically significant | | | | 1062 - 1063 |
| **Confounders** | Not reported | | | | NA |
| **Notes** | None | | | | |

### Number of visits required to achieve target dose

| | Description as stated in report/paper | | | | | | Page number |
|---|---|---|---|---|---|---|---|
| **Outcome** | Number of visits required to reach target dose | | | | | | 1062 |
| **Time point** *(specify from start or end of intervention)* | Through the study period, cumulative at end of intervention | | | | | | 1062 |
| **Results** | Intervention | | | Comparison | | | 1064 |
| | Mean | SD *(or other variance, specify)* | No. participants | Mean | SD *(or other variance, specify)* | No. participants | |

| | | | | | | | |
|---|---|---|---|---|---|---|---|
| | 4.16 | | 64 | 12.94 | | 727 | |
| **Any other results reported** *(e.g. mean difference, CI, P value)* | The pharmacist group…required less visits to reach target dose compared to usual care (4.16 visits vs 12.94 visits; 95% CI -10.30 to 7.26, P < 0.0001). However, there was not a significant difference in the days from initiation to target or maximally tolerated dose (279.32 vs 333.66 days; 95% CI -117.59 to 8.92, P = 0.091) between the pharmacist group and usual care recorded at an in-person visit. | | | | | | 1064 |
| **No. missing participants** | Not reported | | | Not reported | | | NA |
| **Statistical methods used and appropriateness of these** *(e.g. adjustment for correlation)* | A 2-sample t-test was utilized to compare baseline characteristics and other normally distributed data and is presented as means ± standard deviation (SD). For asymmetric distributions, a Wilcoxon rank sum test was used and data are presented as medians and interquartile range (IQR). | | | | | | NA |
| **Confounders** | Not reported | | | | | | NA |
| **Notes** | | | | | | | |

## Number of days required to achieve target/maximally tolerated does

| | **Description as stated in report/paper** | | | | | | **Page number** |
|---|---|---|---|---|---|---|---|
| **Outcome** | Number of visits required to reach target dose | | | | | | 1062 |
| **Time point** *(specify from start or end of intervention)* | Number of days required to achieve target/maximally tolerated does | | | | | | 1062 |
| **Results** | Intervention | | | Comparison | | | 1064 |
| | Mean | SD *(or other variance, specify)* | No. participants | Mean | SD *(or other variance, specify)* | No. participants | |
| | 279.32 | | 64 | 333.66 | | 727 | |
| **Any other results reported** *(e.g. mean difference, CI, P value)* | There was not a significant difference in the days from initiation to target or maximally tolerated dose (279.32 vs 333.66 days; 95% CI -117.59 to 8.92, P = 0.091) between the pharmacist group and usual care recorded at an in-person visit. | | | | | | 1064 |
| **No. missing participants** | Not reported | | | Not reported | | | NA |

| | | |
|---|---|---|
| **Statistical methods used and appropriateness of these** *(e.g. adjustment for correlation)* | A 2-sample t-test was utilized to compare baseline characteristics and other normally distributed data and is presented as means ± standard deviation (SD). For asymmetric distributions, a Wilcoxon rank sum test was used and data are presented as medians and interquartile range (IQR). | NA |
| **Confounders** | Not reported | NA |
| **Notes** | | |

## Hospitalizations

| | Description as stated in report/paper | | | | Page number |
|---|---|---|---|---|---|
| **Outcome** | Hospitalizations | | | | 1062 |
| **Time point** *(specify from start or end of intervention)* | Within the study period | | | | 1062 |
| **Results** | Intervention | | Comparison | | 1064 |
| | No. with event | Total in group | No. with event | Total in group | |
| | 33 | 64 | 398 | 727 | |
| **Any other results reported** | HF hospitalizations occurred in 51.6% (33/64) of patients in the pharmacist group and 51.6% (398/727) patients in the usual care group (95% CI, 10.9 to 76.9; P = 0.733). | | | | 1064 |
| **No. missing participants** | Not reported | | Not reported | | NA |
| **Reasons missing** | Not reported | | Not reported | | NA |
| **Statistical methods used and appropriateness of these** | A 2-sample t-test was utilized to compare baseline characteristics and other normally distributed data and is presented as means ± standard deviation (SD). For asymmetric distributions, a Wilcoxon rank sum test was used and data are presented as medians and interquartile range (IQR). | | | | NA |
| **Confounders** | Not reported | | | | NA |
| **Notes** | None | | | | |

## All-cause death

| | Description as stated in report/paper | | Page number |
|---|---|---|---|
| **Outcome** | All-cause death | | 1062 |
| **Time point** *(specify from start or end of intervention)* | Within the study period | | 1062 |
| **Results** | Intervention | Comparison | 1064 |

| | | | | | |
|---|---|---|---|---|---|
| | No. with event | Total in group | No. with event | Total in group | |
| | 0 | 64 | 47 | 727 | |
| **Any other results reported** | There were 47 all-cause deaths in the usual care group, and no deaths in the pharmacist group (95% CI, -8.3 to 4.7; P = 0.036). | | | | 1064 |
| **No. missing participants** | Not reported | | Not reported | | NA |
| **Reasons missing** | Not reported | | Not reported | | NA |
| **Statistical methods used and appropriateness of these** | A 2-sample t-test was utilized to compare baseline characteristics and other normally distributed data and is presented as means ± standard deviation (SD). For asymmetric distributions, a Wilcoxon rank sum test was used and data are presented as medians and interquartile range (IQR). | | | | NA |
| **Confounders** | Not reported | | | | NA |
| **Notes** | None | | | | |

## Conclusions

| | **Description as stated in report/paper** | **Page number** |
|---|---|---|
| **Key conclusions of study authors** | In conclusion, patients seen by a pharmacist were more likely to achieve target doses of ARNI and a higher total daily dose in less in-person visits compared to usual care. | 1067 |
| **Notes** | | |

# Rashid et al. 2020

## Study eligibility

| **Study Characteristics** | **Eligibility criteria** |
|---|---|
| **Title** | Evaluation of a Pharmacist-Managed Nonsteroidal Anti-Inflammatory Drugs Deprescribing Program in an Integrated Health Care System |
| **Author (year)** | Rasid (2020) |
| **Country** | USA (California) |
| **Type of study** | Retrospective, propensity-score-matched cohort study |
| **Participants** | Adults at least 65 years of age receiving NSAIDs who were at risk of adverse events |
| **Types of intervention** | Pharmacist deprescribing |
| **Types of comparison** | Clinician deprescribing |

| | |
|---|---|
| **Types of outcome measures** | GI bleeds, acute kidney injuries, and exacerbation of pain triggering a hospitalization or emergency room visit, change in monthly NSAID cost |
| **Prescriptive authority** | Collaborative practice agreement |
| **Include/Exclude** | Include |
| **Notes** | |

**DO NOT PROCEED IF STUDY EXCLUDED FROM REVIEW**

## Characteristics of included studies

### Methods

| | Descriptions as stated in report/paper | | Page number |
|---|---|---|---|
| **Aim of study** | To evaluate effectiveness, safety, and economic outcomes of a pharmacist-managed deprescribing of NSAIDs within an integratedhealth care system. | | 919 |
| **Design** | Retrospective, propensity-score-matched cohort study | | 918, 919 |
| **Unit of allocation** *(by individuals, cluster/ groups or body parts)* | Individual | | |
| **Start-end date** | July 1, 2016 – June 30, 2018 | | 919 |
| **Duration of participation** *(from recruitment to last follow-up/ baseline to last follow-up- group level)* *1. Time of consent until last measurement for each individual.* *2. Baseline to final follow-up for each individual* | 6 months or until end of membership or death, whichever occurred first | | 920 |
| **Study duration** *(as above with the exception of interim analyses or other circumstances* | 6 months | | 920 |
| **Ethical approval needed/obtained for study** | Yes | approved by the Institutional Review Board at KPNC. Informed consent was waived because of the retrospective nature of the study. | 919 |
| **Notes** | | | |

### Participants

| | **Description** *Include comparative information for each intervention or comparison group if available* | Page number |
|---|---|---|
| **Population description** *(from which study participants are drawn)* | Adults at least 65 years of age receiving NSAIDs who were at risk of adverse events | 919 |

| | | |
|---|---|---|
| **Setting** *(including location and social context)* | Kaiser Permanente Northern California (KPNC) facilities (ambulatory care) | 919 |
| **Inclusion/exclusion criteria** | Inclusion: at least 65 years of age; receiving at least 270 days supply of NSAIDs within a year before the programme eligibility date; at least 1 risk factor which could be exacerbated by NSAID use with the past 24 hours (diagnosis of GI bleed or disorders, cardiovascular disease, end stage renal disease; or usage of an anticoagulant or prednisone >10 mg per day or an equivalent systemic corticosteroid measured over the previous 12 months)<br>Exclusion: no previous KPNC membership 6 months before the index date; index date outside of inclusion period; no evidence of NSAID use within 90 days of index date. | 920 Figure 1 |
| **Method of recruitment of participants** | EMR identification, PCP approval, Pharmacist contact.<br>EMRs are screened quarterly to identify eligible patients based on an internally created electronic algorithm. Once eligible patients are identified, clinical pharmacists obtain protocol authorization via the EMR from the patient's primary care provider to review for NSAID deprescribing interventions. After receiving approval, clinical pharmacists review each patient's medical chart to determine if the patient is a good candidate for deprescribing interventions. If so, the pharmacist contacts the patient via telephone to discuss an appropriate deprescribing intervention based on the patient's drug regimen, preference of interventions,<br>and lifestyle. | 919 |
| **Informed consent obtained** | No — Informed consent was waived because of the retrospective nature of the study. | 919 |
| **Total no. randomised** *(or total pop. at start of study for NRCTs)* | 2155 (after propensity score matching) | 920 |
| **Clusters** | None reported | NA |
| **Baseline imbalances** | None, propensity-score matching addressed these<br>There were no statistical differences between the 2 matched groups in terms of age, sex, race, CCI score, previous hospitalized GI bleed events, NSAID classes, and pain related diagnoses associated with the use of NSAIDs (Table 1). | 921 |
| **Withdrawals and exclusions** | 605 from usual care not needed after propensity score matching | 920 Figure 1 |
| **Age** | Intervention: 76.1 ± 6.7<br>Comparator: 75.8 ± 7.1 | 921 |
| **Sex (female)** | Intervention: 53.8%<br>Comparator: 55.1% | 921 |

| | | |
|---|---|---|
| **Subgroups measure** | None reported | NA |
| **Subgroups reported** | None reported | NA |
| **Notes** | | |

## Intervention group

| | **Description as stated in report/paper** | **Page number** |
|---|---|---|
| **Group name** | Pharmacist-managed, deprescribed group | 919, 921 |
| **No. randomised/assigned to group** *(specify whether no. people or clusters)* | 431 | 921, Figure 1 |
| **Description** *(include sufficient detail for replication, e.g. content, dose, components)* | EMRs are screened quarterly to identify eligible patients based on an internally created electronic algorithm. Once eligible patients are identified, clinical pharmacists obtain protocol authorization via the EMR from the patient's primary care provider to review for NSAID deprescribing interventions. After receiving approval, clinical pharmacists review each patient's medical chart to determine if the patient is a good candidate for deprescribing interventions. If so, the pharmacist contacts the patient via telephone to discuss an appropriate deprescribing intervention based on the patient's drug regimen, preference of interventions,<br>and lifestyle. | 919 |
| **Duration of treatment period** | 6 months or until end of membership or death, whichever occurred first | 920 |
| **Timing** | Initial contact, follow-up (2-4 weeks), then as needed<br>Finally, a follow-up is typically scheduled within 2 to 4 weeks, but the clinical pharmacists make themselves accessible via secure online messaging through Kaiser Permanente's member website (kp.org) or via telephone between visits. Patients would be monitored for pain control at scheduled follow-ups and would be assessed for the effectiveness of the deprescribing plan. | 919 |
| **Co-interventions** | Chart review, monitoring, titration, counselling | 919 |
| **Notes** | | |

## Comparator group

| | **Description as stated in report/paper** | **Page number** |
|---|---|---|
| **Group name** | Usual care (physician deprescribing) | 919 |

| | | |
|---|---|---|
| **No. randomised/assigned to group** *(specify whether no. people or clusters)* | 1724 | 921, Figure 1 |
| **Description** *(include sufficient detail for replication, e.g. content, dose, components)* | The usual care group included patients who were eligible for the program during the cohort identification period but did not have their medical records reviewed nor had any interventions done by clinical pharmacists because of resource limitations | 919 |
| **Duration of treatment period** | No set treatment period. 6 months or until end of membership or death, whichever occurred first | 920 |
| **Timing** | Not reported | NA |
| **Co-interventions** | Not reported | NA |
| **Notes** | | |

## Outcomes

### Gastrointestinal (GI) bleeding events (hospitalisations and ER visits)

| | **Description as stated in report/paper** | | **Page number** |
|---|---|---|---|
| **Outcome name** | Gastrointestinal (GI) bleeding events (hospitalisations and ER visits) | | 920 |
| **Time points measured** *(specify whether from start or end of intervention)* | At end of intervention (6 months) | | 920 |
| **Time points reported** | At end of intervention (6 months) | | 920 |
| **Outcome definition** *(with diagnostic criteria if relevant)* | Any emergency room (ER) visit or hospitalization with a primary diagnosis of GI bleed using ICD-9/10-CM or DRG codes (Appendix). | | 920 |
| **Unit of measurement** *(if relevant)* | Events (composite ER visits and hospitalisations) | | 922 |
| **Scales: upper and lower limits** *(indicate whether high or low score is good)* | 0 upwards; lower scores are desired | | 922 |
| **Is outcome/tool validated?** | No | | NA |
| **Imputation of missing data** *(e.g. assumptions made for ITT analysis)* | Not reported | | NA |

| | | |
|---|---|---|
| **Assumed risk estimate** *(e.g. baseline or population risk noted in Background)* | Not reported | NA |
| **Power** *(e.g. power & sample size calculation, level of power achieved)* | Approximately 272 patients in the pharmacist-managed group and 1,088 patients in the usual care group were required to achieve 80% power with a type I error of 5% in order to detect a decrease from 11% to 5% in the proportion of patients who experience GI bleeds. | 921 |
| **Notes** | | |

## Acute kidney injury (AKI) events (hospitalisations and ER visits)

| | Description as stated in report/paper | | Page number |
|---|---|---|---|
| **Outcome name** | Acute kidney injury (AKI) events (hospitalisations and ER visits) | | 920 |
| **Time points measured** *(specify whether from start or end of intervention)* | At end of intervention (6 months) | | 920 |
| **Time points reported** | At end of intervention (6 months) | | 920 |
| **Outcome definition** *(with diagnostic criteria if relevant)* | any emergency room (ER) visit or hospitalization with a primary diagnosis of AKI using ICD-9/10-CM or DRG codes (Appendix). | | 920 |
| **Unit of measurement** *(if relevant)* | Events (composite ER visits and hospitalizations) | | 922 |
| **Scales: upper and lower limits** *(indicate whether high or low score is good)* | 0 upwards | | 922 |
| **Is outcome/tool validated?** | No | | NA |
| **Imputation of missing data** *(e.g. assumptions made for ITT analysis)* | Not reported | | NA |
| **Assumed risk estimate** *(e.g. baseline or population risk noted in Background)* | Not reported | | NA |
| **Power** *(e.g. power & sample size calculation, level of power achieved)* | Not reported | | NA |
| **Notes** | | | |

## Pain (hospitalisations and ER visits)

| | Description as stated in report/paper | | Page number |
|---|---|---|---|
| **Outcome name** | Pain (hospitalisations and ER visits) | | 921 |
| **Time points measured** *(specify whether from start or end of intervention)* | At end of intervention (6 months) | | 920 |
| **Time points reported** | At end of intervention (6 months) | | 920 |
| **Outcome definition** *(with diagnostic criteria if relevant)* | Exacerbation of pain or preexisting pain was used as a proxy for safety outcome.<br><br>The proportion of patients diagnosed within 6 months before the index date with at least 1 of the 8 pain-related diagnoses and then presented to the ER or hospitalized primarily because of an exacerbation of the same pain. The 8 pain diagnoses were defined using ICD-9/10-CM codes for general pain, headache, ankylosing spondylitis, gout, osteoarthritis, rheumatoid arthritis,joint pain, or muscle spasm (Appendix). | | 920 |
| **Unit of measurement** *(if relevant)* | Number of exacerbation/number of patients with indications during baseline | | 922 |
| **Scales: upper and lower limits** *(indicate whether high or low score is good)* | Fraction. Smaller numerator is desired. | | 922 |
| **Is outcome/tool validated?** | No | | NA |
| **Imputation of missing data** *(e.g. assumptions made for ITT analysis)* | Not reported | | NA |
| **Assumed risk estimate** *(e.g. baseline or population risk noted in Background)* | Not reported | | NA |
| **Power** *(e.g. power & sample size calculation, level of power achieved)* | Not reported | | NA |
| **Notes** | | | |

## Funding/conflict of interest

| | | |
|---|---|---|
| **Study funding sources** *(including role of funders)* | No funding was provided to support this research study. | 924 |

| | | |
|---|---|---|
| **Possible conflicts of interest** *(for study authors)* | The authors of this study have no actual or potential conflicts of interest to report | 924 |
| **Notes** | | |

## Data and analysis

### Gastrointestinal (GI) bleeding events (hospitalisations and ER visits)

| | Description as stated in report/paper | | | | Page number |
|---|---|---|---|---|---|
| **Outcome** | Gastrointestinal (GI) bleeding events (hospitalisations and ER visits) | | | | 920 |
| **Time point** *(specify from start or end of intervention)* | At end of intervention (6 months) | | | | 920 |
| **Results** | Intervention | | Comparison | | 922 table 2 |
| | No. with event | Total in group | No. with event | Total in group | |
| | 17 | 431 | 113 | 1724 | |
| **Any other results reported** | The unadjusted rate of patients who experienced GI bleed events was significantly lower in the deprescribed group than in the usual care group (3.9% vs. 6.6%, respectively, P = 0.04) and was primarily driven by hospitalization (2.6% vs. 5.1%, respectively, P = 0.03).<br><br>The adjusted odds ratio (OR) for the deprescribed group experiencing GI bleed events was 0.65 (95% confidence interval [CI] = 0.36-1.16, P = 0.15) in comparison with the usual care group (Table 3). | | | | 922 |
| **No. missing participants** | Not reported | | Not reported | | NA |
| **Reasons missing** | Not reported | | Not reported | | NA |
| **Statistical methods used and appropriateness of these** | Conditional logistic regressions were conducted to determine the risk of GI bleed events, AKI, and exacerbation of pain. | | | | 921 |
| **Confounders** | For effectiveness and safety outcomes, the common independent variables used in the conditional logistic regression model included age, sex, CCI score, Caucasian, NSAID classes, and 8 pain diagnoses. For risk of AKI and exacerbation of pain, we also adjusted for renal disease. | | | | 921 |
| **Notes** | None | | | | |

### Acute kidney injury (AKI) events (hospitalisations and ER visits)

| | Description as stated in report/paper | Page number |
|---|---|---|
| **Outcome** | Acute kidney injury (AKI) events (hospitalisations and ER visits) | 920 |

| | | | | | |
|---|---|---|---|---|---|
| **Time point** *(specify from start or end of intervention)* | At end of intervention (6 months) | | | | 920 |
| **Results** | Intervention | | Comparison | | 922 table 2 |
| | No. with event | Total in group | No. with event | Total in group | |
| | 10 | 431 | 68 | 1724 | |
| **Any other results reported** | For the unadjusted rate of AKI, there was no significant difference between the deprescribed group and the usual care group (2.3% vs. 3.9%, respectively, P = 0.11; Table 2).<br><br>The adjusted OR for the deprescribed group experiencing AKI was 0.53 (0.24-1.16, *P* = 0.11) in comparison with the usual care group (Table 3). | | | | 922 |
| **No. missing participants** | Not reported | | Not reported | | NA |
| **Reasons missing** | Not reported | | Not reported | | NA |
| **Statistical methods used and appropriateness of these** | Conditional logistic regressions were conducted to determine the risk of GI bleed events, AKI, and exacerbation of pain | | | | 921 |
| **Confounders** | For effectiveness and safety outcomes, the common independent variables used in the conditional logistic regression model included age, sex, CCI score, Caucasian, NSAID classes, and 8 pain diagnoses. For risk of AKI and exacerbation of pain, we also adjusted for renal disease. | | | | 921 |
| **Notes** | None | | | | |

## Pain (hospitalisations and ER visits)

| | **Description as stated in report/paper** | | | | **Page number** |
|---|---|---|---|---|---|
| **Outcome** | Pain (hospitalisations and ER visits) | | | | 921 |
| **Time point** *(specify from start or end of intervention)* | At end of intervention (6 months) | | | | 920 |
| **Results** | Intervention | | Comparison | | 922 table 2 |
| | No. with event | Total in group | No. with event | Total in group | |
| | 32 | 342 | 220 | 1463 | |

| | | | |
|---|---|---|---|
| **Any other results reported** | Of patients with composite pain, defined as having an ED visit or hospitalization with a primary diagnosis of at least 1 of the 8 pain diagnoses, was lower in the deprescribed group versus the usual care group (9.4% vs. 15.0%, P < 0.01).<br><br>Among the individual pain diagnosis, the proportion of patients with exacerbation of general pain was also significantly lower in the deprescribed group versus the usual care group (5.4% vs. 11.4%, P = 0.02). There was no difference between the deprescribed and usual care groups for the other 7 pain diagnoses (Table 2).<br><br>The adjusted OR for the deprescribed group experiencing exacerbation of pain was 0.50 (95% CI = 0.33-0.77, P < 0.01) in comparison with the usual care group (Table 3). | | 922, Table 3 |
| **No. missing participants** | 89 | 261 | NA |
| **Reasons missing** | Difference is numbers is because patients were selected differently than for effectiveness outcomes – must have had baseline pain diagnosis | Difference is numbers is because patients were selected differently than for effectiveness outcomes – must have had baseline pain diagnosis | 922 Table 2 |
| **Statistical methods used and appropriateness of these** | Conditional logistic regressions were conducted to determine the risk of GI bleed events, AKI, and exacerbation of pain | | 921 |
| **Confounders** | For effectiveness and safety outcomes, the common independent variables used in the conditional logistic regression model included age, sex, CCI score, Caucasian, NSAID classes, and 8 pain diagnoses. for risk of AKI and exacerbation of pain, we also adjusted for renal disease. | | 921 |
| **Notes** | None | | |

## Conclusions

| | Description as stated in report/paper | Page number |
|---|---|---|
| **Key conclusions of study authors** | The outcomes analysis showed a nonsignificant trend towards reduced rates of GI bleed events and AKI between the deprescribed group and the usual care group. There was, however, a statistically significant 2-fold decrease in the rate of exacerbation of pain in the pharmacist-managed deprescribed group versus the usual care group | 924 |
| **Notes** | | |

## Rodriguez et al. 2020

### Study eligibility

| Study Characteristics | Eligibility criteria |
|---|---|
| Title | Association of Pharmacist Prescription With Dispensed Duration of Hormonal Contraception |
| Author (year) | Rodriguez et al. 2020 |
| Country | California, Colorado, Hawaii, Oregon- USA |
| Type of study | Prospective cohort |
| Participants | Women seeking contraception (first time) |
| Types of intervention | Pharmacist prescribing |
| Types of comparison | Clinician prescribing |
| Types of outcome measures | Months of hormonal contraception dispensed; potential contraindications |
| Prescriptive authority | Independent (restricted) |
| Include/Exclude | Include |
| Notes | Linked to Rodriguez et al. 2021 |

**DO NOT PROCEED IF STUDY EXCLUDED FROM REVIEW**

## Characteristics of included studies

### Methods

| | Descriptions as stated in report/paper | | Page number |
|---|---|---|---|
| **Aim of study** | The purpose of this study was to test whether there are differences in the amount of contraceptive supply dispensed by pharmacists compared with clinicians. | | 2 |
| **Design** | Prospective cohort study | | 3 |
| **Unit of allocation** *(by individuals, cluster/ groups or body parts)* | Individuals | | 3 |
| **Start-end date** | January 30, 2019-November 1, 2019 | | |
| **Duration of participation** *(from recruitment to last follow-up/ baseline to last follow-up- group level)* *1. Time of consent until last measurement for each individual.* *2. Baseline to final follow-up for each individual* | The full study is powered to detect a 10% difference in contraceptive continuation rates at 1 year. | | 2 |
| **Study duration** *(as above with the exception of interim analyses or other circumstances* | The full study is powered to detect a 10% difference in contraceptive continuation rates at 1 year. | | 2 |
| **Ethical approval needed/obtained for study** | Yes | The institutional review board at Oregon Health & Science University approved the study protocol. | 2 |
| **Notes** | | | |

### Participants

| | **Description** *Include comparative information for each intervention or comparison group if available* | Page number |
|---|---|---|

| | | | |
|---|---|---|---|
| **Population description** *(from which study participants are drawn)* | Women aged 18 to 50 years who received at least 1 month of hormonal contraception (pill, patch, ring, or injectable) from a clinician or pharmacist prescription. | | 3 |
| **Setting** *(including location and social context)* | Community pharmacies | | 3 |
| **Inclusion/exclusion criteria** | We recruited women aged 18 to 50 years who received at least 1 month of hormonal contraception (pill, patch, ring, or injectable) from a clinician or pharmacist prescription. Only individuals who provided the method of contraception and number of months of contraception they received were included in the study. Participants indicating they were using the method for a medical reason (eg, dysmenorrhea or menorrhagia) or that they had a concomitant permanent form of contraception were excluded. | | 3 |
| **Method of recruitment of participants** | In-person. Pharmacy staff at these sites were trained to provide eligible women with sealed recruitment packets | | 3 |
| **Informed consent obtained** | Yes | The institutional review board at Oregon Health & Science University approved the study protocol, and all participants provided written informed consent. Participants had the option of completing a paper version of the consent form and survey provided in the packet or following a link to an online version of the consent form and survey | 2, 3 |
| **Total no. randomised** *(or total pop. at start of study for NRCTs)* | 448 individuals | | eFigure 2 |
| **Clusters** | California, 46 pharmacies; Colorado, 14 pharmacies; Hawaii, 10 pharmacies; and Oregon, 69 pharmacies | | 2 |
| **Baseline imbalances** | Women receiving contraception from a pharmacist tended to have a lower level of education and were slightly younger; 82 pharmacist prescriptions (56.9%) were to women aged 18 to 24 years vs 115 clinician prescriptions (43.2%) (P = .03), and 38 women (26.4%) with a pharmacist prescription had a bachelor degree vs 100 women (37.6%) with a clinician prescription (P = .002) (Table 1). A greater proportion of women seeking contraception from a pharmacist (16 [11.1%]) than from a clinician (8 [3.0%]) reported being uninsured (P = .001). | | 4 |
| **Withdrawals and exclusions** | Pharmacist group: Excluded due to missing outcome variable (n = 10)<br>Clinician group: Excluded due to ineligibility (n = 12) or missing outcome variable (n= 16) | | eFigure 2 |

| | | |
|---|---|---|
| **Age** | Intervention<br>18-24 years: 56.94%<br>25-39 years: 18.06%<br>30-34 years: 15.28%<br>≥35 years: 9.72%<br><br>Comparator<br>18-24 years: 43.23%<br>25-39 years: 23.31%<br>30-34 years: 15.79%<br>≥35 years: 17.67% | 5 (table 1) |
| **Sex (female)** | Intervention: 100%<br>Comparator: 100% | 2 |
| **Subgroups measure** | Not reported | NA |
| **Subgroups reported** | We examined missingness in our data. We examined demographic characteristics by outcome missingness, and there were no significant differences between those with and without outcome data. All demographic variables were missing under 1% of responses. We conducted several sensitivity analyses. First, we ran models that included previous use of the method and pregnancy intention variables. We also ran a model restricted to the subsample of insured women (n = 385). Finally, we ran models making assumptions about outcomes among those missing outcome data. All results were robust to these changes, and we include only our final model. All analyses were<br>performed using Stata 16.0 (StataCorp LP). P < .05, using a 2-sided test, indicated statistical significance. Data were analyzed from October 1 to November 25, 2019. | 4 |
| **Notes** | None | |

## Intervention group

| | Description as stated in report/paper | Page number |
|---|---|---|
| **Group name** | Pharmacist prescribing | 5 (table 1) |
| **No. randomised/assigned to group** *(specify whether no. people or clusters)* | 144 individuals | 5 (table 1) |
| **Description** *(include sufficient detail for replication, e.g. content, dose, components)* | We recruited women aged 18 to 50 years who received at least 1 month of hormonal contraception (pill, patch, ring, or injectable) from a clinician or pharmacist prescription | 3 |

| | | |
|---|---|---|
| **Duration of treatment period** | No set treatment period. | NA |
| **Timing** | When prescribed | 3 |
| **Co-interventions** | Not reported | NA |
| **Notes** | None | |

## Comparator group

| | **Description as stated in report/paper** | **Page number** |
|---|---|---|
| **Group name** | Clinician prescribing | 5 (table 1) |
| **No. randomised/assigned to group** *(specify whether no. people or clusters)* | 266 individuals | 5 (table 1) |
| **Description** *(include sufficient detail for replication, e.g. content, dose, components)* | We recruited women aged 18 to 50 years who received at least 1 month of hormonal contraception (pill, patch, ring, or injectable) from a clinician or pharmacist prescription | 3 |
| **Duration of treatment period** | No set treatment period. | NA |
| **Timing** | When prescribed | 3 |
| **Co-interventions** | Not reported | NA |
| **Notes** | None | |

## Outcomes

### Potential contraindication

| | **Description as stated in report/paper** | **Page number** |
|---|---|---|
| **Outcome name** | Potential contraindication | 3 |
| **Time points measured** *(specify whether from start or end of intervention)* | After prescribed | 3 |
| **Time points reported** | After prescribed | 3 |

| | | | |
|---|---|---|---|
| **Outcome definition** *(with diagnostic criteria if relevant)* | Our secondary outcome was the proportion of prescriptions containing estrogen for individuals with a potential contraindication to a combined method. We asked individuals if they had health conditions or behaviors that indicated a potential contraindication to hormonal contraceptive use. We utilized the wording and checklist approach validated in previous studies to elicit information on potential contraindications to estrogen. Health conditions classified as category 3 or 4 by the Centers for Disease Control and Prevention's Medical Eligibility Criteria for contraceptive use were used to define a potential contraindication. Women receiving estrogen-containing methods were flagged as having a potential contraindication to combined hormonal contraception if they had history of migraines with aura, history of blood clots, or hypertension or were over 40 years of age with hypertension or current tobacco use (multiple risk factors for atherosclerotic cardiovascular disease). | | 3 |
| **Unit of measurement** *(if relevant)* | Event data | | 7 (table 5) |
| **Scales: upper and lower limits** *(indicate whether high or low score is good)* | Lower score is desired | | NA |
| **Is outcome/tool validated?** | No | Survey self report | NA |
| **Imputation of missing data** *(e.g. assumptions made for ITT analysis)* | We examined missingness in our data. We examined demographic characteristics by outcome missingness, and there were no significant differences between those with and without outcome data. All demographic variables were missing under 1% of responses. We conducted several sensitivity analyses. First, we ran models that included previous use of the method and pregnancy intention variables. We also ran a model restricted to the subsample of insured women (n = 385). Finally, we ran models making assumptions about outcomes among those missing outcome data. All results were robust to these changes, and we include only our final model. All analyses were performed using Stata 16.0 (StataCorp LP). P < .05, using a 2-sided test, indicated statistical significance. Data were analyzed from October 1 to November 25, 2019. | | NA |
| **Assumed risk estimate** *(e.g. baseline or population risk noted in Background)* | Not reported | | NA |

| | | |
|---|---|---|
| **Power** *(e.g. power & sample size calculation, level of power achieved)* | The full study is powered to detect a 10% difference in contraceptive continuation rates at 1 year. However, this study was not powered to examine differences in prescription of contraception to women with potential contraindications to estrogen is needed. | 2<br>8 |
| **Notes** | None | |

## Funding/conflict of interest

| | | |
|---|---|---|
| **Study funding sources** *(including role of funders)* | This study was funded by a grant from Arnold Ventures. REDCap, through the Oregon Health & Science University, was utilized for this study; as such, this research was supported by the National Center for Advancing Translational Sciences of the National Institutes of Health under award number UL1TR0002369. | 10 |
| **Possible conflicts of interest** *(for study authors)* | Dr Rodriguez reported receiving grants from Arnold Ventures during the conduct of the study; grants from the National Institute on Minority Health and Health Disparities, personal fees from the American Congress of Obstetricians and Gynecologists, and personal fees from Merck and Bayer outside the submitted work. Dr Edelman reported receiving grants from Merck, research monies from HRA Pharma, and author royalties from Up To Date and serving as a consultant for the World Health Organization, the Centers for Disease Control and Prevention, Gynuity Health Projects, FHI 360, Exeltis, and Nexplanon and as a trainer for Merck (with all honorariums declined since 2016) outside the submitted work. Dr Anderson reported receiving grants from Arnold Ventures during the conduct of the study and speaker honoraria from the American Society of Health Systems Pharmacy. No other disclosures were reported. | 9 |
| **Notes** | None | |

## Data and analysis

### Potential contraindications

| | **Description as stated in report/paper** | | | | **Page number** |
|---|---|---|---|---|---|
| **Outcome** | Potential contraindications | | | | 7 (table 5) |
| **Time point** *(specify from start or end of intervention)* | After prescribing | | | | 3 |
| **Results** | Intervention | | Comparison | | 7 (table 5) |
| | No. with event | Total in group | No. with event | Total in group | |

| | | | | | |
|---|---|---|---|---|---|
| | 14 | 144 | 32 | 266 | |
| **Any other results reported** | There was no significant difference between pharmacists and clinicians in prescription of a progestin-only method of contraception among women with a potential contraindication to estrogen (8 [20.0%] vs 6 [30.0%] prescriptions; P = 0.52) (table 5). | | | | 5 |
| **No. missing participants** | n = 10 | | n = 28 | | eFigure 2 |
| **Reasons missing** | Excluded due to missing outcome variable | | Excluded due to ineligibility or missing outcome variable | | eFigure 2 |
| **Statistical methods used and appropriateness of these** | We used descriptive statistics and bivariate tests (χ2 or Fisher exact for small cell sizes) of association to characterize the sample by provider type (pharmacist vs clinician) and outcome (6-month supply dispensed). We described number of months of contraceptive supply dispensed and the presence of potential contraindications to estrogen by provider type. We developed a multivariable logistic regression model to evaluate the association between provider type and our outcome (greater than or less than a 6-month supply dispensed). Model covariates included age, insurance status, race/ ethnicity, rural residence, and state of residence. We included state to control for any unmeasured differences in dispensing practices by state. We selected these covariates a priori based on factors associated with method discontinuation and unintended pregnancy.2,33-35 We chose not to include previous use of the method and pregnancy intention, because these were not different by provider type or outcome in bivariate analyses. | | | | 4 |
| **Confounders** | Included age, insurance status, race/ ethnicity, rural residence, and state of residence<br><br>Previous use of the method and pregnancy intention variables. We also ran a model restricted to the subsample of insured women (n = 385). Finally, we ran models making assumptions about outcomes among those missing outcome data. | | | | 4 |
| **Notes** | None | | | | |

## Conclusions

| | **Description as stated in report/paper** | **Page number** |
|---|---|---|

| | | |
|---|---|---|
| **Key conclusions of study authors** | We also found that pharmacists are as likely as clinicians to prescribe a safer, progestin only method for women with a potential contraindication to estrogen. Although there was no difference by provider type, our analysis was underpowered to study this outcome. We did still find that a meaningful proportion (71.7%) of women with potential contraindications received an estrogen-containing method across provider type. This is a higher rate than what has previously been reported in the literature.36,37 We believe that it is unlikely that a woman with a true absolute contraindication to estrogen received an estrogen-containing method from either provider type for the following reasons. | 8 |
| **Notes** | None | |

## Rodriguez et al. 2021a

### Study eligibility

| **Study Characteristics** | **Eligibility criteria** |
|---|---|
| **Title** | Association of pharmacist prescription and 12-month contraceptive continuation rates |
| **Author (year)** | Rodriguez et al. 2021a |
| **Country** | California, Colorado, Hawaii, Oregon- USA |
| **Type of study** | Prospective cohort |
| **Participants** | Women seeking contraception (first time) |
| **Types of intervention** | Pharmacist prescribing |
| **Types of comparison** | Clinician prescribing |
| **Types of outcome measures** | 1. 12-month contraception continuation (effective method use)<br>2. Perfect use across 12-months |
| **Prescriptive authority** | Independent (restricted) |
| **Include/Exclude** | Include |
| **Notes** | |

**DO NOT PROCEED IF STUDY EXCLUDED FROM REVIEW**

## Characteristics of included studies

### Methods

| | Descriptions as stated in report/paper | | Page number |
|---|---|---|---|
| **Aim of study** | To determine whether 12-month rates of continuation of an effective form of contraception or perfect use of contraception differ by prescribing provider (pharmacist or clinician). | | e1 |
| **Design** | Prospective cohort | | E1 |
| **Unit of allocation** *(by individuals, cluster/ groups or body parts)* | Individual | | E1 |
| **Start-end date** | January 2019 through November 2019 | | E2 |
| **Duration of participation** *(from recruitment to last follow-up/ baseline to last follow-up- group level)* *1. Time of consent until last measurement for each individual.* *2. Baseline to final follow-up for each individual* | 12 months | | E2 |
| **Study duration** *(as above with the exception of interim analyses or other circumstances* | 12 months | | E2 |
| **Ethical approval needed/obtained for study** | Yes | The institutional review board at Oregon Health & Science University approved the study protocol. | E2 |
| **Notes** | | | |

### Participants

| | **Description** *Include comparative information for each intervention or comparison group if available* | Page number |
|---|---|---|
| **Population description** *(from which study participants are drawn)* | Women 18-50 presenting to a pharmacy to fill a new prescription from a clinician or initiate a new prescription with a pharmacist | E2 |

| | | | |
|---|---|---|---|
| **Setting** *(including location and social context)* | Community pharmacies in California, Colorado, Hawaii, and Oregon | | E2 |
| **Inclusion/exclusion criteria** | Inclusion: women 18-50 presenting at a pharmacy to fill or initiate a new prescription for contraception (pill, patch, ring, or injectable; CO only allows pill/patch) for prevent pregnancy<br>Exclusion: using contraception for non-contraceptive benefit; not meeting age restriction | | E2 |
| **Method of recruitment of participants** | In-person. Pharmacy staff at these sites were trained to identify eligible women and to provide them with sealed recruitment packets | | E2 |
| **Informed consent obtained** | Yes | Written informed consent was obtained from all participants | E2 |
| **Total no. randomised** *(or total pop. at start of study for NRCTs)* | 456 enrolled<br>388 consented and met eligibility criteria | | E3 Figure 1 |
| **Clusters** | 46 pharmacies in California<br>14 in Colorado<br>10 in Hawaii<br>69 in Oregon | | E2 |
| **Baseline imbalances** | Women with prescriptions from clinicians reported higher levels of education; 21.2% had a master's degree or higher, compared with 12.9% of women with pharmacist prescriptions (=.02). Women with prescriptions from pharmacists were more likely to be uninsured (11.2% pharmacist vs 2.6% clinician; P=.006) and to pay out of pocket (25.8% pharmacist vs 8.7% clinician; P<.001). | | E4 |
| **Withdrawals and exclusions** | 48 lost to follow-up (did not complete 12-month follow up) | | E4, Figure 1 |
| **Age** | Intervention: 18-50, 45.2% 18-24 (no average given)<br>Comparator: 18-50, 56.1% 18-24 (no average given) | | E5 |
| **Sex (female)** | Intervention: 100%<br>Comparator: 100% | | E4, e5 |
| **Subgroups measure** | None reported | | NA |
| **Subgroups reported** | None reported | | NA |
| **Notes** | | | |

## Intervention group

| | Description as stated in report/paper | Page number |
|---|---|---|
| **Group name** | Pharmacist prescribing | E2 |

| | | |
|---|---|---|
| **No. randomised/assigned to group** *(specify whether no. people or clusters)* | 132 | E5 |
| **Description** *(include sufficient detail for replication, e.g. content, dose, components)* | Women presented to pharmacies either to fill a new prescription from a clinician or initiate a new prescription with the pharmacist.<br>Women who consented to participate completed a baseline survey about demographic and clinical characteristics and their experience receiving contraception at the Pharmacy. Participants completed follow-up surveys at 3, 6, and 12 months following baseline to assess continuation, perfect use, method switching, and any reported side effects. | E2 |
| **Duration of treatment period** | No set treatment period. Continuation evaluated at 12 months. | 32 |
| **Timing** | Not reported | NA |
| **Co-interventions** | None reported | NA |
| **Notes** | | |

## Comparator group

| | Description as stated in report/paper | Page number |
|---|---|---|
| **Group name** | Clinician prescribing | E2 |
| **No. randomised/assigned to group** *(specify whether no. people or clusters)* | 208 | E5 |
| **Description** *(include sufficient detail for replication, e.g. content, dose, components)* | Women presented to pharmacies either to fill a new prescription from a clinician or initiate a new prescription with the pharmacist.<br>Women who consented to participate completed a baseline survey about demographic and clinical characteristics and their experience receiving contraception at the Pharmacy. participants completed follow-up surveys at 3, 6, and 12 months following baseline to assess continuation, perfect use, method switching, and any reported side effects. | E2 |
| **Duration of treatment period** | No set treatment period. Continuation evaluated at 12 months. | E2 |
| **Timing** | Not reported | NA |
| **Co-interventions** | None reported | NA |
| **Notes** | | |

## Outcomes

### 12-month contraception continuation

| | Description as stated in report/paper | | Page number |
|---|---|---|---|
| **Outcome name** | 12-month contraception continuation | | E4 |
| **Time points measured** *(specify whether from start or end of intervention)* | 3, 6, 12 months | | E4 |
| **Time points reported** | 12 months (3 and 6 months are factored into this) | | E4 |
| **Outcome definition** *(with diagnostic criteria if relevant)* | Contraceptive methods were classified by tiers of efficacy. Women whose method at 12-month follow-up was a Tier 1 or Tier 2 method (intrauterine devices IUD, implant, pill, patch, ring, injection) were classified as continuing an effective method at 12 months.<br><br>Women who reported using the same method across all time points were classified as continuing their baseline Tier 2 method (pill, patch, ring, injection). Women who reported switching their method during follow-up were classified as switching to a Tier 1 method (IUD, implant), switching to a different Tier 2 method, switching to a Tier 3 method (condoms, withdrawal, natural family planning, breastfeeding, emergency contraception), switching to no method, or stopping their contraception because of a desire for pregnancy | | E4 |
| **Unit of measurement** *(if relevant)* | Binary, use of an effective form or no | | E5, e6 Table 2 |
| **Scales: upper and lower limits** *(indicate whether high or low score is good)* | Yes (use), no | | E5 |
| **Is outcome/tool validated?** | No | | NA |
| **Imputation of missing data** *(e.g. assumptions made for ITT analysis)* | Not reported | | NA |
| **Assumed risk estimate** *(e.g. baseline or population risk noted in Background)* | Not reported | | NA |
| **Power** *(e.g. power & sample size calculation, level of power achieved)* | The study was powered to detect a 10% difference in continuation of an effective contraceptive method at 1 year. | | E2 |

| | |
|---|---|
| **Notes** | |

## Perfect use across 12-months

| | Description as stated in report/paper | | Page number |
|---|---|---|---|
| **Outcome name** | Perfect use across 12-months | | E5 |
| **Time points measured** *(specify whether from start or end of intervention)* | 3, 6, 12 months<br>We asked participants if they had missed any days of contraception at all follow-up time points and if so, how many days per month did they not use their method (this measure included all participants, regardless of switching methods). | | E4 |
| **Time points reported** | All – composited into 12 months | | E5 |
| **Outcome definition** *(with diagnostic criteria if relevant)* | Reporting no missed days of contraception at all follow-up time points. | | E5 |
| **Unit of measurement** *(if relevant)* | Binary – no missed days vs. any missed days | | E5 |
| **Scales: upper and lower limits** *(indicate whether high or low score is good)* | No missed days is desired | | NA |
| **Is outcome/tool validated?** | No | | NA |
| **Imputation of missing data** *(e.g. assumptions made for ITT analysis)* | We conducted a sensitivity analysis for our outcome examining perfect use. Nearly 10% of the sample were missing data at least 1 time point. We therefore explored 3 versions of this outcome: the first utilized available data and ignored the missing values, the second classified participants with any missing time points as having missed some days, and the third classified participants with any missing time points as having perfect use. Model results were robust across all 3 versions, and we used the first version (ignoring missing data) of the variable in our final model | | E3 |
| **Assumed risk estimate** *(e.g. baseline or population risk noted in Background)* | Not reported | | NA |
| **Power** *(e.g. power & sample size calculation, level of power achieved)* | Not reported | | NA |
| **Notes** | | | |

## Funding/conflict of interest

| | | |
|---|---|---|
| **Study funding sources** *(including role of funders)* | This study was funded by a grant from Arnold Ventures. through Oregon Health & Science University. REDCAP was used for this study, and as such, this research was supported by the National Center for Advancing Translational Sciences of the National Institutes of Health under award number UL1TR0002369 | E8 |
| **Possible conflicts of interest** *(for study authors)* | M.I.R. reported receiving grants from Arnold Ventures during the conduct of the study, grants from the National Institute on Minority Health and Health Disparities, personal fees from the American Congress of Obstetricians and Gynecologists, and personal fees from Merck and Bayer outside the submitted work. A.B.E. reported receiving grants from Merck, research monies from HRA Pharma, and author royalties from UpToDate and serving as a consultant for the World Health Organization, the Centers for Disease Control and Prevention, Gynuity Health Projects, FHI 360, and Nexplanon and as a trainer for Merck (with all honoraria declined since 2016) outside the submitted work. L.A. reported receiving speaker honoraria from the American Society of Health-System<br>Pharmacists. B.G.D. reported receiving grants where she is the principal investigator from Merck and the Office of Populations Affairs. No other disclosures were reported. | E8 |
| **Notes** | | |

## Data and analysis

### 12-month contraception continuation

| | Description as stated in report/paper | | | | Page number |
|---|---|---|---|---|---|
| **Outcome** | 12-month contraception continuation | | | | E4 |
| **Time point** *(specify from start or end of intervention)* | 12 months | | | | E4 |
| **Results** | Intervention | | Comparison | | E6, Table 2 |
| | No. with event | Total in group | No. with event | Total in group | |
| | 119 | 132 | 186 | 208 | |

| | | | |
|---|---|---|---|
| **Any other results reported** | The overwhelming majority (90%) of study participants reported using an effective method of contraception at 12 months with no differences by provider prescribing type. The majority of women continued to use a Tier 2 method (80.8% [clinician] and 82.4% [pharmacist] of women at 12 months) (Table 2).<br><br>We found no difference in provider prescribing type for those women that switched from a Tier 2 to a Tier 1 method (IUD or implant) (9.6% clinician vs 6.9% pharmacist), a Tier 3 method (4.3% clinician vs 5.3% pharmacist), or no method (3.9% clinician vs 4.6% pharmacist) (=.81).<br><br>Pharmacist provider type was not significantly associated with continued use of an effective contraceptive method at 12 months (adjusted odds ratio 0.70 CI 0.28 – 1.71) controlling for other woman-level characteristics. | | E4 |
| **No. missing participants** | 17 | 31 | E9 |
| **Reasons missing** | Not reported. Loss to follow-up | Not reported. Loss to follow-up | E9 |
| **Statistical methods used and appropriateness of these** | We subsequently used multivariable logistic regression to develop 2 models to test the association of provider type with our outcomes (use of effective contraception and perfect use at 12 months), controlling for sociodemographic and clinical characteristics.<br><br>We included binary age at baseline, baseline race and ethnicity, baseline education level, insurance status at 3 months, baseline payment for contraception out of pocket, baseline pregnancy intentions, side effects across the study period, and amount dispensed at baseline as covariates.<br><br>In both models, we selected these covariates based on previous literature suggesting that they are associated with continuation of an effective method and gaps in coverage We did not include contraceptive and medical variables that were not significantly different by provider type in bivariate analysis (experience with contraception, history of pregnancy, openness to pregnancy in the next 12 months). | | E3 |
| **Confounders** | Baseline variables: age at baseline, race and ethnicity, education level, payment for contraception out of pocket, pregnancy intentions, and amount dispensed; side effects across the study period and insurance status at 3 months, | | E3 |
| **Notes** | None | | |

## Perfect use across 12-months

| | Description as stated in report/paper | | | | Page number |
|---|---|---|---|---|---|
| **Outcome** | Perfect use across 12-months | | | | E5 |
| **Time point** *(specify from start or end of intervention)* | 12 months | | | | E5 |
| **Results** | Intervention | | Comparison | | E6, Table 2 |
| | No. with event | Total in group | No. with event | Total in group | |
| | 62 | 132 | 112 | 208 | |
| **Any other results reported** | We found no difference in perfect use of contraception by prescriber type; 53.9% of women receiving contraception from a clinician and 47.0% from a pharmacist reported no missed days of use at 12 months.<br><br>Pharmacist provider type was not significantly associated with perfect use (adjusted odds radio 0.87 CI 0.51-1.48) controlling for other woman-level characteristics. | | | | E5 |
| **No. missing participants** | 17 | | 31 | | E9 |
| **Reasons missing** | Not reported. Loss to follow-up | | Not reported. Loss to follow-up | | E9 |
| **Statistical methods used and appropriateness of these** | We subsequently used multivariable logistic regression to develop 2 models to test the association of provider type with our outcomes (use of effective contraception and perfect use at 12 months), controlling for sociodemographic and clinical characteristics.<br><br>We included binary age at baseline, baseline race and ethnicity, baseline education level, insurance status at 3 months, baseline payment for contraception out of pocket, baseline pregnancy intentions, side effects across the study period, and amount dispensed at baseline as covariates.<br><br>In both models, we selected these covariates based on previous literature suggesting that they are associated with continuation of an effective method and gaps in coverage<br>We did not include contraceptive and medical variables that were not significantly different by provider type in bivariate analysis (experience with contraception, history of pregnancy, openness to pregnancy in the next 12 months). | | | | E3 |

| | | |
|---|---|---|
| **Confounders** | Baseline variables: age at baseline, race and ethnicity, education level, payment for contraception out of pocket, pregnancy intentions, and amount dispensed; side effects across the study period and insurance status at 3 months, | E3 |
| **Notes** | None | |

## Conclusions

| | **Description as stated in report/paper** | **Page number** |
|---|---|---|
| **Key conclusions of study authors** | Pharmacist prescription of contraception appears similar to clinic-based prescribing in promoting correct and consistent use of contraception methods after initiation among women who do not desire a pregnancy.<br>Continuing to expand contraceptive services in pharmacies—with equitable insurance coverage—is needed. | E7 |
| **Notes** | | |

# Rodriguez et al. 2021b

## Study eligibility

| **Study Characteristics** | **Eligibility criteria** |
|---|---|
| **Title** | Medical contraindications to combined hormonal contraceptive use among women using methods prescribed by a pharmacist |
| **Author (year)** | Rodriguez et al. (2021b) |
| **Country** | Oregon, USA |
| **Type of study** | Retrospective cohort |
| **Participants** | Women in Oregon (ages 12–51) who had at least one prescription for contraception from 2016 to 2018. |
| **Types of intervention** | Pharmacist prescribed |
| **Types of comparison** | Clinician prescribed |
| **Types of outcome measures** | Proportion of women receiving a combined hormonal method who had a Medical Eligibility Category (MEC) 3 or 4 condition; potential contraindications; prescription error |
| **Prescriptive authority** | Independent |
| **Include/Exclude** | Include |
| **Notes** | None |

**DO NOT PROCEED IF STUDY EXCLUDED FROM REVIEW**

## Characteristics of included studies

### Methods

| | Descriptions as stated in report/paper | | Page number |
|---|---|---|---|
| **Aim of study** | To determine whether pharmacist prescription of contraception is associated with inappropriate prescription to women with medical contraindications | | 548 |
| **Design** | Retrospective cohort study | | 548 |
| **Unit of allocation** *(by individuals, cluster/ groups or body parts)* | Individuals | | 548 |
| **Start-end date** | January 2016- December 2018 | | 548 |
| **Duration of participation** *(from recruitment to last follow-up/ baseline to last follow-upgroup level) 1. Time of consent until last measurement for each individual. 2. Baseline to final follow-up for each individual* | Not reported | | NA |
| **Study duration** *(as above with the exception of interim analyses or other circumstances* | Not reported | | NA |
| **Ethical approval needed/obtained for study** | Not reported | NA | NA |
| **Notes** | None | | |

### Participants

| | **Description** *Include comparative information for each intervention or comparison group if available* | Page number |
|---|---|---|

| | | | |
|---|---|---|---|
| **Population description** *(from which study participants are drawn)* | Women in Oregon (ages 12–51) who had at least one prescription for contraception from 2016 to 2018. | | 548 |
| **Setting** *(including location and social context)* | Community pharmacy | | 548 |
| **Inclusion/exclusion criteria** | Our study population included women in Oregon (ages 12–51) who had at least one prescription for contraception from 2016 to 2018. We included women with both commercial and Medicaid insurance at any point during our study period and imposed no enrollment restrictions. We excluded Medicaid members dually enrolled in Medicare from our study population. We obtained enrollment data, medical and pharmacy claims from the Oregon Health Authority. We utilized Medicaid claims for Medicaid enrollees and the Oregon All Payer All Claims data for commercially insured enrollees. The All Payer All Claims database contains claims from the largest 25 commercial insurers in Oregon, excluding information from small carriers with < 50 0 0 enrollees and from certain federal programs such as the Federal Employee Health Benefits Program. It contains claims on over 93% of state residents [14]. We restricted our sample to women using contraceptive methods that can be prescribed by either a pharmacist or a clinician: progestin injectable, oral contraceptive pills (combined hormonal and progestin only), combined hormonal patch and ring. We focused our analysis on women using contraception to prevent pregnancy, and not to manage other medical conditions. We therefore excluded individuals who were not biologically capable of pregnancy (e.g., diagnoses of menopause, permanent contraception) [15] . We identified receipt of at least one eligible contraceptive using National Drug Classification codes. | | 548 |
| **Method of recruitment of participants** | Oregon's All Payer All Claims database | | 548 |
| **Informed consent obtained** | Not reported | NA | NA |
| **Total no. randomised** *(or total pop. at start of study for NRCTs)* | 439,240 individuals | | 549 |
| **Clusters** | No | | NA |

| | | |
|---|---|---|
| **Baseline imbalances** | Women aged 25 to29 were the most likely to receive contraception from a pharmacist (aOR 2.74, 95% confidence interval [CI] 2.44–3.08), whereas younger adolescent women (ages 12–17) were much less likely to receive contraception from a pharmacist (aOR 0.25, 95% CI 0.18–0.35). Pharmacist prescriptions were also slightly less likely in rural areas (aOR 0.78, 95% CI 0.69–0.89) and among women on Medicaid, relative to those with commercial insurance (aOR 0.21, 95% CI 0.19–0.24). Pharmacist prescriptions were more common in 2017 relative to 2016, but appeared relatively stable between 2017 and 2018. | 549 |
| **Withdrawals and exclusions** | Not reported | NA |
| **Age** | 12-17 years: 11.4%<br>18-24 years: 31.3%<br>25-29 years: 21.8%<br>30-34 years: 16.8%<br>≥35 years: 17.6% | 549 (table 1) |
| **Sex (female)** | Intervention: 100%<br>Comparator: 100% | NA |
| **Subgroups measure** | Not reported | NA |
| **Subgroups reported** | Not reported | NA |
| **Notes** | None | |

## Intervention group

| | **Description as stated in report/paper** | **Page number** |
|---|---|---|
| **Group name** | Pharmacist prescribed | 549 |
| **No. randomised/assigned to group** *(specify whether no. people or clusters)* | 3,782 individuals | 550 (table 3) |

| | | |
|---|---|---|
| **Description** *(include sufficient detail for replication, e.g. content, dose, components)* | In Oregon, the scope of clinical pharmacists includes contraceptive counseling, screening and prescribing combined hormonal methods (pill, patch, or ring) or progestin only methods (pill or injectable). Pharmacists complete a checklist with individuals requesting contraception, to identify possible medical contraindications to combined hormonal contraceptive use, and either offer a progestin only method or refer women [6] .<br>By the Board of Pharmacy's algorithm, Oregon pharmacists may not prescribe and must refer women who self-identify as having a CDC MEC condition that suggests risk outweighs the benefit of contraceptive use (Categories 3 & 4). | 548 |
| **Duration of treatment period** | Prescribed at baseline | NA |
| **Timing** | Not reported | NA |
| **Co-interventions** | Not reported | NA |
| **Notes** | None | |

## Comparator group

| | **Description as stated in report/paper** | **Page number** |
|---|---|---|
| **Group name** | Clinician prescribed | 549 |
| **No. randomised/assigned to group** *(specify whether no. people or clusters)* | 435,458 individuals | 550 (table 3) |
| **Description** *(include sufficient detail for replication, e.g. content, dose, components)* | We restricted our sample to women using contraceptive methods that can be prescribed by either a pharmacist or a clinician: progestin injectable, oral contraceptive pills (combined hormonal and progestin only), combined hormonal patch and ring. | 548 |
| **Duration of treatment period** | Prescribed at baseline | NA |
| **Timing** | Not reported | NA |
| **Co-interventions** | Not reported | NA |
| **Notes** | None | |

# Outcomes

## Potential contraindications

| | Description as stated in report/paper | | Page number |
|---|---|---|---|
| **Outcome name** | Potential contraindiciations | | 549 |
| **Time points measured** *(specify whether from start or end of intervention)* | Prescribed at baseline | | NA |
| **Time points reported** | Prescribed at baseline | | NA |
| **Outcome definition** *(with diagnostic criteria if relevant)* | The contraindication categories are based on the CDC MEC for contraceptive use [2] . We used diagnosis codes consistent with CDC MEC Category 3 or 4 to identify possible contraindications to combined hormonal contraceptive use. A Category 3 condition is one for which the theoretical or proven risks usually outweigh the advantages of using the method.<br>Similarly, a Category 4 condition is one that represents an unacceptable health risk if the contraceptive method is used. For all contraindication categories except postpartum and breastfeeding, we identified conditions based on International Classification of Disease 9 or 10 diagnoses codes from all health encounters 12 months prior to the contraceptive prescription. Postpartum and breastfeeding contraindication extended the contraindication classification window for 21 days after pregnancy, delivery, or breastfeeding diagnosis. | | 548 |
| **Unit of measurement** *(if relevant)* | Event data | | NA |
| **Scales: upper and lower limits** *(indicate whether high or low score is good)* | Lower events desired | | NA |
| **Is outcome/tool validated?** | No | Electronic health record | NA |
| **Imputation of missing data** *(e.g. assumptions made for ITT analysis)* | Not reported | | NA |
| **Assumed risk estimate** *(e.g. baseline or population risk noted in Background)* | Not reported | | NA |
| **Power** *(e.g. power & sample size calculation, level of power achieved)* | Not reported | | NA |
| **Notes** | None | | |

## Funding/conflict of interest

| | | |
|---|---|---|
| **Study funding sources** *(including role of funders)* | Not reported | NA |
| **Possible conflicts of interest** *(for study authors)* | Dr. Rodriguez has served as a contraceptive trainer for ACOG, Bayer and Merck. All other authors report no conflicts of interest. | 551 |
| **Notes** | None | |

## Data and analysis

### Potential contraindications

| | Description as stated in report/paper | | | | Page number |
|---|---|---|---|---|---|
| **Outcome** | Potential contraindications | | | | 550 (table 3) |
| **Time point** *(specify from start or end of intervention)* | After prescription | | | | NA |
| **Results** | Intervention | | Comparison | | 550 (table 3) |
| | No. with event | Total in group | No. with event | Total in group | |
| | 34 (0.9%) | 3,782 total prescriptions | 18,490 (4.25%) | 435,458 total prescriptions | |
| **Any other results reported** | Overall, the rates of women with any potential contraindication who receive combined hormonal prescriptions are much smaller among women receiving prescriptions from pharmacists (0.90%) relative to clinicians (4.25%). Consequently, the error rate was relatively small among pharmacists (0.74%) relative to clinicians (2.11%). The contraindications that were most common among clinicians were hypertension (1.66%), pregnant or < 21 days after pregnancy diagnosis (0.71%), currently breastfeeding or < 21 days after breastfeeding (0.60%), and migraines with aura (0.59%). The contraindications that were most common among pharmacists were hypertension (0.34%), migraines with aura (0.21%), and thrombogenic mutation (0.13%). | | | | 549 |
| **No. missing participants** | NA | | NA | | NA |
| **Reasons missing** | NA | | NA | | NA |

| | | |
|---|---|---|
| **Statistical methods used and appropriateness of these** | We assessed differences in demographic and enrollment variables by prescriber type (clinician vs pharmacist) and payer (commercial vs Medicaid). We used a logistic regression to test the association between prescriber type and key demographic characteristics of our population (age, insurance type, rural location and year since pharmacists became eligible to prescribe). We clustered standard errors on the individual level to account for multiple observations in the study period. For all models, we report odds ratios and 95% confidence intervals. We conducted bivariate descriptive analyses of contraindication condition prevalence and combined hormonal contraceptive prescription error rate by prescriber type. The error rate was defined as the proportion of individuals with a medical contraindication who received a combined hormonal contraceptive prescription. Because of the small number of people prescribed combined hormonal contraception by a pharmacist with evidence of contraindication, we provide descriptive statistics for medical conditions when errors occur. All hypothesis tests were 2-sided, and we considered $p < 0.05$ to be statistically significant. All analyses were conducted using R, version 4.0.3 (R Project for Statistical Computing). | 548/549 |
| **Confounders** | Not reported | NA |
| **Notes** | None | |

## Conclusions

| | Description as stated in report/paper | Page number |
|---|---|---|
| **Key conclusions of study authors** | Our data support the hypothesis that pharmacists can safely screen women for medical contraindications to combined hormonal contraception. Overall, very few women had a Category 3 or 4 contraindication to combined hormonal contraceptive use. The vast majority of these women were appropriately prescribed a safe method. Both pharmacists and clinicians were similarly effective in identifying potential medical contraindications to estrogen use. These findings have important implications for both pharmacist prescription and over-the-counter access to hormonal contraception. | 549 |
| **Notes** | None | |

# Rodriguez et al. 2022

## Study eligibility

| Study Characteristics | Eligibility criteria |
|---|---|
| Title | Association of Pharmacist Prescription of Contraception With Breaks in Coverage |
| Author (year) | Rodriguez et al. 2022 |
| Country | Oregon, USA |
| Type of study | Retrospective cohort |
| Participants | New women users of short-acting contraception |
| Types of intervention | Pharmacist prescribing |
| Types of comparison | Clinician prescribing |
| Types of outcome measures | 12-month contraceptive continuation rate; break in contraceptive coverage |
| Prescriptive authority | Independent |
| Include/Exclude | Include |
| Notes | |

**DO NOT PROCEED IF STUDY EXCLUDED FROM REVIEW**

## Characteristics of included studies

### Methods

| | Descriptions as stated in report/paper | | Page number |
|---|---|---|---|
| **Aim of study** | To assess whether pharmacist prescription of combined hormonal contraception is associated with 12-month contraceptive continuation rates or breaks in contraceptive coverage | | 781 |
| **Design** | Retrospective cohort study | | 782 |
| **Unit of allocation** *(by individuals, cluster/ groups or body parts)* | Individual | | 782 |
| **Start-end date** | January 1, 2016 – December 31, 2018 | | 782 |
| **Duration of participation** *(from recruitment to last follow-up/ baseline to last follow-up- group level)* *1. Time of consent until last measurement for each individual.* *2. Baseline to final follow-up for each individual* | At least 12 months | | 782 |
| **Study duration** *(as above with the exception of interim analyses or other circumstances* | 3 years | | 782 |
| **Ethical approval needed/obtained for study** | Yes | This study was reviewed and approved by the Oregon Health & Science University institutional review board | 782 |
| **Notes** | | | |

### Participants

| | **Description** *Include comparative information for each intervention or comparison group if available* | **Page number** |
|---|---|---|

| | | | |
|---|---|---|---|
| **Population description** *(from which study participants are drawn)* | New women users of short-acting contraception | | 782 |
| **Setting** *(including location and social context)* | Oregon, not specified if any data is restricted to only a certain setting (e.g., only outpatient visits) | | 782 |
| **Inclusion/exclusion criteria** | Inclusion: between 12-51 years of age; new users of short-acting reversible contraception (any method of progestin injectable, oral contraceptive pills, combined hormonal patch, or the ring) who received their prescription between January 1, 2016 and December 31, 2017; has commercial insurance or Medicaid<br>Exclusion: dually enrolled Medicaid and Medicare; those with inaccurate or implausible contraceptive days; those not biologically capable of pregnancy; those using long-acting reversible contraception | | 782 |
| **Method of recruitment of participants** | Not applicable. Information gathered from medical, pharmacy, and insurance claims databases | | 782 |
| **Informed consent obtained** | No | Not indication of individual consent. This study was reviewed and approved by the Oregon Health & Science University institutional review board | 782 |
| **Total no. randomised** *(or total pop. at start of study for NRCTs)* | 172,325 | | 782 Figure 1 |
| **Clusters** | None reported | | NA |
| **Baseline imbalances** | Not reported. Group differences reported but these are not 'baseline' | | 784 |
| **Withdrawals and exclusions** | Not applicable | | NA |
| **Age** | 12-51; No means given;<br>Intervention: 50.5% 25-34<br>Comparator: 42.2% <25 | | 784 |
| **Sex (female)** | Intervention: 100%<br>Comparator: 100% | | 784 |
| **Subgroups measure** | Age at cohort entry, rurality (defined by Rural-Urban Commuting Area codes), payer (Medicaid or commercial), and calendar year of the initial prescription | | 783 |
| **Subgroups reported** | Those who did not discontinue their contraception within the first 6 months of use; age; rurality; payer | | 783, Figure 1, 784 Figure 2 |
| **Notes** | | | |

## Intervention group

| | Description as stated in report/paper | Page number |
|---|---|---|

| | | |
|---|---|---|
| **Group name** | Pharmacist prescribing | 783 |
| **No. randomised/assigned to group** *(specify whether no. people or clusters)* | 1512 | 784 Table 1 |
| **Description** *(include sufficient detail for replication, e.g. content, dose, components)* | In 2016, Oregon became the first state to implement legislation allowing pharmacists to independently prescribe hormonal contraception, including the pill, patch, or ring, directly to the patient without a traditional clinic visit. Pharmacists voluntarily elect to complete additional training and follow a prescribing algorithm, based on the World Health Organization's Medical Eligibility for Contraceptive Use intended to aid health care professionals in safely prescribing contraception. Since the implementation of this policy in Oregon, 16 other states have passed legislation to allow pharmacist prescription of hormonal contraception (without a physician's oversight in a collaborative practice agreement) | 782 |
| **Duration of treatment period** | 12 months | 782 |
| **Timing** | Not specified. Women seeking their first prescription between January 1, 2016 and December 31, 2017 | 782 |
| **Co-interventions** | None reported | NA |
| **Notes** | | |

## Comparator group

| | **Description as stated in report/paper** | **Page number** |
|---|---|---|
| **Group name** | Clinician prescribing | |
| **No. randomised/assigned to group** *(specify whether no. people or clusters)* | 170813 | 784 Table 1 |
| **Description** *(include sufficient detail for replication, e.g. content, dose, components)* | Not specified. Must have accepted Medicaid patients or at least one of 25 commercial insurers in Oregon (from the Oregon All Payer All Claims database) | 782 |
| **Duration of treatment period** | 12 months | 782 |
| **Timing** | Not specified. Women seeking their first prescription between January 1, 2016 and December 31, 2017 | 782 |
| **Co-interventions** | None reported | NA |
| **Notes** | | |

## Outcomes

### 12-month contraceptive continuation rate

| | Description as stated in report/paper | | Page number |
|---|---|---|---|
| **Outcome name** | 12-month contraceptive continuation rate | | 783 |
| **Time points measured** *(specify whether from start or end of intervention)* | 12 months | | 783 |
| **Time points reported** | 12 months | | 783 |
| **Outcome definition** *(with diagnostic criteria if relevant)* | We defined contraceptive continuation as ongoing contraceptive use with no breaks in use of 30 days or greater. The inverse, contraceptive discontinuation, was defined as at least 30 days without a new contraceptive prescription after the end date of the prior contraceptive prescription in the 12-month follow-up period. | | 783 |
| **Unit of measurement** *(if relevant)* | Yes/no | | 783 |
| **Scales: upper and lower limits** *(indicate whether high or low score is good)* | Yes/no | | 783 |
| **Is outcome/tool validated?** | No | | NA |
| **Imputation of missing data** *(e.g. assumptions made for ITT analysis)* | Not reported | | NA |
| **Assumed risk estimate** *(e.g. baseline or population risk noted in Background)* | Not reported | | NA |
| **Power** *(e.g. power & sample size calculation, level of power achieved)* | Not reported | | NA |
| **Notes** | | | |

### Break in contraceptive coverage

| | Description as stated in report/paper | Page number |
|---|---|---|
| **Outcome name** | Break in contraceptive coverage | 783 |

| | | | |
|---|---|---|---|
| **Time points measured** *(specify whether from start or end of intervention)* | 6 month period | | 783 |
| **Time points reported** | 6 month period | | 783 |
| **Outcome definition** *(with diagnostic criteria if relevant)* | We defined a break in contraceptive coverage as missing between 1 and 29 days from the end of one contraceptive prescription and the start of a subsequent prescription. | | 783 |
| **Unit of measurement** *(if relevant)* | Yes/no | | 783 |
| **Scales: upper and lower limits** *(indicate whether high or low score is good)* | Yes/no | | 783 |
| **Is outcome/tool validated?** | No | | NA |
| **Imputation of missing data** *(e.g. assumptions made for ITT analysis)* | Not reported | | NA |
| **Assumed risk estimate** *(e.g. baseline or population risk noted in Background)* | Not reported | | NA |
| **Power** *(e.g. power & sample size calculation, level of power achieved)* | Not reported | | NA |
| **Notes** | | | |

## Funding/conflict of interest

| | | |
|---|---|---|
| **Study funding sources** *(including role of funders)* | Arnold Ventures | 781 |
| **Possible conflicts of interest** *(for study authors)* | Maria I. Rodriguez reported receiving grants from the National Institute of Minority Health & Health Disparities during the conduct of the study and personal fees from the American College of Obstetricians and Gynecologists, Bayer, and Merck & Co outside the submitted work. These potential conflicts of interest were managed by the IRB at Oregon Health & Science University. The other authors did not report any potential conflicts of interest. | 781 |
| **Notes** | | |

## Data and analysis

### 12-month contraceptive continuation rate

<table>
<tr><th></th><th colspan="4">Description as stated in report/paper</th><th>Page number</th></tr>
<tr><td>Outcome</td><td colspan="4">12-month contraceptive continuation rate</td><td>783</td></tr>
<tr><td>Time point (specify from start or end of intervention)</td><td colspan="4">At end of intervention</td><td>783</td></tr>
<tr><td rowspan="3">Results</td><td colspan="2">Intervention</td><td colspan="2">Comparison</td><td rowspan="3">784</td></tr>
<tr><td>No. with event</td><td>Total in group</td><td>No. with event</td><td>Total in group</td></tr>
<tr><td>519</td><td>1,512</td><td>35871</td><td>170,813</td></tr>
<tr><td>Any other results reported</td><td colspan="4">Overall, 34.3% of women with prescriptions from a pharmacist were continuing their method at 12 months, as compared with 21.0% of people receiving a prescription from a clinician (P<0.05, Fig. 2 and Table 2).<br><br>The unadjusted odds ratio for contraception continuation at 12 months was OR 1.97 ( 95% CI 1.77–2.19) (table 3).<br><br>The adjusted odds ratio (aOR) of contraceptive continuation at 12 months were 61% higher for individuals with any pharmacist prescription (aOR 1.61, 95% CI 1.44–1.79), compared with those with clinic-based prescriptions (Table 3).<br><br>Among women enrolled in Medicaid, the odds of contraceptive continuation were 59% lower than for those with private insurance (aOR 0.41, 95% CI 0.40–0.42). Age also played a significant role: individuals aged younger than 25 years (aOR 0.92, 95% CI 0.89–0.95) and those aged 25–34 years (aOR 0.82, 95% CI 0.79– 0.84) were significantly less likely at 12 months to continue contraception than individuals aged 35 years or older.<br><br>**NOTE: all text denotes 34.% is from the pharmacist arm. There is a misprint in Table 2.</td><td>784, 785</td></tr>
<tr><td>No. missing participants</td><td colspan="2">Not reported</td><td colspan="2">Not reported</td><td>NA</td></tr>
<tr><td>Reasons missing</td><td colspan="2">Not reported</td><td colspan="2">Not reported</td><td>NA</td></tr>
</table>

| | | |
|---|---|---|
| **Statistical methods used and appropriateness of these** | We created logistic regression models to test the association of prescriber type (clinician only vs any pharmacist prescription) with contraceptive continuation at 12 months and any breaks in contraceptive coverage over 6 months. Model covariates were selected based on the literature.12,21 For both models, we controlled for rurality, payer, and age. For both models, we report odds ratios and 95% CIs. All hypothesis tests were two-sided, and we considered P=0.05 to be statistically significant.<br>Propensity score matching was used to create a comparison group of people receiving prescriptions from a clinician, similar to the group receiving a prescription from a pharmacist…..Results were consistent and we present the main regression models only. | 783 |
| **Notes** | None | |

## Break in contraceptive coverage

| | Description as stated in report/paper | | | | Page number |
|---|---|---|---|---|---|
| **Outcome** | Break in contraceptive coverage | | | | 783 |
| **Time point** *(specify from start or end of intervention)* | At end of intervention, 6 months | | | | 783 |
| **Results** | Intervention | | Comparison | | 785 for results Appendix 2 for sample size |
| | No. with event | Total in group | No. with event | Total in group | |
| | 548 | 890 | 42,182 | 68,192 | |

| | | | |
|---|---|---|---|
| **Any other results reported** | More than half of women experienced a break in contraceptive coverage over 6 months, with no significant difference among pharmacist (61.6%) or clinician (61.9%, P=0.89) prescriber types (Table 2). Although the majority of women in both groups experienced only one break in contraceptive use, nearly 20% of contraceptive users who received a prescription from either a pharmacist or a clinician experienced three or more breaks<br><br>The unadjusted odds ratio for breaks in contraceptive cover was 1.01 (0.88–1.16) (table 3).<br><br>In multivariate analyses, we found no significant association between a break in coverage and pharmacist prescriber type (Table 4; aOR 1.03, 95% CI 0.90–1.18). The odds of at least one contraceptive break were 6% lower among individuals aged younger than 25 years (aOR 0.94, 95% CI 0.90– 0.98) and 14% higher among those with Medicaid insurance (aOR 1.14, 95% CI 1.10–1.18, Table 4). | | 784, 785, 786 |
| **No. missing participants** | Not reported | Not reported | NA |
| **Reasons missing** | Not reported | Not reported | NA |
| **Statistical methods used and appropriateness of these** | We restricted this analytic cohort to women who did not discontinue their contraception within the first 6 months of use (Fig. 1).<br><br>We created logistic regression models to test the association of prescriber type (clinician only vs any pharmacist prescription) with contraceptive continuation at 12 months and any breaks in contraceptive coverage over 6 months. Model covariates were selected based on the literature.12,21 For both models, we controlled for rurality, payer, and age. For both models, we report odds ratios and 95% CIs. All hypothesis tests were two-sided, and we considered P,.05 to be statistically significant.<br><br>Propensity score matching was used to create a comparison group of people receiving prescriptions from a clinician, similar to the group receiving a prescription from a pharmacist…..Results were consistent and we present the main regression models only. | | 783 |
| **Notes** | None | | |

## Conclusions

| | Description as stated in report/paper | Page number |
|---|---|---|
| **Key conclusions of study authors** | Pharmacist prescription of contraception is associated with higher 12-month contraceptive continuation rates and appears comparable with clinic-based care in frequency of breaks in care. | 786 |
| **Notes** | | |

## Romanelli et al. 2015

### Study eligibility

| **Study Characteristics** | **Eligibility criteria** |
|---|---|
| **Title** | Pharmacist-led medication management program within a patient-centered medical home |
| **Author (year)** | Romanelli et al. 2015 |
| **Country** | California, USA |
| **Type of study** | Retrospective, propensity-score-matched cohort study |
| **Participants** | Adults with face-to-face ambulatory care visits and 12 months of EHR records |
| **Types of intervention** | Medical management program (MMP) pharmacist |
| **Types of comparison** | Patient centered medical home physician care (PCMH); usual care (non PCMH primary care) |
| **Types of outcome measures** | Ambulatory care visits; Emergency department visits; Hospitalizations; Blood pressure (at goal); LDL cholesterol (at goal); HbA1C (at goal) |
| **Prescriptive authority** | Collaborative |
| **Include/Exclude** | Include |
| **Notes** | |

**DO NOT PROCEED IF STUDY EXCLUDED FROM REVIEW**

## Characteristics of included studies

### Methods

| | Descriptions as stated in report/paper | | Page number |
|---|---|---|---|
| **Aim of study** | To test the hypothesis that the addition of a clinical pharmacist to a patient-centered medical home (PCMH) team can augment patient care within a PCMH setting, resulting in reduced healthcare resource use and improved disease management as measured by intermediate clinical outcomes. | | 453 |
| **Design** | Retrospective, propensity-score-matched cohort study | | 454 |
| **Unit of allocation** *(by individuals, cluster/ groups or body parts)* | Individual | | 454 |
| **Start-end date** | November 1, 2011 – December 31, 2013 | | 454 |
| **Duration of participation** *(from recruitment to last follow-up/ baseline to last follow-up- group level)* *1. Time of consent until last measurement for each individual.* *2. Baseline to final follow-up for each individual* | EHR activity was required 12 months before the index date (BP/LDL/HBA1C gathered within 12 months before).<br><br>Active medication orders and healthcare resource use were captured/identified 6 months before.<br><br>BP/LDL/HBA1C Up to 4 weeks after index date, follow up values were captured at least 6 weeks after the index date<br><br>**13.5 months**<br><br>Patients in each group were identified in the EHR from face-to-face ambulatory care visits occurring between November 1, 2011, and June 30, 2013 (identification period).<br>Patient data was censored on December 31, 2013 | | 454 |
| **Study duration** *(as above with the exception of interim analyses or other circumstances* | November 1, 2011 – December 31, 2013 | | 454 |
| **Ethical approval needed/obtained for study** | Yes | This study was approved by the Sutter Health institutional review board. | 454 |
| **Notes** | | | |

### Participants

| | **Description** *Include comparative information for each intervention or comparison group if available* | **Page number** |
|---|---|---|

| | | | |
|---|---|---|---|
| **Population description** *(from which study participants are drawn)* | Adults seeking ambulatory care | | 454 |
| **Setting** *(including location and social context)* | PCMH Davis Family Clinic in Davis, California, was the site of a pharmacist-led medication management program (MMP).<br><br>within the PCMH Davis Family Clinic but were not referred to the MMP pharmacist (PCMH cohort)<br><br>received usual care at two non-PCMH primary care clinics in Davis, California (non-Sutter Health facilities)(usual care cohort). | | 454 |
| **Inclusion/exclusion criteria** | Inclusion: Patients (at least 18 years old) with 12 months of EHR activity before their index date and at least two face-to-face ambulatory care visits<br>Exclusion: < 18, not enough appointments (at least 2), appointments that were not face-to-face | | 454 |
| **Method of recruitment of participants** | Patients were referred to the clinical pharmacist at the discretion of a primary care provider or the PCMH staff or at the request of the patient. | | 454 |
| **Informed consent obtained** | No | This study was approved by the Sutter Health institutional review board.<br>No consent is mentioned. Presumably waiver for EHR | 454 |
| **Total no. randomised** *(or total pop. at start of study for NRCTs)* | 1108 individuals | | 455 |
| **Clusters** | None | | NA |
| **Baseline imbalances** | Propensity-score matched cohorts were well balanced on baseline covariates (Table 1). At baseline, similar proportions of patients were at BP and LDL cholesterol goals in the MMP cohort relative to the usual care and PCMH cohorts (Table 2).<br><br>MMP patients were slightly more likely to have diabetes mellitus and had more active medications at baseline relative to PCMH patients and patients receiving usual care.<br>In addition, MMP patients more frequently used healthcare resources prior to the index date than did patients in the other cohorts.<br><br>The proportion of patients at their HbA1c goal at baseline was significantly lower among MMP patients (53%) relative to both PCMH patients (69%, $p = 0.018$ versus the MMP cohort) and patients receiving usual care (74%, $p < 0.001$ versus the MMP cohort). | | 455 |

| | | |
|---|---|---|
| **Withdrawals and exclusions** | NA | NA |
| **Age** | Intervention: 66.79±16.40<br>Comparator (PMCH): 65.03±16.26<br>Comparator (usual care): 64.86±17.84 | 456, Table 1 |
| **Sex (female)** | Intervention: 197 (70.1%)<br>Comparator (PMCH): 279 (68.7%)<br>Comparator (usual care): 297 (70.6%) | 456, Table 1 |
| **Subgroups measure** | Not reported | NA |
| **Subgroups reported** | Not reported | NA |
| **Notes** | | |

## Intervention group

| | Description as stated in report/paper | Page number |
|---|---|---|
| **Group name** | Medical management programme (pharmacist) | 454 |
| **No. randomised/assigned to group** *(specify whether no. people or clusters)* | 281 | 455 |
| **Description** *(include sufficient detail for replication, e.g. content, dose, components)* | pharmacist's intervention included coordination of care (medication reconciliation, provision of drug information, medication counselling), disease management (disease education, monitoring of laboratory test values, referrals), and medication therapy management (refill orders; adjustment of medication therapy including discontinuation, modification, and addition of alternative medications) | 454 |
| **Duration of treatment period** | No set treatment period. | NA |
| **Timing** | Not reported | NA |
| **Co-interventions** | Coordination of care (medication reconciliation, provision of drug information, medication counselling), disease management (disease education, monitoring of laboratory test values, referrals) | 454 |
| **Notes** | | |

## Comparator group: PCMH

| | Description as stated in report/paper | Page number |
|---|---|---|
| **Group name** | Patient-centred medical home | 454 |

| | | |
|---|---|---|
| **No. randomised/assigned to group** *(specify whether no. people or clusters)* | 406 | 455 |
| **Description** *(include sufficient detail for replication, e.g. content, dose, components)* | The PCMH Davis primary care family clinic patients that were not referred to the MMP pharmacist | 454 |
| **Duration of treatment period** | No set treatment period | NA |
| **Timing** | Not reported | NA |
| **Co-interventions** | Not reported | NA |
| **Notes** | | |

### Comparator group: Usual care

| | Description as stated in report/paper | Page number |
|---|---|---|
| **Group name** | Usual care | 454 |
| **No. randomised/assigned to group** *(specify whether no. people or clusters)* | 421 | 455 |
| **Description** *(include sufficient detail for replication, e.g. content, dose, components)* | Two non-PCMH primary care clinics in Davis, California (non-Sutter Health facilities) | 454 |
| **Duration of treatment period** | No set treatment period | NA |
| **Timing** | Not reported | NA |
| **Co-interventions** | Not reported | NA |
| **Notes** | | |

## Outcomes

### Ambulatory care visits

| | Description as stated in report/paper | Page number |
|---|---|---|
| **Outcome name** | Ambulatory care visits | 454 |
| **Time points measured** *(specify whether from start or end of intervention)* | Up to 6 months before the index date | 454 |
| **Time points reported** | Unclear, entire duration | 454 |

| | | | |
|---|---|---|---|
| **Outcome definition** *(with diagnostic criteria if relevant)* | Not reported | | 454 |
| **Unit of measurement** *(if relevant)* | Event data, continuous, visits | | 454 |
| **Scales: upper and lower limits** *(indicate whether high or low score is good)* | 2 visits upwards (2 was minimum for eligibility) | | 454 |
| **Is outcome/tool validated?** | No | | NA |
| **Imputation of missing data** *(e.g. assumptions made for ITT analysis)* | Not reported | | NA |
| **Assumed risk estimate** *(e.g. baseline or population risk noted in Background)* | Not reported | | NA |
| **Power** *(e.g. power & sample size calculation, level of power achieved)* | Not reported | | NA |
| **Notes** | | | |

## Emergency department visits

| | Description as stated in report/paper | | Page number |
|---|---|---|---|
| **Outcome name** | Emergency (ED) visits | | 454 |
| **Time points measured** *(specify whether from start or end of intervention)* | Up to 6 months before the index date | | 454 |
| **Time points reported** | Unclear, entire duration | | 454 |
| **Outcome definition** *(with diagnostic criteria if relevant)* | Not reported | | 454 |
| **Unit of measurement** *(if relevant)* | Event data, continuous, visits | | 454 |
| **Scales: upper and lower limits** *(indicate whether high or low score is good)* | 0 upwards | | 454 |
| **Is outcome/tool validated?** | No | | NA |

| | | |
|---|---|---|
| **Imputation of missing data** *(e.g. assumptions made for ITT analysis)* | Not reported | NA |
| **Assumed risk estimate** *(e.g. baseline or population risk noted in Background)* | Not reported | NA |
| **Power** *(e.g. power & sample size calculation, level of power achieved)* | Not reported | NA |
| **Notes** | | |

## Hospitalisations

| | Description as stated in report/paper | | Page number |
|---|---|---|---|
| **Outcome name** | Hospitalizations | | 454 |
| **Time points measured** *(specify whether from start or end of intervention)* | Up to 6 months before the index date | | 454 |
| **Time points reported** | Unclear, entire duration | | 454 |
| **Outcome definition** *(with diagnostic criteria if relevant)* | Not reported | | 454 |
| **Unit of measurement** *(if relevant)* | Event data, continuous, visits | | 454 |
| **Scales: upper and lower limits** *(indicate whether high or low score is good)* | 0 upwards | | 454 |
| **Is outcome/tool validated?** | No | | NA |
| **Imputation of missing data** *(e.g. assumptions made for ITT analysis)* | Not reported | | NA |
| **Assumed risk estimate** *(e.g. baseline or population risk noted in Background)* | Not reported | | NA |
| **Power** *(e.g. power & sample size calculation, level of power achieved)* | Not reported | | NA |

| | |
|---|---|
| **Notes** | |

## Blood pressure

| | Description as stated in report/paper | | Page number |
|---|---|---|---|
| **Outcome name** | Blood pressure (percentage at goals) | | 454 |
| **Time points measured** *(specify whether from start or end of intervention)* | Baseline was captured within the 12 months before and up to four weeks after the index date; follow-up values were captured at least six weeks after the index date | | 454 |
| **Time points reported** | Unclear, entire duration | | 454 |
| **Outcome definition** *(with diagnostic criteria if relevant)* | Target values for blood pressure was determined by the Joint National Committee on Prevention, Detection, Evaluation, and Treatment of High Blood Pressure guidelines. | | 454 |
| **Unit of measurement** *(if relevant)* | Percentage patients at goal | | 455 |
| **Scales: upper and lower limits** *(indicate whether high or low score is good)* | 1-100 | | NA |
| **Is outcome/tool validated?** | No | | NA |
| **Imputation of missing data** *(e.g. assumptions made for ITT analysis)* | Not reported. Data dropped (see footnotes of n in Table 4) | | 458 |
| **Assumed risk estimate** *(e.g. baseline or population risk noted in Background)* | Not reported | | NA |
| **Power** *(e.g. power & sample size calculation, level of power achieved)* | Not reported | | NA |
| **Notes** | | | |

## LDL cholesterol

| | Description as stated in report/paper | Page number |
|---|---|---|
| **Outcome name** | LDL cholesterol (percentage at goal) | 454 |
| **Time points measured** *(specify whether from start or end of intervention)* | Baseline was captured within the 12 months before and up to four weeks after the index date; follow-up values were captured at least six weeks after the index date | 454 |

| | | | |
|---|---|---|---|
| **Time points reported** | Unclear, entire duration | | 454 |
| **Outcome definition** *(with diagnostic criteria if relevant)* | Target values for LDL cholesterol were determined by the National Cholesterol Education Program ATP III | | 454 |
| **Unit of measurement** *(if relevant)* | Percentage patients at goal | | 455 |
| **Scales: upper and lower limits** *(indicate whether high or low score is good)* | 1-100 | | NA |
| **Is outcome/tool validated?** | No | | NA |
| **Imputation of missing data** *(e.g. assumptions made for ITT analysis)* | Not reported. Data dropped (see footnotes of n in Table 4) | | 458 |
| **Assumed risk estimate** *(e.g. baseline or population risk noted in Background)* | Not reported | | NA |
| **Power** *(e.g. power & sample size calculation, level of power achieved)* | Not reported | | NA |
| **Notes** | | | |

## HbA1C

| | Description as stated in report/paper | | Page number |
|---|---|---|---|
| **Outcome name** | HbA1C (percentage at goals) | | 454 |
| **Time points measured** *(specify whether from start or end of intervention)* | Baseline was captured within the 12 months before and up to four weeks after the index date; follow-up values were captured at least six weeks after the index date | | 454 |
| **Time points reported** | Unclear, entire duration | | 454 |
| **Outcome definition** *(with diagnostic criteria if relevant)* | Target values for HbA1c was determined by the American Diabetes Association | | 454 |
| **Unit of measurement** *(if relevant)* | Percentage patients at goal | | 455 |
| **Scales: upper and lower limits** *(indicate whether high or low score is good)* | 1-100 | | NA |
| **Is outcome/tool validated?** | No | | NA |

| | | |
|---|---|---|
| **Imputation of missing data** *(e.g. assumptions made for ITT analysis)* | Not reported. Data dropped (see footnotes of n in Table 4) | 458 |
| **Assumed risk estimate** *(e.g. baseline or population risk noted in Background)* | Not reported | NA |
| **Power** *(e.g. power & sample size calculation, level of power achieved)* | Not reported | NA |
| **Notes** | | |

## Funding/conflict of interest

| | | |
|---|---|---|
| **Study funding sources** *(including role of funders)* | Funded by a research grant from the ASHP Research and Education Foundation. | 453 |
| **Possible conflicts of interest** *(for study authors)* | The authors have declared no potential conflicts of interest. | 453 |
| **Notes** | | |

## Data and analysis

### Ambulatory care visits

| | Description as stated in report/paper | | | | | | Page number |
|---|---|---|---|---|---|---|---|
| **Outcome** | Ambulatory care visits | | | | | | 454 |
| **Time point** *(specify from start or end of intervention)* | At end of study (censor date 31 December 2013) | | | | | | 454 |
| **Results** | Intervention (MMP) | | | Comparison (PCMH) | | | 457 Table 3 |
| | Estimated no. events per 100 | 95% CI | No. participants | Estimated no. events per 100 | 95% CI | No. participants | |
| | 680.7 | 642.2-737.1 | 281 | 573.8 | 527.2-620.3 | 406 | |
| | Intervention (MP) | | | Comparison (Usual care) | | | 457 Table 3 |
| | Estimated no. events per 100 | 95% CI | No. participants | Estimated no. events per 100 | 95% CI | No. participants | |

| | | | | | | | |
|---|---|---|---|---|---|---|---|
| | 680.7 | 642.2-737.1 | 281 | 651.5 | 593.7-709.2 | 421 | |

| | | | |
|---|---|---|---|
| **Any other results reported** *(e.g. mean difference, CI, P value)* | The MMP cohort had a significantly higher estimated rate of ambulatory care visits compared with PCMH group (IRR, 1.19; 95% CI, 1.06–1.33; *p* = 0.004)<br><br>The MMP group had a similar rate of ambulatory care visits (IRR, 0.40; 95% CI, 0.25–0.63; *p* <0.001) compared with the usual care group.<br><br>No significant differences in rates of ambulatory care visits, ED visits, or hospitalizations were observed between the PCMH and usual care cohorts. | | 455 |
| **No. missing participants** | Intervention: 0 | PMCH: 0<br>Usual care: 0 | 455, 457 |
| **Statistical methods used and appropriateness of these** *(e.g. adjustment for correlation)* | Statistical analyses. For the primary outcome measure of differences in rates of ambulatory care visits, ED visits, and hospitalizations through the end date, we fit negative binomial regression models for each outcome with statistical adjustment for propensity-score covariates. Additional statistical adjustment was performed for peripheral vascular disease and renal disease, as the proportion of patients with these diagnoses differed after matching (data not shown) and was considered to have the potential to influence outcomes. Estimated number of events per 100 persons and incident rate ratios (IRRs) with 95% confidence intervals (CIs) were calculated.<br><br>In all statistical models, robust standard errors were calculated to account for within-pair correlation. Because multiple comparisons were performed for each endpoint, a Bonferroni-corrected critical value of 0.017 (i.e., 0.05 divided by 3) was used to reduce the chance of a Type I error. Accordingly, the a priori level of significance was 0.017 for all tests of hypotheses. | | 455, 457 |
| **Notes** | | | |

## Emergency department (ED) visits

| | Description as stated in report/paper | | Page number |
|---|---|---|---|
| **Outcome** | ED visits | | 454 |
| **Time point** *(specify from start or end of intervention)* | At end of study (censor date 31 December 2013) | | 454 |
| **Results** | Intervention (MMP) | Comparison (PCMH) | 457 Table 3 |

| | | | | | | | |
|---|---|---|---|---|---|---|---|
| | Estimated no. events per 100 | 95% CI | No. participants | Estimated no. events per 100 | 95% CI | No. participants | |
| | 28.9 | (22.2 – 35.6) | 281 | 36.5 | (29.0 – 44.1) | 406 | |
| | Intervention (MP) | | | Comparison (Usual care) | | | 457 Table 3 |
| | Estimated no. events per 100 | 95% CI | No. participants | Estimated no. events per 100 | 95% CI | No. participants | |
| | 28.9 | (22.2 – 35.6) | 281 | 41.2 | (33.5 – 48.9) | 421 | |
| **Any other results reported** *(e.g. mean difference, CI, P value)* | The MMP cohort had a lower estimated rate of ED visits compared with the PCMH cohort, but this difference was not significant (IRR, 0.79; 95% CI, 0.79 (0.58-1.07), p=0.124)<br><br>The MMP group had significantly lower rates of ED visits (IRR, 0.70; 95% CI, 0.53–0.93; p = 0.014) | | | | | | 455 |
| **No. missing participants** | Intervention: 0 | | | PMCH: 0<br>Usual care: 0 | | | 455, 457 |
| **Statistical methods used and appropriateness of these** *(e.g. adjustment for correlation)* | Statistical analyses. For the primary outcome measure of differences in rates of ambulatory care visits, ED visits, and hospitalizations through the end date, we fit negative binomial regression models for each outcome with statistical adjustment for propensity-score covariates. Additional statistical adjustment was performed for peripheral vascular disease and renal disease, as the proportion of patients with these diagnoses differed after matching (data not shown) and was considered to have the potential to influence outcomes. Estimated number of events per 100 persons and incident rate ratios (IRRs) with 95% confidence intervals (CIs) were calculated.<br><br>In all statistical models, robust standard errors were calculated to account for within-pair correlation. Because multiple comparisons were performed for each endpoint, a Bonferroni-corrected critical value of 0.017 (i.e., 0.05 divided by 3) was used to reduce the chance of a Type I error. Accordingly, the a priori level of significance was 0.017 for all tests of hypotheses. | | | | | | 455, 457 |
| **Notes** | | | | | | | |

## Hospitalisations

<table>
<tr><td></td><td colspan="6">Description as stated in report/paper</td><td>Page number</td></tr>
<tr><td>Outcome</td><td colspan="6">Hospitalisations</td><td>454</td></tr>
<tr><td>Time point (specify from start or end of intervention)</td><td colspan="6">At end of study (censor date 31 December 2013)</td><td>454</td></tr>
<tr><td rowspan="2">Results</td><td colspan="3">Intervention (MMP)</td><td colspan="3">Comparison (PCMH)</td><td rowspan="2">457 Table 3</td></tr>
<tr><td>Estimated no. events per 100</td><td>95% CI</td><td>No. participants</td><td>Estimated no. events per 100</td><td>95% CI</td><td>No. participants</td></tr>
<tr><td></td><td>7.9</td><td>(4.7 – 11.1)</td><td>281</td><td>16.4</td><td>(11.1 – 21.6)</td><td>406</td><td></td></tr>
<tr><td rowspan="2"></td><td colspan="3">Intervention (MP)</td><td colspan="3">Comparison (Usual care)</td><td rowspan="2">457 Table 3</td></tr>
<tr><td>Estimated no. events per 100</td><td>95% CI</td><td>No. participants</td><td>Estimated no. events per 100</td><td>95% CI</td><td>No. participants</td></tr>
<tr><td></td><td>7.9</td><td>(4.7 – 11.1)</td><td>281</td><td>19.9</td><td>(14.4 – 25.3)</td><td>421</td><td></td></tr>
<tr><td>Any other results reported (e.g. mean difference, CI, P value)</td><td colspan="6">The MMP cohort had a lower rate of hospitalizations (IRR, 0.48, 95% CI, 0.30–0.78; p = 0.003) relative to the PCMH group (Table 3).<br><br>The MMP cohort had significantly lower rates of hospitalizations compared with usual care (IRR, 0.40, 95% CI, 0.25-0.63, p<0.001)<br><br>No significant differences were observed between the PMCH and usual care cohorts in rates of hospitalizations.</td><td>455</td></tr>
<tr><td>No. missing participants</td><td colspan="3">Intervention: 0</td><td colspan="3">PMCH: 0<br>Usual care: 0</td><td>455, 457</td></tr>
</table>

| | | |
|---|---|---|
| **Statistical methods used and appropriateness of these** *(e.g. adjustment for correlation)* | Statistical analyses. For the primary outcome measure of differences in rates of ambulatory care visits, ED visits, and hospitalizations through the end date, we fit negative binomial regression models for each outcome with statistical adjustment for propensity-score covariates. Additional statistical adjustment was performed for peripheral vascular disease and renal disease, as the proportion of patients with these diagnoses differed after matching (data not shown) and was considered to have the potential to influence outcomes. Estimated number of events per 100 persons and incident rate ratios (IRRs) with 95% confidence intervals (CIs) were calculated.<br><br>In all statistical models, robust standard errors were calculated to account for within-pair correlation. Because multiple comparisons were performed for each endpoint, a Bonferroni-corrected critical value of 0.017 (i.e., 0.05 divided by 3) was used to reduce the chance of a Type I error. Accordingly, the a priori level of significance was 0.017 for all tests of hypotheses. | 455, 457 |
| **Notes** | | |

## Blood pressure

| | Description as stated in report/paper | | | | | | Page number |
|---|---|---|---|---|---|---|---|
| **Outcome** | Blood pressure goal | | | | | | 454 |
| **Time point** *(specify from start or end of intervention)* | within the 12 months before and up to four weeks after the index date; follow-up: at least 6 weeks after index date; at end of study (censor date 31 December 2013) | | | | | | 454 |
| **Results** | Intervention (MMP) | | | Comparison (PCMH) | | | 458 Table 4 |
| | % patients at goal | 95% CI | No. participants | % patients at goal | 95% CI | No. participants | |
| | 74.0 | (67.9 – 79.2) | 263 | 70.6 | (65.2 – 75.5) | 342 | |
| | Intervention (MP) | | | Comparison (Usual care) | | | 458 Table 4 |
| | % patients at goal | 95% CI | No. participants | % patients at goal | 95% CI | No. participants | |
| | 74.0 | (67.9 – 79.2) | 263 | 66.2 | (60.5 – 71.4) | 351 | |

| | | | |
|---|---|---|---|
| **Any other results reported** *(e.g. mean difference, CI, P value)* | MMP vs. PCMH<br>aOR, 95% CI: 1.16 (0.79-1.71), p=0.454<br><br>MMP vs usual care<br>aOR, 95% CI: 1.47 (1.00-2.16), p=0.052<br><br>Intermediate clinical outcomes also did not significantly differ between these two groups (Table 4). [PMCH and usual care] 1.26 (0.88–1.81), *p* = 0.202 | | 455, 458 (table 4) |
| **No. missing participants** | Intervention: 18 | PMCH: 64<br>Usual care: 70 | 458 |
| **Statistical methods used and appropriateness of these** *(e.g. adjustment for correlation)* | For the secondary outcome measures of differences in intermediate clinical outcomes (percentage of patients at BP, LDL cholesterol, and HbA1c goals) through the censor date, A1c we fit logistic regression models with statistical adjustment for baseline propensity-score covariates, whether baseline values were at goal (yes or no), and use of appropriate medication at baseline (i.e., antihypertensive, lipid- lowering, or antihyperglycemic agents). Adjusted percentages of patients at goal and adjusted odds ratios with corresponding 95% Cis were generated.<br><br>In all statistical models, robust standard errors were calculated to account for within-pair correlation. Because multiple comparisons were performed for each endpoint, a Bonferroni-corrected critical value of 0.017 (i.e., 0.05 divided by 3) was used to reduce the chance of a Type I error. Accordingly, the a priori level of significance was 0.017 for all tests of hypotheses | | 455, 457 |
| **Notes** | | | |

## LDL cholesterol

| | Description as stated in report/paper | | | | | | Page number |
|---|---|---|---|---|---|---|---|
| **Outcome** | LDL cholesterol | | | | | | 454 |
| **Time point** *(specify from start or end of intervention)* | within the 12 months before and up to four weeks after the index date; follow-up: at least 6 weeks after index date; at end of study (censor date 31 December 2013) | | | | | | 454 |
| **Results** | Intervention (MMP) | | | Comparison (PCMH) | | | 458 Table 4 |
| | % patients at goal | 95% CI | No. participants | % patients at goal | 95% CI | No. participants | |
| | 76.1 | (67.2 – 83.1) | 155 | 69.6 | (60.5 – 77.3) | 159 | |
| | Intervention (MP) | | | Comparison (Usual care) | | | 458 Table 4 |

| | | | | | | | |
|---|---|---|---|---|---|---|---|
| | % patients at goal | 95% CI | No. participants | % patients at goal | 95% CI | No. participants | |
| | 76.1 | (67.2 – 83.1) | 155 | 78.8 | (70.9 – 84.5) | 170 | |
| **Any other results reported** *(e.g. mean difference, CI, P value)* | MMP vs. PCMH<br>aOR, 95% CI: 1.32 (0.70-2.46), p=0.390<br><br>MMP vs usual care<br>aOR, 95% CI: 0.83 (0.45-1.54), p=0.565<br><br>Intermediate clinical outcomes also did not significantly differ between these two groups (Table 4). [PMCH and usual care] 0.63 (0.36–1.10), *p* = 0.108 | | | | | | 455, 458 (table 4) |
| **No. missing participants** | Intervention: 126 | | | PMCH: 247<br>Usual care: 251 | | | 458 |
| **Statistical methods used and appropriateness of these** *(e.g. adjustment for correlation)* | For the secondary outcome measures of differences in intermediate clinical outcomes (percentage of patients at BP, LDL cholesterol, and HbA1c goals) through the censor date, A1c we fit logistic regression models with statistical adjustment for baseline propensity-score covariates, whether baseline values were at goal (yes or no), and use of appropriate medication at baseline (i.e., antihypertensive, lipid- lowering, or antihyperglycemic agents). Adjusted percentages of patients at goal and adjusted odds ratios with corresponding 95% Cis were generated.<br><br>In all statistical models, robust standard errors were calculated to account for within-pair correlation. Because multiple comparisons were performed for each endpoint, a Bonferroni-corrected critical value of 0.017 (i.e., 0.05 divided by 3) was used to reduce the chance of a Type I error. Accordingly, the a priori level of significance was 0.017 for all tests of hypotheses | | | | | | 455, 457 |
| **Notes** | | | | | | | |

## HbA1C (at goal)

| | Description as stated in report/paper | | Page number |
|---|---|---|---|
| **Outcome** | HbA1C | | 454 |
| **Time point** *(specify from start or end of intervention)* | within the 12 months before and up to four weeks after the index date; follow-up: at least 6 weeks after index date; at end of study (censor date 31 December 2013) | | 454 |
| **Results** | Intervention (MMP) | Comparison (PCMH) | 458 Table 4 |

| | | | | | | | |
|---|---|---|---|---|---|---|---|
| | % patients at goal | 95% CI | No. participants | % patients at goal | 95% CI | No. participants | |
| | 59.1 | (46.3 – 70.8) | 110 | 62.5 | (49.4 - 73.9) | 105 | |
| | Intervention (MP) | | | Comparison (Usual care) | | | 458 Table 4 |
| | % patients at goal | 95% CI | No. participants | % patients at goal | 95% CI | No. participants | |
| | 59.1 | (46.3 – 70.8) | 110 | 70.9 | (59.1 - 80.4) | 125 | |
| **Any other results reported** *(e.g. mean difference, CI, P value)* | MMP vs. PCMH<br>aOR, 95% CI: 0.61 (0.25-1.49), p=0.278<br><br>MMP vs usual care<br>aOR, 95% CI: 0.44 (0.18-1.08), p=0.074<br><br>Intermediate clinical outcomes also did not significantly differ between these two groups (Table 4). [PMCH and usual care] 0.73 (0.31–1.68), *p* = 0.455 | | | | | | 455, 458 (table 4) |
| **No. missing participants** | Intervention: 171 | | | PMCH: 301<br>Usual care: 296 | | | 458 |
| **Statistical methods used and appropriateness of these** *(e.g. adjustment for correlation)* | For the secondary outcome measures of differences in intermediate clinical outcomes (percentage of patients at BP, LDL cholesterol, and HbA1c goals) through the censor date, A1c we fit logistic regression models with statistical adjustment for baseline propensity-score covariates, whether baseline values were at goal (yes or no), and use of appropriate medication at baseline (i.e., antihypertensive, lipid- lowering, or antihyperglycemic agents). Adjusted percentages of patients at goal and adjusted odds ratios with corresponding 95% Cis were generated.<br><br>In all statistical models, robust standard errors were calculated to account for within-pair correlation. Because multiple comparisons were performed for each endpoint, a Bonferroni-corrected critical value of 0.017 (i.e., 0.05 divided by 3) was used to reduce the chance of a Type I error. Accordingly, the a priori level of significance was 0.017 for all tests of hypotheses | | | | | | 455, 457 |
| **Notes** | | | | | | | |

## Conclusions

| | **Description as stated in report/paper** | **Page number** |
|---|---|---|

| | | |
|---|---|---|
| **Key conclusions of study authors** | Patients in a pharmacist-led MMP had a significantly higher estimated rate of ambulatory care visits but a lower rate of hospitalizations than did patients who attended the same clinic but were not in the MMP.<br><br>MMP patients had a similar rate of ambulatory care visits but significantly lower rates of hospitalizations and ED visits than patients receiving usual care.<br><br>Intermediate clinical outcomes at follow-up did not differ across groups | 456, 459 |
| **Notes** | | |

## Scullin et al. 2007

### Study eligibility

| **Study Characteristics** | **Eligibility criteria** |
|---|---|
| **Title** | An innovative approach to integrated medicines management |
| **Author (year)** | Scullin et al. 2007 |
| **Country** | Northern Ireland, UK |
| **Type of study** | Randomised controlled trial (quality improvement project) |
| **Participants** | Patients at risk of drug-related problems |
| **Types of intervention** | Integrated medicines management |
| **Types of comparison** | Normal care (physician prescribing) |
| **Types of outcome measures** | Length of hospital stay; frequency of hospital readmission; number of hospital readmissions; 12 month mortality |
| **Prescriptive authority** | Protocol |
| **Include/Exclude** | Include |
| **Notes** | |

**DO NOT PROCEED IF STUDY EXCLUDED FROM REVIEW**

## Characteristics of included studies

### Methods

| | Descriptions as stated in report/paper | | Page number |
|---|---|---|---|
| **Aim of study** | To determine whether an increased input by clinical pharmacists at each stage of the patient's hospital journey, from admission through discharge, resulted in an enhanced level of patient care as measured by a number of clinical and economic outcomes. | | 781 |
| **Design** | Randomised controlled trial | | 783 |
| **Unit of allocation** *(by individuals, cluster/ groups or body parts)* | Individuals | | 783 |
| **Start-end date** | Not reported | | NA |
| **Duration of participation** *(from recruitment to last follow-up/ baseline to last follow-up- group level)* *1. Time of consent until last measurement for each individual.* *2. Baseline to final follow-up for each individual* | No set treatment period. Treatment continued until discharge. | | 784 |
| **Study duration** *(as above with the exception of interim analyses or other circumstances* | 1.5 years | | 784 |
| **Ethical approval needed/obtained for study** | No | Not reported | NA |
| **Notes** | | | |

### Participants

| | Description<br>*Include comparative information for each intervention or comparison group if available* | Page number |
|---|---|---|

| | | |
|---|---|---|
| **Population description** *(from which study participants are drawn)* | Patients presenting to wards within three general hospital sites of the United Hospitals Trust (Antrim Area Hospital, Mid-Ulster hospital, and White-abbey hospital) | 782 |
| **Setting** *(including location and social context)* | Three general hospital sites of the United Hospitals Trust (Antrim Area Hospital, Mid-Ulster hospital, and White-abbey hospital) | 782 |
| **Inclusion/exclusion criteria** | Inclusion: any one of the following criteria: taking at least 4 regular medications; taking a high risk drug(s); taking antidepressants and were 65 years of age or older; had a previous hospital admission within the last 6 months. Patients prescribed intravenous antibiotics on day 1 of their admission were also eligible.<br>Exclusion: patients with scheduled admissions; patients admitted from private nursing homes | 783 |
| **Method of recruitment of participants** | Patients meeting the eligibility criteria were randomly assigned to the IMM group or normal care group, using block randomization coupled with a closed envelope technique [26]. Randomization was carried out in blocks of 20 (each block contained 10 IMM and 10 normal care allocations). | 783 |
| **Informed consent obtained** | No | |
| **Total no. randomised** *(or total pop. at start of study for NRCTs)* | 762 individuals | 784 |
| **Clusters** | No | 784 |
| **Baseline imbalances** | There was no significant difference between the two groups with respect to gender (P=0.259). The average age (±SD) of the population who received normal care was 69.9±14.8, compared with an average age of 70.3±13.8 for the IMM population (P=0.732). The majority of patients enrolled in both groups were, therefore retired from work (78.0% normal care; 74.4% IMM). Other demographic statistics included alcohol status (33.2% of the normal care population drank alcohol; 32.6% IMM), smoking status (21.5% of the normal care population current smokers, 20.0% ex-smokers; 18.6% of IMM population current smokers, 17.5% ex-smokers). … Analysis of this information showed a comparable pattern between the two populations with 46.0% of the normal care group having visited their GP compared with 48.8% of the IMM group, 34.8% of normal care patients had been to see a hospital based consultant compared with 33.7% of IMM patients, and 4.1% of normal care patients had been to see an 'other' health care professional compared with 3.1% of IMM patients in the previous 6 months. | 784 |

| | | |
|---|---|---|
| **Withdrawals and exclusions** | Unclear | 784 |
| **Age** | Intervention: 70.3±13.8<br>Comparator: 69.9±14.8 | 784 |
| **Sex (female)** | Intervention: 204 (55%)<br>Comparator: 199 (51%) | 784 |
| **Subgroups measure** | Not reported | NA |
| **Subgroups reported** | Not reported | NA |
| **Notes** | | |

## Intervention group

| | Description as stated in report/paper | Page number |
|---|---|---|
| **Group name** | Integrated medicines management group | 783 |
| **No. randomised/assigned to group** *(specify whether no. people or clusters)* | 391 | 784 |
| **Description** *(include sufficient detail for replication, e.g. content, dose, components)* | Patients received an intensive clinical pharmacy service throughout their hospital stay. Drug treatment was reviewed daily, taking into account therapeutic goals, relevant clinical chemistry and haematology results, and, where appropriate, therapeutic drug monitoring…Counselling, tailored to suit the needs of each individual patient, was provided by the clinical pharmacists…Project technicians implemented an enhanced management of stock on the wards which included: maintenance of stock levels (unit dose dispensing is not used within UK hospitals), daily kardex and drug trolley reviews to manage non-routine stock and transfer of medicines for patients moving between wards… At discharge, the IMM pharmacist generated and authorized a discharge prescription according to protocols agreed by the Trust's Drug and Therapeutics Committee | 783,784 |
| **Duration of treatment period** | No set treatment period. Treatment continued until discharge. | 783,784 |
| **Timing** | Unclear (only available from 8:30 – 5 Monday through Friday) | 784 |
| **Co-interventions** | Medication review; drug monitoring; counselling; enhanced management of stock | 783,784 |
| **Notes** | | |

## Comparator group

| | Description as stated in report/paper | Page number |
|---|---|---|
| **Group name** | Normal care group | 783 |
| **No. randomised/assigned to group** *(specify whether no. people or clusters)* | 371 | 784 |
| **Description** *(include sufficient detail for replication, e.g. content, dose, components)* | Usual care | 782 |
| **Duration of treatment period** | No set treatment period. Treatment continued until discharge. | 783,784 |
| **Timing** | Not reported | NA |
| **Co-interventions** | Not reported | NA |
| **Notes** | | |

## Outcomes

### Length of hospital stay

| | Description as stated in report/paper | | Page number |
|---|---|---|---|
| **Outcome name** | Length of hospital stay | | 784 |
| **Time points measured** *(specify whether from start or end of intervention)* | At end of intervention | | 785 |
| **Time points reported** | At end of intervention | | 785 |
| **Outcome definition** *(with diagnostic criteria if relevant)* | Length of hospital stay | | 785 |
| **Unit of measurement** *(if relevant)* | days | | 785 |
| **Scales: upper and lower limits** *(indicate whether high or low score is good)* | Not reported (1 upwards) | | NA |
| **Is outcome/tool validated?** | No | | NA |
| **Imputation of missing data** *(e.g. assumptions made for ITT analysis)* | Not reported | | NA |

| | | |
|---|---|---|
| **Assumed risk estimate** *(e.g. baseline or population risk noted in Background)* | Not reported | NA |
| **Power** *(e.g. power & sample size calculation, level of power achieved)* | Not reported | NA |
| **Notes** | | |

## Hospital readmission

| | Description as stated in report/paper | | Page number |
|---|---|---|---|
| **Outcome name** | Hospital readmission | | 784 |
| **Time points measured** *(specify whether from start or end of intervention)* | 12 months post discharge | | 786 |
| **Time points reported** | 12 months post discharge | | 786 |
| **Outcome definition** *(with diagnostic criteria if relevant)* | Frequency of readmission within 12 months post discharge<br>Number of readmissions within 12 months post discharge<br><br>over a 12-month follow-up period, readmission data for the two groups were collected from the hospital computer system and included assessment of the time to a further hospital admission as well as the number of readmissions. | | 784, 786 |
| **Unit of measurement** *(if relevant)* | Binary, yes/no<br>Continuous, whole counts | | 786 |
| **Scales: upper and lower limits** *(indicate whether high or low score is good)* | 1-11, Table 5 | | 786 |
| **Is outcome/tool validated?** | No | | NA |
| **Imputation of missing data** *(e.g. assumptions made for ITT analysis)* | No imputation reported (data were unavailable for 8 patients, 7 normal care, 1 IMM) | | 786 |
| **Assumed risk estimate** *(e.g. baseline or population risk noted in Background)* | Not reported | | NA |
| **Power** *(e.g. power & sample size calculation, level of power achieved)* | Not reported | | NA |

| | |
|---|---|
| **Notes** | |

## 12-month mortality

| | Description as stated in report/paper | | Page number |
|---|---|---|---|
| **Outcome name** | 12-month mortality | | 786 |
| **Time points measured** *(specify whether from start or end of intervention)* | 12 months | | 786 |
| **Time points reported** | 12 month | | 786 |
| **Outcome definition** *(with diagnostic criteria if relevant)* | 12 month mortality | | 786 |
| **Unit of measurement** *(if relevant)* | Binary, dead/alive | | 786 |
| **Scales: upper and lower limits** *(indicate whether high or low score is good)* | Not reported | | NA |
| **Is outcome/tool validated?** | No | | NA |
| **Imputation of missing data** *(e.g. assumptions made for ITT analysis)* | Not reported | | NA |
| **Assumed risk estimate** *(e.g. baseline or population risk noted in Background)* | Not reported | | NA |
| **Power** *(e.g. power & sample size calculation, level of power achieved)* | Not reported | | NA |
| **Notes** | | | |

## Funding/conflict of interest

| | | |
|---|---|---|
| **Study funding sources** *(including role of funders)* | Funding for this project was obtained from the Department of Health, Social Services and Public Safety (Northern Ireland) under its Executive Programme Fund scheme. | 787 |
| **Possible conflicts of interest** *(for study authors)* | Not reported | NA |
| **Notes** | | |

## Data and analysis

### Length of hospital stay

| | Description as stated in report/paper | | | | | | Page number |
|---|---|---|---|---|---|---|---|
| **Outcome** | Length of hospital stay | | | | | | 785 |
| **Time point** *(specify from start or end of intervention)* | End of intervention | | | | | | 785 |
| **Results** | Intervention | | | Comparison | | | 785 |
| | Mean | 95% CI | No. participants | Mean | 95% CI | No. participants | |
| | 7.8 | (7.1, 8.6) | 371 | 9.8 | (8.8, 10.9) | 391 | |
| **Any other results reported** *(e.g. mean difference, CI, P value)* | The (geometric) mean=LOS was reduced by 2.0 days (P=0.003; independent samples t-test log) for IMM patients when compared with normal care patients. The mean length of stay (LOS) was reduced from 9.8 days with 95% confidence intervals of 8.8 and 10.9 for normal care patients to 7.8 days with 95% confidence intervals of 7.1 and 8.6 for IMM patients. | | | | | | 785 |
| **No. missing participants** | Not reported | | | | | | NA |
| **Statistical methods used and appropriateness of these** *(e.g. adjustment for correlation)* | Length of hospital stay was compared using the logarithmic independent samples t-test (log e),<br><br>A log transformation was performed on the length of hospital stay data sets owing to their skewed nature. The two samples were tested using the natural logarithm of LOS. | | | | | | 784 |
| **Notes** | | | | | | | |

### Hospital readmission

| | Description as stated in report/paper | | | | Page number |
|---|---|---|---|---|---|
| **Outcome** | Frequency of hospital readmission | | | | 786 |
| **Time point** *(specify from start or end of intervention)* | 12 months post discharge | | | | 786 |
| **Results** | Intervention | | Comparison | | 786 |
| | No. with event | Total in group | No. with event | Total in group | |
| | 141 | 370 | 172 | 383 | |

| | | | |
|---|---|---|---|
| **Any other results reported** | The number of readmissions to hospital was shown to be significantly different between the two groups using Fisher's exact test (P=0.027) with 59.2% of the IMM group not being readmitted within 12 months compared with 50.7% of the normal care population.<br>The average number of readmissions to hospital within 12 months for normal care patients was 1.0 compared with 0.8 for the IMM patients. | | 786 |
| **No. missing participants** | 1 | 7 | 786 |
| **Reasons missing** | Not reported | Not reported | NA |
| **Statistical methods used and appropriateness of these** | In addition the chi square test (or Fisher's exact test) was used to compare the frequency of readmissions over the 12-month follow-up period <between the IMM and normal care groups. A P -value of <0.05 was considered statistically significant in all cases. | | 784 |
| **Notes** | Discrepancy in findings reported in-text and in table 5. Final findings extracted from table 5. | | |

## 12-month mortality

| | **Description as stated in report/paper** | | | | **Page number** |
|---|---|---|---|---|---|
| **Outcome** | 12-month mortality | | | | 786 |
| **Time point** *(specify from start or end of intervention)* | 12 months post discharge | | | | 786 |
| **Results** | Intervention | | Comparison | | 786 |
| | No. with event | Total in group | No. with event | Total in group | |
| | 67 | 371 | 76 | 391 | |
| **Any other results reported** | At the 12 month follow-up the mortality status of the two populations were also analysed. Over the course of the project 76 (19.79%) normal care patients and 67 (18.11%) IMM patients died either at first admission to hospital, while re-admitted to hospital or while being cared for within primary care (P=0.578). | | | | 786 |
| **No. missing participants** | Not reported | | | | NA |
| **Reasons missing** | Not reported | | | | NA |
| **Statistical methods used and appropriateness of these** | Not reported | | | | NA |
| **Notes** | None | | | | |

## Conclusions

| | **Description as stated in report/paper** | **Page number** |
|---|---|---|

| | | |
|---|---|---|
| **Key conclusions of study authors** | The length of hospital stay was significantly reduced for those patients who received the new service<br><br>In addition to a reduced length of hospital stay, the IMM service also resulted in a reduction in the number of readmissions to hospital as well as a longer time to readmission.<br><br>The IMM service could act as a service template, and has the potential to be replicated within other hospital trusts within the UK. | 787 |
| **Notes** | | |

## Thompson 1984

### Study eligibility

| **Study Characteristics** | **Eligibility criteria** |
|---|---|
| **Title** | Clinical Pharmacists Prescribing Drug Therapy in a Geriatric Setting: Outcome of a Trial |
| **Author (year)** | Thompson (1984) |
| **Country** | Not specified, USA |
| **Type of study** | Non-randomised controlled trial |
| **Participants** | Adults in a skilled nursing facility |
| **Types of intervention** | Pharmacist prescribing (including continuing, adjusting, discontinuing, or changing drug class), medication review, ordering of tests, and physical assessment |
| **Types of comparison** | Physician prescribing |
| **Types of outcome measures** | Average number of drugs per patient; hospitalizations; deaths; discharge to lower levels of care |
| **Prescriptive authority** | By formulary |
| **Include/Exclude** | Include |
| **Notes** | |

**DO NOT PROCEED IF STUDY EXCLUDED FROM REVIEW**

## Characteristics of included studies

### Methods

| | Descriptions as stated in report/paper | | Page number |
|---|---|---|---|
| **Aim of study** | The study's objective was to determine whether direct intervention, through drug therapy prescribing and patient care management, could improve the quality of patient care | | 154 |
| **Design** | Non-randomised controlled trial 'quasi-experimental, pretest-post-test control group design' | | 154 |
| **Unit of allocation** *(by individuals, cluster/ groups or body parts)* | Individual | | 155 |
| **Start-end date** | February 1, 1981 – January 31, 1982 | | 155 |
| **Duration of participation** *(from recruitment to last follow-up/ baseline to last follow-up- group level)* *1. Time of consent until last measurement for each individual.* *2. Baseline to final follow-up for each individual* | 12 months | | 155 |
| **Study duration** *(as above with the exception of interim analyses or other circumstances* | 2 years<br><br>Pretreatment year: February 1, 1980 - January 30, 1981.<br>Study year: February 1, 1981 – January 31, 1982 | | 155 |
| **Ethical approval needed/obtained for study** | Yes | All patients in the study facility (n = 152) gave informed consent to participate in the study | 155 |
| **Notes** | | | |

### Participants

| | **Description** *Include comparative information for each intervention or comparison group if available* | **Page number** |
|---|---|---|

| | | | |
|---|---|---|---|
| **Population description** *(from which study participants are drawn)* | Adults in a skilled nursing facility | | 155, 156 Table 1 |
| **Setting** *(including location and social context)* | One skilled nursing facility in the Los Angeles area | | 155 |
| **Inclusion/exclusion criteria** | Inclusion: resident of a skilled nursing facility<br><br>Exclusion: length of stay less than 2 months | | 155 |
| **Method of recruitment of participants** | Unclear. Consented within inpatient nursing facility. | | 155 |
| **Informed consent obtained** | Yes | All patients in the study facility (n = 152) gave informed consent to participate in the study-19 | 155 |
| **Total no. randomised** *(or total pop. at start of study for NRCTs)* | 152. 70 in experimental group and 82 in control group. | | 155 |
| **Clusters** | None | | 156 |
| **Baseline imbalances** | Experimental and control group patients in the baseline year were analyzed for potential differences in several variables. As depicted in Table 1, there were no significant differences in patient age, sex, or length of stay. | | 156 |
| **Withdrawals and exclusions** | All patients who length of stay was less than two months were excluded from the analysis…This short-stay group numbered 19 of the original and study year patients who gave informed consent to participate in the study.<br>[but 139 were analyzed for the study year, not 133 which is 152 consented minus the 19 short-stays] | | 156, Tables 1-3 |
| **Age** | Intervention: 85.1(+/- 10.5 SD)<br>Comparator: 86.3 (+/- 8.1 SD) | | 156, Table 1 |
| **Sex (female)** | Intervention: 55 (82%)<br>Comparator: 61 (85%) | | 156, Table 1 |
| **Subgroups measure** | None reported | | |
| **Subgroups reported** | Not reported | | |
| **Notes** | | | |

## Intervention group

| | Description as stated in report/paper | Page number |
|---|---|---|
| **Group name** | Pharmacist prescribing | 155 |
| **No. randomised/assigned to group** *(specify whether no. people or clusters)* | 70 | 155 |

| | | |
|---|---|---|
| **Description** *(include sufficient detail for replication, e.g. content, dose, components)* | Following the baseline observation period, the clinical pharmacists began prescribing drug therapy and monitoring the experimental patient group. Prescribing and monitoring activities by the clinical pharmacists began February 1, 1981, and continued through January 31, 1982…<br>The conditions of all patients in the treatment group were evaluated by the clinical pharmacists, who reviewed the patients' medical records and noted all past and present medications and physicians' diagnoses. Each patient's medical, social, functional, and drug history was reviewed. Physical assessment was performed to update and verify the past data base and physical findings. Appropriate laboratory tests were then ordered and physical assessment parameters determined in each case to help further confirm or strengthen the data base.<br>On the basis of this information, the patient's medications were reviewed with the options of continuing the present medications, making dose adjustments, or entirely discontinuing and changing the type or class of medication. All patients were then followed up by periodic physical assessment and laboratory evaluation. | 155 |
| **Duration of treatment period** | 12 months | 155 |
| **Timing** | All patients were seen and examined monthly; if necessary, they were seen more frequently. | 155 |
| **Co-interventions** | Medication review, ordering of tests, and physical assessment | 155 |
| **Notes** | | |

## Comparator group

| | Description as stated in report/paper | Page number |
|---|---|---|
| **Group name** | Physician prescribing | 155 |
| **No. randomised/assigned to group** *(specify whether no. people or clusters)* | 82 | 155 |
| **Description** *(include sufficient detail for replication, e.g. content, dose, components)* | The control group consisted of 82 patients cared for by a private community physician, a board-certified internist.<br>During the pretreatment year, from February 1, 1980, through January 30, 1981, baseline observations were made on the experimental and control groups. | 155 |

| | | |
|---|---|---|
| **Duration of treatment period** | 12 months | 155 |
| **Timing** | None reported | NA |
| **Co-interventions** | None reported | NA |
| **Notes** | | |

## Outcomes

### Average number of drugs per patient

| | Description as stated in report/paper | | Page number |
|---|---|---|---|
| **Outcome name** | Average number of drugs per patient | | 157 |
| **Time points measured** *(specify whether from start or end of intervention)* | End of intervention | | 157 |
| **Time points reported** | End of intervention | | 157 |
| **Outcome definition** *(with diagnostic criteria if relevant)* | Average number of drugs per patient | | 157 |
| **Unit of measurement** *(if relevant)* | Prescription drug | | 157 |
| **Scales: upper and lower limits** *(indicate whether high or low score is good)* | Unclear. 0 upwards. | | 157 |
| **Is outcome/tool validated?** | No | | NA |
| **Imputation of missing data** *(e.g. assumptions made for ITT analysis)* | Not reported. | | NA |
| **Assumed risk estimate** *(e.g. baseline or population risk noted in Background)* | Not reported | | NA |
| **Power** *(e.g. power & sample size calculation, level of power achieved)* | The total number of patients to be studied in each group was determined by power analysis from previous studies. On the basis of standard deviations of the average number of drugs per patients be and changes that were thought to important, the study required at least 60 patients in each group. This was based on a two-tailed alpha of 0.05 and a power of 0.70. Thus, when comparing the data, we be could 70 per cent sure that a true difference existed if a difference was found in either direction at the 0.05 level.13 | | 156 |

| | |
|---|---|
| **Notes** | |

## Hospitalisations

| | Description as stated in report/paper | | Page number |
|---|---|---|---|
| **Outcome name** | Hospitalisation | | 157 |
| **Time points measured** *(specify whether from start or end of intervention)* | End of intervention | | 157 |
| **Time points reported** | End of intervention | | 157 |
| **Outcome definition** *(with diagnostic criteria if relevant)* | Acute hospitalisation (dates) | | 155 |
| **Unit of measurement** *(if relevant)* | Date, count occurrence | | 155, 157 |
| **Scales: upper and lower limits** *(indicate whether high or low score is good)* | 0 upwards | | 157 |
| **Is outcome/tool validated?** | No | | NA |
| **Imputation of missing data** *(e.g. assumptions made for ITT analysis)* | Not reported | | NA |
| **Assumed risk estimate** *(e.g. baseline or population risk noted in Background)* | Not reported | | NA |
| **Power** *(e.g. power & sample size calculation, level of power achieved)* | Not reported. | | NA |
| **Notes** | | | |

## Death

| | Description as stated in report/paper | Page number |
|---|---|---|
| **Outcome name** | Death | 157 |
| **Time points measured** *(specify whether from start or end of intervention)* | End of intervention | 157 |
| **Time points reported** | End of intervention | 157 |

| | | | |
|---|---|---|---|
| **Outcome definition** *(with diagnostic criteria if relevant)* | Morality rate | | 155 |
| **Unit of measurement** *(if relevant)* | Count | | 157 |
| **Scales: upper and lower limits** *(indicate whether high or low score is good)* | Alive/Dead | | 157 |
| **Is outcome/tool validated?** | No | | NA |
| **Imputation of missing data** *(e.g. assumptions made for ITT analysis)* | Not reported | | NA |
| **Assumed risk estimate** *(e.g. baseline or population risk noted in Background)* | Not reported | | NA |
| **Power** *(e.g. power & sample size calculation, level of power achieved)* | Not reported | | 156 |
| **Notes** | | | |

## Funding/conflict of interest

| | | |
|---|---|---|
| **Study funding sources** *(including role of funders)* | Supported by grant no. HS 03898 from the National Center for Health Services Research, OASH | 154 |
| **Possible conflicts of interest** *(for study authors)* | Not reported | NA |
| **Notes** | | |

## Data and analysis

### Average number of drugs per patient

| | **Description as stated in report/paper** | | **Page number** |
|---|---|---|---|
| **Outcome** | Average number of drugs per patient | | 157 |
| **Time point** *(specify from start or end of intervention)* | End of intervention | | |
| **Results** | Intervention | Comparison | 157 |

| | | | | | | | |
|---|---|---|---|---|---|---|---|
| | Mean | SD *(or other variance, specify)* | No. participants | Mean | SD *(or other variance, specify)* | No. participants | |
| | 5.7 | 3.29 | 67 | 7.1 | 13.65 | 72 | |
| **Any other results reported** *(e.g. mean difference, CI, P value)* | The average number of drugs prescribed per patient was significantly lower in the pharmacist group compared with the physician group during the study year (p=0.04). | | | | | | 157 |
| **No. missing participants** | Not reported (see earlier note about excluded 19) | | | | | | NA |
| **Statistical methods used and appropriateness of these** *(e.g. adjustment for correlation)* | T-test | | | | | | 157 |
| **Notes** | | | | | | | |

## Hospitalisations

| | **Description as stated in report/paper** | | | | **Page number** |
|---|---|---|---|---|---|
| **Outcome** | Hospitalisation | | | | 157 |
| **Time point** *(specify from start or end of intervention)* | End of intervention | | | | 157 |
| **Results** | Intervention | | Comparison | | 157 |
| | No. with event | Total in group | No. with event | Total in group | |
| | 2 | 67 | 8 | 72 | |
| **Any other results reported** | Although the hospitalization rates of the two groups had not been different in the prestudy period, the post-study analysis revealed a lower hospitalization rate in the pharmacist group compared with the physician group (p = 0.06) | | | | 157 |
| **No. missing participants** | Not reported (see earlier note about excluded 19) | | | | NA |
| **Reasons missing** | Not reported (see earlier note about excluded 19) | | | | NA |
| **Statistical methods used and appropriateness of these** | Chi-squared test | | | | |
| **Notes** | None | | | | |

## Deaths

| | Description as stated in report/paper | | | | Page number |
|---|---|---|---|---|---|
| **Outcome** | Death | | | | 157 |
| **Time point** *(specify from start or end of intervention)* | End of intervention | | | | 157 |
| **Results** | Intervention | | Comparison | | 157 |
| | No. with event | Total in group | No. with event | Total in group | |
| | 3 | 67 | 10 | 72 | |
| **Any other results reported** | The pharmacists' patients had statistically significantly better outcomes, including a lower mortality rate (p = 0.05). | | | | 157 |
| **No. missing participants** | Not reported (see earlier note about excluded 19) | | | | NA |
| **Reasons missing** | Not reported (see earlier note about excluded 19) | | | | NA |
| **Statistical methods used and appropriateness of these** | Chi-squared test | | | | |
| **Notes** | None | | | | |

## Conclusions

| | Description as stated in report/paper | Page number |
|---|---|---|
| **Key conclusions of study authors** | Although no studies to date have demonstrated the relationship in the clinical setting of the clinical pharmacists' superior knowledge of geriatric clinical pharmacology, we believe that this concept is supported by the improved outcomes demonstrated in this study in patients receiving drug therapy by clinical pharmacists. | 158 |
| **Notes** | | |

# Varghese et al. (2024)

## Study eligibility

| Study Characteristics | Eligibility criteria |
|---|---|
| **Title** | Implementation of a Pharmacist-Driven Aspirin Deprescribing Protocol Among Older Veterans in a Primary Care Setting |
| **Author (year)** | Varghese et al. (2024) |
| **Country** | Wisconsin, USA |
| **Type of study** | Prospective cohort |

| | |
|---|---|
| **Participants** | Atherosclerotic cardiovascular disease (ASCVD) |
| **Types of intervention** | Pharmacist-driven deprescribing protocol |
| **Types of comparison** | Primary care provider (PCP) |
| **Types of outcome measures** | Proportion of aspirin deprescribing (medication appropriateness) |
| **Prescriptive authority** | Protocol |
| **Include/Exclude** | Include |
| **Notes** | |

**DO NOT PROCEED IF STUDY EXCLUDED FROM REVIEW**

## Characteristics of included studies

### Methods

| | Descriptions as stated in report/paper | | Page number |
|---|---|---|---|
| **Aim of study** | To implement and evaluate the benefit of a pharmacist driven aspirin deprescribing protocol compared with primary care provider (PCP) education only in a primary care setting | | 229 |
| **Design** | Prospective cohort study | | 229 |
| **Unit of allocation** *(by individuals, cluster/ groups or body parts)* | Individual | | 229 |
| **Start-end date** | November 2021 | | 229 |
| **Duration of participation** *(from recruitment to last follow-up/ baseline to last follow-up- group level)* *1. Time of consent until last measurement for each individual.* *2. Baseline to final follow-up for each individual* | 4 months | | 229 |
| **Study duration** *(as above with the exception of interim analyses or other circumstances* | 4 months | | 229 |
| **Ethical approval needed/obtained for study** | Yes | This project was deemed as quality improvement and was therefore exempt from institutional review board approval at the VHA facility. | 229 |
| **Notes** | | | |

### Participants

| | **Description** *Include comparative information for each intervention or comparison group if available* | Page number |
|---|---|---|
| **Population description** *(from which study participants are drawn)* | Veterans with an active prescription of aspirin for ASCVD who were at least 70 years of age and had an established PCP at the VHA | 229 |

| | | | |
|---|---|---|---|
| **Setting** *(including location and social context)* | The Clement J. Zablocki VHA consists of four primary care clinics with more than 40 PCPs consisting of physician assistants, nurse practitioners, and physicians, and cares for more than 60,000 Veterans annually. The four clinics are located at the same facility and are similar in regard to how they function, and the training clinic staff receive | | 229 |
| **Inclusion/exclusion criteria** | Inclusion: Patients with an established PCP at the VHA as their main provider were evaluated for this project. Patients were included if they were at least 70 years of age and had been prescribed any formulation of aspirin 81 mg for primary prevention of ASCVD.<br><br>Exclusion: if they were prescribed clopidogrel, had a history of ASCVD (myocardial infarction, coronary artery disease, cerebrovascular accident, transient ischemic accident, peripheral artery disease), other documented indication of aspirin use, or were dual care patients (following with a PCP outside of the VHA). | | 230 |
| **Method of recruitment of participants** | One pharmacist in each clinic of the intervention group used a templated script to call eligible patients to have a risk versus benefit discussion. … If a patient was not able to be reached after three call attempts, a letter was sent regarding the updated guidance on the use of aspirin for older people, and the primary care clinic's contact information was provided. Discussions then took place with patients who chose to call the primary care clinics after receiving the letter. | | 230 |
| **Informed consent obtained** | No | | 229 |
| **Total no. randomised** *(or total pop. at start of study for NRCTs)* | 122 | | 230, 231 |
| **Clusters** | By clinic, 2 in intervention, 2 in control | | 229 |
| **Baseline imbalances** | Not reported | | NA |
| **Withdrawals and exclusions** | N = 9 unable to be reached from intervention arm | | 231 |
| **Age** | Intervention and comparator: 75 (70-01) | | 230, 232 |
| **Sex (female)** | Intervention: 0%<br>Comparator: 0% | | 230, 232 |
| **Subgroups measure** | None reported | | NA |
| **Subgroups reported** | None reported | | NA |
| **Notes** | | | |

## Intervention group

| | Description as stated in report/paper | Page number |
|---|---|---|
| **Group name** | Pharmacist deprescribing | 230, 231 |
| **No. randomised/assigned to group** *(specify whether no. people or clusters)* | 65 | 231 |
| **Description** *(include sufficient detail for replication, e.g. content, dose, components)* | In November 2021, a pharmacy resident gave a 10-minute virtual presentation to VHA's 4 primary care clinics on the updated evidence of aspirin use for primary prevention for older people. The presentations were done virtually, because of the COVID-19 social distancing precautions that were in place at the time. A handout of the summary of the evidence was provided for PACT future reference.<br><br>After the initial education, one pharmacist in each clinic of the intervention group used a templated script to call eligible patients to have a risk versus benefit discussion. The script contained an introduction, confirmation of aspirin use, and lack of prior ASCVD, the updated guidance from the 2019 ACC/AHA guideline, and finally the recommendation by the pharmacist to stop aspirin use with shared decision making. Results of the patient calls were documented in the patients' electronic health records using a standardized template, and the aspirin prescription was discontinued by the pharmacist, if appropriate. Given the project was a joint PACT effort and pharmacists have prescriptive authority, the PCP was notified of the patient's decision | 230 |
| **Duration of treatment period** | No set treatment period. Deprescribed once. Patient calls were approximately 5 minutes | 230, 232 |
| **Timing** | Once | 230 |
| **Co-interventions** | Pharmacists received education | 230 |
| **Notes** | | |

## Comparator group

| | Description as stated in report/paper | Page number |
|---|---|---|
| **Group name** | PCP education-only | 230,231 |
| **No. randomised/assigned to group** *(specify whether no. people or clusters)* | 57 | 230,231 |

| | | |
|---|---|---|
| **Description** *(include sufficient detail for replication, e.g. content, dose, components)* | In November 2021, a pharmacy resident gave a 10-minute virtual presentation to VHA's 4 primary care clinics on the updated evidence of aspirin use for primary prevention for older people. The presentations were done virtually, because of the COVID-19 social distancing precautions that were in place at the time. A handout of the summary of the evidence was provided for PACT future reference | 230 |
| **Duration of treatment period** | No set treatment period. Deprescribed once. | 230 |
| **Timing** | Once | 230 |
| **Co-interventions** | None/education | 230 |
| **Notes** | | |

## Outcomes

### Proportion of aspirin deprescribing (medication appropriateness)

| | **Description as stated in report/paper** | | **Page number** |
|---|---|---|---|
| **Outcome name** | Proportion of aspirin deprescribing | | 230 |
| **Time points measured** *(specify whether from start or end of intervention)* | Unclear, once at end of deprescribing intervention | | 230 |
| **Time points reported** | At end of intervention | | 230 |
| **Outcome definition** *(with diagnostic criteria if relevant)* | The primary outcome was the proportion of aspirin deprescribing defined by the number of patients no longer on aspirin at the end of the intervention period divided by the number of patients previously on aspirin. | | 230 |
| **Unit of measurement** *(if relevant)* | Event data | | 230 |
| **Scales: upper and lower limits** *(indicate whether high or low score is good)* | Higher proportion desired | | 230 |
| **Is outcome/tool validated?** | No | | 230 |
| **Imputation of missing data** *(e.g. assumptions made for ITT analysis)* | Not reported | | NA |
| **Assumed risk estimate** *(e.g. baseline or population risk noted in Background)* | Not reported | | NA |

| | | |
|---|---|---|
| **Power** *(e.g. power & sample size calculation, level of power achieved)* | Not reported | NA |
| **Notes** | | |

## Funding/conflict of interest

| | | |
|---|---|---|
| **Study funding sources** *(including role of funders)* | None reported | NA |
| **Possible conflicts of interest** *(for study authors)* | The authors report no real or potential financial conflicts of interest. | 228 |
| **Notes** | | |

## Data and analysis

### Proportion of aspirin deprescribing

| | **Description as stated in report/paper** | | | | **Page number** |
|---|---|---|---|---|---|
| **Outcome** | Proportion of aspirin deprescribing | | | | 230 |
| **Time point** *(specify from start or end of intervention)* | Unclear, once at end of deprescribing intervention | | | | 230 |
| **Results** | Intervention | | Comparison | | 230 |
| | No. with event | Total in group | No. with event | Total in group | |
| | 35 | 65 | 10 | 57 | |
| **Any other results reported** | At the end of the project, the rate of deprescribing was significantly higher in the intervention versus education-only group (35/65 [54%] vs 10/57 [18%]; *P* = 0.0001)<br><br>Of the 19 (29.2%) patients who declined, the most common reason (n = 11) was concern about cardiovascular health (Figure 1). | | | | 230 |
| **No. missing participants** | 0 | | | | NA |
| **Reasons missing** | 0 | | | | NA |
| **Statistical methods used and appropriateness of these** | The 2022 Dotmatics GraphPad Chi Square analysis tool was used to evaluate statistical significance of the primary outcome, and results were considered statistically significant if *P* < 0.05.16 | | | | 230 |
| **Notes** | None | | | | |

## Conclusions

| | **Description as stated in report/paper** | **Page number** |
|---|---|---|

| | | |
|---|---|---|
| **Key conclusions of study authors** | A pharmacist-led aspirin deprescribing protocol for primary prevention of ASCVD can be valuable as it helps individualize guideline-driven therapy to specific patients. This pharmacist-led intervention significantly increased the rate of aspirin deprescribing by three fold compared with PCP education only. | 233 |
| **Notes** | | |

## Villeneuve et al. 2010

### Study eligibility

| **Study Characteristics** | **Eligibility criteria** |
|---|---|
| **Title** | A cluster randomized controlled Trial to Evaluate an Ambulatory primary care Management program for patients with dyslipidemia: the TEAM study |
| **Author (year)** | Villeneuve et al. (2010) |
| **Country** | Québec, Canada |
| **Type of study** | Open cluster randomised controlled trial |
| **Participants** | Patients with dyslipidemia |
| **Types of intervention** | Pharmacist prescribing |
| **Types of comparison** | Usual care (physician prescribing) |
| **Types of outcome measures** | Low-density lipoprotein (LDL) cholesterol; target lipid level achieved; healthcare utilisation (physician appointments); adverse events; adherence |
| **Prescriptive authority** | Collaborative practice agreement |
| **Include/Exclude** | Include |
| **Notes** | |

**DO NOT PROCEED IF STUDY EXCLUDED FROM REVIEW**

## Characteristics of included studies

### Methods

| | Descriptions as stated in report/paper | | Page number |
|---|---|---|---|
| **Aim of study** | To compare a collaborative model involving physicians and pharmacists with usual care for patients with dyslipidemia. | | 447 |
| **Design** | Open cluster randomised controlled trial | | 447 |
| **Unit of allocation** *(by individuals, cluster/ groups or body parts)* | Clusters (15 clusters with 51 physicians and 49 pharmacists in the final analysis following 108 and 117 patients) | | 447 |
| **Start-end date** | May 2005 – January 2008 | | 447 |
| **Duration of participation** *(from recruitment to last follow-up/ baseline to last follow-up- group level)* *1. Time of consent until last measurement for each individual.* *2. Baseline to final follow-up for each individual* | 12 months | | 447 |
| **Study duration** *(as above with the exception of interim analyses or other circumstances* | 12 months | | 447 |
| **Ethical approval needed/obtained for study** | Yes | The study was approved by the ethics review boards of all three institutions. The health care professionals and patients provided written consent. | 448 |
| **Notes** | | | |

### Participants

| | **Description** *Include comparative information for each intervention or comparison group if available* | Page number |
|---|---|---|
| **Population description** *(from which study participants are drawn)* | French- or English-speaking adults (at least 18 years old) who were candidates for statin monotherapy or were already receiving it within inadequate control. | 448 |

| | | | |
|---|---|---|---|
| **Setting** *(including location and social context)* | Conventional/family medicine clinics | | 448 |
| **Inclusion/exclusion criteria** | Inclusion: At least 18 years; able to speak and read French or English; a patient at a participating pharmacy in the same cluster as the patient's physician or having no usual pharmacy; and a candidate (on the basis of laboratory results within the previous three months) for initiation of 3-hydroxy-3-methyl-glutaryl-coenzyme A reductase inhibitor (statin) monotherapy or already receiving statin monotherapy with inadequate control, where<br>inadequate control was defined as LDL cholesterol 2.5 mmol/L or higher and ratio of total cholesterol to high Density lipoprotein (HDL) cholesterol 4.0 or higher for high = risk patients (10-year risk for coronary artery disease 20%) or LDL cholesterol 3.5 mmol/L or higher and ratio of total cholesterol to HDL cholesterol 5.0 or higher for moderate risk patients (10-year risk for coronary artery disease 11%–19%).<br>Exclusion: We excluded patients who were taking more than one lipid-lowering medication, those who had experienced an acute cardiovascular event in the previous six months, those who were unlikely to complete the study because of life threatening disease or poor health, those with triglyceride level greater than 5.0 mmol/L, those with LDL cholesterol greater than 5.0 mmol/L, those with any contraindication to statin treatment and those who had participated in another clinical trial within the past two months. | | 448 |
| **Method of recruitment of participants** | In-person | | 448 |
| **Informed consent obtained** | Yes | The study was approved by the ethics review boards of all three institutions. The health care professionals and patients provided written consent. | 448 |
| **Total no. randomised** *(or total pop. at start of study for NRCTs)* | 225 | | 449 (Figure 1) |
| **Clusters** | Healthcare professionals.<br>We defined a cluster as participating physicians from one or two medical clinics located in close geographic proximity and pharmacists working in community pharmacies located within 5 km of these clinics. Initially, a cluster had to include at least five physicians and at least two pharmacies with a minimum of two pharmacists per pharmacy. In August 2005, to speed up recruitment, the minimum number of physicians per cluster was reduced to two. | | 448 |

| | | |
|---|---|---|
| **Baseline imbalances** | The health care professionals in the collaborative care and usual care groups were similar (Table 1). Compared with the usual care group, women constituted a greater proportion of physicians (56% v. 42%) and pharmacists (86% v. 57%) in the collaborative care group.<br><br>The majority of the patients were men, and most were at high risk for coronary artery disease (Table 1). Patients in the collaborative care group were younger (59.3 v. 62.2 years), were more likely to be new users of a statin (85% v. 73%) and were more likely to be smokers (35% v. 21%).<br><br>At baseline, patients in the collaborative care group had higher LDL cholesterol (3.5 v. 3.2 mmol/L, p = 0.05) and total cholesterol (5.7 v. 5.4 mmol/L, p = 0.01) (Table 2). At 12 months, patients in the collaborative care group had an additional reduction of 0.2 mmol/L in LDL cholesterol (95% CI –0.3 to –0.1) relative to patients in the usual care group. However, the adjusted difference was not statistically significant(–0.05 mmol/L, 95% CI –0.3 to 0.2). | 452 |
| **Withdrawals and exclusions** | 14 total<br>Intervention: 7. 2 withdrew, 5 did not attend final evaluation<br>Comparator: 7. 2 died, 1 withdrew, 4 did not attend final evaluation | 452, Figure 1 |
| **Age** | Intervention: 59.3 (9.6 SD)<br>Comparator: 62.2 (12.0 SD) | 450 |
| **Sex (female)** | Intervention: 15 (56%) physicians; 24 (86%) pharmacists; 31 (36%) patients<br>Comparator: 10 (42%) physicians; 12 (57%) pharmacists; 30 (40%) patients | 450 |
| **Subgroups measure** | None reported | NA |
| **Subgroups reported** | None reported | NA |
| **Notes** | | |

## Intervention group

| | Description as stated in report/paper | Page number |
|---|---|---|
| **Group name** | Pharmacist prescribing | 447 |
| **No. randomised/assigned to group** *(specify whether no. people or clusters)* | 108 individuals, 10 clinic clusters | 449 (Figure 1) |

| | | |
|---|---|---|
| **Description** *(include sufficient detail for replication, e.g. content, dose, components)* | Physician prescribes initial statin therapy.<br>During the patient's initial visit to the pharmacy (which lasted 30 minutes), the pharmacist provided counselling and used a patient decision aid to draw up a treatment plan, which included lifestyle changes and pharmacotherapy. The pharmacist then scheduled so-called titration visits (lasting 15 minutes each) at two-month intervals. During the titration visits, the pharmacist evaluated lifestyle changes, the patient's tolerance of and adherence with the pharmacotherapy, and the drug's efficacy and then adjusted the statin dosage accordingly. If necessary, the pharmacist scheduled an adherence visit (lasting 30 minutes) to discuss strategies to optimize treatment. When target lipid levels were achieved, the pharmacist scheduled a follow-up visit (15 minutes) for three months later.<br><br>The intervention ended when any one of the following milestones occurred: target lipid levels were reached at the follow-up visit, the patient had not achieved target lipid levels at the maximum statin dosage prescribed, the patient experienced severe intolerance to the drug or the patient asked to end the intervention. | 448 |
| **Duration of treatment period** | 12 months | 448 |
| **Timing** | Baseline, 15 minutes every 2 months until target lipid levels were met, then every 3 months. Additional adherence visits (30 minutes) if needed. 12 months time of follow-up in total<br><br>The pharmacist then scheduled so-called titration visits (lasting 15 minutes each) at two-month intervals. During the titration visits, the pharmacist evaluated lifestyle changes, the patient's tolerance of and adherence with the pharmacotherapy, and the drug's efficacy and then adjusted the statin dosage accordingly. If necessary, the pharmacist scheduled an adherence visit (lasting 30 minutes) to discuss strategies to optimize treatment. When target lipid levels were achieved, the pharmacist scheduled a follow-up visit (15 minutes) for three months later | 448 |
| **Co-interventions** | Decision support tool, counselling including lifestyle changes | 448 |
| **Notes** | | |

## Comparator group

| | Description as stated in report/paper | Page number |
|---|---|---|
| **Group name** | Usual care (physician prescribing) | 447 |
| **No. randomised/assigned to group** *(specify whether no. people or clusters)* | 117 individuals, 7 clusters | 449 (Figure 1) |
| **Description** *(include sufficient detail for replication, e.g. content, dose, components)* | Physicians in the usual care group requested laboratory tests and adjusted lipid-lowering pharmacotherapy. Pharmacists dispensed medications and provided usual counselling. The pharmacists in the usual care group did not receive any training specific to the study, and they did not meet the physicians in their cluster. | 448 |
| **Duration of treatment period** | Unspecified, baseline and 12 months | 448 |
| **Timing** | Not reported | NA |
| **Co-interventions** | None reported | NA |
| **Notes** | | |

## Outcomes

### LDL cholesterol

| | Description as stated in report/paper | Page number |
|---|---|---|
| **Outcome name** | Low-density lipoprotein (LDL) cholesterol/target level lipids achieved | 447 |
| **Time points measured** *(specify whether from start or end of intervention)* | Baseline, 12 months | 447 |
| **Time points reported** | Baseline, 12 months | 447 |
| **Outcome definition** *(with diagnostic criteria if relevant)* | Laboratory personnel at the Cité-de-la-Santé Hospital, Laval, Quebec, performed the analyses, and we estimated LDL cholesterol levels using the Friedwald equation. | 448 |
| **Unit of measurement** *(if relevant)* | Mmol/L | 452 |

| | | |
|---|---|---|
| **Scales: upper and lower limits** *(indicate whether high or low score is good)* | Shortly before the end of the recruitment phase, new lipid targets were defined by the 2006 Canadian guidelines, which recommended a target LDL cholesterol level less than 2.0 mmol/L for patients at high risk for coronary artery disease.<br>2003 guidelines = <2.5 (ref 8)<br><br>The target lipid levels were as recommended in the 2003 Canadian guidelines or individualized targets, at the physician's discretion. | 452<br><br>448<br><br>450 |
| **Is outcome/tool validated?** | No<br>12-hour fasting blood samples for measurement of blood glucose and serum lipid levels. | 448 |
| **Imputation of missing data** *(e.g. assumptions made for ITT analysis)* | In accordance with the intention-to treat principle, we estimated missing clinical data at the patient level, using the last-value-carried-forward approach (i.e., last results available in the medical chart or the baseline value). For all lipid values, the mean time (standard deviation [SD]) of the last value carried forward was 139 (109) days after the baseline evaluation in the collaborative care group and 150 (117) days after the base lineevaluation in the usual care group. | 450 |
| **Assumed risk estimate** *(e.g. baseline or population risk noted in Background)* | We used the Framingham equation to estimate the patient's 10-year risk of coronary artery disease. We used the following target levels: LDL cholesterol less than 3.5 mmol/L and ratio of total cholesterol to HDL cholesterol less than 5.0 for patients at moderate risk for coronary artery disease and LDL cholesterol less than 2.5 mmol/L and ratio of total cholesterol to HDL cholesterol less than 4.0 for patients at high risk for coronary artery disease. | 450 |
| **Power** *(e.g. power & sample size calculation, level of power achieved)* | We calculated that with 14 clusters and 16 patients per cluster, the study would have statistical power of 80% to detect a mean difference between groups of 0.5 mmol/L (SD 0.75 mmol/L) in the change in LDL cholesterol from baseline to 12 months, with a two-sided t test and a type 1 error of 5%. We assumed a within-cluster correlation coefficient of 0.08 and a dropout rate of 15%. | 451 |
| **Notes** | | |

## HDL cholesterol

| | **Description as stated in report/paper** | **Page number** |
|---|---|---|
| **Outcome name** | HDL cholesterol | 447 |

| | | | |
|---|---|---|---|
| **Time points measured** *(specify whether from start or end of intervention)* | Baseline, 12 months | | 447 |
| **Time points reported** | Baseline, 12 months | | 447 |
| **Outcome definition** *(with diagnostic criteria if relevant)* | HDL cholesterol | | 447 |
| **Unit of measurement** *(if relevant)* | Mmol/L | | 452 |
| **Scales: upper and lower limits** *(indicate whether high or low score is good)* | The target lipid levels were as recommended in the 2003 Canadian guidelines or individualized targets, at the physician's discretion. | | 448 |
| **Is outcome/tool validated?** | No | 12-hour fasting blood samples for measurement of blood glucose and serum lipid levels. | 448 |
| **Imputation of missing data** *(e.g. assumptions made for ITT analysis)* | In accordance with the intention-to treat principle, we estimated missing clinical data at the patient level, using the last-value-carried-forward approach (i.e., last results available in the medical chart or the baseline value). For all lipid values, the mean time (standard deviation [SD]) of the last value carried forward was 139 (109) days after the baseline evaluation in the collaborative care group and 150 (117) days after the base line evaluation in the usual care group. | | 450 |
| **Assumed risk estimate** *(e.g. baseline or population risk noted in Background)* | We used the Framingham equation to estimate the patient's 10-year risk of coronary artery disease. We used the following target levels: LDL cholesterol less than 3.5 mmol/L and ratio of total cholesterol to HDL cholesterol less than 5.0 for patients at moderate risk for coronary artery disease and LDL cholesterol less than 2.5 mmol/L and ratio of total cholesterol to HDL cholesterol less than 4.0 for patients at high risk for coronary artery disease. | | 450 |
| **Power** *(e.g. power & sample size calculation, level of power achieved)* | Not reported | | NA |
| **Notes** | | | |

## Triglyercides

| | Description as stated in report/paper | Page number |
|---|---|---|
| **Outcome name** | Triglycerides | 451 (table 2) |

| | | | |
|---|---|---|---|
| **Time points measured** *(specify whether from start or end of intervention)* | Baseline, 12 months | | 447 |
| **Time points reported** | Baseline, 12 months | | 447 |
| **Outcome definition** *(with diagnostic criteria if relevant)* | Triglycerides | | 451 (table 2) |
| **Unit of measurement** *(if relevant)* | Mmol/L | | 452 |
| **Scales: upper and lower limits** *(indicate whether high or low score is good)* | The target lipid levels were as recommended in the 2003 Canadian guidelines or individualized targets, at the physician's discretion. | | 448 |
| **Is outcome/tool validated?** | No | 12-hour fasting blood samples for measurement of blood glucose and serum lipid levels. | 448 |
| **Imputation of missing data** *(e.g. assumptions made for ITT analysis)* | In accordance with the intention-to treat principle, we estimated missing clinical data at the patient level, using the last-value-carried-forward approach (i.e., last results available in the medical chart or the baseline value). For all lipid values, the mean time (standard deviation [SD]) of the last value carried forward was 139 (109) days after the baseline evaluation in the collaborative care group and 150 (117) days after the base line evaluation in the usual care group. | | 450 |
| **Assumed risk estimate** *(e.g. baseline or population risk noted in Background)* | Not reported | | NA |
| **Power** *(e.g. power & sample size calculation, level of power achieved)* | Not reported | | NA |
| **Notes** | | | |

## Proportion achieving target lipid levels

| | Description as stated in report/paper | Page number |
|---|---|---|
| **Outcome name** | Proportion achieving target lipid levels | 451 (table 2) |
| **Time points measured** *(specify whether from start or end of intervention)* | Baseline, 12 months | 447 |
| **Time points reported** | Baseline, 12 months | 447 |

| | | | |
|---|---|---|---|
| **Outcome definition** *(with diagnostic criteria if relevant)* | The target lipid levels were as recommended in the 2003 Canadian guidelines or individualized targets, at the physician's discretion. | | 448 |
| **Unit of measurement** *(if relevant)* | Proportion | | 452 |
| **Scales: upper and lower limits** *(indicate whether high or low score is good)* | Higher proportion achieving target is desired | | NA |
| **Is outcome/tool validated?** | No | 12-hour fasting blood samples for measurement of blood glucose and serum lipid levels. | 448 |
| **Imputation of missing data** *(e.g. assumptions made for ITT analysis)* | In accordance with the intention-to treat principle, we estimated missing clinical data at the patient level, using the last-value-carried-forward approach (i.e., last results available in the medical chart or the baseline value). For all lipid values, the mean time (standard deviation [SD]) of the last value carried forward was 139 (109) days after the baseline evaluation in the collaborative care group and 150 (117) days after the base line evaluation in the usual care group. | | 450 |
| **Assumed risk estimate** *(e.g. baseline or population risk noted in Background)* | We used the Framingham equation to estimate the patient's 10-year risk of coronary artery disease. We used the following target levels: LDL cholesterol less than 3.5 mmol/L and ratio of total cholesterol to HDL cholesterol less than 5.0 for patients at moderate risk for coronary artery disease and LDL cholesterol less than 2.5 mmol/L and ratio of total cholesterol to HDL cholesterol less than 4.0 for patients at high risk for coronary artery disease. | | 450 |
| **Power** *(e.g. power & sample size calculation, level of power achieved)* | | | |
| **Notes** | | | |

## Systolic blood pressure

| | Description as stated in report/paper | Page number |
|---|---|---|
| **Outcome name** | Systolic blood pressure | 451 (table 2) |
| **Time points measured** *(specify whether from start or end of intervention)* | Baseline, 12 months | 447 |
| **Time points reported** | Baseline, 12 months | 447 |
| **Outcome definition** *(with diagnostic criteria if relevant)* | Systolic blood pressure | 447 |

| | | | |
|---|---|---|---|
| **Unit of measurement** *(if relevant)* | mm Hg | | 452 |
| **Scales: upper and lower limits** *(indicate whether high or low score is good)* | Lower is desired | | NA |
| **Is outcome/tool validated?** | No | Three blood pressure readings after the patient had been seated for at least five minutes. We calculated and recorded the mean of the second and third readings. | 450 |
| **Imputation of missing data** *(e.g. assumptions made for ITT analysis)* | In accordance with the intention-to treat principle, we estimated missing clinical data at the patient level, using the last-value-carried-forward approach (i.e., last results available in the medical chart or the baseline value). | | 450 |
| **Assumed risk estimate** *(e.g. baseline or population risk noted in Background)* | Not reported | | NA |
| **Power** *(e.g. power & sample size calculation, level of power achieved)* | Not reported | | NA |
| **Notes** | | | |

## Diastolic blood pressure

| | Description as stated in report/paper | | Page number |
|---|---|---|---|
| **Outcome name** | | | |
| **Time points measured** *(specify whether from start or end of intervention)* | Baseline, 12 months | | 447 |
| **Time points reported** | Baseline, 12 months | | 447 |
| **Outcome definition** *(with diagnostic criteria if relevant)* | Diastolic blood pressure | | NA |
| **Unit of measurement** *(if relevant)* | mm Hg | | 452 |
| **Scales: upper and lower limits** *(indicate whether high or low score is good)* | Lower levels are desired | | NA |
| **Is outcome/tool validated?** | No | Three blood pressure readings after the patient had been seated for at least five minutes. We calculated and recorded the mean of the second and third readings. | 450 |

| | | |
|---|---|---|
| **Imputation of missing data** *(e.g. assumptions made for ITT analysis)* | In accordance with the intention-to treat principle, we estimated missing clinical data at the patient level, using the last-value-carried-forward approach (i.e., last results available in the medical chart or the baseline value). | 450 |
| **Assumed risk estimate** *(e.g. baseline or population risk noted in Background)* | Not reported | NA |
| **Power** *(e.g. power & sample size calculation, level of power achieved)* | Not reported | NA |
| **Notes** | | |

## Fasting blood glucose

| | Description as stated in report/paper | | Page number |
|---|---|---|---|
| **Outcome name** | Fasting blood glucose | | 451 (table 2) |
| **Time points measured** *(specify whether from start or end of intervention)* | Baseline, 12 months | | 447 |
| **Time points reported** | Baseline, 12 months | | 447 |
| **Outcome definition** *(with diagnostic criteria if relevant)* | Fasting blood glucose | | 451 (table 2) |
| **Unit of measurement** *(if relevant)* | Mmol/L | | 452 |
| **Scales: upper and lower limits** *(indicate whether high or low score is good)* | Lower level is desired | | NA |
| **Is outcome/tool validated?** | No | 12-hour fasting blood samples for measurement of blood glucose and serum lipid levels. | 448 |
| **Imputation of missing data** *(e.g. assumptions made for ITT analysis)* | In accordance with the intention-to treat principle, we estimated missing clinical data at the patient level, using the last-value-carried-forward approach (i.e., last results available in the medical chart or the baseline value). | | 450 |
| **Assumed risk estimate** *(e.g. baseline or population risk noted in Background)* | Not reported | | NA |
| **Power** *(e.g. power & sample size calculation, level of power achieved)* | Not reported | | NA |
| **Notes** | | | |

## Healthcare utilisation (physician appointments)

| | Description as stated in report/paper | | Page number |
|---|---|---|---|
| **Outcome name** | Healthcare utilisation (physician appointments) | | 453 |
| **Time points measured** *(specify whether from start or end of intervention)* | 12 months | | 453 |
| **Time points reported** | 12 months | | 453 |
| **Outcome definition** *(with diagnostic criteria if relevant)* | Use of health care services -- No. of physician visits | | 453 |
| **Unit of measurement** *(if relevant)* | Visit count | | 453 |
| **Scales: upper and lower limits** *(indicate whether high or low score is good)* | Not reported | | NA |
| **Is outcome/tool validated?** | No | We reviewed the patients' medical charts and the pharmacists' log books to document visits to clinicians and laboratory tests. We reviewed the pharmacies' dispensing charts to document patients' receipt of lipid-lowering pharmacotherapy. | 450 |
| **Imputation of missing data** *(e.g. assumptions made for ITT analysis)* | In accordance with the intention-to treat principle, we estimated missing clinical data at the patient level, using the last-value-carried-forward approach (i.e., last results available in the medical chart or the baseline value). | | 450 |
| **Assumed risk estimate** *(e.g. baseline or population risk noted in Background)* | NA | | NA |
| **Power** *(e.g. power & sample size calculation, level of power achieved)* | Not reported | | NA |
| **Notes** | | | |

## Adherence

| | Description as stated in report/paper | Page number |
|---|---|---|
| **Outcome name** | Adherence | 453 |
| **Time points measured** *(specify whether from start or end of intervention)* | 12 months | 453 |

| | | | |
|---|---|---|---|
| **Time points reported** | 12 months | | 453 |
| **Outcome definition** *(with diagnostic criteria if relevant)* | We deemed as "adherent" those patients for whom the amount of medication dispensed covered at least 80% of the number of days during the follow-up period We computed the proportion of days during the follow-up | | 451 |
| **Unit of measurement** *(if relevant)* | Period with coverage by the medication dispensed = The denominator was the number of days between baseline and the date of the 12-month evaluation (or the date of death). For patients taking more than one lipid-lowering medication and those for whom a new medication was dispensed during the period covered by a previous prescription, we counted the overlapping number of days of available therapy just once. | | 453 |
| **Scales: upper and lower limits** *(indicate whether high or low score is good)* | <80% = not adherent<br>≥80% = adherent | | 451 |
| **Is outcome/tool validated?** | No | We reviewed the pharmacies' dispensing charts to document patients' receipt of lipid-lowering pharmacotherapy. | 450 |
| **Imputation of missing data** *(e.g. assumptions made for ITT analysis)* | Not reported | | NA |
| **Assumed risk estimate** *(e.g. baseline or population risk noted in Background)* | NA | | NA |
| **Power** *(e.g. power & sample size calculation, level of power achieved)* | Not reported | | NA |
| **Notes** | | | |

## Adverse events

| | Description as stated in report/paper | Page number |
|---|---|---|
| **Outcome name** | Adverse events | 453 |
| **Time points measured** *(specify whether from start or end of intervention)* | 12 months | 453 |
| **Time points reported** | 12 months | 453 |

| | | | |
|---|---|---|---|
| **Outcome definition** *(with diagnostic criteria if relevant)* | Treatment stoppage due to adverse events including: muscular pain, effects on the central nervous system, specifically insomnia or headache, or gastrointestinal effects | | 454 |
| **Unit of measurement** *(if relevant)* | Count | | 453 |
| **Scales: upper and lower limits** *(indicate whether high or low score is good)* | Not reported | | NA |
| **Is outcome/tool validated?** | No | Self-report. At the 12-month evaluation, the patient completed a self-administered questionnaire to document lifestyle changes and adverse events. | 450 |
| **Imputation of missing data** *(e.g. assumptions made for ITT analysis)* | Not reported | | 450 |
| **Assumed risk estimate** *(e.g. baseline or population risk noted in Background)* | NA | | NA |
| **Power** *(e.g. power & sample size calculation, level of power achieved)* | Not reported | | NA |
| **Notes** | | | |

## Funding/conflict of interest

| | | |
|---|---|---|
| **Study funding sources** *(including role of funders)* | 'This research was funded by a grant from the Canadian Institutes of Health Research (grant number 200409MCT-133732-RCT) and unrestricted research grants from AstraZeneca Canada Inc., Merck Frosst Canada Ltd. and Pfizer Canada Inc. Research scholars Lyne Lalonde, Lucie Blais and Sylvie Perreault received financial support from the Fonds de la recherche en santé du Québec. | 454 |

| | | |
|---|---|---|
| **Possible conflicts of interest** *(for study authors)* | 'Marie-Thérèse Lussier is the principal investigator for a research project funded by AstraZeneca Canada. She has received educational grants for adaptation and translation of patient communication papers from Merck, AstraZeneca and Sanofi. She received sponsorship from AstraZeneca to attend the European Association for Communication in Health Care (EACH) meeting in September 2008. None declared for all other authors. | 454 |
| **Notes** | | |

## Data and analysis

### LDL cholesterol

| | Description as stated in report/paper | | | | | | Page number |
|---|---|---|---|---|---|---|---|
| **Outcome** | Low-density lipoprotein (LDL) cholesterol | | | | | | 448 |
| **Time point** *(specify from start or end of intervention)* | Baseline, 12 months | | | | | | 448 |
| **Results: Change from baseline to 12 months** | Intervention | | | Comparison | | | 451 (table 2) |
| | Mean difference | 95% CI | No. participants | Mean difference | 95% CI | No. participants | |
| | -1.1 | -1.3 - -1.0 | 108 | -0.9 | -1.0 - -0.8 | 117 | |
| **Any other results reported** *(e.g. mean difference, CI, P value)* | At 12 months, after adjustment for potential confounders, collaborative care was not associated with a significant incremental reduction in LDL cholesterol (–0.05 mmol/L, 95% CI –0.3 to 0.2) (adjusted for use of statin and LDL cholesterol at baseline). [table 2]<br><br>And the probability of reaching target lipid levels was only slightly higher among patients receiving collaborative care (RR 1.16, 95% CI 1.01 to 1.34). [table 3]<br><br>When the analysis was stratified by risk for coronary artery disease, treatment status and type of medical clinic within the clusters, there was no incremental reduction in LDL cholesterol for patients in the collaborative care group (Table 3). | | | | | | 451, 454, 452 |
| **No. missing participants** | 8 | | | 8 | | | |

| | | |
|---|---|---|
| **Statistical methods used and appropriateness of these** *(e.g. adjustment for correlation)* | To compare mean changes in LDL cholesterol (defined as value at 12 months minus baseline value) and other risk factors, with adjustment for baseline characteristics of patients and clusters of health care professionals, we developed multivariable linear regression models taking into account the within cluster correlation.<br><br>We included in the multivariable models those variables deemed significant (p < 0.2) in a bivariable model including treatment group. We then applied a backward selection procedure and included in the final model those variables that were statistically significant at p < 0.1. | 450-451 |
| **Notes** | | |

## HDL cholesterol

| | Description as stated in report/paper | | | | | | Page number |
|---|---|---|---|---|---|---|---|
| **Outcome** | High-density lipoprotein (LDL) cholesterol | | | | | | 448 |
| **Time point** *(specify from start or end of intervention)* | Baseline, 12 months | | | | | | 448 |
| **Results: Change from baseline to 12 months** | Intervention | | | Comparison | | | 451 (table 2) |
| | Mean difference | 95% CI | No. participants | Mean difference | 95% CI | No. participants | |
| | -0.05 | –0.09 to –0.01 | 108 | 0.02 | 0.03 to 0.07 | 117 | |
| **Any other results reported** *(e.g. mean difference, CI, P value)* | The unadjusted difference between collaborative and usual care groups was 0.02 mmol/L (95% CI –0.03 to 0.07). | | | | | | 451 (table 2) |
| **No. missing participants** | 8 | | | 8 | | | |
| **Statistical methods used and appropriateness of these** *(e.g. adjustment for correlation)* | To compare mean changes in LDL cholesterol (defined as value at 12 months minus baseline value) and other risk factors, with adjustment for baseline characteristics of patients and clusters of health care professionals, we developed multivariable linear regression models taking into account the within cluster correlation.<br><br>We included in the multivariable models those variables deemed significant (p < 0.2) in a bivariable model including treatment group. We then applied a backward selection procedure and included in the final model those variables that were statistically significant at p < 0.1. | | | | | | 450-451 |
| **Notes** | | | | | | | |

## Triglycerides

| | Description as stated in report/paper | | | | | | Page number |
|---|---|---|---|---|---|---|---|
| **Outcome** | Triglycerides | | | | | | 448 |
| **Time point** *(specify from start or end of intervention)* | Baseline, 12 months | | | | | | 448 |
| **Results: Change from baseline to 12 months** | Intervention | | | Comparison | | | 451 (Table 2) |
| | Mean difference | 95% CI | No. participants | Mean difference | 95% CI | No. participants | |
| | –0.3 | –0.4 to –0.1 | 108 | –0.3 | –0.4 to –0.2 | 117 | |
| **Any other results reported** *(e.g. mean difference, CI, P value)* | The unadjusted difference between collaborative and usual care groups was 0.02 mmol/L (95% CI –0.2 to 0.2).<br><br>The adjusted difference between collaborative and usual care groups was -0.03 mmol/L (95% CI –0.2 to 0.1) (adjusted for patient's age and hourly number of prescriptions per pharmacist). | | | | | | 451 (table 2) |
| **No. missing participants** | 0 | | | 0 | | | |
| **Statistical methods used and appropriateness of these** *(e.g. adjustment for correlation)* | To compare mean changes in LDL cholesterol (defined as value at 12 months minus baseline value) and other risk factors, with adjustment for baseline characteristics of patients and clusters of health care professionals, we developed multivariable linear regression models taking into account the within cluster correlation.<br><br>We included in the multivariable models those variables deemed significant (p < 0.2) in a bivariable model including treatment group. We then applied a backward selection procedure and included in the final model those variables that were statistically significant at p < 0.1. | | | | | | 450-451 |
| **Notes** | | | | | | | |

## 3.4 Proportion achieving target lipid levels

| | Description as stated in report/paper | | Page number |
|---|---|---|---|
| **Outcome** | Proportion achieving target lipid levels | | 447, 451 |
| **Time point** *(specify from start or end of intervention)* | Baseline, 12 months | | 447, 451 |
| **Results** | Intervention | Comparison | 452 |

| | | | | | |
|---|---|---|---|---|---|
| | No. with event | Total in group | No. with event | Total in group | |
| | 87 | 108 | 86 | 117 | |
| **Any other results reported** | At 12 months, 87 (81%) of the patients in the collaborative care group and 86 (74%) of those in the usual care group had reached their target lipid levels (crude RR 1.10, 95% CI 0.95 to 1.26), as defined by the 2003 Canadian guidelines.8 After adjustment for baseline LDL cholesterol, patients in the collaborative care group were significantly more likely to reach their targets (RR 1.16, 95% CI 1.01 to 1.34).<br><br>Shortly before the end of the recruitment phase, new lipid targets were defined by the 2006 Canadian guidelines,19 which recommended a target LDL cholesterol level less than 2.0 mmol/L for patients at high risk for coronary artery disease. The proportion of high-risk patients who had reached this target at 12 months was 49% (39/80) in the collaborative care group and 43% (42/97) in the usual care group (adjusted RR 1.2, 95% CI 0.9 to 1.7). For patients in the collaborative care group, the physician had to specify the lipid targets on the prescription form. On the basis of this information, 21 patients in the collaborative care group who were at high risk for coronary artery disease had targets prescribed as defined in the 2006 guidelines, and 17 (81%) of them had reached their target at 12 months | | | | 452 |
| **No. missing participants** | 8 | | 8 | | 451 |
| **Reasons missing** | Only 7 listed in flow diagram. 8 noted in table 2 notes | | Only 7 listed in flow diagram. 8 noted in table 2 notes | | 449, 451 |
| **Statistical methods used and appropriateness of these** | We computed the proportion of patients in each group who were at or below their target lipid levels at 12 months. We estimated relative risks (RR) and 95% confidence intervals (95% CI), with the usual care group as the reference group, using generalized linear models, taking into account the within-cluster correlation, with a log link and robust sandwich variance estimator using a modified Poisson regression. The final multivariable models were defined as described previously. | | | | 451 |
| **Notes** | None | | | | |

## Systolic blood pressure

| | Description as stated in report/paper | Page number |
|---|---|---|
| **Outcome** | Systolic blood pressure | 448 |

| | | | | | | | |
|---|---|---|---|---|---|---|---|
| **Time point** *(specify from start or end of intervention)* | Baseline, 12 months | | | | | | 448 |
| **Results: Change from baseline to 12 months** | Intervention | | | Comparison | | | 451 (Table 2) |
| | Mean difference | 95% CI | No. participants | Mean difference | 95% CI | No. participants | |
| | 1.2 | –2.9 to 5.3 | 108 | 2.5 | –1.4 to 6.4 | 117 | |
| **Any other results reported** *(e.g. mean difference, CI, P value)* | The unadjusted difference between collaborative and usual care groups was –1.3 (–6.4 to 3.8). | | | | | | 451 (table 2) |
| **No. missing participants** | 10 | | | 9 | | | 451 (table 2) |
| **Statistical methods used and appropriateness of these** *(e.g. adjustment for correlation)* | To compare mean changes in LDL cholesterol (defined as value at 12 months minus baseline value) and other risk factors, with adjustment for baseline characteristics of patients and clusters of health care professionals, we developed multivariable linear regression models taking into account the within cluster correlation.<br><br>We included in the multivariable models those variables deemed significant ($p < 0.2$) in a bivariable model including treatment group. We then applied a backward selection procedure and included in the final model those variables that were statistically significant at $p < 0.1$. | | | | | | 450-451 |
| **Notes** | | | | | | | |

## Diastolic blood pressure

| | Description as stated in report/paper | | | | | | Page number |
|---|---|---|---|---|---|---|---|
| **Outcome** | Systolic blood pressure | | | | | | 448 |
| **Time point** *(specify from start or end of intervention)* | Baseline, 12 months | | | | | | 448 |
| **Results: Change from baseline to 12 months** | Intervention | | | Comparison | | | 451 (Table 2) |
| | Mean difference | 95% CI | No. participants | Mean difference | 95% CI | No. participants | |
| | –0.3 | –3.5 to 2.9 | 108 | 1.7 | 0.2 to 3.2 | 117 | |

| | | | |
|---|---|---|---|
| **Any other results reported** *(e.g. mean difference, CI, P value)* | The unadjusted difference between collaborative and usual care groups was –1.8 (–5.0 to 1.4). | | 451 (table 2) |
| **No. missing participants** | 10 | 9 | 451 (table 2) |
| **Statistical methods used and appropriateness of these** *(e.g. adjustment for correlation)* | To compare mean changes in LDL cholesterol (defined as value at 12 months minus baseline value) and other risk factors, with adjustment for baseline characteristics of patients and clusters of health care professionals, we developed multivariable linear regression models taking into account the within cluster correlation.<br><br>We included in the multivariable models those variables deemed significant (p < 0.2) in a bivariable model including treatment group. We then applied a backward selection procedure and included in the final model those variables that were statistically significant at p < 0.1. | | 450-451 |
| **Notes** | | | |

## Fasting blood glucose

| | Description as stated in report/paper | | | | | | Page number |
|---|---|---|---|---|---|---|---|
| **Outcome** | Fasting blood glucose | | | | | | 448 |
| **Time point** *(specify from start or end of intervention)* | Baseline, 12 months | | | | | | 448 |
| **Results: Change from baseline to 12 months** | Intervention | | | Comparison | | | 451 (Table 2) |
| | Mean difference | 95% CI | No. participants | Mean difference | 95% CI | No. participants | |
| | –0.1 | –0.4 to 0.1 | 108 | 0.01 | –0.5 to 0.5 | 117 | |
| **Any other results reported** *(e.g. mean difference, CI, P value)* | The unadjusted difference between collaborative and usual care groups was –0.1 (–0.6 to 0.4). | | | | | | 451 (table 2) |
| **No. missing participants** | 9 | | | 9 | | | 451 (table 2) |

| | | |
|---|---|---|
| **Statistical methods used and appropriateness of these** *(e.g. adjustment for correlation)* | To compare mean changes in LDL cholesterol (defined as value at 12 months minus baseline value) and other risk factors, with adjustment for baseline characteristics of patients and clusters of health care professionals, we developed multivariable linear regression models taking into account the within cluster correlation.<br><br>We included in the multivariable models those variables deemed significant (p < 0.2) in a bivariable model including treatment group. We then applied a backward selection procedure and included in the final model those variables that were statistically significant at p < 0.1. | 450-451 |
| **Notes** | | |

## Healthcare utilisation (physician appointments)

| | Description as stated in report/paper | | | | | | Page number |
|---|---|---|---|---|---|---|---|
| **Outcome** | Healthcare utilisation (physician appointments) | | | | | | 453 |
| **Time point** *(specify from start or end of intervention)* | 12 months | | | | | | 453 |
| **Results: Change from baseline to 12 months** | Intervention | | | Comparison | | | 453 (Table 4) |
| | Mean | SD | No. participants | Mean | 95% CI | No. participants | |
| | 3.2 | 2.9 | 108 | 3.7 | 2.4 | 117 | |
| **Any other results reported** *(e.g. mean difference, CI, P value)* | During the 12-month follow-up, the mean number of visits to a physician did not differ between groups.<br>RR 95% CI: -0.45 (-1.48 – 0.58) between group difference | | | | | | 453 (Table 4) |
| **No. missing participants** | 7 | | | 7 | | | 449 (Figure 1) |
| **Statistical methods used and appropriateness of these** *(e.g. adjustment for correlation)* | We compared lipid-lowering treatment and use of health care services between the two groups using univariable linear regression or modified Poisson regression models, taking the within-cluster correlation into account. | | | | | | 451 |
| **Notes** | | | | | | | |

## Adherence

| | Description as stated in report/paper | Page number |
|---|---|---|
| **Outcome** | Adherence | 453 |

| | | | | | |
|---|---|---|---|---|---|
| **Time point** *(specify from start or end of intervention)* | 12 months | | | | 453 |
| **Results** | Intervention | | Comparison | | 453 |
| | No. with event | Total in group | No. with event | Total in group | |
| | 78 | 108 | 79 | 117 | |
| **Any other results reported** | …most had sufficient medication to cover at least 80% of the follow-up period (representing adherence ≥ 80%; 78 [72%] of patients receiving collaborative care and 79 [68%] receiving usual care). As noted above, analysis for a dispensing interval of 45 days did not change the results. | | | | 453 |
| **No. missing participants** | 7 | | 7 | | NA |
| **Reasons missing** | Reasons given in Figure 1 | | Reasons given in Figure 1 | | 449 |
| **Statistical methods used and appropriateness of these** | Not reported. Descriptive. | | | | NA |
| **Notes** | None | | | | |

## Adverse events

| | Description as stated in report/paper | | | | Page number |
|---|---|---|---|---|---|
| **Outcome** | Adverse events | | | | 454 |
| **Time point** *(specify from start or end of intervention)* | 12 months | | | | 454 |
| **Results** | Intervention | | Comparison | | 454 |
| | No. with event | Total in group | No. with event | Total in group | |
| | 8 | 101 | 8 | 110 | |
| **Any other results reported** | Sixteen patients reported stopping their statin treatment because of adverse events (muscular pain, effects on the central nervous system, or gastrointestinal effects). | | | | 454 |
| **No. missing participants** | Not reported | | Not reported | | NA |
| **Reasons missing** | Not reported | | Not reported | | NA |
| **Statistical methods used and appropriateness of these** | Not reported. Descriptive. | | | | NA |
| **Notes** | None | | | | |

## Conclusions

| | Description as stated in report/paper | Page number |
|---|---|---|

| | | |
|---|---|---|
| **Key conclusions of study authors** | Collaborative primary care is feasible for the management of dyslipidemia, but it does not necessarily have any effect on patients' outcomes.<br><br>In a collaborative primary care model in which community pharmacists were responsible for titrating lipid-lowering medication,<br>patients made more visits to health care professionals than patients receiving usual care, underwent more laboratory tests, were more likely to have their lipid-lowering treatment modified and were more likely to report making lifestyle changes. However, these differences did not translate into a significant clinical effect on lipid control. At 12 months, after adjustment for potential confounders, collaborative care was not associated with a significant incremental reduction in LDL cholesterol (–0.05 mmol/L, 95% CI –0.3 to 0.2), and the probability of reaching target lipid levels was only slightly higher among patients receiving collaborative care (RR 1.16, 95% CI 1.01 to 1.34). | 454 |
| **Notes** | | |

## Vivian et al. 2002

### Study eligibility

| **Study Characteristics** | **Eligibility criteria** |
|---|---|
| **Title** | Improving Blood Pressure Control in a Pharmacist-Managed Hypertension Clinic |
| **Author (year)** | Vivian et al. 2002 |
| **Country** | Pennsylvania, USA |
| **Type of study** | Prospective, controlled study |
| **Participants** | (Veterans) Patients with essential hypertension |
| **Types of intervention** | Pharmacist prescribing hypertension medications |
| **Types of comparison** | Standard care from physicians |
| **Types of outcome measures** | Blood pressure; compliance; quality of life |
| **Include/Exclude** | Include |
| **Prescriptive authority** | By protocol (in accordance with hypertension guidelines) |
| **Notes** | |

**DO NOT PROCEED IF STUDY EXCLUDED FROM REVIEW**

## Characteristics of included studies

### Methods

| | Descriptions as stated in report/paper | | Page number |
|---|---|---|---|
| **Aim of study** | To determine whether a pharmacist-managed hypertension clinic improves treatment outcomes in patients with hypertension. | | 1534 |
| **Design** | Prospective, controlled study | | 1534 |
| **Unit of allocation** *(by individuals, cluster/ groups or body parts)* | Individuals | | 1534 |
| **Start-end date** | Not reported | | NA |
| **Duration of participation** *(from recruitment to last follow-up)* | Six months | | 1534 |
| **Study duration** | Six months | | 1534 |
| **Ethical approval needed/obtained for study** | Yes | After the investigational review board approved the study. | 1534 |
| **Notes** | | | |

### Participants

| | **Description** *Include comparative information for each intervention or comparison group if available* | Page number |
|---|---|---|
| **Population description** *(from which study participants are drawn)* | Veterans with a confirmed diagnosis of hypertension | 1534 |
| **Setting** *(including location and social context)* | General medicine clinic within VA | 1534 |

| | | | |
|---|---|---|---|
| **Inclusion/exclusion criteria** | Inclusion: All patients enrolled in the study had stage 1, 2, or 3 hypertension as defined by the eligibility criteria and by the sixth report of the Joint National Committee on the Detection, Evaluation, and Treatment of High Blood Pressure (JNC VI). Inclusion criteria were age older than 18 years, confirmed diagnosis of essential hypertension (defined as systolic blood pressure > 140 mm Hg or diastolic blood pressure > 90 mm Hg), receiving antihypertensive drug therapy (and blood pressure > 140/90 mm Hg), receiving all drugs from a Veterans Affairs Medical Center pharmacy (for assessment of compliance and drug interactions), and not receiving care at the pharmacist-managed clinic (until the study began).<br><br>Exclusion: Patients were excluded if they had a secondary cause of hypertension, such as chronic renal disease, renovascular disease, pheochromocytoma, Cushing's syndrome, and primary aldosteronism; had missed more than three appointments in the last year; or were in hypertensive crisis (defined as systolic blood pressure > 210 mm Hg or diastolic blood pressure > 110 mm Hg). Patients also were excluded if they had a diagnosis of New York Heart Association class III or IV chronic heart failure, end-stage renal disease, a psychiatric disorder, severe hepatic dysfunction defined as transaminase levels greater than 3<br>times the upper limit of normal, or terminal cancer or other condition that limited life expectancy to less than 1 year | | 1534 |
| **Method of recruitment of participants** | In person. All patients who came to the general medicine clinic for management of their hypertension during the 1-month enrolment period were identified as potential candidates for the study | | 1534 |
| **Informed consent obtained** | Yes | The nature of the study was described to the patients, and informed consent forms were signed by those eligible and interested in participating. | 1534 |
| **Total no. randomised** *(or total pop. at start of study for NRCTs)* | 56 | | 1534 |
| **Clusters** | None reported | | NA |
| **Baseline imbalances** | Patients in the intervention group had higher mean diastolic blood pressures at baseline than those of patients in the control group (p=0.0012; Table 2). No other differences in baseline characteristics between groups were detected. | | 1536 (Table 1, 2) |

| | | |
|---|---|---|
| **Withdrawals and exclusions** | One patient in the intervention group withdrew because he could not comply with monthly visits to the pharmacist clinic.<br><br>One patient in the control group withdrew from the study because he relocated to a different state. Another patient in the control group withdrew because he no longer was interested in participating in the study | 1536 |
| **Age** | Intervention: 64.0 ± 10.9<br>Comparator: 65.5 ± 7.8 | 1536 (Table 1) |
| **Sex (female)** | Intervention: 0%<br>Comparator: 0% | 1536 (Table 1) |
| **Subgroups measure** | For patients with diabetes, the goal was below 130/80 mm Hg | 1535 |
| **Subgroups reported** | Patients with diabetes | 1535 |
| **Notes** | | |

## Intervention group

| | Description as stated in report/paper | Page number |
|---|---|---|
| **Group name** | Pharmacist prescribing | 1534 |
| **No. randomised/assigned to group** *(specify whether no. people or clusters)* | 27 | 1534 |
| **Description** *(include sufficient detail for replication, e.g. content, dose, components)* | Patients in the intervention group were scheduled to see the clinical pharmacist once/month at the pharmacist managed hypertension clinic. This pharmacist, who had prescribing authority at the medical center, made appropriate drug therapy changes (in both drug selection and dosage) for blood pressure control in accordance with JNC VI.2 The pharmacist did not make any changes in their patients' other drugs that may adversely affect blood pressure… | 1534-1535 |
| **Duration of treatment period** | 6 months | 1534 |
| **Timing** | Once/month | 1534 |
| **Co-interventions** | Drug counselling, consisting of a thorough discussion about side effects, recommended lifestyle changes, and an assessment of compliance, was provided at each visit. Patients were allowed to receive care for comorbid conditions from their primary care providers during the study period, but these providers could not make changes in the patients' antihypertensive drug regimens. | 1535 |
| **Notes** | | |

## Comparator group

| | Description as stated in report/paper | Page number |
|---|---|---|
| **Group name** | Physician prescribing | 1534 |
| **No. randomised/assigned to group** *(specify whether no. people or clusters)* | 29 | 1534 |
| **Description** *(include sufficient detail for replication, e.g. content, dose, components)* | Patients in the control group received traditional pharmacy services (dispensing, brief counselling about drugs, and review of drug profiles) but did not make monthly visits to the pharmacist-managed hypertension clinic. They received care from their primary care providers as needed, at least once/year. All primary care providers were informed that their patients were enrolled in the study. | 1535 |
| **Duration of treatment period** | 6 months | |
| **Timing** | As needed, at least once/year | 1535 |
| **Co-interventions** | None reported. Traditional pharmacy services | NA |
| **Notes** | | |

## Outcomes

### Systolic blood pressure

| | Description as stated in report/paper | | Page number |
|---|---|---|---|
| **Outcome name** | Changes in blood pressure (systolic and diastolic) | | 1535 |
| **Time points measured** *(specify whether from start or end of intervention)* | Baseline, 6 months | | 1535 |
| **Time points reported** | Baseline, 6 months | | 1535 |
| **Outcome definition** *(with diagnostic criteria if relevant)* | Measurements were obtained by a clinical pharmacist using an auscultatory sphygmomanometer. | | 1535 |
| **Unit of measurement** *(if relevant)* | mm Hg | | 1535 |
| **Scales: upper and lower limits** *(indicate whether high or low score is good)* | The general goal was a blood pressure measurement below 140/90 mm Hg.<br>For patients with diabetes, the goal was below 130/80 mm Hg. | | 1535 |
| **Is outcome/tool validated?** | No | | 1535 |

| | | |
|---|---|---|
| **Imputation of missing data** *(e.g. assumptions made for ITT analysis)* | Not reported | NA |
| **Assumed risk estimate** *(e.g. baseline or population risk noted in Background)* | Not reported | NA |
| **Power** *(e.g. power & sample size calculation, level of power achieved)* | Not reported. 'This study had inadequate power to detect a significant difference in compliance, health perception, and patient satisfaction.' | 1539 |
| **Notes** | | |

## Diastolic blood pressure

| | Description as stated in report/paper | | Page number |
|---|---|---|---|
| **Outcome name** | Changes in blood pressure (systolic and diastolic) | | 1535 |
| **Time points measured** *(specify whether from start or end of intervention)* | Baseline, 6 months | | 1535 |
| **Time points reported** | Baseline, 6 months | | 1535 |
| **Outcome definition** *(with diagnostic criteria if relevant)* | Measurements were obtained by a clinical pharmacist using an auscultatory sphygmomanometer. | | 1535 |
| **Unit of measurement** *(if relevant)* | mm Hg | | 1535 |
| **Scales: upper and lower limits** *(indicate whether high or low score is good)* | The general goal was a blood pressure measurement below 140/90 mm Hg.<br>For patients with diabetes, the goal was below 130/80 mm Hg. | | 1535 |
| **Is outcome/tool validated?** | No | | 1535 |
| **Imputation of missing data** *(e.g. assumptions made for ITT analysis)* | Not reported | | NA |
| **Assumed risk estimate** *(e.g. baseline or population risk noted in Background)* | Not reported | | NA |
| **Power** *(e.g. power & sample size calculation, level of power achieved)* | Not reported. 'This study had inadequate power to detect a significant difference in compliance, health perception, and patient satisfaction.' | | 1539 |

| Notes | |
|---|---|

## Compliance

| | Description as stated in report/paper | | Page number |
|---|---|---|---|
| **Outcome name** | Compliance | | 1535 |
| **Time points measured** *(specify whether from start or end of intervention)* | Intervention group: monthly<br>Control group: at baseline and 6 months | | 1535 |
| **Time points reported** | Baseline, 6 months | | 1535 |
| **Outcome definition** *(with diagnostic criteria if relevant)* | In addition to the compliance evaluation filled out by the patient, records of drug refills were obtained from the pharmacy. Noncompliance was defined as missing more than three doses of drug in 1 week, or having pharmacy records indicate failure to refill drugs within 2 weeks after the scheduled refill date. | | 1535 |
| **Unit of measurement** *(if relevant)* | Reponses to 6 survey questions: 4 coded as percentage reporting ≥once/week; 2 as percentage yes<br>Percentage receiving refills for antihypertensive agents within 2 weeks of the next scheduled refill date | | 1537, Table 5 |
| **Scales: upper and lower limits** *(indicate whether high or low score is good)* | Unclear. | | NA |
| **Is outcome/tool validated?** | No | Compliance evaluation self-reported survey; pharmacy records | |
| **Imputation of missing data** *(e.g. assumptions made for ITT analysis)* | Not reported | | NA |
| **Assumed risk estimate** *(e.g. baseline or population risk noted in Background)* | Not reported | | NA |
| **Power** *(e.g. power & sample size calculation, level of power achieved)* | Not reported. 'This study had inadequate power to detect a significant difference in compliance, health perception, and patient satisfaction.' | | 1539 |
| **Notes** | Not clear if they had access to the pharmacy records for the controls as well | | |

## Quality of life

| | Description as stated in report/paper | Page number |
|---|---|---|
| **Outcome name** | Quality of life | 1536 |

| | | | |
|---|---|---|---|
| **Time points measured** *(specify whether from start or end of intervention)* | Baseline, 6 months | | 1535 |
| **Time points reported** | Baseline, 6 months | | 1535 |
| **Outcome definition** *(with diagnostic criteria if relevant)* | Noncompliance was defined as missing more than three doses of drug in 1 week, or having pharmacy records indicate failure to refill drugs within 2 weeks after the scheduled refill date. | | 1535 |
| **Unit of measurement** *(if relevant)* | Unclear. 'Categorical data, such as compliance and quality of life, were analyzed with the Pearson x2 test.'<br>'Scores range from 0-100' | | 1535, 1538 |
| **Scales: upper and lower limits** *(indicate whether high or low score is good)* | Unclear. | | NA |
| **Is outcome/tool validated?** | Yes | Medical Outcomes Study Short Form-36 health survey | 1535 |
| **Imputation of missing data** *(e.g. assumptions made for ITT analysis)* | Not reported | | NA |
| **Assumed risk estimate** *(e.g. baseline or population risk noted in Background)* | Not reported | | NA |
| **Power** *(e.g. power & sample size calculation, level of power achieved)* | Not reported. 'This study had inadequate power to detect a significant difference in compliance, health perception, and patient satisfaction.' | | 1539 |
| **Notes** | | | |

## Funding/conflict of interest

| | | |
|---|---|---|
| **Study funding sources** *(including role of funders)* | Supported by the Christian R. and Mary F. Lindback Foundation. | 1535 |
| **Possible conflicts of interest** *(for study authors)* | Not reported. | |
| **Notes** | | |

## Data and analysis

### Systolic blood pressure

<table>
<tr><th></th><th colspan="6">Description as stated in report/paper</th><th>Page number</th></tr>
<tr><td>Outcome</td><td colspan="6">Changes in blood pressure (systolic and diastolic)</td><td>1535</td></tr>
<tr><td>Time point (specify from start or end of intervention)</td><td colspan="6">Baseline, 6 months</td><td>1535</td></tr>
<tr><td rowspan="3">Results: Systolic (end of study)</td><td colspan="3">Intervention</td><td colspan="3">Comparison</td><td rowspan="3">1536, Table 2</td></tr>
<tr><td>Mean</td><td>SD</td><td>No. participants</td><td>Mean</td><td>SD</td><td>No. participants</td></tr>
<tr><td>130.5</td><td>13.2</td><td>26</td><td>148.4</td><td>21.0</td><td>27</td></tr>
<tr><td>Any other results reported (e.g. mean difference, CI, P value)</td><td colspan="6">Of the 26 patients in the intervention group, 21 (81%) attained a blood pressure below 140/90 mm Hg at the end of the study versus only eight (30%) of the 27 patients in the control group (p=0.001).<br><br>Systolic blood pressure was significantly lower in intervention compared with comparator groups (p=0.0002).<br><br>Mean changes in systolic blood pressure from baseline for the intervention and control groups were -18.4 (95% confidence interval [CI] -26.3, -10.5) and -3.98 (95% CI -11.8, 3.79), respectively (p=0.01).<br><br>Of the 11 patients with diabetes in the intervention group, 10 (91%) attained the goal blood pressure of below 130/80 mm Hg versus only two (12 %) of 16 patients with diabetes in the control group (p=0.001; Table 3).</td><td>1536, Table 2, Table 3</td></tr>
<tr><td>No. missing participants</td><td colspan="3">Intervention: 1</td><td colspan="3">Comparator: 2</td><td>1535-1536</td></tr>
<tr><td>Statistical methods used and appropriateness of these (e.g. adjustment for correlation)</td><td colspan="6">The paired t test was used to measure differences in blood pressure between baseline and end of study. A two-sample t test was used to assess the significance of the treatment difference between the mean systolic and diastolic blood pressure change from baseline to end of study. This method of analysis assumes that This method of analysis assumes that the data are distributed symmetrically. We checked for significant violations of this assumption and validated the results with a Wilcoxon sum rank test, which requires no parametric assumption.<br>Fisher's exact test was used to compare the number of patients reaching their blood pressure goal in each group, as well as the number of patients with diabetes who reached their goal.</td><td>1535</td></tr>
<tr><td>Notes</td><td colspan="7"></td></tr>
</table>

## Diastolic blood pressure

| | Description as stated in report/paper | Page number |
|---|---|---|
| **Outcome** | Changes in blood pressure (systolic and diastolic) | 1535 |

| | | | | | | | |
|---|---|---|---|---|---|---|---|
| **Time point** *(specify from start or end of intervention)* | Baseline, 6 months | | | | | | 1535 |
| **Results: Diastolic (end of study)** | Intervention | | | Comparison | | | 1536, Table 2 |
| | Mean | SD | No. participants | Mean | SD | No. participants | |
| | 77.5 | 10.7 | 26 | 80.4 | 11.4 | 27 | |
| **Any other results reported** *(e.g. mean difference, CI, P value)* | Of the 26 patients in the intervention group, 21 (81%) attained a blood pressure below 140/90 mm Hg at the end of the study versus only eight (30%) of the 27 patients in the control group (p=0.001).<br><br>Mean changes in diastolic blood pressure from baseline for the intervention and control groups were -12.38 (95% CI -16.49, -8.28) and 2.54 (95% CI -1.49, 6.57), respectively (p=0.001).<br><br>Of the 11 patients with diabetes in the intervention group, 10 (91%) attained the goal blood pressure of below 130/80 mm Hg versus only two (12 %) of 16 patients with diabetes in the control group (p=0.001; Table 3). | | | | | | 1536, Table 2, Table 3 |
| **No. missing participants** | Intervention: 1 | | | Comparator: 2 | | | 1535-1536 |
| **Statistical methods used and appropriateness of these** *(e.g. adjustment for correlation)* | The paired t test was used to measure differences in blood pressure between baseline and end of study. A two-sample t test was used to assess the significance of the treatment difference between the mean systolic and diastolic blood pressure change from baseline to end of study. This method of analysis assumes that This method of analysis assumes that the data are distributed symmetrically. We checked for significant violations of this assumption and validated the results with a Wilcoxon sum rank test, which requires no parametric assumption. Fisher's exact test was used to compare the number of patients reaching their blood pressure goal in each group, as well as the number of patients with diabetes who reached their goal. | | | | | | 1535 |
| **Notes** | | | | | | | |

## Compliance

| | Description as stated in report/paper | | Page number |
|---|---|---|---|
| **Outcome** | Compliance | | 1535 |
| **Time point** *(specify from start or end of intervention)* | Intervention: monthly<br>Comparator: baseline, 6 months | | 1535, 1537 |
| **Results** | Intervention | Comparison | 1537, Table 5 |

| | | | | | |
|---|---|---|---|---|---|
| | No. with event | Total in group | No. with event | Total in group | |
| Forgets ≥1x/wk | 18 | 26 | 13 | 27 | |
| Stops when better ≥1x/wk | 8 | 26 | 5 | 27 | |
| Stops when worse ≥1x/wk | 10 | 26 | 5 | 27 | |
| Take more than prescribed ≥1x/wk | 2 | 26 | 2 | 27 | |
| Away overnight & forgets meds | 2 | 26 | 1 | 27 | |
| Refills on time | 22 | 26 | 25 | 27 | |
| **Any other results reported** | No significant difference in compliance was reported between (p>0.25) or within (p>0.07) the two groups at baseline or end of study (Table 5).<br><br>Pharmacy records indicated that 22 (85%) of the 26 patients from the intervention group received their refills for antihypertensive agents within 2 weeks of the next scheduled refill date, versus 25 (93%) of the 27 patients from the control group (p>0.42). | | | | **≥1x/wk** |
| **No. missing participants** | Intervention: 1 | | Comparator: 2 | | 1535-1536 |
| **Reasons missing** | Could not comply with monthly visits | | Moved, withdrew as no longer interested | | 1535-1536 |
| **Statistical methods used and appropriateness of these** | Categorical data, such as compliance and quality of life, were analysed with the Pearson x2 test. Because of the low cell sizes in the contingency tables for the patient satisfaction data, the Fisher's exact test was used. This procedure gives exact results for 2 x 2 tables in which the standard x2 is not applicable. | | | | |
| **Notes** | None | | | | |

## SF-36 (continuous)

| | **Description as stated in report/paper** | | | | | | **Page number** |
|---|---|---|---|---|---|---|---|
| **Outcome** | Quality of life | | | | | | 1535 |
| **Time point** *(specify from start or end of intervention)* | Baseline, 6 months | | | | | | 1535 |
| **Results** | Intervention | | | Comparison | | | 1538, Table 7 |
| | Mean | SD | No. participants | Mean | SD | No. participants | |

| | | | | | | | |
|---|---|---|---|---|---|---|---|
| **Health perception** | 41.98 | 14.35 | 26 | 41.8 | 15.74 | 27 | |
| **Physical functioning** | 65.97 | 27.7 | 26 | 63.04 | 28.90 | 27 | |
| **Role limitations, physical** | 50.0 | 45.64 | 26 | 56.52 | 43.44 | 27 | |
| **Role limitations, emotional** | 44.0 | 46.34 | 26 | 56.52 | 48.39 | 27 | |
| **Social functioning** | 63.33 | 33.71 | 26 | 67.5 | 37.21 | 27 | |
| **Energy, fatigue** | 59.87 | 18.01 | 26 | 68.2 | 18.53 | 27 | |
| **Bodily pain** | 58.67 | 27.87 | 26 | 70.67 | 29.20 | 27 | |

| | | | |
|---|---|---|---|
| **Any other results reported** *(e.g. mean difference, CI, P value)* | p-values intervention vs. comparator<br>• Health perception: p=0.9670<br>• Physical functioning: p=0.7243<br>• Role limitations, physical: p=0.6152<br>• Role limitations, emotional: p=0.364<br>• Social functioning: p=0.6862<br>• Energy, fatigue: p=0.1141<br>• Bodily pain: p=0.1437<br><br>On the quality-of-life assessment, no statistically significant differences were noted between the two groups from baseline to end of study (Table 7). However, patients in the control group reported higher scores for bodily pain at baseline than patients in the intervention group (p<0.04). No significant difference was reported<br>within the two groups at baseline or end of study (p>0.2) | | 1538 |
| **No. missing participants** | Intervention: 1 | Comparator: 2 | 1535-1536 |
| **Statistical methods used and appropriateness of these** *(e.g. adjustment for correlation)* | With the quality-of-life scores on the Short Form-36 health survey, comparisons between the intervention and control groups were evaluated by two-sample t tests at baseline and after intervention, and the difference between baseline and end of study was calculated. These results were validated with a nonparametric Wilcoxon rank sum test. | | 1535 |
| **Notes** | | | |

## Conclusions

| | **Description as stated in report/paper** | **Page number** |
|---|---|---|

| | | |
|---|---|---|
| **Key conclusions of study authors** | This study demonstrates that pharmaceutical care provided by a pharmacist managed hypertension clinic improves blood pressure control and results in more patients with hypertension reaching their goal blood pressure measurement. | 1539 |
| **Notes** | | |

# Weaver et al. 2023

## Study eligibility

| **Study Characteristics** | **Eligibility criteria** |
|---|---|
| **Title** | Evaluation of a pharmacist-managed anemia service in pregnant patients |
| **Author (year)** | Weaver et al. (2023) |
| **Country** | California, USA |
| **Type of study** | Retrospective cohort |
| **Participants** | Pregnant patients with iron-deficiency anemia |
| **Types of intervention** | Pharmacist managed |
| **Types of comparison** | Standard care (OBGYN) |
| **Types of outcome measures** | Haemoglobin levels (percentage achieving target and mean levels) |
| **Prescriptive authority** | Protocol |
| **Include/Exclude** | Include |
| **Notes** | None |

**DO NOT PROCEED IF STUDY EXCLUDED FROM REVIEW**

## Characteristics of included studies

### Methods

| | Descriptions as stated in report/paper | | Page number |
|---|---|---|---|
| **Aim of study** | To evaluate the impact of a pharmacist-led anemia management service in pregnant patients with IDA. | | S22 |
| **Design** | Retrospective cohort study | | S22 |
| **Unit of allocation** *(by individuals, cluster/ groups or body parts)* | Individuals | | S22 |
| **Start-end date** | June 2020 – December 2021 | | S22 |
| **Duration of participation** *(from recruitment to last follow-up/ baseline to last follow-up- group level)* *1. Time of consent until last measurement for each individual.* *2. Baseline to final follow-up for each individual* | June 2020 – December 2021 | | S22 |
| **Study duration** *(as above with the exception of interim analyses or other circumstances* | June 2020 – December 2021 | | S22 |
| **Ethical approval needed/obtained for study** | Exempt | This study was formally reviewed and received institutional review board exemption approval | S22 |
| **Notes** | None | | |

### Participants

| | **Description** *Include comparative information for each intervention or comparison group if available* | Page number |
|---|---|---|

| | | | |
|---|---|---|---|
| **Population description** *(from which study participants are drawn)* | Pregnant patients with iron-deficiency anaemia managed by a pharmacist | | S22 |
| **Setting** *(including location and social context)* | A telephone-based ambulatory care pharmacy clinic performed the duties of the pharmacist-managed anemia service and followed protocols developed in collaboration with the OBGYN team to optimize care. | | S22 |
| **Inclusion/exclusion criteria** | Patients were included if they were women 16 years of age or older, were pregnant during the study period, and had IDA (Hgb concentration of ≤11.0 g/dL and ferritin concentration of <40 µg/L) during pregnancy. Once the report was obtained, the list of patients was randomized using a random number generator and the charts were reviewed in numerical order. The lead study investigator screened the medical records of the randomized patients for appropriateness based on the study criteria. Patients were excluded if they had anemia due to any cause other than iron deficiency or had a miscarriage or abortion during the study period; if their insurance coverage terminated during the study period; or if "noncompliant," "noncompliance," "nonadherent," or "nonadherence" was documented in their electronic medical record during the study period; if they were discharged from the pharmacist-managed service for any reason other than reaching Hgb goal; and/or if they delivered after November 29, 2021. | | S23 |
| **Method of recruitment of participants** | Patient chart review<br><br>Patients included in the pharmacist managed group were individually matched 1:1 with patients in the cohort receiving standard care. | | S23 |
| **Informed consent obtained** | NA (exempt) | NA | NA |
| **Total no. randomised** *(or total pop. at start of study for NRCTs)* | 200 individuals | | S23 |
| **Clusters** | Not reported | | NA |
| **Baseline imbalances** | Cohort matched on age, gestational age, and risk level for postpartum hemorrhage as assessed at the time of delivery (high, medium, or low risk). | | S23 |

| | | |
|---|---|---|
| **Withdrawals and exclusions** | A total of 420 patients were reviewed, with 164 patients in the pharmacist-managed group and 256 patients in the standard care group. After review, a total of 100 patients were included in each group. Patients were excluded due to not meeting matching criteria, nonadherence, a ferritin level of >40 µg/L, discharge from the pharmacy-managed service, and/or missing documentation. | S23 |
| **Age** | Intervention: 35.0 ± 4<br>Comparator: 35.0 ± 4 | S24 (table 1) |
| **Sex (female)** | Intervention: 100%<br>Comparator: 100% | NA |
| **Subgroups measure** | Not reported | NA |
| **Subgroups reported** | Notreported | NA |
| **Notes** | None | |

## Intervention group

| | **Description as stated in report/paper** | **Page number** |
|---|---|---|
| **Group name** | Pharmacist managed | S22 |
| **No. randomised/assigned to group** *(specify whether no. people or clusters)* | 100 individuals | S22 |

| | | |
|---|---|---|
| **Description** *(include sufficient detail for replication, e.g. content, dose, components)* | A telephone-based ambulatory care pharmacy clinic performed the duties of the pharmacist-managed anemia service and followed protocols developed in collaboration with the OBGYN team to optimize care.<br><br>Under protocol, pharmacists were authorized to initiate and adjust iron therapies, order baseline and follow-up laboratory tests, counsel patients, and assess the efficacy, safety, and tolerability of the iron regimens selected. Patients referred to the pharmacist managed service by their OBGYN providers were contacted by the pharmacy service and started on iron supplementation. After the initial consultation, pharmacists checked in with the patient in 2 weeks to assess tolerability and adherence with their iron regimen. On the basis of the patient's tolerability, iron therapy was either continued or switched to an alternative therapy. Patient laboratory tests were then repeated at 4-week intervals. Patients with Hgb levels above goal were continued on the same iron regimen and discharged from the pharmacy service.<br>Patients with Hgb levels below goal had their oral iron regimen titrated or switched to IV iron infusion per the pharmacist protocol. | S22 |
| **Duration of treatment period** | No set treatment period. Treatment continued until delivery. | NA |
| **Timing** | After the initial consultation, pharmacists checked in with the patient in 2 weeks to assess tolerability and adherence with their iron regimen. On the basis of the patient's tolerability, iron therapy was either continued or switched to an alternative therapy. Patient laboratory tests were then repeated at 4-week intervals. | S22 |
| **Co-interventions** | Patient counselling, ordering tests | S22 |
| **Notes** | None | |

## Comparator group

| | **Description as stated in report/paper** | **Page number** |
|---|---|---|
| **Group name** | Standard care | S22 |
| **No. randomised/assigned to group** *(specify whether no. people or clusters)* | 100 individuals | S22 |

| | | |
|---|---|---|
| **Description** *(include sufficient detail for replication, e.g. content, dose, components)* | Pregnant patients with IDA receiving standard care from the obstetrics and gynecology (OBGYN) team at the healthcare system's site in Santa Clara, | S22 |
| **Duration of treatment period** | No set treatment period. Treatment continued until delivery. | NA |
| **Timing** | Not reported | NA |
| **Co-interventions** | Not reported | NA |
| **Notes** | None | |

## Outcomes

### Percentage of patients achieving a target Hgb level

| | **Description as stated in report/paper** | | **Page number** |
|---|---|---|---|
| **Outcome name** | Percentage of patients achieving a target Hgb level of >11.0 g/dL at delivery | | S23 |
| **Time points measured** *(specify whether from start or end of intervention)* | At delivery | | S23 |
| **Time points reported** | At delivery | | S23 |
| **Outcome definition** *(with diagnostic criteria if relevant)* | Hgb level of >11.0 g/dL at delivery | | S23 |
| **Unit of measurement** *(if relevant)* | Event data | | S23 |
| **Scales: upper and lower limits** *(indicate whether high or low score is good)* | Hgb level of >11.0 g/dL at delivery | | S23 |
| **Is outcome/tool validated?** | No | Bloodwork | S23 |
| **Imputation of missing data** *(e.g. assumptions made for ITT analysis)* | Not reported | | NA |
| **Assumed risk estimate** *(e.g. baseline or population risk noted in Background)* | Not reported | | NA |
| **Power** *(e.g. power & sample size calculation, level of power achieved)* | Not reported | | NA |
| **Notes** | None | | |

## Mean Hgb level

| | Description as stated in report/paper | | Page number |
|---|---|---|---|
| **Outcome name** | Hgb level at delivery | | S23 |
| **Time points measured** *(specify whether from start or end of intervention)* | At delivery | | S23 |
| **Time points reported** | At delivery | | S23 |
| **Outcome definition** *(with diagnostic criteria if relevant)* | Mean Hgb level at delivery | | S23 |
| **Unit of measurement** *(if relevant)* | Continuous g/dL | | S23 |
| **Scales: upper and lower limits** *(indicate whether high or low score is good)* | Hgb level at delivery | | S23 |
| **Is outcome/tool validated?** | No | Bloodwork | S23 |
| **Imputation of missing data** *(e.g. assumptions made for ITT analysis)* | Not reported | | NA |
| **Assumed risk estimate** *(e.g. baseline or population risk noted in Background)* | Not reported | | NA |
| **Power** *(e.g. power & sample size calculation, level of power achieved)* | Not reported | | NA |
| **Notes** | None | | |

## Funding/conflict of interest

| | | |
|---|---|---|
| **Study funding sources** *(including role of funders)* | Not reported | NA |
| **Possible conflicts of interest** *(for study authors)* | The authors have declared no potential conflicts of interest. | S27 |
| **Notes** | None | |

## Data and analysis

### Percentage of patients achieving a target Hgb level

| | Description as stated in report/paper | | | | Page number |
|---|---|---|---|---|---|
| **Outcome** | Percentage of patients achieving a target Hgb level | | | | S23 |
| **Time point** *(specify from start or end of intervention)* | At delivery | | | | S23 |
| **Results** | Intervention | | Comparison | | S23 |
| | No. with event | Total in group | No. with event | Total in group | |
| | 87 | 100 | 71 | 100 | |
| **Any other results reported** | The primary outcome of the percentage of patients achieving a target Hgb level of >11.0 g/dL at delivery was significantly higher in the pharmacist managed group than in the group receiving standard care (87% vs 71%; *P* < 0.01) | | | | S23 |
| **No. missing participants** | NA | | NA | | NA |
| **Reasons missing** | NA | | NA | | NA |
| **Statistical methods used and appropriateness of these** | $\chi 2$ tests were used to analyze the primary outcome of the percentage of patients achieving target Hgb levels at labor and delivery and the secondary outcomes of patients receiving blood transfusions, the percentage of patients receiving IV iron infusions, and patient demographics. Paired *t* tests were used to assess the secondary outcomes of mean Hgb level at delivery and patient demographics. | | | | S23 |
| **Confounders** | Matched cohort | | | | S23 |
| **Notes** | None | | | | |

## HgB level

| | Description as stated in report/paper | | | | | | Page number |
|---|---|---|---|---|---|---|---|
| **Outcome** | HgB level | | | | | | |
| **Time point** *(specify from start or end of intervention)* | At delivery | | | | | | S23 |
| **Results** | Intervention | | | Comparison | | | S23 |
| | Mean | SD *(or other variance, specify)* | No. participants | Mean | SD *(or other variance, specify)* | No. participants | |
| | 12.1 G/Dl | 1.0 | 100 | 11.6 | 1.1 | 100 | |
| **Any other results reported** *(e.g. mean difference, CI, P value)* | The secondary outcome of mean (SD) Hgb level at delivery was significantly higher in the pharmacist-managed group than in the group receiving standard care at 12.1 (1.0) g/dL vs 11.6 (1.1) g/dL, respectively (*P* < 0.01). | | | | | | S23 |

| | | | |
|---|---|---|---|
| **No. missing participants** | NA | NA | NA |
| **Statistical methods used and appropriateness of these** *(e.g. adjustment for correlation)* | $\chi 2$ tests were used to analyse the primary outcome of the percentage of patients achieving target Hgb levels at labour and delivery and the secondary outcomes of patients receiving blood transfusions, the percentage of patients receiving IV iron infusions, and patient demographics. Paired *t* tests were used to assess the secondary outcomes of mean Hgb level at delivery and patient demographics. | | S23 |
| **Confounders** | Matched cohort | | S23 |
| **Notes** | None | | |

## Conclusions

| | Description as stated in report/paper | Page number |
|---|---|---|
| **Key conclusions of study authors** | Addition of a pharmacist-managed service for IDA in pregnancy to standard care significantly increases the number of pregnant patients who achieve target Hgb levels at delivery. | S27 |
| **Notes** | None | |

# Xu et al. 2020

## Study eligibility

| Study Characteristics | Eligibility criteria |
|---|---|
| **Title** | Impact of team-based pharmaceutical care on the humanistic outcomes among patients with long-standing diabetes: An interim analysis of a randomized, controlled, multicenter study |
| **Author (year)** | Xu et al. (2020) |
| **Country** | Singapore |
| **Type of study** | Open-label randomised controlled study |
| **Participants** | Adults ≥21 years with type 2 diabetes mellitus (T2DM) for at least 5 years which was uncontrolled (A1C >7%) and had polypharmacy (at least 5 chronic prescriptions) |
| **Types of intervention** | Team-based pharmaceutical care (physicians, nurses, dieticians, and clinical pharmacists) |
| **Types of comparison** | Physician care with referral to nurses and dieticians as needed |

| | |
|---|---|
| **Types of outcome measures** | HbA1c (glycemic control); diabetes-specific quality of life; work productivity (quality of life) |
| **Prescriptive authority** | Collaborative |
| **Include/Exclude** | Include |
| **Notes** | |

**DO NOT PROCEED IF STUDY EXCLUDED FROM REVIEW**

## Characteristics of included studies

### Methods

| | Descriptions as stated in report/paper | | Page number |
|---|---|---|---|
| **Aim of study** | Aim: To evaluate the impact of team-based pharmaceutical care on clinical, humanistic, and economic outcomes.<br><br>Primary objective: To examine the impact of pharmaceutical care on glycemia and self-care in patients with long-standing diabetes.<br><br>Secondary objectives: To compare diabetes-dependent quality of life, diabetes distress, and productivity loss between pharmaceutical care and usual care. | | 681 |
| **Design** | Open-label randomised controlled study | | 681 |
| **Unit of allocation** *(by individuals, cluster/ groups or body parts)* | Individuals | | 681 |
| **Start-end date** | April 2018 – August 2019 | | 681 |
| **Duration of participation** *(from recruitment to last follow-up)* | 6-months | | 681 |
| **Study duration** | 12-months (This study was part of a bigger ongoing study where 125 participants were required in each arm to evaluate the effectiveness of pharmaceutical-based care for 12 months (ClinicalTrial ID: NCT03553862) | | 683 |
| **Ethical approval needed/obtained for study** | Yes | The study was approved by the National Healthcare Group Domain Specific Review Board (2017/01191) and registered at ClinicalTrial. gov (ID: NCT03553862). | 681 |
| **Notes** | | | |

### Participants

| | **Description**<br>*Include comparative information for each intervention or comparison group if available* | | **Page number** |
|---|---|---|---|
| **Population description**<br>*(from which study participants are drawn)* | Patients aged ≥21 years with a T2DM diagnosis of at least 5 years with current uncontrolled T2DM and reporting polypharmacy | | 681 |
| **Setting**<br>*(including location and social context)* | Two outpatient primary health institutions in Singapore | | 681, 682 |
| **Inclusion/exclusion criteria** | Inclusion: Patients aged ≥21 years with uncontrolled T2DM (defined as A1C >7%), reporting polypharmacy (defined as taking =5 chronic prescription medications) and identified to be diabetes-experienced (defined as those with ≥5 years of documented diabetes history) were included in the study.<br><br>Exclusion: Patients who had type 1 diabetes or were unable to communicate independently were excluded. | | 681 |
| **Method of recruitment of participants** | In-person | | 681 |
| **Informed consent obtained** | Yes | Upon agreeing to partake in the study by signing the informed consent form, participants were randomized into either the control arm or the intervention arm. | 682 |
| **Total no. randomised**<br>*(or total pop. at start of study for NRCTs)* | 248 | | 683 (Figure 1) |
| **Clusters** | Study site was set a random effect to account for the clustering of groups over time | | 684 |
| **Baseline imbalances** | Table 1. Everything looks comparable except perhaps slightly more Chinese (67% vs. 59%) males (69 vs. 61%) in the intervention than control | | 684 |

| | | |
|---|---|---|
| **Withdrawals and exclusions** | Intervention: loss to follow up n = 16<br>Comparator: loss to follow up n = 20<br>The baseline characteristics were comparable between those who completed the study and those who dropped out (Appendix Table S1). | 683 (Figure 1), 684 |
| **Age** | Intervention: 59.7 ± 7.3<br>Comparator: 59.9 ± 6.8 | 684 (Table 1) |
| **Sex (female)** | Intervention: 69%<br>Comparator: 75 61% | 684 (Table 1) |
| **Subgroups measure** | NA | |
| **Subgroups reported** | NA | |
| **Notes** | None | |

## Intervention group

| | **Description as stated in report/paper** | **Page number** |
|---|---|---|
| **Group name** | Team-based pharmaceutical care | 682 |
| **No. randomised/assigned to group** *(specify whether no. people or clusters)* | 126 | 683 (Figure 1) |

| | | |
|---|---|---|
| **Description** *(include sufficient detail for replication, e.g. content, dose, components)* | The intervention arm consisted of team-based pharmaceutical care which included physicians, nurses, dietitians, and trained clinical pharmacists.<br>Specifically, clinical pharmacists followed up with patients every 4 to 6 weeks as appropriate. The frequency of contacts with the clinical pharmacist was determined by the care team based on the needs of the participants.<br><br>The clinical pharmacists were affiliated with pharmacies, performing hybrid activities which included dispensing and order-process as well as clinical activities in the clinic together with the primary care team…<br><br>On clinic days, clinical pharmacists provided comprehensive medication management to patients regularly. During each face-to-face clinic consultation, a clinical pharmacist would provide medication optimization through medication review, dose adjustments, and insulin initiation and titration, as well as switching of drugs. The clinical pharmacists worked under a collaborative agreement and furnished prescriptions on behalf of the medical physicians.<br><br>In addition to comprehensive medication management, each consultation session conducted by clinical pharmacists also included counselling on education regarding lifestyle (healthy diet and physical activity), self-monitoring of blood glucose, avoidance of diabetes-related complications such as infection to soft tissues, correct administration of insulin, and prevention of adverse reactions.<br><br>The care process was based on an established protocol published by the American College of Clinical Pharmacy where detailed clinical activities are documented.30 Furthermore, clinical pharmacists also updated patients' medical records and prescriptions in the system, providing a streamlined workflow to improve inter-professional collaboration.<br><br>Visits to nurses and dietitians were scheduled on an as needed basis by physicians or pharmacists when the patient required an eye screening, foot screening, or nutritional therapy counselling | 682 |

| | | |
|---|---|---|
| **Duration of treatment period** | 6 months | 681, 683 |
| **Timing** | Every 4 to 6 weeks as appropriate | 682 |
| **Co-interventions** | Comprehensive medication management (medication review, dose adjustments, insulin initiation, switching of drugs, furnished prescriptions) and counseling on education regarding lifestyle, self-monitoring of blood glucose, avoidance of diabetes-related complications, correct administration of insulin, and prevention of adverse events | 682 |
| **Notes** | | |

## Comparator group

| | Description as stated in report/paper | Page number |
|---|---|---|
| **Group name** | Usual care (with physician) | 682 |
| **No. randomised/assigned to group** *(specify whether no. people or clusters)* | 122 | 683 (Figure 1) |
| **Description** *(include sufficient detail for replication, e.g. content, dose, components)* | Patients in the control arm received usual care, which included 10-minute regular follow-up visits with physicians and were subjected to referral to the nurses or dietitians as needed. Patients in the control arm had no contact with clinical pharmacists except when collecting medications from the pharmacy. | 682 |
| **Duration of treatment period** | 6 months | 681, 683 |
| **Timing** | Unclear frequency. 10-minute regular follow-up visits with physicians | 682 |
| **Co-interventions** | Were subjected to referral to the nurses and dieticians as needed | 682 |
| **Notes** | | |

## Outcomes

### HbA1c (glycemic control)

| | Description as stated in report/paper | Page number |
|---|---|---|
| **Outcome name** | HbA1c (glycemic control) | 682 |
| **Time points measured** *(specify whether from start or end of intervention)* | Baseline and 6 months | 682 |

| | | | |
|---|---|---|---|
| **Time points reported** | Baseline and 6 months | | 682 |
| **Outcome definition** *(with diagnostic criteria if relevant)* | Glycosylated haemoglobin | | 681, 684 |
| **Unit of measurement** *(if relevant)* | Unclear. Stratified at randomization by percentages but standard clinical measurements are in Mmol/mol | | 682 |
| **Scales: upper and lower limits** *(indicate whether high or low score is good)* | 7.1->10% | | 682 |
| **Is outcome/tool validated?** | No | | NA |
| **Imputation of missing data** *(e.g. assumptions made for ITT analysis)* | 20 imputations were generated for each outcome by entering variables of gender, age, ethnicity, diabetes duration, and number of comorbidities. | | 684 |
| **Assumed risk estimate** *(e.g. baseline or population risk noted in Background)* | Not reported | | NA |
| **Power** *(e.g. power & sample size calculation, level of power achieved)* | The sample size was performed based on an effect size of a 0.40 in glycemic control between the intervention and control arms over 12 months. After accounting for a drop-out rate of 20%, approximately 125 people were required in each arm (two-sided, alpha = 5%, power = 80%) | | 683 |
| **Notes** | They didn't have 125 in each arm (control had 122) so power is less than 80 in the end | | |

## Diabetes-specific quality of life

| | Description as stated in report/paper | Page number |
|---|---|---|
| **Outcome name** | Diabetes-specific quality of life | 682 |
| **Time points measured** *(specify whether from start or end of intervention)* | Baseline and 6 months | 682 |
| **Time points reported** | Baseline and 6 months | 685 |
| **Outcome definition** *(with diagnostic criteria if relevant)* | The ADDQOL is a diabetes-specific measure to evaluate the impact of diabetes on various aspects of life and the importance of these aspects to patients.<br>This questionnaire consists of questions in 19 specific life domains | 682 |

| | | | |
|---|---|---|---|
| **Unit of measurement** *(if relevant)* | Average weighted impact (AWI) score (see scoring information below) | | 683 |
| **Scales: upper and lower limits** *(indicate whether high or low score is good)* | Each domain consists of one hypothetical question examining the impact of diabetes on a particular life domain and one question on the importance of that life domain to patients.<br><br>The hypothetical question was rated on a fivepoint Likert scale ranging from−3 (greatest negative impact) to +1 (positive impact).<br><br>The question on importance was rated on a four-point scale ranging from 0 (not at all important) to 3 (very important).<br><br>The two scores in each domain were multiplied together to generate the weighted impact score. The weighted impact score of each domain was summed and then divided by the number of applicable domains to produce the average weighted impact (AWI) score. | | 682-683 |
| **Is outcome/tool validated?** | Yes | Diabetes-Dependent Quality of Life (ADDQOL) | 682 |
| **Imputation of missing data** *(e.g. assumptions made for ITT analysis)* | 20 imputations were generated for each outcome by entering variables of gender, age, ethnicity, diabetes duration, and number of comorbidities. | | 684 |
| **Assumed risk estimate** *(e.g. baseline or population risk noted in Background)* | Not reported | | NA |
| **Power** *(e.g. power & sample size calculation, level of power achieved)* | NA, power calculation based on Hb1Ac | | 683 |
| **Notes** | | | |

## Funding/conflict of interest

| | | |
|---|---|---|
| **Study funding sources** *(including role of funders)* | This study was supported by the Health Services Research Competitive Research Grant from the Ministry of Health Singapore (HSRG14may006) granted to the Principal Investigator Joyce Yu-Chia Lee. | 686 |
| **Possible conflicts of interest** *(for study authors)* | The authors declare no conflicts of interest. | 686 |
| **Notes** | | |

## Data and analysis

### Hb1Ac (glycemic control)

| | Description as stated in report/paper | | | | | | Page number |
|---|---|---|---|---|---|---|---|
| **Outcome** | HbA1c (glycemic control) | | | | | | 682 |
| **Time point** *(specify from start or end of intervention)* | Baseline and 6 months | | | | | | 682 |
| **Results** | Intervention | | | Comparison | | | 684 |
| | Mean | 95% CI *(or other variance, specify)* | No. participants | Mean | 95% CI *(or other variance, specify)* | No. participants | |
| | -0.50 | (0.24, 0.75) | 126 | -0.11 | (-0.20, 0.42) | 122 | |
| **Any other results reported** *(e.g. mean difference, CI, P value)* | A greater average HbA1c reduction was observed in the intervention arm with a difference of 0.44% (P = 0.003, 95% confidence interval (CI): [0.15, 0.73]) at 6 months when compared with the control arm | | | | | | 684 |
| **No. missing participants** | N = 20 missing in intervention<br>N = 16 missing in control arm | | | | | | 684 |
| **Statistical methods used and appropriateness of these** *(e.g. adjustment for correlation)* | Differences in the clinical parameters and patient-reported outcomes between the control arm and the intervention arm were analyzed using linear mixed models from baseline to 6 months. Linear mixed models were utilized by setting time-by-arm interaction terms. Study site was set a random effect to account for the clustering of groups over time, and social demographics were set as covariates.<br><br>Using multiple imputations (13.1% and 15.8% missing in the control arm and in the intervention arm, respectively) and mixed effects models. | | | | | | 684 |
| **Notes** | | | | | | | |

### Diabetes-specific quality of life

| | Description as stated in report/paper | | Page number |
|---|---|---|---|
| **Outcome** | Diabetes-specific quality of life | | 682 |
| **Time point** *(specify from start or end of intervention)* | Baseline and 6 months | | 682 |
| | Intervention | Comparison | 685, 683 |

| **Results (baseline)** | Mean | SD *(or other variance, specify)* | No. participants | Mean | SD *(or other variance, specify)* | No. participants | |
|---|---|---|---|---|---|---|---|
| | -2.92 | 2.02 | 126 | -2.88 | 2.07 | 122 | |
| **Results (six-month follow-up)** | -2.95 | 2.29 | 106 | -3.14 | 2.09 | 106 | 685, 683 |
| **Any other results reported** *(e.g. mean difference, CI, P value)* | At the 6-month follow-up visit, the mean AWI score in the intervention arm did not differ significantly (−2.92 ± 2.02 at baseline and − 2.95 ± 2.29 at 6 months, P = .893, 95% CI: [−0.50, 0.58]), demonstrating that quality of life in this group was maintained. However, a decreasing trend in quality of life was observed in the control arm, with the AWI score decreasing from −2.88 ± 2.07 to −3.14 ± 2.09 (P = .346, 95% CI: [−0.18, 0.81]). The between-group difference in the changes was insignificant (0.29, P = .464, 95% CI: [−0.49, 1.06]) | | | | | | 685 |
| **No. missing participants** | N = 20 missing in intervention<br>N = 16 missing in control arm | | | | | | 684 |
| **Statistical methods used and appropriateness of these** *(e.g. adjustment for correlation)* | Differences in the clinical parameters and patient-reported outcomes between the control arm and the intervention arm were analysed using linear mixed models from baseline to 6 months. Linear mixed models were utilized by setting time-by-arm interaction terms. Study site was set a random effect to account for the clustering of groups over time, and social demographics were set as covariates.<br><br>Using multiple imputations (13.1% and 15.8% missing in the control arm and in the intervention arm, respectively) and mixed effects models. | | | | | | 684 |
| **Notes** | | | | | | | |

## Conclusions

| | **Description as stated in report/paper** | **Page number** |
|---|---|---|
| **Key conclusions of study authors** | Our study indicated that patients with long-standing diabetes benefitted from team-based pharmaceutical care over a six-month period. In addition to positive glycemic outcomes, team-based pharmaceutical care significantly improved overall self-care and glycemia without deteriorating quality of life and incurring productivity loss. | 686 |
| **Notes** | | |

# Appendix E National Heart, Lung, and Blood Institute tool quality assessments

National Heart, Lung, and Blood Institute controlled studies assessment

| | 1. Was the study described as randomised, a randomised trial, a randomised clinical trial, or an RCT? | 2. Was the method of randomisation adequate? | 3. Was the treatment allocation concealed? | 4. Were study participants and providers blinded to treatment group assignment? | 5. Were the people assessing the outcomes blinded to the participants' group assignments? | 6. Were the groups similar at baseline on important characteristics that could affect outcomes? | 7. Was the overall drop-out rate at endpoint 20% or lower of the number allocated to treatment? | 8. Was the differential drop-out rate at endpoint 15 percentage points or lower? | 9. Was there high adherence to the intervention protocols for each treatment group? | 10. Were other interventions avoided or similar in the groups? | 11. Were outcomes assessed using valid and reliable measures, implemented consistently across all study participants? | 12. Was the sample size sufficiently large to to detect a difference in the main outcome between groups with at least 80% power? | 13. Were outcomes reported or subgroups analysed pre-specified? | 14. Were all randomised participants analysed in the group to which they were originally assigned? |
|---|---|---|---|---|---|---|---|---|---|---|---|---|---|---|
| Boddy (2001) | No | No | No | No | No | Yes | Yes | Yes | Yes | CD | Yes | No | Yes | CD |
| Bruhn et al. (2013) | Yes | Yes | Yes | No | No | Yes | No | Yes | Yes | No | Yes | No | Yes | Yes |
| Damaske et al. (2005) | No | No | No | No | No | NR | Yes | Yes | Yes | Yes | Yes | No | Yes | Yes |
| Holland et al. (2023) | Yes | Yes | Yes | No | No | No | Yes | Yes | Yes | Yes | Yes | Yes | Yes | Yes |
| Jameson et al. (2010) | Yes | Yes | Yes | No | No | No | Yes | Yes | Yes | Yes | Yes | Yes | Yes | Yes |
| Lau et al. (2022) | Yes | Yes | Yes | No | Yes | No | No | Yes | Yes | Yes | Yes | Yes | Yes | No |
| Marotti et al. (2011) | Yes | Yes | Yes | No | CD | Yes | Yes | Yes | Yes | Yes | Yes | No | Yes | Yes |
| McAllister et al. (2014) | Yes | Yes | Yes | No | Yes | Yes | No | Yes | Yes | Yes | Yes | No | Yes | Yes |
| Ogilve et al. (2022) | Yes | Yes | Yes | No | No | CD | No | Yes | Yes | Yes | Yes | Yes | Yes | No |

| | | | | | | | | | | | | | | |
|---|---|---|---|---|---|---|---|---|---|---|---|---|---|---|
| Thompson et al. (1984) | No | No | No | No | No | Yes | Yes | Yes | Yes | Yes | Yes | No | No | No |
| Villeneueve et al. (2010) | Yes | Yes | No | No | No | No | Yes | Yes | Yes | No | Yes | Yes | Yes | Yes |
| Vivian (2002) | Yes | No | No | No | No | No | Yes | Yes | Yes | Yes | Yes | No | Yes | No |
| Xu et al. (2021) | Yes | Yes | Yes | No | No | CD | Yes | Yes | Yes | No | Yes | Yes | Yes | Yes |

National Heart, Lung, and Blood Institute cohort and observational studies assessment

| Study | 1. Was the research question or objective in this paper clearly stated? | 2. Was the study population clearly specified and defined? | 3. Was the participation rate of eligible persons at least 50%? | 4. Were all the subjects selected or recruited from similar populations? Were inclusion/ exclusion criteria prespecified and applied uniformly? | 5. Was a sample size justification, power description, or variance and effect estimates provided? | 6. For the analyses in this paper, were the exposure(s) of interest measured prior to the outcome(s) being measured? | 7. Was the timeframe sufficient so that one could reasonably expect to see an association between exposure and outcome if it existed? | 8. For exposures that can vary in amount or level, did the study examine different levels of the exposure as related to the outcome? | 9. Were the exposure measures clearly defined, valid, reliable, and implemented consistently across all study participants? | 10. Was the exposure(s) assessed more than once over time? | 11. Were the outcome measures clearly defined, valid, reliable, and implemented consistently across all study participants? | 12. Were the outcome assessors blinded to the exposure status of participants? | 13. Was loss to follow-up after baseline 20% or less? | 14. Were key potential confounding variables measured and adjusted statistically for the relationship between exposure(s) and outcome(s)? |
|---|---|---|---|---|---|---|---|---|---|---|---|---|---|---|
| Aspinall et al. (2012) | Yes | Yes | CD | Yes | No | Yes | Yes | No | Yes | NA | Yes | No | Yes | NR |
| Beahm et al. (2018) | Yes | Yes | Yes | Yes | Yes | Yes | Yes | No | Yes | NA | Yes | No | Yes | No |

| Study | 1. Was the research question or objective in this paper clearly stated? | 2. Was the study population clearly specified and defined? | 3. Was the participation rate of eligible persons at least 50%? | 4. Were all the subjects selected or recruited from similar populations? Were inclusion/ exclusion criteria prespecified and applied uniformly? | 5. Was a sample size justification, power description, or variance and effect estimates provided? | 6. For the analyses in this paper, were the exposure(s) of interest measured prior to the outcome(s) being measured? | 7. Was the timeframe sufficient so that one could reasonably expect to see an association between exposure and outcome if it existed? | 8. For exposures that can vary in amount or level, did the study examine different levels of the exposure as related to the outcome? | 9. Were the exposure measures clearly defined, valid, reliable, and implemented consistently across all study participants? | 10. Was the exposure(s) assessed more than once over time? | 11. Were the outcome measures clearly defined, valid, reliable, and implemented consistently across all study participants? | 12. Were the outcome assessors blinded to the exposure status of participants? | 13. Was loss to follow-up after baseline 20% or less? | 14. Were key potential confounding variables measured and adjusted statistically for the relationship between exposure(s) and outcome(s)? |
|---|---|---|---|---|---|---|---|---|---|---|---|---|---|---|
| Beahm et al. (2021) | Yes | Yes | Yes | Yes | No | Yes | Yes | No | Yes | NA | Yes | No | Yes | No |
| Cohen et al. (1985) | Yes | Yes | Yes | Yes | No | Yes | Yes | No | Yes | NA | Yes | No | NA | No |
| Cowart et al. (2020) | Yes | Yes | Yes | Yes | No | Yes | Yes | No | Yes | NA | Yes | No | Yes | No |
| Cowart et al. (2022) | Yes | Yes | Yes | Yes | No | Yes | Yes | No | Yes | NA | Yes | No | Yes | Yes |
| Hall et al. (2011) | Yes | Yes | Yes | Yes | Yes | Yes | Yes | No | Yes | NA | Yes | No | Yes | Yes |

| Study | 1. Was the research question or objective in this paper clearly stated? | 2. Was the study population clearly specified and defined? | 3. Was the participation rate of eligible persons at least 50%? | 4. Were all the subjects selected or recruited from similar populations? Were inclusion/ exclusion criteria prespecified and applied uniformly? | 5. Was a sample size justification, power description, or variance and effect estimates provided? | 6. For the analyses in this paper, were the exposure(s) of interest measured prior to the outcome(s) being measured? | 7. Was the timeframe sufficient so that one could reasonably expect to see an association between exposure and outcome if it existed? | 8. For exposures that can vary in amount or level, did the study examine different levels of the exposure as related to the outcome? | 9. Were the exposure measures clearly defined, valid, reliable, and implemented consistently across all study participants? | 10. Was the exposure(s) assessed more than once over time? | 11. Were the outcome measures clearly defined, valid, reliable, and implemented consistently across all study participants? | 12. Were the outcome assessors blinded to the exposure status of participants? | 13. Was loss to follow-up after baseline 20% or less? | 14. Were key potential confounding variables measured and adjusted statistically for the relationship between exposure(s) and outcome(s)? |
|---|---|---|---|---|---|---|---|---|---|---|---|---|---|---|
| Hahn et al. (2019) | Yes | Yes | Yes | Yes | No | Yes | Yes | No | Yes | NA | Yes | No | Yes | No |
| Hernández-Muñoz et al. (2021) | Yes | Yes | Yes | Yes | No | Yes | Yes | No | Yes | NA | Yes | No | CD | Yes |
| Lum et al. (2023) | Yes | Yes | Yes | Yes | Yes | Yes | Yes | No | Yes | NA | Yes | No | Yes | Yes |
| Maeng et al (2018) | Yes | Yes | Yes | Yes | No | Yes | Yes | No | Yes | NA | Yes | No | Yes | Yes |

| Study | 1. Was the research question or objective in this paper clearly stated? | 2. Was the study population clearly specified and defined? | 3. Was the participation rate of eligible persons at least 50%? | 4. Were all the subjects selected or recruited from similar populations? Were inclusion/ exclusion criteria prespecified and applied uniformly? | 5. Was a sample size justification, power description, or variance and effect estimates provided? | 6. For the analyses in this paper, were the exposure(s) of interest measured prior to the outcome(s) being measured? | 7. Was the timeframe sufficient so that one could reasonably expect to see an association between exposure and outcome if it existed? | 8. For exposures that can vary in amount or level, did the study examine different levels of the exposure as related to the outcome? | 9. Were the exposure measures clearly defined, valid, reliable, and implemented consistently across all study participants? | 10. Was the exposure(s) assessed more than once over time? | 11. Were the outcome measures clearly defined, valid, reliable, and implemented consistently across all study participants? | 12. Were the outcome assessors blinded to the exposure status of participants? | 13. Was loss to follow-up after baseline 20% or less? | 14. Were key potential confounding variables measured and adjusted statistically for the relationship between exposure(s) and outcome(s)? |
|---|---|---|---|---|---|---|---|---|---|---|---|---|---|---|
| Manzoor et al. (2018) | Yes | Yes | Yes | Yes | Yes | Yes | No | No | Yes | NA | Yes | No | Yes | Yes |
| McFarland et al. (2009) | Yes | Yes | Yes | Yes | No | Yes | Yes | No | Yes | NA | Yes | No | Yes | No |
| Morello et al. (2016) | Yes | Yes | Yes | Yes | Yes | Yes | Yes | No | Yes | NA | Yes | No | Yes | Yes |
| O'Neill et al. (2014) | Yes | Yes | Yes | CD | Yes | Yes | Yes | No | Yes | NA | Yes | No | No | Yes |
| Rana et al. (2023) | Yes | Yes | Yes | Yes | No | Yes | Yes | No | Yes | NA | Yes | No | Yes | No |

| Study | 1. Was the research question or objective in this paper clearly stated? | 2. Was the study population clearly specified and defined? | 3. Was the participation rate of eligible persons at least 50%? | 4. Were all the subjects selected or recruited from similar populations? Were inclusion/ exclusion criteria prespecified and applied uniformly? | 5. Was a sample size justification, power description, or variance and effect estimates provided? | 6. For the analyses in this paper, were the exposure(s) of interest measured prior to the outcome(s) being measured? | 7. Was the timeframe sufficient so that one could reasonably expect to see an association between exposure and outcome if it existed? | 8. For exposures that can vary in amount or level, did the study examine different levels of the exposure as related to the outcome? | 9. Were the exposure measures clearly defined, valid, reliable, and implemented consistently across all study participants? | 10. Was the exposure(s) assessed more than once over time? | 11. Were the outcome measures clearly defined, valid, reliable, and implemented consistently across all study participants? | 12. Were the outcome assessors blinded to the exposure status of participants? | 13. Was loss to follow-up after baseline 20% or less? | 14. Were key potential confounding variables measured and adjusted statistically for the relationship between exposure(s) and outcome(s)? |
|---|---|---|---|---|---|---|---|---|---|---|---|---|---|---|
| Rashid et al. (2020) | Yes | Yes | Yes | Yes | Yes | Yes | Yes | No | Yes | NA | Yes | No | Yes | Yes |
| Rodriguez et al. (2020) | Yes | Yes | Yes | No | Yes | Yes | Yes | No | Yes | NA | Yes | No | Yes | Yes |
| Rodriguez et al. (2021) | Yes | Yes | Yes | No | Yes | Yes | Yes | No | Yes | NA | Yes | No | Yes | Yes |
| Rodriguez et al. (2021b) | Yes | Yes | Yes | Yes | No | Yes | Yes | No | Yes | NA | Yes | No | Yes | No |
| Rodriguez et al. (2022) | Yes | Yes | Yes | Yes | No | Yes | Yes | No | Yes | NA | Yes | No | Yes | Yes |

| Study | 1. Was the research question or objective in this paper clearly stated? | 2. Was the study population clearly specified and defined? | 3. Was the participation rate of eligible persons at least 50%? | 4. Were all the subjects selected or recruited from similar populations? Were inclusion/ exclusion criteria prespecified and applied uniformly? | 5. Was a sample size justification, power description, or variance and effect estimates provided? | 6. For the analyses in this paper, were the exposure(s) of interest measured prior to the outcome(s) being measured? | 7. Was the timeframe sufficient so that one could reasonably expect to see an association between exposure and outcome if it existed? | 8. For exposures that can vary in amount or level, did the study examine different levels of the exposure as related to the outcome? | 9. Were the exposure measures clearly defined, valid, reliable, and implemented consistently across all study participants? | 10. Was the exposure(s) assessed more than once over time? | 11. Were the outcome measures clearly defined, valid, reliable, and implemented consistently across all study participants? | 12. Were the outcome assessors blinded to the exposure status of participants? | 13. Was loss to follow-up after baseline 20% or less? | 14. Were key potential confounding variables measured and adjusted statistically for the relationship between exposure(s) and outcome(s)? |
|---|---|---|---|---|---|---|---|---|---|---|---|---|---|---|
| Romanelli et al. (2015) | Yes | Yes | Yes | Yes | No | Yes | No | No | Yes | NA | Yes | No | Yes | Yes |
| Varghese et al. (2024) | Yes | Yes | Yes | Yes | No | Yes | Yes | No | Yes | NA | Yes | No | Yes | No |
| Weaver et al. (2023) | Yes | Yes | CD | Yes | No | Yes | CD | No | Yes | NA | Yes | No | Yes | Yes |

# Appendix F Table of characteristics

## Table of characteristics for effectiveness and safety studies

| Author | Country | Research question | Study design (sample size) | Study population(s) | Female (%) | Age | Setting | Prescriptive authority | Intervention description | Comparator/s | Duration |
|---|---|---|---|---|---|---|---|---|---|---|---|
| Aspinall et al. (2012) (55) | USA | To compare the quality of erythropoietin stimulating agents prescribing and monitoring for patients with non–dialysis-dependent chronic kidney disease in Veterans Affairs Medical Centers with and without pharmacist-managed erythropoietin stimulating agents clinics. | Retrospective cohort (n=572) | Veterans receiving long-term erythropoietin stimulating agents treatment | Intervention: 1.9%<br><br>Comparator 1: 3.3%<br><br>Comparator 2: 3.0% | Intervention: 73.9 ± 10.9<br><br>Comparator 1: 76.2 ± 12.0<br><br>Comparator 2: 78.4 ± 8.8 | Outpatient | Collaborative practice agreement | Pharmacists' scope of practice allowed them to dose and monitor erythropoietin stimulating agents therapy. Patients at most sites were referred to the pharmacist-managed erythropoietin stimulating agents clinic by a medical provider. | 1. Physician managed erythropoiesis-stimulating agent clinic<br><br>2. Usual care | Six months |
| Beahm et al. (2018) (56) | New Brunswick, Canada | To evaluate effectiveness, safety and patient satisfaction with pharmacist prescribing and care in patients with uncomplicated urinary tract infection. | Non-randomised trial (n= 750) | Patients aged 19 years and over with symptoms suggestive of urinary tract infection | Intervention: 100%<br><br>Comparator: 100% | Intervention: 40.4 ± 15.9<br><br>Comparator: 43.7 ± 16.1 | Community pharmacy | Independent prescribing | Pharmacists performed patient assessments for symptoms of urinary tract infection and prescribed antibacterial therapy, modified antibacterial therapy, provided education only or referred to physician, as appropriate. | Physician prescribing | Two weeks |

| Author | Country | Research question | Study design (sample size) | Study population(s) | Female (%) | Age | Setting | Prescriptive authority | Intervention description | Comparator/s | Duration |
|---|---|---|---|---|---|---|---|---|---|---|---|
| Beahm et al. (2021) (66) | New Brunswick, Canada | To further evaluate the appropriateness of antibacterial prescribing in the RxOUTMAP study. | Non-randomised trial (n= 750) | Patients aged 19 years and over with symptoms suggestive of urinary tract infection | Intervention: 100% Comparator: 100% | Intervention: 40.4 ± 15.9 Comparator: 43.7 ± 16.1 | Community pharmacy | Independent prescribing | Pharmacists performed patient assessments for symptoms of UTI and prescribed antibacterial therapy to patients presenting without a prescription. | Physician prescribing | Two weeks |
| Boddy (2001) (51) | England, UK | To evaluate the anticoagulation control on the medical wards, to implement warfarin guidelines, and to investigate the benefits of the guidelines for the doctors compared to pharmacists. | Non-randomised trial (n= 138) | Patients in acute care medical wards | Intervention: 51.4% Comparator: 46.9% | Intervention: 54 (range 22 - 74) Comparator: 57 (range 23 - 74) | Inpatient | Protocol | Warfarin dosing was conducted by haematology pharmacists. | Physician-prescribing | Unclear |
| Bruhn et al. (2013) (63) | England and Scotland, UK | To compare the effectiveness of pharmacist medication review, with or without pharmacist prescribing, with standard care, for patients with chronic pain. | RCT (n=196) | Patients aged 18 years and over, receiving regular prescribed medication for pain | Intervention: 54.4% Comparator 1: 74.2% Comparator 2: 58.7% | Intervention: 66.1 ± 12.1 Comparator 1: 65.7 ± 14.2 Comparator 2: 64.9 ± 11.6 | Primary care | Independent prescribing | Pharmacists conducted a medication review, agreed a pharmaceutical care plan with the patient, and issued any required prescriptions. | 1. GP prescribing with medication review 2. GP prescribing | 12 weeks |
| Chenella et al. (1983) (53) | California, USA | To determine the ability of the pharmacist to independently adjust heparin | RCT (n=81) | Hospitalised patients who were referred to the anticoagulant | Intervention (mean): 54.8% Comparator | Intervention: 46.0 ± 16.0 | Inpatient | Independent prescribing | Patients in the pharmacist–prescriber group had a pharmacist write daily heparin | Physician prescribing | Unclear |

| Author | Country | Research question | Study design (sample size) | Study population(s) | Female (%) | Age | Setting | Prescriptive authority | Intervention description | Comparator/s | Duration |
|---|---|---|---|---|---|---|---|---|---|---|---|
| | | and warfarin dosages. In this paper, the results of that study are reported. | | service by a primary care provider | r mean): 59.0% | Comparator: 52 ± 16.0 | | | and warfarin dosage adjustments. | | |
| Cohen et al. (1985) (49) | Michigan, USA | To compare the management of patients on warfarin therapy by the anticoagulation surveillance clinic and by other Veterans Administration Medical Centre clinics at the same institution. | Retrospective cohort (n=95) | Male outpatients who, over a 2.5-year period, had been monitored for warfarin therapy for a minimum of 3 months | Intervention: 0% Comparator: 0% | Intervention: 55.8 ± 10.5 Comparator: 57.8 ± 9.4 | Outpatient | Protocol | Patients were managed by clinic pharmacists according to a VAMC-approved protocol including education, drug-drug interaction screening and have warfarin dosage adjustments. | Physician prescribing | Unclear |
| Cowart et al. (2020) (35) | Florida, USA | To analyse the time to achieve an HbA1c of less than 7% for a pharmacist–physician managed cohort, as compared with a usual medical care cohort of patients with type 2 diabetes. | Retrospective cohort (n=257) | Patients aged 18 years and over with type 2 diabetes for at least 12 months | Not reported | Intervention: 59.8 ±11.6 Comparator: 57.9 ± 12.6 | Primary care | Collaborative practice agreement | Advanced practice pharmacists had authority to initiate, titrate, or discontinue antidiabetic medications; order drug therapy–related laboratory tests; and provide diabetes self-management education. | Primary care provider prescribing | Until goal achieved or last clinic visit |
| Cowart et al. (2022) (34) | Florida, USA | To evaluate time to treatment intensification in a pharmacist–physician management as compared with | Retrospective cohort (n=56) | Patients aged 18 years and over with type 2 diabetes for at least 12 months | Not reported | Intervention: 60.9 ± 11.2 Comparato | Primary care | Collaborative practice agreement | Advanced practice pharmacists to initiated, titrated, or discontinued antidiabetic medications, order antidiabetic | Physician prescribing | Eight visits |

| Author | Country | Research question | Study design (sample size) | Study population(s) | Female (%) | Age | Setting | Prescriptive authority | Intervention description | Comparator/s | Duration |
|---|---|---|---|---|---|---|---|---|---|---|---|
| | | usual medical care and to explore characteristics (method and type) of antidiabetic treatment intensification in the pharmacist–physician management and usual medical care cohorts. | | | | r: 60.6 ± 11.8 | | | therapy–related laboratory tests, and provide diabetes-related counselling. | | |
| Damaske et al. (2005) (50) | Texas, USA | To compare results from the protocol with results from usual, physician-directed warfarin therapy. | Non-randomised trial (n=51) | All patients in the identified service lines with an indication for warfarin | Not reported | Not reported | Inpatient | Protocol | Pharmacists followed a warfarin dosing protocol for 6 days. Dosage changes continued on day 7 of warfarin therapy until discharge by adjusting the dose 10% to 20% if the patient was still not within the target therapeutic range. | Physician prescribing | 6 days or until therapeutic range was reached |
| Hall et al. (2011) (52) | Pennsylvania, USA | To evaluate the differences in health care expenditures while accounting for operational costs, therapeutic outcomes, and patient compliance with laboratory tests | Retrospective cohort (n=350) | Patients aged 18 years and over with an index heart failure exacerbation admission | Intervention: 77%<br>Comparator: 77% | Intervention: 63.7<br>Comparator: 65.1 | Outpatient | Collaborative practice agreement | The pharmacists manage patients' anticoagulation through a collaborative care agreement and protocol under the referring physician's authority. | Physician prescribing | Not reported |

| Author | Country | Research question | Study design (sample size) | Study population(s) | Female (%) | Age | Setting | Prescriptive authority | Intervention description | Comparator/s | Duration |
|---|---|---|---|---|---|---|---|---|---|---|---|
| | | and warfarin refills in patients receiving warfarin therapy management by a pharmacist managed anticoagulation service compared with those receiving usual medical care. | | | | | | | | | |
| Hahn et al. (2019) (42) | Texas, USA | To evaluate post-discharge care provided by either a clinical pharmacy specialist with collaborative practice agreement, medication therapy management MTM pharmacist without collaborative practice agreement, or no pharmacist and their impact on all-cause and heart failre readmission rates. | Retrospective cohort (n=98) | Patients receiving anticoagulant therapy | Intervention: 62.9%<br><br>Comparator 1: 46.4%<br><br>Comparator 2: 34.3% | Intervention: 60.0 ± 13.0<br><br>Comparator 1: 63.0 ± 14.0<br><br>Comparator 2: 59.0 ± 14.0 | Outpatient | Collaborative practice agreement | Interventions provided by the clinical pharmacist specialist included medication therapy management, ordering referrals, nutrition, smoking cessation, medication access specialist, and anticoagulation management), making therapeutic medication changes including medication discontinuations and initiations, ordering medication refills and labs, and triaging acute issues by admitting patients directly to the emergency department. | 1. Medication therapy management pharmacist<br><br>2. Endocrinologist prescribing | Discharge, within 10 days after discharge, follow-up care during 21 days post-discharge |

| Author | Country | Research question | Study design (sample size) | Study population(s) | Female (%) | Age | Setting | Prescriptive authority | Intervention description | Comparator/s | Duration |
|---|---|---|---|---|---|---|---|---|---|---|---|
| Hernández-Muñoz et al. (2021) (39) | Texas, USA | To analyse a year's worth of endocrinologists' referral data to describe the impact of the pharmacist-led diabetes care collaborative model programme on the absolute change in HbA1c from baseline as compared to the group of diabetic patients not referred to the pharmacists by the endocrinologists. | Retrospective cohort (n=121) | Patients aged 18 years and over with type 2 diabetes mellitus | Not reported by subgroup | Not reported by subgroup | Outpatient | Collaborative practice agreement | A pharmacist, via written protocol with the endocrinologists of the clinic, could independently adjust, substitute, or discontinue the patient's diabetic pharmacotherapy regimen as needed. | 1. Endocrinologist prescribing | Endocrinologist determined the number of days between the initial endocrinologist visit and the first scheduled pharmacist visit. Pharamcist determined any subsequent visits. The mean number of days between pre and post index dates was 108. |
| Holland et al. (2023) (57) | UK | To estimate the effectiveness, cost effectiveness (to be reported elsewhere), and safety of pharmacy independent prescribers in care homes. | Cluster RCT (n=882) | People aged 65 years and over residing in a care home | Intervention: 72.0%<br>Comparator: 67.0% | Intervention: 85.1 ± 7.7<br>Comparator: 85.4 ± 7.6 | Long-term care | Independent prescribing | The pharmacist independent prescriber visited care homes to do medication reviews and optimise therapy for all participating residents. | GP prescribing | Six months |
| Jameson et al. (2010) (41) | Michigan, USA | To investigate the effect of pharmacist management of poorly controlled | RCT (n=104) | Patients aged 18 years and over with diabetics and an A1C levels | Intervention: 51.1% | Intervention: 49.3 ± 10.8 | Primary care | Collaborative practice agreement | Clinical pharmacists worked in the clinic on a part-time basis and were responsible for | Primary care provider prescribing | 12 months |

| Author | Country | Research question | Study design (sample size) | Study population(s) | Female (%) | Age | Setting | Prescriptive authority | Intervention description | Comparator/s | Duration |
|---|---|---|---|---|---|---|---|---|---|---|---|
| | | diabetes mellitus in a community-based primary care group. | | of 9.0% or higher | Comparator: 51.0% | Comparator: 49.7 ± 10.9 | | | managing drug therapy including starting or altering drug regimens, and ordering and assessing laboratory tests to monitor patient outcomes. | | |
| Lau et al. (2022) (58) | Alberta, Canada | To compare the efficacy and safety of combining home-based blood pressure telemonitoring and protocolised case management, and enhanced usual care with home-based blood pressure monitoring only, in older, community-dwelling adults. | RCT (n=120) | Patients aged 65 years and older residing in community-based supportive living | Intervention: 69% Comparator: 85% | Intervention: 79.8 ± 7.7 Comparator: 79.2 ± 7.4 | Long-term care | Protocol | Pharmacists administered behavioural counselling, education, reviewed telemonitored blood pressure summaries, remind participants and adjusted medications according to protocol. | Physician prescribing | 12 months |
| Lum et al. (2023) (36) | Singapore | To evaluate the changes in mean HbA1c level over 12 months and to identify care activities that were associated with this change. | Retrospective cohort (n=420) | Adults aged 21 years and over with type 2 diabetes | Intervention: 30.5% Comparator: 35.7% | Intervention: 65.8 ± 11.2 Comparator: 65.4 ± 11.1 | Outpatient | Collaborative practice agreement | Pharmacists provided medication review, identified drug–drug problems, and furnished prescriptions on behalf of the cardiologists with appropriate dose adjustments. | Cardiologist prescribing | 3-12 months |

| Author | Country | Research question | Study design (sample size) | Study population(s) | Female (%) | Age | Setting | Prescriptive authority | Intervention description | Comparator/s | Duration |
|---|---|---|---|---|---|---|---|---|---|---|---|
| Maeng et al. (2018) (38) | Pennsylvania and New Jersey, USA | To assess the impact of a medication therapy diabetes programme on the achievement of guideline-based disease targets, health care utilisation, and cost. | Retrospective cohort (n=5500) | Adults with a primary diagnosis of diabetes | Intervention: 50.0%<br><br>Comparator: 48.0% | Intervention: 59.0 ± 13<br><br>Comparator: 59.0 ± 13 | Primary care | Collaborative practice agreement | The pharmacist is authorised to manage prescriptions for all the diabetes mellitus related conditions. | Primary care provider prescribing | Minimum 12 months |
| Manzoor et al. (2018) (54) | Illinois, USA | To compare the quality of anticoagulation-related outcomes via two models of care, pharmacist-managed anticoagulation clinic and a nurse managed anticoagulation clinic. | Retrospective cohort (n=200) | Patients aged 18 years and over, being treated with warfarin | Intervention: 65.0%<br><br>Comparator: 52.0% | Intervention: 58.7 ± 15.5<br><br>Comparator: 64.2 ± 13.2 | Outpatient | Collaborative practice agreement | Pharmacists dose and manage warfarin under institutional collaborative practice agreements. | Nurse prescribing | 15 months |
| Marotti et al. (2011) (71) | Australia | To measure the effect of pharmacist involvement in medication history taking and supplementary prescribing in the perioperative setting. | RCT (n=332) | Elective surgical patients taking regular medications with a postoperative hospital stay of one night or more | Intervention: 49.0%<br><br>Comparator 1: 55.0%<br><br>Comparator 2: 51.0% | Intervention: 64 median (IQR 47-75)<br><br>Comparator 1: 62 median (IQR 52-71)<br><br>Comparator 2: 65 | Inpatient | Supplementary | Pharmacist prescribing was guided by protocols. Where patients did not fit the protocol, prescribing was guided by discussion with the patient's medical team. | 1. Medication review with physician prescribing<br><br>2. Physician prescribing | Six months |

| Author | Country | Research question | Study design (sample size) | Study population(s) | Female (%) | Age | Setting | Prescriptive authority | Intervention description | Comparator/s | Duration |
|---|---|---|---|---|---|---|---|---|---|---|---|
| | | | | | | median (IQR 54-75) | | | | | |
| McAlister et al. (2014) (45) | Alberta, Canada | A controlled comparison of two modes of case management: active prescribing (pharmacist-led case management) versus screening and delegating to primary care physicians (nurse-led case management). | RCT (n=279) | Patients aged 18 years and over who had a ischemic stroke or transient ischemic attack | Intervention: 39.2%<br><br>Comparator: 44.8% | Intervention: 66.8 ± 11.1<br><br>Comparator: 66.3 ± 11.3 | Primary care | Collaborative practice agreement | Pharmacists initiated or titrated antihypertensive and/or lipid-lowering therapy as appropriate | Physician prescribing with nurse case management | Six months |
| McFarland et al. (2009) (65) | Tennessee, USA | To evaluate the prevalence of potentially inappropriate dosing in patients for whom sitagliptin was initiated by a pharmacist versus patients in whom sitagliptin was initiated by a non-pharmacist prescriber. | Retrospective cohort (n=290) | Patients initiated on sitagliptin by a provider in the department of internal medicine | Not reported | Not reported | Outpatient | Collaborative practice agreement | A pharmacist had the authority to order laboratory tests, initiate medications, make referrals, and schedule follow-up when deemed necessary. | Physician prescribing | Not reported |
| Morello et al. (2016) (37) | California, USA | to assess mean change in A1C at 6 months after the baseline visit in the diabetes intense medical | Retrospective cohort (n=155) | Adults with type 2 diabetes with an A1C ≥8% | Intervention: 2.0%<br><br>Comparator: 3.6% | Intervention: 62.2 ± 8.1<br><br>Comparato | Outpatient | Collaborative practice agreement | The pharmacist had full laboratory ordering and prescribing authority to initiate, adjust, | Primary care provider prescribing | Six months |

| Author | Country | Research question | Study design (sample size) | Study population(s) | Female (%) | Age | Setting | Prescriptive authority | Intervention description | Comparator/s | Duration |
|---|---|---|---|---|---|---|---|---|---|---|---|
| | | management clinic and compare this group with a similar comparator group of type 2 diabetes mellitus patients who were not referred to the diabetes intense medical management clinic. | | | | r: 62.4 ± 10.0 | | | monitor, or discontinue medication therapy for diabetes and all related conditions | | |
| Ogilvie et al. (2022) (70) | Australia | An evaluation of a collaborative pharmacist prescribing model compared to the usual medical prescribing model in the emergency department. | RCT (n=94) | Adults, referred for medical admission from emergency into the hospital | Intervention: 47.4% Comparator: 40.0% | Intervention: 69.8 (range 44-89) Comparator: 70.9 (range 40-89) | Emergency department | Collaborative practice agreement | The pharmacist's scope of prescribing involved withholding or continuation of regular medications, and prescribing any new therapy based on the agreed pharmaceutical plan between admitting medical practitioner and pharmacist prescriber. | Physician prescribing | At admission |
| O'Neill et al. (2014) (47) | Michigan, USA | To compare blood pressure between clinical pharmacy specialists-directed and physician-directed | Retrospective cohort (n=126) | Veterans with uncontrolled hypertension | Intervention: 3.2% Comparator: 1.6% | Intervention: 63.4 ± 9.8 Comparator: 63.8 ± 10.0 | Outpatient | Collaborative practice agreement | Nurse assesses each patient present uncontrolled hypertension the case either to a physician or a CPS to collaboratively | Physician prescribing with nurse case management | Unclear, at least 2 visits |

| Author | Country | Research question | Study design (sample size) | Study population(s) | Female (%) | Age | Setting | Prescriptive authority | Intervention description | Comparator/s | Duration |
|---|---|---|---|---|---|---|---|---|---|---|---|
| | | registered nurse case management. | | | | | | | design a plan of care including education changing medication therapy, consulting referral services, laboratory test ordering and/or arranging follow-up. | | |
| Rana et al. (2023) (43) | USA | To determine if a pharmacist-led outpatient angiotensin receptor/neprilysin inhibitor replacement and titration programme led to more patients achieving target doses of sacubitril–valsartan compared to usual care. | Retrospective cohort (n=791) | Aged 18 years and over, diagnosed with heart failure with reduced ejection fraction, with an active sacubitril-valsartan prescription | Intervention: 32.8%<br>Comparator: 34.4% | Intervention: 57.1 ± 12.6<br>Comparator: 64.5 ± 12.6 | Outpatient | Collaborative practice agreement | Pharmacists initiate, titrate, monitor, clinical review, follow-up, counselling, insurance authorization/appeals for patient prescribed angiotensin receptor/neprilysin inhibitor. | Physician prescribing | Until target dose achieved |
| Rashid et al. (2020) (67) | California, USA | To evaluate effectiveness, safety, and economic outcomes of a pharmacist-managed deprescribing of non-steroidal anti-inflammatory drugs within an | Retrospective cohort (n=2155) | Adults at least 65 years of age receiving non-steroidal anti-inflammatory drugs | Intervention: 53.8%<br>Comparator: 55.1% | Intervention: 76.1 ± 6.7<br>Comparator: 75.8 ± 7.1 | Outpatient | Collaborative practice agreement | Clinical pharmacists obtain protocol authorization to review and deprescribe medications | Physician deprescribing | 6 months |

| Author | Country | Research question | Study design (sample size) | Study population(s) | Female (%) | Age | Setting | Prescriptive authority | Intervention description | Comparator/s | Duration |
|---|---|---|---|---|---|---|---|---|---|---|---|
| | | integrated health care system. | | | | | | | | | |
| Rodriguez et al. (2020) (68) | California, Colorado, Hawaii, and Oregon, USA | To test whether there are differences in the amount of contraceptive supply dispensed by pharmacists compared with clinicians. | Prospective cohort study (n=448) | Women aged 18-50 years presenting at a pharmacy to fill or initiate a new prescription for contraception for prevent pregnancy | Intervention: 100%<br>Comparator: 100% | Intervention<br>18-24 years: 56.94%<br>25-39 years: 18.06%<br>30-34 years: 15.28%<br>≥35 years: 9.72%<br>Comparator<br>18-24 years: 43.23%<br>25-39 years: 23.31%<br>30-34 years: 15.79%<br>≥35 years: 17.67% | Community pharmacy | Independent prescribing | Pharmacist initiate a new prescription for a contraceptive (pill, patch, ring, or injectable) | Physician prescribing | 12 months |
| Rodriguez et al. (2021a) (60) | California, Colorado, Hawaii, and Oregon, USA | To determine whether 12-month rates of continuation of an effective form of contraception or perfect use of contraception differ by prescribing provider | Prospective cohort (n=388) | Women aged 18-50 years presenting at a pharmacy to fill or initiate a new prescription for contraception | Intervention: 100%<br>Comparator: 100% | Mean: Not reported; Range: 18-50<br>Intervention: 45.2% 18-24<br>Comparator: 56.1% 18-24 | Community pharmacy | Independent prescribing | Pharmacist initiate a new prescription for a contraceptive (pill, patch, ring, or injectable) | Physician prescribing | 12 months |

| Author | Country | Research question | Study design (sample size) | Study population(s) | Female (%) | Age | Setting | Prescriptive authority | Intervention description | Comparator/s | Duration |
|---|---|---|---|---|---|---|---|---|---|---|---|
| | | (pharmacist or clinician). | | for prevent pregnancy | | | | | | | |
| Rodriguez et al. (2021b) (69) | Oregon, USA | To determine whether pharmacist prescription of contraception is associated with inappropriate prescription to women with medical contraindications | Retrospective cohort (n=439,240) | Women aged 18-50 years presenting at a pharmacy to fill or initiate a new prescription for contraception for prevent pregnancy | Intervention: 100% Comparator: 100% | 12-17 years: 11.4% 18-24 years: 31.3% 25-29 years: 21.8% 30-34 years: 16.8% ≥35 years: 17.6% | Community pharmacy | Independent prescribing | Pharmacist initiate a new prescription for a contraceptive (pill, patch, ring, or injectable) | Physician prescribing | After prescription |
| Rodriguez et al. (2022) (61) | Oregon, USA | To assess whether pharmacist prescription of combined hormonal contraception is associated with 12-month contraceptive continuation rates or breaks in contraceptive coverage | Retrospective cohort (n=172,325) | Females aged 12-51 years, who new users of short-acting reversible contraception | Intervention: 100% Comparator: 100% | Intervention: 50.5% 25-34 Comparator: 42.2% <25 | Community pharmacy | Independent prescribing | Pharmacist initiate a new prescription for a contraceptive (pill, patch, ring, or injectable) | Physician prescribing | 12 months |
| Romanelli et al. (2015) (33) | California, USA | To test the hypothesis that the addition of a clinical pharmacist to a patient-centered medical home team can augment patient | Retrospective cohort (n=1,108) | Adults seeking ambulatory care | Intervention: 66.79 ± 16.40 Comparator 1: 65.03 ± 16.26 Comparato | Intervention: 70.1% Comparator 1: 68.7% Comparator 2: 70.6% | patient centred medical home | Collaborative practice agreement | Pharmacist's intervention included coordination of care , disease management, and medication therapy management (refill orders; adjustment | 1. Physician prescribing 2. Primary care provider prescribing | 13.5 months |

| Author | Country | Research question | Study design (sample size) | Study population(s) | Female (%) | Age | Setting | Prescriptive authority | Intervention description | Comparator/s | Duration |
|---|---|---|---|---|---|---|---|---|---|---|---|
| | | care within a patient-centered medical home setting, resulting in reduced healthcare resource use and improved disease management as measured by intermediate clinical outcomes. | | | r 2: 64.86 ± 17.84 | | | | of medication therapy including discontinuation, modification, and addition of alternative medications) | | |
| Scullin et al. (2007) (64) | Northern Ireland, UK | To determine whether an increased input by clinical pharmacists at each stage of the patient's hospital journey, from admission through discharge, resulted in an enhanced level of patient care as measured by a number of clinical and economic outcomes. | RCT (n=762) | Patients who met one of the following criteria: 1. taking at least 4 regular medications; 2. taking a high-risk drug(s); 3. taking antidepressants and were 65 years of age or older; and 4. had a previous hospital admission within the last 6 months | Intervention: 55.0%<br>Comparator: 51.0% | Intervention: 70.3 ± 13.8<br>Comparator: 69.9 ± 14.8 | Inpatient | Protocol | Patients received an intensive clinical pharmacy service throughout their hospital stay including generating and authorising a discharge prescription according to protocols. | Physician prescribing | Until discharge |
| Thompson et al. (1984) (59) | California, USA | To determine whether direct intervention, through drug therapy | Non-randomised trial (n=152) | Adults residing in a nursing home | Intervention: 82.0% | Intervention: 85.1 ± 10.5 | Long-term care | Formulary | Clinical pharmacists made dose adjustments, or discontinued. | Physician prescribing | 12 months |

| Author | Country | Research question | Study design (sample size) | Study population(s) | Female (%) | Age | Setting | Prescriptive authority | Intervention description | Comparator/s | Duration |
|---|---|---|---|---|---|---|---|---|---|---|---|
| | | prescribing and patient care management, could improve the quality of patient care | | | Comparator: 85.0% | Comparator: 86.3 ± 8.1 | | | | | |
| Varghese et al. (2024) (44) | Wisconsin, USA | To implement and evaluate the benefit of a pharmacist driven aspirin deprescribing protocol compared with primary care provider education only in a primary care setting | Prospective cohort (n=122) | Veterans with an active prescription of aspirin for atherosclerotic cardiovascular disease who were at least 70 years of age | Intervention: 0% Comparator: 0% | Intervention and comparator: 75 (Range 70-81) | Primary care | Protocol | Using a standardized template, and the aspirin prescription was discontinued by the pharmacist, if appropriate. | Physician deprescribing | Unclear |
| Villeneueve et al. (2010) (46) | Québec, Canada | To compare a collaborative model involving physicians and pharmacists with usual care for patients with dyslipidaemia. | Cluster RCT (n=225) | Patients aged 18 and over with dyslipidaemia | Intervention: 36.0% Comparator: 40.0% | Intervention: 59.3 ± 9.6 Comparator: 62.2 ± 12.0 | Primary care | Collaborative practice agreement | The pharmacist provided counselling and used a patient decision aid to draw up a treatment plan, which included lifestyle changes and pharmacotherapy. The pharmacist then scheduled titration visits which included evaluate lifestyle changes, assessing tolerance of and adherence with the pharmacotherapy, and then adjusting | Physician prescribing | 12 months |

| Author | Country | Research question | Study design (sample size) | Study population(s) | Female (%) | Age | Setting | Prescriptive authority | Intervention description | Comparator/s | Duration |
|---|---|---|---|---|---|---|---|---|---|---|---|
| | | | | | | | | | the statin dosage if appropriate. | | |
| Vivian (2002) (48) | Pennsylvania, USA | To determine whether a pharmacist-managed hypertension clinic improves treatment outcomes in patients with hypertension. | RCT (n=56) | Patients aged 18 years and over with confirmed diagnosis of hypertension | Intervention: 0%<br>Comparator: 0% | Intervention: 64 ± 10.9<br>Comparator: 65.5 ± 7.8 | Primary care | Protocol | The pharmacist made drug therapy changes (in both drug selection and dosage) for blood pressure control following a protocol. | Physician prescribing | 6 months |
| Weaver et al. (2023) (62) | California, USA | To evaluate the impact of a pharmacist-led anaemia management service in pregnant patients with iron deficiency anaemia. | Retrospective cohort (n=100) | Pregnant women aged 16 years of age or older with iron deficiency anaemia | Intervention: 100%<br>Comparator: 100% | Intervention: 35.0 ± 4<br>Comparator: 35.0 ± 4 | Outpatient | Collaborative practice agreement | In a telephone-based ambulatory care pharmacy clinic performed pharmacists were authorized to initiate and adjust iron therapies, order baseline and follow-up laboratory tests, counsel patients, and assess the efficacy, safety, and tolerability of the iron regimens selected. | Obstetrician gynecologist prescribing | At delivery |
| Xu et al. (2021) (40) | Singapore | To examine the impact of pharmaceutical care on glycemia and self-care in patients with long-standing diabetes. | RCT (n=248) | Patients aged 21 years and over with uncontrolled type 2 diabetes (defined as A1C >7%); | Intervention: 69.0%<br>Comparator: 61.0% | Intervention: 59.7 ± 7.3<br>Comparator: 59.9 ± 6.8 | Primary care | Collaborative practice agreement | Pharmacists conducted medication review, dose adjustments, and insulin initiation and titration, switching drugs, and | Physician prescribing | 6 months |

| Author | Country | Research question | Study design (sample size) | Study population(s) | Female (%) | Age | Setting | Prescriptive authority | Intervention description | Comparator/s | Duration |
|---|---|---|---|---|---|---|---|---|---|---|---|
| | | | | | | | | | furnishing prescriptions. | | |

# Appendix G Feasibility assessment for meta-analysis

## Feasibility assessment for effectiveness outcomes

| Population | Effectiveness outcome | Outcome description | Author | Setting | Intervention | Comparison | Study design | Meta-analysis feasibility decision |
|---|---|---|---|---|---|---|---|---|
| Chronic kidney disease | Haemoglobin | Proportion of haemoglobin values within the target range of 10-12 g/dl | Aspinall *et al.* (2012) (intervention vs. clinic physician) | Outpatient clinic | Collaborative practice agreement | Clinic physician | Retrospective cohort | Too few studies |
| Chronic kidney disease | | Proportion of haemoglobin values within the target range of 10-12 g/dl | Aspinall *et al.* (2012) (intervention vs. usual care) | Outpatient clinic | Collaborative practice agreement | Usual care | Retrospective cohort | Too few studies |
| Urinary tract infection | Clinical cure | Clinical cure at two weeks | Beahm *et al.* (2018) | Community pharmacy | Independent | Physician prescribing | NRCT | Too few studies |
| Urinary tract infection | Waiting time | Time to access care | Beahm *et al.* (2018) | Community pharmacy | Independent | Physician prescribing | NRCT | Too few studies |
| Urinary tract infection | Adherence | Adherence (taken as prescribed) | Beahm *et al.* (2018) | Community pharmacy | Independent | Physician prescribing | NRCT | Too few studies |
| Anticoagulation | INR goal achieved | INR control achieved | Boddy (2001) | Inpatient | Protocol | Physician prescribing | NRCT | Too few studies |
| | INR goal achieved | INR within therapeutic range | Hall *et al.* (2011) | Outpatient clinic | Collaborative practice agreement | Usual care | Retrospective cohort | |
| Anticoagulation | Time to achieve therapeutic range | No. days to achieve therapeutic proconvertin and prothrombin | Chenella *et al.* (1983) | Inpatient | Independent | Physician prescribing | RCT | Too few studies |
| Anticoagulation | Time to achieve therapeutic range | Average time to therapeutic INR | Damaske *et al.* (2005) | Inpatient | Protocol | Physician prescribing | NRCT | Too few studies |
| Anticoagulation | | Time that INR was within therapeutic range | Hall *et al.* (2011) | Outpatient clinic | Collaborative practice agreement | Usual care | Retrospective cohort | Too few studies |
| Anticoagulation | Partial thromboplastin time (PTT) | Partial thromboplastin time (PTT) | Chenella *et al.* (1983) | Inpatient | Independent | Physician prescribing | RCT | Too few studies |

| Population | Effectiveness outcome | Outcome description | Author | Setting | Intervention | Comparison | Study design | Meta-analysis feasibility decision |
|---|---|---|---|---|---|---|---|---|
| Anticoagulation | Prothrombin time ratio | Prothrombin time ratio | Cohen *et al.* (1985) | Outpatient clinic | Protocol | Physician prescribing | Retrospective cohort | Too few studies |
| Anticoagulation | INR | Proportion INR in therapeutic range | Manzoor *et al.* 2018 | Outpatient clinic | Collaborative practice agreement | Nurse presrcibing | Retrospective cohort | Too few studies |
| Chronic pain | Health-related quality of life | Health-related quality of life | Bruhn *et al.* (2013) | Primary care | Independent | | RCT | Too few studies |
| Chronic pain | Health-related quality of life | Health-quality of life (physical component score) | Bruhn *et al.* (2013) (intervention vs medication review) | Primary care | Independent | Medication review | RCT | Too few studies |
| Chronic pain | Health-related quality of life | Health-related quality of life (physical component score) | Bruhn *et al.* (2013) (intervention vs usual care) | Primary care | Independent | Usual care | RCT | Too few studies |
| Chronic pain | Health-related quality of life | Health-related quality of life (mental component score) | Bruhn *et al.* (2013) (intervention vs medication review) | Primary care | Independent | Medication review | RCT | Too few studies |
| Chronic pain | Health-related quality of life | Health-related quality of life (mental component score) | Bruhn *et al.* (2013) (intervention vs usual care) | Primary care | Independent | Usual care | RCT | Too few studies |
| Chronic pain | Chronic pain | Chronic pain intensity | Bruhn *et al.* (2013) (intervention vs medication review) | Primary care | Independent | Medication review | RCT | Too few studies |
| Chronic pain | Chronic pain | Chronic pain intensity | Bruhn *et al.* (2013) (intervention vs usual care) | Primary care | Independent | Usual care | RCT | Too few studies |
| Chronic pain | Chronic pain | Chronic pain disability | Bruhn *et al.* (2013) (intervention vs medication review) | Primary care | Independent | Medication review | RCT | Too few studies |
| Chronic pain | Chronic pain | Chronic pain disability | Bruhn *et al.* (2013) (intervention vs usual care) | Primary care | Independent | Usual care | RCT | Too few studies |
| Chronic pain | Mental health | Depression | Bruhn *et al.* (2013) (intervention vs. medication review) | Primary care | Independent | Medication review | RCT | Too few studies |
| Chronic pain | Mental health | Depression | Bruhn *et al.* (2013) (intervention vs. usual care) | Primary care | Independent | Usual care | RCT | Too few studies |

| Population | Effectiveness outcome | Outcome description | Author | Setting | Intervention | Comparison | Study design | Meta-analysis feasibility decision |
|---|---|---|---|---|---|---|---|---|
| Chronic pain | Mental health | Anxiety | Bruhn *et al.* (2013) (intervention vs. medication review) | Primary care | Independent | Medication review | RCT | Too few studies |
| Chronic pain | Mental health | Anxiety | Bruhn *et al.* (2013) (intervention vs. usual care) | Primary care | Independent | Usual care | RCT | Too few studies |
| Type 2 diabetes | HbA1C | Goal achieved (yes/no) | Cowart *et al.* (2020) | Primary care | Collaborative practice agreement | Usual care | Retrospective cohort | Too few studies |
| Type 2 diabetes | HbA1C | | Cowart *et al.* (2022) | Primary care | Collaborative practice agreement | Physician prescribing | Retrospective cohort | |
| Type 2 diabetes | HbA1C | | Maeng *et al.* (2018) | Primary care | Collaborative practice agreement | Usual care (primary care provider clinic) | Retrospective cohort | |
| Type 2 diabetes | HbA1C | Time to achieve HbA1c goal | Cowart *et al.* (2020) | Primary care | Collaborative practice agreement | Usual care | Retrospective cohort | Too few studies |
| Type 2 diabetes | HbA1C | Change in HbA1C (pre and post index date) | Hernández-Muñoz *et al.* (2021) | Outpatient clinic | Collaborative practice agreement | Usual care (endocrinologist) | Retrospective cohort | Too few studies |
| Type 2 diabetes | HbA1C | Change in HbA1C | Jameson *et al.* (2010) | Primary care | Collaborative practice agreement | Usual care (primary care providers) | RCT | Comparator different, study designs too different |
| Type 2 diabetes | HbA1C | | Lum *et al.* (2023) | Outpatient clinic | Collaborative practice agreement | Cardiologist managed clinic | Retrospective cohort | |
| Type 2 diabetes | HbA1C | | Xu *et al.* (2021) | Primary care | Collaborative practice agreement | Physician prescribing | RCT | |
| Type 2 diabetes | HbA1C | Patients who achieved at least a 1.0% decrease in HbA1C | Jameson *et al.* (2010) | Primary care | Collaborative practice agreement | Usual care (primary care providers) | RCT | Too few studies |
| Type 2 diabetes | HbA1C | Mean HbA1c levels | Morello *et al.* (2016) | Outpatient clinic | Collaborative practice agreement | Usual care | Retrospective cohort | Too few studies |

| Population | Effectiveness outcome | Outcome description | Author | Setting | Intervention | Comparison | Study design | Meta-analysis feasibility decision |
|---|---|---|---|---|---|---|---|---|
| Type 2 diabetes | HbA1C | | Maeng *et al.* (2018) | Primary care | Collaborative practice agreement | Usual care (primary care provider clinic) | Retrospective cohort | |
| Type 2 diabetes | HbA1C | Relative risk for achieving an A1C of 7% or below | Irons *et al.* (2002) | Prison primary care | Collaborative practice agreement | Usual care (primary care providers) | Retrospective cohort | Too few studies |
| Type 2 diabetes | HbA1C | Fasting blood glucose | Morello *et al.* (2016) | Outpatient clinic | Collaborative practice agreement | Usual care | Retrospective cohort | Too few studies |
| Type 2 diabetes | LDL cholesterol | Mean LDL | Morello *et al.* (2016) | Outpatient clinic | Collaborative practice agreement | Usual care | Retrospective cohort | Too few studies |
| Type 2 diabetes | LDL cholesterol | | Maeng *et al.* (2018) | Primary care | Collaborative practice agreement | Usual care (primary care provider clinic) | Retrospective cohort | |
| Type 2 diabetes | LDL cholesterol | Goal achieved (yes/no) | Maeng *et al.* (2018) | Primary care | Collaborative practice agreement | Usual care (primary care provider clinic) | Retrospective cohort | Too few studies |
| Type 2 diabetes | HDL cholesterol | HDL cholesterol | Morello *et al.* (2016) | Outpatient clinic | Collaborative practice agreement | Usual care | Retrospective cohort | Too few studies |
| Type 2 diabetes | Triglycerides | Triglycerides | Morello *et al.* (2016) | Outpatient clinic | Collaborative practice agreement | Usual care | Retrospective cohort | Too few studies |
| Type 2 diabetes | Systolic blood pressure | Systolic blood pressure levels | Morello *et al.* (2016) | Outpatient clinic | Collaborative practice agreement | Usual care | Retrospective cohort | Too few studies |
| Type 2 diabetes | Systolic blood pressure | | Maeng *et al.* (2018) | Primary care | Collaborative practice agreement | Usual care (primary care provider clinic) | Retrospective cohort | |
| Type 2 diabetes | Diastolic blood pressure | Diastolic blood pressure levels | Morello *et al.* (2016) | Outpatient clinic | Collaborative practice agreement | Usual care | Retrospective cohort | Too few studies |
| Type 2 diabetes | Diastolic blood pressure | | Maeng *et al.* (2018) | Primary care | Collaborative practice agreement | Usual care (primary care provider clinic) | Retrospective cohort | |
| Type 2 diabetes | Blood pressure goal reached | Blood pressure goal reached | Maeng *et al.* (2018) | Primary care | Collaborative practice agreement | Usual care (primary care provider clinic) | Retrospective cohort | Too few studies |

| Population | Effectiveness outcome | Outcome description | Author | Setting | Intervention | Comparison | Study design | Meta-analysis feasibility decision |
|---|---|---|---|---|---|---|---|---|
| Type 2 diabetes | Time to treatment intensification | Time to treatment intensification | Cowart *et al.* (2022) | Primary care | Collaborative practice agreement | Physician prescribing | Retrospective cohort | Too few studies |
| Type 2 diabetes | Healthcare utilisation | Scheduled diabetes-related clinic visits | Irons *et al.* (2002) | Prison primary care | Collaborative practice agreement | Usual care (primary care providers) | Retrospective cohort | Too few studies |
| Type 2 diabetes | Healthcare utilisation | Unscheduled diabetes-related clinic visits | Irons *et al.* (2002) | Prison primary care | Collaborative practice agreement | Usual care (primary care providers) | Retrospective cohort | Too few studies |
| Type 2 diabetes | Health-related quality of life | Diabetes-specific quality of life | Xu *et al.* (2021) | Primary care | Collaborative practice agreement | Physician prescribing | RCT | Too few studies |
| Heart failure | Hospitalisations (healthcare utilisation) | 30-day all-cause readmission rate | Hahn *et al.* (2019) (intervention vs. medication therapy management with no CPA) | Outpatient clinic | Collaborative practice agreement | Pharmacist medication management | Retrospective cohort | Too few studies |
| Heart failure | Hospitalisations (healthcare utilisation) | 30-day all-cause readmission rate | Hahn *et al.* (2019) (intervention vs. usual care) | Outpatient clinic | Collaborative practice agreement | Usual care | Retrospective cohort | Too few studies |
| Heart failure | Hospitalisations (healthcare utilisation) | 30-day HF readmissions | Hahn *et al.* (2019) (intervention vs. medication therapy management with no CPA) | Outpatient clinic | Collaborative practice agreement | Pharmacist medication management | Retrospective cohort | Too few studies |
| Heart failure | Hospitalisations (healthcare utilisation) | 30-day HF readmissions | Hahn *et al.* (2019) (intervention vs. usual care) | Outpatient clinic | Collaborative practice agreement | Usual care | Retrospective cohort | Too few studies |
| Heart failure | Emergency department visits (healthcare utilisation) | Emergency department visits | Hahn *et al.* (2019) (intervention vs. medication therapy management with no CPA) | Outpatient clinic | Collaborative practice agreement | MTM (no CPA) | Retrospective cohort | Different populations |
| Heart failure | Emergency department visits (healthcare utilisation) | Emergency department visits | Hahn *et al.* (2019) (intervention vs. usual care) | Outpatient clinic | Collaborative practice agreement | Usual care | Retrospective cohort | Different populations |

| Population | Effectiveness outcome | Outcome description | Author | Setting | Intervention | Comparison | Study design | Meta-analysis feasibility decision |
|---|---|---|---|---|---|---|---|---|
| Heart failure | Target dose achieved | Percentage of patients achieving target ARNI dose | Rana *et al.* (2023) | Outpatient clinic | Collaborative practice agreement | Physician prescribing | Retrospective cohort | Too few studies |
| Heart failure | Target dose achieved | Number of visits required to achieve target dose | Rana *et al.* (2023) | Outpatient clinic | Collaborative practice agreement | Physician prescribing | Retrospective cohort | Too few studies |
| Heart failure | Target dose achieved | Days required to achieve target/maximally tolerated ARNI dose | Rana *et al.* (2023) | Outpatient clinic | Collaborative practice agreement | Physician prescribing | Retrospective cohort | Too few studies |
| Heart failure | Deprescribing | Proportion of aspirin deprescribing | Varghese *et al.* (2024) | Primary care | Protocol | Physician deprescribing | Prospective cohort study | Too few studies |
| Older person care | Falls | Fall rate/person at six months | Holland *et al.* (2023) | Long term care | Independent | Usual care (GP) | RCT | Too few studies |
| Older person care | Health-related quality of life | Health-related quality of life (EQ-5D by proxy) | Holland *et al.* (2023) | Long term care | Independent | Usual care (GP) | RCT | Too few studies |
| Older person care | Drug burden | Drug Burden Index | Holland *et al.* (2023) | Long term care | Independent | Usual care (GP) | RCT | Too few studies |
| Older person care | Hospitalisations (healthcare utilisation) | Hospitalisations | Holland *et al.* (2023) | Long term care | Independent | Usual care (GP) | RCT | Different interventions |
| Older person care | Hospitalisations (healthcare utilisation) | | Lau *et al.* (2022) | Long term care | Protocol | Enhanced usual care | RCT | |
| Older person care | Hospitalisations (healthcare utilisation) | | Thompson *et al.* (1984) | Long term care | Formulary | Physician prescribing | NRCT | |
| Older person care | Falls | Fall requiring medical attention | Lau *et al.* (2022) | Long term care | Protocol | Enhanced usual care | RCT | Too few studies |
| Older person care | Health-related quality of life | Health related quality of life | Lau *et al.* (2022) | Long term care | Protocol | Enhanced usual care | RCT | Too few studies |
| Older person care | Systolic blood pressure | Goal achieved (yes/no) | Lau *et al.* (2022) | Long term care | Protocol | Enhanced usual care | RCT | Too few studies |
| Older person care | Mental health | Depression | Lau *et al.* (2022) | Long term care | Protocol | Enhanced usual care | RCT | Too few studies |
| Older person care | Mental health | Anxiety | Lau *et al.* (2022) | Long term care | Protocol | Enhanced usual care | RCT | Too few studies |

| Population | Effectiveness outcome | Outcome description | Author | Setting | Intervention | Comparison | Study design | Meta-analysis feasibility decision |
|---|---|---|---|---|---|---|---|---|
| Older person care | Emergency department visits (healthcare utilisation) | Emergency department admissions | Lau *et al.* (2022) | Long term care | Protocol | Enhanced usual care | RCT | Different populations |
| Older person care | Drug burden | Average number of drugs per patient | Thompson *et al.* (1984) | Long term care | Formulary | Physician prescribing | NRCT | Too few studies |
| Stroke | Systolic blood pressure | Attained optimal systolic blood pressure and lipid level by 6 months | McAllister *et al.* (2014) | Primary care | Collaborative practice agreement | Nurse case management | RCT | Too few studies |
| Stroke | Systolic blood pressure | Systolic BP | McAllister *et al.* (2014) | Primary care | Collaborative practice agreement | Nurse case management | RCT | Too few studies |
| Stroke | LDL cholesterol | Mean LDL | McAllister *et al.* (2014) | Primary care | Collaborative practice agreement | Nurse case management | RCT | Too few studies |
| Stroke | HDL cholesterol | Change in HDL cholesterol | McAllister *et al.* (2014) | Primary care | Collaborative practice agreement | Nurse case management | RCT | Different populations |
| Stroke | Adherence | Self-reported adherence of 75% or higher for blood pressure or lipid-lowering medications | McAllister *et al.* (2014) | Primary care | Collaborative practice agreement | Nurse case management | RCT | Too few studies |
| Stroke | General health | Self-rated health | McAllister *et al.* (2014) | Primary care | Collaborative practice agreement | Nurse case management | RCT | Too few studies |
| Stroke | Health-related quality of life | Health-related quality of life | McAllister *et al.* (2014) | Primary care | Collaborative practice agreement | Nurse case management | RCT | Too few studies |
| Hypertension | Blood pressure goal achieved | Goal achieved (yes/no) | O'Neill *et al.* (2014) | Outpatient clinic | Collaborative practice agreement | Physician prescribing with nurse case management | Retrospective cohort | Too few studies |
| Hypertension | Systolic blood pressure | Systolic blood pressure change | O'Neill *et al.* (2014) | Outpatient clinic | Collaborative practice agreement | Physician prescribing with nurse case management | Retrospective cohort | Too few studies |

| Population | Effectiveness outcome | Outcome description | Author | Setting | Intervention | Comparison | Study design | Meta-analysis feasibility decision |
|---|---|---|---|---|---|---|---|---|
| Hypertension | Diastolic blood pressure | Diastolic blood pressure change | O'Neill *et al.* (2014) | Outpatient clinic | Collaborative practice agreement | Physician prescribing with nurse case management | Retrospective cohort | Too few studies |
| Hypertension | Systolic blood pressure | Systolic blood pressure | Vivian (2002) | Outpatient clinic | Protocol | Physician prescribing | NRCT | Too few studies |
| Hypertension | Diastolic blood pressure | Diastolic blood pressure | Vivian (2002) | Outpatient clinic | Protocol | Physician prescribing | NRCT | Too few studies |
| Hypertension | Adherence | Adherence | Vivian (2002) | Outpatient clinic | Protocol | Physician prescribing | NRCT | Too few studies |
| Hypertension | Adherence | Adherence | Vivian (2002) | Outpatient clinic | Protocol | Physician prescribing | NRCT | Too few studies |
| Hypertension | Health-related quality of life | Health-related quality of life | Vivian (2002) | Outpatient clinic | Protocol | Physician prescribing | NRCT | Too few studies |
| Hypertension | Health-related quality of life | Physical functioning | Vivian (2002) | Outpatient clinic | Protocol | Physician prescribing | NRCT | Too few studies |
| Hypertension | Health-related quality of life | Physical functioning | Vivian (2002) | Outpatient clinic | Protocol | Physician prescribing | NRCT | Too few studies |
| Hypertension | Health-related quality of life | Role limitations, physical | Vivian (2002) | Outpatient clinic | Protocol | Physician prescribing | NRCT | Too few studies |
| Hypertension | Health-related quality of life | Role limitations, emotional | Vivian (2002) | Outpatient clinic | Protocol | Physician prescribing | NRCT | Too few studies |
| Hypertension | Health-related quality of life | Social functioning | Vivian (2002) | Outpatient clinic | Protocol | Physician prescribing | NRCT | Too few studies |
| Hypertension | Health-related quality of life | Energy, fatigue | Vivian (2002) | Outpatient clinic | Protocol | Physician prescribing | NRCT | Too few studies |
| Hypertension | Health-related quality of life | Bodily pain | Vivian (2002) | Outpatient clinic | Protocol | Physician prescribing | NRCT | Too few studies |
| Contraception | Continuation | 12-month contraceptive continuation rate | Rodriguez *et al.* (2021) | Community pharmacy | Independent | Clinician prescribing | Prospective cohort | Too few studies |
| Contraception | Continuation | | Rodriguez *et al.* (2022) | Community pharmacy | Independent | Clinician prescribing | Retrospective cohort | |
| Contraception | Adherence | Perfect use across 12-months | Rodriguez *et al.* (2021) | Community pharmacy | Independent | Clinician prescribing | Prospective cohort | Too few studies, |

| Population | Effectiveness outcome | Outcome description | Author | Setting | Intervention | Comparison | Study design | Meta-analysis feasibility decision |
|---|---|---|---|---|---|---|---|---|
| Contraception | Adherence | | Rodriguez *et al.* (2022) | Community pharmacy | Independent | Clinician prescribing | Retrospective cohort | different study designs |
| Mixed health conditions | Clinic visits | Ambulatory care visits | Romanelli *et al.* (2015) (intervention medication management programme vs. patient centred medical home) | Primary care (medical home) | Collaborative practice agreement | Patient centred medical home | Retrospective cohort | Too few studies |
| Mixed health conditions | Clinic visits | Ambulatory care visits | Romanelli *et al.* (2015) (intervention vs. usual care) | Primary care (medical home) | Collaborative practice agreement | Usual care | Retrospective cohort | Too few studies |
| Mixed health conditions | Emergency department visits (healthcare utilisation) | Emergency department visits | Romanelli *et al.* (2015) (intervention medication management programme vs. patient centred medical home) | Primary care (medical home) | Collaborative practice agreement | Patient centred medical home | Retrospective cohort | Different populations |
| Mixed health conditions | Emergency department visits (healthcare utilisation) | Emergency department visits | Romanelli *et al.* (2015) (intervention vs. usual care) | Primary care (medical home) | Collaborative practice agreement | Usual care | Retrospective cohort | Different populations |
| Mixed health conditions | Hospitalisations (healthcare utilisation) | Hospitalisations | Romanelli *et al.* (2015) (intervention medication management programme vs. patient centred medical home) | Primary care (medical home) | Collaborative practice agreement | Patient centred medical home | Retrospective cohort | Too few studies |
| Mixed health conditions | Hospitalisations (healthcare utilisation) | Hospitalisations | Romanelli s*et al.* (2015) (intervention vs. usual care) | Primary care (medical home) | Collaborative practice agreement | Usual care | Retrospective cohort | Too few studies |

| Population | Effectiveness outcome | Outcome description | Author | Setting | Intervention | Comparison | Study design | Meta-analysis feasibility decision |
|---|---|---|---|---|---|---|---|---|
| Mixed health conditions | Systolic/diastolic blood pressure | Goal achieved (yes/no) | Romanelli *et al.* (2015) (intervention medication management programme vs. patient centred medical home) | Primary care (medical home) | Collaborative practice agreement | Patient centred medical home | Retrospective cohort | Too few studies |
| Mixed health conditions | Systolic/diastolic blood pressure | Goal achieved (yes/no) | Romanelli *et al.* (2015) (intervention vs. usual care) | Primary care (medical home) | Collaborative practice agreement | Usual care | Retrospective cohort | Too few studies |
| Mixed health conditions | LDL cholesterol | Goal achieved (yes/no) | Romanelli *et al.* (2015) (intervention medication management programme vs. patient centred medical home) | Primary care (medical home) | Collaborative practice agreement | Patient centred medical home | Retrospective cohort | Too few studies |
| Mixed health conditions | LDL cholesterol | Goal achieved (yes/no) | Romanelli *et al.* (2015) (intervention vs. usual care) | Primary care (medical home) | Collaborative practice agreement | Usual care | Retrospective cohort | Too few studies |
| Mixed health conditions | HbA1C | Goal achieved (yes/no) | Romanelli *et al.* (2015) (intervention medication management programme vs. patient centred medical home) | Primary care (medical home) | Collaborative practice agreement | Patient centred medical home | Retrospective cohort | Too few studies |
| Mixed health conditions | HbA1C | Goal achieved (yes/no) | Romanelli *et al.* (2015) (intervention vs. usual care) | Primary care (medical home) | Collaborative practice agreement | Usual care | Retrospective cohort | Too few studies |
| Mixed health conditions | Healthcare utilisation | Length of hospital stay | Scullin *et al.* (2007) | Inpatient | Protocol | Physician prescribing | RCT | Too few studies |
| Mixed health conditions | Hospitalisations (healthcare utilisation) | Hospital readmission | Scullin *et al.* (2007) | Inpatient | Protocol | Physician prescribing | RCT | Too few studies |

| Population | Effectiveness outcome | Outcome description | Author | Setting | Intervention | Comparison | Study design | Meta-analysis feasibility decision |
|---|---|---|---|---|---|---|---|---|
| Dyslipidaemia | LDL cholesterol | Mean LDL | Villeneueve *et al.* (2010) | Outpatient clinic | Collaborative practice agreement | Physician prescribing | RCT | Too few studies |
| Dyslipidaemia | Lipid levels achieved | Proportion achieving target lipid levels | Villeneueve *et al.* (2010) | Outpatient clinic | Collaborative practice agreement | Physician prescribing | RCT | Too few studies |
| Dyslipidaemia | HDL cholesterol | HDL cholesterol | Villeneueve *et al.* (2010) | Outpatient clinic | Collaborative practice agreement | Physician prescribing | RCT | Too few studies |
| Dyslipidaemia | Triglycerides | Triglycerides | Villeneueve *et al.* (2010) | Outpatient clinic | Collaborative practice agreement | Physician prescribing | RCT | Too few studies |
| Dyslipidaemia | Systolic blood pressure | Systolic blood pressure | Villeneueve *et al.* (2010) | Outpatient clinic | Collaborative practice agreement | Physician prescribing | RCT | Too few studies |
| Dyslipidaemia | Diastolic blood pressure | Diastolic blood pressure | Villeneueve *et al.* (2010) | Outpatient clinic | Collaborative practice agreement | Physician prescribing | RCT | Too few studies |
| Dyslipidaemia | HbA1C | Fasting blood glucose | Villeneueve *et al.* (2010) | Outpatient clinic | Collaborative practice agreement | Physician prescribing | RCT | Too few studies |
| Dyslipidaemia | Healthcare utilisation | Healthcare utilisation (number of physician visits) | Villeneueve *et al.* (2010) | Outpatient clinic | Collaborative practice agreement | Physician prescribing | RCT | Too few studies |
| Dyslipidaemia | Adherence | Adherence | Villeneueve *et al.* (2010) | Outpatient clinic | Collaborative practice agreement | Physician prescribing | RCT | Too few studies |
| Pregnancy anaemia | Haemoglobin | Percentage achieving target haemoglobin | Weaver *et al.* 2023 | Outpatient clinic | Protocol | Usual care (OB/GYN) | Retrospective cohort | Too few studies |
| Pregnancy anaemia | Haemoglobin | Haemoglobin level | Weaver *et al.* 2023 | Outpatient clinic | Protocol | Usual care (OB/GYN) | Retrospective cohort | Too few studies |

## Feasibility assessment for safety outcomes

| Population | Safety outcome | Outcome description | Author | Setting | Intervention | Comparison | Study design | Meta-analysis feasibility decision |
|---|---|---|---|---|---|---|---|---|
| Chronic kidney disease | Serious adverse drug reaction | Thromboembolica (adverse event) | Aspinall *et al.* (2012) (intervention vs. clinic physician) | Outpatient | Collaborative practice agreement | Physician managed | Retrospective cohort | Too few studies |
| Chronic kidney disease | Serious adverse drug reaction | Thromboembolica (adverse event) | Aspinall *et al.* (2012) (intervention vs. usual care) | Outpatient | Collaborative practice agreement | Usual care | Retrospective cohort | Too few studies |
| Chronic kidney disease | Serious adverse drug reaction | Heart failure (adverse event) | Aspinall *et al.* (2012) (intervention vs. clinic physician) | Outpatient | Collaborative practice agreement | Physician managed | Retrospective cohort | Too few studies |
| Chronic kidney disease | Serious adverse drug reaction | Heart failure (adverse event) | Aspinall *et al.* (2012) (intervention vs. usual care) | Outpatient | Collaborative practice agreement | Usual care | Retrospective cohort | Too few studies |
| Chronic kidney disease | Serious adverse drug reaction | Uncontrolled hypertension (adverse event) | Aspinall *et al.* (2012) (intervention vs. clinic physician) | Outpatient | Collaborative practice agreement | Physician managed | Retrospective cohort | Too few studies |
| Chronic kidney disease | Serious adverse drug reaction | Uncontrolled hypertension (adverse event) | Aspinall *et al.* (2012) (intervention vs. usual care) | Outpatient | Collaborative practice agreement | Usual care | Retrospective cohort | Too few studies |
| Chronic kidney disease | Inappropriate prescribing | Rates of inappropriate initial dosing | McFarland *et al.* (2009) | Outpatient | Collaborative practice agreement | Usual care | Retrospective cohort | Too few studies |
| Urinary tract infection | Serious adverse drug reaction | All adverse events | Beahm *et al.* (2018) | Community pharmacy | Independent | Physician prescribing | Trial (prospective registry) | Too few studies |
| Urinary tract infection | Serious adverse drug reaction | Gastrointestinal adverse events | Beahm *et al.* (2018) | Community pharmacy | Independent | Physician prescribing | Trial (prospective registry) | Too few studies |
| Urinary tract infection | Serious adverse drug reaction | Vaginal candidiasis adverse events | Beahm *et al.* (2018) | Community pharmacy | Independent | Physician prescribing | Trial (prospective registry) | Too few studies |
| Urinary tract infection | Serious adverse drug reaction | Headache adverse events | Beahm *et al.* (2018) | Community pharmacy | Independent | Physician prescribing | Trial (prospective registry) | Too few studies |
| Urinary tract infection | Serious adverse drug reaction | Other adverse events | Beahm *et al.* (2018) | Community pharmacy | Independent | Physician prescribing | Trial (prospective registry) | Too few studies |
| Urinary tract infection | Drug related hospital admissions | Physician or emergency department events | Beahm *et al.* (2018) | Community pharmacy | Independent | Physician prescribing | Trial (prospective registry) | Too few studies |

| Population | Safety outcome | Outcome description | Author | Setting | Intervention | Comparison | Study design | Meta-analysis feasibility decision |
|---|---|---|---|---|---|---|---|---|
| Urinary tract infection | Medication appropriateness | Antibacterial therapy guideline concordance | Beahm *et al.* (2021) | Community pharmacy | Independent | Physician prescribing | Trial (prospective registry) | Too few studies |
| Coagulation disorders | Serious adverse drug reaction | Warfarin-related complications (bleeding) | Cohen *et al.* (1985) | Outpatient | Protocol | Physician-managed Coagulation disorders clinic | Retrospective cohort | Different populations |
| Coagulation disorders | Serious adverse drug reaction | Thromboembolic events | Cohen *et al.* (1985) | Outpatient | Protocol | Physician-managed Coagulation disorders clinic | Retrospective cohort | Different populations |
| Coagulation disorders | Serious adverse drug reaction | Bleeds/adverse drug events | Damaske *et al.* (2005) | Inpatient | Protocol | Physician managed | Trial (pilot controlled) | Different populations |
| Coagulation disorders | Serious adverse drug reaction | Coagulation disorders-related adverse events | Hall *et al.* (2011) | Outpatient | Collaborative practice agreement | Usual care | Retrospective cohort | Different populations |
| Coagulation disorders | Drug related hospital admissions | Coagulation disorders-related emergency department visits | Hall *et al.* (2011) | Outpatient | Collaborative practice agreement | Usual care | Retrospective cohort | Too few studies |
| Coagulation disorders | Drug related hospital admissions | Coagulation disorders-related hospital admissions | Hall *et al.* (2011) | Outpatient | Collaborative practice agreement | Usual care | Retrospective cohort | Too few studies |
| Coagulation disorders | Drug related hospital admissions | Warfarin related hospitalisations/ emergency department | Manzoor *et al.* 2019 | Outpatient | Collaborative practice agreement | Nurse-managed Coagulation disorders clinic | Retrospective cohort | Too few studies |
| Type 2 diabetes | Serious adverse drug reaction | Frequency of hypoglycaemic events | Irons *et al.* (2002) | Primary care | Collaborative practice agreement | Usual care (primary care providers) | Retrospective cohort | Too few studies |
| Heart failure | Drug related hospital admissions | Heart failure hospitalisations | Rana *et al.* (2023) | Outpatient | Collaborative practice agreement | Physician prescribing | Retrospective cohort | Too few studies |
| Heart failure | Mortality | All-cause death | Rana *et al.* (2023) | Outpatient | Collaborative practice agreement | Physician prescribing | Retrospective cohort | Too few studies |

| Population | Safety outcome | Outcome description | Author | Setting | Intervention | Comparison | Study design | Meta-analysis feasibility decision |
|---|---|---|---|---|---|---|---|---|
| Older people in long-term care | Mortality | Mortality | Holland *et al.* (2023) | Long-term care | Independent | Usual care (GP) | RCT | Too few studies |
| Older people in long-term care | Serious adverse drug reaction | Syncope | Lau *et al.* (2022) | Long-term care | Protocol | Enhanced usual care | RCT | Too few studies |
| Older people in long-term care | Serious adverse drug reaction | Hypotension | Lau *et al.* (2022) | Long-term care | Protocol | Enhanced usual care | RCT | Too few studies |
| Older people in long-term care | Serious adverse drug reaction | Hypokalaemia | Lau *et al.* (2022) | Long-term care | Protocol | Enhanced usual care | RCT | Too few studies |
| Older people in long-term care | Serious adverse drug reaction | Hyperkalaemia | Lau *et al.* (2022) | Long-term care | Protocol | Enhanced usual care | RCT | Too few studies |
| Older people in long-term care | Serious adverse drug reaction | Hyponatremia | Lau *et al.* (2022) | Long-term care | Protocol | Enhanced usual care | RCT | Too few studies |
| Older people in long-term care | Serious adverse drug reaction | Orthostatic presyncope | Lau *et al.* (2022) | Long-term care | Protocol | Enhanced usual care | RCT | Too few studies |
| Older people in long-term care | Serious adverse drug reaction | Change in eGFR | Lau *et al.* (2022) | Long-term care | Protocol | Enhanced usual care | RCT | Too few studies |
| Mixed health conditions | Drug related hospital admissions | Gastrointestinal bleeding events (hospitalisations and emergency department visits) | Rashid *et al.* (2020) | Outpatient | Collaborative practice agreement | Physician deprescribing | Retrospective cohort | Too few studies |
| Mixed health conditions | Drug related hospital admissions | Acute kidney injury events (hospitalisations and ER visits) | Rashid *et al.* (2020) | Outpatient | Collaborative practice agreement | Physician deprescribing | Retrospective cohort | Too few studies |
| Mixed health conditions | Drug related hospital admissions | Pain (hospitalisations and emergency department visits) | Rashid *et al.* (2020) | Outpatient | Collaborative practice agreement | Physician deprescribing | Retrospective cohort | Too few studies |
| Older people in long-term | Mortality | Mortality | Thompson *et al.* (1984) | Long-term care | Formulary | Physician prescribing | NRCT | Too few studies |

| Population | Safety outcome | Outcome description | Author | Setting | Intervention | Comparison | Study design | Meta-analysis feasibility decision |
|---|---|---|---|---|---|---|---|---|
| care in long-term care | | | | | | | | |
| Stroke | Mortality | Mortality | McAllister *et al.* (2014) | Primary care | Collaborative practice agreement | Usual care | RCT | Too few studies |
| Stroke | Serious adverse drug reaction | Vascular event | McAllister *et al.* (2014) | Primary care | Collaborative practice agreement | Usual care | RCT | Too few studies |
| Surgery | Underuse | Doses missed during inpatient stay | Marotti *et al.* (2011) (intervention vs. medication review) | Hospital | Supplementary | Medication review | RCT | Too few studies |
| Surgery | Underuse | Doses missed during inpatient stay | Marotti *et al.* (2011) (intervention vs usual care) | Hospital | Supplementary | Usual care | RCT | Too few studies |
| Surgery | Medication appropriateness | Medication charted at incorrect dose | Marotti *et al.* (2011) (intervention vs. medication review) | Hospital | Supplementary | Medication review | RCT | Too few studies |
| Surgery | Medication appropriateness | Medication charted at incorrect dose | Marotti *et al.* (2011) (intervention vs usual care) | Hospital | Supplementary | Usual care | RCT | Too few studies |
| Surgery | Medication appropriateness | Medications charted at incorrect frequency | Marotti *et al.* (2011) (intervention vs. medication review) | Hospital | Supplementary | Medication review | RCT | Too few studies |
| Surgery | Medication appropriateness | Medications charted at incorrect frequency | Marotti *et al.* (2011) (intervention vs usual care) | Hospital | Supplementary | Usual care | RCT | Too few studies |
| Emergency department | Prescribing errors | Prescribing errors | Ogilve *et al.* (2022) | Emergency department | Collaborative practice agreement | Physician prescribing | RCT | Too few studies |
| At risk of drug related problems | Mortality | 12-month mortality | Scullin *et al.* (2007) | Inpatient | Protocol | Physician prescribing | RCT | Too few studies |
| Dyslipidemia | Serious adverse drug reaction | Adverse events | Villeneueve *et al.* (2010) | Family clinic | Collaborative practice agreement | Physician prescribing | RCT | Too few studies |
| Female contraceptive users | Clinically significant | Medical contraindications | Rodriguez *et al.* 2020 | Community pharmacy | Independent | Clinician prescribing | Prospective cohort | Too few studies |

| Population | Safety outcome | Outcome description | Author | Setting | Intervention | Comparison | Study design | Meta-analysis feasibility decision |
|---|---|---|---|---|---|---|---|---|
| | drug-drug interaction | | | | | | | |
| Female contraceptive users | Clinically significant drug-drug interaction | Medical contraindications | Rodriguez *et al.* 2021b | Community pharmacy | Independent | Clinician prescribing | Retrospective cohort | Too few studies |

# Appendix H Synthesis Without Meta-analysis (SWiM)

| SWiM reporting item | Item description | Page in manuscript where item is reported |
|---|---|---|
| *Methods* | | |
| **1** Grouping studies for synthesis | 1a) Provide a description of, and rationale for, the groups used in the synthesis (e.g., groupings of populations, interventions, outcomes, study design) | p6 |
| | 1b) Detail and provide rationale for any changes made subsequent to the protocol in the groups used in the synthesis | Not applicable |
| **2** Describe the standardised metric and transformation methods used | Describe the standardised metric for each outcome. Explain why the metric(s) was chosen, and describe any methods used to transform the intervention effects, as reported in the study, to the standardised metric, citing any methodological guidance consulted | Not applicable |
| **3** Describe the synthesis methods | Describe and justify the methods used to synthesise the effects for each outcome when it was not possible to undertake a meta-analysis of effect estimates | p6 |
| **4** Criteria used to prioritise results for summary and synthesis | Where applicable, provide the criteria used, with supporting justification, to select the particular studies, or a particular study, for the main synthesis or to draw conclusions from the synthesis (e.g., based on study design, risk of bias assessments, directness in relation to the review question) | Not applicable |
| **5** Investigation of heterogeneity in reported effects | State the method(s) used to examine heterogeneity in reported effects when it was not possible to undertake a meta-analysis of effect estimates and its extensions to investigate heterogeneity | Not applicable |
| **6** Certainty of evidence | Describe the methods used to assess certainty of the synthesis findings | p6 |
| **7** Data presentation methods | Describe the graphical and tabular methods used to present the effects (e.g., tables, forest plots, harvest plots).<br>Specify key study characteristics (e.g., study design, risk of bias) used to order the studies, in the text and any tables or graphs, clearly referencing the studies included | p6 |
| *Results* | | |
| **8** Reporting results | For each comparison and outcome, provide a description of the synthesised findings, and the certainty of the findings. Describe the result in language that is consistent with the question the synthesis addresses, and indicate which studies contribute to the synthesis | p8-13, Appendix F, Appendix M |
| *Discussion* | | |
| **9** Limitations of the synthesis | Report the limitations of the synthesis methods used and/or the groupings used in the synthesis, and how these affect the conclusions that can be drawn in relation to the original review question | p14-15 |

# Appendix I Excluded studies

A list of studies excluded at full-text screening stage are provided in this appendix. Reasons for exclusion included not meeting eligibility criteria for population, intervention, comparator, outcome or study design outlined in Section **Error! Reference source not found.**, Section **Error! Reference source not found.**, or Section **Error! Reference source not found.**. One study was excluded as it was unavailable (i.e. the full article could not be accessed).

## Excluded studies for Q1 and Q2 (effectiveness and safety)

| Excluded on population (n=1) | |
|---|---|
| 1. | Study on Impact of Clinical Pharmacist's Interventions in the Optimal Use of Oral Anticoagulants in Stroke Patients Lakshmi et al. Indian J Pharm Sci. 2013 Jan-Feb;75(1):53–59 |

| Excluded on intervention (n=182) | |
|---|---|
| 1. | Abah I O, Ojeh V B, Falang K D, et al. Pharmaceutical care outcomes in an outpatient human immunodeficiency virus treatment center in Jos, Nigeria. Journal of Basic and Clinical Pharmacy 2014;5:57–61. doi:10.4103/0976-0105.139727 |
| 2. | Abdulrhim Sara, Awaisu Ahmed, Ibrahim Mohamed Izham Mohamed, et al. Impact of pharmacist-involved collaborative care on diabetes management in a primary healthcare setting using real-world data. International journal of clinical pharmacy 2022;44:153–62. doi:10.1007/s11096-021-01327-x |
| 3. | Adibe Maxwell Ogochukwu, Ukwe Chinwe V, Aguwa Cletus N. The Impact of Pharmaceutical Care Intervention on the Quality of Life of Nigerian Patients Receiving Treatment for Type 2 Diabetes. Value in health regional issues 2013;2:240–7. doi:10.1016/j.vhri.2013.06.007 |
| 4. | Adler David A, Bungay Kathleen M, Wilson Ira B, et al. The impact of a pharmacist intervention on 6-month outcomes in depressed primary care patients. General hospital psychiatry 2004;26:199–209. doi:10.1016/j.genhosppsych.2003.08.005 |
| 5. | Airee Anita, Guirguis Alexander B, Mohammad Rima A. Clinical outcomes and pharmacists' acceptance of a community hospital's anticoagulation management service utilizing decentralized clinical staff pharmacists. The Annals of pharmacotherapy 2009;43:621–8. doi:10.1345/aph.1L460 |
| 6. | Al Mazroui Nadia Rashid, Kamal Mostafa Mohamed, Ghabash Naserdeen Mehana, et al. Influence of pharmaceutical care on health outcomes in patients with Type 2 diabetes mellitus. British journal of clinical pharmacology 2009;67:547–57. doi:10.1111/j.1365-2125.2009.03391.x |
| 7. | Al-Taie Anmar, Izzettin Fikret V, Sancar Mesut, et al. Impact of clinical pharmacy recommendations and patient counselling program among patients with diabetes and cancer in outpatient oncology setting. European journal of cancer care 2020;29:e13261. doi:10.1177/0145721708316625.; |
| 8. | Albsoul-Younes Abla M, Hammad Eman A, Yasein Nada A, et al. Pharmacist-physician collaboration improves blood pressure control. Saudi medical journal 2011;32:288–92. |

| Excluded on intervention (n=182) | |
|---|---|
| 9. | Alex Sumana, Adenew Ayne B, Arundel Cherinne, et al. Medication Errors Despite Using Electronic Health Records: The Value of a Clinical Pharmacist Service in Reducing Discharge-Related Medication Errors. Quality management in health care 2016;25:32–7. doi:10.1097/QMH.0000000000000080 |
| 10. | Amariles Pedro, Sabater-Hernández Daniel, García-Jiménez Emilio, et al. Effectiveness of Dader Method for Pharmaceutical Care on Control of Blood Pressure and Total Cholesterol in Outpatients with Cardiovascular Disease or Cardiovascular Risk: EMDADER-CV Randomized Controlled Trial. Journal of managed care pharmacy : JMCP 2012;18:311–23. doi:10.18553/jmcp.2012.18.4.311 |
| 11. | Ammerman Catherine A, Simpkins Brent A, Warman Nora, et al. Potentially Inappropriate Medications in Older Adults: Deprescribing with a Clinical Pharmacist. Journal of the American Geriatrics Society 2019;67:115–8. doi:10.1111/jgs.15623 |
| 12. | Anaya JP, Rivera JO, Lawson K, et al. Evaluation of pharmacist-managed diabetes mellitus under a collaborative drug therapy agreement. American Journal of Health-System Pharmacy 2008;65:1841–5. doi:10.2146/ajhp070568 |
| 13. | Anderegg Maxwell D, Gums Tyler H, Uribe Liz, et al. Physician-Pharmacist Collaborative Management: Narrowing the Socioeconomic Blood Pressure Gap. Hypertension (Dallas, Tex : 1979) 2016;68:1314–20. doi:10.1161/HYPERTENSIONAHA.116.08043 |
| 14. | Armour Carol L, Reddel Helen K, LeMay Kate S, et al. Feasibility and effectiveness of an evidence-based asthma service in Australian community pharmacies: a pragmatic cluster randomized trial. The Journal of asthma : official journal of the Association for the Care of Asthma 2012;50:302–9. doi:10.3109/02770903.2012.754463 |
| 15. | Ashjian Emily, Kurtz Brian, Renner Elizabeth, et al. Evaluation of a pharmacist-led outpatient direct oral anticoagulant service. American journal of health-system pharmacy : AJHP : official journal of the American Society of Health-System Pharmacists 2017;74:483–9. doi:10.2146/ajhp151026 |
| 16. | Aubert Carole E, Blum Manuel R, Gastens Viktoria, et al. Prescribing, deprescribing and potential adverse effects of proton pump inhibitors in older patients with multimorbidity: an observational study. CMAJ open 2023;11:E170–8. doi:10.9778/cmajo.20210240 |
| 17. | Bacci Jennifer L, Marcum Zachary A, Rodriguez Patricia, et al. Community pharmacist intervention to optimize statin adherence in diabetes care: The GuIDE-S study. Journal of the American Pharmacists Association : JAPhA 2023;63:946–51. doi:10.1016/j.japh.2023.03.002 |
| 18. | Baqir W, Hughes J, Jones T, et al. Impact of medication review, within a shared decision-making framework, on deprescribing in people living in care homes. European Journal of Hospital Pharmacy 2017;24:30–3. doi:10.1136/ejhpharm-2016-000900 |
| 19. | Batran Radwa Ahmed, Sabri Nagwa Ali, Ali Ihab, et al. Cost-Effectiveness of the Pharmacist-Managed Warfarin Therapy vs. Standard Care for Patients With Mechanical Mitral Valve Prostheses: An Egyptian Healthcare Perspective. Frontiers in cardiovascular medicine 2022;9:889197-NA. doi:10.3389/fcvm.2022.889197 |
| 20. | Bernsten Cecilia, Björkman Ingeborg, Caramona Margarida, et al. Improving the well-being of elderly patients via community pharmacy-based provision of pharmaceutical care: a multicentre study in seven European countries. Drugs & aging 2001;18:63–77. doi:10.2165/00002512-200118010-00005 |

| Excluded on intervention (n=182) | |
|---|---|
| 21. | Biscup-Horn Paula J, Streiff Michael B, Ulbrich Timothy R, et al. Impact of an Inpatient Anticoagulation Management Service on Clinical Outcomes. The Annals of pharmacotherapy 2008;42:777–82. doi:10.1345/aph.1l027 |
| 22. | Bloom Caitlyn I, Ku Mary, Williams Mikesha. Clinical pharmacy specialists' impact in patient aligned care teams for type 2 diabetes management. Journal of the American Pharmacists Association : JAPhA 2019;59:717–21. doi:10.1016/j.japh.2019.05.002 |
| 23. | Blyler Ciantel A, Rader Florian. Sustainability of blood pressure reduction in black barbershops. Current opinion in cardiology 2019;34:693–9. doi:10.1097/HCO.0000000000000674 |
| 24. | Bogden Paul E, Abbott Robert D, Williamson Pam, et al. Comparing Standard Care with a Physician and Pharmacist Team Approach for Uncontrolled Hypertension. Journal of general internal medicine 1998;13:740–5. doi:10.1046/j.1525-1497.1998.00225.x |
| 25. | Bond C, Matheson C, Williams S, et al. Repeat prescribing: a role for community pharmacists in controlling and monitoring repeat prescriptions. The British journal of general practice : the journal of the Royal College of General Practitioners 2000;50:271–5. |
| 26. | Bowers Brandi L, Drew Amy M, Verry Christian. Impact of Pharmacist-Physician Collaboration on Osteoporosis Treatment Rates. The Annals of pharmacotherapy 2018;52:876–83. doi:10.1177/1060028018770622 |
| 27. | Brook-Barclay L, Delaney C L, Scicchitano M, et al. Pharmacist influence on prescribing in peripheral arterial disease (PIPER). Vascular Medicine (United Kingdom) 2014;19:118–24. doi:10.1177/1358863X14523064 |
| 28. | Bucaloiu ID, Akers G, Bermudez MC, et al. Outpatient erythropoietin administered through a protocol-driven, pharmacist-managed program may produce significant patient and economic benefits. Manag Care Interface 2007;20:26–30. |
| 29. | Bungard Tammy J, Ritchie Bruce, Garg Sipi, et al. Sustained impact of anticoagulant control achieved in an anticoagulation management service after transfer of management to the primary care physician. Pharmacotherapy 2012;32:112–9. doi:10.1002/PHAR.1011 |
| 30. | Burns Naomi. Evaluation of warfarin dosing by pharmacists for elderly medical in-patients. Pharmacy world & science : PWS 2004;26:232–7. doi:10.1023/b:phar.0000035885.00408.9e |
| 31. | Carter Barry L, Coffey Christopher S, Ardery Gail, et al. Cluster-randomized trial of a physician/pharmacist collaborative model to improve blood pressure control. Circulation Cardiovascular quality and outcomes 2015;8:235–43. doi:10.1161/CIRCOUTCOMES.114.001283 |
| 32. | Carter Barry L, Doucette William R, Franciscus Carrie L, et al. Deterioration of blood pressure control after discontinuation of a physician-pharmacist collaborative intervention. Pharmacotherapy 2010;30:228–35. doi:10.1592/phco.30.3.228 |
| 33. | Chang Alex R, Evans Michael A, Yule Christina, et al. Using pharmacists to improve risk stratification and management of stage 3A chronic kidney disease: a feasibility study. BMC nephrology 2016;17:168–168. doi:10.1186/s12882-016-0383-7 |

| Excluded on intervention (n=182) | |
|---|---|
| 34. | Chen Dayu, Wen Bo, Wu Xuanyu, et al. Pharmacist-Driven Dosing Services and Pharmaceutical Care Increase Probability of Teicoplanin Target Concentration Attainment and Improve Clinical and Economic Outcomes in Non-Critically Ill Patients. Infectious diseases and therapy 2023;12:1579–92. doi:10.1007/s40121-023-00812-2 |
| 35. | Chen Q, Wang Q, Zhang Y. Clinical Intervention Increases Rational Use of Proton Pump Inhibitors in the General Surgery Department. Frontiers in Pharmacology 2022;13:864081. doi:10.3389/fphar.2022.864081 |
| 36. | Chen Ziqian, Ernst Michael E, Ardery Gail, et al. Physician-pharmacist co-management and 24-hour blood pressure control. Journal of clinical hypertension (Greenwich, Conn) 2013;15:337–43. doi:10.1111/jch.12077 |
| 37. | Chiquette E, Amato M G, Bussey H I. Comparison of an anticoagulation clinic with usual medical care: anticoagulation control, patient outcomes, and health care costs. Archives of Internal Medicine 1998;158:1641–7. doi:10.1001/archinte.158.15.1641 |
| 38. | Choe Hae Mi, Mitrovich Sonya, Dubay Daniel, et al. Proactive case management of high-risk patients with type 2 diabetes mellitus by a clinical pharmacist: a randomized controlled trial. The American journal of managed care 2005;11:253–60. |
| 39. | Cranor C W, Christensen D B. The Asheville project: Short-term outcomes of a community pharmacy diabetes care program. Journal of the American Pharmacists Association 2012;52:838–40. doi:10.1331/JAPhA.2012.12542 |
| 40. | Damaske DL, and Baird RW. Development and Implementation of a Pharmacist-Managed Inpatient Warfarin Protocol. Baylor University Medical Center Proceedings 2005;18:397–400. doi:10.1080/08998280.2005.11928100 |
| 41. | Dashti-Khavidaki Simin, Sharif Zahra, Khalili Hossein, et al. The use of pharmaceutical care to improve health-related quality of life in hemodialysis patients in Iran. International journal of clinical pharmacy 2013;35:260–7. doi:10.1007/s11096-012-9748-6 |
| 42. | Dawson Nancy L, Porter Ivan E, Klipa Dusko, et al. Inpatient warfarin management: pharmacist management using a detailed dosing protocol. Journal of thrombosis and thrombolysis 2011;33:178–84. doi:10.1007/s11239-011-0655-9 |
| 43. | DeLucenay Alexander, Curran Kelly, Karnes Angela. Impact of a pharmacist and nurse led clinic on patient blood pressure control. Journal of Interprofessional Education & Practice 2017;8:57–9. doi:10.1016/j.xjep.2017.06.003 |
| 44. | Dineen-Griffin Sarah, Benrimoj Shalom I, Rogers Kris, et al. Cluster randomised controlled trial evaluating the clinical and humanistic impact of a pharmacist-led minor ailment service. BMJ quality & safety 2020;29:921–31. doi:10.1136/bmjqs-2019-010608 |
| 45. | Dixon Dave L, Baker William L, Buckley Leo F, et al. Effect of a Physician/Pharmacist Collaborative Care Model on Time in Target Range for Systolic Blood Pressure: Post Hoc Analysis of the CAPTION Trial. Hypertension (Dallas, Tex : 1979) 2021;78:966–72. doi:10.1161/HYPERTENSIONAHA.121.17873 |
| 46. | Edey Rachel, Edwards Nicholas, Von Sychowski Jonah, et al. Impact of deprescribing rounds on discharge prescriptions: an interventional trial. International journal of clinical pharmacy 2019;41:159–66. doi:10.1007/s11096-018-0753-2 |

| Excluded on intervention (n=182) | |
|---|---|
| 47. | Ellis Samuel L, Carter Barry L, Malone Daniel C, et al. Clinical and Economic Impact of Ambulatory Care Clinical Pharmacists in Management of Dyslipidemia in Older Adults: The IMPROVE Study. Pharmacotherapy 2000;20:1508–16. doi:10.1592/phco.20.19.1508.34852 |
| 48. | Falamić Slaven, Lucijanic Marko, Ortner-Hadžiabdić Maja, et al. Pharmacists' influence on adverse reactions to warfarin: a randomised controlled trial in elderly rural patients. International journal of clinical pharmacy 2019;41:1166–73. doi:10.1007/s11096-019-00894-4 |
| 49. | Farrer K, Harper L, Shaffer J, et al. Can pharmacists and dietitians safely prescribe and administer parenteral nutrition? Pharmaceutical Journal 2008;280:626–30. |
| 50. | Fay Lauren N, Wolf Lauren M, Brandt Kasey L, et al. Pharmacist-led antimicrobial stewardship program in an urgent care setting. American journal of health-system pharmacy : AJHP : official journal of the American Society of Health-System Pharmacists 2019;76:175–81. doi:10.1093/ajhp/zxy023 |
| 51. | Finley Patrick R, Rens Heidi R, Pont Joan T, et al. Impact of a Collaborative Care Model on Depression in a Primary Care Setting: A Randomized Controlled Trial. Pharmacotherapy 2003;23:1175–85. doi:10.1592/phco.23.10.1175.32760 |
| 52. | Finn Shannon, D'Arcy Emily, Donovan Peter, et al. A randomised trial of pharmacist-led discharge prescribing in an Australian geriatric evaluation and management service. International journal of clinical pharmacy 2021;43:847–57. doi:10.1007/s11096-020-01184-0 |
| 53. | Fitzpatrick K, Addie K, Shaw M, et al. Implementing an innovative, patient-centered approach to day case arthroplasty: improving patient outcomes through remote preoperative pharmacist consultations. European Journal of Hospital Pharmacy 2023;:ejhpharm-2022. doi:10.1136/ejhpharm-2022-003573 |
| 54. | Freeman Christopher R, Scott Ian A, Hemming Karla, et al. Reducing Medical Admissions and Presentations Into Hospital through Optimising Medicines (REMAIN HOME): a stepped wedge, cluster randomised controlled trial. The Medical journal of Australia 2021;214:212–7. doi:10.5694/mja2.50942 |
| 55. | Fussell Sarah E, Butler Eamonn, Curtain Colin M, et al. Improving the accuracy of discharge medication documentation in people with kidney disease through pharmacist-led partnered prescribing. Internal medicine journal 2022;53:2102–10. doi:10.1111/imj.15979 |
| 56. | Gammaitoni A R, Gallagher R M, Welz M, et al. Palliative pharmaceutical care: a randomized, prospective study of telephone-based prescription and medication counseling services for treating chronic pain. Pain medicine (Malden, Mass) 2000;1:317–31. doi:10.1046/j.1526-4637.2000.00043.x |
| 57. | Gu Hongyan, Sun Lulu, Sheng Bo, et al. Benefits of pharmacist intervention in the critical care patients with infectious diseases: A propensity score matching retrospective cohort study. Australian critical care : official journal of the Confederation of Australian Critical Care Nurses 2023;36:933–9. doi:10.1016/j.aucc.2022.12.011 |
| 58. | Gums Tyler H, Uribe Liz, Vander Weg, et al. Pharmacist intervention for blood pressure control: medication intensification and adherence. Journal of the American Society of Hypertension : JASH 2015;9:569–78. doi:10.1016/j.jash.2015.05.005 |
| 59. | Gupta Aditi, Ellis Shellie D, Burkhardt Crystal, et al. Implementing a home-based virtual hypertension programme-a pilot feasibility study. Family practice 2023;40:414–22. doi:10.1093/fampra/cmac084 |

| Excluded on intervention (n=182) | |
|---|---|
| 60. | Gutierrez Margarita M, Sakulbumrungsil Rungpetch. Effectiveness of a pharmacist-led expert system intervention for medication adherence and blood pressure control of adults with hypertension: A randomized controlled trial. Research in social & administrative pharmacy : RSAP 2023;19:931–43. doi:10.1016/j.sapharm.2023.03.004 |
| 61. | Haider Ibrahim, Kosari Sam, Naunton Mark, et al. Impact of on-site pharmacists in residential aged care facilities on the quality of medicines use: a cluster randomised controlled trial (PiRACF study). Scientific reports 2023;13:15962. doi:10.1038/s41598-023-42894-5 |
| 62. | Hale A R, Coombes I D, Stokes J, et al. Perioperative medication management: expanding the role of the preadmission clinic pharmacist in a single centre, randomised controlled trial of collaborative prescribing. BMJ open 2013;3. doi:10.1136/bmjopen-2013-003027 |
| 63. | Hanna J. Real-world application of MedsCheck opportunities: The Costco pharmacists intervention trial for reduction of cardiovascular risk. Canadian Pharmacists Journal 2013;146:325–8. doi:10.1177/1715163513506831 |
| 64. | Haverkamp Kayla, Newberry Paula, Baker Jennifer. Impact of a pharmacist-run weight loss medication management service. Journal of the American Pharmacists Association : JAPhA 2022;62:883–8. doi:10.1016/j.japh.2021.11.022 |
| 65. | Hay Elaine M, Foster Nadine E, Thomas Elaine, et al. Effectiveness of community physiotherapy and enhanced pharmacy review for knee pain in people aged over 55 presenting to primary care: pragmatic randomised trial. BMJ (Clinical research ed) 2006;333:995–995. doi:10.1136/bmj.38977.590752.0b |
| 66. | Hedegaard Ulla, Kjeldsen Lene Juel, Pottegård Anton, et al. Improving Medication Adherence in Patients with Hypertension: A Randomized Trial. The American journal of medicine 2015;128:1351–61. doi:10.1016/j.amjmed.2015.08.011 |
| 67. | Hester E K, Caulder C R, Penzak S R. Impact of team-based care on appropriate statin therapy prescribing in ***HIV-infected patients. JACCP Journal of the American College of Clinical Pharmacy 2020;3:615–22. doi:10.1002/jac5.1206 |
| 68. | Hick H L, Deady P E, Wright D J, et al. The impact of the pharmacist on an elective general surgery pre-admission clinic. Pharmacy world & science : PWS 2001;23:65–9. doi:10.1023/a:1011231622947 |
| 69. | Ho P Michael, Lambert-Kerzner Anne, Carey Evan P, et al. Multifaceted intervention to improve medication adherence and secondary prevention measures after acute coronary syndrome hospital discharge: a randomized clinical trial. JAMA internal medicine 2014;174:186–93. doi:10.1001/jamainternmed.2013.12944 |
| 70. | Holden J, Holden K. Comparative effectiveness of general practitioner versus pharmacist dosing of patients requiring anticoagulation in the community. Journal of clinical pharmacy and therapeutics 2000;25:49–54. doi:10.1046/j.1365-2710.2000.00262.x |
| 71. | Hui Rita L, Chang Christopher C, Niu Fang, et al. Evaluation of a Pharmacist-Managed Antidiabetic Deprescribing Program in an Integrated Health Care System. Journal of managed care & specialty pharmacy 2019;25:927–34. doi:10.18553/jmcp.2019.25.8.927 |
| 72. | Hunt Jacquelyn S, Siemienczuk Joseph, Pape Ginger, et al. A randomized controlled trial of team-based care: impact of physician-pharmacist collaboration on uncontrolled hypertension. Journal of general internal medicine 2008;23:1966–72. doi:10.1007/s11606-008-0791-x |

| Excluded on intervention (n=182) | |
|---|---|
| 73. | Hunt Jacquelyn S, Siemienczuk Joseph, Pape Ginger, et al. A randomized controlled trial of team-based care: impact of physician-pharmacist collaboration on uncontrolled hypertension. Journal of general internal medicine 2008;23:1966–72. doi:10.1007/s11606-008-0791-x |
| 74. | Jacobs Michelle, Sherry Pamela S, Taylor Leigh M, et al. Pharmacist Assisted Medication Program Enhancing the Regulation of Diabetes (PAMPERED) study. Journal of the American Pharmacists Association : JAPhA 2012;52:613–21. doi:10.1331/JAPhA.2012.10183 |
| 75. | Jang Ha Young, Kim Yon Su, Oh Jung Mi. Clinical Effectiveness of Renal Transplant Outpatient Pharmaceutical Care Services in Korea. Healthcare (Basel, Switzerland) 2023;11:2597–2597. doi:10.3390/healthcare11182597 |
| 76. | Jarab Anan Sadeq, Alqudah Salam Ghazi, Mukattash Tareq Lewis, et al. Randomized controlled trial of clinical pharmacy management of patients with type 2 diabetes in an outpatient diabetes clinic in Jordan. Journal of managed care pharmacy : JMCP 2012;18:516–26. doi:10.18553/jmcp.2012.18.7.516 |
| 77. | Javaid Zaida, Imtiaz Unaiza, Khalid Imtiaz, et al. A randomized control trial of primary care-based management of type 2 diabetes by a pharmacist in Pakistan. BMC health services research 2019;19:409. doi:10.1186/s12913-019-4274-z |
| 78. | Jones Aubrey E, King Jordan B, Kim Kibum, et al. The role of clinical pharmacy anticoagulation services in direct oral anticoagulant monitoring. Journal of thrombosis and thrombolysis 2020;50:739–45. doi:10.1007/s11239-020-02064-0 |
| 79. | Khadela Avinash, Bhikadiya Vishal, Vyas Bhavin. Impact of oncology pharmacist services on humanistic outcome in patients with breast cancer. Journal of oncology pharmacy practice : official publication of the International Society of Oncology Pharmacy Practitioners 2022;28:302–9. doi:10.1177/1078155220988333 |
| 80. | Khadela Avinash, Vyas Bhavin, Bhikadiya Vishal, et al. Impact of clinical pharmacist services on quality adjusted life years in head and neck cancer patients. International journal of clinical pharmacy 2021;43:1208–17. doi:10.1007/s11096-021-01235-0 |
| 81. | Khazaka Michael, Laverdière Jeanne, Li Chen Chen, et al. Medication appropriateness on an acute geriatric care unit: the impact of the removal of a clinical pharmacist. Age and ageing 2021;50:527–33. doi:10.1093/ageing/afaa175 |
| 82. | Kido Kazuhiko, Fang Wei, Broscious Rachael, et al. Evaluation of a pharmacist-provider collaborative clinic for treatment of iron deficiency in patients with heart failure. American journal of health-system pharmacy : AJHP : official journal of the American Society of Health-System Pharmacists 2023;80:1326–35. doi:10.1093/ajhp/zxad149 |
| 83. | Kulchaitanaroaj Puttarin, Brooks John M, Ardery Gail, et al. Incremental costs associated with physician and pharmacist collaboration to improve blood pressure control. Pharmacotherapy 2012;32:772–80. doi:10.1002/j.1875-9114.2012.01103.x |
| 84. | Ladhar Simroop, Hawkins Nathaniel M, Virani Sean A, et al. Evaluation of PHARM-HF, a pharmacist-led heart failure medication titration clinic: A pre-post study. JACCP: JOURNAL OF THE AMERICAN COLLEGE OF CLINICAL PHARMACY 2024;7:416–25. doi:10.1002/jac5.1920 |
| 85. | Lee Jeannie K, Grace Karen A, Taylor Allen J. Effect of a Pharmacy Care Program on Medication Adherence and Persistence, Blood Pressure, and Low-Density Lipoprotein Cholesterol: A Randomized Controlled Trial. JAMA 2006;296:2563–71. doi:10.1001/jama.296.21.joc60162 |

| Excluded on intervention (n=182) | |
|---|---|
| 86. | Leehey David J, Braun Barbara I, Tholl Debra A, et al. Can pharmacokinetic dosing decrease nephrotoxicity associated with aminoglycoside therapy. Journal of the American Society of Nephrology : JASN 1993;4:81–90. doi:10.1681/asn.v4181 |
| 87. | Leendertse Anne J, De Koning G H. P, Goudswaard A N, et al. Preventing hospital admissions by reviewing medication (PHARM) in primary care: An open controlled study in an elderly population. Journal of clinical pharmacy and therapeutics 2013;38:379–87. doi:10.1111/jcpt.12069 |
| 88. | Lenssen Rebekka, Schmitz K, Griesel C M, et al. Comprehensive pharmaceutical care to prevent drug-related readmissions of dependent-living elderly patients: a randomized controlled trial. BMC geriatrics 2018;18:135–135. doi:10.1186/s12877-018-0814-3 |
| 89. | Lim Phei Ching, Lim Kelvin, Embee Zubaidah Che, et al. Study investigating the impact of pharmacist involvement on the outcomes of diabetes medication therapy adherence program Malaysia. Pakistan journal of pharmaceutical sciences 2016;29:595–601. |
| 90. | Lowrie Richard, Mair Frances S, Greenlaw Nicola, et al. Pharmacist intervention in primary care to improve outcomes in patients with left ventricular systolic dysfunction. European heart journal 2012;33:314–24. doi:10.1093/eurheartj/ehr433 |
| 91. | Lowrie Richard, Stock Kate, Lucey Sharon, et al. Pharmacist led homeless outreach engagement and non-medical independent prescribing (Rx) (PHOENIx) intervention for people experiencing homelessness: a non- randomised feasibility study. International journal for equity in health 2021;20:19. doi:10.1186/s12939-020-01337-7 |
| 92. | Lum Zheng Kang, Chang Kai Li, Tsou Keith Yu-Kei, et al. Enhancing diabetes care with community pharmacist-involved collaborative care model: A multi-centre randomised controlled trial. Diabetes research and clinical practice 2022;185:109238. doi:10.1016/j.diabres.2022.109238 |
| 93. | Malone Daniel C, Carter Barry L, Billups Sarah J, et al. Can clinical pharmacists affect SF-36 scores in veterans at high risk for medication-related problems? Medical care 2001;39:113–22. doi:10.1097/00005650-200102000-00002 |
| 94. | Marque Philippine, Le Moal Gwenael, Labarre Chloé, et al. Assessment of the impact of pharmacist-led intervention with antibiotics in patients with bone and joint infection. Infectious diseases now 2023;53:104671. doi:10.1016/j.idnow.2023.104671 |
| 95. | Marra Carlo A, Cibere Jolanda, Grubisic Maja, et al. Pharmacist-initiated intervention trial in osteoarthritis: a multidisciplinary intervention for knee osteoarthritis. Arthritis care & research 2012;64:1837–45. doi:10.1002/acr.21763 |
| 96. | Mashni Ola K, Nazer Lama H, Khalil Haya Z, et al. Impact of Clinical Pharmacy Services on Patient Management in the Chemotherapy Infusion Clinics: A 5-Year Study at a Comprehensive Cancer Center. Journal of pharmacy practice 2022;35:686–90. doi:10.1177/08971900211003446 |
| 97. | Masuda Nobutoshi, Maiguma Takayoshi, Komoto Atsushi, et al. Impact of pharmacist intervention on preventing nephrotoxicity from vancomycin. International journal of clinical pharmacology and therapeutics 2015;53:284–91. doi:10.5414/CP202274 |
| 98. | Masuda Quamrun N, Smith Jamie E, Gaines JaViere, et al. Outcomes of Pharmacist-Led Treatment of Hepatitis C in the Virginia Department of Corrections. Journal of correctional health care : the official journal of the National Commission on Correctional Health Care 2023;29:430–8. doi:10.1089/jchc.23.03.0025 |

| Excluded on intervention (n=182) | |
|---|---|
| 99. | Matzke Gary R, Moczygemba Leticia R, Williams Karen J, et al. Impact of a pharmacist-physician collaborative care model on patient outcomes and health services utilization. American journal of health-system pharmacy : AJHP : official journal of the American Society of Health-System Pharmacists 2018;75:1039–47. doi:10.2146/ajhp170789 |
| 100. | Mazzolini Timothy A, Irons Brian K, Schell Evans C, et al. Lipid Levels and Use of Lipid-Lowering Drugs for Patients in Pharmacist-Managed Lipid Clinics Versus Usual Care in 2 VA Medical Centers. Journal of managed care pharmacy : JMCP 2005;11:763–71. doi:10.18553/jmcp.2005.11.9.763 |
| 101. | McGhan William F, Stimmel Glen L, Hall Thomas G, et al. A Comparison of Pharmacists and Physicians on the Quality of Prescribing for Ambulatory Hypertensive Patients. Medical care 1983;21:435–44. doi:10.1097/00005650-198304000-00006 |
| 102. | Michal Jessica, Henry Thomas, Street Connie. Impact of a pharmacist-driven protocol to decrease proton pump inhibitor use in non-intensive care hospitalized adults. American journal of health-system pharmacy : AJHP : official journal of the American Society of Health-System Pharmacists 2016;73:S126–32. doi:10.2146/ajhp150519 |
| 103. | Michalets Elizabeth, Creger Julie, Shillinglaw William R. Outcomes of expanded use of clinical pharmacist practitioners in addition to team-based care in a community health system intensive care unit. American journal of health-system pharmacy : AJHP : official journal of the American Society of Health-System Pharmacists 2015;72:47–53. doi:10.2146/ajhp140105 |
| 104. | Miele C, Taylor M, Shah A. Assessment of direct oral anticoagulant prescribing and monitoring pre- and post-implementation of a pharmacy protocol at a community teaching hospital. Hospital Pharmacy 2017;52:207–13. doi:10.1310/hpj5203-207 |
| 105. | Morello Candis M, Rotunno Taylor, Khoan John, et al. Improved Glycemic Control With Minimal Change in Medication Regimen Complexity in a Pharmacist-Endocrinologist Diabetes Intense Medical Management (DIMM) ‘Tune Up’ Clinic. The Annals of pharmacotherapy 2018;52:1091–7. doi:10.1177/1060028018776663 |
| 106. | Morgado Manuel, Rolo Sandra, Castelo-Branco Miguel. Pharmacist intervention program to enhance hypertension control: a randomised controlled trial. International journal of clinical pharmacy 2011;33:132–40. doi:10.1007/s11096-010-9474-x |
| 107. | Nagy Michael W, Gruber Stephanie, McConnell Macy. Implementation of a Pilot Pharmacist Testosterone Therapy Management Service. Journal of pharmacy practice 2020;33:654–60. doi:10.1177/0897190019830251 |
| 108. | Neto Paulo Roque Obreli, Marusic Srecko, de Lyra Júnior Divaldo Pereira, et al. Effect of a 36-month pharmaceutical care program on the coronary heart disease risk in elderly diabetic and hypertensive patients. Journal of pharmacy & pharmaceutical sciences : a publication of the Canadian Society for Pharmaceutical Sciences, Societe canadienne des sciences pharmaceutiques 2011;14:249–63. doi:10.18433/j3259q |
| 109. | Nguyen Anny D, Lam Alice, Banakh Iouri, et al. Improved Medication Management With Introduction of a Perioperative and Prescribing Pharmacist Service. Journal of pharmacy practice 2020;33:299–305. doi:10.1177/0897190018804961 |

| Excluded on intervention (n=182) | |
|---|---|
| 110. | Nguyen Tan Thanh, Truong Mai Thi Xuan, Lam Dung Ngoc, et al. Effect of Pharmacist-Led Interventions on Medication Adherence among Vietnamese Patients with Asthma: A Randomized Controlled Trial. Advances in respiratory medicine 2023;91:254–67. doi:10.3390/arm91030020 |
| 111. | Nola KM, Gourley DR, Portner TS, et al. Clinical and Humanistic Outcomes of a Lipid Management Program in the Community Pharmacy Setting. Journal of the American Pharmaceutical Association (1996) 2000;40:166–73. doi:10.1016/S1086-5802(16)31060-9 |
| 112. | Noor Afnan, Khan Mansoor A, Warsi Ashraf, et al. Evaluation of a pharmacist vs. Haematologist-managed anticoagulation clinic: A retrospective cohort study. Saudi pharmaceutical journal : SPJ : the official publication of the Saudi Pharmaceutical Society 2021;29:1173–80. doi:10.1016/j.jsps.2021.08.015 |
| 113. | Norton Melissa C, Haftman Meghan E, Buzzard Lyndsey N. Impact of Physician-Pharmacist Collaboration on Diabetes Outcomes and Health Care Use. Journal of the American Board of Family Medicine : JABFM 2020;33:745–53. doi:10.3122/jabfm.2020.05.200044 |
| 114. | Obarcanin Emina, Krüger Manfred, Müller Petra, et al. PHARMACEUTICAL CARE OF ADOLESCENTS WITH DIABETES MELLITUS TYPE 1: THE DIADEMA STUDY, A RANDOMIZED CONTROLLED TRIAL. International journal of clinical pharmacy 2015;37:790–8. doi:10.1007/s11096-015-0122-3 |
| 115. | Odeh Mohanad, Scullin Claire, Hogg Anita, et al. A novel approach to medicines optimisation post-discharge from hospital: pharmacist-led medicines optimisation clinic. International journal of clinical pharmacy 2020;42:1036–49. doi:10.1007/s11096-020-01059-4 |
| 116. | Okamoto Mark P, Nakahiro Randall K. Pharmacoeconomic evaluation of a pharmacist-managed hypertension clinic. Pharmacotherapy 2001;21:1337–44. doi:10.1592/phco.21.17.1337.34424 |
| 117. | Okere Arinze Nkemdirim, Renier Colleen M, Willemstein Megan. Comparison of a pharmacist-hospitalist collaborative model of inpatient care with multidisciplinary rounds in achieving quality measures. American journal of health-system pharmacy : AJHP : official journal of the American Society of Health-System Pharmacists 2016;73:216–24. doi:10.2146/ajhp150225 |
| 118. | Olesen Charlotte, Harbig Philipp, Buus Kirsten Marie, et al. Impact of pharmaceutical care on adherence, hospitalisations and mortality in elderly patients. International journal of clinical pharmacy 2013;36:163–71. doi:10.1007/s11096-013-9898-1 |
| 119. | Omran Dima, Majumdar Sumit R, Johnson Jeffrey A, et al. Pharmacists on primary care teams: Effect on antihypertensive medication management in patients with type 2 diabetes. Journal of the American Pharmacists Association : JAPhA 2015;55:265–8. doi:10.1331/japha.2015.14225 |
| 120. | Ortiz Jasmin, Rasch Megan. Assessing the proportion of patients with hepatitis C treated before and after initiation of an ambulatory pharmacist-led hepatitis C program: A retrospective analysis. Journal of the American Pharmacists Association : JAPhA 2023;63:440–6. doi:10.1016/j.japh.2022.09.006 |
| 121. | Pals Haley, Bratberg Jeffrey. Improving access to care via psychiatric clinical pharmacist practitioner collaborative management of buprenorphine for opioid use disorder. Journal of the American Pharmacists Association : JAPhA 2022;62:1422–9. doi:10.1016/j.japh.2022.03.006 |
| 122. | Papastergiou John, Trieu Chantal Rene, Saltmarche Deborah, et al. Community pharmacist-directed point-of-care group A Streptococcus testing: Evaluation of a Canadian program. Journal of the American Pharmacists Association : JAPhA 2018;58:450–6. doi:10.1016/j.japh.2018.03.003 |

| Excluded on intervention (n=182) | |
|---|---|
| 123. | Pape Ginger A, Hunt Jacquelyn S, Butler Kristina L, et al. Team-based care approach to cholesterol management in diabetes mellitus: two-year cluster randomized controlled trial. Archives of internal medicine 2011;171:1480–6. doi:10.1001/archinternmed.2011.417 |
| 124. | Peek Niels, Gude Wouter T, Keers Richard N, et al. Evaluation of a pharmacist-led actionable audit and feedback intervention for improving medication safety in UK primary care: An interrupted time series analysis. PLoS medicine 2020;17:e1003286. doi:10.1371/journal.pmed.1003286 |
| 125. | Peterson Jasmine, Hinds April, Garza Aida, et al. Impact of Physician-Pharmacist Covisits at a Primary Care Clinic in Patients With Uncontrolled Diabetes. Journal of pharmacy practice 2020;33:321–5. doi:10.1177/0897190018807374 |
| 126. | Planas Lourdes G, Crosby Kimberly M, Farmer Kevin C, et al. Evaluation of a diabetes management program using selected HEDIS measures. Journal of the American Pharmacists Association : JAPhA 2012;52:e130-8. doi:10.1331/japha.2012.11148 |
| 127. | Price-Haywood Eboni G, Amering Sarah, Luo Qingyang, et al. Clinical Pharmacist Team-Based Care in a Safety Net Medical Home: Facilitators and Barriers to Chronic Care Management. Population health management 2017;20:123–31. doi:10.1089/pop.2015.0177 |
| 128. | Prudencio J, Cajudoy P, Waddell D. Optimization of medication regimens in patients with type 2 diabetes and clinical atherosclerotic cardiovascular disease. Pharmacy 2021;9:186. doi:10.3390/pharmacy9040186 |
| 129. | Qudah Bonyan, Albsoul-Younes Abla, Alawa Ezat, et al. Role of clinical pharmacist in the management of blood pressure in dialysis patients. International journal of clinical pharmacy 2016;38:931–40. doi:10.1007/s11096-016-0317-2 |
| 130. | Rebolledo Julio A, Rhodes Nathaniel J, Valdes Angeles M, et al. Implementation of a clinical pharmacist-driven comprehensive medication management program in an outpatient wound healing center. Journal of the American Pharmacists Association : JAPhA 2022;62:475. doi:10.1016/j.japh.2021.10.021 |
| 131. | Rizvi A, Harrison M. The impact of a medicines administration pharmacist on patient safety and medicines management: a service evaluation. Pharmaceutical Journal 2021;307. doi:10.1211/PJ.2021.1.102600 |
| 132. | Roman Cristina, Dooley Michael, Fitzgerald Mark, et al. Pharmacists in Trauma: a randomised controlled trial of emergency medicine pharmacists in trauma response teams. Emergency medicine journal : EMJ 2024;41:397–403. doi:10.1136/emermed-2022-212934 |
| 133. | Rose Anne E, Robinson Erin N, Premo Joan A, et al. Improving Warfarin Management Within the Medical Home: A Health-System Approach. The American Journal of Medicine 2016;130:3–5. |
| 134. | Rudd Kelly M, Dier John G. Comparison of two different models of anticoagulation management services with usual medical care. Pharmacotherapy 2010;30:330–8. doi:10.1592/phco.30.4.330 |
| 135. | Sadik A, Yousif M, McElnay J C. Pharmaceutical care of patients with heart failure. British journal of clinical pharmacology 2005;60:183–93. doi:10.1111/j.1365-2125.2005.02387.x |
| 136. | Saklad S R, Ereshefsky L, Jann M W, et al. Clinical pharmacists' impact on prescribing in an acute adult psychiatric facility. Drug intelligence & clinical pharmacy 1984;18:632–4. doi:10.1177/106002808401800718 |

| Excluded on intervention (n=182) | |
|---|---|
| 137. | Sakthong Phantipa, Sangthonganotai Todsaporn. A randomized controlled trial of the impact of pharmacist-led patient-centered pharmaceutical care on patients' medicine therapy-related quality of life. Research in social & administrative pharmacy : RSAP 2018;14:332–9. doi:10.1016/j.sapharm.2017.05.001 |
| 138. | Sakthong Phantipa, Soipitak Porntip, Winit-Watjana Win. Comparison of the sensitivities of pharmacotherapy-related and disease-specific quality of life measures in response to pharmacist-led pharmaceutical care for cancer outpatients: a randomised controlled trial. International journal of clinical pharmacy 2024;46:463–70. doi:10.1007/s11096-023-01692-9 |
| 139. | Sakthong P, Boonyanuwat W. Impact of pharmacist-led pharmaceutical care on health-related and pharmaceutical therapy-related quality of life in patients with heart failure: A randomized controlled trial. Research in Social and Administrative Pharmacy 2024;20:1058–63. doi:10.1016/j.sapharm.2024.08.003 |
| 140. | Saya F G, Coleman L T, Martinoff J T. Pharmacist-directed heparin therapy using a standard dosing and monitoring protocol. American journal of hospital pharmacy 1985;42:1965–9. |
| 141. | Schillig Jessica, Kaatz Scott, Hudson Michael P, et al. Clinical and safety impact of an inpatient pharmacist-directed anticoagulation service. Journal of hospital medicine 2011;6:322–8. doi:10.1002/jhm.910 |
| 142. | Schneiderhan Mark E, Shuster Sara M, Davey Cynthia S. Twelve-month prospective randomized study of pharmacists utilizing point-of-care testing for metabolic syndrome and related conditions in subjects prescribed antipsychotics. The primary care companion for CNS disorders 2014;16:0–0. doi:10.4088/pcc.14m01669 |
| 143. | Schommer J C, Mott D A, Schneider P J, et al. Performance measurement of a pharmacist-directed anticoagulation management service. Hospital Pharmacy 2001;36:1164–9. doi:10.1177/001857870103601106 |
| 144. | Scott David M, Boyd Steven T, Stephan Michelle, et al. Outcomes of pharmacist-managed diabetes care services in a community health center. American journal of health-system pharmacy : AJHP : official journal of the American Society of Health-System Pharmacists 2006;63:2116–22. doi:10.2146/ajhp060040 |
| 145. | Sease Julie M, Blake Elizabeth W, Gowan Mollie, et al. Evaluation of Anticoagulation Management and Chronic Disease State Control in a Pharmacist-Run Pharmacotherapy/Anticoagulation Clinic. Journal of Pharmacy Technology 2011;27:3–8. doi:10.1177/875512251102700102 |
| 146. | Shao Hua, Chen Guoming, Zhu Chao, et al. Effect of pharmaceutical care on clinical outcomes of outpatients with type 2 diabetes mellitus. Patient preference and adherence 2017;11:897–903. doi:10.2147/ppa.s92533 |
| 147. | Shim Yee Wei, Chua Siew Siang, Wong Hui Chin, et al. Collaborative intervention between pharmacists and physicians on elderly patients: a randomized controlled trial. Therapeutics and clinical risk management 2018;14:1115–25. doi:10.2147/TCRM.S146218 |

| Excluded on intervention (n=182) | |
|---|---|
| 148. | Siaw M Y. L, Ko Y, Malone D C, et al. Impact of pharmacist-involved collaborative care on the clinical, humanistic and cost outcomes of high-risk patients with type 2 diabetes (IMPACT): a randomized controlled trial. Journal of clinical pharmacy and therapeutics 2017;42:475–82. doi:10.1111/jcpt.12536 |
| 149. | Smith Sarah, Porteous Terry, Bond Christine, et al. The Help for Hay Fever community pharmacy-based pilot randomised controlled trial for intermittent allergic rhinitis. NPJ primary care respiratory medicine 2020;30:23. doi:10.1038/s41533-020-0180-4 |
| 150. | Smith Steven M, Carris Nicholas W, Dietrich Eric, et al. Physician-pharmacist collaboration versus usual care for treatment-resistant hypertension. Journal of the American Society of Hypertension : JASH 2016;10:307–17. doi:10.1016/j.jash.2016.01.010 |
| 151. | Song Y K, Jeong S, Han N, et al. Effectiveness of clinical pharmacist service on drug-related problems and patient outcomes for hospitalized patients with chronic kidney disease: A randomized controlled trial. Journal of Clinical Medicine 2021;10:1788. doi:10.3390/jcm10081788 |
| 152. | Stone Rachel, Oganesyan Aida, Marco Noachim, et al. The Impact of a Pharmacist-Led Hypertension Medication Management Program on Older People in a Skilled Nursing Facility. The Senior care pharmacist 2022;37:62–72. doi:10.4140/TCP.n.2022.62 |
| 153. | Straka Robert J, Taheri Reza, Cooper Susan L, et al. Achieving cholesterol target in a managed care organization (ACTION) trial. Pharmacotherapy 2005;25:360–71. doi:10.1592/phco.25.3.360.61601 |
| 154. | Swearingen Sara M, White Cyle, Weidert Sara, et al. A multidimensional antimicrobial stewardship intervention targeting aztreonam use in patients with a reported penicillin allergy. International journal of clinical pharmacy 2016;38:213–7. doi:10.1007/s11096-016-0248-y |
| 155. | Sweiss Karen, Calip Gregory S, Wirth Scott, et al. Polypharmacy and potentially inappropriate medication use is highly prevalent in multiple myeloma patients and is improved by a collaborative physician-pharmacist clinic. Journal of oncology pharmacy practice : official publication of the International Society of Oncology Pharmacy Practitioners 2020;26:536–42. doi:10.1177/1078155219851550 |
| 156. | Tadesse Tamrat Assefa, Gebremedhin Amha, Yadeta Dejuma, et al. Comparison of anticoagulation control and outcomes between usual medical care and pharmacist-led anticoagulation service in ambulatory patients taking warfarin at tertiary hospital in Ethiopia: a quasi-experimental study. Journal of pharmaceutical health care and sciences 2024;10:32-NA. doi:10.1186/s40780-024-00355-9 |
| 157. | Taveira Tracey H, Friedmann Peter D, Cohen Lisa B, et al. Pharmacist-led group medical appointment model in type 2 diabetes. The Diabetes educator 2010;36:109–17. doi:10.1177/0145721709352383 |
| 158. | Teichert Martina, Griens Fabienne, Buijs Edgar, et al. Effectiveness of interventions by community pharmacists to reduce risk of gastrointestinal side effects in nonselective nonsteroidal anti-inflammatory drug users. Pharmacoepidemiology and drug safety 2014;23:382–9. doi:10.1002/pds.3587 |
| 159. | Tian Jiao, Wang Meng-Meng, Jiang Xun, et al. Effect of pharmacist interventions on antibiotic use in the general pediatric ward. Pakistan journal of pharmaceutical sciences 2020;33:1389–95. |
| 160. | Tran T, Taylor S E, Hardidge A, et al. Impact of pharmacists assisting with prescribing and undertaking medication review on oxycodone prescribing and supply for patients discharged from surgical wards. Journal of clinical pharmacy and therapeutics 2017;42:567–72. doi:10.1111/jcpt.12540 |

| Excluded on intervention (n=182) | |
|---|---|
| 161. | Tran T, Taylor S E, Hardidge A, et al. Pharmacist-assisted electronic prescribing at the time of admission to an inpatient orthopaedic unit and its impact on medication errors: a pre- and postintervention study. Therapeutic Advances in Drug Safety 2019;10:1–10. doi:10.1177/2042098619863985 |
| 162. | Tse Stacy Saithy, Sands Bruce E, Keefer Laurie, et al. Improved Smoking Cessation Rates in a Pharmacist-Led Program Embedded in an Inflammatory Bowel Disease Specialty Medical Home. Journal of pharmacy practice 2022;35:827–35. doi:10.1177/08971900211000682 |
| 163. | Vastrad M, Wali S, Ganachari M S, et al. Clinical pharmacist approach towards effectiveness of pulmonary rehabilitation of the patient with COPD: A randomized controlled study. Indian Journal of Pharmaceutical Education and Research 2021;55:S318–24. doi:10.5530/ijper.55.1s.65 |
| 164. | Weber Cynthia A, Ernst Michael E, Sezate Genesis S, et al. Pharmacist-physician comanagement of hypertension and reduction in 24-hour ambulatory blood pressures. Archives of internal medicine 2010;170:1634–9. doi:10.1001/archinternmed.2010.349 |
| 165. | Weeks G R, Fyfe R, Amerena J, et al. Hospital pharmacist-led lipid clinic for surgical patients with peripheral vascular disease at a regional Australian hospital. Journal of Pharmacy Practice and Research 2012;42:17–21. doi:10.1002/j.2055-2335.2012.tb00124.x |
| 166. | Wei Erin T, Gregory Patrick, Halpern David J, et al. Impact of a clinical pharmacist on provider prescribing patterns in a primary care clinic. Journal of the American Pharmacists Association : JAPhA 2022;62:209. doi:10.1016/j.japh.2021.10.007 |
| 167. | Wentzlaff Danielle M, Carter Barry L, Ardery Gail, et al. Sustained blood pressure control following discontinuation of a pharmacist intervention. Journal of clinical hypertension (Greenwich, Conn) 2011;13:431–7. doi:10.1111/j.1751-7176.2011.00435.x |
| 168. | Willeford A, Leiman V, Noel Z R. Impact of a pharmacist-to-dose direct oral anticoagulant protocol on medication errors at an academic medical center. JACCP Journal of the American College of Clinical Pharmacy 2021;4:1392–400. doi:10.1002/jac5.1503 |
| 169. | Wilson S Jo-Anne, Wells Philip S, Kovacs Michael J, et al. Comparing the quality of oral anticoagulant management by anticoagulation clinics and by family physicians: a randomized controlled trial. CMAJ : Canadian Medical Association journal = journal de l’Association medicale canadienne 2003;169:293–8. |
| 170. | Wilt Vickie M, Gums John G, Ahmed Osman I, et al. Outcome Analysis of a Pharmacist-Managed Anticoagulation Service. Pharmacotherapy: The Journal of Human Pharmacology and Drug Therapy 1995;15:732–9. doi:10.1002/j.1875-9114.1995.tb02889.x |
| 171. | Wong Y M, Quek Y N, Tay J C, et al. Efficacy and safety of a pharmacist-managed inpatient anticoagulation service for warfarin initiation and titration. Journal of clinical pharmacy and therapeutics 2010;36:585–91. doi:10.1111/j.1365-2710.2010.01216.x |
| 172. | Wu Chia-Wei, Wu Chien-Chih, Chen Chien-Hao, et al. The Impact of Pharmacist-Managed Service on Warfarin Therapy in Patients after Mechanical Valve Replacement. International journal of clinical practice 2022;2022:1617135–6. doi:10.1155/2022/1617135 |
| 173. | Wu Wen-Chih, Taveira Tracey H, Jeffery Sean, et al. Costs and effectiveness of pharmacist-led group medical visits for type-2 diabetes: A multi-center randomized controlled trial. PloS one 2018;13:e0195898. doi:10.1371/journal.pone.0195898 |

| Excluded on intervention (n=182) | |
|---|---|
| 174. | Xiao J, Wang Q, Tan S, et al. Analysis of patient medication compliance and quality of life of physician-pharmacist collaborative clinics for T2DM management in primary healthcare in China: A mixed-methods study. Frontiers in Pharmacology 2023;14:1098207. doi:10.3389/fphar.2023.1098207 |
| 175. | Xin C, Xia Z, Jiang C, et al. Effect of pharmaceutical care on medication adherence of patients newly prescribed insulin therapy: A randomized controlled study. Patient Preference and Adherence 2015;9:797–802. doi:10.2147/PPA.S84411 |
| 176. | Xuan Si, Colayco Danielle, Hashimoto Jonathan, et al. Impact of Adding Pharmacists and Comprehensive Medication Management to a Medical Group's Transition of Care Services. Medical care 2021;59:519–27. doi:10.1097/MLR.0000000000001520 |
| 177. | Yang Qiong, He Junhong, Yuan Fangfang. Improvement of proportion of days covered for denosumab under implementation of clinical pharmacist adherence management system: normal and COVID-19 period. Osteoporosis international : a journal established as result of cooperation between the European Foundation for Osteoporosis and the National Osteoporosis Foundation of the USA 2024;35:309–16. doi:10.1007/s00198-023-06933-1 |
| 178. | Zhang Hai-Xia, Li Xin, Huo Hai-Qin, et al. Pharmacist interventions for prophylactic antibiotic use in urological inpatients undergoing clean or clean-contaminated operations in a Chinese hospital. PloS one 2014;9:e88971. doi:10.1371/journal.pone.0088971 |
| 179. | Zhang Le, Geng Shikai, Qian Liping, et al. Multidisciplinary care in patients with systemic lupus erythematosus: a randomized controlled trial in China. International journal of clinical pharmacy 2019;41:1247–55. doi:10.1007/s11096-019-00870-y |
| 180. | Zheng Xiaowei, Ding Haiying, Xu Silu, et al. Pharmacist-Led Management Improves Treatment Adherence and Quality of Life in Opioid-Tolerant Patients With Cancer Pain: A Randomized Controlled Trial. Pain and therapy 2022;11:241–52. doi:10.1007/s40122-021-00342-0 |
| 181. | Zhou Ling, Ma Jingjing, Gao Jie, et al. Optimizing Prophylactic Antibiotic Practice for Cardiothoracic Surgery by Pharmacists' Effects. Medicine 2016;95:e2753. doi:10.1097/MD.0000000000002753 |
| 182. | Zhou Xindie, Gong Jinhong, Su Dan, et al. Effect of pharmacist intervention on antibiotic prophylaxis in orthopedic internal fixation: A retrospective study. Research in social & administrative pharmacy : RSAP 2023;19:301–7. doi:10.1016/j.sapharm.2022.10.002 |

| Excluded on comparator (n=29) | |
|---|---|
| 1. | Al Hamarneh YN; H Brenda R; Hassan, Imran; Jones, Charlotte; Tsuyuki, Ross T. The Effectiveness of Pharmacist Interventions on Cardiovascular Risk in Adult Patients with Type 2 Diabetes: The Multicentre Randomized Controlled RxEACH Trial. Canadian journal of diabetes 2017;41:580–6. doi:10.1016/j.jcjd.2017.08.244 |
| 2. | Al Hamarneh YN; M Carlo A; Gniadecki, Robert; Keeling, Stephanie; Morgan, Andrea; Tsuyuki, Ross T. RxIALTA: evaluating the effect of a pharmacist-led intervention on CV risk in patients with |

| Excluded on comparator (n=29) | |
|---|---|
| | chronic inflammatory diseases in a community pharmacy setting: a prospective pre-post intervention study. BMJ open 2021;11:e043612-NA. doi:10.1136/bmjopen-2020-043612 |
| 3. | Al Hamarneh Y, Tsuyuki R, Jones C, et al. Effectiveness of Pharmacist Interventions on Cardiovascular Risk in Patients With CKD: a Subgroup Analysis of the Randomized Controlled RxEACH Trial. American journal of kidney diseases 2018;71:42-51. doi:10.1053/j.ajkd.2017.07.012 |
| 4. | Alotaibi MSA Ali F; Alharbi, Abdulmajeed S; Alfaraj, Lulwa A; Alenazi, Maram A; Alkhrshawy, Fahad F. Implementation and Outcome of Clinical Pharmacist-led Anticoagulation Clinic at Cardiac Center: A Retrospective Cohort Study. Saudi Journal of Clinical Pharmacy 2024;3:154–61. doi:10.4103/sjcp.sjcp_3_24 |
| 5. | Bhat Shubha, Nunes David. Pharmacist-Managed Helicobacter pylori Treatment Service Within a Gastroenterology Clinic: Workflow and Real-World Experiences. The Annals of pharmacotherapy 2022;56:162–9. doi:10.1177/10600280211021501 |
| 6. | Bomkamp John P, Isom Caleb, Wells Makayla L. EVALPAX: Evaluation of a pharmacist-driven protocol for nirmatrelvir/ritonavir prescribing in a community hospital system. American journal of health-system pharmacy : AJHP : official journal of the American Society of Health-System Pharmacists 2025;82:65–72. doi:10.1093/ajhp/zxae211 |
| 7. | Carter B, Vander Weg M, Parker C, et al. Sustained Blood Pressure Control Following Discontinuation of a Pharmacist Intervention for Veterans. Journal of clinical hypertension (Greenwich, Conn) 2015;17:701-708. doi:10.1111/jch.12577 |
| 8. | Cross V J, Parker J T, Law Min, et al. Pharmacist prescribing in critical care: An evaluation of the introduction of pharmacist prescribing in a single large UK teaching hospital. European Journal of Hospital Pharmacy 2018;25:E2–6. doi:10.1136/ejhpharm-2017-001267 |

| Excluded on comparator (n=29) | |
|---|---|
| 9. | Dolder Nicole M, Dolder Christian R. Comparison of a pharmacist-managed lipid clinic: in-person versus telephone. Journal of the American Pharmacists Association : JAPhA 2010;50:375–8. doi:10.1331/JAPhA.2010.09048 |
| 10. | Farland MZ; B Debbie C; McFarland, M Shawn; Thomas, Jeremy; Franks, Andrea S; George, Christa M; Gross, Benjamin N; Guirguis, Alexander B; Suda, Katie J. Pharmacist-Physician Collaboration for Diabetes Care: The Diabetes Initiative Program. The Annals of pharmacotherapy 2013;47:781–9. doi:10.1345/aph.1s079 |
| 11. | Garton LC Joseph F. A retrospective assessment comparing pharmacist-managed anticoagulation clinic with physician management using international normalized ratio stability. Journal of thrombosis and thrombolysis 2011;32:426–30. doi:10.1007/s11239-011-0612-7 |
| 12. | Hale GMH Sonia L; Hummel, Scott L; Lewis, Carrie; Ratz, David; Brenner, Michael. Impact of a Pharmacist-Managed Heart Failure Postdischarge (Bridge) Clinic for Veterans: The Annals of pharmacotherapy 2017;51:555–62. doi:10.1177/1060028017698974 |
| 13. | Ko John J, Lu Jackie, Rascati Karen, et al. Analysis of Glycemic Control of a Pharmacist-Led Medication Management Program in Patients with Type 2 Diabetes. Journal of managed care & specialty pharmacy 2016;22:32–7. doi:10.18553/jmcp.2016.22.1.32 |
| 14. | Kose E, Endo H, Hori H, et al. Association of Pharmacist-led Deprescribing Intervention with the Functional Recovery in Convalescent Setting. Die Pharmazie 2022;77:165–70. doi:10.1691/ph.2022.2323 |
| 15. | Leef George C, Perino Alexander C, Askari Mariam, et al. Appropriateness of Direct Oral Anticoagulant Dosing in Patients With Atrial Fibrillation: Insights From the Veterans Health Administration. Journal of pharmacy practice 2020;33:647–53. doi:10.1177/0897190019828270 |
| 16. | Lewis Jelena, Nguyen Tiffany, Althobaiti Hana, et al. Impact of an Advanced Practice Pharmacist Type 2 Diabetes Management Program: A Pilot Study. Innovations in pharmacy 2019;10. doi:10.24926/iip.v10i4.2237 |

| Excluded on comparator (n=29) | |
|---|---|
| 17. | Lin Hsiang-Wen, Lin Chih-Hsueh, Chang Chin-Kai, et al. Economic outcomes of pharmacist-physician medication therapy management for polypharmacy elderly: A prospective, randomized, controlled trial. Journal of the Formosan Medical Association = Taiwan yi zhi 2018;117:235–43. doi:10.1016/j.jfma.2017.04.017 |
| 18. | McGowan Neil, Cockburn Alison, Strachan Mark W. J, et al. Initial and sustained cardiovascular risk reduction in a pharmacist-led diabetes cardiovascular risk clinic. The British Journal of Diabetes & Vascular Disease 2008;8:34–8. doi:10.1177/14746514080080010801 |
| 19. | Morello Candis M, Nguyen Tran, Tao Lillian, et al. Improved Glycemic Control Outcomes Regardless of Mental Health Disorders in a Pharmacist-Endocrinologist Diabetes Intense Medical Management (DIMM) 'Tune Up' Clinic. The Annals of pharmacotherapy 2020;54:858–65. doi:10.1177/1060028020908856 |
| 20. | Radley Andrew, Hall J, Farrow M, et al. Evaluation of anticoagulant control in a pharmacist operated anticoagulant clinic. Journal of clinical pathology 1995;48:545–7. doi:10.1136/jcp.48.6.545 |
| 21. | Reid Fiona, Murray Pat, Storrie Marion. Implementation of a pharmacist-led clinic for hypertensive patients in primary care--a pilot study. Pharmacy world & science : PWS 2005;27:202–7. doi:10.1007/s11096-004-2563-y |
| 22. | Starikova Svetlana, Castelvecchi Ashley, Corboy Alexander. Evaluation of Diabetes Mellitus Type 2 Control in Home-Based Primary Care Patients Managed by Clinical Pharmacy Specialists. The Senior care pharmacist 2022;37:366–73. doi:10.4140/TCP.n.2022.366 |
| 23. | Tsuyuki R T, Rosenthal M, Pearson G J. A randomized trial of a community-based approach to dyslipidemia management: Pharmacist prescribing to achieve cholesterol targets (RxACT Study). Canadian Pharmacists Journal 2016;149:283–92. doi:10.1177/1715163516662291 |

| Excluded on comparator (n=29) | |
|---|---|
| 24. | Tsuyuki Ross T, Al Hamarneh Yazid N, Jones Charlotte A, et al. The Effectiveness of Pharmacist Interventions on Cardiovascular Risk: The Multicenter Randomized Controlled RxEACH Trial. Journal of the American College of Cardiology 2016;67:2846–54. doi:10.1016/j.jacc.2016.03.528 |
| 25. | Tsuyuki Ross T, Houle Sherilyn K. D, Charrois Theresa L, et al. Randomized Trial of the Effect of Pharmacist Prescribing on Improving Blood Pressure in the Community: The Alberta Clinical Trial in Optimizing Hypertension (RxACTION). Circulation 2015;132:93–100. doi:10.1161/CIRCULATIONAHA.115.015464 |
| 26. | Warrington LA Phil; Baldwin, Anna Marie; Wallace, Virginia; Riche, Krista D; Saulters, Robert; Waldrop, Oliver Grey; Dyess, Teri; Delashmet, Gordon Bart; Peeples, Samuel; Horsley, W Stewart; Harris, William J; Butler, Kenneth R. Implementation of a pharmacist-led, multidisciplinary diabetes management team. American journal of health-system pharmacy : AJHP : official journal of the American Society of Health-System Pharmacists 2012;69:1240–5. doi:10.2146/ajhp110297 |
| 27. | Weschules DJ; M Terni; Reifsnyder, JoAnne; Knowlton, Calvin H. Are newer, more expensive pharmacotherapy options associated with superior symptom control compared to less costly agents used in a collaborative practice setting?: The American journal of hospice & palliative care 2006;23:135–49. doi:10.1177/104990910602300211 |
| 28. | Wooley AC; B Amie D; Stacy, Zachary A. Effect of a Clinical Pharmacist–Managed Service on Blood Pressure in an Underserved Population With Resistant Hypertension. Journal of Pharmacy Technology 2016;32:135–42. doi:10.1177/8755122515624221 |
| 29. | Zed PJ; F Lyne. Clinical outcomes and patient satisfaction of a pharmacist-managed, emergency department-based outpatient treatment program for venous thromboembolic disease. CJEM 2008;10:10–7. doi:10.1017/s1481803500009957 |

| Excluded on outcome (n=4) | |
|---|---|
| 1. | Philips Z, Whynes D, Parnham S, et al. The role of community pharmacists in prescribing medication for the treatment of head lice. Journal of public health medicine 2001;23:114–20. doi:10.1093/pubmed/23.2.114 |
| 2. | Ballister BH Rebecca L; Quffa, Lieth H; Franck, Andrew J. Clinical Pharmacy Specialist Collaborative Management and Prescription of Diabetes Medications with Cardiovascular Benefit. Journal of pharmacy practice 2022;37:435–41. doi:10.1177/08971900221144399 |
| 3. | Boudreau DM, Capoccia K, Stevens NG. Collaborative Care Model to Improve Outcomes in Major Depression. The Annals of Pharmacotherapy 2002;36:585–91. doi:10.1345/aph.1a259 |
| 4. | Campbell Gayle, Razouk Roula, Auyeung Vivian, et al. Evaluation of the impact of the addition of a heart failure prescribing pharmacist to consultant-led heart failure ward round at a tertiary hospital. European journal of hospital pharmacy : science and practice 2023;30:e14–8. doi:10.1136/ejhpharm-2021-002869 |

| Excluded on duplicate (n=1) | |
|---|---|
| 1. | Varghese CJ, Grunske M, Nagy MW. Implementation of a Pharmacist-Driven Aspirin Deprescribing Protocol Among Older Veterans in a Primary Care Setting. *The Senior care pharmacist* 2024;39:228–34. doi:10.4140/tcp.n.2024.228 |

| Excluded on study design (n=56) | |
|---|---|
| 1. | Anonymous. Pharmacological treatment of frailty in the elderly. Australian Journal of Pharmacy 2010;91:56–8. |
| 2. | Asai Yuki, Yanagawa Tatsuki, Takahashi Masaaki. Effect of pharmacist-led intervention protocol on preventing postoperative delirium after elective cardiovascular surgery. PloS one 2023;18:e0292786. doi:10.1371/journal.pone.0292786 |
| 3. | Baqir Wasim, Miller David, Richardson Graeme. A brief history of pharmacist prescribing in the UK. European Journal of Hospital Pharmacy: Science and Practice 2012;19:487–8. doi:10.1136/ejhpharm-2012-000189 |
| 4. | Beahm Nathan P, Smyth Daniel, Tsuyuki Ross T. 200. Pharmacist Prescribing and Care for Patients with Uncomplicated Urinary Tract Infections in the Community: Antimicrobial Utilization and Stewardship Results of the RxOUTMAP Study. Open Forum Infectious Diseases 2018;5:S87–S87. doi:10.1093/ofid/ofy210.213 |

| Excluded on study design (n=56) | |
|---|---|
| 5. | Bishop Martin A, Streiff Michael B, Ensor Christopher R, et al. Pharmacist-managed international normalized ratio patient self-testing is associated with increased time in therapeutic range in patients with left ventricular assist devices at an academic medical center. ASAIO journal (American Society for Artificial Internal Organs : 1992) 2014;60:193–8. doi:10.1097/mat.0000000000000047 |
| 6. | Bond C A, Raehl Cynthia L. Clinical and economic outcomes of pharmacist-managed aminoglycoside or vancomycin therapy. American Journal of Health-System Pharmacy 2005;62:1596–605. doi:10.2146/ajhp040555 |
| 7. | Boren Lacey L, Locke Amanda M, Friedman Andrew S, et al. Team-Based Medicine: Incorporating a Clinical Pharmacist into Pain and Opioid Practice Management. PM & R : the journal of injury, function, and rehabilitation 2019;11:1170–7. doi:10.1002/pmrj.12127 |
| 8. | Carter Barry L, Malone Daniel C, Billups Sarah J, et al. Interpreting the findings of the IMPROVE study. American journal of health-system pharmacy : AJHP : official journal of the American Society of Health-System Pharmacists 2001;58:1330–7. doi:10.1093/ajhp/58.14.1330 |
| 9. | Carter Mary, Chapman Sarah, Rogers Philip, et al. Practice pharmacists and their influence on prescribing in UK general practice: a cross-sectional study. International Journal of Pharmacy Practice 2024;32:69–75. doi:10.1093/ijpp/riad075 |
| 10. | Chau Tom, Rotbard Morris, King Sharon, et al. Implementation and Evaluation of a Warfarin Dosing Service for Rehabilitation Medicine: Report from a Pilot Project. The Canadian Journal of Hospital Pharmacy 2006;59:NA-NA. |
| 11. | Chonko K, Axtell S, Mayzel B. Pharmacist Hypertension Management Quality Review at an Ambulatory Care Clinic. Journal of Pharmacy Technology 2022;38:31–8. doi:10.1177/87551225211064240 |
| 12. | Cooper R J, Guillaume L, Avery A, et al. The safety climate of pharmacist supplementary prescribing in England [poster]. International Journal of Pharmacy Practice 2008;16:A21. |
| 13. | Cordero L, Fernandez-Llimos F, Cadavid M I, et al. Protocols for minor ailments of the TESEMED project: Haemorrhoids. Pharmaceutical Care Espana 2001;3:324–36. |
| 14. | Do Vincent, Haakinson Danielle, Belfort-DeAguiar Renata, et al. Implementing a pharmacist-led transition of care model for posttransplant hyperglycemia. American journal of health-system pharmacy : AJHP : official journal of the American Society of Health-System Pharmacists 2021;78:1207–15. doi:10.1093/ajhp/zxab151 |
| 15. | Donoghue K, Boniface S, Brobbin E, et al. Adjunctive Medication Management and Contingency Management to enhance adherence to acamprosate for alcohol dependence: the ADAM trial RCT. Health Technology Assessment 2023;27. doi:10.3310/DQKL6124 |
| 16. | Fisher J, Kinnear M, Reid F, et al. What supports hospital pharmacist prescribing in Scotland? – A mixed methods, exploratory sequential study. Research in Social and Administrative Pharmacy 2018;14:488–97. doi:10.1016/j.sapharm.2017.06.007 |
| 17. | Furniss L. Use of medicines in nursing homes for older people. Advances in Psychiatric Treatment 2002;8:198–203. doi:10.1192/apt.8.3.198 |

| Excluded on study design (n=56) | |
|---|---|
| 18. | Gupta Vasudha, Kogut Stephen J, Thompson Sarah. Evaluation of differences in percentage of international normalized ratios in range between pharmacist-led and physician-led anticoagulation management services. Journal of pharmacy practice 2015;28:249–55. doi:10.1177/0897190013516368 |
| 19. | Hammond Aubrie, Porter Regan, Lynch Kevin E, et al. Impact of emergency medicine clinical pharmacist practitioner-driven sepsis antibiotic interventions. The American journal of emergency medicine 2024;76:24–8. doi:10.1016/j.ajem.2023.11.012 |
| 20. | Hashimoto Masayoshi, Asai Satomi, Umezawa Kazuo, et al. Impact of ward pharmacist-led antimicrobial stewardship in intensive care units. Journal of chemotherapy (Florence, Italy) 2023;35:188–97. doi:10.1080/1120009X.2022.2087652 |
| 21. | Hegland Andrea J, Bolduc Jennifer, Jones Lindsey, et al. Pharmacist-Driven Deprescribing of Inhaled Corticosteroids in Patients with Stable Chronic Obstructive Pulmonary Disease. Annals of the American Thoracic Society 2021;18:730–3. doi:10.1513/annalsats.202007-871rl |
| 22. | Hosmane Sharath, Tucker Johanna, Osman Dave, et al. Inpatient Oral Anticoagulation Management by Clinical Pharmacists: Safety and Cost effectiveness. Journal of clinical medicine research 2010;2:90–2. doi:10.4021/jocmr2010.03.283w |
| 23. | Huff JM, Falter RA, Scheinberg N. Retrospective Comparison of Appropriate Statin Use Between Patients With Diabetes in the Primary Care Setting Managed by Pharmacists or Internal Medicine Providers. Diabetes Spectr 2019;32:349–54. doi:10.2337/ds18-0067 |
| 24. | International Pharmaceutical Federation (FIP. Pharmacy-led common ailment schemes: a global intelligrnace report (executive summary). Published Online First: 2024.https://www.fip.org/file/5909 |
| 25. | Ip EJ; S Bijal M; Yu, Junhua; Chan, James; Nguyen, Lynda T; Bhatt, DeempalC. Enhancing diabetes care by adding a pharmacist to the primary care team. American journal of health-system pharmacy : AJHP : official journal of the American Society of Health-System Pharmacists 2013;70:877–86. doi:10.2146/ajhp120238 |
| 26. | Irons BKL Ranee J; Anderson, Stephanie L; Wharton, Benita L; Habeger, Butch; Anderson, H Glenn. A retrospective cohort analysis of the clinical effectiveness of a physician-pharmacist collaborative drug therapy management diabetes clinic. Pharmacotherapy 2002;22:1294–300. doi:10.1592/phco.22.15.1294.33476 |
| 27. | Irons BK, Meyerrose G, Laguardia S, et al. A collaborative cardiologist-pharmacist care model to improve hypertension management in patients with or at high risk for cardiovascular disease. Pharmacy Practice (Granada) 2012;10:25–32. |
| 28. | Kang Amy, Thompson Ashley, Rau Johnny, et al. Effects of clinical decision support and pharmacist prescribing authority on a therapeutic interchange program. American journal of health-system pharmacy : AJHP : official journal of the American Society of Health-System Pharmacists 2018;75:S77–81. doi:10.2146/ajhp170465 |

| Excluded on study design (n=56) | |
|---|---|
| 29. | Martin Christina Y, Hermann Staci A, Couldry Rick J. Impact of pharmacist-managed warfarin on patient outcomes in an academic medical center. American journal of health-system pharmacy: AJHP: official journal of the American Society of Health-System Pharmacists 2015;72:S31. doi:10.2146/sp150008 |
| 30. | Martinez Amanda S, Saef Jerold, Paszczuk Anna, et al. Implementation of a pharmacist-managed heart failure medication titration clinic. American journal of health-system pharmacy : AJHP : official journal of the American Society of Health-System Pharmacists 2013;70:1070–6. doi:10.2146/ajhp120267 |
| 31. | NHS Education for Scotland. Independent prescribing, consultation and clinical assessment skills training. 2024.https://www.nes.scot.nhs.uk/our-work/pharmacy/independent-prescribing-consultation-and-clinical-assessment-skills-training/ |
| 32. | O'Neil Meredith J, Garr BreAnn N, Faircloth Jenna M, et al. Utility of a pharmacist-managed Anticoagulation Program in patients with congenital heart disease. Cardiology in the young 2024;34:628–33. doi:10.1017/S1047951123003268 |
| 33. | Owen Kathryn, Winters Holli, Palettas Marilly, et al. Impact of a pharmacist-led tacrolimus management protocol in the outpatient setting. Journal of the American Pharmacists Association : JAPhA 2022;62:1912–8. doi:10.1016/j.japh.2022.06.007 |
| 34. | Padiyara RS; D Jennifer J; Rihani, Rami S. Clinical Pharmacist Intervention and the Proportion of Diabetes Patients Attaining Prevention Objectives in a Multispecialty Medical Group. Journal of managed care pharmacy : JMCP 2011;17:456–62. doi:10.18553/jmcp.2011.17.6.456 |
| 35. | Penson D F. Implementation of treatment guidelines to support judicious use of antibiotic therapy [see comment below]. Journal of Urology 2011;185:156–7. doi:10.1016/j.juro.2010.09.069 |
| 36. | Poon IO, Lal L, Brown EN, et al. The impact of pharmacist-managed oral anticoagulation therapy in older veterans. J Clin Pharm Ther 2007;32:21–9. doi:10.1111/j.1365-2710.2007.00792.x |
| 37. | Portman David B. Implementing a pharmacist-run Lyme disease postexposure prophylaxis clinic augmented by academic detailing within the Veterans Health Administration. Journal of the American Pharmacists Association : JAPhA 2020;60:e70–5. doi:10.1016/j.japh.2020.01.002 |
| 38. | Prudencio JC Timothy W; Roberts, Stephanie; Marin, Stephanie; Wilson, Machelle D. The Effect of Clinical Pharmacist-Led Comprehensive Medication Management on Chronic Disease State Goal Attainment in a Patient-Centered Medical Home. Journal of managed care & specialty pharmacy 2018;24:423–9. doi:10.18553/jmcp.2018.24.5.423 |
| 39. | Pugazhenthi Vidya, Dick Travis B, Call Matthew. Outcomes of a pharmacist-managed glucose collaborative practice agreement. American journal of health-system pharmacy : AJHP : official journal of the American Society of Health-System Pharmacists 2016;73:S148–54. doi:10.2146/ajhp150648 |
| 40. | Rodriguez Keren E, Chelewski Rachel J, Peter Megan E, et al. Integrating pharmacists into a kidney transplant clinic: Developing and implementing a collaborative pharmacy practice agreement. Journal of the American Pharmacists Association : JAPhA 2022;62:349–56. doi:10.1016/j.japh.2021.07.004 |

| Excluded on study design (n=56) | |
|---|---|
| 41. | Samuel E, Thomas S, Shah K, et al. Evaluation of a pharmacist-managed warfarin dosing service in a community hospital. Pharmacy & Pharmacology International Journal 2021;9:34–7. doi:10.15406/ppij.2021.09.00324 |
| 42. | Schwenka Natalie, Donovan Anthony, Franck Logan, et al. Patient-centered medical home pharmacists' impact on composite quality care measures for patients with uncontrolled type 2 diabetes. Journal of the American Pharmacists Association : JAPhA 2023;63:1545. doi:10.1016/j.japh.2023.06.004 |
| 43. | Siegel D. In uncontrolled hypertension, a pharmacist-prescribing intervention reduced blood pressure at 6 months. Annals of Internal Medicine 2015;163:JC7. doi:10.7326/ACPJC-2015-163-10-007 |
| 44. | Silvia Richard, Plum Michelle, Dufresne Robert. Efficiencies and outcomes of depression treatment by a psychiatric pharmacist in a primary care clinic compared with treatment within a behavioral health clinic. Journal of the American Pharmacists Association : JAPhA 2020;60:S98–106. doi:10.1016/j.japh.2020.05.015 |
| 45. | Stimmel Glen L, McGhan William F, Wincor Michael Z, et al. Comparison of pharmacist and physician prescribing for psychiatric inpatients. American journal of hospital pharmacy 1982;39:1483–6. doi:10.1093/ajhp/39.9.1483 |
| 46. | Stimmel GL, McGhan WF, Wincor MZ, et al. Comparison of pharmacist and physician prescribing for psychiatric inpatients. American Journal of Hospital Pharmacy 1982;39:1483–6. doi:10.1093/ajhp/39.9.1483 |
| 47. | Sykora Daniel, Olson Nicole, Churchill Robert, et al. Pharmacist Medication Titration Program for Patients With Cardiac Sarcoidosis and Systolic Heart Failure: A Retrospective Cohort Study. Journal of the American Heart Association 2024;13:e038965. doi:10.1161/JAHA.124.038965 |
| 48. | Till LT, Voris JC, Horst JB. Assessment of clinical pharmacist management of lipid-lowering therapy in a primary care setting. J Manag Care Pharm 2003;9:269–73. doi:10.18553/jmcp.2003.9.3.269 |
| 49. | To Linh L, Stoner Carol P, Stolley Stephen N, et al. Effectiveness of a pharmacist-implemented anemia management protocol in an outpatient hemodialysis unit. American journal of health-system pharmacy : AJHP : official journal of the American Society of Health-System Pharmacists 2001;58:2061–5. doi:10.1093/ajhp/58.21.2061 |
| 50. | Tsuyuki Ross T, Rosenthal Meagen, Pearson Glen J. IMPROVING DYSLIPIDEMIA MANAGEMENT IN THE COMMUNITY: A RANDOMIZED TRIAL OF PHARMACIST PRESCRIBING, THE RxACT STUDY. Canadian Journal of Cardiology 2014;30:S118–9. doi:10.1016/j.cjca.2014.07.151 |
| 51. | Witt Daniel M, Humphries Tammy L. A retrospective evaluation of the management of excessive anticoagulation in an established clinical pharmacy anticoagulation service compared to traditional care. Journal of thrombosis and thrombolysis 2003;15:113–8. doi:10.1023/b:thro.0000003325.62542.43 |
| 52. | Witt Daniel M, Sadler Melanie A, Shanahan Roberta L, et al. Effect of a centralized clinical pharmacy anticoagulation service on the outcomes of anticoagulation therapy. Chest 2005;127:1515–22. doi:10.1378/chest.127.5.1515 |

| Excluded on study design (n=56) | |
|---|---|
| 53. | Wright A, Vaillancourt R, Bussieres J F, et al. Best of both worlds: A comparison of canadian and international best practices for hospital pharmacy services. Canadian Journal of Hospital Pharmacy 2015;68:48–53. doi:10.4212/cjhp.v68i1.1424 |
| 54. | Young Stephanie, Bishop Lisa, Twells Laurie, et al. Comparison of pharmacist managed anticoagulation with usual medical care in a family medicine clinic. BMC family practice 2011;12:88. doi:10.1186/1471-2296-12-88 |
| 55. | About the Digital Prescribing and Dispensing Pathways programme. National Services Scotland. https://www.nss.nhs.scot/national-programmes/digital-prescribing-and-dispensing-pathways/about-the-digital-prescribing-and-dispensing-pathways-programme/ |
| 56. | Achieving excellence in pharmaceutical care: a strategy for Scotland. https://www.gov.scot/publications/achieving-excellence-pharmaceutical-care-strategy-scotland/ |

| Excluded on unavailable (n=1) | |
|---|---|
| 1. | Parekh R, Ghee C. Evaluation of a pharmacist controlled anticoagulation clinic. *Br J Pharm Prac* 2007;10:370–81. |

# Appendix J Cochrane Risk of Bias 2 tool (for parallel randomised controlled trials)

| Author (year) | Category | Outcome | Primary or secondary outcome | Intervention | Comparator | 1. Randomisation | 2. Deviations from intended interventions | 3. Missing data | 4. Measurement of outcome | 5. Selection of the reported result | Overall |
|---|---|---|---|---|---|---|---|---|---|---|---|
| Chenella et al. (1983) | Effectiveness | No. days to achieve therapeutic proconvertin and prothrombin | Not reported | Pharmacist prescribing | Physician prescribing | Some concerns | Low | High | Low | Some concerns | High |
| Chenella et al. (1983) | Effectiveness | Partial thromboplast in time (PTT) | Not reported | Pharmacist prescribing | Physician prescribing | Some concerns | Low | High | Low | Some concerns | High |
| Bruhn et al. (2013) | Effectiveness | Health-related quality of life | Primary | Pharmacist prescribing | 1. Medication review 2. Usual care | Low | Low | High | Low | Low | High |
| Bruhn et al. (2013) (intervention vs medication review) | Effectiveness | Quality of life (physical component score) | Primary | Pharmacist prescribing | Medication review | Low | Low | High | Low | Low | High |

| Author (year) | Category | Outcome | Primary or secondary outcome | Intervention | Comparator | 1. Randomisation | 2. Deviations from intended interventions | 3. Missing data | 4. Measurement of outcome | 5. Selection of the reported result | Overall |
|---|---|---|---|---|---|---|---|---|---|---|---|
| Bruhn et al. (2013) (intervention vs medication review) | Effectiveness | Quality of life (mental component score) | Primary | Pharmacist prescribing | Medication review | Low | Low | High | Low | Low | High |
| Bruhn et al. (2013) (intervention vs medication review) | Effectiveness | Chronic pain intensity | Secondary | Pharmacist prescribing | Medication review | Low | Low | High | Low | Low | High |
| Bruhn et al. (2013) (intervention vs medication review) | Effectiveness | Chronic pain disability | Secondary | Pharmacist prescribing | Medication review | Low | Low | High | Low | Low | High |
| Bruhn et al. (2013) (intervention vs usual care) | Effectiveness | Quality of life (physical component score) | Primary | Pharmacist prescribing | Usual care | Low | Low | High | Low | Low | High |

| Author (year) | Category | Outcome | Primary or secondary outcome | Intervention | Comparator | 1. Randomisation | 2. Deviations from intended interventions | 3. Missing data | 4. Measurement of outcome | 5. Selection of the reported result | Overall |
|---|---|---|---|---|---|---|---|---|---|---|---|
| Bruhn et al. (2013) (intervention vs usual care) | Effectiveness | Quality of life (mental component score) | Primary | Pharmacist prescribing | Usual care | Low | Low | High | Low | Low | High |
| Bruhn et al. (2013) (intervention vs usual care) | Effectiveness | Chronic pain intensity | Secondary | Pharmacist prescribing | Usual care | Low | Low | High | Low | Low | High |
| Bruhn et al. (2013) (intervention vs usual care) | Effectiveness | Chronic pain disability | Secondary | Pharmacist prescribing | Usual care | Low | Low | High | Low | Low | High |
| Bruhn et al. (2013) (intervention vs. medication review) | Effectiveness | Depression | Secondary | Pharmacist prescribing | Medication review | Low | Low | High | Low | Low | High |
| Bruhn et al. (2013) (intervention vs. medication review) | Effectiveness | Anxiety | Secondary | Pharmacist prescribing | Medication review | Low | Low | High | Low | Low | High |

| Author (year) | Category | Outcome | Primary or secondary outcome | Intervention | Comparator | 1. Randomisation | 2. Deviations from intended interventions | 3. Missing data | 4. Measurement of outcome | 5. Selection of the reported result | Overall |
|---|---|---|---|---|---|---|---|---|---|---|---|
| Bruhn et al. (2013) (intervention vs. usual care) | Effectiveness | Depression | Secondary | Pharmacist prescribing | Usual care | Low | Low | High | Low | Low | High |
| Bruhn et al. (2013) (intervention vs. usual care) | Effectiveness | Anxiety | Secondary | Pharmacist prescribing | Usual care | Low | Low | High | Low | Low | High |
| Jameson et al. (2010) | Effectiveness | Change in HbA1C | Primary | Pharmacist management of diabetes | Physician prescribing with nurse case management | Some concerns | Low | Low | Low | Some concerns | Some concerns |

| Author (year) | Category | Outcome | Primary or secondary outcome | Intervention | Comparator | 1. Randomisation | 2. Deviations from intended interventions | 3. Missing data | 4. Measurement of outcome | 5. Selection of the reported result | Overall |
|---|---|---|---|---|---|---|---|---|---|---|---|
| Jameson et al. (2010) | Effectiveness | Patients who achieved at least a 1.0% decrease in HbA1C | Secondary | Pharmacist management of diabetes | Physician prescribing with nurse case management | Some concerns | Low | Low | Low | Some concerns | Some concerns |
| Lau et al. (2022) | Effectiveness | Fall requiring medical attention | Secondary | Pharmacist-led case management | Enhanced usual care | Some concerns | Low | Some concerns | Low | Low | Some concerns |
| Lau et al. (2022) | Effectiveness | Health related quality of life | Secondary | Pharmacist-led case management | Enhanced usual care | Some concerns | Low | Some concerns | Low | Low | Some concerns |
| Lau et al. (2022) | Effectiveness | Hospitalisations | Secondary | Pharmacist-led case management | Enhanced usual care | Some concerns | Low | Some concerns | Low | Low | Some concerns |

| Author (year) | Category | Outcome | Primary or secondary outcome | Intervention | Comparator | 1. Randomisation | 2. Deviations from intended interventions | 3. Missing data | 4. Measurement of outcome | 5. Selection of the reported result | Overall |
|---|---|---|---|---|---|---|---|---|---|---|---|
| Lau et al. (2022) | Effectiveness | Proportion achieving systolic BP targets on 24-h ambulatory BP monitoring | Primary | Pharmacist-led case management | Enhanced usual care | Some concerns | Low | Some concerns | Low | Low | Some concerns |
| Lau et al. (2022) | Effectiveness | Depression | Secondary | Pharmacist-led case management | Enhanced usual care | Some concerns | Low | Some concerns | Low | Low | Some concerns |
| Lau et al. (2022) | Effectiveness | Anxiety | Secondary | Pharmacist-led case management | Enhanced usual care | Some concerns | Low | Some concerns | Low | Low | Some concerns |
| Lau et al. (2022) | Effectiveness | ED admissions | Secondary | Pharmacist-led case management | Enhanced usual care | Some concerns | Low | Some concerns | Low | Low | Some concerns |
| McAllister et al. (2014) | Effectiveness | Attained optimal systolic blood pressure and lipid level by 6 months | Secondary | Pharmacist-led case management | Nurse-led case management | Low | Low | High | Low | High | Some concerns |

| Author (year) | Category | Outcome | Primary or secondary outcome | Intervention | Comparator | 1. Randomisation | 2. Deviations from intended interventions | 3. Missing data | 4. Measurement of outcome | 5. Selection of the reported result | Overall |
|---|---|---|---|---|---|---|---|---|---|---|---|
| McAllister et al. (2014) | Effectiveness | Systolic BP | Secondary | Pharmacist-led case management | Nurse-led case management | Low | Low | Low | Low | High | Some concerns |
| McAllister et al. (2014) | Effectiveness | Mean LDL | Secondary | Pharmacist-led case management | Nurse-led case management | Low | Low | High | Low | High | Some concerns |
| McAllister et al. (2014) | Effectiveness | Change in HDL cholesterol | Secondary | Pharmacist-led case management | Nurse-led case management | Low | Low | High | Low | High | Some concerns |
| McAllister et al. (2014) | Effectiveness | Self-reported adherence of 75% or higher for blood pressure or lipid-lowering medications | Secondary | Pharmacist-led case management | Nurse-led case management | Low | Low | High | High | High | Some concerns |
| McAllister et al. (2014) | Effectiveness | Self-rated health | Secondary | Pharmacist-led case management | Nurse-led case management | Low | Low | High | High | High | Some concerns |
| McAllister et al. (2014) | Effectiveness | Quality of life | Secondary | Pharmacist-led case management | Nurse-led case management | Low | Low | High | High | High | Some concerns |

| Author (year) | Category | Outcome | Primary or secondary outcome | Intervention | Comparator | 1. Randomisation | 2. Deviations from intended interventions | 3. Missing data | 4. Measurement of outcome | 5. Selection of the reported result | Overall |
|---|---|---|---|---|---|---|---|---|---|---|---|
| Scullin et al. (2007) | Effectiveness | Length of hospital stay | Primary | Pharmacist prescribing (Integrated medicines management) | Usual care, physician prescribing | Low | High | Low | Low | Some concerns | High |
| Scullin et al. (2007) | Effectiveness | Hospital readmission | Secondary | Pharmacist prescribing (Integrated medicines management) | Usual care, physician prescribing | Low | High | Low | Low | Some concerns | High |
| Vivian (2002) | Effectiveness | Systolic blood pressure | Not reported | Pharmacist prescribing | Physician prescribing | High | Some concerns | Low | Low | Low | High |
| Vivian (2002) | Effectiveness | Diastolic blood pressure | Not reported | Pharmacist prescribing | Physician prescribing | High | Some concerns | Low | Low | Low | High |
| Vivian (2002) | Effectiveness | Blood pressure goal reached | Not reported | Pharmacist prescribing | Physician prescribing | High | Some concerns | Low | Low | Low | High |

| Author (year) | Category | Outcome | Primary or secondary outcome | Intervention | Comparator | 1. Randomisation | 2. Deviations from intended interventions | 3. Missing data | 4. Measurement of outcome | 5. Selection of the reported result | Overall |
|---|---|---|---|---|---|---|---|---|---|---|---|
| Vivian (2002) | Effectiveness | Adherence | Not reported | Pharmacist prescribing | Physician prescribing | High | Some concerns | Low | Some concerns | Low | High |
| Vivian (2002) | Effectiveness | Quality of life | Not reported | Pharmacist prescribing | Physician prescribing | High | Some concerns | Low | Low | Low | High |
| Xu et al. (2021) | Effectiveness | Change in HbA1C | Primary | Team-based pharmaceutical care | Usual care, physician prescribing | Low | Low | Some concerns | Low | High | High |
| Xu et al. (2021) | Effectiveness | Diabetes-specific quality of life | Secondary | Team-based pharmaceutical care | Usual care, physician prescribing | Low | Low | Some concerns | Some concerns | High | High |
| Lau et al. (2022) | Safety | Syncope | Secondary | Pharmacist-led case management | Enhanced usual care | Some concerns | Low | Some concerns | Low | Low | High |

| Author (year) | Category | Outcome | Primary or secondary outcome | Intervention | Comparator | 1. Randomisation | 2. Deviations from intended interventions | 3. Missing data | 4. Measurement of outcome | 5. Selection of the reported result | Overall |
|---|---|---|---|---|---|---|---|---|---|---|---|
| Lau et al. (2022) | Safety | Hypotension | Secondary | Pharmacist-led case management | Enhanced usual care | Some concerns | Low | Some concerns | Low | Low | High |
| Lau et al. (2022) | Safety | Hypokalemia | Secondary | Pharmacist-led case management | Enhanced usual care | Some concerns | Low | Some concerns | Low | Low | High |
| Lau et al. (2022) | Safety | Hyperkalemia | Secondary | Pharmacist-led case management | Enhanced usual care | Some concerns | Low | Some concerns | Low | Low | High |
| Lau et al. (2022) | Safety | Hyponatremia | Secondary | Pharmacist-led case management | Enhanced usual care | Some concerns | Low | Some concerns | Low | Low | High |
| Lau et al. (2022) | Safety | Orthostatic presyncope | Secondary | Pharmacist-led case management | Enhanced usual care | Some concerns | Low | Some concerns | Low | Low | High |
| Lau et al. (2022) | Safety | Change in eGFR | Secondary | Pharmacist-led case management | Enhanced usual care | Some concerns | Low | Some concerns | Low | Low | High |

| Author (year) | Category | Outcome | Primary or secondary outcome | Intervention | Comparator | 1. Randomisation | 2. Deviations from intended interventions | 3. Missing data | 4. Measurement of outcome | 5. Selection of the reported result | Overall |
|---|---|---|---|---|---|---|---|---|---|---|---|
| Marotti et al. (2011) (intervention vs usual care) | Safety | Doses missed during inpatient stay | Primary | Pharmacist medication review and prescribing | Usual care | Low | Low | Low | High | Some concerns | High |
| Marotti et al. (2011) (intervention vs usual care) | Safety | Medication charted at incorrect dose | Secondary | Pharmacist medication review and prescribing | Usual care | Low | Low | Low | High | Some concerns | High |
| Marotti et al. (2011) (intervention vs usual care) | Safety | Medications charted at incorrect frequency | Secondary | Pharmacist medication review and prescribing | Usual care | Low | Low | Low | High | Some concerns | High |
| Marotti et al. (2011) (intervention vs. medication review) | Safety | Doses missed during inpatient stay | Primary | Pharmacist medication review and prescribing | Medication review | Low | Low | Low | High | Some concerns | Hign |
| Marotti et al. (2011) (intervention vs. medication review) | Safety | Medication charted at incorrect dose | Secondary | Pharmacist medication review and prescribing | Medication review | Low | Low | Low | High | Some concerns | Hign |

| Author (year) | Category | Outcome | Primary or secondary outcome | Intervention | Comparator | 1. Randomisation | 2. Deviations from intended interventions | 3. Missing data | 4. Measurement of outcome | 5. Selection of the reported result | Overall |
|---|---|---|---|---|---|---|---|---|---|---|---|
| Marotti et al. (2011) (intervention vs. medication review) | Safety | Medications charted at incorrect frequency | Secondary | Pharmacist medication review and prescribing | Medication review | Low | Low | Low | High | Some concerns | Hign |
| McAlister et al. (2014) | Safety | Mortality | Secondary | Pharmacist-led case management | Nurse-led case management | Low | Low | High | Low | High | Hign |
| McAlister et al. (2014) | Safety | Vascular event | Secondary | Pharmacist-led case management | Nurse-led case management | Low | Low | High | Low | High | Hign |
| Ogilvie et al. (2022) | Safety | Prescribing errors | Not reported | Pharmacist prescribing with nurse case management | Medical practitioner prescribing | Low | Low | High | Low | Some concerns | Hign |
| Scullin et al. (2007) | Safety | 12-month mortality | Secondary | Pharmacist prescribing (Integrated medicines management) | Usual care, physician prescribing | Low | High | Low | Low | Some concerns | Hign |

# Appendix K Cochrane Risk of Bias 2 tool (for cluster randomised controlled trials)

| Author (year) | Category | Outcome | Primary or secondary outcome | Intervention | Comparator | 1a. Randomisation | 1b. Timing of identification or recruitment | 2. Deviations from intended interventions | 3. Missing data | 4. Measurement of outcome | 5. Selection of the reported result | Overall |
|---|---|---|---|---|---|---|---|---|---|---|---|---|
| Holland et al. (2023) | Effectiveness | Fall rate/person at six months | Primary | Pharmacist prescribing | Usual care, GP prescribing | Some concerns | Some concerns | High | High | Low | Low | High |
| Holland et al. (2023) | Effectiveness | Quality of life (EQ-5D by proxy) | Secondary | Pharmacist prescribing | Usual care, GP prescribing | Some concerns | Some concerns | High | High | High | Low | High |
| Holland et al. (2023) | Effectiveness | Drug Burden Index | Secondary | Pharmacist prescribing | Usual care, GP prescribing | Some concerns | Some concerns | High | High | Low | Low | High |
| Holland et al. (2023) | Effectiveness | Hospital admissions | Secondary | Pharmacist prescribing | Usual care, GP prescribing | Some concerns | Some concerns | High | High | Low | Low | High |
| Villeneuve et al. (2010) | Effectiveness | Mean LDL | Primary | Pharmacist prescribing | Usual care (physician prescribing) | Some concerns | Some concerns | Low | Low | Low | Low | Some concerns |
| Villeneuve et al. (2010) | Effectiveness | Proportion achieving target lipid levels | Secondary | Pharmacist prescribing | Usual care (physician prescribing) | Some concerns | Some concerns | Low | Low | Low | Low | Some concerns |
| Villeneuve et al. (2010) | Effectiveness | HDL cholesterol | Secondary | Pharmacist prescribing | Usual care (physician prescribing) | Some concerns | Some concerns | Low | Low | Low | Low | Some concerns |

| | | | | | | | | | | | | |
|---|---|---|---|---|---|---|---|---|---|---|---|---|
| Villeneueve et al. (2010) | Effectiveness | Triglycerides | Secondary | Pharmacist prescribing | Usual care (physician prescribing) | Some concerns | Some concerns | Low | Low | Low | Low | Some concerns |
| Villeneueve et al. (2010) | Effectiveness | Systolic blood pressure | Secondary | Pharmacist prescribing | Usual care (physician prescribing) | Some concerns | Some concerns | Low | Low | Low | Low | Some concerns |
| Villeneueve et al. (2010) | Effectiveness | Diastolic blood pressure | Secondary | Pharmacist prescribing | Usual care (physician prescribing) | Some concerns | Some concerns | Low | Low | Low | Low | Some concerns |
| Villeneueve et al. (2010) | Effectiveness | Fasting blood glucose | Secondary | Pharmacist prescribing | Usual care (physician prescribing) | Some concerns | Some concerns | Low | Low | Low | Low | Some concerns |
| Villeneueve et al. (2010) | Effectiveness | Healthcare utilisation (number of physician visits) | Secondary | Pharmacist prescribing | Usual care (physician prescribing) | Some concerns | Some concerns | Low | Low | Low | Some concerns | Some concerns |
| Villeneueve et al. (2010) | Effectiveness | Adherence | Secondary | Pharmacist prescribing | Usual care (physician prescribing) | Some concerns | Some concerns | Low | Low | Low | Low | Some concerns |
| Holland et al. (2023) | Safety | Mortality | Secondary | Pharmacist prescribing | Usual care, GP prescribing | Some concerns | Some concerns | High | High | Low | Low | High |
| Villeneueve et al. (2010) | Safety | Adverse events | Secondary | Pharmacist prescribing | Usual care (physician prescribing) | Some concerns | Some concerns | Low | Low | Low | Some concerns | Some concerns |

# Appendix L Risk Of Bias In Non-Randomised Studies – of Interventions assessment

| Author (year) | Category | Outcome | Primary or secondary outcome | B1 Did the authors make any attempt to control for confounding? | *B2 Is there sufficient potential for confounding that an unadjusted result should not be considered further?* | B3 Was the method of measuring the outcome inappropriate? | Proceed or appraised | 1. CONFOUNDING (variant A) | 2. CLASSIFICATION OF INTERVENTIONS | 3. SELECTION OF PARTICIPANTS | 4. DEVIATIONS FROM INTENDED INTERVENTIONS (variant A) | 5. MISSING DATA | 6. MEASUREMENT OF OUTCOME | 7. SELECTION OF THE REPORTED RESULT | Overall |
|---|---|---|---|---|---|---|---|---|---|---|---|---|---|---|---|
| Aspinall et al. (2012) (intervention vs. clinic physician) | Effectiveness | Proportion of haemoglobin values within the target range of 10-12 g/dl | Primary | Y | NA | N | Proceed | Serious | Serious | Serious | Serious | Serious | Serious | Serious | Critical |
| Aspinall et al. (2012) (intervention vs. usual care) | Effectiveness | Proportion of haemoglobin values within the target range of 10-12 g/dl | Primary | Y | NA | N | Proceed | Moderate | Serious | Serious | Moderate | Moderate | Serious | Serious | Critical |
| Beahm et al. (2018) | Effectiveness | Clinical cure | Primary | N | Y | PN | Critical | | | | | | | | Critical |

| Author (year) | Category | Outcome | Primary or secondary outcome | B1 Did the authors make any attempt to control for confounding? | *B2 Is there sufficient potential for confounding that an unadjusted result should not be considered further?* | B3 Was the method of measuring the outcome inappropriate? | Proceed or appraised | 1. CONFOUNDING (variant A) | 2. CLASSIFICATION OF INTERVENTIONS | 3. SELECTION OF PARTICIPANTS | 4. DEVIATIONS FROM INTENDED INTERVENTIONS (variant A) | 5. MISSING DATA | 6. MEASUREMENT OF OUTCOME | 7. SELECTION OF THE REPORTED RESULT | Overall |
|---|---|---|---|---|---|---|---|---|---|---|---|---|---|---|---|
| Beahm et al. (2018) | Effectiveness | Waiting time | Secondary | N | Y | PN | Critical | | | | | | | | Critical |
| Beahm et al. (2018) | Effectiveness | Adherence | Secondary | N | Y | PN | Critical | | | | | | | | Critical |
| Boddy (2001) | Effectiveness | INR goal achieved | Not reported | N | Y | N | Critical | | | | | | | | Critical |
| Boddy (2001) | Effectiveness | INR <2.0 | Not reported | N | Y | N | Critical | | | | | | | | Critical |

| **Author (year)** | **Category** | **Outcome** | **Primary or secondary outcome** | **B1 Did the authors make any attempt to control for confounding?** | ***B2 Is there sufficient potential for confounding that an unadjusted result should not be considered further?*** | **B3 Was the method of measuring the outcome inappropriate?** | **Proceed or appraised** | **1. CONFOUNDING (variant A)** | **2. CLASSIFICATION OF INTERVENTIONS** | **3. SELECTION OF PARTICIPANTS** | **4. DEVIATIONS FROM INTENDED INTERVENTIONS (variant A)** | **5. MISSING DATA** | **6. MEASUREMENT OF OUTCOME** | **7. SELECTION OF THE REPORTED RESULT** | **Overall** |
|---|---|---|---|---|---|---|---|---|---|---|---|---|---|---|---|
| Boddy (2001) | Effectiveness | INR >6.0 | Not reported | N | Y | N | Critical | | | | | | | | Critical |
| Cohen et al. (1985) | Effectiveness | Prothrombin time ratio | Not reported | N | Y | N | Critical | | | | | | | | Critical |
| Cowart et al. (2020) | Effectiveness | HbA1C | Not reported | N | Y | N | Critical | | | | | | | | Critical |
| Cowart et al. (2020) | Effectiveness | HbA1C | Not reported | N | Y | N | Critical | | | | | | | | Critical |
| Cowart et al. (2022) | Effectiveness | Time to treatment intensification | Not reported | Y | NA | PN | Proceed | Serious | Critical | Critical | Serious | Moderate | Serious | Serious | Critical |

| Author (year) | Category | Outcome | Primary or secondary outcome | B1 Did the authors make any attempt to control for confounding? | *B2 Is there sufficient potential for confounding that an unadjusted result should not be considered further?* | B3 Was the method of measuring the outcome inappropriate? | Proceed or appraised | 1. CONFOUNDING (variant A) | 2. CLASSIFICATION OF INTERVENTIONS | 3. SELECTION OF PARTICIPANTS | 4. DEVIATIONS FROM INTENDED INTERVENTIONS (variant A) | 5. MISSING DATA | 6. MEASUREMENT OF OUTCOME | 7. SELECTION OF THE REPORTED RESULT | Overall |
|---|---|---|---|---|---|---|---|---|---|---|---|---|---|---|---|
| Cowart et al. (2022) | Effectiveness | HbA1C | Not reported | Y | NA | N | Proceed | Serious | Critical | Critical | Serious | Moderate | Serious | Serious | Critical |
| Damaske et al. (2005) | Effectiveness | Average time to therapeutic INR | Not reported | N | Y | Y | Critical | | | | | | | | Critical |
| Hahn et al. (2019) (intervention vs. medication therapy management with no CPA) | Effectiveness | Hospitalisations (all-cause) | Primary | N | Y | N | Critical | | | | | | | | Critical |
| Hahn et al. (2019) (intervention vs. medication therapy management with no CPA) | Effectiveness | Hospitalisations (all-cause) | Primary | N | Y | N | Critical | | | | | | | | Critical |

| Author (year) | Category | Outcome | Primary or secondary outcome | B1 Did the authors make any attempt to control for confounding? | *B2 Is there sufficient potential for confounding that an unadjusted result should not be considered further?* | B3 Was the method of measuring the outcome inappropriate? | Proceed or appraised | 1. CONFOUNDING (variant A) | 2. CLASSIFICATION OF INTERVENTIONS | 3. SELECTION OF PARTICIPANTS | 4. DEVIATIONS FROM INTENDED INTERVENTIONS (variant A) | 5. MISSING DATA | 6. MEASUREMENT OF OUTCOME | 7. SELECTION OF THE REPORTED RESULT | Overall |
|---|---|---|---|---|---|---|---|---|---|---|---|---|---|---|---|
| Hahn et al. (2019) (intervention vs. medication therapy management with no CPA) | Effectiveness | Emergency department visits | Secondary | N | Y | N | Critical | | | | | | | | Critical |
| Hahn et al. (2019) (intervention vs. usual care) | Effectiveness | Hospitalisations (heart failure cause) | Secondary | N | Y | N | Critical | | | | | | | | Critical |
| Hahn et al. (2019) (intervention vs. usual care) | Effectiveness | Hospitalisations (heart failure cauyse) | Secondary | N | Y | N | Critical | | | | | | | | Critical |
| Hahn et al. (2019) (intervention vs. usual care) | Effectiveness | Emergency department visits | Secondary | N | Y | N | Critical | | | | | | | | Critical |

| Author (year) | Category | Outcome | Primary or secondary outcome | B1 Did the authors make any attempt to control for confounding? | *B2 Is there sufficient potential for confounding that an unadjusted result should not be considered further?* | B3 Was the method of measuring the outcome inappropriate? | Proceed or appraised | 1. CONFOUNDING (variant A) | 2. CLASSIFICATION OF INTERVENTIONS | 3. SELECTION OF PARTICIPANTS | 4. DEVIATIONS FROM INTENDED INTERVENTIONS (variant A) | 5. MISSING DATA | 6. MEASUREMENT OF OUTCOME | 7. SELECTION OF THE REPORTED RESULT | Overall |
|---|---|---|---|---|---|---|---|---|---|---|---|---|---|---|---|
| Hall et al. (2011) | Effectiveness | INR goal achieved | Not reported | Y | NA | N | Proceed | Serious | Low | Critical | Serious | Serious | Serious | Serious | Critical |
| Hall et al. (2011) | Effectiveness | Time therapeutic goal maintained | Not reported | Y | NA | N | Proceed | Serious | Low | Critical | Serious | Serious | Serious | Serious | Critical |
| Hernández-Muñoz et al. (2021) | Effectiveness | HbA1C | Primary | Y | NA | N | Proceed | Serious | Low | Moderate | Serious | Critical | Low | Serious | Critical |
| Lum et al. (2023) | Effectiveness | HbA1C | Primary | Y | NA | N | Proceed | Serious | Serious | Serious | Low | Low | Low | Serious | Critical |

| Author (year) | Category | Outcome | Primary or secondary outcome | B1 Did the authors make any attempt to control for confounding? | *B2 Is there sufficient potential for confounding that an unadjusted result should not be considered further?* | B3 Was the method of measuring the outcome inappropriate? | Proceed or appraised | 1. CONFOUNDING (variant A) | 2. CLASSIFICATION OF INTERVENTIONS | 3. SELECTION OF PARTICIPANTS | 4. DEVIATIONS FROM INTENDED INTERVENTIONS (variant A) | 5. MISSING DATA | 6. MEASUREMENT OF OUTCOME | 7. SELECTION OF THE REPORTED RESULT | Overall |
|---|---|---|---|---|---|---|---|---|---|---|---|---|---|---|---|
| Maeng et al. (2018) | Effectiveness | HbA1C | Not reported | Y | NA | N | Proceed | Serious | Serious | Serious | Low | Low | Low | Serious | Critical |
| Maeng et al. (2018) | Effectiveness | HbA1C | Not reported | Y | NA | N | Proceed | Serious | Serious | Serious | Low | Low | Low | Serious | Critical |
| Maeng et al. (2018) | Effectiveness | LDL cholesterol | Not reported | Y | NA | N | Proceed | Serious | Serious | Serious | Low | Low | Low | Serious | Critical |
| Maeng et al. (2018) | Effectiveness | LDL cholesterol | Not reported | Y | NA | N | Proceed | Serious | Serious | Serious | Low | Low | Low | Serious | Critical |
| Maeng et al. (2018) | Effectiveness | Systolic blood pressure | Not reported | Y | NA | N | Proceed | Serious | Serious | Serious | Low | Low | Low | Serious | Critical |
| Maeng et al. (2018) | Effectiveness | Diastolic blood pressure | Not reported | Y | NA | N | Proceed | Serious | Serious | Serious | Low | Low | Low | Serious | Critical |
| Maeng et al. (2018) | Effectiveness | Blood pressure goal reached | Not reported | Y | NA | N | Proceed | Serious | Serious | Serious | Low | Low | Low | Serious | Critical |
| Manzoor et al. (2018) | Effectiveness | INR | Primary | Y | NA | N | Proceed | Serious | Serious | Moderate | Low | Low | Low | Serious | Serious |
| Morello et al. (2016) | Effectiveness | HbA1C | Primary | Y | NA | N | Proceed | Serious | Serious | Critical | Low | Low | Serious | Serious | Critical |

| Author (year) | Category | Outcome | Primary or secondary outcome | B1 Did the authors make any attempt to control for confounding? | *B2 Is there sufficient potential for confounding that an unadjusted result should not be considered further?* | B3 Was the method of measuring the outcome inappropriate? | Proceed or appraised | 1. CONFOUNDING (variant A) | 2. CLASSIFICATION OF INTERVENTIONS | 3. SELECTION OF PARTICIPANTS | 4. DEVIATIONS FROM INTENDED INTERVENTIONS (variant A) | 5. MISSING DATA | 6. MEASUREMENT OF OUTCOME | 7. SELECTION OF THE REPORTED RESULT | Overall |
|---|---|---|---|---|---|---|---|---|---|---|---|---|---|---|---|
| Morello et al. (2016) | Effectiveness | Fasting blood glucose | Secondary | Y | NA | N | Proceed | Serious | Serious | Critical | Low | Low | Serious | Serious | Critical |
| Morello et al. (2016) | Effectiveness | LDL cholesterol | Secondary | Y | NA | N | Proceed | Serious | Serious | Critical | Low | Serious | Serious | Serious | Critical |
| Morello et al. (2016) | Effectiveness | HDL cholesterol | Secondary | Y | NA | N | Proceed | Serious | Serious | Critical | Low | Serious | Serious | Serious | Critical |
| Morello et al. (2016) | Effectiveness | Triglycerides | Secondary | Y | NA | N | Proceed | Serious | Serious | Critical | Low | Serious | Serious | Serious | Critical |
| Morello et al. (2016) | Effectiveness | Systolic blood pressure | Secondary | Y | NA | N | Proceed | Serious | Serious | Critical | Low | Low | Serious | Serious | Critical |
| Morello et al. (2016) | Effectiveness | Diastolic blood pressure | Secondary | Y | NA | N | Proceed | Serious | Serious | Critical | Low | Low | Serious | Serious | Critical |

| Author (year) | Category | Outcome | Primary or secondary outcome | B1 Did the authors make any attempt to control for confounding? | *B2 Is there sufficient potential for confounding that an unadjusted result should not be considered further?* | B3 Was the method of measuring the outcome inappropriate? | Proceed or appraised | 1. CONFOUNDING (variant A) | 2. CLASSIFICATION OF INTERVENTIONS | 3. SELECTION OF PARTICIPANTS | 4. DEVIATIONS FROM INTENDED INTERVENTIONS (variant A) | 5. MISSING DATA | 6. MEASUREMENT OF OUTCOME | 7. SELECTION OF THE REPORTED RESULT | Overall |
|---|---|---|---|---|---|---|---|---|---|---|---|---|---|---|---|
| O'Neill et al. (2014) | Effectiveness | Blood pressure goal achieved | Primary | Y | NA | N | Proceed | Serious | Low | Critical | Low | Serious | Serious | Serious | Critical |
| O'Neill et al. (2014) | Effectiveness | Systolic blood pressure | Primary | Y | NA | N | Proceed | Serious | Low | Critical | Low | Serious | Serious | Serious | Critical |
| O'Neill et al. (2014) | Effectiveness | Diastolic blood pressure | Primary | Y | NA | N | Proceed | Serious | Low | Critical | Low | Serious | Serious | Serious | Critical |
| Rana et al. (2023) | Effectiveness | Target dose achieved (proportion) | Primary | N | Y | N | Critical | | | | | | | | Critical |
| Rana et al. (2023) | Effectiveness | Target dose achieved | Secondary | N | Y | N | Critical | | | | | | | | Critical |

| Author (year) | Category | Outcome | Primary or secondary outcome | B1 Did the authors make any attempt to control for confounding? | *B2 Is there sufficient potential for confounding that an unadjusted result should not be considered further?* | B3 Was the method of measuring the outcome inappropriate? | Proceed or appraised | 1. CONFOUNDING (variant A) | 2. CLASSIFICATION OF INTERVENTIONS | 3. SELECTION OF PARTICIPANTS | 4. DEVIATIONS FROM INTENDED INTERVENTIONS (variant A) | 5. MISSING DATA | 6. MEASUREMENT OF OUTCOME | 7. SELECTION OF THE REPORTED RESULT | Overall |
|---|---|---|---|---|---|---|---|---|---|---|---|---|---|---|---|
| | | (median) | | | | | | | | | | | | | |
| Rana et al. (2023) | Effectiveness | Target dose achieved (number of days) | Secondary | N | Y | N | Critical | | | | | | | | Critical |
| Rodriguez et al. (2021a) | Effectiveness | Continuation | Primary | Y | NA | N | Proceed | Moderate | Low | Low | Low | Low | Low | Serious | Serious |
| Rodriguez et al. (2021a) | Effectiveness | Adherence | Secondary | Y | NA | N | Proceed | Moderate | Low | Low | Low | Low | Low | Serious | Serious |
| Rodriguez et al. (2022) | Effectiveness | Continuation | Primary | Y | NA | N | Proceed | Moderate | Low | Low | Low | Low | Low | Serious | Serious |
| Rodriguez et al. (2022) | Effectiveness | Adherence | Secondary | Y | NA | N | Proceed | Moderate | Low | Low | Low | Low | Low | Serious | Serious |
| Romanelli et al. (2015) (intervention medication management | Effectiveness | Clinic visits | Primary | Y | NA | N | Proceed | Moderate | Serious | Serious | Low | Low | Serious | Serious | Critical |

| Author (year) | Category | Outcome | Primary or secondary outcome | B1 Did the authors make any attempt to control for confounding? | *B2 Is there sufficient potential for confounding that an unadjusted result should not be considered further?* | B3 Was the method of measuring the outcome inappropriate? | Proceed or appraised | 1. CONFOUNDING (variant A) | 2. CLASSIFICATION OF INTERVENTIONS | 3. SELECTION OF PARTICIPANTS | 4. DEVIATIONS FROM INTENDED INTERVENTIONS (variant A) | 5. MISSING DATA | 6. MEASUREMENT OF OUTCOME | 7. SELECTION OF THE REPORTED RESULT | Overall |
|---|---|---|---|---|---|---|---|---|---|---|---|---|---|---|---|
| programme vs. patient centred medical home) | | | | | | | | | | | | | | | |
| Romanelli et al. (2015) (intervention medication management programme vs. patient centred medical home) | Effectiveness | Emergency department visits | Primary | Y | NA | N | Proceed | Moderate | Serious | Serious | Low | Low | Serious | Serious | Critical |
| Romanelli et al. (2015) (intervention medication management program | Effectiveness | Hospitalisations | Primary | Y | NA | N | Proceed | Moderate | Serious | Serious | Low | Low | Serious | Serious | Critical |

| Author (year) | Category | Outcome | Primary or secondary outcome | B1 Did the authors make any attempt to control for confounding? | *B2 Is there sufficient potential for confounding that an unadjusted result should not be considered further?* | B3 Was the method of measuring the outcome inappropriate? | Proceed or appraised | 1. CONFOUNDING (variant A) | 2. CLASSIFICATION OF INTERVENTIONS | 3. SELECTION OF PARTICIPANTS | 4. DEVIATIONS FROM INTENDED INTERVENTIONS (variant A) | 5. MISSING DATA | 6. MEASUREMENT OF OUTCOME | 7. SELECTION OF THE REPORTED RESULT | Overall |
|---|---|---|---|---|---|---|---|---|---|---|---|---|---|---|---|
| me vs. patient centred medical home) | | | | | | | | | | | | | | | |
| Romanelli et al. (2015) (intervention medication management programme vs. patient centred medical home) | Effectiveness | Systolic/ diastolic blood pressure (% at goal) | Secondary | Y | NA | N | Proceed | Moderate | Serious | Serious | Low | Serious | Serious | Serious | Critical |
| Romanelli et al. (2015) (intervention medication management programme vs. | Effectiveness | LDL cholesterol | Secondary | Y | NA | N | Proceed | Moderate | Serious | Serious | Low | Serious | Serious | Serious | Critical |

| Author (year) | Category | Outcome | Primary or secondary outcome | B1 Did the authors make any attempt to control for confounding? | *B2 Is there sufficient potential for confounding that an unadjusted result should not be considered further?* | B3 Was the method of measuring the outcome inappropriate? | Proceed or appraised | 1. CONFOUNDING (variant A) | 2. CLASSIFICATION OF INTERVENTIONS | 3. SELECTION OF PARTICIPANTS | 4. DEVIATIONS FROM INTENDED INTERVENTIONS (variant A) | 5. MISSING DATA | 6. MEASUREMENT OF OUTCOME | 7. SELECTION OF THE REPORTED RESULT | Overall |
|---|---|---|---|---|---|---|---|---|---|---|---|---|---|---|---|
| patient centred medical home) | | | | | | | | | | | | | | | |
| Romanelli et al. (2015) (intervention medication management programme vs. patient centred medical home) | Effectiveness | HbA1C | Secondary | Y | NA | N | Proceed | Moderate | Serious | Serious | Low | Serious | Serious | Serious | Critical |
| Romanelli et al. (2015) (intervention vs. usual care) | Effectiveness | Clinic visits | Primary | Y | NA | N | Proceed | Moderate | Serious | Serious | Low | Low | Serious | Serious | Critical |
| Romanelli et al. (2015) (intervention vs. | Effectiveness | Emergency department visits | Primary | Y | NA | N | Proceed | Moderate | Serious | Serious | Low | Low | Serious | Serious | Critical |

| Author (year) | Category | Outcome | Primary or secondary outcome | B1 Did the authors make any attempt to control for confounding? | *B2 Is there sufficient potential for confounding that an unadjusted result should not be considered further?* | B3 Was the method of measuring the outcome inappropriate? | Proceed or appraised | 1. CONFOUNDING (variant A) | 2. CLASSIFICATION OF INTERVENTIONS | 3. SELECTION OF PARTICIPANTS | 4. DEVIATIONS FROM INTENDED INTERVENTIONS (variant A) | 5. MISSING DATA | 6. MEASUREMENT OF OUTCOME | 7. SELECTION OF THE REPORTED RESULT | Overall |
|---|---|---|---|---|---|---|---|---|---|---|---|---|---|---|---|
| usual care) | | | | | | | | | | | | | | | |
| Romanelli et al. (2015) (intervention vs. usual care) | Effectiveness | Hospitalisations | Primary | Y | NA | N | Proceed | Moderate | Serious | Serious | Low | Low | Serious | Serious | Critical |
| Romanelli et al. (2015) (intervention vs. usual care) | Effectiveness | Systolic/diastolic blood pressure (% at goal) | Secondary | Y | NA | N | Proceed | Moderate | Serious | Serious | Low | Serious | Serious | Serious | Critical |
| Romanelli et al. (2015) (intervention vs. usual care) | Effectiveness | LDL cholesterol | Secondary | Y | NA | N | Proceed | Moderate | Serious | Serious | Low | Serious | Serious | Serious | Critical |
| Romanelli et al. (2015) (intervention vs. usual care) | Effectiveness | HbA1C | Secondary | Y | NA | N | Proceed | Moderate | Serious | Serious | Low | Serious | Serious | Serious | Critical |

| Author (year) | Category | Outcome | Primary or secondary outcome | B1 Did the authors make any attempt to control for confounding? | *B2 Is there sufficient potential for confounding that an unadjusted result should not be considered further?* | B3 Was the method of measuring the outcome inappropriate? | Proceed or appraised | 1. CONFOUNDING (variant A) | 2. CLASSIFICATION OF INTERVENTIONS | 3. SELECTION OF PARTICIPANTS | 4. DEVIATIONS FROM INTENDED INTERVENTIONS (variant A) | 5. MISSING DATA | 6. MEASUREMENT OF OUTCOME | 7. SELECTION OF THE REPORTED RESULT | Overall |
|---|---|---|---|---|---|---|---|---|---|---|---|---|---|---|---|
| Thompson et al. (1984) | Effectiveness | Average number of drugs per patient | | N | Y | N | Critical | | | | | | | | Critical |
| Thompson et al. (1984) | Effectiveness | Hospitalisations | Not reported | N | Y | N | | | | | | | | | Critical |
| Varghese et al. (2024) | Effectiveness | Deprescribing | Primary | N | Y | N | | | | | | | | | Critical |
| Weaver et al. 2023 | Effectiveness | Haemoglobin (proportion) | Primary | Y | N | N | Proceed | Serious | Low | Critical | Serious | Critical | Serious | Serious | Critical |

| Author (year) | Category | Outcome | Primary or secondary outcome | B1 Did the authors make any attempt to control for confounding? | *B2 Is there sufficient potential for confounding that an unadjusted result should not be considered further?* | B3 Was the method of measuring the outcome inappropriate? | Proceed or appraised | 1. CONFOUNDING (variant A) | 2. CLASSIFICATION OF INTERVENTIONS | 3. SELECTION OF PARTICIPANTS | 4. DEVIATIONS FROM INTENDED INTERVENTIONS (variant A) | 5. MISSING DATA | 6. MEASUREMENT OF OUTCOME | 7. SELECTION OF THE REPORTED RESULT | Overall |
|---|---|---|---|---|---|---|---|---|---|---|---|---|---|---|---|
| Weaver et al. 2023 | Effectiveness | Haemoglobin (mean levels) | Secondary | Y | N | N | Proceed | Serious | Low | Critical | Serious | Critical | Serious | Serious | Critical |
| Aspinall et al. (2012) (intervention vs. clinic physician) | Safety | Thromboembolica adverse event | Secondary | Y | NA | N | Proceed | Moderate | Serious | Serious | Moderate | Moderate | Serious | Serious | Critical |
| Aspinall et al. (2012) (intervention vs. clinic physician) | Safety | Heart failure adverse event | Secondary | Y | NA | N | Proceed | Moderate | Serious | Serious | Moderate | Moderate | Serious | Serious | Critical |
| Aspinall et al. (2012) (intervention vs. clinic physician) | Safety | Uncontrolled hypertension adverse event | Secondary | Y | NA | N | Proceed | Moderate | Serious | Serious | Moderate | Moderate | Serious | Serious | Critical |

| Author (year) | Category | Outcome | Primary or secondary outcome | B1 Did the authors make any attempt to control for confounding? | *B2 Is there sufficient potential for confounding that an unadjusted result should not be considered further?* | B3 Was the method of measuring the outcome inappropriate? | Proceed or appraised | 1. CONFOUNDING (variant A) | 2. CLASSIFICATION OF INTERVENTIONS | 3. SELECTION OF PARTICIPANTS | 4. DEVIATIONS FROM INTENDED INTERVENTIONS (variant A) | 5. MISSING DATA | 6. MEASUREMENT OF OUTCOME | 7. SELECTION OF THE REPORTED RESULT | Overall |
|---|---|---|---|---|---|---|---|---|---|---|---|---|---|---|---|
| Aspinall et al. (2012) (intervention vs. usual care) | Safety | Thromboembolica adverse event | Secondary | Y | NA | N | Proceed | Moderate | Serious | Serious | Moderate | Moderate | Serious | Serious | Critical |
| Aspinall et al. (2012) (intervention vs. usual care) | Safety | Heart failure adverse event | Secondary | Y | NA | N | Proceed | Moderate | Serious | Serious | Moderate | Moderate | Serious | Serious | Critical |
| Aspinall et al. (2012) (intervention vs. usual care) | Safety | Uncontrolled hypertension adverse event | Secondary | Y | NA | N | Proceed | Moderate | Serious | Serious | Moderate | Moderate | Serious | Serious | Critical |
| Beahm et al. (2018) | Safety | All adverse events | Secondary | N | Y | PN | Critical | | | | | | | | Critical |
| Beahm et al. (2018) | Safety | Gastrointestinal adverse events | Secondary | N | Y | PN | Critical | | | | | | | | Critical |

| Author (year) | Category | Outcome | Primary or secondary outcome | B1 Did the authors make any attempt to control for confounding? | *B2 Is there sufficient potential for confounding that an unadjusted result should not be considered further?* | B3 Was the method of measuring the outcome inappropriate? | Proceed or appraised | 1. CONFOUNDING (variant A) | 2. CLASSIFICATION OF INTERVENTIONS | 3. SELECTION OF PARTICIPANTS | 4. DEVIATIONS FROM INTENDED INTERVENTIONS (variant A) | 5. MISSING DATA | 6. MEASUREMENT OF OUTCOME | 7. SELECTION OF THE REPORTED RESULT | Overall |
|---|---|---|---|---|---|---|---|---|---|---|---|---|---|---|---|
| Beahm et al. (2018) | Safety | Vaginal candidiasis adverse events | Secondary | N | Y | PN | Critical | | | | | | | | Critical |
| Beahm et al. (2018) | Safety | Headache adverse events | Secondary | N | Y | PN | Critical | | | | | | | | Critical |
| Beahm et al. (2018) | Safety | Other adverse events | Secondary | N | Y | PN | Critical | | | | | | | | Critical |
| Beahm et al. (2018) | Safety | Physician or emergency department events | Secondary | N | Y | PN | Critical | | | | | | | | Critical |
| Beahm et al. (2021) | Safety | Antibacterial therapy guideline concordance | Primary | N | Y | N | Critical | | | | | | | | Critical |

| Author (year) | Category | Outcome | Primary or secondary outcome | B1 Did the authors make any attempt to control for confounding? | *B2 Is there sufficient potential for confounding that an unadjusted result should not be considered further?* | B3 Was the method of measuring the outcome inappropriate? | Proceed or appraised | 1. CONFOUNDING (variant A) | 2. CLASSIFICATION OF INTERVENTIONS | 3. SELECTION OF PARTICIPANTS | 4. DEVIATIONS FROM INTENDED INTERVENTIONS (variant A) | 5. MISSING DATA | 6. MEASUREMENT OF OUTCOME | 7. SELECTION OF THE REPORTED RESULT | Overall |
|---|---|---|---|---|---|---|---|---|---|---|---|---|---|---|---|
| Cohen et al. (1985) | Safety | Warfarin-related complications (bleeding) | Not reported | N | Y | N | Critical | | | | | | | | Critical |
| Cohen et al. (1985) | Safety | Warfarin-related complications (thromboembolic events) | Not reported | N | Y | N | Critical | | | | | | | | Critical |
| Damaske et al. (2005) | Safety | Bleeds/adverse drug events | Not reported | N | Y | Y | Critical | | | | | | | | Critical |
| Hall et al. (2011) | Safety | Anticoagulation-related adverse events | Not reported | Y | NA | N | Proceed | Serious | Low | Critical | Serious | Serious | Serious | Serious | Critical |

| Author (year) | Category | Outcome | Primary or secondary outcome | B1 Did the authors make any attempt to control for confounding? | *B2 Is there sufficient potential for confounding that an unadjusted result should not be considered further?* | B3 Was the method of measuring the outcome inappropriate? | Proceed or appraised | 1. CONFOUNDING (variant A) | 2. CLASSIFICATION OF INTERVENTIONS | 3. SELECTION OF PARTICIPANTS | 4. DEVIATIONS FROM INTENDED INTERVENTIONS (variant A) | 5. MISSING DATA | 6. MEASUREMENT OF OUTCOME | 7. SELECTION OF THE REPORTED RESULT | Overall |
|---|---|---|---|---|---|---|---|---|---|---|---|---|---|---|---|
| Hall et al. (2011) | Safety | Anticoagulation-related emergency department visits | Not reported | Y | NA | N | Proceed | Serious | Low | Critical | Serious | Serious | Serious | Serious | Critical |
| Hall et al. (2011) | Safety | Anticoagulation-related hospital admissions | Not reported | Y | NA | N | Proceed | Serious | Low | Critical | Serious | Serious | Serious | Serious | Critical |
| Manzoor et al. (2018) | Safety | Warfarin related hospitalisations/ ED visits | Secondary | Y | NA | N | Proceed | Serious | Serious | Moderate | Low | Low | Low | Serious | Serious |
| McFarland et al. (2009) | Safety | Rates of inappropriate | Secondary | N | Y | N | Critical | | | | | | | | Serious |

| **Author (year)** | **Category** | **Outcome** | **Primary or secondary outcome** | **B1 Did the authors make any attempt to control for confounding?** | ***B2 Is there sufficient potential for confounding that an unadjusted result should not be considered further?*** | **B3 Was the method of measuring the outcome inappropriate?** | **Proceed or appraised** | **1. CONFOUNDING (variant A)** | **2. CLASSIFICATION OF INTERVENTIONS** | **3. SELECTION OF PARTICIPANTS** | **4. DEVIATIONS FROM INTENDED INTERVENTIONS (variant A)** | **5. MISSING DATA** | **6. MEASUREMENT OF OUTCOME** | **7. SELECTION OF THE REPORTED RESULT** | **Overall** |
|---|---|---|---|---|---|---|---|---|---|---|---|---|---|---|---|
| | | initial dosing | | | | | | | | | | | | | |
| Rana et al. (2023) | Safety | HF hospitalizations | Secondary | N | Y | N | Critical | | | | | | | | Critical |
| Rana et al. (2023) | Safety | All-cause death | Secondary | N | Y | N | Critical | | | | | | | | Critical |
| Rashid et al. (2020) | Safety | Gastrointestinal (GI) bleeding events (hospitalisations and ER visits) | Not reported | Y | NA | N | Proceed | Serious | Serious | Low | Low | Critical | Low | Serious | Critical |
| Rashid et al. (2020) | Safety | Acute kidney injury (AKI) events (hospitalisations and ER visits) | Not reported | Y | NA | N | Proceed | Serious | Serious | Low | Low | Critical | Low | Serious | Critical |
| Rashid et al. (2020) | Safety | Pain (hospitalisations and ER visits) | Not reported | Y | NA | N | Proceed | Serious | Serious | Low | Low | Critical | Low | Serious | Critical |

| Author (year) | Category | Outcome | Primary or secondary outcome | B1 Did the authors make any attempt to control for confounding? | *B2 Is there sufficient potential for confounding that an unadjusted result should not be considered further?* | B3 Was the method of measuring the outcome inappropriate? | Proceed or appraised | 1. CONFOUNDING (variant A) | 2. CLASSIFICATION OF INTERVENTIONS | 3. SELECTION OF PARTICIPANTS | 4. DEVIATIONS FROM INTENDED INTERVENTIONS (variant A) | 5. MISSING DATA | 6. MEASUREMENT OF OUTCOME | 7. SELECTION OF THE REPORTED RESULT | Overall |
|---|---|---|---|---|---|---|---|---|---|---|---|---|---|---|---|
| Rodriguez et al. (2020) | Safety | Medical contraindications | Secondary | Y | NA | N | Proceed | Moderate | Low | Low | Low | Low | Low | Serious | Serious |
| Rodriguez et al. (2021b) | Safety | Medical contraindications | Primary | N | Y | N | Critical | | | | | | | | Critical |
| Thompson et al. (1984) | Safety | Death | Not reported | N | Y | N | Critical | | | | | | | | Critical |

# Appendix M Grading of Recommendations Assessment, Development, and Evaluation

Diabetes effectiveness outcomes

| Participants (studies) | Risk of bias | Inconsistency | Indirectness | Imprecision | Publication bias | Overall certainty of evidence |
|---|---|---|---|---|---|---|
| HbA1c goal achieved (assessed with: Yes/No) | | | | | | |
| 5813 (3 non-randomised studies) | very serious[a] | serious[b,c] | not serious | not serious[d,e] | none | ⨁◯◯◯ Very low[a,b,c,d,e] |
| HbA1c levels (assessed with: Mean) | | | | | | |
| 1400 (2 non-randomised studies) | very serious[a] | serious[b,c] | not serious | not serious | none | ⨁◯◯◯ Very low[a,b,c] |
| Change in HbA1c levels (assessed with: Mean change) | | | | | | |
| 541 (2 non-randomised studies) | very serious[a] | very serious[b,c] | not serious | serious[b] | none | ⨁◯◯◯ Very low[a,b,c] |
| Change in HbA1c levels (assessed with: Mean change) | | | | | | |
| 351 (2 RCTs) | serious[f] | very serious[b,c] | not serious | not serious[e] | none | ⨁◯◯◯ Very low[b,c,e,f] |
| Time to achieve HbA1c goal (assessed with: Mean days) | | | | | | |
| 257 (1 non-randomised study) | very serious[a] | not serious | not serious | very serious[g,h] | none | ⨁◯◯◯ Very low[a,g,h] |
| Time to antidiabetic treatment intensification (assessed with: Mean days) | | | | | | |
| 56 (1 non-randomised study) | very serious[a] | not serious | not serious | very serious[g,h] | none | ⨁◯◯◯ Very low[a,g,h] |

| Diabetes effectiveness outcomes | | | | | | |
|---|---|---|---|---|---|---|
| Participants (studies) | Risk of bias | Inconsistency | Indirectness | Imprecision | Publication bias | Overall certainty of evidence |
| Achieving 1.0% decrease in HbA1c levels (assessed with: Yes/No) | | | | | | |
| 103<br>(1 RCT) | serious[f] | not serious | not serious | very serious[g,h] | none | ⨁◯◯◯<br>Very low[f,g,h] |
| Fasting blood glucose levels (assessed with: Mean) | | | | | | |
| 215<br>(1 RCT) | serious[f] | not serious | not serious | very serious[g,h] | none | ⨁◯◯◯<br>Very low[f,g,h] |
| Blood pressure goals achieved (assessed with: Yes/No) | | | | | | |
| 5500<br>(1 non-randomised study) | very serious[a] | not serious | not serious | serious[g] | none | ⨁◯◯◯<br>Very low[a,g] |
| 5655<br>(2 non-randomised studies) | very serious[a] | very serious[b,c,i] | not serious | not serious | none | ⨁◯◯◯<br>Very low[a,b,c,i] |
| Systolic blood pressure levels (assessed with: Mean) | | | | | | |
| 5655<br>(2 non-randomised studies) | very serious[a] | very serious[b,c,i] | not serious | not serious | none | ⨁◯◯◯<br>Very low[a,b,c,i] |
| HDL cholesterol levels (assessed with: Mean) | | | | | | |
| 150<br>(1 non-randomised study) | very serious[a] | not serious | not serious | very serious[g,h] | none | ⨁◯◯◯<br>Very low[a,g,h] |
| LDL cholesterol levels (assessed with: Mean) | | | | | | |

| Diabetes effectiveness outcomes | | | | | | |
|---|---|---|---|---|---|---|
| Participants (studies) | Risk of bias | Inconsistency | Indirectness | Imprecision | Publication bias | Overall certainty of evidence |
| 5655 (2 non-randomised studies) | very serious[a] | serious[b] | not serious | not serious | none | ⊕◯◯◯ Very low[a,b] |
| LDL cholesterol goal achieved (assessed with: Yes/No) | | | | | | |
| 5500 (1 non-randomised study) | very serious[a] | not serious | not serious | serious[g] | none | ⊕◯◯◯ Very low[a,g] |
| 248 (1 RCT) | very serious[a] | not serious | not serious | serious[g] | none | ⊕◯◯◯ Very low[a,g] |
| Triglycerides levels (assessed with: Mean) | | | | | | |
| 142 (1 non-randomised study) | very serious[a] | not serious | not serious | serious[g,h] | none | ⊕◯◯◯ Very low[a,g,h] |

Explanations
a. All studies were high risk of bias
b. Unpooled findings
c. Inconsistent effect across studies
d. Wide confidence intervals
e. Variations in effect ranges
f. Some studies had some concerns risk of bias
g. Single study
h. Small sample size
i. I-squared >75 in exploratory meta analyses

| Heart failure effectiveness outcomes | | | | | | |
|---|---|---|---|---|---|---|
| Participants (studies) | Risk of bias | Inconsistency | Indirectness | Imprecision | Publication bias | Overall certainty of evidence |
| 30-day all-cause readmission rate (assessed with: No. events) | | | | | | |
| 63 (1 non-randomised study) | very serious[a] | not serious | not serious | extremely serious[b,c] | none | ⊕◯◯◯ Very low[a,b,c] |
| 30-day all-cause readmission rate (assessed with: No. events) | | | | | | |
| 60 (1 non-randomised study) | very serious[a] | not serious | not serious | extremely serious[b,c] | none | ⊕◯◯◯ Very low[a,b,c] |
| 30-day heart failure readmission rate (assessed with: No. events) | | | | | | |
| 63 (1 non-randomised study) | very serious[a] | not serious | not serious | extremely serious[b,c] | none | ⊕◯◯◯ Very low[a,b,c] |
| 30-day heart failure readmission rate (assessed with: No. events) | | | | | | |
| 60 (1 non-randomised study) | very serious[a] | not serious | not serious | extremely serious[b,c] | none | ⊕◯◯◯ Very low[a,b,c] |
| Emergency department visits (assessed with: No. events) | | | | | | |
| 63 (1 non-randomised study) | very serious[a] | not serious | not serious | extremely serious[b,c] | none | ⊕◯◯◯ Very low[a,b,c] |
| Emergency department visits (assessed with: No. events) | | | | | | |

| Heart failure effectiveness outcomes | | | | | | |
|---|---|---|---|---|---|---|
| Participants (studies) | Risk of bias | Inconsistency | Indirectness | Imprecision | Publication bias | Overall certainty of evidence |
| 60 (1 non-randomised study) | very serious[a] | not serious | not serious | extremely serious[b,c] | none | ⊕◯◯◯ Very low[a,b,c] |
| Angiotensin receptor neprilysin inhibitor (ARNI) target dose achieved (assessed with: Yes/No) | | | | | | |
| 791 (1 non-randomised study) | very serious[a] | not serious | not serious | serious[b] | none | ⊕◯◯◯ Very low[a,b] |
| Number of visits required to achieve target dose (assessed with: Mean ) | | | | | | |
| 791 (1 non-randomised study) | very serious[a] | not serious | not serious | serious[b] | none | ⊕◯◯◯ Very low[a,b] |
| Time to achieve target/maximally tolerated angiotensin receptor neprilysin inhibitor (ARNI) dose (assessed with: Mean days) | | | | | | |
| 791 (1 non-randomised study) | very serious[a] | not serious | not serious | serious[b] | none | ⊕◯◯◯ Very low[a,b] |
| Aspirin deprescribed (assessed with: Yes/No) | | | | | | |
| 122 (1 non-randomised study) | very serious[a] | not serious | not serious | very serious[b,c] | none | ⊕◯◯◯ Very low[a,b,c] |

Explanations
a. All studies were high risk of bias
b. Single study
c. Small sample size

| Stroke effectiveness outcomes | | | | | | |
|---|---|---|---|---|---|---|
| Participants (studies) | Risk of bias | Inconsistency | Indirectness | Imprecision | Publication bias | Overall certainty of evidence |
| Systolic blood pressure levels (assessed with: Mean) | | | | | | |
| 279 (1 RCT) | very serious[a] | not serious | not serious | serious[b] | none | ⊕◯◯◯ Very low[a,b] |
| LDL cholesterol levels (assessed with: Mean) | | | | | | |
| 279 (1 RCT) | very serious[a] | not serious | not serious | serious[b] | none | ⊕◯◯◯ Very low[a,b] |
| Change in HDL cholesterol levels (assessed with: Mean change) | | | | | | |
| 279 (1 RCT) | very serious[a] | not serious | not serious | serious[b] | none | ⊕◯◯◯ Very low[a,b] |
| Adherence (assessed with: Yes/No, self-reported adherence of 75% of higher to blood pressure and lipid lower medications) | | | | | | |
| 279 (1 RCT) | very serious[a] | not serious | not serious | serious[b] | none | ⊕◯◯◯ Very low[a,b] |
| Health (assessed with: Self-reported) | | | | | | |
| 279 (1 RCT) | very serious[a] | not serious | not serious | serious[b] | none | ⊕◯◯◯ Very low[a,b] |
| Quality of life (assessed with: EQ-5D) | | | | | | |
| 279 (1 RCT) | very serious[a] | not serious | not serious | serious[b] | none | ⊕◯◯◯ Very low[a,b] |

Explanations

a. Most studies are high risk of bias

b. Single study

| Dyslipidaemia effectiveness outcomes | | | | | | |
|---|---|---|---|---|---|---|
| Participants (studies) | Risk of bias | Inconsistency | Indirectness | Imprecision | Publication bias | Overall certainty of evidence |
| Target lipid levels achieved (assessed with: Yes/No) | | | | | | |
| 223 (1 RCT) | serious[a] | not serious | not serious | serious[b,c] | none | ⊕⊕◯◯ Low[a,b,c] |
| LDL cholesterol levels (assessed with: Mean) | | | | | | |
| 211 (1 RCT) | serious[a] | not serious | not serious | serious[b,c] | none | ⊕⊕◯◯ Low[a,b,c] |
| HDL cholesterol levels (assessed with: Mean) | | | | | | |
| 225 (1 RCT) | serious[a] | not serious | not serious | serious[b,c] | none | ⊕⊕◯◯ Low[a,b,c] |
| Triglyceride levels (assessed with: Mean) | | | | | | |
| 225 (1 RCT) | serious[a] | not serious | not serious | serious[b,c] | none | ⊕⊕◯◯ Low[a,b,c] |
| Systolic blood pressure levels (assessed with: Mean) | | | | | | |
| 225 (1 RCT) | serious[a] | not serious | not serious | serious[b,c] | none | ⊕⊕◯◯ Low[a,b,c] |
| Diastolic blood pressure levels (assessed with: Mean) | | | | | | |
| 225 (1 RCT) | serious[a] | not serious | not serious | serious[b,c] | none | ⊕⊕◯◯ Low[a,b,c] |
| Fasting blood glucose levels (assessed with: Mean) | | | | | | |
| 225 (1 RCT) | serious[a] | not serious | not serious | serious[b,c] | none | ⊕⊕◯◯ Low[a,b,c] |
| Healthcare utilisation (No. physician visits) (assessed with: Mean) | | | | | | |

| Dyslipidaemia effectiveness outcomes | | | | | | |
|---|---|---|---|---|---|---|
| Participants (studies) | Risk of bias | Inconsistency | Indirectness | Imprecision | Publication bias | Overall certainty of evidence |
| 225<br>(1 RCT) | serious[a] | not serious | not serious | serious[b,c] | none | ⊕⊕◯◯<br>Low[a,b,c] |
| Adherence (assessed with: Yes/No ?) | | | | | | |
| 215<br>(1 RCT) | serious[a] | not serious | not serious | serious[b,c] | none | ⊕⊕◯◯<br>Low[a,b,c] |

Explanations
a. Some studies were some concern risk of bias
b. Single study
c. Small sample size

| Hypertension certainty outcomes | | | | | | |
|---|---|---|---|---|---|---|
| Participants (studies) Follow-up | Risk of bias | Inconsistency | Indirectness | Imprecision | Publication bias | Overall certainty of evidence |
| Blood pressure goal achieved (assessed with: Yes/No) | | | | | | |
| 126<br>(1 RCT) | very serious[a] | not serious | not serious | very serious[b,c] | none | ⊕◯◯◯<br>Very low[a,b,c] |
| Blood pressure goal achieved (assessed with: Yes/No) | | | | | | |
| 126<br>(1 non-randomised study) | very serious[a] | not serious | not serious | very serious[b,c] | none | ⊕◯◯◯<br>Very low[a,b,c] |
| Systolic blood pressure levels (assessed with: Mean) | | | | | | |
| 53<br>(1 non-randomised study) | very serious[a] | not serious | not serious | extremely serious[b,c] | none | ⊕◯◯◯<br>Very low[a,b,c] |

| Hypertension certainty outcomes | | | | | | |
|---|---|---|---|---|---|---|
| Change in systolic blood pressure levels (assessed with: Mean change ) | | | | | | |
| 126<br>(1 non-randomised study) | very serious[a] | not serious | not serious | very serious[b,c] | none | ⊕◯◯◯<br>Very low[a,b,c] |
| Change in systolic blood pressure (assessed with: Mean change) | | | | | | |
| 126<br>(1 RCT) | very serious[a] | not serious | not serious | very serious[b,c] | none | ⊕◯◯◯<br>Very low[a,b,c] |
| Diastolic blood pressure levels (assessed with: Mean) | | | | | | |
| 53<br>(1 non-randomised study) | very serious[a] | not serious | not serious | extremely serious[b,c] | none | ⊕◯◯◯<br>Very low[a,b,c] |
| Change in diastolic blood pressure (assessed with: Mean change) | | | | | | |
| 126<br>(1 RCT) | very serious[a] | not serious | not serious | very serious[b,c] | none | ⊕◯◯◯<br>Very low[a,b,c] |
| Change in diastolic blood pressure (assessed with: Mean change) | | | | | | |
| 126<br>(1 non-randomised study) | very serious[a] | not serious | not serious | very serious[b,c] | none | ⊕◯◯◯<br>Very low[a,b,c] |
| Adherence (assessed with: No. refills) | | | | | | |
| 53<br>(1 RCT) | very serious[a] | not serious | not serious | extremely serious[b,c] | none | ⊕◯◯◯<br>Very low[a,b,c] |
| Quality of life (assessed with: Mean) | | | | | | |
| 52<br>(1 RCT) | very serious[a] | not serious | not serious | extremely serious[b,c] | none | ⊕◯◯◯<br>Very low[a,b,c] |

Explanations
a. High risk of bias in all studies
b. Single study
c. Small sample size

| Coagulation disorders certainty outcomes | | | | | | |
|---|---|---|---|---|---|---|
| Participants (studies) | Risk of bias | Inconsistency | Indirectness | Imprecision | Publication bias | Overall certainty of evidence |
| Time to achieve therapeutic INR (assessed with: Mean days) | | | | | | |
| 51 (1 non-randomised study) | very serious[a] | not serious | not serious | very serious[b,c] | none | ⊕◯◯◯ Very low[a,b,c] |
| INR control achieved (assessed with: Yes/No) | | | | | | |
| 138 (1 non-randomised study) | very serious[a] | not serious | not serious | very serious[b,c] | none | ⊕◯◯◯ Very low[a,b,c] |
| INR within therapeutic range (assessed with: Yes/No) | | | | | | |
| 350 (1 non-randomised study) | very serious[a] | not serious | not serious | serious[b] | none | ⊕◯◯◯ Very low[a,b] |
| Difference between groups in those achieving INR within therapeutic range (assessed with: Mean change) | | | | | | |
| 200 (1 non-randomised study) | very serious[a] | not serious | not serious | serious[b] | none | ⊕◯◯◯ Very low[a,b] |
| Time to achieve therapeutic proconvertin and prothrombin (assessed with: Mean days) | | | | | | |
| 81 (1 RCT) | very serious[a] | not serious | not serious | very serious[b,c] | none | ⊕◯◯◯ Very low[a,b,c] |
| Partial thromboplastic time (assessed with: Mean seconds) | | | | | | |
| 81 (1 RCT) | very serious[a] | not serious | not serious | very serious[b,c] | none | ⊕◯◯◯ Very low[a,b,c] |
| Prothrombin time ratio (assessed with: Time ratio) | | | | | | |
| 95 (1 non-randomised study) | very serious[a] | not serious | not serious | very serious[b,c] | none | ⊕◯◯◯ Very low[a,b,c] |

Explanations

a. All studies were high risk of bias

b. Single study

c. Small sample size

| Urinary tract infection certainty outcomes | | | | | | |
|---|---|---|---|---|---|---|
| Participants (studies) | Risk of bias | Inconsistency | Indirectness | Imprecision | Publication bias | Overall certainty of evidence |
| Clinical cure (assessed with: Yes/No) | | | | | | |
| 686 (1 non-randomised study) | very serious[a] | not serious | not serious | serious[b] | none | ⨁◯◯◯ Very low[a,b] |
| Waiting time (assessed with: Mean days to access care) | | | | | | |
| 750 (1 non-randomised study) | very serious[a] | not serious | not serious | serious[b] | none | ⨁◯◯◯ Very low[a,b] |
| Adherence (assessed with: Yes/No taken as prescribed) | | | | | | |
| 686 (1 non-randomised study) | very serious[a] | not serious | not serious | serious[b] | none | ⨁◯◯◯ Very low[a,b] |

Explanations

a. Most studies were high risk of bias

b. Single study

| Older people in long-term care effectiveness outcomes | | | | | | |
|---|---|---|---|---|---|---|
| Participants (studies) | Risk of bias | Inconsistency | Indirectness | Imprecision | Publication bias | Overall certainty of evidence |
| Falls (assessed with: Mean) | | | | | | |
| 968 (2 RCTs) | very serious[a] | serious[b] | not serious | serious[c] | none | ⨁◯◯◯ Very low[a,b,c] |

| Older people in long-term care effectiveness outcomes | | | | | | |
|---|---|---|---|---|---|---|
| Participants (studies) | Risk of bias | Inconsistency | Indirectness | Imprecision | Publication bias | Overall certainty of evidence |
| Systolic blood pressure goal achieved (assessed with: Yes/No) | | | | | | |
| 92<br>(1 RCT) | serious[d] | not serious | not serious | extremely serious[e,f] | none | ⊕◯◯◯<br>Very low[d,e,f] |
| Drug burden (assessed with: Drug burden index) | | | | | | |
| 449<br>(1 RCT) | very serious[a] | not serious | not serious | serious[e] | none | ⊕◯◯◯<br>Very low[a,e] |
| Drug burden (assessed with: Mean no. drugs per patient) | | | | | | |
| 139<br>(1 non-randomised study) | very serious[a] | not serious | not serious | very serious[e,f] | none | ⊕◯◯◯<br>Very low[a,e,f] |
| Health-related quality of life (assessed with: Standardised scale?) | | | | | | |
| 968<br>(2 RCTs) | very serious[a] | serious[b] | not serious | serious[c] | none | ⊕◯◯◯<br>Very low[a,b,c] |
| Depression (assessed with: HADS?) | | | | | | |
| 92<br>(1 RCT) | serious[d] | not serious | not serious | extremely serious[e,f] | none | ⊕◯◯◯<br>Very low[d,e,f] |
| Anxiety (assessed with: HADS?) | | | | | | |
| 92<br>(1 RCT) | serious[d] | not serious | not serious | extremely serious[e,f] | none | ⊕◯◯◯<br>Very low[d,e,f] |
| Hospitalisations (assessed with: Mean) | | | | | | |
| 968<br>(2 RCTs) | very serious[a] | not serious | not serious | not serious | none | ⊕⊕◯◯<br>Low[a] |
| Hospitalisations (assessed with: Mean) | | | | | | |
| 139<br>(1 non-randomised study) | very serious[a] | not serious | not serious | very serious[e,f] | none | ⊕◯◯◯<br>Very low[a,e,f] |
| Emergency department visits (assessed with: Mean) | | | | | | |

| Older people in long-term care effectiveness outcomes | | | | | | |
|---|---|---|---|---|---|---|
| Participants (studies) | Risk of bias | Inconsistency | Indirectness | Imprecision | Publication bias | Overall certainty of evidence |
| 92<br>(1 RCT) | serious[d] | not serious | not serious | extremely serious[e,f] | none | ⊕▯▯▯<br>Very low[d,e,f] |

Explanations

a. Most studies were high risk of bias
b. Inconsistent effect across studies
c. Variations in effect ranges
d. Some studies had some risk of concerns
e. Single study
f. Small sample size

| Female contraceptive users effectiveness outcomes | | | | | | |
|---|---|---|---|---|---|---|
| Participants (studies) | Risk of bias | Inconsistency | Indirectness | Imprecision | Publication bias | Overall certainty of evidence |
| Continuation (follow-up: 6 months; assessed with: Yes/No) | | | | | | |
| 172665<br>(2 non-randomised studies) | very serious[a] | serious[b] | not serious | serious[c] | none | ⊕▯▯▯<br>Very low[a,b,c] |
| Adherence (follow-up: 6 months; assessed with: Yes/No) | | | | | | |
| 172665<br>(2 non-randomised studies) | very serious[a] | not serious | not serious | serious[c] | none | ⊕▯▯▯<br>Very low[a,c] |

Explanations

a. All studies were high risk of bias
b. Inconsistent effect across studies
c. Wide confidence intervals

| Anaemia in pregnancy effectiveness outcomes | | | | | | |
|---|---|---|---|---|---|---|
| Participants (studies) | Risk of bias | Inconsistency | Indirectness | Imprecision | Publication bias | Overall certainty of evidence |
| Haemoglobin goal achieved (assessed with: Yes/No) | | | | | | |
| 200 (1 non-randomised study) | very serious[a] | not serious | not serious | serious[b] | none | ⨁◯◯◯ Very low[a,b] |
| Haemoglobin levels (assessed with: Mean) | | | | | | |
| 200 (1 non-randomised study) | very serious[a] | not serious | not serious | serious[b] | none | ⨁◯◯◯ Very low[a,b] |

Explanations
a. Most studies were high risk of bias
b. Single study

| Chronic pain conditions effectiveness outcomes | | | | | | |
|---|---|---|---|---|---|---|
| Participants (studies) | Risk of bias | Inconsistency | Indirectness | Imprecision | Publication bias | Overall certainty of evidence |
| Quality of life (physical) (assessed with: EQ-5D scale) | | | | | | |
| 84 (1 RCT) | very serious[a] | not serious | not serious | extremely serious[b,c] | none | ⨁◯◯◯ Very low[a,b,c] |
| Quality of life (physical) (assessed with: EQ-5D) | | | | | | |
| 86 (1 RCT) | very serious[a] | not serious | not serious | extremely serious[b,c] | none | ⨁◯◯◯ Very low[a,b,c] |
| Quality of life (psychological) (assessed with: EQ-5D) | | | | | | |

| Chronic pain conditions effectiveness outcomes | | | | | | |
|---|---|---|---|---|---|---|
| Participants (studies) | Risk of bias | Inconsistency | Indirectness | Imprecision | Publication bias | Overall certainty of evidence |
| 85 (1 RCT) | very serious[a] | not serious | not serious | extremely serious[b,c] | none | ⨁◯◯◯ Very low[a,b,c] |
| Quality of life (psychological) (assessed with: EQ-5D) | | | | | | |
| 87 (1 RCT) | very serious[a] | not serious | not serious | extremely serious[b,c] | none | ⨁◯◯◯ Very low[a,b,c] |
| Chronic pain intensity (assessed with: Chronic Pain Scale) | | | | | | |
| 89 (1 RCT) | very serious[a] | not serious | not serious | extremely serious[b,c] | none | ⨁◯◯◯ Very low[a,b,c] |
| Chronic pain intensity (assessed with: Chronic pain scale) | | | | | | |
| 97 (1 RCT) | very serious[a] | not serious | not serious | extremely serious[b,c] | none | ⨁◯◯◯ Very low[a,b,c] |
| Chronic pain disability (assessed with: Chronic Pain Scale) | | | | | | |
| 94 (1 RCT) | very serious[a] | not serious | not serious | extremely serious[b,c] | none | ⨁◯◯◯ Very low[a,b,c] |
| Chronic pain disability (assessed with: Chronic Pain Scale) | | | | | | |
| 101 (1 RCT) | very serious[a] | not serious | not serious | very serious[b,c] | none | ⨁◯◯◯ Very low[a,b,c] |
| Depression (assessed with: HADS-D) | | | | | | |
| 86 (1 RCT) | very serious[a] | not serious | not serious | extremely serious[b,c] | none | ⨁◯◯◯ Very low[a,b,c] |
| Depression (assessed with: HADS-D) | | | | | | |
| 93 (1 RCT) | very serious[a] | not serious | not serious | extremely serious[b,c] | none | ⨁◯◯◯ Very low[a,b,c] |
| Anxiety (assessed with: HADS-A) | | | | | | |

| Chronic pain conditions effectiveness outcomes | | | | | | |
|---|---|---|---|---|---|---|
| Participants (studies) | Risk of bias | Inconsistency | Indirectness | Imprecision | Publication bias | Overall certainty of evidence |
| 87<br>(1 RCT) | very serious[a] | not serious | not serious | extremely serious[b,c] | none | ⊕□□□<br>Very low[a,b,c] |
| Anxiety (assessed with: HADS-A) | | | | | | |
| 92<br>(1 RCT) | very serious[a] | not serious | not serious | extremely serious[b,c] | none | ⊕□□□<br>Very low[a,b,c] |

Explanations
a. Most studies are high risk of bias
b. Single study
c. Small sample size

| Mixed health conditions effectiveness outcomes | | | | | | |
|---|---|---|---|---|---|---|
| Participants (studies) | Risk of bias | Inconsistency | Indirectness | Imprecision | Publication bias | Overall certainty of evidence |
| HbA1c goal achieved (assessed with: Yes/No) | | | | | | |
| 215<br>(1 non-randomised study) | very serious[a] | not serious | not serious | serious[b] | none | ⊕□□□<br>Very low[a,b] |
| HbA1c goal achieved (assessed with: Yes/No) | | | | | | |
| 235<br>(1 non-randomised study) | very serious[a] | not serious | not serious | serious[b] | none | ⊕□□□<br>Very low[a,b] |
| Blood pressure goal achieved (assessed with: Yes/No) | | | | | | |
| 605<br>(1 non-randomised study) | very serious[a] | not serious | not serious | serious[b] | none | ⊕□□□<br>Very low[a,b] |

| Mixed health conditions effectiveness outcomes | | | | | | |
|---|---|---|---|---|---|---|
| Participants (studies) | Risk of bias | Inconsistency | Indirectness | Imprecision | Publication bias | Overall certainty of evidence |
| Blood pressure goal achieved (assessed with: Yes/No) | | | | | | |
| 614 (1 non-randomised study) | very serious[a] | not serious | not serious | serious[b] | none | ⊕◯◯◯ Very low[a,b] |
| LDL cholesterol goal achieved (assessed with: Yes/No) | | | | | | |
| 314 (1 non-randomised study) | very serious[a] | not serious | not serious | serious[b] | none | ⊕◯◯◯ Very low[a,b] |
| LDL cholesterol goal achieved (assessed with: Yes/No) | | | | | | |
| 325 (1 non-randomised study) | very serious[a] | not serious | not serious | serious[b] | none | ⊕◯◯◯ Very low[a,b] |
| Clinic visits (assessed with: Relative risk) | | | | | | |
| 605 (1 non-randomised study) | very serious[a] | not serious | not serious | serious[b] | none | ⊕◯◯◯ Very low[a,b] |
| Clinic visits (assessed with: Relative risk) | | | | | | |
| 614 (1 non-randomised study) | very serious[a] | not serious | not serious | serious[b] | none | ⊕◯◯◯ Very low[a,b] |
| Hospitalisations (assessed with: Relative risk) | | | | | | |
| 605 (1 non-randomised study) | very serious[a] | not serious | not serious | serious[b] | none | ⊕◯◯◯ Very low[a,b] |
| Hospitalisations (assessed with: Relative risk) | | | | | | |
| 614 (1 non-randomised study) | very serious[a] | not serious | not serious | serious[b] | none | ⊕◯◯◯ Very low[a,b] |
| Hospitalisations (assessed with: No. events) | | | | | | |

| Mixed health conditions effectiveness outcomes | | | | | | |
|---|---|---|---|---|---|---|
| Participants (studies) | Risk of bias | Inconsistency | Indirectness | Imprecision | Publication bias | Overall certainty of evidence |
| 753<br>(1 RCT) | very serious[a] | not serious | not serious | serious[b] | none | ⊕□□□<br>Very low[a,b] |
| Length of hospital stay (assessed with: Mean no. days) | | | | | | |
| 762<br>(1 RCT) | very serious[a] | not serious | not serious | serious[b] | none | ⊕□□□<br>Very low[a,b] |
| Emergency department visits (assessed with: Relative risk) | | | | | | |
| 605<br>(1 non-randomised study) | very serious[a] | not serious | not serious | serious[b] | none | ⊕□□□<br>Very low[a,b] |
| Emergency department visits (assessed with: Relative risk) | | | | | | |
| 614<br>(1 non-randomised study) | very serious[a] | not serious | not serious | serious[b] | none | ⊕□□□<br>Very low[a,b] |

Explanations

a. Most studies have high risk of bias

b. Single study

| Heart failure safety outcomes | | | | | | |
|---|---|---|---|---|---|---|
| Participants (studies) | Risk of bias | Inconsistency | Indirectness | Imprecision | Publication bias | Overall certainty of evidence |
| Hospitalisations due to heart failure (assessed with: Yes/No) | | | | | | |
| 791<br>(1 non-randomised study) | very serious[a] | not serious | not serious | serious[b] | none | ⊕□□□<br>Very low[a,b] |

| Heart failure safety outcomes | | | | | | |
|---|---|---|---|---|---|---|
| Participants (studies) | Risk of bias | Inconsistency | Indirectness | Imprecision | Publication bias | Overall certainty of evidence |
| Mortality (assessed with: No. events) | | | | | | |
| 791 (1 non-randomised study) | very serious[a] | not serious | not serious | serious[b] | none | ⊕◯◯◯ Very low[a,b] |

Explanations
a. Most studies were high risk of bias
b. Single study

| Stroke safety outcomes | | | | | | |
|---|---|---|---|---|---|---|
| Participants (studies) | Risk of bias | Inconsistency | Indirectness | Imprecision | Publication bias | Overall certainty of evidence |
| Mortality (assessed with: No. events) | | | | | | |
| 279 (1 RCT) | very serious[a] | not serious | not serious | serious[b] | none | ⊕◯◯◯ Very low[a,b] |
| Vascular adverse event (assessed with: No. events) | | | | | | |
| 279 (1 RCT) | very serious[a] | not serious | not serious | serious[b] | none | ⊕◯◯◯ Very low[a,b] |

Explanations
a. Most studies were high risk of bias
b. Single study

| Dyslipidaemia safety outcomes | | | | | | |
|---|---|---|---|---|---|---|
| Participants (studies) | Risk of bias | Inconsistency | Indirectness | Imprecision | Publication bias | Overall certainty of evidence |
| Adverse events | | | | | | |
| 211<br>(1 RCT) | serious[a] | not serious | not serious | serious[b] | none | ⊕⊕◯◯<br>Low[a,b] |

Explanations
a. Some studies were some concerns risk of bias
b. Single study

| Coagulation disorder safety outcomes | | | | | | |
|---|---|---|---|---|---|---|
| Participants (studies) | Risk of bias | Inconsistency | Indirectness | Imprecision | Publication bias | Overall certainty of evidence |
| Warfarin-related complications (bleeding) | | | | | | |
| 95<br>(1 non-randomised study) | very serious[a] | not serious | not serious | extremely serious[b,c] | none | ⊕◯◯◯<br>Very low[a,b,c] |
| Thromboembolic adverse events | | | | | | |
| 95<br>(1 non-randomised study) | very serious[a] | not serious | not serious | extremely serious[b,c] | none | ⊕◯◯◯<br>Very low[a,b,c] |
| Anticoagulation-related adverse events | | | | | | |
| 350<br>(1 non-randomised study) | very serious[a] | not serious | not serious | very serious[b] | none | ⊕◯◯◯<br>Very low[a,b] |
| Bleeds/adverse drug events | | | | | | |
| 51<br>(1 non-randomised study) | very serious[a] | not serious | not serious | extremely serious[b,c] | none | ⊕◯◯◯<br>Very low[a,b,c] |
| Anticoagulation-related hospital admissions | | | | | | |

| Coagulation disorder safety outcomes | | | | | | |
|---|---|---|---|---|---|---|
| Participants (studies) | Risk of bias | Inconsistency | Indirectness | Imprecision | Publication bias | Overall certainty of evidence |
| 350 (1 non-randomised study) | very serious[a] | not serious | not serious | very serious[b] | none | ⊕◯◯◯ Very low[a,b] |
| Anticoagulation-related emergency department visits | | | | | | |
| 350 (1 non-randomised study) | very serious[a] | not serious | not serious | very serious[b] | none | ⊕◯◯◯ Very low[a,b] |
| Warfarin related hospitalisations/ED visits | | | | | | |
| 200 (1 non-randomised study) | very serious[a] | not serious | not serious | very serious[b,d] | none | ⊕◯◯◯ Very low[a,b,d] |

Explanations
a. Most studies were high risk of bias
b. Single study
c. Small sample size
d. Wide confidence intervals

| Urinary tract infection safety outcomes | | | | | | |
|---|---|---|---|---|---|---|
| Participants (studies) | Risk of bias | Inconsistency | Indirectness | Imprecision | Publication bias | Overall certainty of evidence |
| All adverse events | | | | | | |
| 686 (1 non-randomised study) | very serious[a] | not serious | not serious | serious[b] | none | ⊕◯◯◯ Very low[a,b] |
| Gastrointestinal adverse events | | | | | | |
| 686 (1 non-randomised study) | very serious[a] | not serious | not serious | serious[b] | none | ⊕◯◯◯ Very low[a,b] |

| Urinary tract infection safety outcomes | | | | | | |
|---|---|---|---|---|---|---|
| Participants (studies) | Risk of bias | Inconsistency | Indirectness | Imprecision | Publication bias | Overall certainty of evidence |
| Vaginal candidiasis adverse events | | | | | | |
| 686 (1 non-randomised study) | very serious[a] | not serious | not serious | serious[b] | none | ⊕▯▯▯ Very low[a,b] |
| Headache adverse events | | | | | | |
| 686 (1 non-randomised study) | very serious[a] | not serious | not serious | serious[b] | none | ⊕▯▯▯ Very low[a,b] |
| Other adverse events | | | | | | |
| 686 (1 non-randomised study) | very serious[a] | not serious | not serious | serious[b] | none | ⊕▯▯▯ Very low[a,b] |
| Physician or emergency department events | | | | | | |
| 686 (1 non-randomised study) | very serious[a] | not serious | not serious | serious[b] | none | ⊕▯▯▯ Very low[a,b] |
| Antibacterial therapy guideline concordance | | | | | | |
| 686 (1 non-randomised study) | very serious[a] | not serious | not serious | serious[b] | none | ⊕▯▯▯ Very low[a,b] |

Explanations

a. Most studies were high risk of bias

b. Single study

| Older people in long-term care safety outcomes | | | | | | |
|---|---|---|---|---|---|---|
| Participants (studies) | Risk of bias | Inconsistency | Indirectness | Imprecision | Publication bias | Overall certainty of evidence |
| Mortality (assessed with: No. events) | | | | | | |
| 876<br>(1 RCT) | very serious[a] | not serious | not serious | serious[b] | none | ⊕◯◯◯<br>Very low[a,b] |
| Mortality (assessed with: No. events) | | | | | | |
| 139<br>(1 non-randomised study) | very serious[a] | not serious | not serious | very serious[b,c] | none | ⊕◯◯◯<br>Very low[a,b,c] |
| Syncope | | | | | | |
| 92<br>(1 RCT) | serious[d] | not serious | not serious | extremely serious[b,c] | none | ⊕◯◯◯<br>Very low[b,c,d] |
| Hypotension | | | | | | |
| 92<br>(1 RCT) | serious[d] | not serious | not serious | extremely serious[b,c] | none | ⊕◯◯◯<br>Very low[b,c,d] |
| Hypokalemia | | | | | | |
| 92<br>(1 RCT) | serious[d] | not serious | not serious | extremely serious[b,c] | none | ⊕◯◯◯<br>Very low[b,c,d] |
| Hyperkalemia | | | | | | |
| 92<br>(1 RCT) | serious[d] | not serious | not serious | extremely serious[b,c] | none | ⊕◯◯◯<br>Very low[b,c,d] |
| Hyponatremia | | | | | | |
| 92<br>(1 RCT) | serious[d] | not serious | not serious | extremely serious[b,c] | none | ⊕◯◯◯<br>Very low[b,c,d] |
| Orthostatic presyncope | | | | | | |

| Older people in long-term care safety outcomes | | | | | | |
|---|---|---|---|---|---|---|
| Participants (studies) | Risk of bias | Inconsistency | Indirectness | Imprecision | Publication bias | Overall certainty of evidence |
| 92<br>(1 RCT) | serious[d] | not serious | not serious | extremely serious[b,c] | none | ⨁□□□<br>Very low[b,c,d] |
| Change in eGFR | | | | | | |
| 92<br>(1 RCT) | serious[d] | not serious | not serious | extremely serious[b,c] | none | ⨁□□□<br>Very low[b,c,d] |

Explanations

a. Most studies were high risk of bias

b. Single study

c. Small sample size

d. Some studies were some concerns risk of bias

| Female contraceptive users safety outcomes | | | | | | |
|---|---|---|---|---|---|---|
| Participants (studies) | Risk of bias | Inconsistency | Indirectness | Imprecision | Publication bias | Overall certainty of evidence |
| Medical contraindications (assessed with: Yes/No) | | | | | | |
| 439650<br>(2 non-randomised studies) | very serious[a] | not serious | not serious | not serious | none | ⨁□□□<br>Very low[a] |

Explanations

a. Most studies were high risk of bias

| Surgery patients safety outcomes | | | | | | |
|---|---|---|---|---|---|---|
| Participants (studies) | Risk of bias | Inconsistency | Indirectness | Imprecision | Publication bias | Overall certainty of evidence |
| Prescribing errors (assessed with: No. events) | | | | | | |
| 73<br>(1 RCT) | very serious[a] | not serious | not serious | extremely serious[b,c] | none | ⊕□□□<br>Very low[a,b,c] |

Explanations
a. Most studies were high risk of bias
b. Single study
c. Small sample size

| People at risk of drug-related problems outcomes | | | | | | |
|---|---|---|---|---|---|---|
| Participants (studies) | Risk of bias | Inconsistency | Indirectness | Imprecision | Publication bias | Overall certainty of evidence |
| Mortality (assessed with: Yes/No) | | | | | | |
| 762<br>(1 RCT) | very serious[a] | not serious | not serious | very serious[b] | none | ⊕□□□<br>Very low[a,b] |

Explanations
a. Most studies were high risk of bias
b. Single study

| Mixed health conditions safety outcomes | | | | | | |
|---|---|---|---|---|---|---|
| Participants (studies) | Risk of bias | Inconsistency | Indirectness | Imprecision | Publication bias | Overall certainty of evidence |
| Gastrointestinal (GI) bleeding events (hospitalisations and ER visits) | | | | | | |

| Mixed health conditions safety outcomes | | | | | | |
|---|---|---|---|---|---|---|
| Participants (studies) | Risk of bias | Inconsistency | Indirectness | Imprecision | Publication bias | Overall certainty of evidence |
| 2155 (1 non-randomised study) | very serious[a] | not serious | not serious | serious[b] | none | ⊕◯◯◯ Very low[a,b] |
| Acute kidney injury (AKI) events (hospitalisations and ER visits) | | | | | | |
| 2155 (1 non-randomised study) | very serious[a] | not serious | not serious | serious[b] | none | ⊕◯◯◯ Very low[a,b] |
| Pain (hospitalisations and ER visits) | | | | | | |
| 1805 (1 non-randomised study) | very serious[a] | not serious | not serious | serious[b] | none | ⊕◯◯◯ Very low[a,b] |

Explanations

a. Most studies were high risk of bias

b. Single study

# Appendix N Table of effectiveness findings

| Study author (year) | Health population | Outcome domain | Outcome | Study design | Prescriptive authority | Setting | No. participants | Effect estimates | Intervention group | Comparator group | p-value |
|---|---|---|---|---|---|---|---|---|---|---|---|
| Maeng *et al.* (2018) (38) | Diabetes | Blood sugar | HbA1C goal achieved | Retrospective cohort | CPA | Primary care | 5500 | No. events (%) | 1,396/2,750 (50.8%) | 1,564/2,750 (56.9%) | 0.0001*** |
| Cowart *et al.* (2022) (34) | Diabetes | Blood sugar | HbA1C goal achieved | Retrospective cohort | CPA | Primary care | 56 | No. events (%) | 7/28 (25.0%) | 5/28 (17.9%) | 0.61 |
| Cowart et al. (2020) (35) | Diabetes | Blood sugar | HbA1C goal achieved | Retrospective cohort | CPA | Primary care | 257 | No. events (%) | 38/76 (50.0%) | 78/181 (43.1%) | 0.31 |
| Morello *et al.* (2016) (37) | Diabetes | Blood sugar | Mean HbA1c levels | Retrospective cohort | CPA | Outpatient | 155 | Mean (SD) | 8.2% (1.9) | 9.0% (1.5) | 0.001** |
| Maeng et al. (2018) (38) | Diabetes | Blood sugar | Mean HbA1c levels | Retrospective cohort | CPA | Primary care | 5500 | Mean (SD) | 8.3% (1.8) | 8.0% (1.7) | 0.0001*** |
| Hernández-Muñoz et al. (2020) (39) | Diabetes | Blood sugar | Mean change in HbA1c | Retrospective cohort | CPA | Outpatient | 121 | Mean (SE) | −0.2 (0.3) | −0.02 (0.40) | 0.95 |
| Lum *et al.* (2023) (36) | Diabetes | Blood sugar | Mean change in HbA1c | Retrospective cohort | CPA | Outpatient | 420 | Mean difference | −0.4% | −0.2% | 0.001** |
| Morello et al. (2016) (37) | Diabetes | Blood sugar | Fasting blood glucose levels | Retrospective cohort | CPA | Outpatient | 155 | Mean (SD) | 159.0 (83.2) | 194.3 (112) | 0.08 |
| Jameson *et al.* (2010) (41) | Diabetes | Blood sugar | Mean change in HbA1c | RCT | CPA | Primary care | 104 | Median (IQR) | −1.50 (−0.03 to −2.68) | −0.40 (0.50 to −2.10) | 0.06 |

| Study author (year) | Health population | Outcome domain | Outcome | Study design | Prescriptive authority | Setting | No. participants | Effect estimates | Intervention group | Comparator group | p-value |
|---|---|---|---|---|---|---|---|---|---|---|---|
| Xu *et al.* (2021) (40) | Diabetes | Blood sugar | Mean change in HbA1c | RCT | CPA | Primary care | 248 | Mean (95% CI) | −0.50 (−0.24 to −0.75) | −0.11 (−0.20 to 0.42) | 0.03* |
| Jameson *et al.* (2010) (41) | Diabetes | Blood sugar | Minimum 1% decrease in HbA1c levels | RCT | CPA | Primary care | 104 | No. events (%) | 35/52 (67.3%) | 21/51 (41.2%) | 0.02* |
| Cowart et al. (2022) (34) | Diabetes | Treatment optimisation | Time to treatment intensification | Retrospective cohort | CPA | Primary care | 257 | Median (IQR) | 37.5 (8 to 216.5) | 142 (16 to 465) | 0.19 |
| Maeng et al. (2018) (38) | Diabetes | Blood pressure | Blood pressure goal achieved | Retrospective cohort | CPA | Primary care | 5500 | No. events (%) | 1,287/2,750 (46.8%) | 1,241/2,750 (45.1%) | 0.21 |
| Morello et al. (2016) (37) | Diabetes | Blood pressure | Diastolic blood pressure | Retrospective cohort | CPA | Outpatient | 155 | Mean (SD) | 71.8 (12.0) | 74.5 (14.9) | 0.59 |
| Maeng et al. (2018) (38) | Diabetes | Blood pressure | Diastolic blood pressure | Retrospective cohort | CPA | Primary care | 5500 | Mean (SD) | 72 (10) | 73 (10) | 0.7 |
| Morello et al. (2016) (37) | Diabetes | Blood pressure | Systolic blood pressure | Retrospective cohort | CPA | Outpatient | 155 | Mean (SD) | 127.0 (14.4) | 136.7 (20.0) | 0.11 |
| Maeng et al. (2018) (38) | Diabetes | Blood pressure | Systolic blood pressure | Retrospective cohort | CPA | Primary care | 5500 | Mean (SD) | 129 (16) | 129 (17) | 0.57 |
| Maeng et al. (2018) (38) | Diabetes | Lipids | LDL goal achieved | Retrospective cohort | CPA | Primary care | 5500 | No. events (%) | 1,138/2,750 (41.4%) | 1,078/2,750 (39.2%) | 0.08 |

| Study author (year) | Health population | Outcome domain | Outcome | Study design | Prescriptive authority | Setting | No. participants | Effect estimates | Intervention group | Comparator group | p-value |
|---|---|---|---|---|---|---|---|---|---|---|---|
| Morello et al. (2016) (37) | Diabetes | Lipids | Mean LDL cholesterol levels | Retrospective cohort | CPA | Outpatient | 155 | Mean (SD) | 84.0 (28.6) | 82.8 (32.0) | 0.58 |
| Maeng et al. (2018) (38) | Diabetes | Lipids | Mean LDL cholesterol levels | Retrospective cohort | CPA | Primary care | 5500 | Mean (SD) | 91 (37) | 92 (36) | 0.47 |
| Morello et al. (2016) (37) | Diabetes | Lipids | Mean HDL cholesterol | Retrospective cohort | CPA | Outpatient | 155 | Mean (SD) | 40.7 (11.8) | 42.9 (12.8) | 0.57 |
| Morello et al. (2016) (37) | Diabetes | Lipids | Mean triglyceride levels | Retrospective cohort | CPA | Outpatient | 155 | Mean (SD) | 185.9 (125.2) | 189.2 (164.5) | 0.33 |
| Xu *et al.* (2021) (40) | Diabetes | Quality of life | Health-related quality of life | RCT | CPA | Primary care | 248 | Mean (SD) | −2.95 (2.29) | −2.88 (2.07) | 0.46 |
| Hahn *et al.* (2018) (42) | Heart failure | Healthcare utilisation | 30-day all-cause readmission events | Retrospective cohort | CPA | Outpatient | 98 | No. events (%) | 3/35 (8.6%) | 9/35 (25.7%) | 0.05* |
| Hahn *et al.* (2018) (42) | Heart failure | Healthcare utilisation | 30-day heart failure readmission | Retrospective cohort | CPA | Outpatient | 98 | No. events (%) | 1/35 (2.8%) | 2/35 (8.0%) | 0.11 |
| Hahn *et al.* (2018) (42) | Heart failure | Healthcare utilisation | ED visits | Retrospective cohort | CPA | Outpatient | 98 | No. events (%) | 5/35 (14.3%) | 0/35 (0.0%) | NR |
| Rana *et al.* (2023) (43) | Heart failure | Treatment optimisation | Target ARNI dose achieved | Retrospective cohort | CPA | Outpatient | 791 | OR | 2.38 | NA | 0.0001*** |

| Study author (year) | Health population | Outcome domain | Outcome | Study design | Prescriptive authority | Setting | No. participants | Effect estimates | Intervention group | Comparator group | p-value |
|---|---|---|---|---|---|---|---|---|---|---|---|
| Rana *et al.* (2023) (43) | Heart failure | Healthcare utilisation | No. visits to achieve target ARNI dose | Retrospective cohort | CPA | Outpatient | 791 | Mean visits | 4.16 | 12.94 | 0.0001*** |
| Rana *et al.* (2023) (43) | Heart failure | Treatment optimisation | No. days to achieve ARNI dose | Retrospective cohort | CPA | Outpatient | 791 | Mean days | 279.32 | 333.66 | 0.091 |
| McAlister *et al.* (2014) (45) | Stroke | Blood pressure | Blood pressure and lipid level achieved | RCT | CPA | Primary care | 279 | Number of events (%) | 62/143 (43.4%) | 42/136 (30.9%) | NR |
| McAlister *et al.* (2014) (45) | Stroke | Blood pressure | Systolic blood pressure levels | RCT | CPA | Primary care | 279 | Mean (SD) | 126.5 (17.9) | 122.2 (13.0) | NR |
| McAlister *et al.* (2014) (45) | Stroke | Lipids | LDL cholesterol levels | RCT | CPA | Primary care | 279 | Mean (SD) | 2.21 (0.73) | 2.35 (0.81) | NR |
| McAlister *et al.* (2014) (45) | Stroke | Lipids | Change in HDL cholesterol levels | RCT | CPA | Primary care | 279 | Mean change (SD) | −0.01 (0.23) | −0.04 (0.19) | NR |
| McAlister *et al.* (2014) (45) | Stroke | Adherence | Adherence | RCT | CPA | Primary care | 279 | No. events (%) | 138/143 (96.5%) | 132/136 (97.1%) | NR |
| McAlister *et al.* (2014) (45) | Stroke | Quality of life | Self-rated health | RCT | CPA | Primary care | 279 | Mean (SD) | 3.5 (0.9) | 3.4 (0.8) | NR |
| McAlister *et al.* (2014) (45) | Stroke | Quality of life | Health-related quality of life | RCT | CPA | Primary care | 279 | Mean (SD) | 0.84 (0.15) | 0.86 (0.17) | NR |

| Study author (year) | Health population | Outcome domain | Outcome | Study design | Prescriptive authority | Setting | No. participants | Effect estimates | Intervention group | Comparator group | p-value |
|---|---|---|---|---|---|---|---|---|---|---|---|
| Villeneuve *et al.* (2010) (46) | Dyslipidaemia | Lipids | Lipid levels target reached | Cluster RCT | CPA | Primary care | 225 | Relative risk (95% CI) | 1.16 (1.01 to 1.34) | Ref | Significant[a] |
| Villeneuve *et al.* (2010) (46) | Dyslipidaemia | Lipids | LDL cholesterol levels | Cluster RCT | CPA | Primary care | 225 | Mean difference (SD) | −0.05 (−0.3 to 0.2) | Ref | Not significant[a] |
| Villeneuve *et al.* (2010) (46) | Dyslipidaemia | Lipids | HDL cholesterol | Cluster RCT | CPA | Primary care | 225 | Mean difference (95% CI) | 0.02 (−0.03 to 0.07) | Ref | Not significant[a] |
| Villeneuve *et al.* (2010) (46) | Dyslipidaemia | Lipids | Triglyceride levels | Cluster RCT | CPA | Primary care | 225 | Mean difference (95% CI) | −0.03 (−0.2 to 0.1) | Ref | Not significant[a] |
| Villeneuve *et al.* (2010) (46) | Dyslipidaemia | Blood pressure | Systolic blood pressure levels | Cluster RCT | CPA | Primary care | 225 | Mean difference (95% CI) | −1.3 (−6.4 to 3.8) | Ref | Not significant[a] |
| Villeneuve *et al.* (2010) (46) | Dyslipidaemia | Blood pressure | Diastolic blood pressure levels | Cluster RCT | CPA | Primary care | 225 | Mean difference (95% CI) | −1.8 (−5.0 to 1.4) | Ref | Not significant[a] |
| Villeneuve *et al.* (2010) (46) | Dyslipidaemia | Blood sugar | Fasting blood glucose levels | Cluster RCT | CPA | Primary care | 225 | Mean difference (95% CI) | −0.1 (−0.6 to 0.4) | Ref | Not significant[a] |
| Villeneuve *et al.* (2010) (46) | Dyslipidaemia | Healthcare utilisation | Healthcare utilisation | Cluster RCT | CPA | Primary care | 225 | Relative risk (95% CI) | −0.45 (−1.48 to 0.58) | Ref | Not significant[a] |
| Villeneuve *et al.* (2010) (46) | Dyslipidaemia | Adherence | Adherence | Cluster RCT | CPA | Primary care | 225 | No. events (%) | 78/108 (72.2%) | 79/117 (67.5%) | NR |

| Study author (year) | Health population | Outcome domain | Outcome | Study design | Prescriptive authority | Setting | No. participants | Effect estimates | Intervention group | Comparator group | p-value |
|---|---|---|---|---|---|---|---|---|---|---|---|
| O'Neill *et al.* (2014) (47) | Hypertension | Blood pressure | Blood pressure goal achieved | Retrospectiv e cohort | CPA | Outpatient | 126 | Odds ratio | 0.95 (0.40 to 2.26) | Ref | 0.24 |
| O'Neill *et al.* (2014) (47) | Hypertension | Blood pressure | Change in systolic blood pressure levels | Retrospectiv e cohort | CPA | Outpatient | 126 | Mean change (SD) | 14 (13) | 10 (11) | 0.42 |
| O'Neill *et al.* (2014) (47) | Hypertension | Blood pressure | Change in diastolic blood pressure levels | Retrospectiv e cohort | CPA | Outpatient | 126 | Mean difference (SD) | 6 (10) | 6 (7) | 0.93 |
| Vivian (2002) (48) | Hypertension | Blood pressure | Blood pressure goal achieved | RCT | Protocol | Outpatient | 56 | No. events (%) | 21/26 (81%) | 8/27 (30%) | 0.001*** |
| Vivian (2002) (48) | Hypertension | Blood pressure | Systolic blood pressure level | RCT | Protocol | Outpatient | 56 | Mean (SD) | 130.5 (13.2) | 148.4 (21.0) | 0.0002*** |
| Vivian (2002) (48) | Hypertension | Blood pressure | Change in systolic blood pressure levels | RCT | Protocol | Outpatient | 56 | Mean change (95% CI) | −18.4 (−26.3 to 10.5) | −3.98 (−11.8 to 3.79 | 0.01* |
| Vivian (2002) (48) | Hypertension | Blood pressure | Diastolic blood pressure levels | RCT | Protocol | Outpatient | 56 | Mean (SD) | 77.5 (10.7) | 80.4 (11.4) | 0.259 |

| Study author (year) | Health population | Outcome domain | Outcome | Study design | Prescriptive authority | Setting | No. participants | Effect estimates | Intervention group | Comparator group | p-value |
|---|---|---|---|---|---|---|---|---|---|---|---|
| Vivian (2002) (48) | Hypertension | Blood pressure | Change in diastolic blood pressure levels | RCT | Protocol | Outpatient | 56 | Mean change (95% CI) | −12.38 (−16.49 to −8.28) | 2.54 (−1.49 to 6.57) | 0.001* |
| Vivian (2002) (48) | Hypertension | Adherence | Adherence | RCT | Protocol | Outpatient | 56 | NR | NR | NR | >0.25 |
| Vivian (2002) (48) | Hypertension | Quality of life | Health related quality of life | RCT | Protocol | Outpatient | 56 | NR | NR | NR | >0.2 |
| Boddy (2001) (51) | Coagulation disorders | Therapeutic control | INR control achieved | Non-randomised trial | Protocol | Inpatient | 138 | No. events (%) | 43/74 (58.1%) | 12/64 (18.8%) | <0.001** |
| Hall *et al.* (2011) (52) | Coagulation disorders | Therapeutic control | INR control achieved | Retrospective cohort | CPA | Outpatient | 350 | No. events (%) | 118/175 (67.4%) | -54.90% | <0.0001*** |
| Manzoor *et al.* (2018) (54) | Coagulation disorders | Therapeutic control | INR control achieved | Retrospective cohort | CPA | Outpatient | 200 | Mean difference | −8.41 | Ref | 0.07 |
| Hall *et al.* (2011) (52) | Coagulation disorders | Therapeutic control | Percentage time INR was in therapeutic range | Retrospective cohort | CPA | Outpatient | 350 | Percentage of time | 73.70% | 61.10% | <0.0001*** |
| Damaske and Baird (2005) (50) | Coagulation disorders | Therapeutic control | Average time to therapeutic INR | Retrospective cohort | Protocol | Inpatient | 51 | Mean number of days (range) | 5.6 (4 to 11) | 6.0 (4 to 11) | NR |
| Boddy (2001) (51) | Coagulation disorders | Therapeutic control | INR control achieved | Non-randomised trial | Protocol | Inpatient | 138 | No. events (%) | 43/74 (58.1%) | 12/64 (18.8%) | <0.001** |

| Study author (year) | Health population | Outcome domain | Outcome | Study design | Prescriptive authority | Setting | No. participants | Effect estimates | Intervention group | Comparator group | p-value |
|---|---|---|---|---|---|---|---|---|---|---|---|
| Cohen *et al.* (1985) (49) | Coagulation disorders | Treatment optimisati on | Prothrombin time ratio in the therapeutic range | Retrospectiv e cohort | Protocol | Outpatient | 95 | Proportion of participants (%) | 68/78 (87.0%) | 14/17 (82.4%) | >0.05 |
| Chenella *et al.* (1983) (53) | Coagulation disorders | Treatment optimisati on | Average time to achieve therapeutic proconvertin and prothrombin levels | RCT | Independen t | Inpatient | 81 | Mean number of days (SD) | 5.7 (1.4) | 5.8 (1.4) | >0.05 |
| Chenella *et al.* (1983) (53) | Coagulation disorders | Treatment optimisati on | Partial thromboplastin time | RCT | Independen t | Inpatient | 81 | Mean number of seconds (SD) | 82.0 (14) | 84.0 (17) | >0.05 |
| Aspinall (2012) (55) | Chronic kidney disease | Haemoglo bin | Haemoglobin goal achieved | Retrospectiv e cohort | CPA | Outpatient | 572 | No. events (%) | 1,284/1,807 (71.1%) | 179/346 (51.7%) | 0.001*** |
| Beahm *et al.* (2018) (56) | Urinary tract infection | Clinical cure | Clinical cure at two weeks | Non-randomised trial | Independen t | Communit y pharmacy | 750 | No. events (%) | 528/596 (88.6%) | 82/90 (91.1%) | 0.99 |
| Beahm *et al.* (2018) (56) | Urinary tract infection | Healthcare utilisation | Time to access care | Non-randomised trial | Independen t | Communit y pharmacy | 750 | Mean number of days (SD) | 1.7 (2.4) | 2.8 (3.8) | 0.02* |
| Beahm *et al.* (2018) (56) | Urinary tract infection | Adherence | Adherence | Non-randomised trial | Independen t | Communit y pharmacy | 750 | No. events (%) | 575/596 (96.5%) | 81/90 (90.0%) | 0.0008*** |

| Study author (year) | Health population | Outcome domain | Outcome | Study design | Prescriptive authority | Setting | No. participants | Effect estimates | Intervention group | Comparator group | p-value |
|---|---|---|---|---|---|---|---|---|---|---|---|
| Holland *et al.* (2023) (57) | Older people in long-term care | Falls | Falls | Cluster RCT | Independen t | Long-term care | 882 | No. events (%) | 1.26/449 (0.3%) | 1.55/427 (0.4%) | 0.58 |
| Lau *et al.* (2022) (58) | Older people in long-term care | Falls | Falls | RCT | Protocol | Long-term care | 120 | No. events (%) | 9/47 (19.1%) | 9/45 (20.0%) | NR |
| Varghese *et al.* (2024) (44) | Older people in long-term care | Treatment optimisati on | Rates of aspirin deprescribing | Prospective cohort | Protocol | Primary care | 122 | No events (%) | 35/65 (53.8%) | 10/57 (17.5%) | 0.0001*** |
| Thompson *et al.* (1984) (59) | Older people in long-term care | Treatment optimisati on | Drug burden | Non-randomised trial | Formulary | Long-term care | 152 | Mean (SD) | 5.7 (3.29) | 7.1 (13.65) | 0.04* |
| Holland *et al.* (2023) (57) | Older people in long-term care | Treatment optimisati on | Drug burden | Cluster RCT | Independen t | Long-term care | 882 | Mean (SD) | 0.66 (0.74) | 0.73 (0.69) | 0.001** |
| Holland *et al.* (2023) (57) | Older people in long-term care | Quality of life | Health-related quality of life | Cluster RCT | Independen t | Long-term care | 882 | Mean (SD) | 0.26 (0.35) | 0.21 (0.33) | 0.04* |
| Lau *et al.* (2022) (58) | Older people in long-term care | Quality of life | Health-related quality of life | RCT | Protocol | Long-term care | 120 | Mean change (SD) | −0.0 (0.4) | 0.0 (0.1) | 0.57 |
| Lau *et al.* (2022) (58) | Older people in long-term care | Mental health | Depression Lau | RCT | Protocol | Long-term care | 120 | Mean change (SD | 1.0 (3.8) | 1.2 (3.1) | 0.92 |

| Study author (year) | Health population | Outcome domain | Outcome | Study design | Prescriptive authority | Setting | No. participants | Effect estimates | Intervention group | Comparator group | p-value |
|---|---|---|---|---|---|---|---|---|---|---|---|
| Lau *et al.* (2022) (58) | Older people in long-term care | Mental health | Anxiety | RCT | Protocol | Long-term care | 120 | Mean change (SD) | 0.4 (1.8) | 0.1 (1.4) | 0.43 |
| Lau *et al.* (2022) (58) | Older people in long-term care | Blood pressure | Systolic blood pressure | RCT | Protocol | Long-term care | 120 | No. events (%) | 31/47 (66%) | 25/45 (55.5%) | 0.15 |
| Holland *et al.* (2023) (57) | Older people in long-term care | Healthcare utilisation | Hospitalisation s | Cluster RCT | Independen t | Long-term care | 882 | Mean (SD) | 0.19 (0.5) | 0.18 (0.47) | 0.57 |
| Lau *et al.* (2022) (58) | Older people in long-term care | Healthcare utilisation | Hospitalisation s | RCT | Protocol | Long-term care | 120 | Mean change (SD) | 0.31 (0.70) | 0.24 (0.73) | 0.57 |
| Thompson *et al.* (1984) (59) | Older people in long-term care | Healthcare utilisation | Hospitalisation s | Non-randomised trial | Formulary | Long-term care | 152 | No. events (%) | 2/67 (3.0%) | 8/72 (11.1%) | 0.06 |
| Lau *et al.* (2022) (58) | Older people in long-term care | Healthcare utilisation | ED admissions | RCT | Protocol | Long-term care | 120 | Mean (SD) | 0.59 (1.19) | 0.39 (0.77) | 0.276 |
| Rodriguez *et al.* (2022) (61) | Female contraceptive users | Adherence | Medication adherence | Retrospectiv e cohort | Independen t prescribing | Communit y pharmacy | 172,325 | No. events (%) | 548 (61.6%) | 42,182 (61.9%) | 0.89 |

| Study author (year) | Health population | Outcome domain | Outcome | Study design | Prescriptive authority | Setting | No. participants | Effect estimates | Intervention group | Comparator group | p-value |
|---|---|---|---|---|---|---|---|---|---|---|---|
| Rodriguez *et al.* (2021) (60) | Female contraceptive users | Adherence | Medication continuation | Retrospective cohort | Independent prescribing | Community pharmacy | 172,325 | No. events (%) | 519 (34.3%) | 1,512 (21.0%) | <0.05 |
| Rodriguez *et al.* (2022) (61) | Female contraceptive users | Adherence | Medication continuation | Prospective cohort | Independent prescribing | Community pharmacy | 338 | Odds ratio (95% CI) | 0.70 (0.28 to 1.71) | Ref | NS |
| Rodriguez *et al.* (2021) (60) | Female contraceptive users | Adherence | Medication adherence | Prospective cohort | Independent prescribing | Community pharmacy | 338 | Odds ratio (95% CI) | 0.87 (0.51 to 1.48) | Ref | NS |
| Weaver *et al.* (2023) (62) | Anaemia in pregnancy | Haemoglobin | Haemoglobin goal achieved | Retrospective cohort | CPA | Outpatient | 100 | No. events (%) | 87/100 (87.0%) | versus 71/100 (71.0%) | <0.01** |
| Weaver *et al.* (2023) (62) | Anaemia in pregnancy | Haemoglobin | Haemoglobin levels | Retrospective cohort | CPA | Outpatient | 100 | Mean haemoglobin (SD) | 12.1 (1.0) | 11.6 (1.1) | <0.01** |
| Bruhn *et al.* (2013) (63) | Chronic pain conditions | Pain | Chronic pain intensity | RCT | Independent | Primary care | 196 | Mean (SD) | 58.1 (19.5) | 65.6 (19.6) | NR |
| Bruhn *et al.* (2013) (63) | Chronic pain conditions | Pain | Chronic pain disability | RCT | Independent | Primary care | 196 | Median (IQR) | 40.0 (20.0 to 60.1) | 50.0 (25.0 to 80.0) | NR |
| Bruhn *et al.* (2013) (63) | Chronic pain conditions | Quality of life | Health-related quality of life (physical) | RCT | Independent | Primary care | 196 | Mean (SD) | 35.3 (10.8) | 32.59 (9.14) | NR |

| Study author (year) | Health population | Outcome domain | Outcome | Study design | Prescriptive authority | Setting | No. participants | Effect estimates | Intervention group | Comparator group | p-value |
|---|---|---|---|---|---|---|---|---|---|---|---|
| Bruhn *et al.* (2013) (63) | Chronic pain conditions | Quality of life | Health-related quality of life (mental) | RCT | Independen t | Primary care | 196 | Median (IQR) | 49.6 (42.8 to 58.2) | 44.7 (37.6 to 55.8) | NR |
| Bruhn *et al.* (2013) (63) | Chronic pain conditions | Mental health | Depression | RCT | Independen t | Primary care | 196 | Median (IQR) | 4.0 (2.0 to 8.0) | 5.0 (2.0 to 10.0) | NR |
| Bruhn *et al.* (2013) (63) | Chronic pain conditions | Mental health | Anxiety | RCT | Independen t | Primary care | 196 | Median (IQR) | 5.0 (2.3 to 9.8) | 7.0 (4.0 to 10.0) | NR |
| Romanelli *et al.* (2015) (33) | Mixed health conditions | Healthcare utilisation | Ambulatory care visits | Retrospectiv e cohort | CPA | Primary care (medical home) | 1108 | Incidence rate ratio (95% CI) | 1.19 (1.06 to 1.33) | Ref (Arm 2) | 0.004** |
| Romanelli *et al.* (2015) (33) | Mixed health conditions | Healthcare utilisation | Ambulatory care visits | Retrospectiv e cohort | CPA | Primary care (medical home) | 1108 | Incidence rate ratio (95% CI) | 1.04 (0.92 to 1.18) | Ref (Arm 3) | 0.479 |
| Romanelli *et al.* (2015) (33) | Mixed health conditions | Healthcare utilisation | ED visits | Retrospectiv e cohort | CPA | Primary care (medical home) | 1108 | Incidence rate ratio (95% CI) | 0.79 (0.58 to 1.07) | Ref (Arm 2) | 0.124 |
| Romanelli *et al.* (2015) (33) | Mixed health conditions | Healthcare utilisation | ED visits | Retrospectiv e cohort | CPA | Primary care (medical home) | 1108 | Incidence rate ratio (95% CI) | 0.70 (0.53 to 0.93) | Ref (Arm 3) | 0.014** |

| Study author (year) | Health population | Outcome domain | Outcome | Study design | Prescriptive authority | Setting | No. participants | Effect estimates | Intervention group | Comparator group | p-value |
|---|---|---|---|---|---|---|---|---|---|---|---|
| Romanelli *et al.* (2015) (33) | Mixed health conditions | Healthcare utilisation | Hospitalisations | Retrospective cohort | CPA | Primary care (medical home) | 1108 | Incidence rate ratio (95% CI) | 0.48 (0.30 to 0.78) | Ref (Arm 2) | 0.003** |
| Romanelli *et al.* (2015) (33) | Mixed health conditions | Healthcare utilisation | Hospitalisations | Retrospective cohort | CPA | Primary care (medical home) | 1108 | Incidence rate ratio (95% CI) | 0.40 (0.25 to 0.63) | Ref (Arm 3) | 0.001** |
| Scullin *et al.* (2007) (64) | Mixed health conditions | Healthcare utilisation | Length of hospital stay | RCT | Protocol | Inpatient | 762 | Mean (95% CI) | 7.8 (7.1 to 8.6) | 9.8 (8.8 to 10.9) | 0.003** |
| Scullin *et al.* (2007) (64) | Mixed health conditions | Healthcare utilisation | Hospital readmission | RCT | Protocol | Inpatient | 762 | Number of events (%) | 141/370 (38%) | 172/383 (45%) | 0.027* |
| Romanelli *et al.* (2015) (33) | Mixed health conditions | Blood pressure | Blood pressure goal achieved | Retrospective cohort | CPA | Primary care (medical home) | 1108 | Odds ratio (95% CI) | 1.16 (0.79 to 1.71) | Ref (Arm 2) | 0.454 |
| Romanelli *et al.* (2015) (33) | Mixed health conditions | Blood pressure | Blood pressure goal achieved | Retrospective cohort | CPA | Primary care (medical home) | 1108 | Odds ratio (95% CI) | 1.47 (1.00 to 2.16) | Ref (Arm 3) | 0.05* |
| Romanelli *et al.* (2015) (33) | Mixed health conditions | Lipids | LDL cholesterol goal achieved | Retrospective cohort | CPA | Primary care (medical home) | 1108 | Odds ratio (95% CI) | 1.32 (0.70 to 2.46) | Ref (Arm 2) | 0.39 |
| Romanelli *et al.* (2015) (33) | Mixed health conditions | Lipids | LDL cholesterol goal achieved | Retrospective cohort | CPA | Primary care (medical home) | 1108 | Odds ratio (95% CI) | 0.83 (0.45 to 1.54) | Ref (Arm 3) | 0.565 |

| Study author (year) | Health population | Outcome domain | Outcome | Study design | Prescriptive authority | Setting | No. participants | Effect estimates | Intervention group | Comparator group | p-value |
|---|---|---|---|---|---|---|---|---|---|---|---|
| Romanelli *et al.* (2015) (33) | Mixed health conditions | Blood sugar | HbA1c goal achieved | Retrospective cohort | CPA | Primary care (medical home) | 1108 | Odds ratio (95% CI) | 0.61 (0.25 to 1.49) | Ref (Arm 2) | 0.278 |
| Romanelli *et al.* (2015) (33) | Mixed health conditions | Blood sugar | HbA1c goal achieved | Retrospective cohort | CPA | Primary care (medical home) | 1108 | Odds ratio (95% CI) | 0.44 (0.18 to 1.08) | Ref (Arm 3) | 0.074 |

*<0.05; **<0.01; ***<0.001; [a]author assessment of significance based on confidence interval reported

Acronyms: angiotensin receptor-neprilysin inhibitor (ARNI), confidence interval (CI), collaborative practice agreement (CPA), glycated haemoglobin (HbA1C), high-density lipoprotein (HDL), low-density lipoprotein (LDL), not reported (NR), no. (number of) randomised controlled trial (RCT), standard deviation (SD), standard error (SE)

# Appendix O Table of safety findings

| Study author (year) | Health population | Outcome domain | Outcome | Study design | Prescriptive authority | Setting | No. participants | Effect estimates | Intervention group | Comparator group | p-value |
|---|---|---|---|---|---|---|---|---|---|---|---|
| Rana *et al.* (2023) (43) | Heart failure | Hospitalisations/ ED visits due to adverse events | Hospitalisations due to heart failure | Retrospective cohort | CPA | Outpatient | 791 | No. events (%) | 33/64 (51.6%) | 398/727 (54.7%) | 0.73 |
| Rana *et al.* (2023) (43) | Heart failure | Mortality | Mortality | Retrospective cohort | CPA | Outpatient | 791 | No. events (%) | 0/64 (0%) | 47/727 (6.5%) | 0.04* |
| McAlister *et al.* (2014) (45) | Stroke | Mortality | Mortality | RCT | CPA | Primary care | 279 | No. events (%) | 0/143 (0.0%) | 1/136 (0.7%) | NR |
| McAlister *et al.* (2014) (45) | Stroke | Adverse events | Adverse vascular events | RCT | CPA | Primary care | 279 | No. events (%) | 9/143 (6.3%) | 8/136 (5.9%) | NR |
| Villeneuve *et al.* (2010) (46) | Dyslipidaemia | Adverse events | Adverse vascular events | Cluster RCT | CPA | Primary care | 225 | No. events (%) | 8/101 (7.9%) | 8/110 (7.3%) | NR |
| Cohen *et al.* (1985) (49) | Coagulation disorders | Adverse events | Bleeding adverse events | Retrospective cohort | Protocol | Outpatient | 95 | No. events (%) | 4/78 (5.1%) | 1/17 (5.9%) | >0.05 |
| Cohen *et al.* (1985) (49) | Coagulation disorders | Adverse events | Thromboembolic adverse events | Retrospective cohort | Protocol | Outpatient | 95 | No. events (%) | 2/78 (2.6%) | 0/17 (0.0%) | >0.05 |

| Study author (year) | Health population | Outcome domain | Outcome | Study design | Prescriptive authority | Setting | No. participants | Effect estimates | Intervention group | Comparator group | p-value |
|---|---|---|---|---|---|---|---|---|---|---|---|
| Hall *et al.* (2011) (52) | Coagulation disorders | Adverse events | Anticoagulation-related adverse events | Retrospective cohort | CPA | Outpatient | 350 | No. events (%) | 14/175 (8.0%) | 41/175 (23.4%) | <0.0001*** |
| Damaske and Baird (2005) (50) | Coagulation disorders | Adverse events | Bleeding adverse events /adverse drug events | Non-randomised trial | Protocol | Inpatient | 51 | No. events (%) | 2/22 (9.1%) | 3/29 (10.3%) | NR |
| Hall *et al.* (2011) (52) | Coagulation disorders | Hospitalisations/ED visits due to adverse events | Hospitalisation | Retrospective cohort | CPA | Outpatient | 350 | No. events (%) | 58/175 (33.1%) | 134/175 (76.6%) | <0.0001*** |
| Hall *et al.* (2011) (52) | Coagulation disorders | Hospitalisations/ED visits due to adverse events | ED visits | Retrospective cohort | CPA | Outpatient | 350 | No. events (%) | 3/175 (1.7%) | 14/175 (8.0%) | <0.0001*** |
| Manzoor *et al.* (2018) (54) | Coagulation disorders | Hospitalisations/ ED visits due to adverse events | Warfarin-related hospitalisations/ ED visits | Retrospective cohort | CPA | Outpatient | 200 | Odds ratio (95% CI) | Ref | 7.68 (1.06 to 55.94) | <0.05* |
| Aspinall (2012) (55) | Chronic kidney disease | Adverse events | Thromboembolic adverse events | Retrospective cohort | CPA | Outpatient | 572 | No. events (rate per 180 days) | 6 (0.02) | Arm 2<br>3 (0.04) | NR |
| | | Adverse events | | Retrospective cohort | CPA | Outpatient | 572 | No. events | 6 (0.02) | Arm 3 | NR |

| Study author (year) | Health population | Outcome domain | Outcome | Study design | Prescriptive authority | Setting | No. participants | Effect estimates | Intervention group | Comparator group | p-value |
|---|---|---|---|---|---|---|---|---|---|---|---|
| Aspinall (2012) (55) | Chronic kidney disease | | Thromboembolic adverse events | | | | | (rate per 180 days) | | 7 (0.05) | |
| Aspinall (2012) (55) | Chronic kidney disease | Adverse events | Heart failure adverse events | Retrospective cohort | CPA | Outpatient | 572 | No. events (rate per 180 days) | 18 (0.06) | Arm 2<br>7 (0.10) | NR |
| Aspinall (2012) (55) | Chronic kidney disease | Adverse events | Heart failure adverse events | Retrospective cohort | CPA | Outpatient | 572 | No. events (rate per 180 days) | 18 (0.06) | Arm 3<br>9 (0.06) | NR |
| Aspinall (2012) (55) | Chronic kidney disease | Adverse events | Uncontrolled hypertension adverse events | Retrospective cohort | CPA | Outpatient | 572 | No. events (rate per 180 days) | 185 (0.66) | Arm 2<br>50 (0.69) | NR |
| Aspinall (2012) (55) | Chronic kidney disease | Adverse events | Uncontrolled hypertension adverse events | Retrospective cohort | CPA | Outpatient | 572 | No. events (rate per 180 days) | 185 (0.66) | Arm 3<br>73 (0.48) | NR |
| McFarland *et al.* (2009) (65) | Chronic kidney disease | Prescribing quality | Prescribing errors | Retrospective cohort | CPA | Outpatient | 290 | No. events (%) | 1/158 (0.6%) | 34/132 (25.8%) | NR |
| Beahm *et al.* (2018) (56) | Urinary tract infection | Adverse events | Total adverse events | Non-randomised trial | Independent | Community pharmacy | 750 | No. events (%) | 44/596 (7.4%) | 10/90 (11.1%) | NR |

| Study author (year) | Health population | Outcome domain | Outcome | Study design | Prescriptive authority | Setting | No. participants | Effect estimates | Intervention group | Comparator group | p-value |
|---|---|---|---|---|---|---|---|---|---|---|---|
| Beahm *et al.* (2018) (56) | Urinary tract infection | Adverse events | Gastrointestinal adverse events | Non-randomised trial | Independent | Community pharmacy | 750 | No. events (%) | 27/596 (4.5%) | 5/90 (5.5%) | 0.79 |
| Beahm *et al.* (2018) (56) | Urinary tract infection | Adverse events | Vaginal candidiasis adverse events | Non-randomised trial | Independent | Community pharmacy | 750 | No. events (%) | 5/596 (0.8%) | 3/90 (3.3%) | 0.11 |
| Beahm *et al.* (2018) (56) | Urinary tract infection | Adverse events | Headache adverse events | Non-randomised trial | Independent | Community pharmacy | 750 | No. events (%) | 90/596 (15.1%) | 0/90 (0.0%) | 0.7551 |
| Beahm *et al.* (2018) (56) | Urinary tract infection | Adverse events | Other adverse events | Non-randomised trial | Independent | Community pharmacy | 750 | No. events (%) | 6/596 (1.0%) | 2/90 (2.2%) | 0.5963 |
| Beahm *et al.* (2018) (56) | Urinary tract infection | Hospitalisations/ ED visits due to adverse events | Physician or ED visits | Non-randomised trial | Independent | Community pharmacy | 750 | No. events (%) | 3/596 (0.005%) | 2/90 (0.2%) | 0.2273 |
| Beahm *et al.* (2021) (66) | Urinary tract infection | Prescribing quality | Antimicrobial therapy guideline concordance | Non-randomised trial | Independent | Community pharmacy | 750 | No. events (%) | 624/656 (95.1%) | 33/94 (35.1%) | <0.001** |
| Holland *et al.* (2023) (57) | Older people in long-term care | Mortality | Mortality | Cluster RCT | Independent | Long-term care | 882 | No. events (%) | 66/449 (14.7%) | 71/427 (16.6%) | 0.68 |

| Study author (year) | Health population | Outcome domain | Outcome | Study design | Prescriptive authority | Setting | No. participants | Effect estimates | Intervention group | Comparator group | p-value |
|---|---|---|---|---|---|---|---|---|---|---|---|
| Thompson *et al.* (1984) (59) | Older people in long-term care | Mortality | Mortality | Non-randomised trial | Formulary | Long-term care | 152 | No. events (%) | 3/67 (4.5%) | 10/72 (13.9%) | 0.05* |
| Lau *et al.* (2022) (58) | Older people in long-term care | Adverse events | Syncope adverse events | RCT | Protocol | Long-term care | 120 | No. events (%) | 11/47 (23.4%) | 9/45 (20.0%) | 0.683 |
| Lau *et al.* (2022) (58) | Older people in long-term care | Adverse events | Hypotension adverse events | RCT | Protocol | Long-term care | 120 | No. events (%) | 13/47 (27.7%) | 3/45 (6.7%) | 0.009** |
| Lau *et al.* (2022) (58) | Older people in long-term care | Adverse events | Hypokalaemia adverse events | RCT | Protocol | Long-term care | 120 | No. events (%) | 3/47 (6.4%) | 5/45 (11.1%) | 0.435 |
| Lau *et al.* (2022) (58) | Older people in long-term care | Adverse events | Hyperkalaemia adverse events | RCT | Protocol | Long-term care | 120 | No. events (%) | 6/47 (12.8%) | 4/45 (8.9%) | 0.545 |
| Lau *et al.* (2022) (58) | Older people in long-term care | Adverse events | Hyponatraemia adverse events | RCT | Protocol | Long-term care | 120 | No. events (%) | 1/47 (2.1%) | 2/45 (4.4%) | 0.539 |
| Lau *et al.* (2022) (58) | Older people in long-term care | Adverse events | Orthostatic presyncope adverse events | RCT | Protocol | Long-term care | 120 | No. events (%) | 7/47 (14.9%) | 5/45 (11.1%) | 0.59 |

| Study author (year) | Health population | Outcome domain | Outcome | Study design | Prescriptive authority | Setting | No. participants | Effect estimates | Intervention group | Comparator group | p-value |
|---|---|---|---|---|---|---|---|---|---|---|---|
| Lau *et al.* (2022) (58) | Older people in long-term care | Adverse events | Estimated glomerular filtration rate to monitor kidney function | RCT | Protocol | Long-term care | 120 | Mean change (SD) | −2.68 (8.75) | −1.05 (9.14) | 0.381 |
| Rodriguez *et al.* (2020) (68) | Female contraceptive users | Prescribing quality | Medical contraindications | Prospective cohort | Independent prescribing | Community pharmacy | 448 | No. events (%) | 14/20 (70%) | 32/40 (80%) | 0.52 |
| Rodriguez *et al.* (2021) (69) | Female contraceptive users | Prescribing quality | Medical contraindications | Retrospective cohort | Independent prescribing | Community pharmacy | 439,240 | No. events (%) | 28/3,782 (0.8%) | 9,392/18,490 (2.2%) | NR |
| Ogilvie *et al.* (2022) (70) | Emergency department users | Prescribing quality<br>Prescribing quality | Prescribing errors | RCT | CPA | Emergency department | 94 | No. events (%) | 68/412 (16.5%) | 279/357 (78.2%) | 0.001*** |
| Marotti *et al.* (2011) (71) | Surgery patients | Prescribing quality | Medications charted at incorrect frequency | RCT | Supplementary | Inpatient surgery | 332 | Mean (95% CI) | 0.015 (0.00 to 0.07) | 0.29 (0.19 to 0.39) | <0.01** |
| Marotti *et al.* (2011) (71) | Surgery patients | Prescribing quality | Medications charted at incorrect dose | RCT | Supplementary | Inpatient surgery | 332 | Mean (95% CI) | 0.02 (0.00 to 0.05) | 0.48 (0.35 to 0.18) | <0.01** |

| Study author (year) | Health population | Outcome domain | Outcome | Study design | Prescriptive authority | Setting | No. participants | Effect estimates | Intervention group | Comparator group | p-value |
|---|---|---|---|---|---|---|---|---|---|---|---|
| Marotti *et al.* (2011) (71) | Surgery patients | Prescribing quality | Doses missed during inpatient stay | RCT | Supplementary | Inpatient surgery | 332 | Mean (95% CI) | 1.07 (0.9 to 1.26) | 3.21 (2.89 to 3.5) | <0.001*** |
| Scullin *et al.* (2007) (64) | People at risk of drug-related problems | Mortality | Mortality | RCT | Protocol | Inpatient | 762 | No. events (%) | 67/371 (18.1%) | 76/391 (19.4%) | 0.578 |
| Rashid *et al.* (2022) (67) | Mixed health conditions | Hospitalisations/ ED visits due to adverse events | Acute kidney injury events (hospitalisations and ED visits | Retrospective cohort | CPA | Outpatient | 2155 | Odds ratio (95% CI) | 0.53 (0.24 to 1.16) | Ref | 0.11 |
| Rashid *et al.* (2022) (67) | Mixed health conditions | Hospitalisations/ ED visits due to adverse events | Gastrointestinal bleeding events (hospitalisations and ED visits) | Retrospective cohort | CPA | Outpatient | 2155 | Odds ratio (95% CI) | 0.65 (0.36 to 1.16) | Ref | 0.15 |

| Study author (year) | Health population | Outcome domain | Outcome | Study design | Prescriptive authority | Setting | No. participants | Effect estimates | Intervention group | Comparator group | p-value |
|---|---|---|---|---|---|---|---|---|---|---|---|
| Rashid *et al.* (2022) (67) | Mixed health conditions | Hospitalisations/ ED visits due to adverse events | Pain (hospitalisations and ED visits) | Retrospective cohort | CPA | Outpatient | 2155 | Odds ratio (95% CI) | 0.50 (0.33 to 0.77) | Ref | <0.01** |

*<0.05; **<0.01; ***<0.001

Acronyms: confidence interval (CI), collaborative practice agreement (CPA), emergency department (ED), not reported (NR), randomised controlled trial (RCT), reference case (ref), standard deviation (SD)

# Appendix P Reported outcome significance across models of prescriptive authority

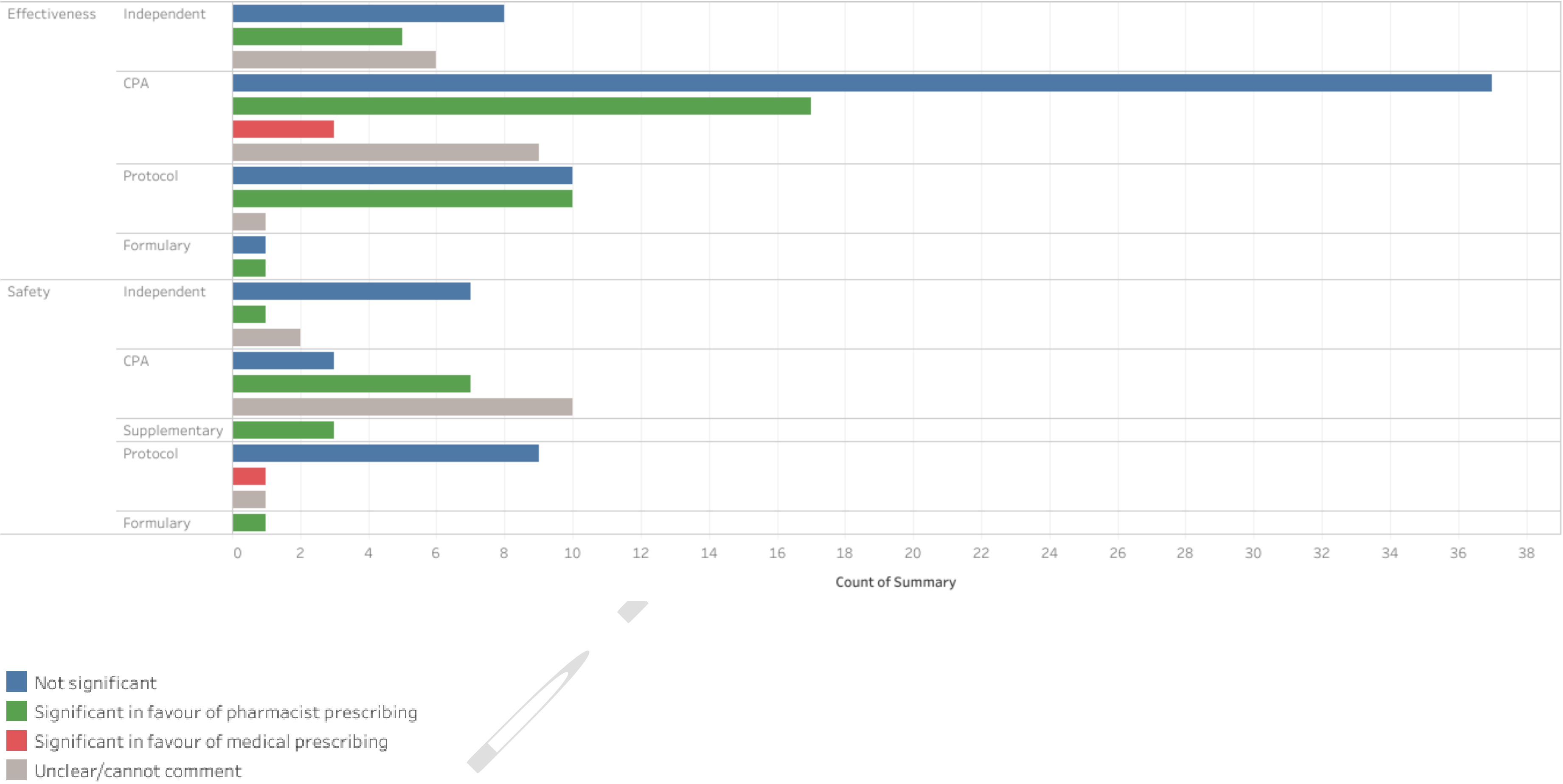


Acronyms: collaborative practice agreement (CPA)

# Appendix Q Number of outcomes reported by outcome domain

| Outcome type | Outcome domain | |
|---|---|---|
| Effectiveness | Blood pressure | 20 |
| | Healthcare utilisation | 18 |
| | Blood sugar | 14 |
| | Lipids | 13 |
| | Treatment optimisation | 9 |
| | Quality of life | 8 |
| | Adherence | 8 |
| | Therapeutic control | 6 |
| | Mental health | 4 |
| | Haemoglobin | 3 |
| | Pain | 2 |
| | Falls | 2 |
| | Clinical cure | 1 |
| Safety | Adverse events | 24 |
| | Prescribing quality | 8 |
| | Hospitalisations/ED visits due to adverse events | 8 |
| | Mortality | 5 |

Acronyms: emergency department (ED)
