# Supplementary material for: Effectiveness and safety of pharmacist prescribing: a systematic review
Source: BMJ Open. 2026 Jun 25;16(6):e112886. doi: 10.1136/bmjopen-2025-112886 (PMC13311757; doi:10.1136/bmjopen-2025-112886)
Supplement: Supplementary file 2 [file bmjopen-2025-112886.reviewer_comments.pdf]

PEER REVIEW HISTORY

BMJ Open publishes all reviews undertaken for accepted manuscripts. Reviewers are asked to complete a checklist review form and are provided with free text boxes to elaborate on their assessment. These free text comments are reproduced below.

ARTICLE DETAILS

Title (Provisional)

The effectiveness and safety of pharmacist prescribing: A systematic review

Authors

Teahan, Áine; Sharp, Melissa; Farragher, Ailish; Strawbridge, Judith; Long, Jean

---

VERSION 1 - REVIEW

**Reviewer**	**1**
**Name**	**Bond, Christine**
**Affiliation**	**University of Aberdeen**
**Date**	**21-Nov-2025**
**COI manuscript**	**I am an author of several of the studies reported in this**

This is a very comprehensive review of a topical issue, especially relevant to countries like the UK where all pharmacists are graduating ready to prescribe once registered. The authors are to be congratulated on a very large piece of detailed data extraction and reporting.

However the very comprehensiveness is also a challenge for reporting. As noted below the details for each section are inconsistent. Further there is no critical synthesis of the findings. For example could the stronger studies be commented on compared to the others, or a comparison of the strengths of effects of different type of independent prescribing? Whilst I agree that despite all the short comings the trend supports IP I still think it would be useful for more consideration of the issues above. Some detailed points follow including a query about the accuracy of reporting of a study with which I was personally involved as PI.

In the below the page and line numbers refer to those on the pdf

Introduction

1. P 4, Lines 31-35 You write 'Dependent prescribing, where pharmacists are limited to prescribing according to protocols or formularies, is practiced in Singapore, Australia and Ireland (5), Australia is actively piloting expanded pharmacist prescribing in several states (6) while Ireland is considering broader reforms (7).'. I was just wondering whether supply of a

medicine under a PGD (Patient group directions: who can use them - GOV.UK) would count as dependent prescribing. This is widely used in the UK.

2. P 5 lines 42-7 Give initials of researchers who did the data extraction for consistency with earlier sections on screening . Might also be useful to add a blank form as Supplementary material

3. In Table 1 six types of pharmacist prescribing are listed but they are not defined in the earlier section in the Introduction which just lists three and these three categories do not map exactly against the six. This is explained by Figure 1 but something linking the Figure to the text would be good.

4. The PRISMA flow chart- could an extra box be added at the bottom explicitly adding the 14 papers from the additional searches to the 25 from the original search?

5. Please give the dates when the original search was undertaken and the second additional search shown in the PRISMA chart. Date of main search is implied in the detail in te Appendix but needs to be in narrative

6. Not sure if I have missed it but were other systematic reviews included and the individual studies extracted from them and reported?

7. P 7 Lines 22-33 Please give the citations for the relevant studies in each category

8. Figure 3 Please add N of studies in the Figure legend. I am also surprised that the overall risk of bias seems intuitively higher than the individual elements might suggest. This needs more explanation

9. Figure 4 Likewise add N to legend. In this Figure the overall risk of bias is intuitively more what would be expected from the individual criteria

10. What are the numbers in brackets in Table 2 in the Study design column, I don't think they can be citations as I am assuming the chronic pain one is Bruhn et al and the citation for that would be 54 not 30.

11. P 10 Linked to 9 above, the authors write-' One study reported improvement in chronic pain intensity, chronic pain disability, health-related quality of life, depression, and anxiety in pharmacist prescribing compared with medical prescribing (54). However, due to the analysis chosen by the authors, it is not possible to comment on the significance of findings' This is a study I was involved with and this is not true – see Table 2 of the paper which reports a p of 0.02. Tables also show HADS for depression and anxiety are compared and are not significant. I have not checked all the other outcomes in this paper, nor other papers.

12. When describing clinical outcomes please be consistent with providing study details of included studies,. Eg for hypertension, two studies mentioned (38,39) not mentioned yet in previous two paragraphs on stroke and dislipidaemia, study design is mentioned. It might also be helpful to say where the study conducted and sector eg for chronic pain would say a

randomised controlled pilot study in general practice in the UK . Although this is all in the tables would be good to have in narrative as well for ease of access .

13. P 9 Line 46-50 something missing in the sentence ’No significant difference was reported time to achieve therapeutic proconvertin and prothrombin levels (44), partial thromboplastin time (44), or prothrombin time ratio in the therapeutic range between pharmacist prescribing and medical prescribing groups (40). ‘

14. Similarly when reporting the mixed conditions studies different details are provided- the first example provides a narrative description of the three groups this is not consistently provided. even the type of prescribing is not reported consistently

15. Our included studies only investigate pharmacist prescribing in adults, no studies investigating paediatric populations met our inclusion criteria- I just wonder if paediatric had been included as a search term if this would have found studies in that population?

16. The Discussion should be more nuanced and recognise the limitations to the conclusions imposed by the weakness of the studies despite the overall trend being in favour of IP. Again as per above because there is no stratification of findings by type of prescribing intervention the implications for future policy and practice are unclear.

Reviewer **2**

Name **Keers, Richard**

Affiliation **University of Manchester, Centre for Pharmacoepidemiology and Drug Safety Research**

Date **19-Dec-2025**

COI **None**

This was an excellently written paper on an important topic in modern health care policy and systems. The authors should be commended for a high quality study that covers a diverse literature base and for writing a well structured manuscript. My comments on this paper are generally minor (although some are more significant) and they generally relate to the need to provide additional detail or justifications for the reader to fully understand and process the data.

Abstract

- please add the search date range

- please add n=x for each setting

- please add a sentence summarising country of origin for the data

Introduction

- line 7,12,13: these sentences imply non medical prescribers are better but is it not the purpose of the review to establish this? Perhaps subtle wording changes might help clarify this point.

- line 37 onward. As above, the way it is worded here currently, it feels like a review of the evidence isn't actually needed? Can the authors please review and clarify in the text the position of the paper in the context of existing literature

Methods

- the cost-effectiveness study should be referenced if finished or say it is underway (as per the discussion)

- line 24. Please clarify in the text if authors were contacted for additional information.

- when the authors say that a clear statement of pharmacists prescriptive authority was needed, can they explain a little more what they meant in the text to make this as transparent as possible. For example were definitions needed or did the authors interpret the type of prescribing based on any text given as long as there was enough to use for that purpose.

Results

- table 1. Please can the authors clarify in the table whether adverse drug events that were not serious were included, and if not why not. Please can they also do the same for preventable adverse drug events (ie not due to ADRs).

- line 53-56. Please add n=x for each source of bias listed, and consider adding top 3 sources for each design.

- there is much use of the word 'significant' referring to results. Whilst the tables 2-3 showcase the reason why (referring to statistical significance), this isn't immediately apparent and a statement in the methods referring to the use of this term to indicate statistical significance of at least $p<0.05$ would help the reader with interpretation

- page 7 line 32. '… was reported in the pharmacist prescribing group compared…'

- page 7 line48: '… significance of this finding…'

- page 7 line 56: please can the authors describe deprescribing of what exactly in the text

- page 8 line 8: '…goal…'

- tables 2/3. Please include study duration and year of publication in the tables. This will help to understand how many older studies there were

- related to the above point, the authors need to include the full database search date duration in the methods and justify why pre-2000 studies were included - they are unlikely to represent the modern health care era and could distort the results. This should also be acknowledged as a limitation of the study later in the discussion section.

- in the results can the authors please comment on the value of adding some text concerning comparisons between outcome data and type of prescribing model. It would be interesting to see whether this factor was associated with neutral or positive outcomes for pharmacists. However I understand this might not be practical given the smaller number of studies in certain model groups and so this isn't a major point.

- p6 line 15-20. Please can the authors include in the text a sentence describing how many studies compared pharmacist prescribing to medical prescribing and how many to nurse prescribing.

- p10 line 4 and 12. '… pharmacist prescribing group compared…'

- p10 line 30. The word 'similar' appears twice , please can the authors review

- p10 line 45-51. '… in pharmacist prescribing groups…' or similar wording change for completeness

- p11 line 39-42. Hypotension events were reported as increased and had no significant difference for the same study , can the authors please review

- please can the authors include a table summarising the main outcome measures used in the included studies, and report the top 3 categories for effectiveness and safety in the text.

Discussion

- the study limitations should be expanded to include a lack of independent data data extraction by the authors , and the use of evidence pre dating the modern health care era.

**Reviewer**	**3**
**Name**	**Benrimoj, Shalom**
**Affiliation**	**University of Granada Faculty of Pharmacy, Faculty of Pharmacy**
**Date**	**11-Jan-2026**
**COI**	**None**

Summary

The manuscript addresses an important and timely topic and demonstrates a substantial research effort. The compilation of relevant studies is valuable; however, the paper faces significant challenges in scope, clarity, and interpretation. Assessing safety and efficacy—or comparing these outcomes with medical prescribing—across diverse conditions, settings, and prescribing models is inherently complex. These conditions are not been fully explored by the authors .At present, the evidence base does not support definitive conclusions.

Strengths

- The topic is of high relevance to readers and stakeholders.
- The authors have undertaken a comprehensive literature search.
- The narrative review approach is appropriate given the limited quantity and quality of available studies.
- Data extraction is comprehensive

Major Limitations

1. **Scope and Definitions**
    - Indicators of "efficacy" and "safety," which are central to the review, are not defined in the Methods section.
    - The paper adopts a specific definition of pharmacist prescribing without justification, despite ongoing debate in the field.
2. **Presentation of Data**
    - Tables are excessively long and difficult to interpret (e.g., Table 2 spans six pages).
    - Outcomes are inconsistently reported (e.g., mean change vs. goal achieved).
    - Study design is not systematically organized.
    - Consider alternative formats such as data visualization tools (e.g., Tableau) to improve clarity.
3. **Interpretation**
    - The discussion section is superficial, largely reiterating results without meaningful analysis.
    - Factors influencing safety and efficacy (e.g., prescribing model, prescriptive authority, patient cohort, setting) are not adequately considered.
4. **Supporting Information**
    - Absence of a TIDieR checklist limits clarity on intervention details.
    - Readers must consult original papers to understand interventions fully.

Specific Comments

Introduction

- **Line 10:** Reference 1 pertains only to hospital prescribing. Amend or add evidence from other settings.
- **Line 21:** Clarify the claim regarding nonmedical prescribing in 2016 and provide supporting evidence.
- **Lines 22–34:** Justify the choice of definitions for pharmacist prescribing.
- **Lines 37–39:** Define cost-effectiveness and consider limiting the claim.
- **Lines 49–51:** Clarify differences between minor ailments and pharmacist prescribing.
- **Lines 55–58:** Discuss variability in prescriptive authority and its potential impact.

- **Line 59:** Define indicators for “effectiveness” and “safety” and report results accordingly.

Methods

- Methods are generally robust; however, include TIDieR checklist for intervention details.

Results

- Reorganize tables by defined indicators.
- Remove duplication and ensure consistent outcome reporting
- Present in a formant which is more concise yet interpretable .

Discussion

- Expand interpretation beyond summary of results.
- Address contextual factors influencing safety and efficacy.

Recommendation

Major revisions required. The manuscript should be reconsidered after substantial restructuring, clarification of definitions, improved data presentation, and deeper interpretation of findings.

VERSION 1 - AUTHOR RESPONSE

Reviewer 1

Reviewer 1	Comments	Author response
Overall comment	This is a very comprehensive review of a topical issue, especially relevant to countries like the UK where all pharmacists are graduating ready to prescribe once registered. The authors are to be congratulated on a very large piece of detailed data extraction and reporting.<br><br>However the very comprehensiveness is also a challenge for reporting. As noted below the details for each section are inconsistent. Further there is no critical synthesis of the findings. For example could the stronger studies be commented on compared to the others, or a comparison of the strengths of effects of different type of independent prescribing? Whilst I agree that despite all the short comings the trend supports IP I still think it would be useful for more consideration of the issues above. Some detailed points follow including a query about the accuracy of reporting of a study with which I was personally involved as PI.	Thank you for your review and comments. We have responded to each of your specific comments below.<br><br>We have included the following text in the discussion to provide a more critical assessment of our findings.<br><br>*‘A methodological limitation of this study was the lack of independent data extraction. Additionally three of the included studies are dated pre-2000, limiting their relevance to modern health systems [49,53,59].’*<br><br>*‘Our evidence base was characterised by several methodological limitations, including a predominance of observational designs and high risk of bias, particularly due to confounding in non-randomised studies. Consequently, the findings demonstrate a consistent positive trend but do not provide strong causal evidence.’*

		'Although studies generally specified the type of prescribing authority, heterogeneity in study design, clinical context, outcomes measured, and implementation models limited the feasibility of stratified synthesis by prescribing type. As a result, the implications for policy and practice should be considered in light of this variability. Future research using robust comparative designs within clearly defined prescribing models would strengthen the evidence base and support more precise policy conclusions.'
Introduction	1. P 4, Lines 31-35 You write 'Dependent prescribing, where pharmacists are limited to prescribing according to protocols or formularies, is practiced in Singapore, Australia and Ireland (5), Australia is actively piloting expanded pharmacist prescribing in several states (6) while Ireland is considering broader reforms (7).'. I was just wondering whether supply of a medicine under a PGD (Patient group directions: who can use them - GOV.UK) would count as dependent prescribing. This is widely used in the UK.	Thank you for your comment. We have amended the test to indicate dependent prescribing is also practiced in the UK and referenced the provided source.<br>*'Dependent prescribing, where pharmacists are limited to prescribing according to protocols or formularies, is practiced in Singapore, Australia, the UK, and Ireland [12,13]'*
	3. In Table 1 six types of pharmacist prescribing are listed but they are not defined in the earlier section in the Introduction which just lists three and these three categories do not map exactly against the six. This is explained by Figure 1 but something linking the Figure to the text would be good.	The following text and cross-reference to Figure 1 has been included in the manuscript.<br>*'This review specifically focuses on prescribing by pharmacists. Pharmacists' scope of prescriptive authority varies internationally and formal definitions vary. Some frameworks and policy documents classify pharmacist prescribing only independent or dependent (with dependent prescribing including collaborative approaches) [7,8], whereas others provide distinct classifications for independent, collaborative, and dependent pharmacist prescribing [9]. In this paper we have categorised pharmacist prescribing into three models: independent prescribing, collaborative prescribing, and dependent prescribing (Figure 1) to better reflect variation in prescriptive authority across studies.'*
Methods	2. P 5 lines 42-7 Give initials of researchers who did the data extraction for consistency with earlier sections on screening . Might also be useful to add a blank form as Supplementary material	Thank you, this information has been added to the manuscript.<br>*'Data extraction was performed by one reviewer (ÁT or MS) and checked by a second (ÁT or MS).'*<br>A full completed extraction form for each of the included studies has been included in Appendix D.
	5. Please give the dates when the original search was undertaken and the second additional search shown in the PRISMA chart. Date of main search is implied in the detail in the Appendix but needs to be in narrative	Thank you. The following text has been included in the methods section<br>*'The original database search were conducted between 10th and 22nd July 2024. The updated database searches were conducted on 29th January 2025'*

	6. Not sure if I have missed it but were other systematic reviews included and the individual studies extracted from them and reported?	Systematic reviews did not meet our inclusion criteria for this systematic review.<br><br>As part of our supplementary search, we did complete citation search of relevant systematic reviews for to identify any primary studies meeting our inclusion criteria. A list of systematic reviews searched is included in Appendix C.<br><br>Additional text has been added to the manuscript to clarify this stage of searching.<br><br>*'We also conducted citation searching of relevant systematic reviews in this field (Appendix C).'*<br><br>Your next comment on expanding the PRISMA diagram now shows that 8 articles were included from this stage of the systematic review citation chasing.
Findings	4. The PRISMA flow chart- could an extra box be added at the bottom explicitly adding the 14 papers from the additional searches to the 25 from the original search?	We have expanded details of the included 39 studies in the PRISMA diagram to specify the sources of each include.<br><br>*'Studies included in review (n=39):*<br><br>*Database records (n=25)*<br><br>*Citation chasing (n=6)*<br><br>*Systematic review citation chasing (n=8)' Figure 2*
	7. P 7 Lines 22-33 Please give the citations for the relevant studies in each category	References are now included for each category<br><br>*'Of the 32 studies assessing effectiveness, 3 were based in community pharmacies [56,60,61], 12 were based in outpatient clinics [36,37,39,42,43,47–49,52,54,55,62], 10 in primary care [33–35,38,40,41,44–46,63], 3 in long-term care [57–59], and 4 in inpatient settings [50,51,53,64]. The prescriptive authority varied: 18 studies assessed collaborative practice agreements [33–43,45–47,52,54,55,62], 7 assessed protocol prescribing [44,48–51,58,64], 1 assessed formulary prescribing [59], and 6 assessed independent prescribing [53,56,57,60,61,63].*<br><br>*Of the 20 studies assessing safety, 4 studies were based in community pharmacies [56,66,68,69], 7 in outpatient clinics [43,49,52,54,55,65,67], 2 in primary care [45,46], 3 in long-term care [57–59], 3 in inpatient settings [50,64,71], and 1 in an emergency department [70]. The prescriptive authority varied: 9 studies assessed collaborative practice agreements [43,45,50,52,54,55,65,67,70], 4 assessed protocol prescribing [49,50,58,64], 1 assessed formulary prescribing [59], 1 assessed supplementary prescribing [71], and 5 assessed independent prescribing [56,57,66,68,69].'*

	8. Figure 3 Please add N of studies in the Figure legend. I am also surprised that the overall risk of bias seems intuitively higher than the individual elements might suggest. This needs more explanation<br><br>9. Figure 4 Likewise add N to legend. In this Figure the overall risk of bias is intuitively more what would be expected from the individual criteria	Thank you for your comment. We have added the N of studies after each figure.<br><br>• In relation to the overall risk of bias being intuitively higher, as per Cochrane guidelines, if even one domain is high risk of bias, then the overall study is scored as having high risk of bias. The risk of bias tools employed are widely used and well validated by Cochrane.<br>• For transparency we have included the full RoB assessments for each outcome in Appendices K, J, and L.
	10. What are the numbers in brackets in Table 2 in the Study design column, I don't think they can be citations as I am assuming the chronic pain one is Bruhn et al and the citation for that would be 54 not 30.	Thank you for raising this. These were citations. A referencing software was used, when we created a separate document for Tables and Figures, the in-text citations reformatted. We have addressed this error.<br><br>On request of another reviewer, Table 2 and 3 are now Appendices N and O.
	11. P 10 Linked to 9 above, the authors write-' One study reported improvement in chronic pain intensity, chronic pain disability, health-related quality of life, depression, and anxiety in pharmacist prescribing compared with medical prescribing (54). However, due to the analysis chosen by the authors, it is not possible to comment on the significance of findings' This is a study I was involved with and this is not true – see Table 2 of the paper which reports a p of 0.02. Tables also show HADS for depression and anxiety are compared and are not significant. I have not checked all the other outcomes in this paper, nor other papers.	Thank you for raising this. We have rewritten this paragraph to better reflect the findings of the Bruhn *et al.* (2013) paper.<br><br>*'One study reported improvement in chronic pain intensity (p=0.02), chronic pain disability (p=0.55), health-related quality of life (p=0.04), depression (p=0.32), and anxiety (p=0.14) in a one-way analysis of variance between three trial arms (pharmacist prescribing vs. pharmacist medication review vs. medical prescribing) (54). The one-way analysis of variance does not identify which specific arm comparisons account for the observed difference. Subsequently, it is not possible to comment on the significance of the effect between either pharmacist prescribing and medical prescribing, or pharmacist prescribing and pharmacist medication review.'*<br><br>The one-way ANOVA provides an omnibus test of differences across the three trial arms and indicates that at least one group mean differs (arm 1 vs. arm 2 vs. arm 3; p = 0.02). However, without reporting pairwise comparisons (arm 1 vs arm 2, arm 1 vs arm 3), it is not possible to determine which specific arm or arms are driving the observed difference.<br><br>For example, if Arm 2 were primarily responsible for the overall effect, reporting p=0.02 as reflecting a difference between Arm 1 and Arm 3 would be incorrect and would over-interpret the omnibus test.
	12. When describing clinical outcomes please be consistent with providing study details of included studies,. Eg for hypertension, two studies mentioned (38,39) not mentioned yet in previous two paragraphs on stroke and dislipidaemia, study design is mentioned. It might also be helpful to say where the study conducted and sector eg for chronic pain would say a randomised controlled pilot study in general practice in the UK . Although this is all in the	Thanks for your comment.<br><br>We have included details of study design, and associated citations for each section.<br><br>In regards to country and sector, we would prefer to omit these from the narrative text as becomes confusing in sections describing multiple studies.

	tables would be good to have in narrative as well for ease of access .	We have included full extraction forms in the appendices, detailed information on country and sector are available in Appendices N and O (previously Tables 2 and 3).
	13. P 9 Line 46-50 something missing in the sentence 'No significant difference was reported time to achieve therapeutic proconvertin and prothrombin levels (44), partial thromboplastin time (44), or prothrombin time ratio in the therapeutic range between pharmacist prescribing and medical prescribing groups (40). '	Thank you, we have corrected this in the main manuscript<br>*'No significant difference was reported in time to achieve therapeutic proconvertin and prothrombin levels [53], partial thromboplastin time [53], or prothrombin time ratio in the therapeutic range between pharmacist prescribing and medical prescribing groups [49].'*
	14. Similarly when reporting the mixed conditions studies different details are provided- the first example provides a narrative description of the three groups this is not consistently provided. even the type of prescribing is not reported consistently	Thank you for your feedback. We agree and have removed the narrative description of the three groups to improved consistency of reporting throughout the results section.
Discussion	15. Our included studies only investigate pharmacist prescribing in adults, no studies investigating paediatric populations met our inclusion criteria- I just wonder if paediatric had been included as a search term if this would have found studies in that population?	Thank you for this comment.<br>We did not include the term "paediatric" because the search strategy was intentionally population-inclusive. No age limits were applied, and the search focused on pharmacist prescribing concepts. Therefore, studies involving children would have been retrieved if they met the intervention criteria.<br>Adding paediatric-specific terms would have narrowed the search and risked excluding mixed-population studies. On review of screening records and included studies, no eligible paediatric pharmacist prescribing studies were missed.<br>The search strategy was also peer-reviewed according to the PRESS guidelines, providing additional assurance of its comprehensiveness and methodological rigour.
	16. The Discussion should be more nuanced and recognise the limitations to the conclusions imposed by the weakness of the studies despite the overall trend being in favour of IP. Again as per above because there is no stratification of findings by type of prescribing intervention the implications for future policy and practice are unclear.	• The following text has been included in the discussion section recognising the limitations of the work<br>*A methodological limitation of this study was the lack of independent data extraction. Additionally three of the included studies are dated pre-2000, limiting their relevance to modern health systems [49,53,59].'*<br>*'Our evidence base was characterised by several methodological limitations, including a predominance of observational designs and high risk of bias, particularly due to confounding in non-randomised studies. Consequently, the findings demonstrate a consistent positive trend but do not provide strong causal evidence.'*<br>*'Although studies generally specified the type of prescribing authority, heterogeneity in study design, clinical context, outcomes measured, and implementation models limited the feasibility of stratified synthesis by prescribing type. As a result, the implications for policy and*

		practice should be considered in light of this variability. Future research using robust comparative designs within clearly defined prescribing models would strengthen the evidence base and support more precise policy conclusions.'<br>• We have included a graph in Appendix P illustrating the breakdown of reported outcomes direction of effect in Appendix P. The following text has been included in the main text:<br>*'Overall, the majority of reported outcomes showed no significant difference, or significantly improved outcomes in pharmacist prescribing compared with medical prescribing across all models of prescriptive authority (see Appendix P).'*

Reviewer 2

Reviewer 2	Comments	Author responses
Overall comment	This was an excellently written paper on an important topic in modern health care policy and systems. The authors should be commended for a high quality study that covers a diverse literature base and for writing a well structured manuscript. My comments on this paper are generally minor (although some are more significant) and they generally relate to the need to provide additional detail or justifications for the reader to fully understand and process the data.	Many thanks to the reviewer for your comments and review of this manuscript. We have responded to each of your specific comments below.
Abstract	- please add the search date range<br>- please add n=x for each setting<br>- please add a sentence summarising country of origin for the data	Thank you for your feedback, the following information has been included in the abstract:<br>*'Database searches were conducted from database inception to 29 January 2025.'*<br>*'Healthcare settings included outpatient (n=14), primary care (n=10), community pharmacy (n=6), inpatient (n=5), emergency department (n=1), and long-term care (n=3).'*<br>*'These studies were based in the USA (n=26), Canada (n=5), UK (n=4), Australia (n=2), and Singapore (n=2).'*
Introduction	- line 7,12,13: these sentences imply non medical prescribers are better but is it not the purpose of the review to establish this? Perhaps subtle wording changes might help clarify this point.	Thank you for your comment. We have made edits throughout the introduction to provide a more neutral framing of non-medical prescribing as a policy response, rather than implying superiority over medical prescribers.<br>*'Prescribing by healthcare professionals, other than medical doctors and dentists, has been introduced in many jurisdictions as a strategy to address workforce pressures and improve access to medicines.'*

		We also state the finding of the most recent Cochrane review apply to non-medical prescribing more broadly and are not specific to pharmacist prescribing).<br><br>*'A Cochrane review in 2018 reported that non-medical prescribers achieve outcomes comparable to medical prescribers across various health metrics, including systolic blood pressure, glycated haemoglobin, low-density lipoprotein cholesterol levels, medication adherence, patient satisfaction, and health-related quality of life [6]. However, much of the synthesised evidence was not specific to pharmacists, and substantial heterogeneity existed across professional groups and prescribing models.'*
	- line 37 onward. As above, the way it is worded here currently, it feels like a review of the evidence isn't actually needed? Can the authors please review and clarify in the text the position of the paper in the context of existing literature	Thank you for your comment. We have revised the introduction to more clearly position this review within the existing literature. We distinguish between broader systematic reviews of non-medical prescribing and reviews limited to particular pharmacy services or settings. We state that no previous review has synthesised evidence on the effectiveness and safety of pharmacist prescribing across multiple healthcare settings and prescribing models. We also highlight variation in international scope of practice and ongoing policy reforms to clarify the relevance and added value of the present review.<br><br>*'From a health system perspective, introducing pharmacist prescribing may have beneficial economic implications for healthcare systems [15,16]'.'*<br><br>*'Some studies have reported associations between pharmacist involvement in prescribing and reductions in medication errors and inappropriate prescribing, which may translate into downstream cost implications [16–18]'*<br><br>*'However, these reviews were limited to specific settings or service models and did not synthesis evidence across multiple healthcare settings.'*<br><br>*'Given international variation in prescribing models and ongoing policy reforms, a comprehensive synthesis of the evidence specific to pharmacist prescribing is warranted.'*
Methods	- the cost-effectiveness study should be referenced if finished or say it is underway (as per the discussion)	Thank you for your comment. The following text has been included in the manuscript.<br><br>*'A separate search was conducted on cost-effectiveness as part of this protocol; these findings are currently being prepared for publication by the authors.'*

	- line 24. Please clarify in the text if authors were contacted for additional information.	Thank you for your comment. The following text has been included in the manuscript:<br>*'Authors of included studies were not contacted for additional information.'*
	- when the authors say that a clear statement of pharmacists prescriptive authority was needed, can they explain a little more what they meant in the text to make this as transparent as possible. For example were definitions needed or did the authors interpret the type of prescribing based on any text given as long as there was enough to use for that purpose.	The following text has been added to the manuscript:<br>*'A clear statement of pharmacists' prescriptive authority, explicitly reporting that pharmacists prescribed, was required for study inclusion. In studies involving collaborative or dependent prescribing, a description of the role of other healthcare professionals in the prescribing process was also required.'*
Findings	- table 1. Please can the authors clarify in the table whether adverse drug events that were not serious were included, and if not why not. Please can they also do the same for preventable adverse drug events (ie not due to ADRs).	There was a typo in this text. Thank you for pointing it out. We included all patient adverse events.<br>As with the preceding lists, the bullet points are intended to provide context rather than be comprehensive. The following text is now included in Table 1.<br>'Patient adverse events including:<br>• drug-related hospital admissions<br>• serious adverse drug reactions.'
	- line 53-56. Please add n=x for each source of bias listed, and consider adding top 3 sources for each design.	Thank you for your comment, we have added n=x for each source of bias listed.<br>We have not summarised the top three sources of bias for each study design. Ranking domains based on frequency could be misleading. A domain rated as 'some concerns' across many outcomes does not necessarily represent a greater limitation than a domain rated as 'high risk' in fewer outcomes. Frequency does not reflect severity.<br>We prefer the reader refer to Figures 3-5 for the full outcome-level domain assessments, which transparently present both the distribution and severity of bias across all included study outcomes, or Appendices J-L which provide the raw breakdowns per outcome.
	- there is much use of the word 'significant' referring to results. Whilst the tables 2-3 showcase the reason why (referring to statistical significance), this isn't immediately apparent and a statement in the methods referring to the use of this term to indicate statistical significance of at least p<0.05 would help the reader with interpretation	Thank you for your comment. The following text has been added to the manuscript:<br>'A p-value <0.05 was considered statistically significant, this was recorded as 'significant' in the findings sections.'
	- page 7 line 32. '… was reported in the pharmacist prescribing group compared…'	Addressed
	- page 7 line48: '… significance of this finding…'	Addressed

	- page 7 line 56: please can the authors describe deprescribing of what exactly in the text	Thank you for your comment.<br>We have specified ‘aspirin deprescribing’ in the manuscript.
	- page 8 line 8: ‘…goal…’	Addressed
	- tables 2/3. Please include study duration and year of publication in the tables. This will help to understand how many older studies there were	As per another peer reviewers request, Tables 2 and 3 are now Appendices N and O. Study year has been included in these tables.<br>Full data extraction forms including study duration are now included in Appendix D.
	- related to the above point, the authors need to include the full database search date duration in the methods and justify why pre-2000 studies were included - they are unlikely to represent the modern health care era and could distort the results. This should also be acknowledged as a limitation of the study later in the discussion section.	• It is generally not recommended to include a date limit in full systematic reviews to minimise the risk of missing relevant studies. The search strategy was developed by an experienced information specialist, and peer reviewed by another experienced information specialist. The following text has been added to the manuscript: *‘Database searches were conducted from database inception to 29 January 2026. No date limit was included to minimise the risk of missing relevant studies.’*<br>• Your point regarding lack of applicability of studies dated pre-2000 to modern health systems is very valid. For transparency we have identified three studies dated pre-2000 and included the following text in the discussion section: *‘Additionally three of the included studies are dated pre-2000, limiting their relevance to modern health systems (40,44,50).’*
	- in the results can the authors please comment on the value of adding some text concerning comparisons between outcome data and type of prescribing model. It would be interesting to see whether this factor was associated with neutral or positive outcomes for pharmacists. However I understand this might not be practical given the smaller number of studies in certain model groups and so this isn’t a major point.	We have included a graph in Appendix P illustrating the breakdown of reported outcomes direction of effect in Appendix P. The following text has been included in the main text:<br>*‘Overall, the majority of reported outcomes showed no significant difference, or significantly improved outcomes in pharmacist prescribing compared with medical prescribing across all models of prescriptive authority (see Appendix P).’*
	- p6 line 15-20. Please can the authors include in the text a sentence describing how many studies compared pharmacist prescribing to medical prescribing and how many to nurse prescribing.	The following text has been included in the main text:<br>*‘Comparator groups included medical prescribing (n=38) (24–35,37–44,46–54,56,57,59–65) and nurse prescribing (n=1) (45)’*
	- p10 line 4 and 12. ‘… pharmacist prescribing group compared…’	Addressed

	- p10 line 30. The word 'similar' appears twice , please can the authors review	Addressed
	- p10 line 45-51. '… in pharmacist prescribing groups…' or similar wording change for completeness	Addressed
	- p11 line 39-42. Hypotension events were reported as increased and had no significant difference for the same study , can the authors please review	This was a typo and has been corrected in the main text. The author reported significantly higher hypotension events in pharmacist prescribing compared with medical prescribing.
	- please can the authors include a table summarising the main outcome measures used in the included studies, and report the top 3 categories for effectiveness and safety in the text.	A table summarising outcome domains reported across effectiveness and safety has been included in Appendix Q. The following text has been included in the manuscript:<br>*'The most frequently reported outcome domains were blood pressure, healthcare utilisation, and blood sugar (Appendix Q).'*<br>*'The most frequently reported outcome domains were adverse events, prescribing quality, and hospitalisations/emergency department visits due to adverse events (Appendix Q).'*
Discussion	- the study limitations should be expanded to include a lack of independent data data extraction by the authors , and the use of evidence pre dating the modern health care era.	The following text has been added to the discussion:<br>'A methodological limitation of this study was the lack of independent data extraction. Additionally three of the included studies are dated pre-2000, limiting their relevance to modern health systems [49,53,59].'

Reviewer 3

Reviewer 3		
Overall comment	The manuscript addresses an important and timely topic and demonstrates a substantial research effort. The compilation of relevant studies is valuable; however, the paper faces significant challenges in scope, clarity, and interpretation. Assessing safety and efficacy—or comparing these outcomes with medical prescribing—across diverse conditions, settings, and prescribing models is inherently complex. These conditions are not been fully explored by the authors. At present, the evidence base does not support definitive conclusions.<br>Strengths<br>• The topic is of high relevance to readers and stakeholders.	Many thanks to the reviewer for their review of our manuscript and comments. We have provided a response to each of your specific comments below.

	• The authors have undertaken a comprehensive literature search.<br><br>• The narrative review approach is appropriate given the limited quantity and quality of available studies.<br><br>• Data extraction is comprehensive<br><br><br>Recommendation<br><br>Major revisions required. The manuscript should be reconsidered after substantial restructuring, clarification of definitions, improved data presentation, and deeper interpretation of findings.	
Introduction	1. Scope and Definitions<br>o Indicators of "efficacy" and "safety," which are central to the review, are not<br><br>defined in the Methods section.<br><br>o The paper adopts a specific definition of pharmacist prescribing without<br><br>justification, despite ongoing debate in the field.	The following effectiveness and safety definition has been included:<br><br>*'Effectiveness is defined as the extent to which pharmacist prescribing produces the intended outcomes in clinical practice [25]. Safety is defined as the occurrence and prevention of adverse effects and reduction of risk of unnecessary harm associated with pharmacist prescribing [26,27].'*<br><br>The following text has been added to the introduction section:<br><br>*'This review specifically focuses on prescribing by pharmacists. Pharmacists' scope of prescriptive authority varies internationally and formal definitions vary. Some frameworks and policy documents classify pharmacist prescribing only independent or dependent (with dependent prescribing including collaborative approaches) (6,7) , whereas others provide distinct classifications for independent, collaborative, and dependent pharmacist prescribing (8). In this paper we have categorised pharmacist prescribing into three models: independent prescribing, collaborative prescribing, and dependent prescribing (Figure 1) to better reflect variation in prescriptive authority across studies.'*
	• Line 10: Reference 1 pertains only to hospital prescribing. Amend or add evidence from other settings.	We have added three additional references to better support our statement across a wider range of healthcare settings.<br><br>• Stewart D, Jebara T, Cunningham S, Awaisu A, Pallivalapila A, MacLure K. Future perspectives on nonmedical prescribing. Therapeutic advances in drug safety. 2017;8(6):183–97. Located at: EPISTEMONIKOS. doi:10.1177/2042098617693546<br>• Nancarrow SA. Six principles to enhance health workforce flexibility. Hum Resour Health. 2015 Dec;13(1):9. doi:10.1186/1478-4491-13-9<br>• Noblet T, Marriott J, Graham-Clarke E, Shirley D, Rushton A. Clinical and cost-effectiveness of non-medical prescribing: a systematic review of randomised controlled trials. PLOS ONE. 2018 Mar

		6;13(3):e0193286. doi:10.1371/journal.pone.0193286<br>• World Health Orgaisation. (2008). *Task Shifting Global Recommendations and Guidelines*.
	• Line 21: Clarify the claim regarding nonmedical prescribing in 2016 and provide supporting evidence.	Additional text has been including to clarify this statement refers to non-medical prescribers.<br>*'A Cochrane review in 2018 reported that non-medical other prescriber s achieve outcomes comparable to medical prescribers across various health metrics, including systolic blood pressure, glycated haemoglobin, low-density lipoprotein cholesterol levels, medication adherence, patient satisfaction, and health-related quality of life [6]'*
	• Lines 22–34: Justify the choice of definitions for pharmacist prescribing.	The following text has been added to the introduction section:<br>*'This review specifically focuses on prescribing by pharmacists. Pharmacists' scope of prescriptive authority varies internationally and formal definitions vary. Some frameworks and policy documents classify pharmacist prescribing only independent or dependent (with dependent prescribing including collaborative approaches) (6,7) , whereas others provide distinct classifications for independent, collaborative, and dependent pharmacist prescribing (8). In this paper we have categorised pharmacist prescribing into three models: independent prescribing, collaborative prescribing, and dependent prescribing (Figure 1) to better reflect variation in prescriptive authority across studies.'*
	• Lines 37–39: Define cost-effectiveness and consider limiting the claim.	We have rephrased this sentence and limited the our claims regarding cost avoidance and cost savings.<br>*'From a health system perspective, introducing pharmacist prescribing may have beneficial economic implications for healthcare systems'*
	• Lines 49–51: Clarify differences between minor ailments and pharmacist prescribing.	In this context, the minor management ailment schemes refer to pharmacist prescribing for minor ailments. We have rephrased the text to clarify our meaning.<br>*'We identified two systematic reviews on pharmacist prescribing in hospital settings [1] and pharmacist prescribing for minor ailments [20].'*
	• Lines 55–58: Discuss variability in prescriptive authority and its potential impact.	We did not identify any existing literature directly comparing the effectiveness or safety of different prescriptive authority models. We have added the following text to the discussion section to address your comment.<br>*'Although studies generally specified the type of prescribing authority, heterogeneity in study design, clinical context, outcomes measured, and implementation models limited the feasibility of stratified synthesis by prescribing type. As a result, the implications for policy and practice should be considered in light of this variability. Future research using robust comparative designs within clearly defined prescribing models would strengthen the evidence base and support more precise policy conclusions.'*

Methods	• Methods are generally robust; however, include TIDieR checklist for intervention details.	Many thanks for your comment, we agree that not providing study details limits clarity for the reader. Subsequently we have included the full data extraction for each study in the Appendix D. This includes all intervention details and associated page numbers from the original papers.<br><br>We did not include as TIDieR checklist as this tool has been developed for primary study authors to report intervention details, and has not been developed for systematic review reporting.
Findings	2. Presentation of Data<br><br>o Tables are excessively long and difficult to interpret (e.g., Table 2 spans six pages).<br><br>o Consider alternative formats such as data visualization tools (e.g., Tableau) to improve clarity.	Many thanks to the reviewer for suggesting Tableau, we have included a bubble chart for effectiveness and safety outcomes in the results section. Tables 2 and 3 have been moved to Appendices N and O section to improve readability and interpretability.
	o Outcomes are inconsistently reported (e.g., mean change vs. goal achieved).	The outcomes measures we have reported directly reflect the outcomes reported by the original study authors. We have followed Cochrane guidance on this matter to avoid selective reporting of outcomes.<br><br>To improve interpretation for readers we have categorised each outcome measure within a broader outcome domain. Outcome domains associated with each outcome measure are included in Appendices N and O in the appendices
	o Study design is not systematically organized.	We have moved the original tables 2 and 3 to Appendices N and O. Within both tables data has been ordered as follows: 1. Healthcare population, 2. outcome domain (grouped together under 1), 3. study design (study designs grouped together under 1 and 2).
	Reorganize tables by defined indicators.	We have moved the original tables 2 and 3 to Appendices N and O. Within both tables data has been ordered as follows: 1. Healthcare population, 2. outcome domain (grouped together under 1), 3. study design (study designs grouped together under 1 and 2).
	Remove duplication and ensure consistent outcome reporting	We have followed Cochrane guidance on this matter to avoid selective reporting of outcomes. Subsequently we cannot remove any outcome which have been reported using different outcome measurements.<br><br>To improve interpretation for readers we have categorised each outcome measure within a broader outcome domain. Outcome domains associated with each outcome measure are included in Appendices N and O.
	Present in a formant which is more concise yet interpretable.	We have included Figures 3 and 4 to make the key findings more interpretable. We have moved tables 2 and 3 to the appendices section.
Discussion	3. Interpretation<br><br>The discussion section is superficial, largely reiterating results without meaningful<br><br>analysis.	The following text has been included in the discussion section to provide a more critical interpretation of our findings:<br><br>*'A methodological limitation of this study was the lack of independent data extraction. Additionally three of the included studies are dated pre-2000, limiting their relevance to modern health systems [49,53,59].'*
	Factors influencing safety and efficacy (e.g., prescribing model, prescriptive	

	authority, patient cohort, setting) are not adequately considered.<br><br>Expand interpretation beyond summary of results.<br><br>Address contextual factors influencing safety and efficacy.	*'Our evidence base was characterised by several methodological limitations, including a predominance of observational designs and high risk of bias, particularly due to confounding in non-randomised studies. Consequently, the findings demonstrate a consistent positive trend but do not provide strong causal evidence.'*<br><br>*'Although studies generally specified the type of prescribing authority, heterogeneity in study design, clinical context, outcomes measured, and implementation models limited the feasibility of stratified synthesis by prescribing type. As a result, the implications for policy and practice should be considered in light of this variability. Future research using robust comparative designs within clearly defined prescribing models would strengthen the evidence base and support more precise policy conclusions.'*
Appendices	4. Supporting Information<br><br>o Absence of a TIDieR checklist limits clarity on intervention details.<br><br>o Readers must consult original papers to understand interventions fully.	Many thanks to the review for their comments. We agree the absence of full intervention details limited clarity for readers and thank the reviewer for their comments.<br><br>The TIDieR checklist is a very useful tool for the original primary paper authors to describe their intervention. However, it omits certain details of the included studies which we believe are also required by readers to understand the studies fully. It has not been developed for systematic review reporting.<br><br>In Appendix D, we have included a more comprehensive account of each study's details including intervention, comparator, population, setting, outcomes, funding and ethics in our appendices (with page number location in the original paper) to allow for readers to fully understand each study. We hope this meets the reviewers comment comprehensively and increases clarity on all included study details.

VERSION 2 - REVIEW

Reviewer 3

Name Benrimoj, Shalom

Affiliation University of Granada Faculty of Pharmacy, Faculty of Pharmacy

Date 11-Apr-2026

COI

the paper is vastly improved form the previous version . I still think that the implications of the work for example how do different models of prescribing affect outcomes have not be fully addressed. Equally the legislative authority's associated with the country specific ie

protocols/ national guidelines have not been discussed. However the authors have undertaken a very large tasks and the at this stage i would recommend publication as is.

VERSION 2 - AUTHOR RESPONSE

Peer reviewer 3	Author response
I still think that the implications of the work for example how do different models of prescribing affect outcomes have not be fully addressed. Equally the legislative authority's associated with the country specific ie protocols/ national guidelines have not been discussed.	Many thanks to the peer reviewer for their comprehensive review. We have added the following text to the discussion section to address the comments:<br><br>'Overall, most reported outcomes showed either no significant difference or improved outcomes for pharmacist prescribing compared with medical prescribing across models of prescriptive authority. However, not all models have been evaluated across all health populations or healthcare settings; for example, no studies of independent prescribing were conducted in outpatient settings, and no CPA studies were based in community settings. Subsequently, it is not possible to determine a single 'best' model, and different models of prescriptive authority may be more appropriate depending on the healthcare setting and population. In addition, variations in country-specific legislative frameworks, protocols, and national guidelines were not examined in detail across the included studies and may influence the implementation and effectiveness of different models.'
